# Assessing causal associations between neurodegenerative diseases and neurological tumors with biological aging: a bidirectional Mendelian randomization study

**DOI:** 10.3389/fnins.2023.1321246

**Published:** 2023-12-19

**Authors:** Zhiyun Zhang, Ningfang Liu, Xuyang Pan, Chuyi Zhang, Yifan Yang, Xinyun Li, Ying Shao

**Affiliations:** ^1^Department of Plastic and Reconstructive Surgery, The First Hospital of Jilin University, Changchun, China; ^2^Department of Anesthesiology, The First Affiliated Hospital of Xiamen University, School of Medicine, Xiamen University, Xiamen, China; ^3^The Second Hospital of Jilin University, Changchun, Jilin, China; ^4^Infection Department, The First Bethune Hospital of Jilin University, Changchun, China

**Keywords:** neurodegenerative diseases, neurological tumors, biological aging, bidirectional Mendelian randomization study, genome-wide association study (GWAS)

## Abstract

**Background:**

Aging is a significant risk factor for many neurodegenerative diseases and neurological tumors. Previous studies indicate that the frailty index, facial aging, telomere length (TL), and epigenetic aging clock acceleration are commonly used biological aging proxy indicators. This study aims to comprehensively explore potential relationships between biological aging and neurodegenerative diseases and neurological tumors by integrating various biological aging proxy indicators, employing Mendelian randomization (MR) analysis.

**Methods:**

Two-sample bidirectional MR analyses were conducted using genome-wide association study (GWAS) data. Summary statistics for various neurodegenerative diseases and neurological tumors, along with biological aging proxy indicators, were obtained from extensive meta-analyses of GWAS. Genetic single-nucleotide polymorphisms (SNPs) associated with the exposures were used as instrumental variables, assessing causal relationships between three neurodegenerative diseases (Alzheimer’s disease, Parkinson’s disease, amyotrophic lateral sclerosis), two benign neurological tumors (vestibular schwannoma and meningioma), one malignant neurological tumor (glioma), and four biological aging indicators (frailty index, facial aging, TL, and epigenetic aging clock acceleration). Sensitivity analyses were also performed.

**Results:**

Our analysis revealed that genetically predicted longer TL reduces the risk of Alzheimer’s disease but increases the risk of vestibular schwannoma and glioma (All Glioma, GBM, non-GBM). In addition, there is a suggestive causal relationship between some diseases (PD and GBM) and DNA methylation GrimAge acceleration. Causal relationships between biological aging proxy indicators and other neurodegenerative diseases and neurological tumors were not observed.

**Conclusion:**

Building upon prior investigations into the causal relationships between telomeres and neurodegenerative diseases and neurological tumors, our study validates these findings using larger GWAS data and demonstrates, for the first time, that Parkinson’s disease and GBM may promote epigenetic age acceleration. Our research provides new insights and evidence into the causal relationships between biological aging and the risk of neurodegenerative diseases and neurological tumors.

## Introduction

As the global aging process continues to intensify, it is projected that the global elderly population will exceed 2 billion by 2050. Aging is associated with a variety of age-related health issues, among which the risks of neurodegenerative diseases and neurological tumors are particularly prominent, posing a significant threat to healthy life expectancy and quality of life. In older age groups, having a disease-free brain is a rare occurrence. Neurodegenerative diseases such as Alzheimer’s disease (AD), Parkinson’s disease (PD), and amyotrophic lateral sclerosis (ALS) are closely linked to aging, with their incidence sharply increasing with age (Hou et al., 2019). For instance, the incidence of AD almost doubles every 5 years after the age of 65, and by the ninth decade of life, approximately one in three adults meets the criteria for dementia (Alzheimer’s Association, 2015). Similarly, the incidence of PD steadily rises with age on a global scale (Bloem et al., 2021). Furthermore, the prevalence of ALS peaks around the age of 80 (Mehta et al., 2018). In addition to neurodegenerative diseases, in the realm of neurological tumors, particularly glioblastoma (GBM), age has been identified as a clear risk factor for both disease onset and prognosis (Thakkar et al., 2014). The incidence of GBM sharply increases after the age of 54, reaching its peak between the ages of 74 and 85 (Ostrom et al., 2017). However, chronological age alone cannot accurately gauge the extent of biological aging or predict the risks associated with these diseases. Therefore, the assessment of an individual’s biological age becomes paramount, as different individuals may exhibit variations in biological age at the same chronological age. When biological age surpasses chronological age, the body enters a state of accelerated aging, resulting in elevated disease risks and reduced quality of life (Jylhävä et al., 2017).

Over the years, researchers have been actively seeking reliable biomarkers to assess an individual’s biological age (Jylhävä et al., 2017). Among these, telomere length (TL) is a well-known biological aging marker closely associated with neurodegenerative diseases and neurological tumors (Hou et al., 2019; Saunders et al., 2022). Recently, Blanca et al. successfully demonstrated a causal relationship between shortened TL and an increased risk of AD using Mendelian randomization (MR) (Rodríguez-Fernández et al., 2022a). However, the relationship between TL and other neurodegenerative diseases such as PD and ALS remains unclear. Rodríguez-Fernández and colleagues found that, apart from its association with AD, there is no causal relationship between the length of TL and the risk of other neurodegenerative diseases. Similarly, Chen and colleagues also did not find a causal relationship between TL and the onset of PD (Chen and Zhan, 2021; Rodríguez-Fernández et al., 2022b). Additionally, there is evidence indicating a significant genetic association between leukocyte TL (LTL) increase and glioma risk (Saunders et al., 2022). These findings appear contradictory to the notion that aging increases the risk of neurodegenerative diseases and neurological tumors. Furthermore, these studies have not validated the reverse causal relationship between aging and neurodegenerative diseases or neurological tumors. The true nature of the relationship between these factors remains a subject of considerable controversy. This prompts us to further investigate the intricate relationship between biological aging and the risk of neurodegenerative diseases and neurological tumors.

Therefore, this study aims to comprehensively explore the potential relationships between biological aging and neurodegenerative diseases as well as neurological tumors by integrating multiple biological age proxy indicators (Yu et al., 2020; Duan et al., 2022; Chen et al., 2023). These indicators include molecular biomarkers such as TL and DNA methylation epigenetic age acceleration and phenotypic biomarkers such as frailty index and facial visual aging. Notably, we have selected the latest generation of epigenetic clock acceleration, GrimAge Acceleration, as one of the biological age proxy indicators. Epigenetic age acceleration, where an individual’s biological age exceeds their chronological age, has been associated with increased mortality and the risk of age-related diseases, including cancer (Yu et al., 2020). Furthermore, GrimAge utilizes a DNA methylation pattern at specific CpG sites to predict biological age and is considered one of the most robust methods for assessing biological age (Duan et al., 2022). Distinguishing itself from other Epigenetic Clocks, GrimAge stands out for its predictive capabilities of health outcomes and lifespan. GrimAge incorporates data from 1,030 CpGs associated with smoking pack-years and seven plasma proteins (cystatin C, leptin, tissue inhibitor of metalloproteinases 1, adrenomedullin, β-2 microglobulin, growth differentiation factor 15, and plasminogen activator inhibitor 1) (Lu et al., 2019). In various disease contexts, epigenetic age has been found to be greater than chronological age, while in long-lived populations, it tends to be lower than chronological age, providing strong evidence for the reflection of biological age by epigenetic age (Jylhävä et al., 2017; Yu et al., 2020; Tang et al., 2022). Through this multidimensional research approach, we aim to gain a deeper understanding of the relationship between biological age and the risk of neurodegenerative diseases as well as brain tumors, providing a scientific basis for future intervention strategies.

MR is an increasingly popular and effective causal inference method in recent years (Weith and Beyer, 2023). It employs genetic variation (single-nucleotide polymorphisms, SNPs) as instrumental variables (IVs) to infer causal relationships between exposures and outcomes, effectively circumventing confounding biases present in traditional epidemiological studies (Birney, 2022). MR analysis reduces confounding and reverse causality due to the segregation and independent assortment of genes passed from parents to offspring. In the absence of horizontal pleiotropy (i.e., genetic variants being independently associated with the putative exposure and the putative outcome) and population stratification, MR can provide clear estimates of disease risk (Bowden and Holmes, 2019).

In this study, we adopted a two-sample and bidirectional MR analysis aiming to assess the causal relationships between three neurodegenerative diseases (Alzheimer’s disease, Parkinson’s disease, amyotrophic lateral sclerosis), two benign neurological tumors (vestibular schwannoma and meningioma), and one malignant neurological tumor (glioma) with four biological age proxies (frailty index, facial aging, TL, and epigenetic aging clock acceleration). Previous research has conducted some MR analyses on the associations between AD, PD, ALS, glioma, meningioma, and TL (Chen and Zhan, 2021; Saunders et al., 2022; Rodríguez-Fernández et al., 2022a,b; Yu et al., 2023), as well as AD and frailty index using MR analysis (Liu et al., 2022). However, some of the findings from these studies are in partial contradiction to the notion that aging is a crucial risk factor for neurodegenerative diseases and the development of neurological tumors. Notably, to date, there has been no MR causal inference analysis conducted on neurodegenerative diseases, benign and malignant neurological tumors, in relation to frailty index and epigenetic aging clock acceleration. Therefore, this study, for the first time, incorporates a variety of biological aging proxy indicators, with special attention to the epigenetic aging clock acceleration. We also employ larger sample GWAS data in the hope of ultimately elucidating the direction and magnitude of the causal relationships between biological aging and the risk of neurodegenerative diseases and neurological tumors, providing new insights and understanding to this field of research.

## Methods

### Data sources

#### Neurodegenerative disease

For the investigation of AD, we utilized recently published summary statistics data from the GWAS Catalog (Schwartzentruber et al., 2021). This comprehensive meta-analysis data pertains to a large-scale GWAS conducted on European populations, incorporating data from the UK Biobank (53,042 cases and 355,900 controls), the AD GWAS meta-analysis by Kunkle et al. (21,982 cases and 41,944 controls), the GR@ACE project (4,120 cases and 3,289 controls), and the FinnGen biobank (3,697 cases and 131,941 controls), among others. These datasets ultimately unveiled 13 risk loci (*p*-value <5 × 10^–8^), including 10 loci previously reported in studies. Genome-wide association study (GWAS) summary statistics data for PD patients were obtained from the International Parkinson’s Disease Genomics Consortium,^1^ encompassing 33,674 cases and 449,056 controls of European descent (Nalls et al., 2019). Large-scale European ancestry ALS GWAS summary data, including 12,577 ALS patients and 23,475 controls, were acquired from a recent study (van Rheenen et al., 2016). All patients were diagnosed by specialized neurologists following the (revised) El Escorial criteria.

#### Benign neurological tumor

To obtain GWAS summary statistics data for vestibular schwannoma, we retrieved data from Wouter et al., who conducted a GWAS using 911 sporadic vestibular schwannoma cases from the Type 2 Neurofibromatosis Gene Testing Service in Northwest England and 5,500 control samples from the UK Biobank resource (Sadler et al., 2023). Summary statistics data for meningioma were obtained from the UK Biobank, comprising 307 cases and 456,041 controls of European ancestry. The data were analyzed using the fastGWA-GLMM method with adjustments for relevant variables (Jiang et al., 2021).

#### Malignant neurological tumor

The glioma GWAS data were sourced from a recent meta-analysis of 12,488 glioma cases and 18,169 control samples of European ancestry available on the European Genome-Phenome Archive (EGA). Gliomas encompass various subtypes, some of which are defined by their malignant grade (e.g., pilocytic astrocytoma - World Health Organization [WHO] grade I, diffuse “low-grade” glioma - WHO grade II, anaplastic glioma - WHO grade III, glioblastoma multiforme [GBM] - WHO grade IV). In this study, gliomas were categorized into two subtypes: GBM (*n* = 6,183) and non-GBM (*n* = 5,820) (Melin et al., 2017).

#### Molecular aging biomarkers

We utilized the open GWAS^2^ database, which is the largest repository of genetic variation to date. This database comprises a sizable population-based cohort collected by the UK Biobank between 2006 and 2010, with participants aged between 40 and 69 years. These individuals underwent comprehensive profiling through questionnaires, physical examinations, plasma biomarkers, whole-genome analyses, and other investigations. Codd et al. (2022) conducted an analysis of 489,092 peripheral blood leukocyte DNA samples obtained from the UK Biobank, reporting measurements and initial characterizations of LTL for 472,174 UK Biobank participants. The GWAS summary statistics for genetic association estimates of epigenetic age acceleration measures, specifically GrimAge, were derived from a recent meta-analysis of biological aging, encompassing 34,467 participants of European ancestry. Among the participants included in the analysis from 28 European ancestry studies, 57.3% were female. Detailed descriptions of the methods employed can be found in McCartney et al.’s publication (McCartney et al., 2021).

#### Phenotypic aging biomarkers

A questionnaire-based survey was conducted to investigate non-subjective perception of facial aging and explore the relationship between participants’ biological age and their subjectively perceived age. A total of 8,630 participants reported appearing older than their actual age, 103,300 participants reported appearing their actual age, and 312,062 participants reported appearing younger. These observations were made by independent third parties unaware of the participants’ actual ages. Participants were coded as 1 for appearing younger, 0 for appearing older, and 0.5 for appearing their actual age. Subsequently, mixed-effects linear models were employed, considering covariates such as age, gender, and study participation center, to transform perceived age (FA) into an ordered categorical variable. Log odds ratios (OR) were derived from linear scale statistical data using a Taylor expansion series, where an OR > 1 indicates a greater chance of appearing younger (Jiang et al., 2021). The study also associated frailty index (FI) and genetic variants, sourced from a GWAS meta-analysis of 164,610 UK Biobank participants and 10,616 TwinGene participants. The frailty index is based on an accumulation of deficits model, where each individual’s FI is calculated as the number of deficits they possess divided by a total of 49 possible deficits. Results revealed that the average deficit proportion for UK Biobank participants was 0.129 ± 0.075, while TwinGene participants exhibited an average deficit proportion of 0.121 ± 0.080 (Atkins et al., 2021).

### MR design

We conducted a two-sample bidirectional MR study based on extensive GWAS research. Specifically, we incorporated four biological aging proxy indicators, including molecular biomarkers (such as TL and DNA methylation epigenetic age) and phenotypic biomarkers (such as frailty index and facial visual aging), to investigate the causal relationship between chronological aging and age-related neurodegenerative diseases (including AD, PD, and ALS) as well as benign and malignant neurological tumors (vestibular schwannoma, meningioma, and glioblastoma).

Reliable MR analysis requires adherence to three core assumptions: (1) genetic variants are strongly associated with the exposure factor; (2) genetic variants are independent of any potential confounding factors; (3) genetic variants are independent of the outcome and affect the outcome solely through the exposure factor. Additionally, certain other assumptions need to be met, including the absence of linear relationships and statistical interactions (Birney, 2022). Furthermore, we selected single-nucleotide polymorphism (SNP) sites that demonstrated a genome-wide significance level (*p*-value <5 × 10^−8^). However, due to the limited sample sizes in the GWAS summary statistics for meningioma and vestibular schwannoma, we relaxed the genome-wide significance levels for both to identify an adequate number of SNPs for causal relationship inference (*p*-value <5 × 10^−6^). If there is linkage disequilibrium (LD) present in the single nucleotide polymorphisms (SNPs) of the genetic instrumental variable, it could lead to misleading results. To mitigate this impact, we employed the clustering procedure within the two-sample MR package, clustering SNPs based on their LD relationships within a given genomic region. In this clustering process, we utilized a threshold of *r*^2^ < 0.001 and a window size of 10,000 kb to identify independent SNPs. Additionally, we calculated the phenotype variance explained by the genetic instrumental variables (*R*^2^) and the F-statistics of these variable regression analyses to assess the reliability of these genetic instrumental variable SNPs. The formulas for calculating *R*^2^ and F-statistics are as follows: $R^{2}=2\times \left(1-MAF\right)\times MAF\times \frac{BETA}{SE\times \sqrt{N}}$ and $F=\frac{N-K-1}{K}\times \frac{R^{2}}{1-R^{2}}$ , where MAF denotes the minor allele frequency for the SNP, BETA represents the magnitude of the SNP’s impact on the phenotype, SE represents the standard error of the SNP’s impact on the phenotype, N denotes the sample size of the GWAS, and K represents the number of SNPs selected for MR analysis after filtering. SNPs with strong instrumentation were identified as having an F-statistic > 10 (Lawlor et al., 2008).

### Statistical analysis

We initiated our analysis by assessing the causality of each SNP through the application of the Wald ratio. In instances where more than one SNP could potentially be employed as an instrumental variable, we utilized the inverse variance weighted (IVW) method to conduct a meta-analysis of Wald estimates. The meta-analysis of Wald estimates for each individual SNP was computed using the IVW method in the following formulas: $\beta =\sum X_{k}Y_{k}\sigma_{Y_{k}}^{-2}/\sum X_{k}^{2}\sigma_{Y_{k}}^{-2}$ with σMR=1∑Xk2σYk−2 where *X*_k_ represents the association of SNP_k_ with the exposure, and *Y*_k_ corresponds to the association of SNP_k_ with the outcome, both accompanied by their respective standard errors. IVW is recognized as the most robust method with the highest statistical power available, although it assumes the effectiveness of all instrumental covariates and may deviate when the mean multifactor effect deviates from zero. Furthermore, we complemented our analysis with the use of MR-Egger and weighted median methods alongside IVW (Atkins et al., 2021). The weighted median method yields consistent causal estimates under the assumption that at least 50% of SNPs are effective. In cases of substantial heterogeneity, we applied a random effects model.

Furthermore, we executed MR-Egger intercept analysis (Bowden et al., 2015) and MR-PRESSO (Verbanck et al., 2018) tests to scrutinize the potential presence of horizontal pleiotropy and outlier SNPs in our study. A MR-Egger intercept value of p exceeding 0.05 signifies the absence of horizontal pleiotropic effects. In cases where we detected outliers, we reported the MR causal estimate recalculated using the MR-PRESSO method as our primary outcome; otherwise, we relied on the IVW method. To ensure the resilience of our MR analysis, we harnessed Cochran Q statistics to gauge heterogeneity among SNPs (Hemani et al., 2018). To pinpoint possibly influential SNPs, we conducted a “leave-one-out” sensitivity analysis, systematically excluding one SNP at a time and performing an IVW-random method on the remaining SNPs to assess the potential impact of outlying variants on our estimates (Supplementary Figures S1–S65). Forest and scatter plots were generated for further scrutiny of heterogeneity. To rectify the bias from multiple comparisons, we used a Benjamini–Hochberg false discovery rate (FDR). A causal relationship was concluded if the direction and estimates of the causal effects of the IVW and weighted median methods were consistent and the *p* value with the FDR was less than 0.05 after correction for heterogeneity and horizontal polymorphism. A *p* < 0.05 but with an FDR >0.05 was interpreted as a suggestive causal relationship. Our analysis was conducted utilizing the “Two-Sample MR” and “MR-PRESSO” packages within R 4.2.3 software.

## Results

The sources, sample sizes, and population information for the GWAS summary statistics data used in our study are presented in Table 1. Following the selection of instrumental variables, the number of SNPs used for two-sample bidirectional MR analyses ranged from 7 to 144, with the explained variance (*R*^2^) ranging from 0.19 to 22.8% (Supplementary Tables S3–S14). Additionally, after calculating the F-statistics, values ranged from 36.05 to 712.83, indicating sufficient instrument strength and mitigating the risk of weak instrument bias (F-statistics >10) (Lawlor et al., 2008).

**Table 1:** Data sources used in the Mendelian randomization for the current study.

Phenotype				Source	PMID	Total or cases/controls	Ancestry
Neurodegenerative diseases		Alzheimer’s disease		GWAS Catalog	33,589,840	75,024/397,844	European
		Parkinson’s disease		International Parkinson’s Disease Genomics Consortium	31,701,892	33,674/ 449,056	European
		Amyotrophic lateral sclerosis		Project MinE	27,455,348	12,577/ 23,475	European
Benign brain tumor		Vestibular schwannomas		GWAS Catalog	36,546,557	911/5,500	European
		Meningioma		GWAS Catalog	34,737,426	307 /456,041	European
Malignant brain tumor		All-glioma		European genome-phenome archive (EGA)	28,346,443	12,488/18,169	European
		GBM				6,183/18,169	
		Non-GBM				5,820/18,169	
Biological aging proxy indicators	Molecular biomarkers	Telomere length		MRC-IEU	37,117,760	472,174	European
		Epigenetic aging clock	DNA methylation Hannum age	GWAS catalog	34,187,551	34,449	European
	Phenotypic biomarkers	Frailty index		MRC-IEU	34,431,594	175,226	European
		Facial aging		UK Biobank	31,768,069	423,999	European

### Neurodegenerative disease

#### Alzheimer’s disease

AD to biological aging: we did not find evidence of a causal impact of genetically predicted AD on biological aging (Supplementary Table S1).

**Figure 1:**
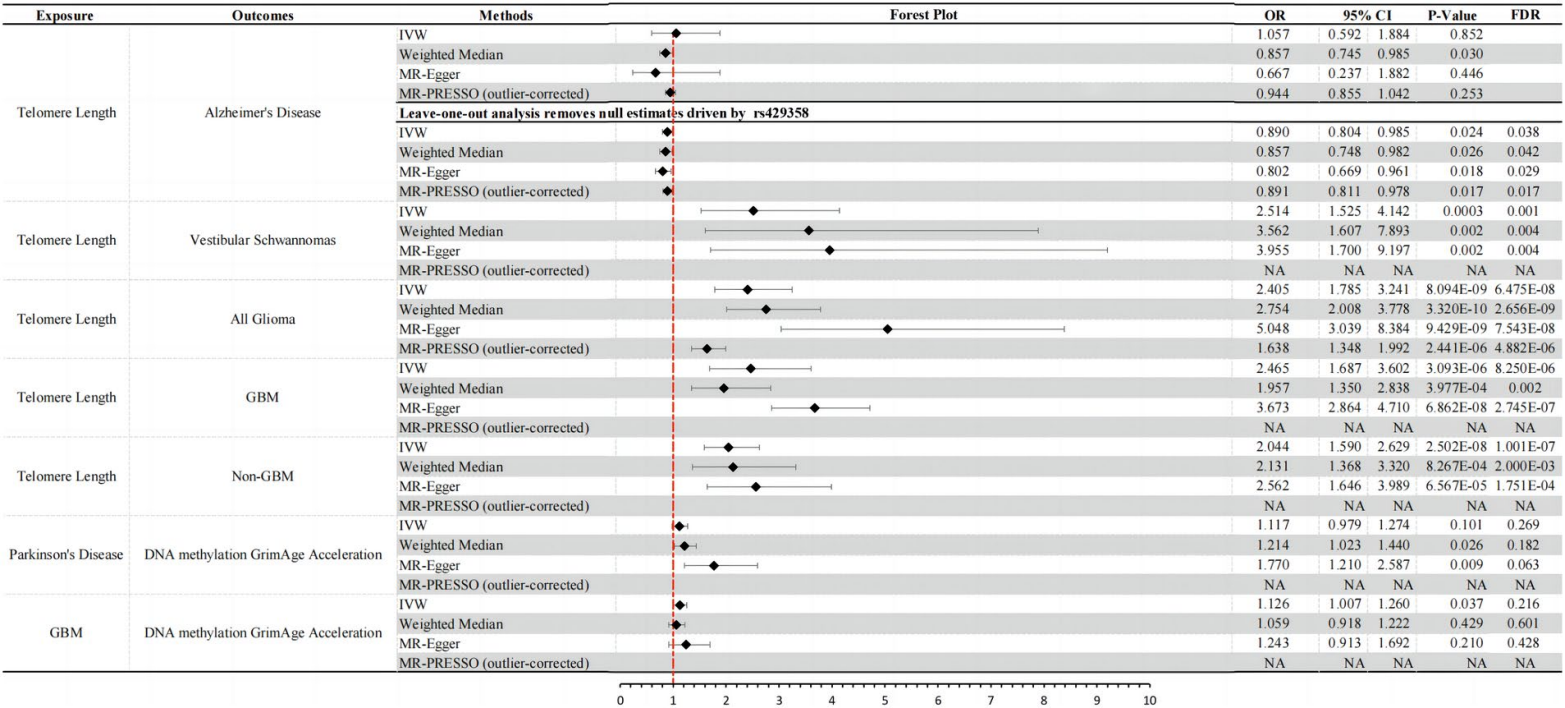
Significant results and forest plots from IVW, weighted-median, MR-Egger regression, and outlier-corrected MR-PRESSO.

Biological aging to AD: in reverse causal inference analysis, we excluded the ineffective genetic instrument rs429358 through leave-one-out analysis (Supplementary Figure S1A). Consistently across three MR analysis methods, genetically predicted longer TL was associated with a decreased risk of AD [IVW: OR = 0.890, 95% CI = 0.804 ~ 0.985, *p*-value (corrected) = 0.038; weighted median: OR = 0.857, 95% CI = 0.748 ~ 0.982, *p*-value (corrected) = 0.042; MR-Egger: OR = 0.802, 95% CI = 0.669 ~ 0.961, *p*-value (corrected) = 0.029] (Figure 1). Furthermore, we conducted tests for pleiotropy and MR-PRESSO analysis, which indicated that this result was not influenced by horizontal pleiotropy (Supplementary Tables S1, S2). Although heterogeneity tests showed some degree of heterogeneity in the results (heterogeneity test: *p* < 0.05), it did not affect our causal inference regarding the relationship between the two (Supplementary Table S2). Additionally, our study did not find that other biological aging proxy indicators had an impact on the risk of AD (Supplementary Table S1).

#### Parkinson’s disease

PD to biological aging: although MR analysis using the IVW method did not provide definitive evidence that an increased genetic risk for PD leads to epigenetic aging acceleration (DNA methylation GrimAge acceleration) (IVW: OR = 1.117, 95% CI = 0.979 ~ 1.274, *p* = 0.101), the other two analysis methods both indicated a causal relationship between them. All three methods consistently showed a direction of causality suggesting an increased risk (OR > 1) (weighted median: OR = 1.214, 95% CI = 1.023 ~ 1.440, *p* = 0.026; MR-Egger: OR = 1.770, 95% CI = 1.210 ~ 2.587, *p* = 0.009) (Figure 1). Importantly, MR-PRESSO analysis results indicated that this association was not influenced by horizontal pleiotropy (MR-PRESSO test: *p* > 0.05; Supplementary Table S1), and there was no apparent heterogeneity or confounding effects (heterogeneity test: *p* > 0.05; Supplementary Table S2). However, after further adjustment using Benjamini–Hochberg false discovery rate (FDR), we found that the corrected *p*-values were all >0.05 (Supplementary Table S1). In summary, our results suggest a suggestive causal relationship between PD and DNA methylation GrimAge acceleration. An increase in PD risk may promote DNA methylation acceleration. However, we did not find evidence that an increased risk for PD has a significant impact on other biological aging proxy indicators.

Biological aging to PD: in reverse MR analysis, we did not find that genetically predicted biological aging proxy indicators significantly affect the risk of PD (Supplementary Table S1).

#### Amyotrophic lateral sclerosis

There is no evidence of a causal relationship between ALS and biological aging in the current results of this study (Supplementary Table S1).

### Benign neurological tumor

#### Vestibular schwannomas

Vestibular schwannomas to biological aging: we did not find that genetically predicted risk of vestibular schwannomas significantly affect biological aging (Supplementary Table S1).

Biological aging to vestibular schwannomas: by employing three different MR analysis methods, including IVW, Weighted Median, and MR-Egger, we consistently observed a significant positive association between genetically predicted longer TL and an increased risk of vestibular schwannoma [IVW: OR = 2.514, 95% CI = 1.525 ~ 4.412, *p*-value (corrected) = 0.001; weighted median: OR = 3.562, 95% CI = 1.607 ~ 7.893, *p*-value (corrected) = 0.004; MR-Egger: OR = 3.955, 95% CI = 1.700 ~ 9.197, *p*-value (corrected) = 0.004] (Figure 1). It’s noteworthy that our results were further validated through MR-PRESSO and heterogeneity tests, demonstrating that this causal relationship is not influenced by horizontal pleiotropy (*P* > 0.05; Supplementary Tables S1, S2) and is not disrupted by heterogeneity confounding factors (heterogeneity test: *p* > 0.05; Supplementary Table S2). Furthermore, our study did not find any significant causal relationships between other genetically predicted biological aging proxy indicators and the risk of vestibular schwannoma (Supplementary Table S1).

#### Meningioma

In the current findings of this study, there is no evidence to suggest causal relationship between meningioma and biological aging (Supplementary Table S1).

### Malignant neurological tumor

#### All glioma

All glioma to biological aging: we did not find that genetically predicted risk of glioma significantly affects biological aging (Supplementary Table S1).

Biological aging to all glioma: consistent with previous research findings (Saunders et al., 2022), we observed a positive association between genetically predicted longer TL and the risk of glioma. We employed various causal inference methods to validate this association, including IVW, Weighted Median, and MR-Egger analysis. The results of these analyses all indicate a significant causal relationship between TL and glioma risk [IVW: OR = 2.405, 95% CI = 1.785 ~ 3.241, *p*-value (corrected) = 6.475E-08; weighted median: OR = 3.562, 95% CI = 1.607 ~ 7.893, *p*-value (corrected) = 2.656E-09; MR-Egger: OR = 3.955, 95% CI = 1.700 ~ 9.197, *p*-value (corrected) = 7.543E-08] (Figure 1). It is worth noting that we conducted tests for horizontal pleiotropy and heterogeneity, which revealed some degree of influence on this causal inference due to pleiotropy (pleiotropy test: *p* = 0.001) and heterogeneity (heterogeneity test: *p* < 0.001; Supplementary Table S2). However, after correction using the MR-PRESSO Outlier Corrected method, the impact of horizontal pleiotropy was eliminated, and the results still demonstrated a significant causal relationship between TL and glioma risk [MR-PRESSO (outlier-corrected): OR = 1.638, 95% CI = 1.348 ~ 1.992, *p*-value (corrected) = 4.882E-06] (Figure 1). Other biological aging proxy indicators with All Glioma yielded negative results (Supplementary Table S1).

#### GBM

GBM to biological aging: we found that there is a suggestive casual relationship between increased risk of GBM and epigenetic age acceleration (DNA methylation GrimAge acceleration) (IVW: OR = 1.126, 95% CI = 1.007 ~ 1.260, *p*-value = 0.037, *p*-value (corrected) = 0.216). The other two MR analysis methods yielded consistent causal effect directions with IVW (Figure 1), and they were not affected by horizontal pleiotropy (pleiotropy test: *p* > 0.05) and heterogeneity (heterogeneity test: *p* > 0.05; Supplementary Table S2).

Biological aging to GBM: similar to the results observed in All Glioma, we also found a significant causal relationship between genetically predicted TL and the risk of GBM (glioblastoma) [IVW: OR = 2.465, 95% CI = 1.687 ~ 3.602, *p*-value (corrected) = 8.250E-06; weighted median: OR = 1.957, 95% CI = 1.350 ~ 2.838, *p*-value (corrected) = 0.002; MR-Egger: OR = 3.673, 95% CI = 2.864 ~ 4.710, *p*-value (corrected) = 2.745E-07] (Figure 1). Similarly, we conducted tests for horizontal pleiotropy and heterogeneity, which indicated some heterogeneity interference with the current causal inference (heterogeneity test: *p* < 0.05), but no influence from horizontal pleiotropy (Supplementary Table S2). MR-PRESSO results also confirmed the absence of horizontal pleiotropy impact (Figure 1). Apart from these two findings above, bidirectional MR analysis results of other biological aging proxy indicators with GBM were negative (Supplementary Table S1).

#### Non-GBM

Non-GBM to biological aging: we did not find that genetically predicted risk of Non-GBM significantly affects biological aging (Supplementary Table S1).

Biological aging to non-GBM: longer telomeres also increase the risk of Non-GBM (non-Glioblastoma) [IVW: OR = 2.044, 95% CI = 1.590 ~ 2.629, *p*-value (corrected) = 1.001E-07; Weighted Median: OR = 2.131, 95% CI = 1.368 ~ 3.320, *p*-value (corrected) = 2.000E-03; MR-Egger: OR = 2.562, 95% CI = 1.646 ~ 3.989, *p*-value (corrected) = 1.751E-04] (Figure 1). It is worth noting that we conducted tests for horizontal pleiotropy and heterogeneity, and the results indicated that this causal relationship inference was influenced by heterogeneity (heterogeneity test: *p* < 0.001) but not affected by horizontal pleiotropy (Supplementary Table S2). Bidirectional MR analysis results of other biological aging proxy indicators with Non-GBM were negative (Supplementary Table S1).

## Discussion

In this bidirectional MR study examining the association between biological aging and neurodegenerative diseases and neurological tumors, we found that TL influences the risk of AD, Vestibular Schwannoma, All Glioma, GBM, and Non-GBM. Notably, telomere shortening, typically considered a hallmark of biological aging, was only found to increase the risk of AD while reducing the risk of the latter four conditions. It is worth mentioning that we observed there is a suggestive causal relationship between some diseases (PD and GBM) and DNA methylation GrimAge acceleration, suggesting that these two diseases might, to some extent, accelerate biological aging. Ultimately, for the two key characteristics of biological aging, namely frailty index and facial aging, we did not find any evidence of a positive or negative causal relationship with the neurodegenerative diseases and neurological tumors considered in this study.

Our MR estimates regarding the causal inference between TL and the risk of AD align with the findings of Blanca et al.’s MR study (Rodríguez-Fernández et al., 2022b). Utilizing a larger sample size from GWAS studies for MR analysis, we corroborated that shorter telomeres are associated with an increased risk of AD, further underscoring the significance of TL in AD pathology. Surprisingly, aside from TL, other physiological aging proxy measures, including frailty index, facial aging, and DNA methylation GrimAge acceleration, did not exhibit causal associations with the risk of AD. This outcome prompts significant discussions and reflections. Firstly, it is essential to recognize that different physiological aging proxy measures may reflect aging processes at various biological levels. TL is commonly regarded as a cellular-level marker of aging, and its shortening may be linked to biological processes such as decreased cellular function, increased inflammation, and apoptosis, which might play crucial roles in the pathogenesis of AD (Rodríguez-Fernández et al., 2022a). Conversely, phenotypic measures like the frailty index and facial aging are more likely to reflect the overall decline in physical health and function, influenced by multiple factors, including lifestyle, nutrition, environment, and genetics (Atkins et al., 2021). Thus, while these indicators play crucial roles in the overall manifestation of aging, their direct causal relationship with AD might be weaker or more complex. Secondly, DNA methylation GrimAge acceleration, as an epigenetic aging clock, has been closely associated with overall mortality and age-related health conditions (Duan et al., 2022). However, its causal relationship with the risk of AD remains inconclusive. Some studies suggest there is currently no evidence of an association between epigenetic aging and dementia/mild cognitive impairment, while others provide evidence of an association, particularly concerning GrimAge acceleration (Zhou et al., 2022).

In prior research, TL has similarly been demonstrated to have no causal association with other neurodegenerative diseases (PD and ALS) (Rodríguez-Fernández et al., 2022b). Furthermore, although frailty index and facial aging are both important proxies of physiological aging, and frailty may impact the clinical presentation and progression of neurodegenerative diseases, the relationship between these factors and PD remains unclear in most current studies. Only a few studies have found that PD patients may be more prone to frailty or that frailty is associated with motor and non-motor features of PD (Belvisi et al., 2022; Borda et al., 2022). There is almost no research on the relationship between frailty index and ALS, with only a few studies focusing on the frailty status of ALS patients (Larson and Wilbur, 2020). In addition to the findings mentioned above, it is noteworthy that we have, for the first time, discovered that PD may accelerate DNA methylation GrimAge. Previous research has predominantly focused on understanding how aging impacts PD, with some studies illustrating a connection between DNAm-age acceleration and the age of PD onset (Tang et al., 2022). However, there has been limited investigation into the influence of PD on DNAm age (Salvioli et al., 2023). A case–control analysis revealed that PD patients exhibit a higher DNAm age based on different epigenetic clocks (Horvath and Ritz, 2015; Paul et al., 2021). Some of these associations are also correlated with a more rapid decline in cognitive abilities and the progression of motor symptoms in patients (Paul et al., 2021). Nevertheless, another longitudinal study of PD patients did not observe such a correlation (Tang et al., 2022). Due to the constraints of traditional observational studies and ethical considerations in clinical research, exploring the impact of neurodegenerative diseases on aging has been nearly impractical. We employed the MR method, marking the first instance, to demonstrate that PD may contribute to the acceleration of GrimAge.

In exploring the causal associations between physiological aging proxies and benign neurological tumors, we have, for the first time, employed MR analysis to reveal that genetically predicted longer TL is associated with an elevated risk of vestibular schwannoma. Furthermore, our results have been validated through sensitivity analyses, including tests for heterogeneity and horizontal pleiotropy, confirming the robustness of our findings. To the best of our knowledge, no prior studies have investigated the relationship between TL and vestibular schwannoma. However, it should be noted that the GWAS study sample size for vestibular schwannoma used in our analysis is relatively small, and we plan to validate our results in the future using larger GWAS summary statistics datasets. Additionally, we did not find any causal relationship between physiological aging and benign neurological tumors (vestibular schwannoma and meningioma).

Finally, our results are consistent with previous MR studies, confirming that genetically predicted longer TL is associated with an increased risk of glioma (All-Glioma, GBM, Non-GBM) (Saunders et al., 2022). Additionally, for the first time, we utilized MR analysis to confirm that an elevated genetic risk of GBM is associated with accelerated DNA methylation GrimAge. Although a prior study by Liao et al. demonstrated that epigenetic age is generally accelerated in glioma patients and is an important independent predictor of survival, they did not establish a causal relationship between the two (Liao et al., 2018). In our analysis, we not only used GWAS summary statistics for glioma with the largest available sample size, but we also examined the causal relationship between glioma subtypes (All-Glioma, GBM, non-GBM) and DNA methylation GrimAge acceleration separately. Ultimately, our findings clarify that GBM, the most malignant subtype of glioma, promotes epigenetic aging.

In this study, various neurodegenerative and neurological tumor diseases included are typically found to be more prevalent in the elderly population. However, whether this association is truly linked to aging remains largely unclear (Thakkar et al., 2014; Alzheimer’s Association, 2015; Mehta et al., 2018; Hou et al., 2019; Bloem et al., 2021). MR methods have a strong capacity for uncovering potential causal relationships, and in this study, we utilized the bidirectional two-sample MR approach along with a larger sample size of GWAS data to unveil causal relationships among PD, glioblastoma multiforme (GBM), epigenetic aging, and TL for the first time, laying a theoretical foundation for further research on the relationship between aging and neurodegenerative diseases and neurological tumors. However, there are some limitations in this study that should be noted, including the absence of gender or age stratification in the GWAS data and the lack of genetic data, as we were restricted to using whole-genome association data from individuals of European ancestry. In addition, the GWAS meta-analysis sample size for meningiomas and vestibular schwannomas is limited. We relaxed the genome-wide significance thresholds for both to identify a sufficient number of SNPs for causal inference (*p*-value <5 × 10^−6^). This adjustment may, to some extent, impact the inference of causal relationships. Ideally, our future objective is to expand the scope of analysis, including as many diverse populations as possible, and to further analyze using larger GWAS datasets.

## Data availability statement

The original contributions presented in the study are included in the article/Supplementary material, further inquiries can be directed to the corresponding author.

## Author contributions

ZZ: Conceptualization, Data curation, Formal analysis, Investigation, Methodology, Project administration, Software, Supervision, Validation, Visualization, Writing – original draft, Writing – review & editing. NL: Writing – review & editing, Data curation, Methodology, Validation, Investigation. XP: Data curation, Supervision, Validation, Visualization, Writing – review & editing. CZ: Investigation, Methodology, Software, Writing – review & editing. YY: Investigation, Software, Writing – review & editing. XL: Writing - review & editing, Data curation, Investigation. YS: Conceptualization, Data curation, Investigation, Project administration, Software, Supervision, Writing – original draft, Writing – review & editing.

## Footnotes

^1^https://pdgenetics.org/

^2^https://gwas.mrcieu.ac.uk/
