# Corrigendum: Assessing causal associations between neurodegenerative diseases and neurological tumors with biological aging: a bidirectional Mendelian randomization study

**DOI:** 10.3389/fnins.2024.1418325

**Published:** 2024-05-08

**Authors:** Zhiyun Zhang, Ningfang Liu, Xuyang Pan, Chuyi Zhang, Yifan Yang, Xinyun Li, Ying Shao

**Affiliations:** ^1^Department of Plastic and Reconstructive Surgery, The First Hospital of Jilin University, Changchun, China; ^2^Department of Anesthesiology, The First Affiliated Hospital of Xiamen University, School of Medicine, Xiamen University, Xiamen, China; ^3^The Second Hospital of Jilin University, Changchun, Jilin, China; ^4^Infection Department, The First Bethune Hospital of Jilin University, Changchun, China

**Keywords:** neurodegenerative diseases, neurological tumors, biological aging, bidirectional Mendelian randomization study, genome-wide association study (GWAS)

In the published article, there was an error in the Funding statement. Funding from “Jilin Province Science and Technology Department Key Research and Development Project” was missing. The correct Funding statement appears below.

The author(s) declare financial support was received for the research, authorship, and/or publication of this article. This work was supported by the Jilin Province Science and Technology Department Key Research and Development Project (grant no. 20210204154YY).

The authors apologize for this error and state that this does not change the scientific conclusions of the article in any way. The original article has been updated.

