# Supplementary material for: Assessing causal associations between neurodegenerative diseases and neurological tumors with biological aging: a bidirectional Mendelian randomization study
Source: Front Neurosci. 2023 Dec 19;17:1321246. doi: 10.3389/fnins.2023.1321246 (PMC10758410; doi:10.3389/fnins.2023.1321246)

Supplementary Material

(1) Details of each of the three two-sample MR methods that were used

In method 1, which we considered to be the main analysis (with other methods providing important sensitivity analyses), the SNP-specific Wald ratio estimates were combined using the inverse-variance weighted (IVW) approach. This method may be biased if any of the instrumental variables are invalid (i.e. if they influence the outcome by other pathways that are independent from the exposure of interest, known as horizontal pleiotropy)[1]. Thus, we also used weighted median (method 2) and MR-Egger (method 3) to combine the SNP specific estimates[2]. These methods seek to obtain an MR estimate that is robust to horizontal pleiotropy. The different assumptions of each method are described as below.

Inverse Variance Weighted (IVW) Method

The IVW method combines the SNP-specific Wald estimates (ratio of SNP on outcome to SNP on exposure) using the following formulas:

$$\hat{\beta}_{IVW} = \frac{\sum_{K=1}^{K} E_k D_k \sigma_{D_k}^{-2}}{\sum_{K=1}^{K} E_K^2 \sigma_{D_k}^{-2}} \qquad SE_{\hat{\beta}_{IVW}} = \sqrt{\frac{1}{\sum_{k=1}^{k} E_K^2 \sigma_{D_k}^{-2}}}$$

Where $\boldsymbol{E_k}$ is the mean change in exposure level per additional effect allele of SNP k and $\boldsymbol{D_k}$ is the mean change in outcomes per additional effect allele of SNP k with standard error $\boldsymbol{\sigma_{Dk}}$.

This method provides a consistent estimate of the causal effect if all genetic variants (SNPs) used as instrumental variables satisfy the instrumental variable (IV) assumptions, specifically, 1) Genetic variants are strongly associated with the exposure factor; 2) Genetic variants are independent of any potential confounding factors; 3) Genetic variants are independent of the outcome and affect the outcome solely through the exposure factor. It does not test for or take account of horizontal pleiotropy, which if present would result in violation of the exclusion restriction criteria assumption and could importantly bias the estimate of causal effect.

The IVW estimate is a statistically efficient method, but can be biased even if just one genetic variant is invalid (i.e. if just one variant has horizontal pleiotropic effects). For this reason, we used weighted median method in addition to the IVW to account for the possibility of the existence of invalid genetic IVs.

Weighted median estimator

The weighted median estimator is the median of a distribution having estimate $\boldsymbol{\beta_j}$ as its $\mathbf{P_j = 100(S_j - W_j /2)^{th}}$ percentile, where **P** is the percentile for the $\mathbf{j^{th}}$ ordered ratio estimate, $\mathbf{W_j}$ is the weight given to the $\mathbf{j^{th}}$ ordered ratio estimate, proportional to the inverse of the IV variance, and $\mathbf{S_j}$ is the sum of weights up to and including the weight of the $\mathbf{j^{th}}$ ordered ratio estimates, calculated using the following equation:

$$S_j = \sum_{K=1}^{j} W_k$$

Weights are standardized, so that the sum of the weights $\mathbf{S_j}$ is one. As with the simple median, this method assumes that no more than 50% of the genetic IVs are invalid. Additionally it assumes that no single IV contributes more than 50% of the weight. It is more statistically efficient than the simple median method.

MR-Egger regression

The MR-Egger method was developed by Bowden et al. to specifically test for horizontal pleiotropy and correct for this in MR analyses[1]. MR Egger uses a weighted linear regression of the gene–outcome coefficients $\boldsymbol{\theta_j}$ on the gene–exposure coefficients $\boldsymbol{\delta_j}$: $\boldsymbol{\theta_j = \beta_{0E} + \beta_E \times \delta_j}$, in which all the $\boldsymbol{\delta_j}$ associations are orientated to be positive, and the weights in the regression are the inverse-variances of the gene–outcome associations

References

1. Bowden J, Davey Smith G, Haycock PC, Burgess S. Consistent Estimation in Mendelian Randomization with Some Invalid Instruments Using a Weighted Median Estimator. Genet Epidemiol. 2016;40: 304–314.

2. Bowden J, Davey Smith G, Burgess S. Mendelian randomization with invalid instruments: effect estimation and bias detection through Egger regression. Int J Epidemiol. 2015;44: 512–525.

3. Varbo A, Benn M, Smith GD, Timpson NJ, Tybjaerg-Hansen A, Nordestgaard BG. Remnant cholesterol, low-density lipoprotein cholesterol, and blood pressure as mediators from obesity to ischemic heart disease. Circ Res. 2015;116: 665–673.

Supplementary Table-1 Results from IVW, Weighted-Median, MR-Egger Regression, and Outlier-Corrected MR-PRESSO

Exposure	Outcomes	Methods	Effect (OR)	95% CI		SE	P-Value	FDR
Alzheimer's Disease	Facial Aging	IVW	0.996	0.990	1.001	0.003	0.107	0.560
		Weighted Median	0.995	0.990	1.000	0.003	0.046	0.368
		MR-Egger	0.997	0.987	1.006	0.005	0.487	0.997
		MR-PRESSO (outlier-corrected)	0.997	0.992	1.000	0.002	0.134	0.410
Alzheimer's Disease	Frailty Index	IVW	0.989	0.966	1.012	0.012	0.346	0.428
		Weighted Median	1.007	0.991	1.024	0.008	0.391	0.447
		MR-Egger	1.017	0.979	1.057	0.019	0.383	0.771
		MR-PRESSO (outlier-corrected)	NA	NA	NA	NA	NA	NA
Alzheimer's Disease	Telomere Length	IVW	1.004	0.992	1.017	0.006	0.498	0.818
		Weighted Median	1.010	0.998	1.022	0.006	0.113	0.452
		MR-Egger	1.023	0.999	1.047	0.012	0.072	0.413
		MR-PRESSO (outlier-corrected)	NA	NA	NA	NA	NA	NA
Alzheimer's Disease	DNA methylation GrimAge Acceleration	IVW	0.948	0.829	1.085	0.068	0.439	0.702
		Weighted Median	0.908	0.760	1.086	0.091	0.291	0.509
		MR-Egger	0.882	0.674	1.154	0.137	0.367	0.428
		MR-PRESSO (outlier-corrected)	NA	NA	NA	NA	NA	NA
Facial Aging	Alzheimer's Disease	IVW	1.066	0.732	1.552	0.192	0.738	0.891
		Weighted Median	1.182	0.758	1.844	0.227	0.461	0.960
		MR-Egger	1.020	0.486	1.418	0.378	0.958	0.958
		MR-PRESSO (outlier-corrected)	1.018	0.731	1.382	0.169	0.917	0.917
Frailty Index	Alzheimer's Disease	IVW	0.998	0.996	1.001	0.001	0.172	0.892
		Weighted Median	0.998	0.994	1.001	0.002	0.144	0.936
		MR-Egger	0.989	0.980	0.999	0.005	0.053	0.122
		MR-PRESSO (outlier-corrected)	NA	NA	NA	NA	NA	NA
Telomere Length	Alzheimer's Disease	IVW	1.057	0.592	1.884	0.295	0.852	
		Weighted Median	0.857	0.745	0.985	0.071	0.030	
		MR-Egger	0.667	0.237	1.882	0.529	0.446	
		MR-PRESSO (outlier-corrected)	0.944	0.855	1.042	0.050	0.253	
		**Leave-one-out analysis removes null estimates driven by rs429358**						
		IVW	0.890	0.804	0.985	0.052	0.024	0.038
		Weighted Median	0.857	0.748	0.982	0.070	0.026	0.042
		MR-Egger	0.802	0.669	0.961	0.092	0.018	0.029
		MR-PRESSO (outlier-corrected)	0.891	0.811	0.978	0.048	0.017	0.017
DNA methylation GrimAge Acceleration	Alzheimer's Disease	IVW	1.033	0.981	1.088	0.026	0.221	0.842
		Weighted Median	1.003	0.944	1.066	0.031	0.917	0.968
		MR-Egger	0.966	0.866	1.078	0.056	0.565	0.805

		MR-PRESSO (outlier-corrected)	NA	NA	NA	NA	NA	NA
Parkinson's Disease	Facial Aging	IVW	1.001	0.997	1.005	0.002	0.745	0.745
		Weighted Median	1.000	0.995	1.006	0.003	0.938	0.938
		MR-Egger	1.001	0.991	1.012	0.006	0.791	0.997
		MR-PRESSO (outlier-corrected)	1.000	0.997	1.005	0.002	0.748	0.807
Parkinson's Disease	Frailty Index	IVW	0.977	0.954	1.000	0.012	0.054	0.236
		Weighted Median	0.991	0.975	1.007	0.008	0.284	0.447
		MR-Egger	0.976	0.918	1.037	0.031	0.442	0.771
		MR-PRESSO (outlier-corrected)	0.989	0.978	1.001	0.006	0.104	0.273
Parkinson's Disease	Telomere Length	IVW	1.002	0.991	1.012	0.005	0.770	0.882
		Weighted Median	0.998	0.988	1.008	0.005	0.671	0.798
		MR-Egger	0.980	0.956	1.005	0.013	0.133	0.413
		MR-PRESSO (outlier-corrected)	1.004	0.998	1.011	0.003	0.231	0.231
Parkinson's Disease	DNA methylation GrimAge Acceleration	IVW	1.117	0.979	1.274	0.067	0.101	0.269
		Weighted Median	1.214	1.023	1.440	0.087	0.026	0.182
		MR-Egger	1.770	1.210	2.587	0.194	0.009	0.063
		MR-PRESSO (outlier-corrected)	NA	NA	NA	NA	NA	NA
Facial Aging	Parkinson's Disease	IVW	0.918	0.534	1.580	0.277	0.758	0.891
		Weighted Median	1.289	0.505	3.294	0.478	0.595	0.960
		MR-Egger	1.039	0.356	3.036	0.547	0.944	0.958
		MR-PRESSO (outlier-corrected)	NA	NA	NA	NA	NA	NA
Frailty Index	Parkinson's Disease	IVW	1.009	0.595	1.711	0.269	0.974	0.994
		Weighted Median	1.041	0.482	2.250	0.393	0.919	0.936
		MR-Egger	0.090	0.009	0.897	1.174	0.061	0.122
		MR-PRESSO (outlier-corrected)	NA	NA	NA	NA	NA	NA
Telomere Length	Parkinson's Disease	IVW	1.144	0.964	1.358	0.087	0.122	0.139
		Weighted Median	0.898	0.698	1.157	0.129	0.405	0.463
		MR-Egger	0.948	0.703	1.279	0.153	0.728	0.728
		MR-PRESSO (outlier-corrected)	NA	NA	NA	NA	NA	NA
DNA methylation GrimAge Acceleration	Parkinson's Disease	IVW	0.972	0.877	1.077	0.052	0.590	0.842
		Weighted Median	0.957	0.858	1.069	0.056	0.439	0.968
		MR-Egger	0.917	0.714	1.177	0.128	0.527	0.805
		MR-PRESSO (outlier-corrected)	NA	NA	NA	NA	NA	NA
Amyotrophic Lateral Sclerosis	Facial Aging	IVW	0.986	0.943	1.031	0.023	0.542	0.745
		Weighted Median	0.977	0.931	1.025	0.025	0.341	0.598
		MR-Egger	0.956	0.827	1.105	0.074	0.571	0.997
		MR-PRESSO (outlier-corrected)	NA	NA	NA	NA	NA	NA
Amyotrophic Lateral Sclerosis	Frailty Index	IVW	0.976	0.948	1.006	0.015	0.112	0.298
		Weighted Median	0.978	0.946	1.011	0.017	0.192	0.447
		MR-Egger	0.968	0.873	1.074	0.053	0.600	0.771
		MR-PRESSO (outlier-corrected)	NA	NA	NA	NA	NA	NA

Amyotrophic Lateral Sclerosis	Telomere Length	IVW	0.987	0.968	1.006	0.010	0.183	0.732
		Weighted Median	0.993	0.973	1.014	0.010	0.530	0.798
		MR-Egger	0.962	0.922	1.005	0.022	0.155	0.413
		MR-PRESSO (outlier-corrected)	NA	NA	NA	NA	NA	NA
Amyotrophic Lateral Sclerosis	DNA methylation GrimAge Acceleration	IVW	1.110	0.792	1.556	0.172	0.545	0.718
Facial Aging	Amyotrophic Lateral Sclerosis	IVW	0.844	0.571	1.248	0.199	0.396	0.891
		Weighted Median	1.345	0.684	2.645	0.345	0.390	0.960
		MR-Egger	1.727	0.756	3.941	0.421	0.199	0.398
		MR-PRESSO (outlier-corrected)	NA	NA	NA	NA	NA	NA
Frailty Index	Amyotrophic Lateral Sclerosis	IVW	0.860	0.586	1.263	0.196	0.442	0.892
		Weighted Median	1.163	0.721	1.874	0.243	0.536	0.936
		MR-Egger	0.811	0.135	4.873	0.915	0.823	0.823
		MR-PRESSO (outlier-corrected)	NA	NA	NA	NA	NA	NA
Telomere Length	Amyotrophic Lateral Sclerosis	IVW	1.062	0.940	1.200	0.062	0.335	0.335
		Weighted Median	0.980	0.817	1.175	0.093	0.827	0.827
		MR-Egger	0.891	0.716	1.110	0.112	0.305	0.407
		MR-PRESSO (outlier-corrected)	NA	NA	NA	NA	NA	NA
DNA methylation GrimAge Acceleration	Amyotrophic Lateral Sclerosis	IVW	0.986	0.888	1.095	0.054	0.790	0.842
		Weighted Median	0.931	0.847	1.024	0.048	0.140	0.968
		MR-Egger	1.050	0.828	1.332	0.121	0.704	0.805
		MR-PRESSO (outlier-corrected)	NA	NA	NA	NA	NA	NA
Vestibular Schwannomas	Facial Aging	IVW	0.999	0.997	1.001	0.001	0.164	0.560
		Weighted Median	0.999	0.997	1.001	0.001	0.374	0.598
		MR-Egger	0.999	0.996	1.003	0.002	0.623	0.997
		MR-PRESSO (outlier-corrected)	NA	NA	NA	NA	NA	NA
Vestibular Schwannomas	Frailty Index	IVW	1.006	1.000	1.012	0.003	0.059	0.236
		Weighted Median	1.008	1.000	1.016	0.004	0.039	0.312
		MR-Egger	1.002	0.990	1.015	0.006	0.757	0.771
		MR-PRESSO (outlier-corrected)	1.004	0.999	1.008	0.002	0.109	0.273
Vestibular Schwannomas	Telomere Length	IVW	1.000	0.996	1.003	0.002	0.894	0.894
		Weighted Median	0.999	0.994	1.004	0.003	0.622	0.798
		MR-Egger	1.000	0.993	1.007	0.003	0.936	0.998
		MR-PRESSO (outlier-corrected)	NA	NA	NA	NA	NA	NA
Vestibular Schwannomas	DNA methylation GrimAge Acceleration	IVW	0.994	0.922	1.072	0.038	0.877	0.877
		Weighted Median	1.016	0.920	1.121	0.050	0.755	0.755
		MR-Egger	1.096	0.919	1.307	0.090	0.336	0.428
		MR-PRESSO (outlier-corrected)	NA	NA	NA	NA	NA	NA
Facial Aging	Vestibular Schwannomas	IVW	0.681	0.677	0.685	0.003	0.291	0.891
		Weighted Median	2.812	2.768	2.856	0.008	0.813	0.960
		MR-Egger	0.299	0.259	0.345	0.073	0.471	0.754
		MR-PRESSO (outlier-corrected)	NA	NA	NA	NA	NA	NA

Frailty Index	Vestibular Schwannomas	IVW	1.765	1.721	1.811	0.013	0.669	0.892
		Weighted Median	2.241	2.223	2.258	0.004	0.584	0.936
		MR-Egger	7.267	7.182	7.353	0.006	0.520	0.594
		MR-PRESSO (outlier-corrected)	NA	NA	NA	NA	NA	NA
Telomere Length	Vestibular Schwannomas	IVW	2.514	1.525	4.142	0.255	0.0003	0.001
		Weighted Median	3.562	1.607	7.893	0.406	0.002	0.004
		MR-Egger	3.955	1.700	9.197	0.431	0.002	0.004
		MR-PRESSO (outlier-corrected)	NA	NA	NA	NA	NA	NA
DNA methylation GrimAge Acceleration	Vestibular Schwannomas	IVW	1.034	0.745	1.435	0.167	0.842	0.842
		Weighted Median	0.986	0.671	1.451	0.197	0.945	0.968
		MR-Egger	0.813	0.394	1.679	0.370	0.606	0.805
		MR-PRESSO (outlier-corrected)	NA	NA	NA	NA	NA	NA
Meningioma	Facial Aging	IVW	1.000	0.999	1.002	0.001	0.733	0.745
		Weighted Median	1.000	0.998	1.002	0.001	0.721	0.938
		MR-Egger	1.000	0.998	1.002	0.001	0.997	0.997
		MR-PRESSO (outlier-corrected)	NA	NA	NA	NA	NA	NA
Meningioma	Frailty Index	IVW	1.002	0.996	1.007	0.003	0.563	0.563
		Weighted Median	1.000	0.994	1.006	0.003	0.879	0.879
		MR-Egger	1.002	0.992	1.012	0.005	0.662	0.771
		MR-PRESSO (outlier-corrected)	NA	NA	NA	NA	NA	NA
Meningioma	Telomere Length	IVW	0.998	0.996	1.001	0.001	0.151	0.732
		Weighted Median	0.999	0.996	1.003	0.002	0.699	0.798
		MR-Egger	0.999	0.994	1.003	0.002	0.635	0.998
		MR-PRESSO (outlier-corrected)	NA	NA	NA	NA	NA	NA
Meningioma	DNA methylation GrimAge Acceleration	IVW	0.962	0.906	1.023	0.031	0.217	0.434
		Weighted Median	0.933	0.866	1.006	0.038	0.070	0.245
		MR-Egger	0.967	0.820	1.140	0.084	0.701	0.701
		MR-PRESSO (outlier-corrected)	NA	NA	NA	NA	NA	NA
Facial Aging	Meningioma	IVW	0.681	0.663	0.699	0.014	0.780	0.891
		Weighted Median	2.812	2.698	2.930	0.021	0.625	0.960
		MR-Egger	0.299	0.278	0.322	0.037	0.747	0.958
		MR-PRESSO (outlier-corrected)	NA	NA	NA	NA	NA	NA
Frailty Index	Meningioma	IVW	1.765	1.720	1.812	0.013	0.669	0.892
		Weighted Median	2.241	2.175	2.308	0.015	0.593	0.936
		MR-Egger	3.435	3.053	3.864	0.060	0.520	0.594
		MR-PRESSO (outlier-corrected)	NA	NA	NA	NA	NA	NA
Telomere Length	Meningioma	IVW	1.905	0.952	3.812	0.354	0.069	0.092
		Weighted Median	2.098	0.681	6.470	0.574	0.197	0.263
		MR-Egger	1.490	0.456	4.866	0.604	0.510	0.583
		MR-PRESSO (outlier-corrected)	NA	NA	NA	NA	NA	NA
DNA methylation GrimAge Acceleration	Meningioma	IVW	0.943	0.568	1.567	0.259	0.822	0.842

		Weighted Median	1.060	0.609	1.844	0.283	0.837	0.968
		MR-Egger	1.156	0.334	3.997	0.633	0.830	0.830
		MR-PRESSO (outlier-corrected)	NA	NA	NA	NA	NA	NA
All Glioma	Facial Aging	IVW	0.996	0.991	1.002	0.003	0.210	0.560
		Weighted Median	0.998	0.992	1.003	0.003	0.357	0.598
		MR-Egger	0.999	0.988	1.010	0.006	0.879	0.997
		MR-PRESSO (outlier-corrected)	1.000	0.996	1.005	0.002	0.807	0.807
All Glioma	Frailty Index	IVW	1.012	0.995	1.029	0.008	0.167	0.334
		Weighted Median	1.011	0.996	1.026	0.007	0.139	0.447
		MR-Egger	1.022	0.989	1.056	0.017	0.221	0.771
		MR-PRESSO (outlier-corrected)	1.005	0.989	1.021	0.008	0.590	0.794
All Glioma	Telomere Length	IVW	0.999	0.997	1.001	0.001	0.511	0.818
		Weighted Median	1.000	0.997	1.003	0.002	0.896	0.896
		MR-Egger	1.000	0.997	1.004	0.002	0.815	0.998
		MR-PRESSO (outlier-corrected)	NA	NA	NA	NA	NA	NA
All Glioma	DNA methylation GrimAge Acceleration	IVW	1.165	0.997	1.362	0.080	0.054	0.216
		Weighted Median	1.122	0.945	1.332	0.087	0.188	0.438
		MR-Egger	1.227	0.832	1.808	0.198	0.332	0.428
		MR-PRESSO (outlier-corrected)	NA	NA	NA	NA	NA	NA
Facial Aging	All Glioma	IVW	0.994	0.472	2.095	0.380	0.988	0.988
		Weighted Median	0.905	0.341	2.401	0.498	0.840	0.960
		MR-Egger	3.320	1.969	5.597	0.267	0.037	0.272
		MR-PRESSO (outlier-corrected)	NA	NA	NA	NA	NA	NA
Frailty Index	All Glioma	IVW	1.167	0.598	2.276	0.341	0.651	0.892
		Weighted Median	1.037	0.499	2.155	0.373	0.923	0.936
		MR-Egger	0.001	3.059E-06	0.400	3.006	0.045	0.122
		MR-PRESSO (outlier-corrected)	NA	NA	NA	NA	NA	NA
Telomere Length	All Glioma	IVW	2.405	1.785	3.241	0.152	8.094E-09	6.475E-08
		Weighted Median	2.754	2.008	3.778	0.161	3.320E-10	2.656E-09
		MR-Egger	5.048	3.039	8.384	0.259	9.429E-09	7.543E-08
		MR-PRESSO (outlier-corrected)	1.638	1.348	1.992	0.100	2.441E-06	4.882E-06
DNA methylation GrimAge Acceleration	All Glioma	IVW	1.020	0.874	1.190	0.079	0.800	0.842
		Weighted Median	1.003	0.875	1.149	0.069	0.968	0.968
		MR-Egger	1.232	0.890	1.708	0.166	0.277	0.805
		MR-PRESSO (outlier-corrected)	NA	NA	NA	NA	NA	NA
GBM	Facial Aging	IVW	0.999	0.995	1.002	0.002	0.498	0.745
		Weighted Median	1.000	0.995	1.004	0.002	0.868	0.938
		MR-Egger	0.997	0.987	1.007	0.005	0.576	0.997
		MR-PRESSO (outlier-corrected)	NA	NA	NA	NA	NA	NA
GBM	Frailty Index	IVW	1.007	0.992	1.022	0.008	0.375	0.428
		Weighted Median	1.006	0.993	1.019	0.006	0.366	0.447

		MR-Egger	1.006	0.966	1.048	0.021	0.771	0.771
		MR-PRESSO (outlier-corrected)	1.002	0.989	1.014	0.006	0.794	0.794
GBM	Telomere Length	IVW	1.017	0.978	1.057	0.020	0.412	0.818
		Weighted Median	1.009	0.999	1.019	0.005	0.063	0.452
		MR-Egger	1.048	0.944	1.163	0.053	0.405	0.813
		MR-PRESSO (outlier-corrected)	1.006	0.998	1.014	0.004	0.167	0.231
GBM	DNA methylation GrimAge Acceleration	IVW	1.126	1.007	1.260	0.057	0.037	0.216
		Weighted Median	1.059	0.918	1.222	0.073	0.429	0.601
		MR-Egger	1.243	0.913	1.692	0.158	0.210	0.428
		MR-PRESSO (outlier-corrected)	NA	NA	NA	NA	NA	NA
Facial Aging	GBM	IVW	0.714	0.293	1.742	0.455	0.459	0.891
		Weighted Median	0.988	0.293	3.332	0.620	0.985	0.985
		MR-Egger	2.258	1.397	3.649	0.245	0.130	0.347
		MR-PRESSO (outlier-corrected)	0.873	0.431	1.766	0.360	0.706	0.917
Frailty Index	GBM	IVW	1.003	0.435	2.314	0.426	0.994	0.994
		Weighted Median	0.963	0.393	2.419	0.47	0.936	0.936
		MR-Egger	0.155	0.083	0.292	0.322	0.257	0.411
		MR-PRESSO (outlier-corrected)	NA	NA	NA	NA	NA	NA
Telomere Length	GBM	IVW	2.465	1.687	3.602	0.193	3.093E-06	8.250E-06
		Weighted Median	1.957	1.350	2.838	0.190	3.977E-04	0.002
		MR-Egger	3.673	2.864	4.710	0.127	6.862E-08	2.745E-07
		MR-PRESSO (outlier-corrected)	NA	NA	NA	NA	NA	NA
DNA methylation GrimAge Acceleration	GBM	IVW	1.085	0.950	1.239	0.068	0.227	0.842
		Weighted Median	1.077	0.917	1.266	0.082	0.367	0.968
		MR-Egger	1.223	0.909	1.645	0.151	0.254	0.805
		MR-PRESSO (outlier-corrected)	NA	NA	NA	NA	NA	NA
Non-GBM	Facial Aging	IVW	0.999	0.995	1.003	0.002	0.664	0.745
		Weighted Median	1.002	0.999	1.005	0.002	0.255	0.598
		MR-Egger	1.003	0.997	1.010	0.003	0.354	0.997
		MR-PRESSO (outlier-corrected)	1.002	0.999	1.004	0.001	0.205	0.410
Non-GBM	Frailty Index	IVW	1.007	0.992	1.022	0.008	0.375	0.428
		Weighted Median	1.006	0.993	1.019	0.007	0.368	0.447
		MR-Egger	1.006	0.966	1.048	0.021	0.771	0.771
		MR-PRESSO (outlier-corrected)	1.002	0.989	1.014	0.006	0.794	0.794
Non-GBM	Telomere Length	IVW	1.006	0.976	1.038	0.016	0.680	0.882
		Weighted Median	0.997	0.991	1.004	0.003	0.409	0.798
		MR-Egger	1.000	0.951	1.052	0.026	0.998	0.998
		MR-PRESSO (outlier-corrected)	1.009	0.999	1.018	0.005	0.083	0.231
Non-GBM	DNA methylation GrimAge Acceleration	IVW	1.050	0.863	1.276	0.100	0.628	0.718
		Weighted Median	1.057	0.892	1.253	0.087	0.524	0.611
		MR-Egger	2.699	1.237	5.888	0.398	0.030	0.105

		MR-PRESSO (outlier-corrected)	1.076	0.948	1.222	0.065	0.278	0.278
Facial Aging	Non-GBM	IVW	1.405	0.479	4.119	0.549	0.535	0.891
		Weighted Median	1.427	0.379	5.373	0.677	0.599	0.960
		MR-Egger	2.249	0.827	6.114	0.510	0.068	0.272
		MR-PRESSO (outlier-corrected)	0.592	0.267	1.312	0.406	0.200	0.600
Frailty Index	Non-GBM	IVW	1.457	0.632	3.358	0.426	0.377	0.892
		Weighted Median	1.213	0.429	3.429	0.530	0.716	0.936
		MR-Egger	2.964E-04	1.706E-07	0.515	3.806	0.056	0.122
		MR-PRESSO (outlier-corrected)	NA	NA	NA	NA	NA	NA
Telomere Length	Non-GBM	IVW	2.044	1.590	2.629	0.128	2.502E-08	1.001E-07
		Weighted Median	2.131	1.368	3.320	0.226	8.267E-04	2.000E-03
		MR-Egger	2.562	1.646	3.989	0.226	6.567E-05	1.751E-04
		MR-PRESSO (outlier-corrected)	NA	NA	NA	NA	NA	NA
DNA methylation GrimAge Acceleration	Non-GBM	IVW	0.969	0.770	1.220	0.117	0.788	0.842
		Weighted Median	0.977	0.821	1.163	0.089	0.794	0.968
		MR-Egger	1.176	0.689	2.009	0.273	0.584	0.805
		MR-PRESSO (outlier-corrected)	NA	NA	NA	NA	NA	NA

Supplementary Table-2 Heterogeneity Test of the IVW and MR Egger analyses and Pleiotropy Test (Egger Intercept)

Exposure	Outcome	Methods	Cochran's Q	P-value (Heterogeneity Test)	P-Value (Pleiotropy Test)
Alzheimer's Disease	Facial Aging	MR Egger	90.144	5.628E-07	0.768
		Inverse variance weighted	90.379	8.696E-07	
Alzheimer's Disease	Frailty Index	MR Egger	152.117	7.026E-17	0.080
		Inverse variance weighted	166.697	4.556E-19	
Alzheimer's Disease	Telomere Length	MR Egger	78.046	1.015E-05	0.090
		Inverse variance weighted	85.511	1.528E-06	
Alzheimer's Disease	DNA methylation GrimAge Acceleration	MR Egger	32.684	0.336	0.544
		Inverse variance weighted	33.095	0.365	
Facial Aging	Alzheimer's Disease	MR Egger	161.581	5.149E-09	0.893
		Inverse variance weighted	161.623	7.782E-09	
Frailty Index	Alzheimer's Disease	MR Egger	12.732	0.469	0.088
		Inverse variance weighted	16.130	0.306	
Telomere Length	Alzheimer's Disease	MR Egger	216.631	6.098E-08	0.732
		Inverse variance weighted	216.849	7.969E-08	
		**Leave-one-out analysis removes null estimates driven by**			**rs429358**
		MR Egger	262.414	4.596E-10	0.177
		Inverse variance weighted	265.965	2.669E-10	
DNA methylation GrimAge Acceleration	Alzheimer's Disease	MR Egger	3.588	0.610	0.233
		Inverse variance weighted	5.426	0.490	

Parkinson's Disease	Facial Aging	MR Egger	32.237	0.041	0.877
		Inverse variance weighted	32.277	0.055	
Parkinson's Disease	Frailty Index	MR Egger	107.731	5.083E-14	0.965
		Inverse variance weighted	107.741	1.203E-13	
Parkinson's Disease	Telomere Length	MR Egger	47.790	4.553E-04	0.082
		Inverse variance weighted	55.787	5.423E-05	
Parkinson's Disease	DNA methylation GrimAge Acceleration	MR Egger	15.260	0.506	0.024
		Inverse variance weighted	21.499	0.205	
Facial Aging	Parkinson's Disease	MR Egger	81.048	0.194	0.793
		Inverse variance weighted	81.127	0.216	
Frailty Index	Parkinson's Disease	MR Egger	10.405	0.661	0.054
		Inverse variance weighted	14.873	0.387	
Telomere Length	Parkinson's Disease	MR Egger	174.864	0.005	0.136
		Inverse variance weighted	177.889	0.004	
DNA methylation GrimAge Acceleration	Parkinson's Disease	MR Egger	6.575	0.254	0.632
		Inverse variance weighted	6.917	0.329	
Amyotrophic Lateral Sclerosis	Facial Aging	MR Egger	9.742	0.083	0.676
		Inverse variance weighted	10.124	0.120	
Amyotrophic Lateral Sclerosis	Frailty Index	MR Egger	0.591	0.744	0.881
		Inverse variance weighted	0.620	0.892	
Amyotrophic Lateral Sclerosis	Telomere Length	MR Egger	5.840	0.211	0.274
		Inverse variance weighted	8.185	0.146	
Amyotrophic Lateral Sclerosis	DNA methylation GrimAge Acceleration	Inverse variance weighted	0.265	0.607	NA
Facial Aging	Amyotrophic Lateral Sclerosis	MR Egger	64.594	0.628	0.058
		Inverse variance weighted	68.316	0.535	
Frailty Index	Amyotrophic Lateral Sclerosis	MR Egger	15.516	0.214	0.949
		Inverse variance weighted	15.522	0.276	
Telomere Length	Amyotrophic Lateral Sclerosis	MR Egger	170.590	0.027	0.062
		Inverse variance weighted	174.989	0.018	
DNA methylation GrimAge Acceleration	Amyotrophic Lateral Sclerosis	MR Egger	11.000	0.051	0.582
		Inverse variance weighted	11.762	0.068	
Vestibular Schwannomas	Facial Aging	MR Egger	12.040	0.798	0.816
		Inverse variance weighted	12.096	0.842	
Vestibular Schwannomas	Frailty Index	MR Egger	19.446	0.246	0.490
		Inverse variance weighted	20.052	0.272	
Vestibular Schwannomas	Telomere Length	MR Egger	17.171	0.375	0.987
		Inverse variance weighted	17.171	0.443	
Vestibular Schwannomas	DNA methylation GrimAge Acceleration	MR Egger	6.363	0.703	0.262
		Inverse variance weighted	7.792	0.649	
Facial Aging	Vestibular Schwannomas	MR Egger	53.359	0.538	0.813
		Inverse variance weighted	53.415	0.573	

Frailty Index	Vestibular Schwannomas	MR Egger	8.073	0.779	0.611
		Inverse variance weighted	8.346	0.820	
Telomere Length	Vestibular Schwannomas	MR Egger	136.108	0.060	0.195
		Inverse variance weighted	138.172	0.054	
DNA methylation GrimAge Acceleration	Vestibular Schwannomas	MR Egger	11.000	0.051	0.582
		Inverse variance weighted	11.762	0.068	
Meningioma	Facial Aging	MR Egger	9.406	0.494	0.811
		Inverse variance weighted	9.466	0.579	
Meningioma	Frailty Index	MR Egger	17.912	0.056	0.874
		Inverse variance weighted	17.959	0.083	
Meningioma	Telomere Length	MR Egger	9.385	0.496	0.692
		Inverse variance weighted	9.552	0.571	
Meningioma	DNA methylation GrimAge Acceleration	MR Egger	9.083	0.169	0.956
		Inverse variance weighted	9.088	0.246	
Facial Aging	Meningioma	MR Egger	53.359	0.538	0.813
		Inverse variance weighted	53.415	0.573	
Frailty Index	Meningioma	MR Egger	20.668	0.055	0.570
		Inverse variance weighted	21.255	0.068	
Telomere Length	Meningioma	MR Egger	126.669	0.235	0.616
		Inverse variance weighted	126.946	0.250	
DNA methylation GrimAge Acceleration	Meningioma	MR Egger	5.393	0.249	0.737
		Inverse variance weighted	5.567	0.351	
All Glioma	Facial Aging	MR Egger	38.979	2.561E-05	0.594
		Inverse variance weighted	40.163	3.355E-05	
All Glioma	Frailty Index	MR Egger	33.745	2.040E-04	0.496
		Inverse variance weighted	35.432	2.102E-04	
All Glioma	Telomere Length	MR Egger	18.523	0.357	0.473
		Inverse variance weighted	19.109	0.385	
All Glioma	DNA methylation GrimAge Acceleration	MR Egger	13.780	0.088	0.782
		Inverse variance weighted	13.921	0.125	
Facial Aging	All Glioma	MR Egger	44.059	0.775	0.454
		Inverse variance weighted	44.629	0.787	
Frailty Index	All Glioma	MR Egger	13.008	0.293	0.056
		Inverse variance weighted	19.411	0.079	
Telomere Length	All Glioma	MR Egger	324.090	6.053E-25	0.001
		Inverse variance weighted	362.006	1.620E-30	
DNA methylation GrimAge Acceleration	All Glioma	MR Egger	7.293	0.121	0.273
		Inverse variance weighted	10.235	0.069	
GBM	Facial Aging	MR Egger	11.002	0.139	0.725
		Inverse variance weighted	11.213	0.190	
GBM	Frailty Index	MR Egger	29.115	3.027E-04	0.979

		Inverse variance weighted	29.118	0.001	
GBM	Telomere Length	MR Egger	515.689	3.024E-106	0.553
		Inverse variance weighted	540.451	1.241E-110	
GBM	DNA methylation GrimAge Acceleration	MR Egger	8.095	0.324	0.523
		Inverse variance weighted	8.619	0.375	
Facial Aging	GBM	MR Egger	69.162	0.080	0.058
		Inverse variance weighted	73.960	0.045	
Frailty Index	GBM	MR Egger	17.909	0.084	0.255
		Inverse variance weighted	20.257	0.062	
Telomere Length	GBM	MR Egger	134.752	0.014	0.075
		Inverse variance weighted	139.058	0.009	
DNA methylation GrimAge Acceleration	GBM	MR Egger	3.710	0.447	0.426
		Inverse variance weighted	4.493	0.481	
Non-GBM	Facial Aging	MR Egger	29.115	0.000	0.979
		Inverse variance weighted	29.118	0.001	
Non-GBM	Frailty Index	MR Egger	37.549	3.396E-04	0.205
		Inverse variance weighted	44.972	4.122E-05	
Non-GBM	Telomere Length	MR Egger	579.146	2.132E-115	0.753
		Inverse variance weighted	583.753	1.522E-115	
Non-GBM	DNA methylation GrimAge Acceleration	MR Egger	20.191	0.043	0.034
		Inverse variance weighted	31.011	0.002	
Facial Aging	Non-GBM	MR Egger	88.211	0.002	0.086
		Inverse variance weighted	93.218	0.001	
Frailty Index	Non-GBM	MR Egger	12.057	0.359	0.064
		Inverse variance weighted	17.575	0.129	
Telomere Length	Non-GBM	MR Egger	138.935	0.007	0.228
		Inverse variance weighted	140.959	0.006	
DNA methylation GrimAge Acceleration	Non-GBM	MR Egger	11.347	0.023	0.471
		Inverse variance weighted	13.139	0.022	

Supplementary Table-3 Characteristics of the single nucleotide polymorphisms (SNP) used as instrumental variables for Alzheimer's Disease

SNP	Effect Allele	Other Allele	Effect (BETA)	EAF	MAF	Standard Error	P-Value
rs10437655	A	G	0.0634589	0.387521	0.387521	0.0097291	6.91E-11
rs10792832	G	A	0.103282	0.629462	0.370538	0.00979201	5.21E-26
rs10933431	C	G	0.0770169	0.781729	0.218271	0.0120054	1.41E-10
rs111243475	T	C	0.19979	0.031773	0.031773	0.0283736	1.90E-12
rs11218343	C	T	-0.186386	0.037005	0.037005	0.0247939	5.59E-14
rs114812713	C	G	0.208485	0.026623	0.026623	0.0292669	1.05E-12
rs1171814	T	G	-0.0523917	0.480668	0.480668	0.00952613	3.80E-08
rs12151021	G	A	-0.0772974	0.674169	0.325831	0.0105546	2.41E-13
rs12590654	A	G	-0.0762777	0.339674	0.339674	0.0101981	7.45E-14
rs12703526	T	G	0.0654321	0.51609	0.48391	0.00960547	9.63E-12
rs1385742	T	A	-0.0690235	0.646725	0.353275	0.010164	1.11E-11
rs141622900	A	G	-0.437274	0.052416	0.052416	0.0235666	7.45E-77
rs157580	A	G	0.352722	0.605067	0.394933	0.00991069	1.00E-200
rs17125924	G	A	0.102405	0.092233	0.092233	0.0163414	3.69E-10
rs1859788	G	A	0.0896617	0.679387	0.320613	0.0103045	3.28E-18
rs1878036	G	T	0.0701099	0.793475	0.206525	0.0117898	2.74E-09
rs268134	G	A	-0.0617528	0.749812	0.250188	0.0109167	1.54E-08
rs2830489	T	C	-0.0589648	0.274903	0.274903	0.0106508	3.09E-08
rs28615360	A	G	0.576298	0.014744	0.014744	0.042979	5.37E-41
rs2884738	A	C	-0.0620662	0.281382	0.281382	0.0105808	4.47E-09
rs2927437	G	A	-0.212244	0.807332	0.192668	0.0120396	1.48E-69
rs36096565	G	A	-0.103604	0.210869	0.210869	0.0131209	2.88E-15
rs4311	C	T	0.0542811	0.529632	0.470368	0.00952698	1.21E-08
rs4351014	C	T	-0.0706868	0.735069	0.264931	0.0106002	2.59E-11
rs442495	C	T	-0.0675868	0.319058	0.319058	0.0101428	2.67E-11
rs4575098	A	G	0.0609053	0.23499	0.23499	0.0111182	4.30E-08
rs6014724	G	A	-0.109364	0.087805	0.087805	0.016939	1.07E-10
rs61182333	C	T	-0.0849788	0.875068	0.124932	0.0140204	1.35E-09
rs6733839	T	C	0.155615	0.391492	0.391492	0.00999227	1.10E-54
rs679515	C	T	-0.122753	0.822818	0.177182	0.0122652	1.40E-23
rs72924626	C	T	-0.0892049	0.371215	0.371215	0.00980658	9.33E-20
rs73223431	T	C	0.0790484	0.366564	0.366564	0.00995628	2.03E-15
rs78789557	G	C	-0.0722935	0.197469	0.197469	0.0119888	1.64E-09
rs7920721	G	A	0.0668997	0.380517	0.380517	0.00984571	1.08E-11
rs867230	A	C	0.105124	0.586804	0.413196	0.0100016	7.71E-26

The variants together explain 2.4% of the variation in Alzheimer's Disease (R^2 = 0.024).

F-statistics = 324.228

EAF: Effect Allele Frequency

Supplementary Table-4 Characteristics of the single nucleotide polymorphisms (SNP) used as instrumental variables for Parkinson's Disease

SNP	Effect Allele	Other Allele	Effect (BETA)	EAF	MAF	Standard Error	P-Value
rs10451230	T	A	-0.096	0.565	0.435	0.0175	4.42E-08
rs10513789	G	T	-0.1596	0.1826	0.1826	0.0219	3.18E-13
rs10847864	T	G	0.1274	0.3625	0.3625	0.0179	9.81E-13
rs12934900	T	A	0.1215	0.6571	0.3429	0.0184	4.33E-11
rs144814361	T	C	0.4411	0.0174	0.0174	0.068	9.07E-11
rs329647	C	G	-0.1133	0.6662	0.3338	0.0178	1.94E-10
rs34311866	C	T	0.2272	0.1958	0.1958	0.0231	7.97E-23
rs35265698	G	C	-0.2	0.1547	0.1547	0.0303	3.93E-11
rs356203	T	C	-0.2398	0.6169	0.3831	0.0178	3.01E-41
rs35749011	A	G	0.7508	0.0191	0.0191	0.0659	5.02E-30
rs4488803	A	G	-0.1136	0.3746	0.3746	0.0199	1.08E-08
rs4588066	A	G	0.1046	0.326	0.326	0.0178	4.45E-09
rs4613239	G	C	0.1784	0.1326	0.1326	0.0248	6.21E-13
rs4698412	A	G	0.1258	0.553	0.447	0.0168	7.05E-14
rs4774417	A	G	0.1052	0.7397	0.2603	0.0192	4.63E-08
rs58879558	C	T	-0.2383	0.2229	0.2229	0.025	1.36E-21
rs620490	G	T	-0.1174	0.2762	0.2762	0.019	6.46E-10
rs6741007	G	T	-0.1233	0.4507	0.4507	0.0175	2.09E-12
rs75505347	T	C	0.3917	0.0195	0.0195	0.0674	6.12E-09
rs75646569	G	T	0.1916	0.1117	0.1117	0.0266	5.62E-13
rs7695720	C	A	-0.1255	0.2091	0.2091	0.0208	1.53E-09
rs823106	C	G	-0.1492	0.8488	0.1512	0.0239	4.10E-10
rs858295	G	A	-0.1039	0.3947	0.3947	0.0176	3.83E-09

The variants together explain 3.1% of the variation in Parkinson's Disease ($R^2 = 0.031$).
F-statistics = 671.591
EAF: Effect Allele Frequency

Supplementary Table-5 Characteristics of the single nucleotide polymorphisms (SNP) used as instrumental variables for Amyotrophic Lateral Sclerosis

SNP	Effect Allele	Other Allele	Effect (BETA)	EAF	MAF	Standard Error	P-Value
rs10139154	T	C	0.0200826	0.320604	0.320604	0.00368288	4.95E-08
rs12608932	C	A	0.0227481	0.352952	0.352952	0.00360193	2.69E-10
rs35714695	A	G	-0.029513	0.176036	0.176036	0.00455194	8.96E-11
rs3849943	T	C	-0.0405097	0.752045	0.247955	0.00396593	1.71E-24
rs616147	G	A	-0.0216478	0.714326	0.285674	0.00381824	1.43E-08
rs75087725	A	C	0.0887445	0.015528	0.015528	0.0149099	2.65E-09

rs7813314	C	G	-0.0320673	0.09878	0.09878	0.0057949	3.14E-08

The variants together explain 0.2% of the variation in Amyotrophic Lateral Sclerosis ($R^2 = 0.002$).

F-statistics = 36.052

EAF: Effect Allele Frequency

Supplementary Table-6 Characteristics of the single nucleotide polymorphisms (SNP) used as instrumental variables for Vestibular Schwannomas

SNP	Effect Allele	Other Allele	Effect (BETA)	EAF	MAF	Standard Error	P-Value
rs11238349	A	G	0.334968	0.0629893	0.0629893	0.0629893	1.05E-07
rs114116956	A	G	1.20223	0.258095	0.258095	0.258095	3.19E-06
rs115403498	C	G	1.22349	0.263281	0.263281	0.263281	3.37E-06
rs118082474	C	T	1.32848	0.279617	0.279617	0.279617	2.02E-06
rs12886404	A	G	0.427068	0.089015	0.089015	0.0899015	2.03E-06
rs137872210	A	G	0.973111	0.205601	0.205601	0.205601	2.21E-06
rs138641986	T	C	1.23291	0.26176	0.26176	0.26176	2.48E-06
rs139839818	C	A	0.852859	0.174374	0.174374	0.174374	1.00E-06
rs143566853	A	G	0.963142	0.202859	0.202859	0.202859	2.06E-06
rs147936255	T	C	0.554865	0.120474	0.120474	0.120474	4.11E-06
rs1556516	C	G	-0.409844	0.0554571	0.0554571	0.0554571	1.47E-13
rs2078795	C	T	0.991882	0.213048	0.213048	0.213048	3.23E-06
rs311398	T	C	-0.268638	0.0569268	0.0569268	0.0569268	2.37E-06
rs34779176	T	C	0.735265	0.151803	0.151803	0.151803	1.28E-06
rs4934068	T	C	-1.2582	0.274948	0.274948	0.274948	4.74E-06
rs59356242	A	C	0.912155	0.192582	0.192582	0.192582	2.17E-06
rs6683123	G	T	-0.278744	0.0585131	0.0585131	0.0585131	1.90E-06
rs7087412	T	C	0.483049	0.103297	0.103297	0.103297	2.92E-06
rs73910511	G	A	1.19799	0.221488	0.221488	0.221488	6.34E-08
rs79873818	C	G	0.757262	0.155861	0.155861	0.155861	1.18E-06
rs9599965	C	T	-0.322746	0.0670357	0.0670357	0.0670357	1.48E-06

The variants together explain 22.8% of the variation in Vestibular Schwannomas ($R^2 = 0.228$)

F-statistics = 89.853

EAF: Effect Allele Frequency

Supplementary Table-7 Characteristics of the single nucleotide polymorphisms (SNP) used as instrumental variables for Meningioma

SNP	Effect Allele	Other Allele	Effect (BETA)	EAF	MAF	Standard Error	P-Value
rs10833988	A	G	0.415964	0.296141	0.296141	0.0896314	3.47E-06
rs146864255	T	C	2.67588	0.0076243	0.00762431	0.581002	4.11E-06
rs17033809	A	C	0.534657	0.156613	0.156613	0.114792	3.20E-06
rs192521781	T	C	2.60899	0.0085917	0.00859174	0.544207	1.63E-06
rs2050271	A	T	1.67141	0.0173855	0.0173855	0.359636	3.36E-06
rs289744	G	T	0.420115	0.299177	0.299177	0.0907327	3.65E-06
rs56895157	C	T	1.13682	0.0335349	0.0335349	0.248273	4.67E-06
rs73944983	A	G	1.60717	0.0184158	0.0184158	0.347488	3.74E-06
rs78907823	G	A	0.746534	0.0854608	0.0854608	0.153739	1.20E-06
rs79682198	A	G	2.0352	0.0158388	0.0158388	0.390601	1.88E-07
rs9365501	G	T	-0.440217	0.285697	0.285697	0.0915552	1.52E-06
rs9961255	C	T	0.423611	0.315848	0.315848	0.0894279	2.17E-06

The variants together explain 1.0% of the variation in Meningioma ($R^2 = 0.010$)

F-statistics = 391.689

EAF: Effect Allele Frequency

Supplementary Table-8 Characteristics of the single nucleotide polymorphisms (SNP) used as instrumental variables for All Glioma

SNP	Effect Allele	Other Allele	Effect (BETA)	EAF	MAF	Standard Error	P-Value
rs10069690	T	C	0.370794	0.7237	0.2763	0.0215479	2.32E-66
rs12752552	C	T	-0.163018	0.1302	0.1302	0.0275626	3.33E-09
rs12803321	C	G	-0.158054	0.3569	0.3569	0.019711	1.07E-15
rs2157719	T	C	-0.258795	0.5765	0.4235	0.0180817	1.83E-46
rs2297440	C	T	0.305702	0.7962	0.2038	0.0224229	2.53E-42
rs35850753	T	C	0.725825	0.0249	0.0249	0.0575216	1.67E-36
rs3751667	T	C	0.131263	0.2078	0.2078	0.0214464	9.33E-10
rs4608623	T	G	-0.104404	0.4463	0.4463	0.0183408	1.25E-08
rs4951389	T	G	0.109915	0.3181	0.3181	0.0196466	2.21E-08
rs55705857	G	A	0.685745	0.9433	0.0567	0.0365759	1.99E-78
rs75061358	G	T	0.351788	0.0994	0.0994	0.0326764	4.99E-27
rs759169	C	T	-0.188506	0.8608	0.1392	0.0246297	1.95E-14
rs8051902	T	C	0.135548	0.2903	0.2903	0.0202689	2.27E-11

The variants together explain 3.7% of the variation in All Glioma ($R^2 = 0.037$).

F-statistics = 87.215

EAF: Effect Allele Frequency

Supplementary Table-9 Characteristics of the single nucleotide polymorphisms (SNP) used as instrumental variables for GBM

SNP	Effect Allele	Other Allele	Effect (BETA)	EAF	MAF	Standard Error	P-Value
rs11143912	A	C	0.217465	0.0875	0.0875	0.0388249	2.13E-08
rs11233250	T	C	-0.2272	0.8678	0.1322	0.0357478	2.08E-10
rs12752552	C	T	-0.210951	0.1302	0.1302	0.0338086	4.39E-10
rs2235573	A	G	-0.147889	0.493	0.493	0.0222789	3.18E-11
rs2297440	C	T	0.397572	0.7962	0.2038	0.0274672	1.76E-47
rs2562152	T	A	0.186651	0.1501	0.1501	0.034225	4.94E-08
rs35850753	T	C	0.778679	0.0249	0.0249	0.0728291	1.11E-26
rs4389139	A	T	0.173029	0.7097	0.2903	0.024932	3.92E-12
rs634537	G	T	0.320183	0.5895	0.4105	0.0225307	7.83E-46
rs75061358	G	T	0.480633	0.0994	0.0994	0.0402169	6.41E-33
rs759169	C	T	-0.226579	0.8608	0.1392	0.0301352	5.53E-14

The variants together explain 7.3% of the variation in GBM ($R^2 = 0.073$).

F-statistics = 161.47

EAF: Effect Allele Frequency

Supplementary Table-10 Characteristics of the single nucleotide polymorphisms (SNP) used as instrumental variables for Non-GBM

SNP	Effect Allele	Other Allele	Effect (BETA)	EAF	MAF	Standard Error	P-Value
rs10069690	T	C	0.248487	0.7237	0.2763	0.0288265	6.69E-18
rs10131032	A	G	-0.287266	0.0845	0.0845	0.0431851	2.89E-11
rs11599775	A	G	-0.149069	0.3797	0.3797	0.0250404	2.63E-09
rs11706832	C	A	0.136034	0.5437	0.4563	0.023686	9.29E-09
rs12076373	C	G	-0.211379	0.1630	0.163	0.0335615	3.01E-10
rs1275600	A	T	-0.150991	0.4046	0.4046	0.0248445	1.22E-09
rs12803321	C	G	-0.352934	0.3569	0.3569	0.0254908	1.35E-43
rs1556515	T	C	-0.201373	0.5765	0.4235	0.0238997	3.58E-17
rs2297440	C	T	0.185712	0.7962	0.2038	0.0290183	1.56E-10
rs35850753	T	C	0.78837	0.0249	0.0249	0.0793936	3.09E-23
rs3751667	T	C	0.16857	0.2078	0.2078	0.028249	2.41E-09
rs4252707	A	G	0.17569	0.2197	0.2197	0.0296846	3.25E-09
rs500629	A	C	-0.149232	0.3250	0.325	0.0256333	5.82E-09
rs55705857	G	A	1.23195	0.9433	0.0567	0.0472602	8.55E-150
rs7107785	C	T	-0.152201	0.5209	0.4791	0.0237729	1.53E-10
rs75061358	G	T	0.24542	0.0994	0.0994	0.0444095	3.27E-08
rs7572263	G	A	-0.175542	0.7565	0.2435	0.0284548	6.87E-10
rs77387260	T	C	0.306241	0.9135	0.0865	0.0408115	6.20E-14

The variants together explain 9.9% of the variation in Non-GBM (R^2 = 0.099).
F-statistics = 131.835
EAF: Effect Allele Frequency

Supplementary Table-11 Characteristics of the single nucleotide polymorphisms (SNP) used as instrumental variables for Facial Aging

SNP	Effect Allele	Other Allele	Effect (BETA)	EAF	MAF	Standard Error	P-Value
rs10011319	C	G	0.00715899	0.390695	0.390695	0.00108577	4.30E-11
rs10200279	C	T	0.00701337	0.720094	0.279906	0.00118788	3.50E-09
rs1022034	G	T	0.00860179	0.796214	0.203786	0.00131711	6.50E-11
rs10434895	T	A	-0.00986258	0.440705	0.440705	0.00106715	2.40E-20
rs10838726	G	C	-0.00987549	0.301514	0.301514	0.00115407	1.20E-17
rs10956486	C	T	-0.0179121	0.319653	0.319653	0.0011366	5.90E-56
rs11042557	C	T	0.00792521	0.390203	0.390203	0.00109016	3.60E-13
rs11242899	A	G	-0.00699108	0.267081	0.267081	0.00120186	6.00E-09
rs112537099	G	C	0.00660902	0.460039	0.460039	0.00106333	5.10E-10
rs112881196	G	C	-0.0152057	0.038788	0.038788	0.00276672	3.90E-08
rs11571404	T	C	0.00727558	0.202683	0.202683	0.00132266	3.80E-08
rs116254882	T	G	0.0231913	0.042905	0.042905	0.00261208	6.80E-19
rs11657730	T	C	0.00680668	0.36277	0.36277	0.00111058	8.80E-10
rs11684254	G	C	0.00807275	0.342192	0.342192	0.00111652	4.80E-13
rs121908120	A	T	0.0219628	0.026858	0.026858	0.00331665	3.50E-11
rs12203592	T	C	0.0487237	0.21789	0.21789	0.00126573	1.00E-200
rs12257409	T	C	0.00752765	0.347984	0.347984	0.00111443	1.40E-11
rs12350739	A	G	0.0126128	0.607244	0.392756	0.00109152	6.90E-31
rs12441130	C	T	-0.00948348	0.484986	0.484986	0.00105998	3.70E-19
rs12664003	T	C	0.00631632	0.484143	0.484143	0.00106036	2.60E-09
rs12878653	T	G	0.00900598	0.712081	0.287919	0.00117335	1.60E-14
rs12882664	T	C	-0.00687523	0.524005	0.475995	0.00106493	1.10E-10
rs13061457	C	T	0.0061388	0.39027	0.39027	0.00108798	1.70E-08
rs13096474	C	T	0.00718131	0.740045	0.259955	0.00120922	2.90E-09
rs13107325	T	C	-0.014413	0.075466	0.075466	0.00200788	7.10E-13
rs1313996	A	T	-0.00730611	0.636397	0.363603	0.00109808	2.90E-11
rs13287803	G	C	-0.00739649	0.229071	0.229071	0.00126937	5.60E-09
rs138880	C	A	0.00965914	0.19651	0.19651	0.0013374	5.10E-13
rs139356332	C	G	-0.025688	0.023325	0.023325	0.00361903	1.30E-12
rs1438898	C	A	0.0144967	0.249269	0.249269	0.00122943	4.30E-32
rs1495741	A	G	0.00725473	0.779232	0.220768	0.00127726	1.30E-08
rs150962800	T	C	-0.0195835	0.026188	0.026188	0.00332023	3.70E-09
rs1635852	C	T	0.006262	0.506653	0.493347	0.00105905	3.40E-09
rs16886260	G	A	0.00990538	0.102143	0.102143	0.00175531	1.70E-08
rs16891982	G	C	0.024759	0.971899	0.028101	0.00314766	3.70E-15

rs17036328	C	T	0.00945614	0.120817	0.120817	0.00162264	5.60E-09
rs17220628	A	G	-0.00833888	0.159506	0.159506	0.00144964	8.80E-09
rs17265513	C	T	-0.007926	0.198303	0.198303	0.00132969	2.50E-09
rs174548	G	C	-0.00931993	0.313765	0.313765	0.00114144	3.20E-16
rs17523800	C	T	-0.00604408	0.365552	0.365552	0.00110558	4.60E-08
rs1772855	T	C	0.00675074	0.640825	0.359175	0.00110379	9.60E-10
rs17855988	C	G	-0.0119446	0.09887	0.09887	0.00180473	3.60E-11
rs1805007	T	C	0.0154556	0.101849	0.101849	0.0017448	8.10E-19
rs214084	G	A	0.00595059	0.578887	0.421113	0.00107178	2.80E-08
rs2219271	G	C	0.00586886	0.468844	0.468844	0.00106449	3.50E-08
rs232138	T	C	0.00782575	0.420204	0.420204	0.00107654	3.60E-13
rs2721929	A	G	-0.00645306	0.55822	0.44178	0.0010705	1.70E-09
rs2819861	G	A	0.0066921	0.641404	0.358596	0.00111321	1.80E-09
rs28897169	C	T	0.0104875	0.606107	0.393893	0.00108444	4.00E-22
rs2972344	G	T	-0.00706814	0.537528	0.462472	0.00107307	4.50E-11
rs34827462	G	C	0.00984458	0.153607	0.153607	0.00152425	1.10E-10
rs3745473	C	T	0.00821542	0.174651	0.174651	0.00139579	4.00E-09
rs45499591	G	C	0.0121936	0.12608	0.12608	0.00159603	2.20E-14
rs4949691	A	G	-0.00870258	0.137107	0.137107	0.00155238	2.10E-08
rs520015	G	C	0.0170965	0.487309	0.487309	0.00106109	2.10E-58
rs55811103	A	C	0.00720015	0.2068	0.2068	0.00131056	3.90E-08
rs56086233	G	T	0.00749992	0.399192	0.399192	0.00108683	5.20E-12
rs61263161	A	G	-0.0212172	0.16995	0.16995	0.00141105	4.20E-51
rs61821643	C	T	-0.0255759	0.026314	0.026314	0.00350829	3.10E-13
rs62211989	C	G	0.0138176	0.097983	0.097983	0.00183711	5.40E-14
rs62287198	A	G	-0.00632755	0.536642	0.463358	0.00106914	3.30E-09
rs6740259	G	A	0.013734	0.109055	0.109055	0.00169947	6.40E-16
rs6996198	T	C	0.00953086	0.155996	0.155996	0.001462	7.10E-11
rs7089911	C	G	0.00615094	0.438255	0.438255	0.00107686	1.10E-08
rs7306710	C	T	-0.00583764	0.520735	0.479265	0.00106813	4.60E-08
rs74562379	T	C	-0.0187385	0.039472	0.039472	0.00286778	6.40E-11
rs76032374	G	A	0.0168917	0.133042	0.133042	0.00156039	2.60E-27
rs7740614	A	G	0.00633172	0.486551	0.486551	0.00106501	2.80E-09
rs7809464	A	G	-0.00695606	0.536947	0.463053	0.00106844	7.50E-11
rs7995814	C	G	0.00653392	0.580486	0.419514	0.00107702	1.30E-09
rs8031948	T	G	0.00661004	0.334359	0.334359	0.00112487	4.20E-09
rs9298467	T	C	-0.00739113	0.811151	0.188849	0.00135352	4.70E-08
rs9328259	A	C	0.00875018	0.717738	0.282262	0.00118088	1.30E-13
rs955683	C	T	0.00634197	0.513882	0.486118	0.00107222	3.30E-09
rs9601280	C	T	-0.00759506	0.204827	0.204827	0.00132069	8.90E-09
rs9882022	G	A	0.00904128	0.526018	0.473982	0.00106196	1.70E-17
rs9896202	C	T	0.00588527	0.497663	0.497663	0.00106142	2.90E-08
rs9924898	A	G	0.00588674	0.49427	0.49427	0.00106522	3.30E-08

The variants together explain 11.6% of the variation in Facial Aging ($R^2 = 0.116$).

F-statistics = 712.826

EAF: Effect Allele Frequency

Supplementary Table-12 Characteristics of the single nucleotide polymorphisms (SNP) used as instrumental variables for Frailty Index

SNP	Effect Allele	Other Allele	Effect (BETA)	EAF	MAF	Standard Error	P-Value
rs10891490	C	T	-0.0188	0.5915	0.4085	0.0034	2.00E-08
rs12739243	C	T	-0.0242	0.2206	0.2206	0.004	1.28E-09
rs1363103	C	T	-0.0191	0.38	0.38	0.0034	2.23E-08
rs17612102	C	T	0.0187	0.5933	0.4067	0.0034	2.85E-08
rs2071207	C	T	-0.0187	0.478	0.478	0.0033	1.47E-08
rs2396766	A	G	0.0201	0.4725	0.4725	0.0033	1.22E-09
rs3959554	G	A	0.0189	0.4177	0.4177	0.0034	1.74E-08
rs4146140	T	C	-0.0198	0.3811	0.3811	0.0034	6.83E-09
rs4952693	T	C	-0.0194	0.3734	0.3734	0.0034	1.47E-08
rs56299474	A	C	0.0241	0.1733	0.1733	0.0044	3.94E-08
rs583514	C	T	0.0199	0.5111	0.4889	0.0033	1.65E-09
rs8089807	T	C	-0.0248	0.1866	0.1866	0.0043	6.50E-09
rs82334	C	A	-0.0223	0.3177	0.3177	0.0035	3.13E-10
rs9275160	A	G	0.0382	0.3397	0.3397	0.0035	7.18E-28

The variants together explain 0.3% of the variation in Frailty Index ($R^2 = 0.003$).

F-statistics = 38.103

EAF: Effect Allele Frequency

Supplementary Table-13 Characteristics of the single nucleotide polymorphisms (SNP) used as instrumental variables for Telomere Length

SNP	Effect Allele	Other Allele	Effect (BETA)	EAF	MAF	Standard Error	P-Value
rs1003322	A	A	0.0141734	0.213742	0.213742	0.00247546	1.00E-08
rs10112752	A	A	-0.0287522	0.430369	0.430369	0.00202518	9.50E-46
rs1023767	A	A	-0.0183732	0.237595	0.237595	0.00234772	5.00E-15
rs10768683	G	G	0.0469922	0.841033	0.158967	0.00277015	1.50E-64
rs10773176	G	G	-0.0172009	0.741214	0.258786	0.00228534	5.20E-14
rs10774624	A	A	0.0149944	0.53276	0.46724	0.00205494	2.90E-13
rs10805346	C	C	0.0117072	0.439339	0.439339	0.00202147	7.00E-09
rs10840270	G	G	0.014383	0.655684	0.344316	0.00212494	1.30E-11
rs10845387	A	A	-0.0141214	0.352666	0.352666	0.00209396	1.50E-11
rs10905255	T	T	-0.0182493	0.57919	0.42081	0.00203099	2.60E-19
rs11085072	T	T	-0.0131806	0.236909	0.236909	0.00236713	2.60E-08

rs11117354	C	C	0.0232506	0.696513	0.303487	0.00219601	3.40E-26
rs111527438	C	C	0.0125	0.351251	0.351251	0.00211016	3.10E-09
rs111950327	C	C	0.0238271	0.063628	0.063628	0.00409406	5.90E-09
rs11212631	C	C	-0.0193458	0.199229	0.199229	0.00256554	4.70E-14
rs112394943	C	C	-0.0198961	0.162741	0.162741	0.00281641	1.60E-12
rs113525195	A	A	-0.0124075	0.290254	0.290254	0.00224132	3.10E-08
rs11557154	T	T	-0.0343719	0.13003	0.13003	0.00298538	1.10E-30
rs11579626	C	C	0.0265113	0.084882	0.084882	0.00357752	1.30E-13
rs11584821	T	T	-0.0306517	0.176208	0.176208	0.00263623	3.00E-31
rs116863223	A	A	-0.0817874	0.011763	0.011763	0.00937157	2.60E-18
rs11699829	A	A	0.0641957	0.034146	0.034146	0.00602028	1.50E-26
rs117034449	A	A	0.0374377	0.023304	0.023304	0.00667898	2.10E-08
rs117407747	T	T	0.0450533	0.027571	0.027571	0.00611706	1.80E-13
rs117512405	A	A	-0.0790134	0.017043	0.017043	0.00824611	9.50E-22
rs117630647	A	A	0.059565	0.021346	0.021346	0.00720413	1.40E-16
rs11769630	A	A	-0.0256807	0.072227	0.072227	0.00389475	4.30E-11
rs11991877	A	A	-0.030138	0.889309	0.110691	0.00318686	3.20E-21
rs12369950	C	C	-0.0178308	0.140675	0.140675	0.00290205	8.00E-10
rs12412214	A	A	-0.0245174	0.279765	0.279765	0.00222685	3.40E-28
rs12451892	C	C	-0.0116145	0.380511	0.380511	0.00207578	2.20E-08
rs1291143	C	C	0.0493145	0.849026	0.150974	0.0027991	1.80E-69
rs12925933	C	C	-0.0146622	0.66221	0.33779	0.00213796	7.00E-12
rs12932179	G	G	-0.0136257	0.561399	0.438601	0.0020276	1.80E-11
rs13062095	C	C	0.0138552	0.327843	0.327843	0.00214113	9.70E-11
rs13230646	C	C	-0.0173277	0.248945	0.248945	0.00232377	8.90E-14
rs1332941	G	G	0.0256552	0.820466	0.179534	0.00273159	5.90E-21
rs137901416	A	A	0.04572	0.100311	0.100311	0.00332355	4.70E-43
rs139669835	T	T	-0.0612563	0.009365	0.009365	0.0105346	6.10E-09
rs139795227	C	C	0.0599379	0.014021	0.014021	0.00873247	6.70E-12
rs142426306	T	T	-0.0504903	0.039544	0.039544	0.00539933	8.70E-21
rs143190905	T	T	-0.0723995	0.080404	0.080404	0.00369421	1.60E-85
rs144204502	T	T	-0.100574	0.012562	0.012562	0.00913369	3.40E-28
rs145114957	G	G	0.0272605	0.042648	0.042648	0.00498872	4.60E-08
rs150150565	T	T	0.063762	0.021455	0.021455	0.00739877	6.80E-18
rs1611236	A	A	-0.0160135	0.32687	0.32687	0.00213359	6.10E-14
rs16978028	T	T	-0.029945	0.143727	0.143727	0.00285068	8.20E-26
rs17445108	A	A	-0.0168922	0.12695	0.12695	0.00300983	2.00E-08
rs17677991	G	G	0.0222664	0.342123	0.342123	0.00210806	4.40E-26
rs17803849	T	T	0.0273203	0.405161	0.405161	0.00203482	4.20E-41
rs182059586	C	C	-0.0571159	0.02511	0.02511	0.00680853	4.90E-17
rs185174247	A	A	0.0372806	0.05609	0.05609	0.00435145	1.10E-17
rs188918174	T	T	0.0403062	0.036106	0.036106	0.00543604	1.20E-13
rs1907702	A	A	0.0150247	0.766771	0.233229	0.00242651	5.90E-10

rs1957937	T	T	0.0209365	0.16018	0.16018	0.00273361	1.90E-14
rs1985369	G	G	-0.0311893	0.868178	0.131822	0.00300952	3.60E-25
rs201558190	C	C	-0.0181748	0.363271	0.363271	0.00217477	6.40E-17
rs2056726	A	A	-0.0228078	0.214376	0.214376	0.00243638	7.90E-21
rs2230590	C	C	-0.0158022	0.510897	0.489103	0.00200806	3.60E-15
rs2276182	G	G	0.0233529	0.403227	0.403227	0.00204247	2.80E-30
rs2282764	G	G	-0.0224234	0.142384	0.142384	0.00289392	9.30E-15
rs2293579	A	A	-0.012915	0.386274	0.386274	0.00205481	3.30E-10
rs2306646	C	C	-0.0209417	0.559475	0.440525	0.00201898	3.30E-25
rs2538745	C	C	-0.012942	0.602841	0.397159	0.002056	3.10E-10
rs2555104	C	C	-0.0139717	0.434255	0.434255	0.00203498	6.60E-12
rs2763979	T	T	-0.0277713	0.359721	0.359721	0.00208092	1.30E-40
rs28363070	A	A	0.0755557	0.013389	0.013389	0.00959987	3.50E-15
rs28502153	A	A	-0.0215916	0.377958	0.377958	0.00206208	1.20E-25
rs28577594	C	C	0.0187657	0.709829	0.290171	0.00224024	5.40E-17
rs2967355	C	C	-0.0461595	0.774276	0.225724	0.00238972	4.00E-83
rs2977608	C	C	0.0129483	0.743949	0.256051	0.00233716	3.00E-08
rs3093888	A	A	-0.028973	0.05131	0.05131	0.00452459	1.50E-10
rs34896435	G	G	0.0154567	0.469158	0.469158	0.00206109	6.40E-14
rs35446936	A	A	-0.0940025	0.243674	0.243674	0.00232918	1.00E-200
rs35640778	A	A	-0.209011	0.020757	0.020757	0.00702087	9.59E-195
rs3767952	A	A	0.0134472	0.226709	0.226709	0.00238826	1.80E-08
rs3785074	G	G	0.023863	0.289672	0.289672	0.00220455	2.60E-27
rs3891167	G	G	-0.0425685	0.253435	0.253435	0.00239551	1.20E-70
rs41269079	A	A	0.0153617	0.188991	0.188991	0.0025499	1.70E-09
rs41304832	A	A	0.0611702	0.012378	0.012378	0.0093095	5.00E-11
rs429358	C	C	0.0173498	0.153969	0.153969	0.00277091	3.80E-10
rs4498805	T	T	0.0150601	0.546632	0.453368	0.00200376	5.70E-14
rs4530278	T	T	0.0138793	0.59815	0.40185	0.0020567	1.50E-11
rs45604339	T	T	-0.020433	0.34242	0.34242	0.00211433	4.30E-22
rs4616688	T	T	-0.0173476	0.525394	0.474606	0.00200198	4.50E-18
rs4695407	G	G	0.0141511	0.507843	0.492157	0.00199925	1.50E-12
rs4724	A	A	-0.0547446	0.116598	0.116598	0.00312441	9.80E-69
rs4731541	G	G	-0.0206119	0.624901	0.375099	0.00205962	1.40E-23
rs4743037	T	T	0.0147971	0.230874	0.230874	0.00238094	5.10E-10
rs55747751	A	A	-0.0211612	0.077375	0.077375	0.00375164	1.70E-08
rs56061761	A	A	-0.0203556	0.332642	0.332642	0.0022296	6.90E-20
rs56178008	A	A	0.0143739	0.437497	0.437497	0.00201464	9.70E-13
rs56799554	G	G	-0.0259793	0.170183	0.170183	0.00267858	3.00E-22
rs5742915	C	C	0.0193377	0.445829	0.445829	0.00202886	1.60E-21
rs59409453	G	G	0.0202133	0.730602	0.269398	0.00230175	1.60E-18
rs6007020	C	C	0.0144904	0.367823	0.367823	0.00209637	4.80E-12
rs6054257	A	A	-0.0141684	0.793522	0.206478	0.00247729	1.10E-08

rs611646	A	A	-0.0368309	0.408685	0.408685	0.00203547	3.50E-73
rs61405042	T	T	-0.0501874	0.029288	0.029288	0.00602955	8.50E-17
rs61748181	T	T	-0.059181	0.028928	0.028928	0.00595394	2.80E-23
rs6536702	A	A	0.0534148	0.774647	0.225353	0.00238875	9.40E-111
rs6584579	G	G	0.0114923	0.398876	0.398876	0.00204674	2.00E-08
rs6587577	G	G	-0.0182148	0.826346	0.173654	0.0026359	4.80E-12
rs6590343	G	G	0.0121739	0.516385	0.483615	0.00201444	1.50E-09
rs6659669	T	T	-0.0117091	0.605095	0.394905	0.00205167	1.10E-08
rs6669563	A	A	0.0182358	0.437768	0.437768	0.00202476	2.10E-19
rs66731853	A	A	-0.0177791	0.317304	0.317304	0.00215421	1.50E-16
rs670180	A	A	-0.0115801	0.569104	0.430896	0.00203062	1.20E-08
rs6751209	C	C	-0.0140465	0.204231	0.204231	0.00248465	1.60E-08
rs6776756	A	A	-0.0174439	0.597562	0.402438	0.00203747	1.10E-17
rs6790988	G	G	0.0145728	0.741902	0.258098	0.00228428	1.80E-10
rs6881568	A	A	0.0169256	0.36258	0.36258	0.00207735	3.70E-16
rs7099229	A	A	-0.0153288	0.273301	0.273301	0.00224403	8.40E-12
rs7164950	G	G	0.0129362	0.405979	0.405979	0.00204001	2.30E-10
rs7209057	A	A	0.011819	0.561044	0.438956	0.00202865	5.70E-09
rs7221585	T	T	0.0143271	0.22401	0.22401	0.00247042	6.70E-09
rs73581419	T	T	0.0229838	0.106605	0.106605	0.00324156	1.30E-12
rs73730598	A	A	0.0273632	0.054793	0.054793	0.00439272	4.70E-10
rs75664430	G	G	-0.0235179	0.248028	0.248028	0.0023186	3.60E-24
rs76065543	T	T	0.0342843	0.137527	0.137527	0.00290708	4.20E-32
rs76219171	A	A	0.0359839	0.058433	0.058433	0.00431741	7.80E-17
rs762679	A	A	0.0310104	0.856501	0.143499	0.00285024	1.40E-27
rs76666449	C	C	0.0295125	0.100625	0.100625	0.00333186	8.20E-19
rs7705526	A	A	0.0776022	0.326578	0.326578	0.00216124	1.00E-200
rs77231040	C	C	0.0989303	0.005742	0.005742	0.0134649	2.00E-13
rs7772289	T	T	0.017549	0.503081	0.496919	0.00200003	1.70E-18
rs77732866	A	A	0.0177942	0.137597	0.137597	0.00290595	9.20E-10
rs7790856	T	T	-0.0437199	0.289139	0.289139	0.00220526	1.80E-87
rs78491606	C	C	-0.0756311	0.018433	0.018433	0.00741168	1.90E-24
rs79977579	A	A	0.0281517	0.09555	0.09555	0.00343182	2.30E-16
rs80116508	A	A	-0.0352672	0.062356	0.062356	0.00415151	2.00E-17
rs80324517	A	A	0.0396515	0.048259	0.048259	0.00466286	1.80E-17
rs8102497	A	A	-0.0149654	0.431828	0.431828	0.0020233	1.40E-13
rs8105767	G	G	0.0328384	0.29467	0.29467	0.00220117	2.50E-50
rs869785	C	C	-0.0147303	0.672471	0.327529	0.00212801	4.40E-12
rs871134	T	T	-0.0182986	0.569032	0.430968	0.0020263	1.70E-19
rs932002	T	T	-0.0402052	0.150843	0.150843	0.00279667	7.30E-47
rs9398196	G	G	-0.0143586	0.52005	0.47995	0.00201175	9.50E-13
rs939916	A	A	0.0241795	0.669967	0.330033	0.00216724	6.60E-29
rs9419958	C	C	-0.0810098	0.86139	0.13861	0.00293847	2.60E-167

rs9600019	T	T	0.0127134	0.335579	0.335579	0.00213096	2.40E-09
rs9878436	T	T	-0.0143407	0.434393	0.434393	0.00201819	1.20E-12
rs9940099	T	T	-0.033609	0.062718	0.062718	0.00411613	3.20E-16
rs9955360	A	A	-0.0190311	0.869288	0.130712	0.00299791	2.20E-10

The variants together explain 3.4% of the variation in Telomere Length (R^2 = 0.034).
F-statistics = 112.797
EAF: Effect Allele Frequency

Supplementary Table-14 Characteristics of the single nucleotide polymorphisms (SNP) used as instrumental variables for DNA methylation GrimAge Acceleration

SNP	Effect Allele	Other Allele	Effect (BETA)	EAF	MAF	Standard Error	P-Value
rs1008084	A	G	0.251305	0.449294	0.449294	0.0334254	4.66E-15
rs28364475	T	C	0.0644384	0.05437	0.05437	0.111228	8.41E-13
rs2858830	A	G	0.158434	0.584715	0.415285	0.0360635	1.81E-10
rs4710069	G	A	0.112496	0.360576	0.360576	0.038769	6.51E-10
rs56298289	A	G	0.0392917	0.267686	0.267686	0.0391327	3.32E-10
rs613131	C	T	-0.186522	0.114473	0.114473	0.0441029	2.21E-08
rs78784579	A	T	0.388305	0.035317	0.035317	0.0810607	1.94E-10

The variants together explain 3.8% of the variation in DNA methylation GrimAge Acceleration (R^2 = 0.038).
F-statistics = 195.945
EAF: Effect Allele Frequency

Supplementary Figure-1 Leave-one-out Analysis, Scatter Plot, Forest Plot, and Funnel Plot of Alzheimer's Disease on Facial Aging

Supplementary Figure-1A Leave-one-out Analysis

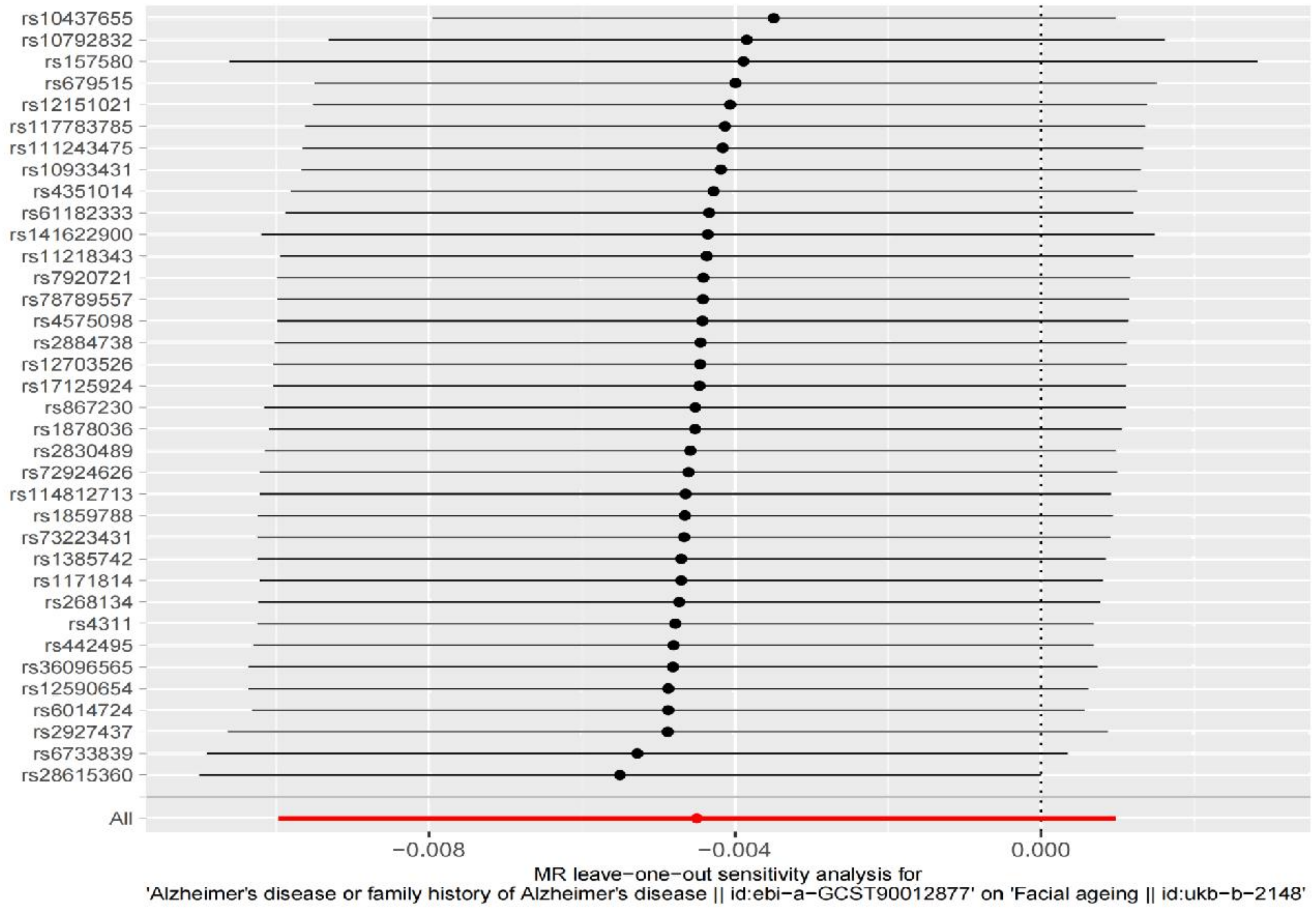

Supplementary Figure-1B Scatter Plot

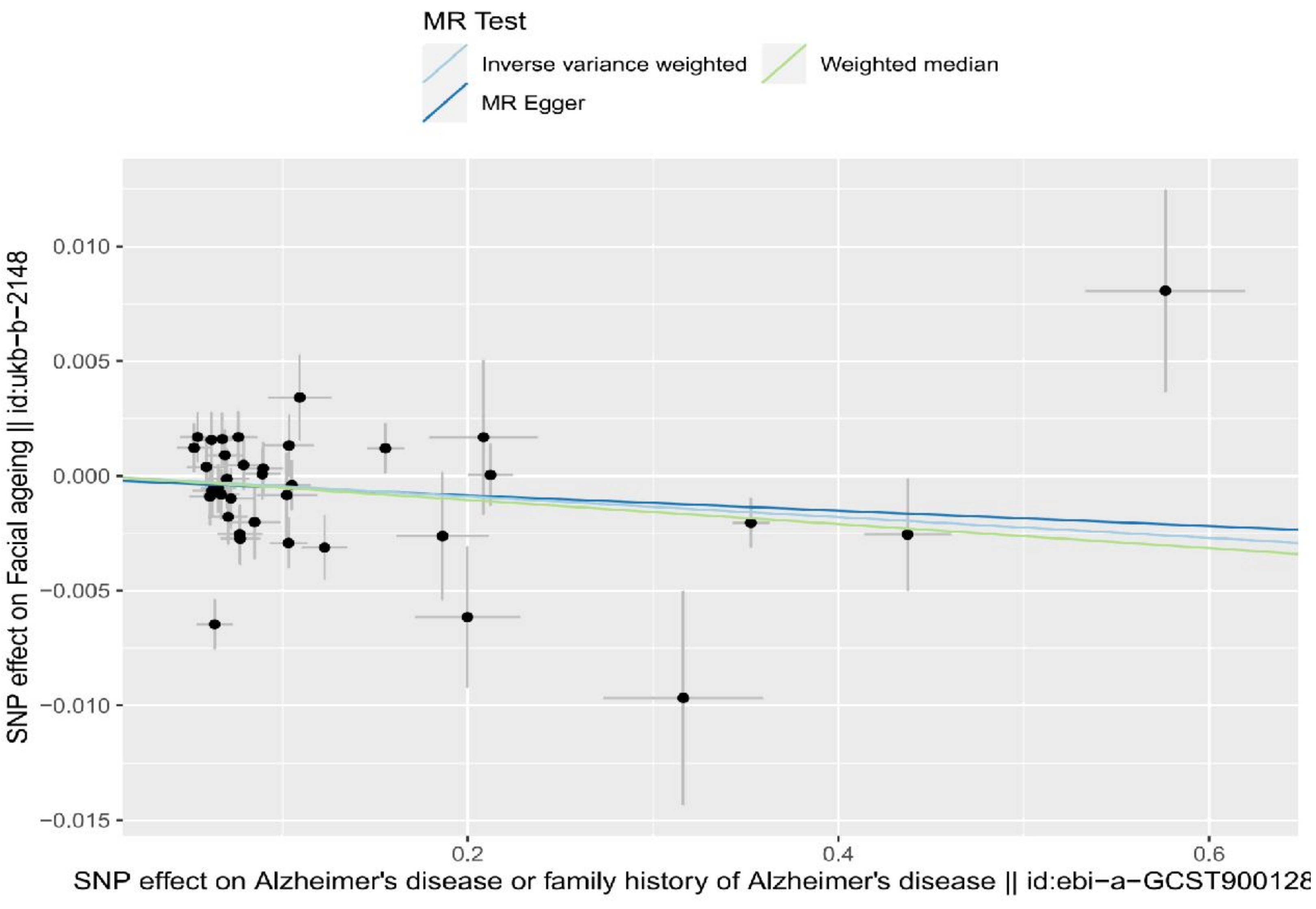

Supplementary Figure-1C Forest Plot

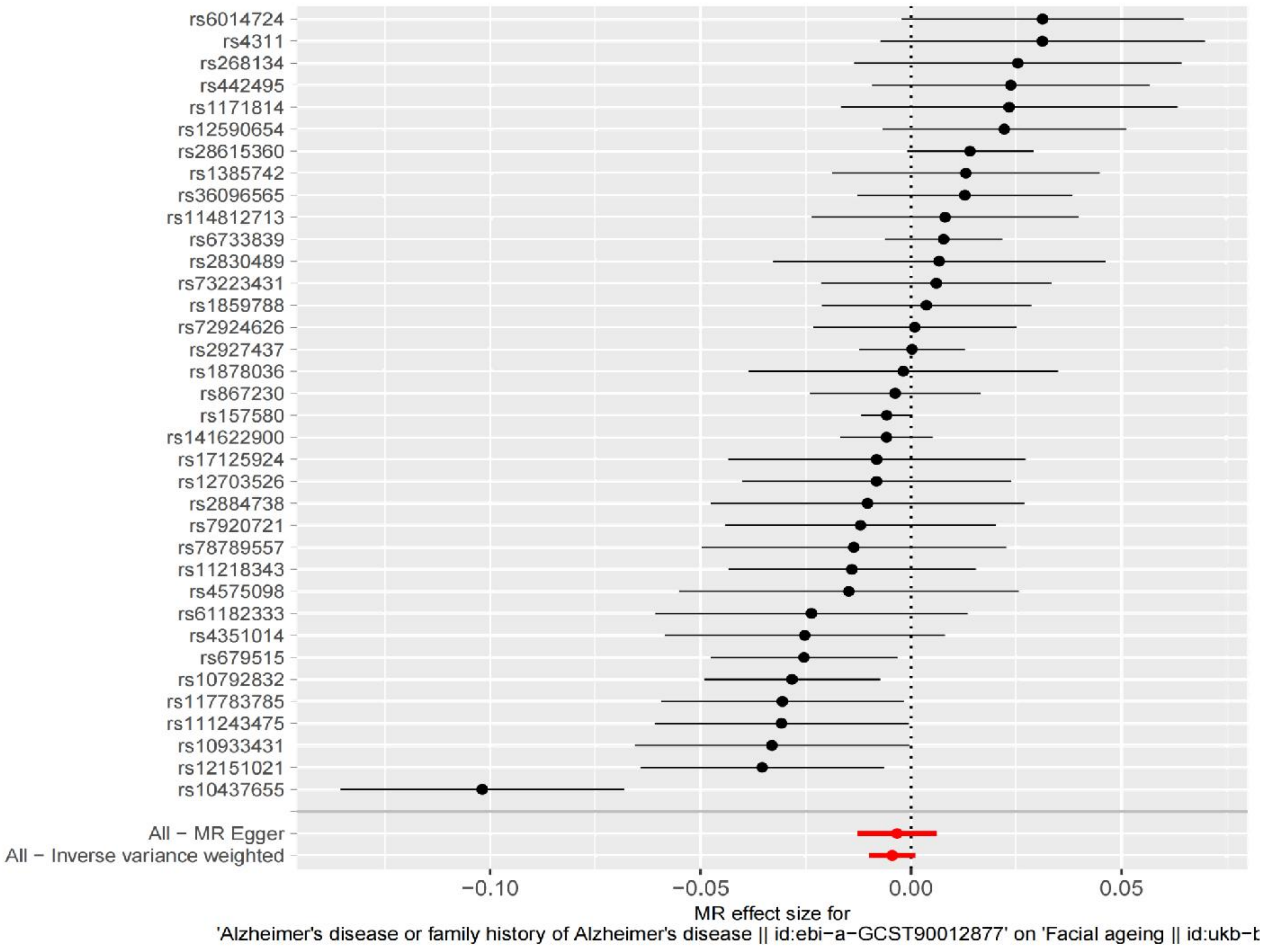

Supplementary Figure-1D Funnel Plot

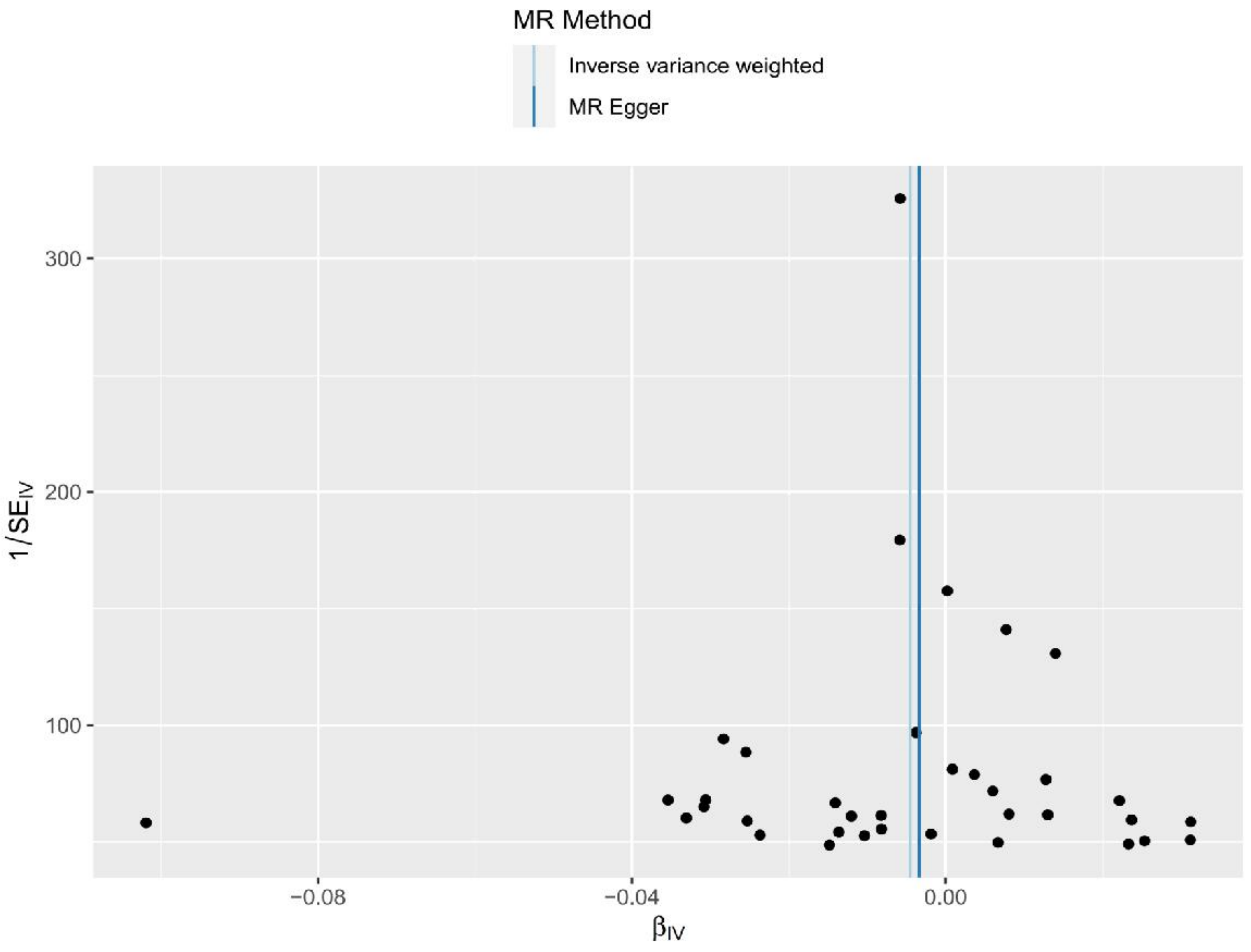

Supplementary Figure-2 Leave-one-out Analysis, Scatter Plot, Forest Plot, and Funnel Plot of Alzheimer's Disease on Frailty Index

Supplementary Figure-2A Leave-one-out Analysis

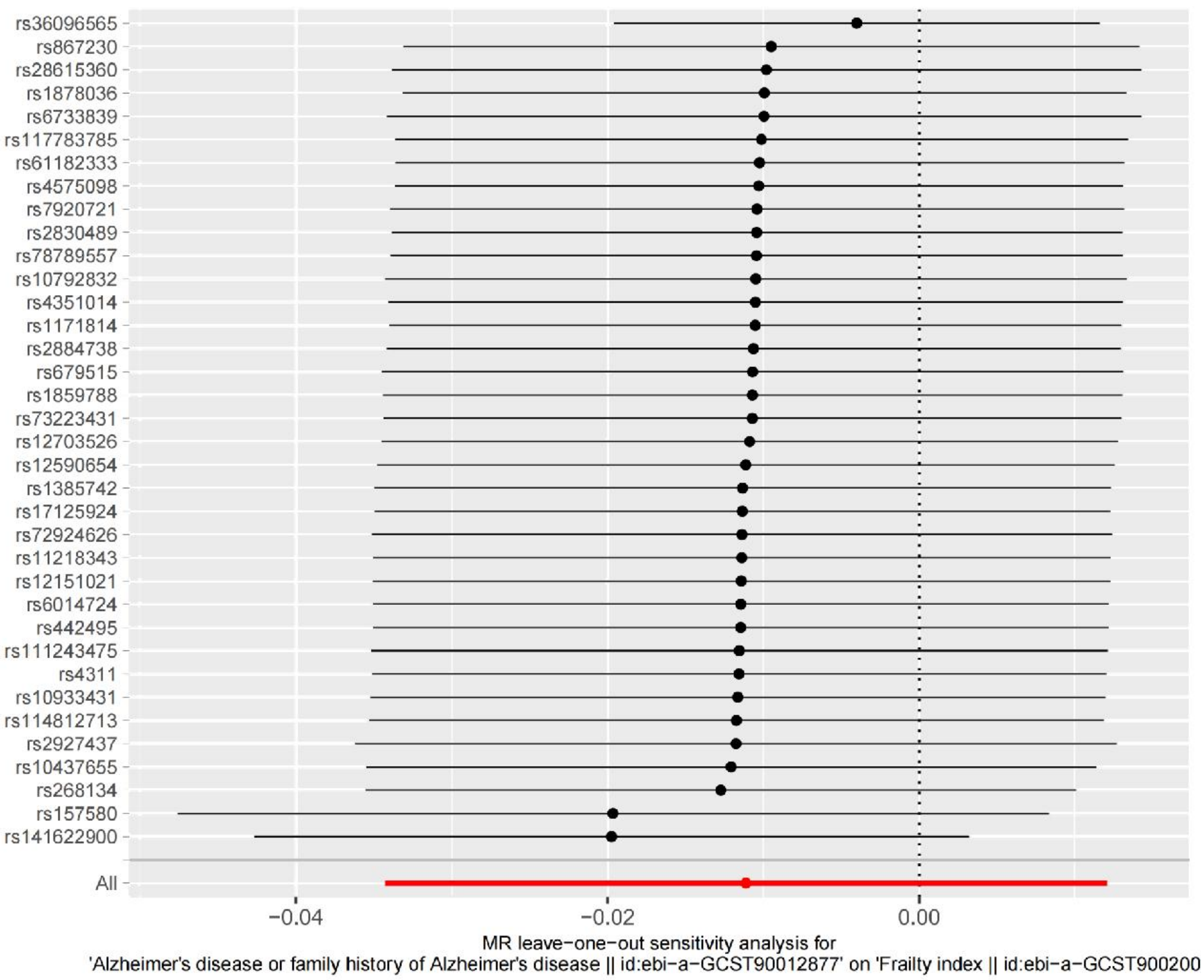

Supplementary Figure-2B Scatter

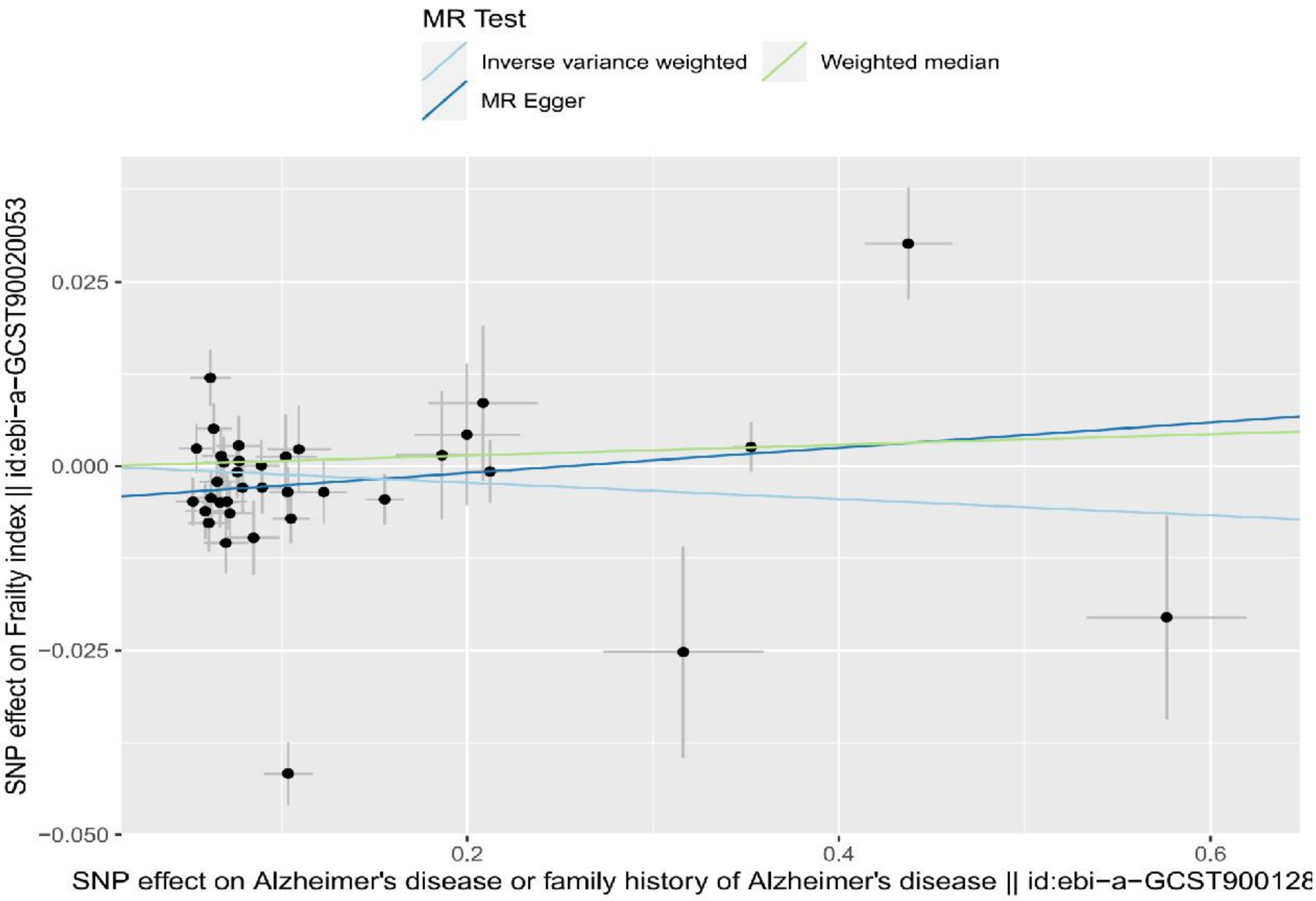

Plot

Supplementary Figure-2C Forest Plot

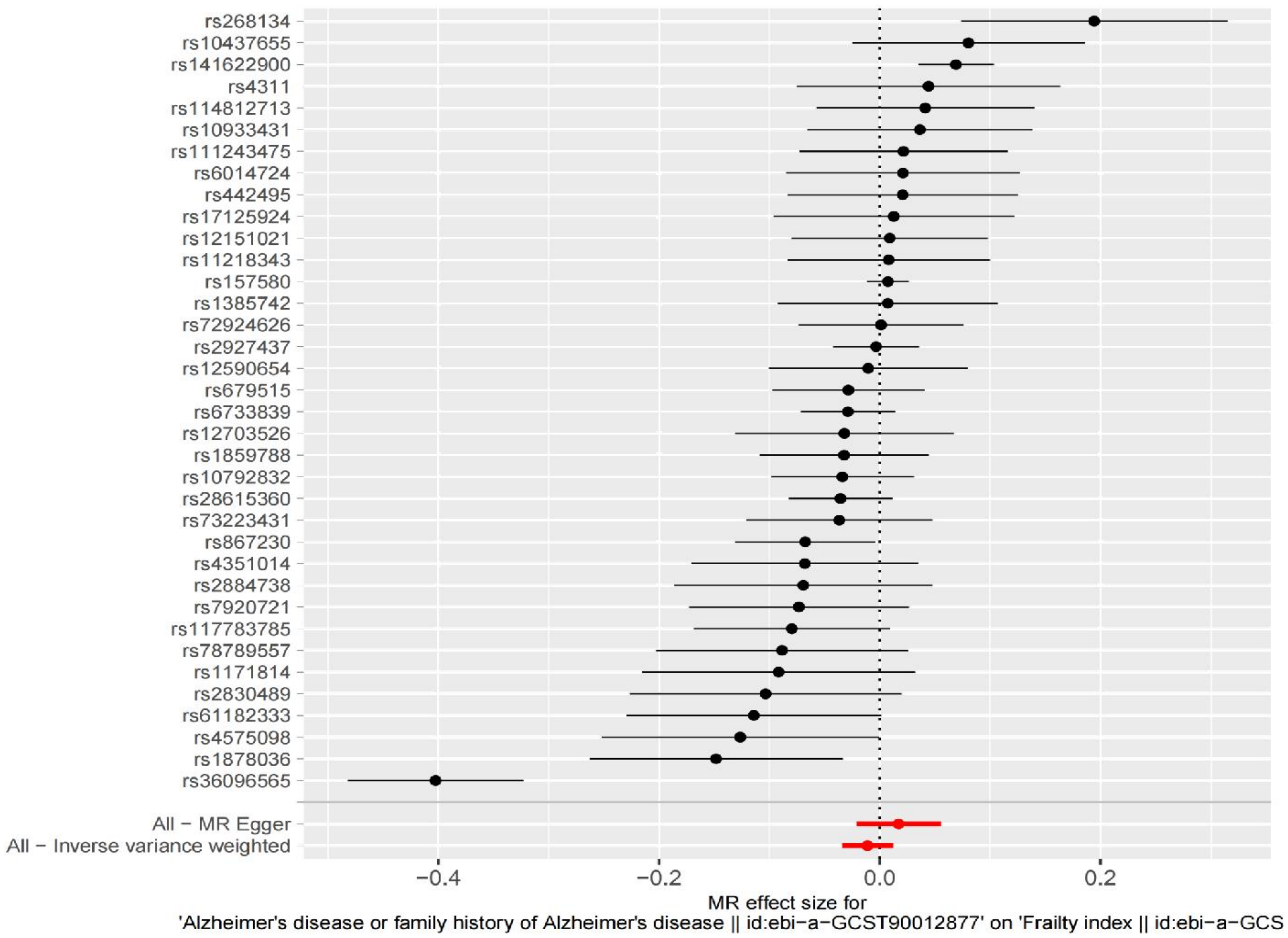

Supplementary Figure-2D Funnel Plot

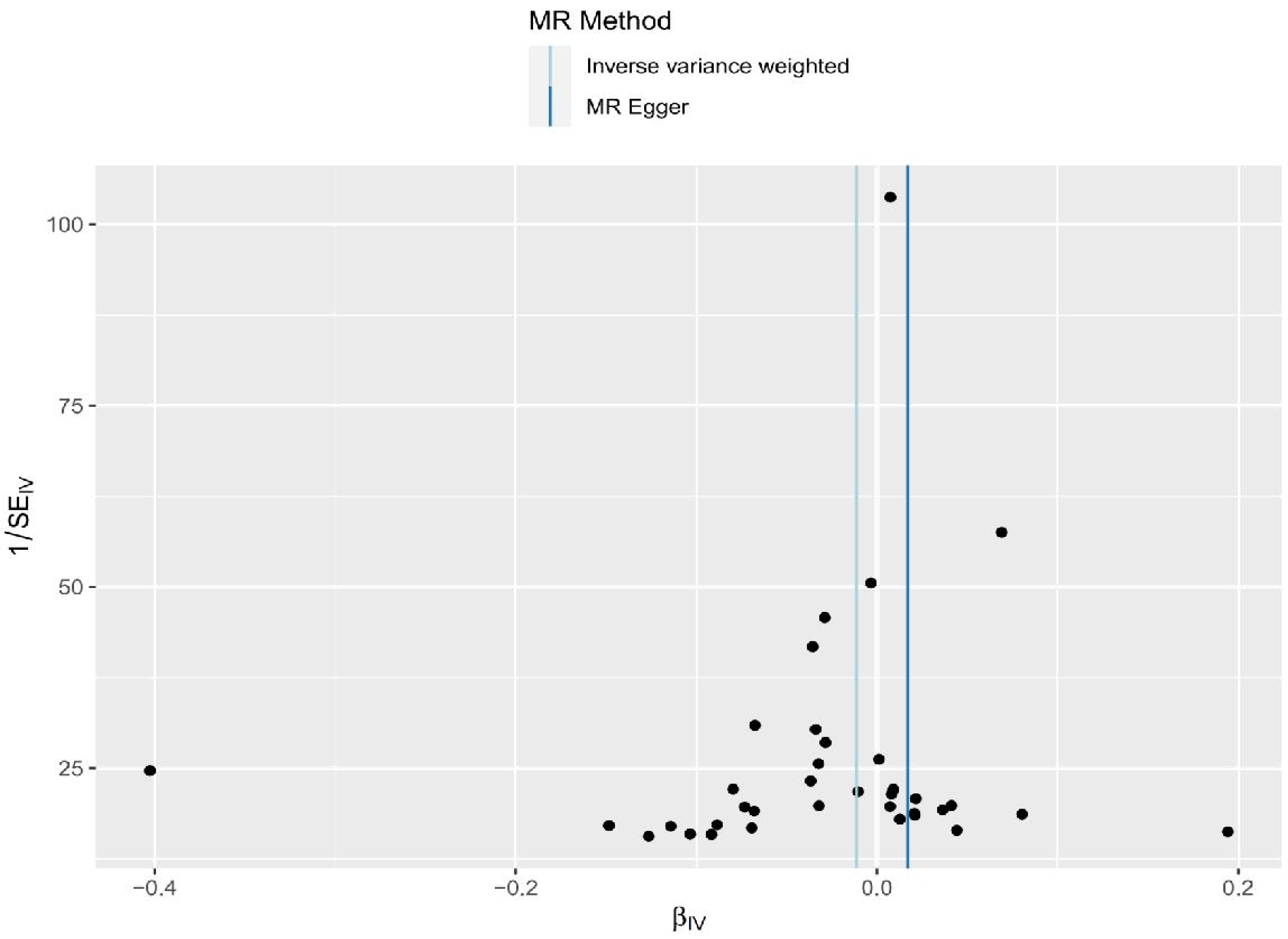

Supplementary Figure-3 Leave-one-out Analysis, Scatter Plot, Forest Plot, and Funnel Plot of Alzheimer's Disease on DNA methylation GrimAge Acceleration

Supplementary Figure-3A Leave-one-out Analysis

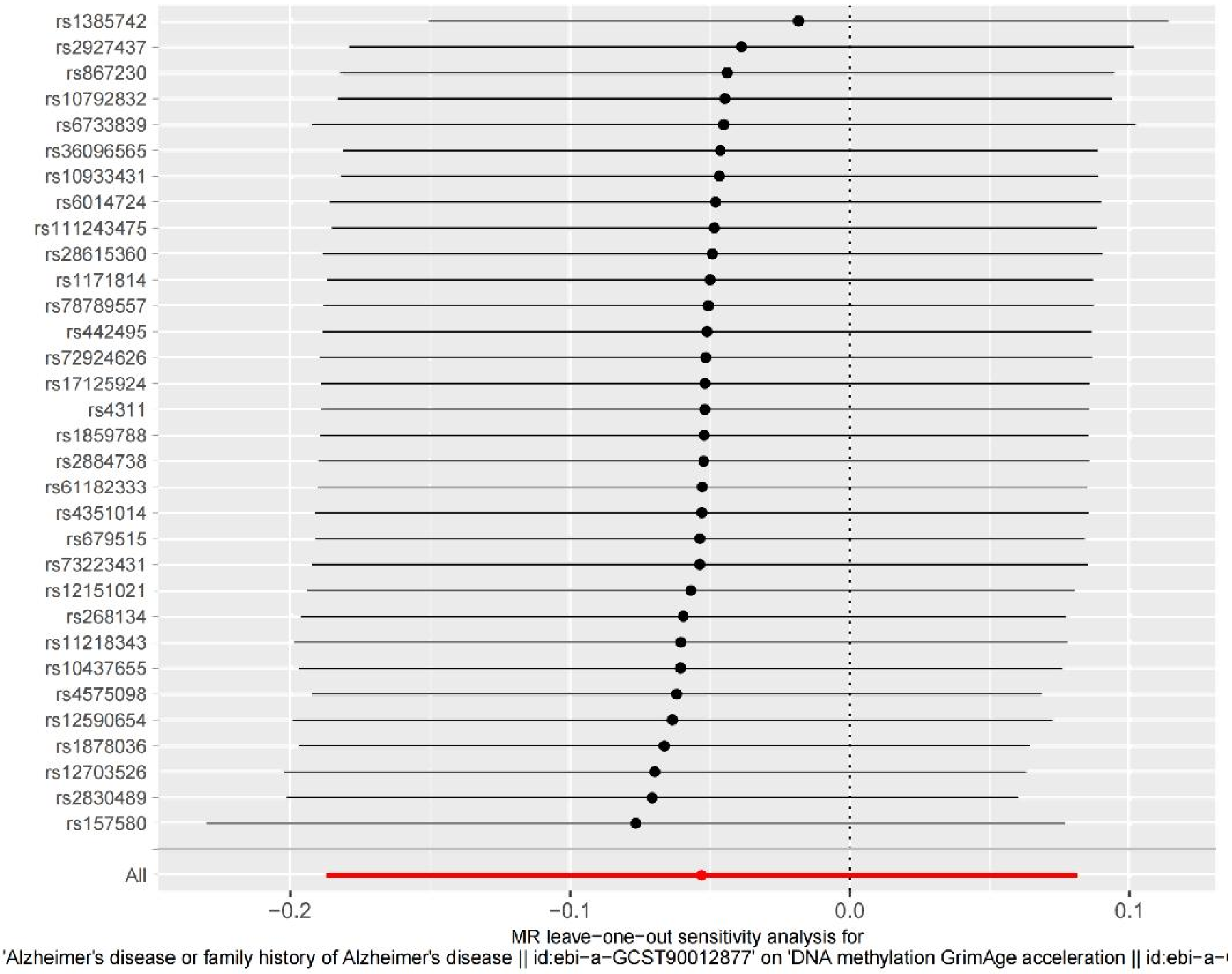

Supplementary Figure-3B Scatter

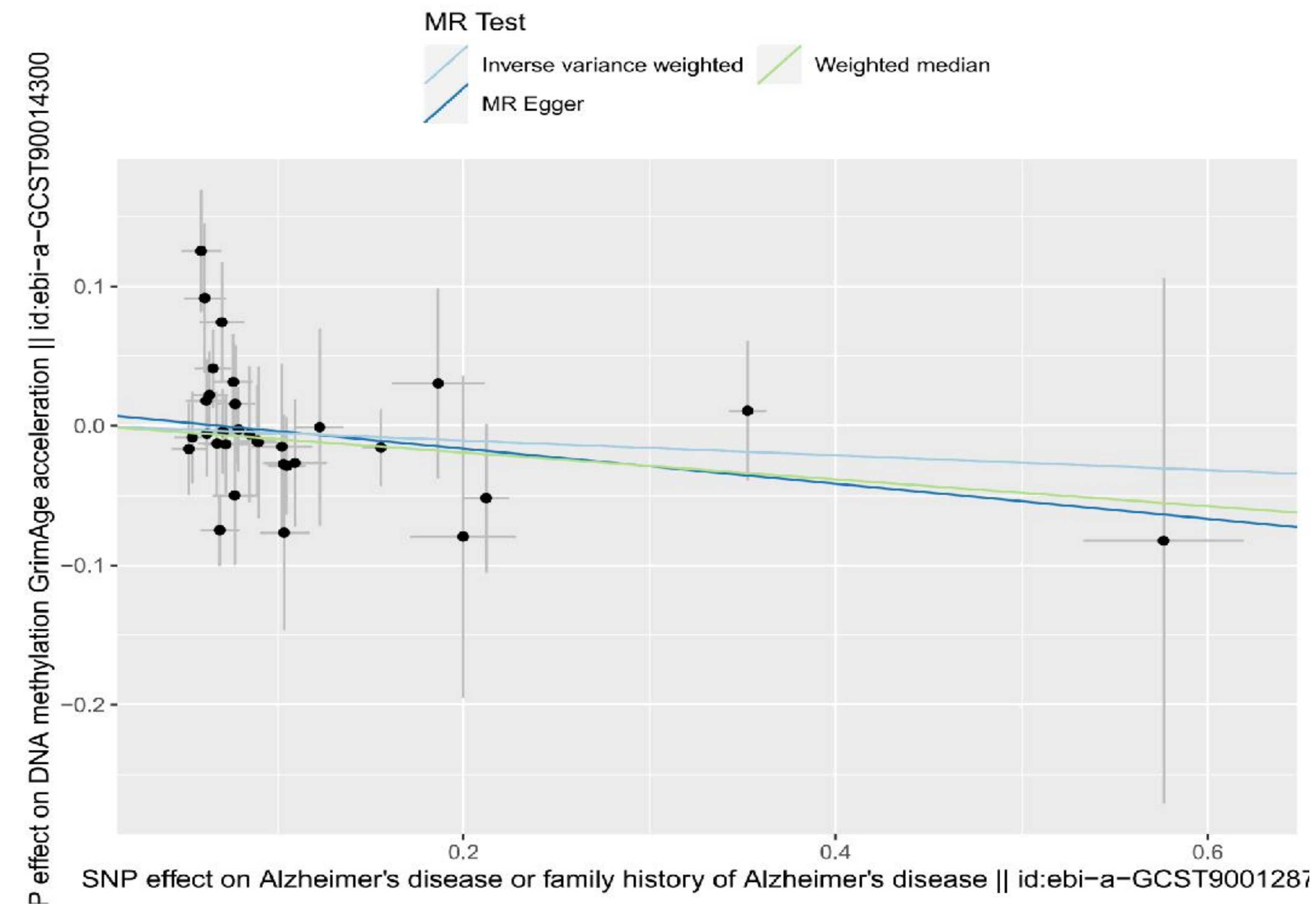

Supplementary Figure-3C Forest Plot

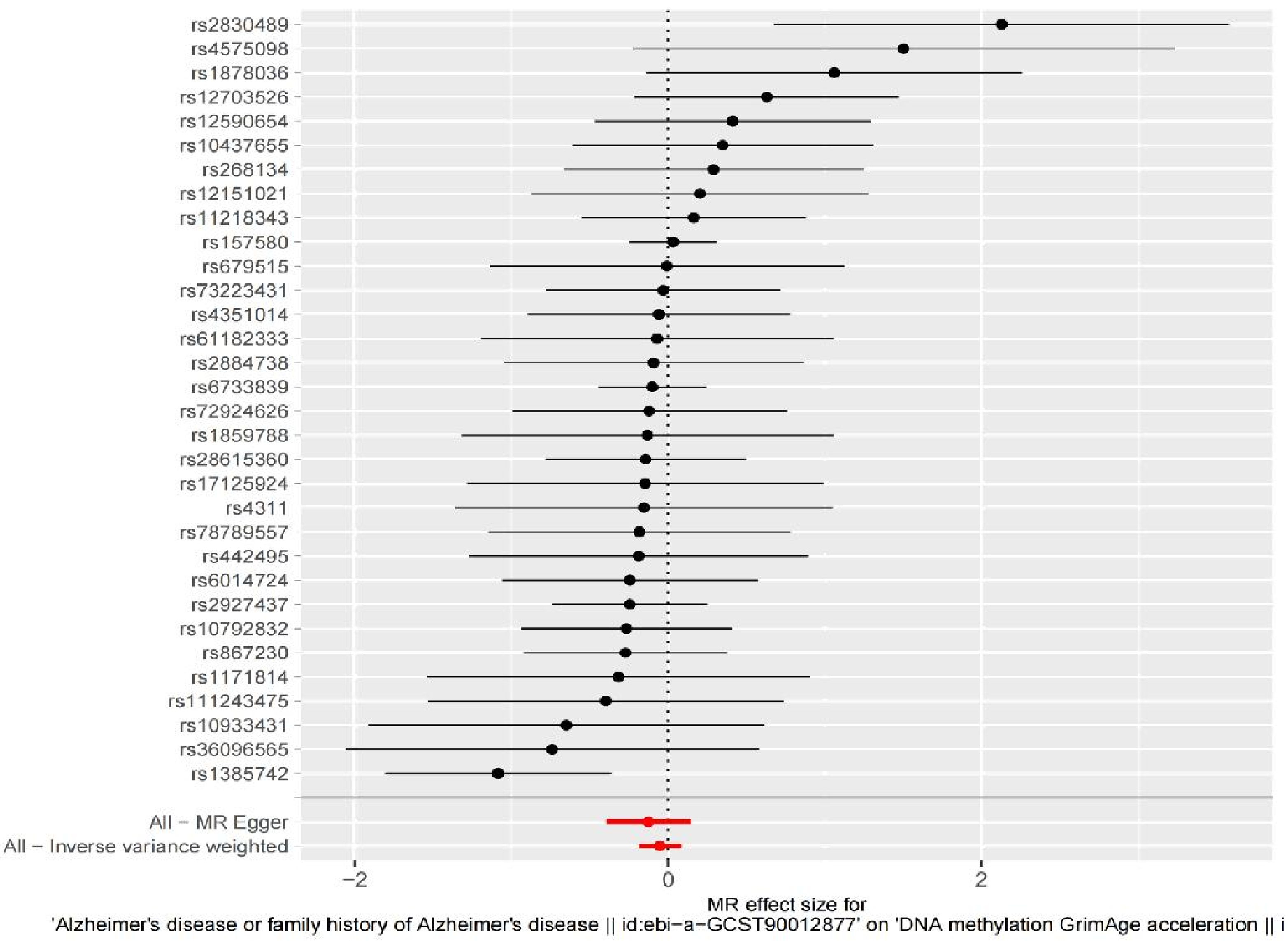

Supplementary Figure-3D Funnel Plot

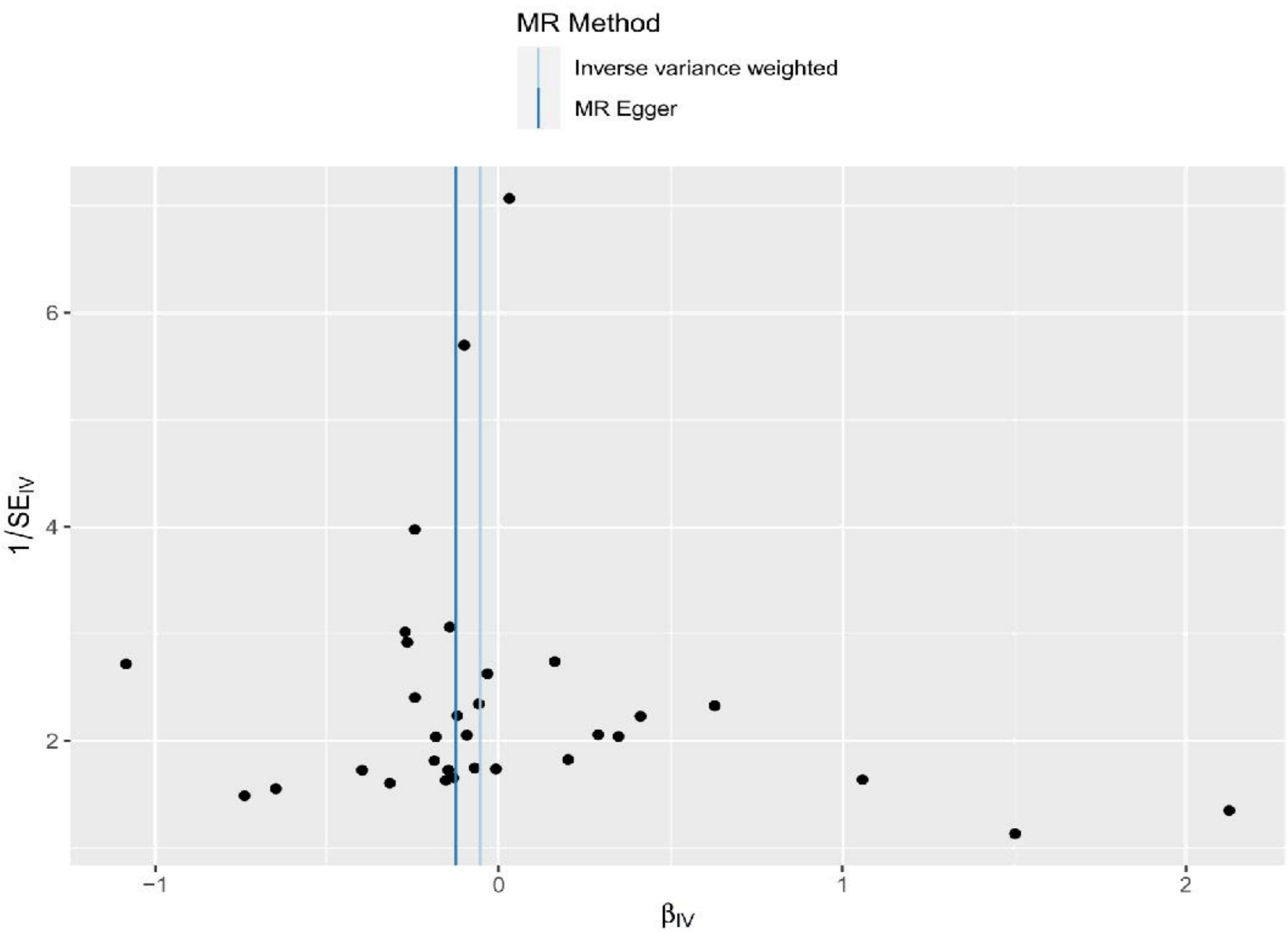

Supplementary Figure-4 Leave-one-out Analysis, Scatter Plot, Forest Plot, and Funnel Plot of Alzheimer's Disease on Telomere Length

Supplementary Figure-4A Leave-one-out Analysis

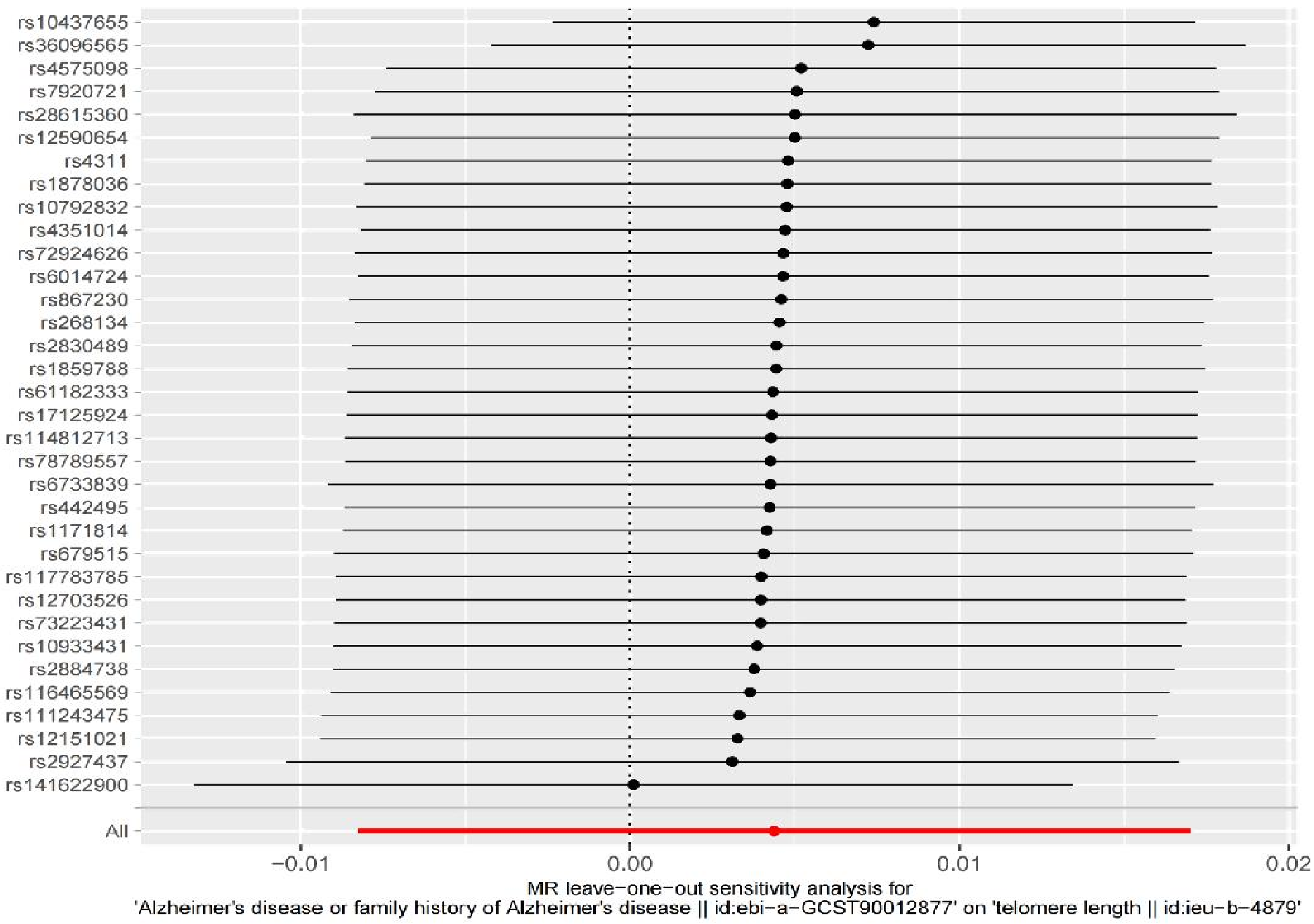

Supplementary Figure-4B Scatter

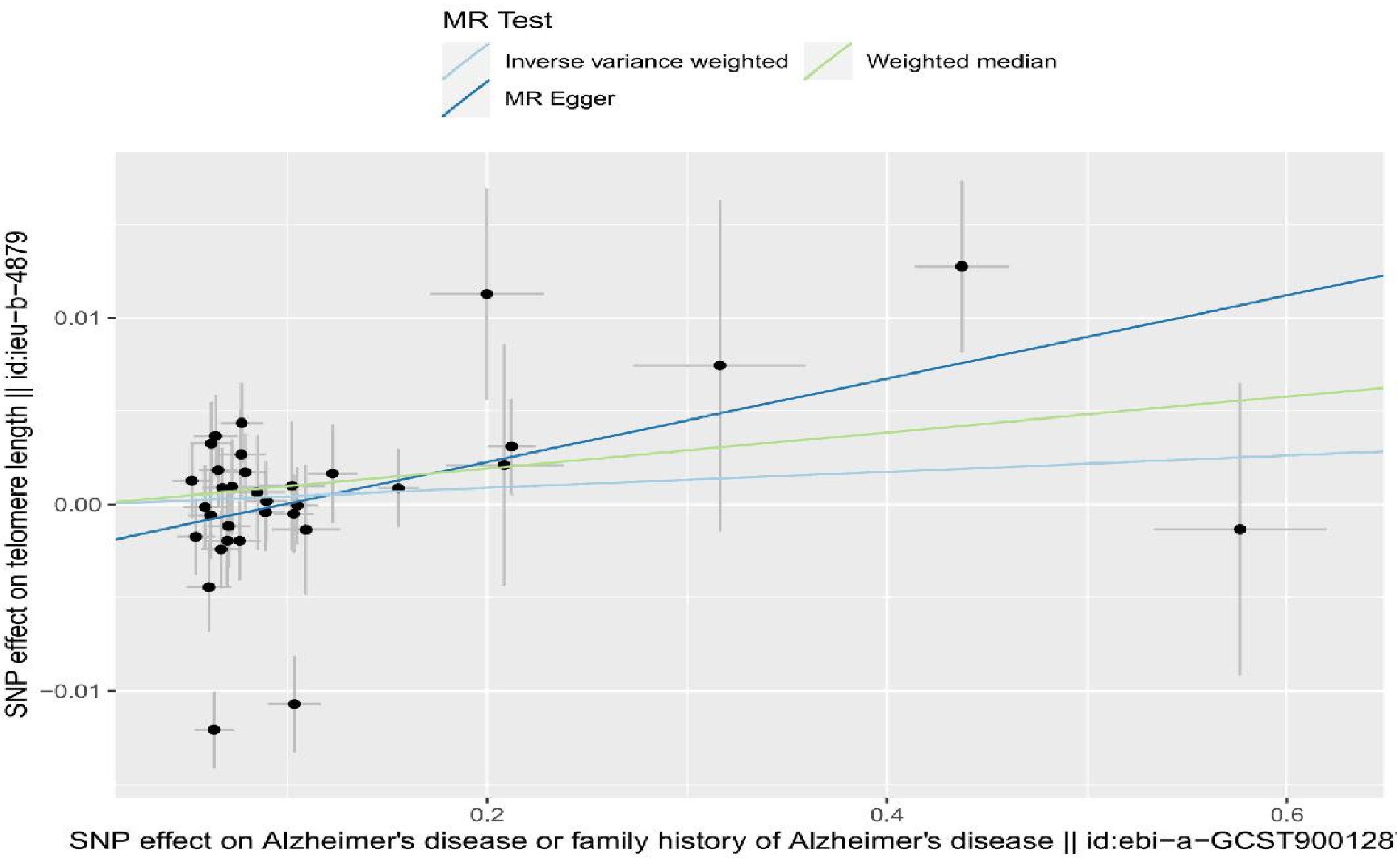

Supplementary Figure-4C Forest Plot

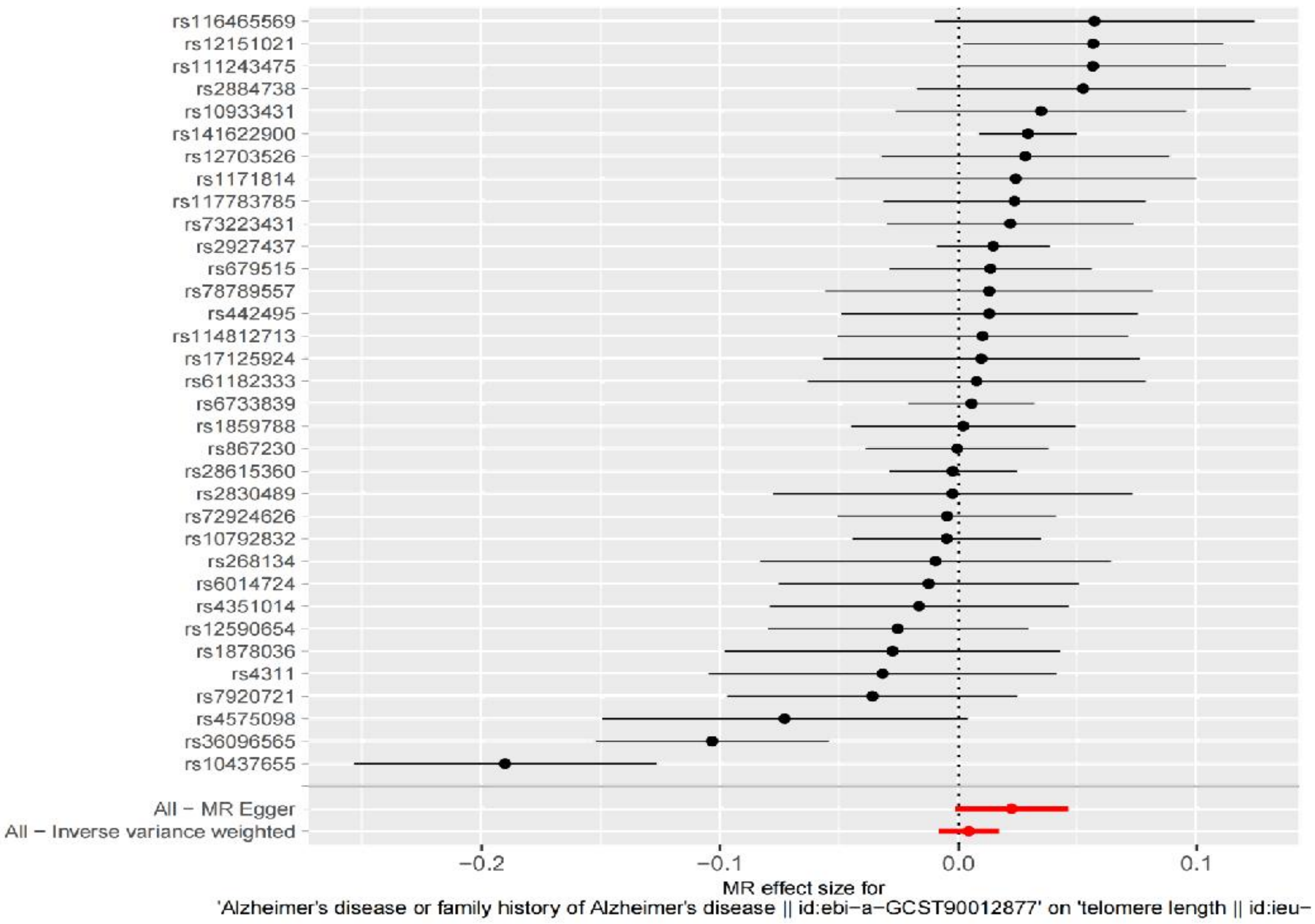

Supplementary Figure-4D Funnel Plot

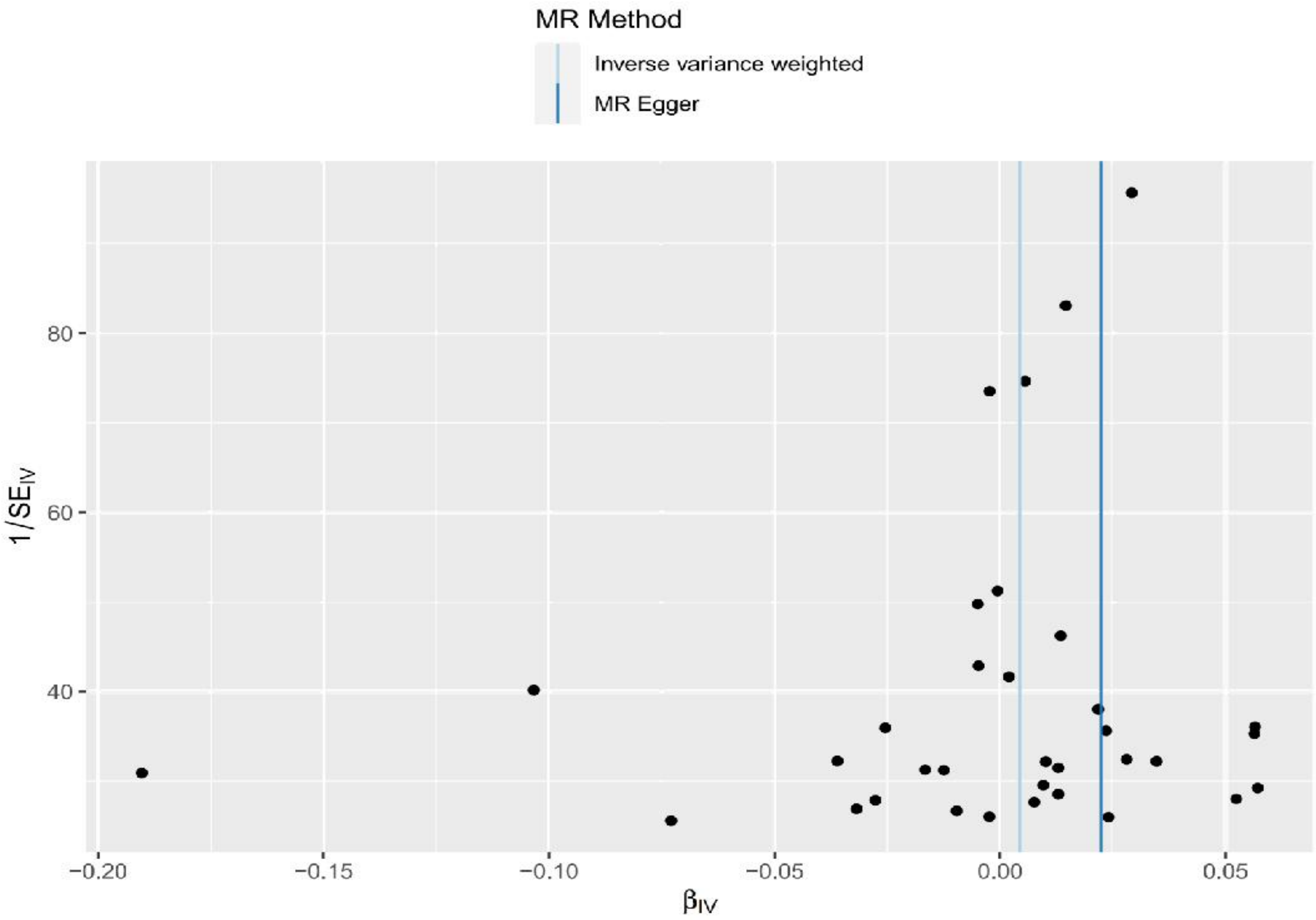

Supplementary Figure-5 Leave-one-out Analysis, Scatter Plot, Forest Plot, and Funnel Plot of All Glioma on Facial Aging

Supplementary Figure-5A Leave-one-out Analysis

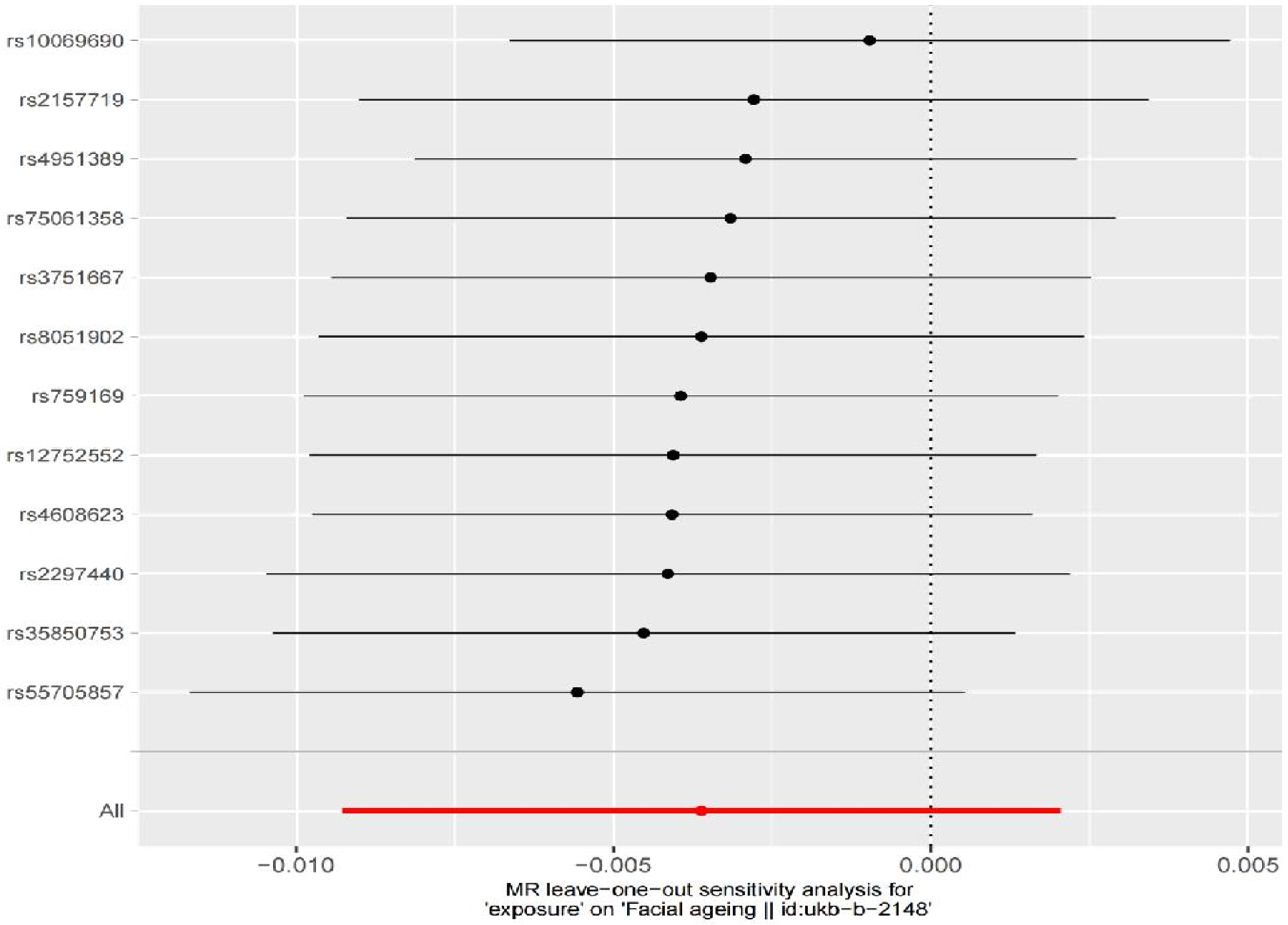

Supplementary Figure-5B Scatter

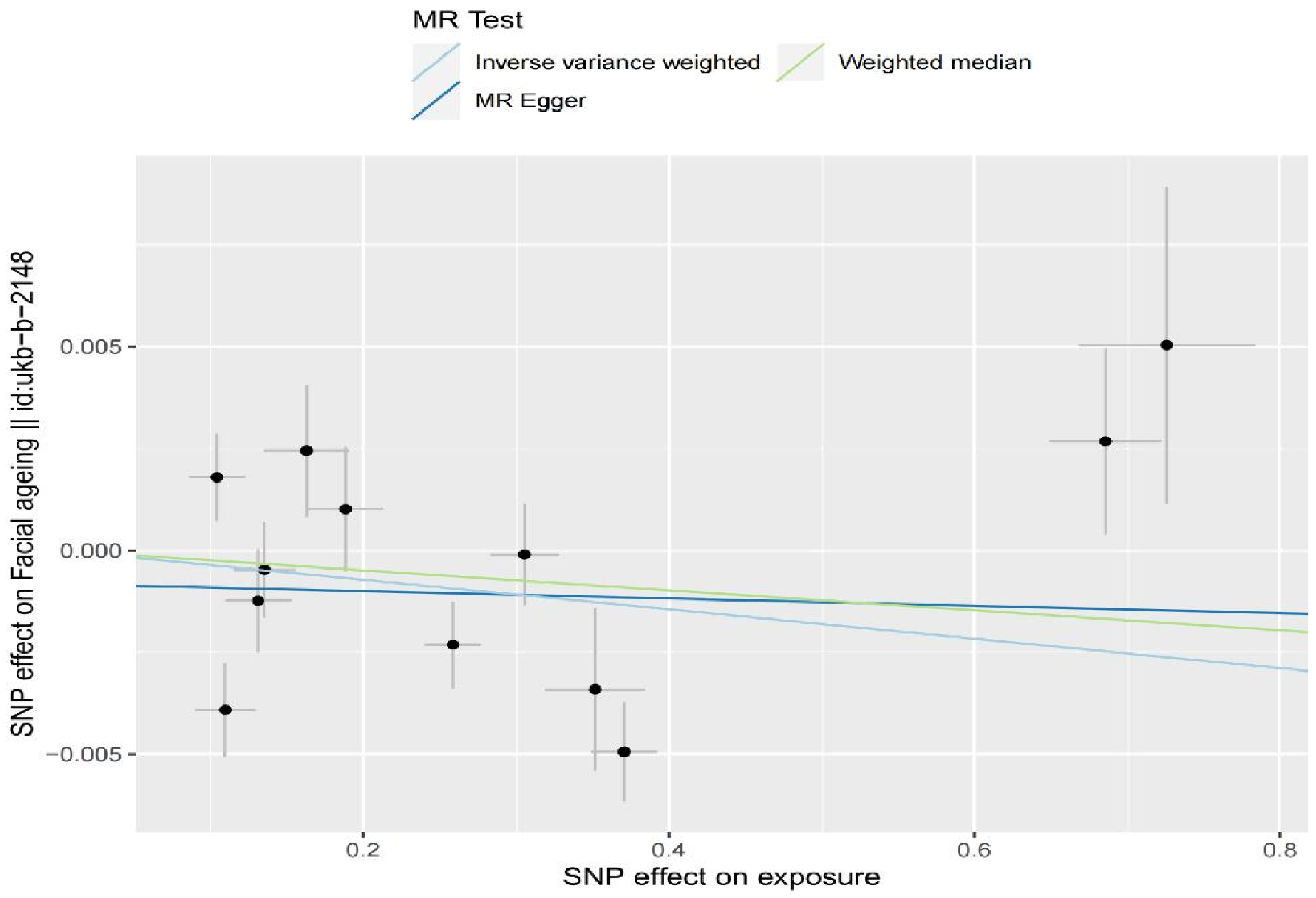

Supplementary Figure-5C Forest Plot

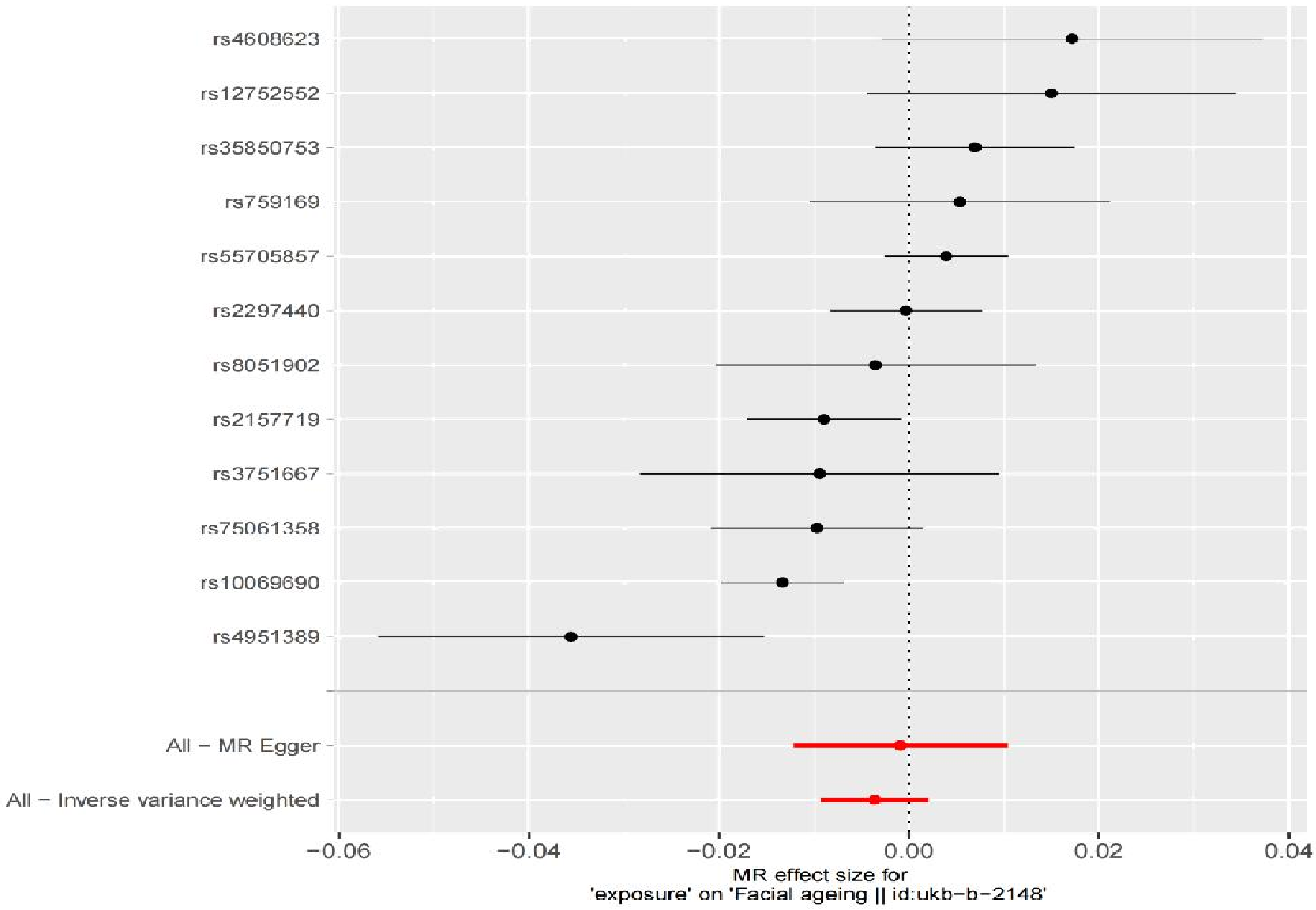

Supplementary Figure-5D Funnel Plot

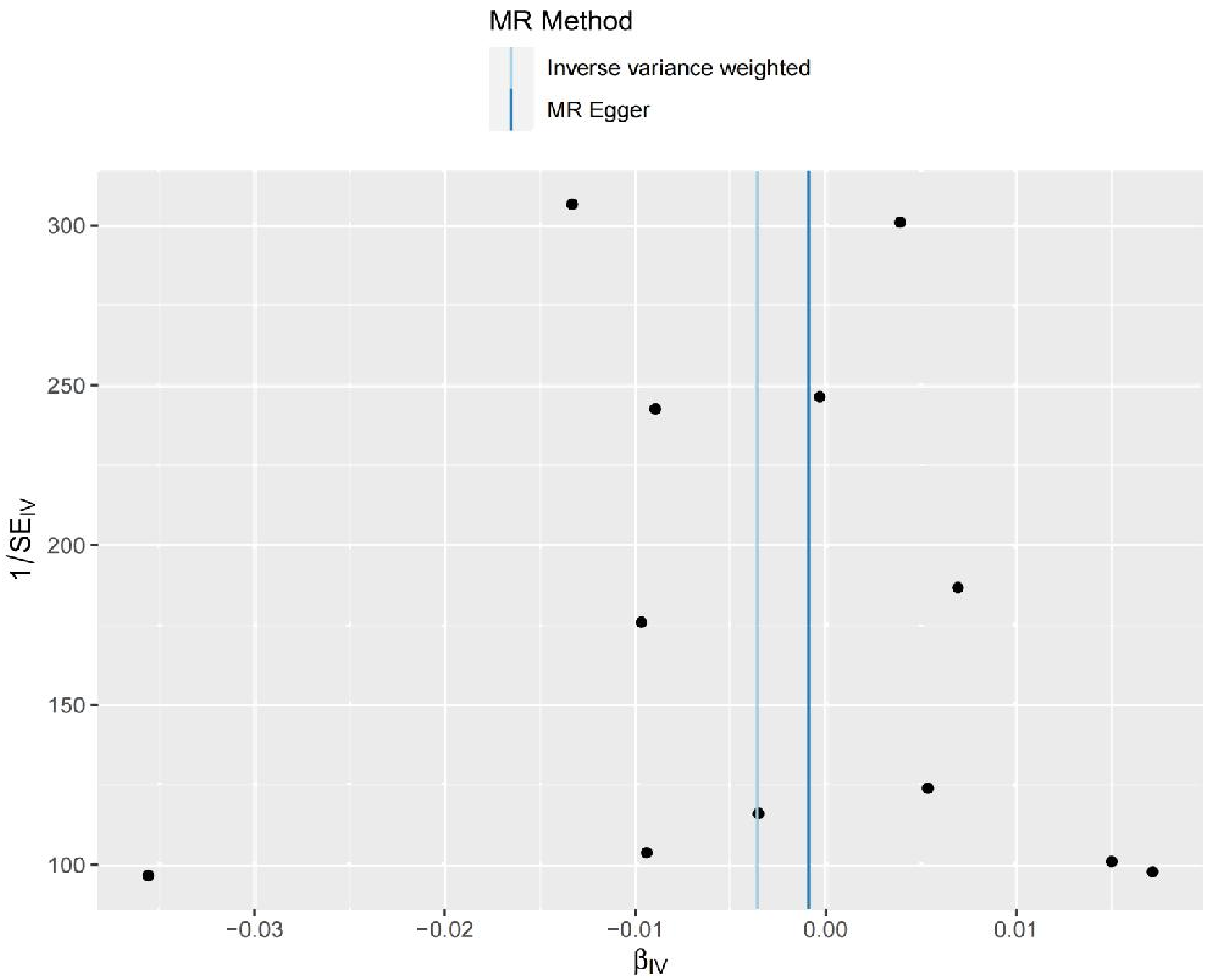

Supplementary Figure-6 Leave-one-out Analysis, Scatter Plot, Forest Plot, and Funnel Plot of All Glioma on Frailty Index

Supplementary Figure-6A Leave-one-out Analysis

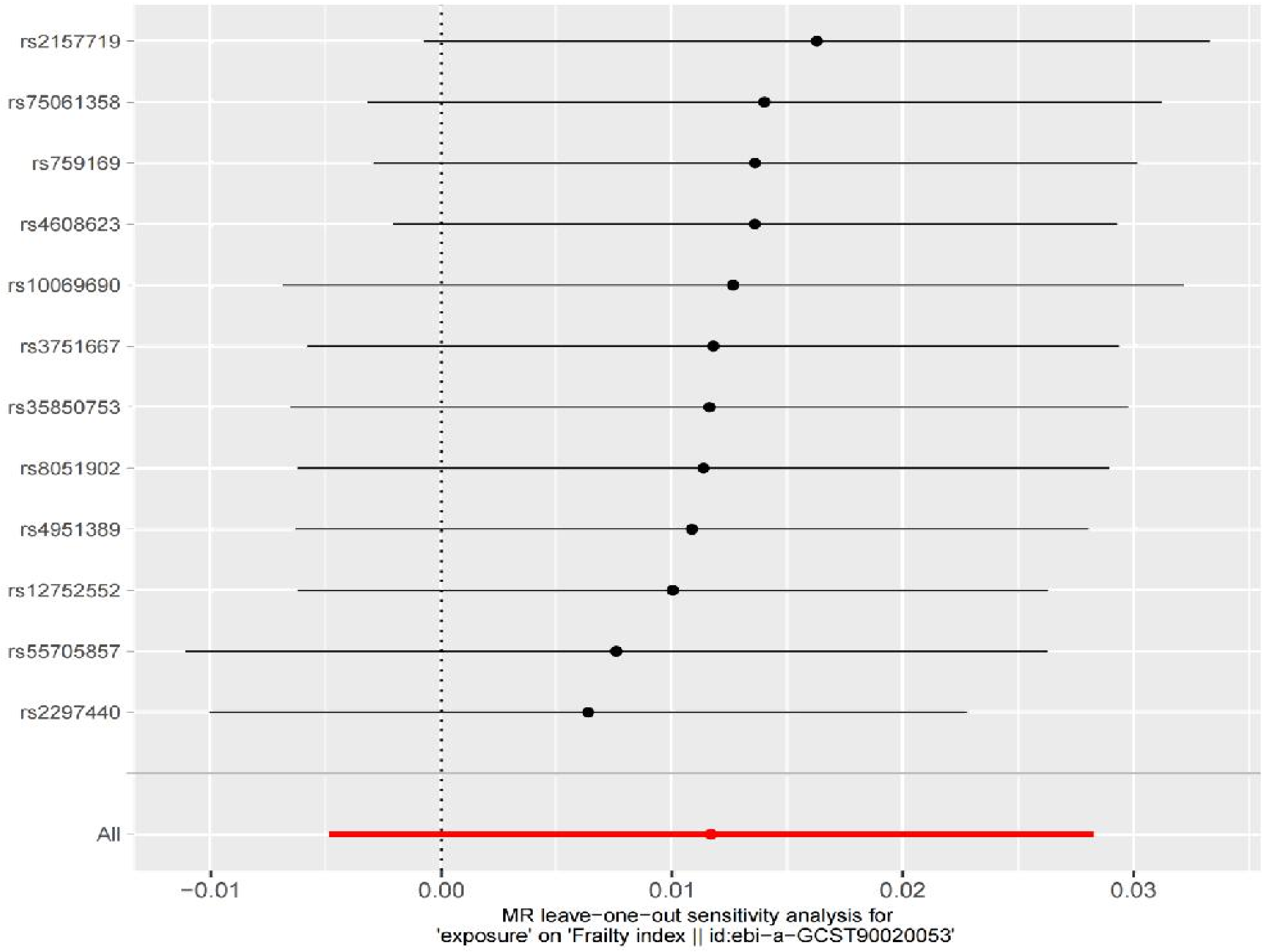

Supplementary Figure-6B Scatter

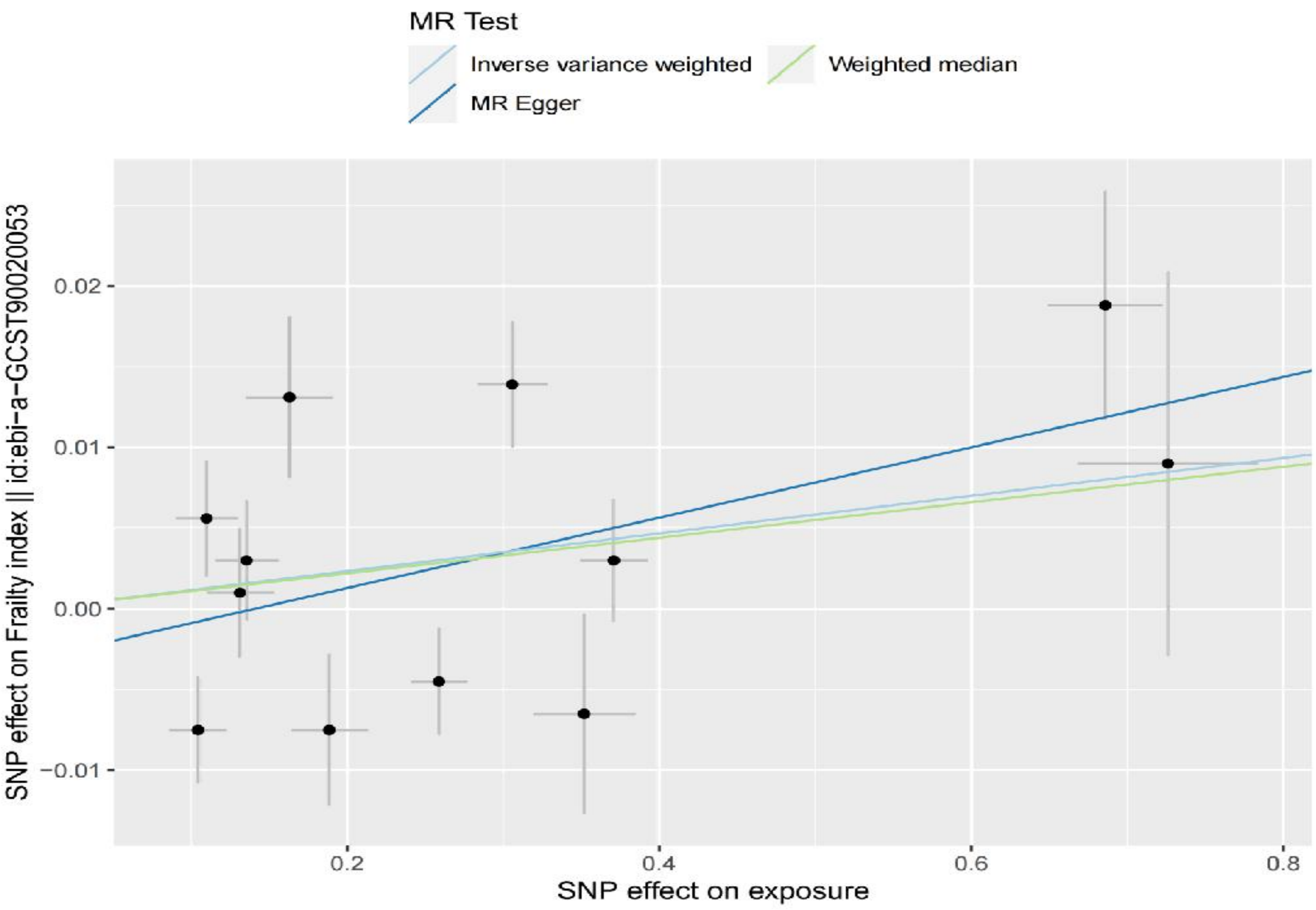

Supplementary Figure-6C Forest Plot

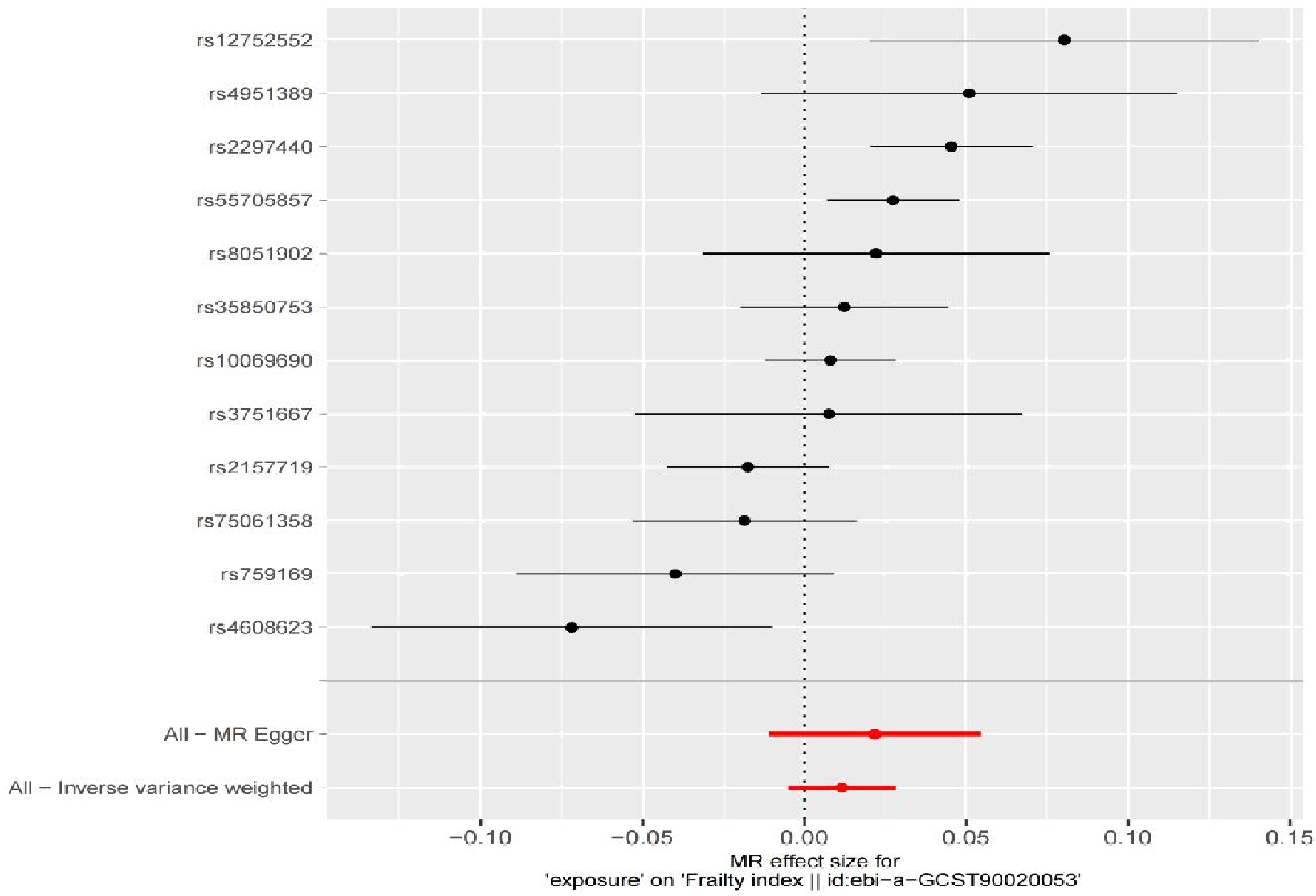

Supplementary Figure-6D Funnel Plot

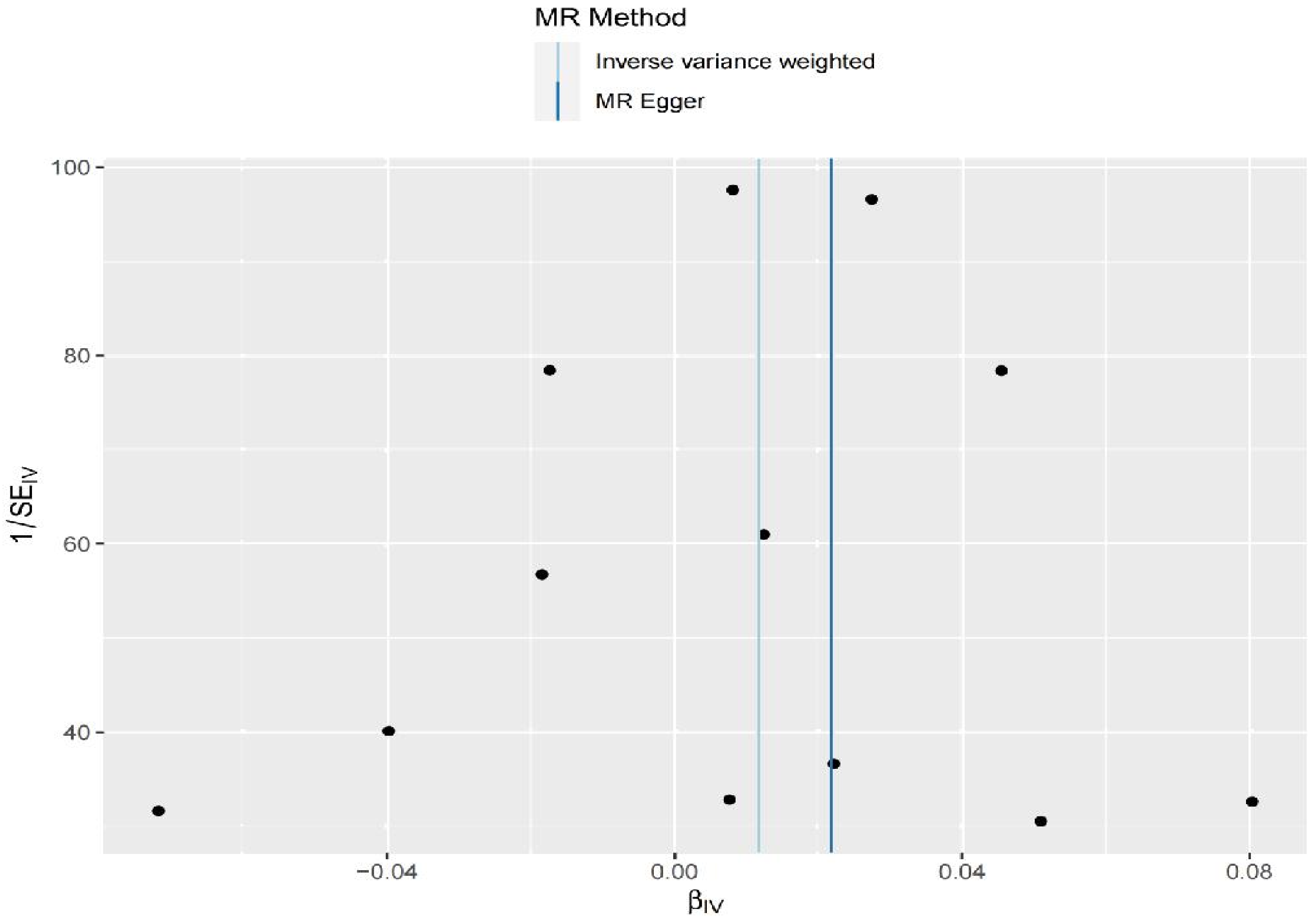

Supplementary Figure-7 Leave-one-out Analysis, Scatter Plot, Forest Plot, and Funnel Plot of All Glioma on DNA methylation GrimAge Acceleration

Supplementary Figure-7A Leave-one-out Analysis

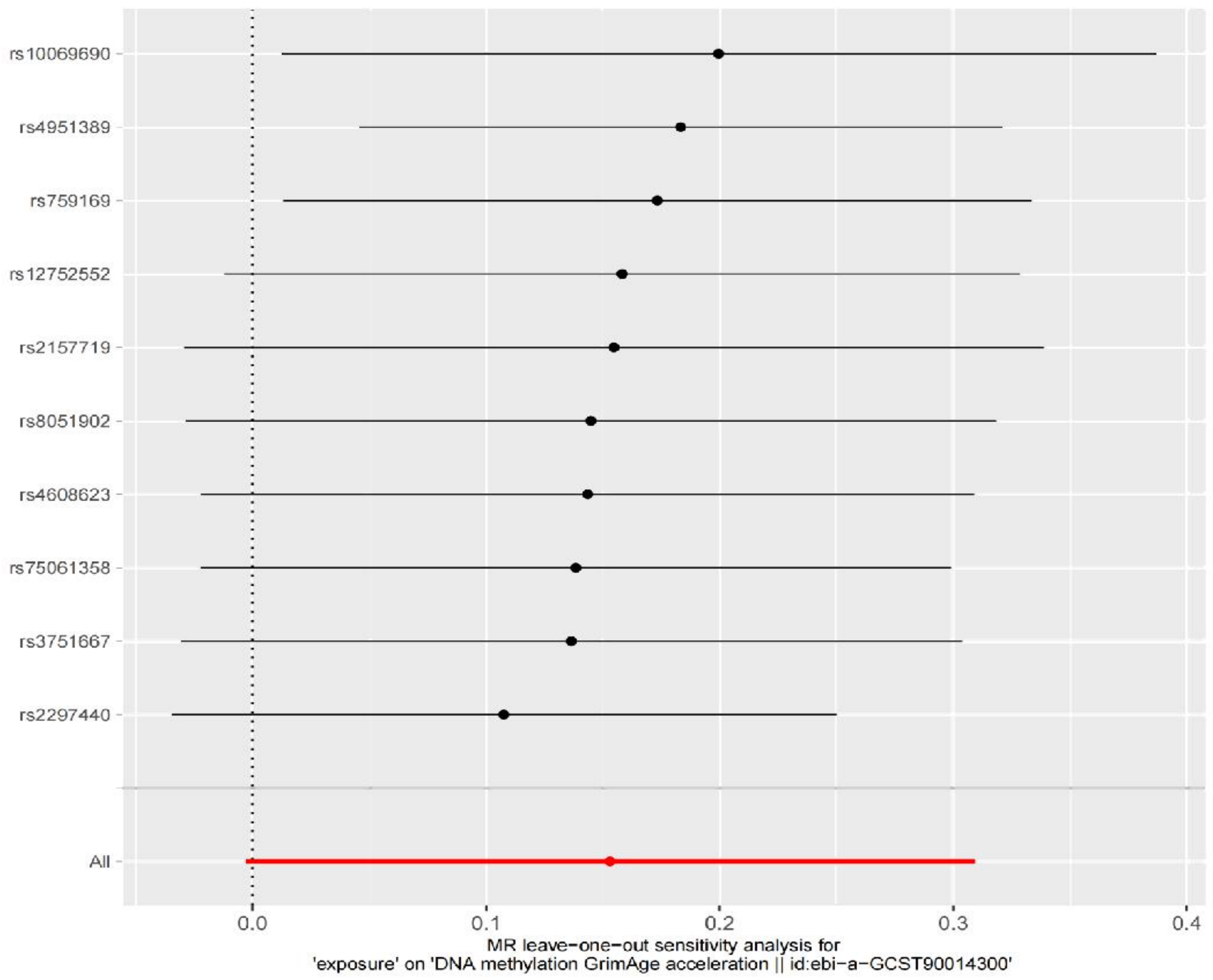

Supplementary Figure-7B Scatter

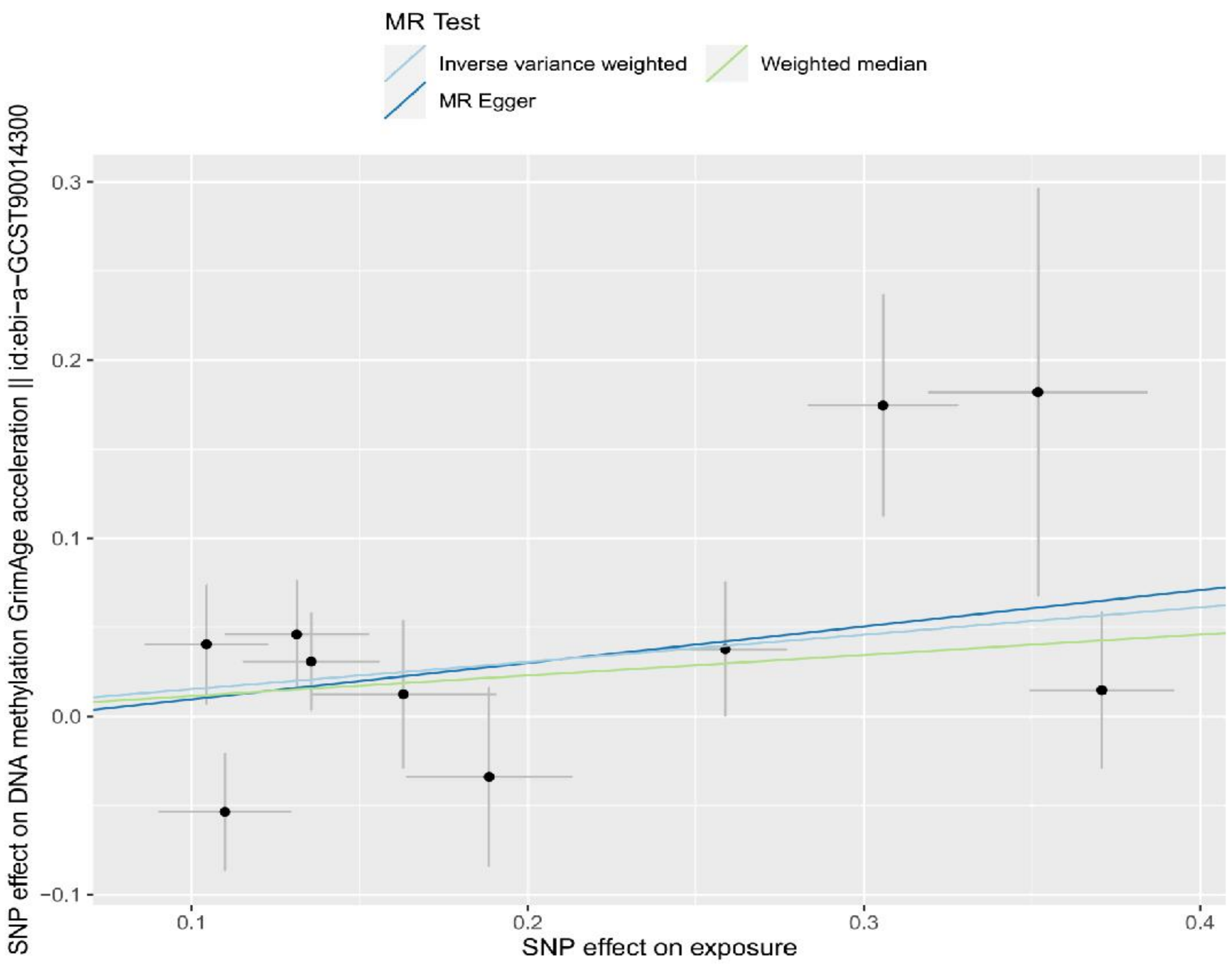

Supplementary Figure-7C Forest Plot

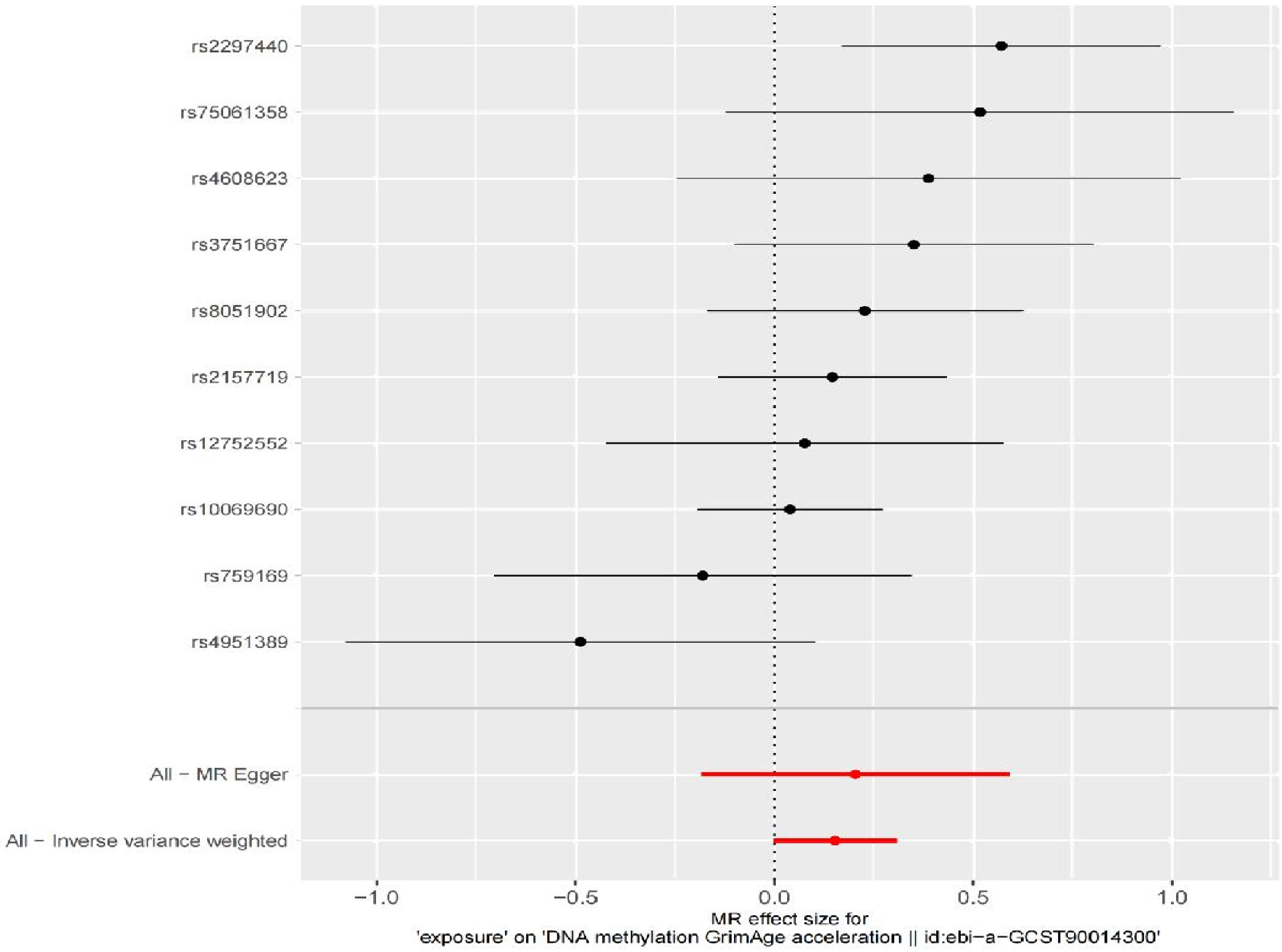

Supplementary Figure-7D Funnel Plot

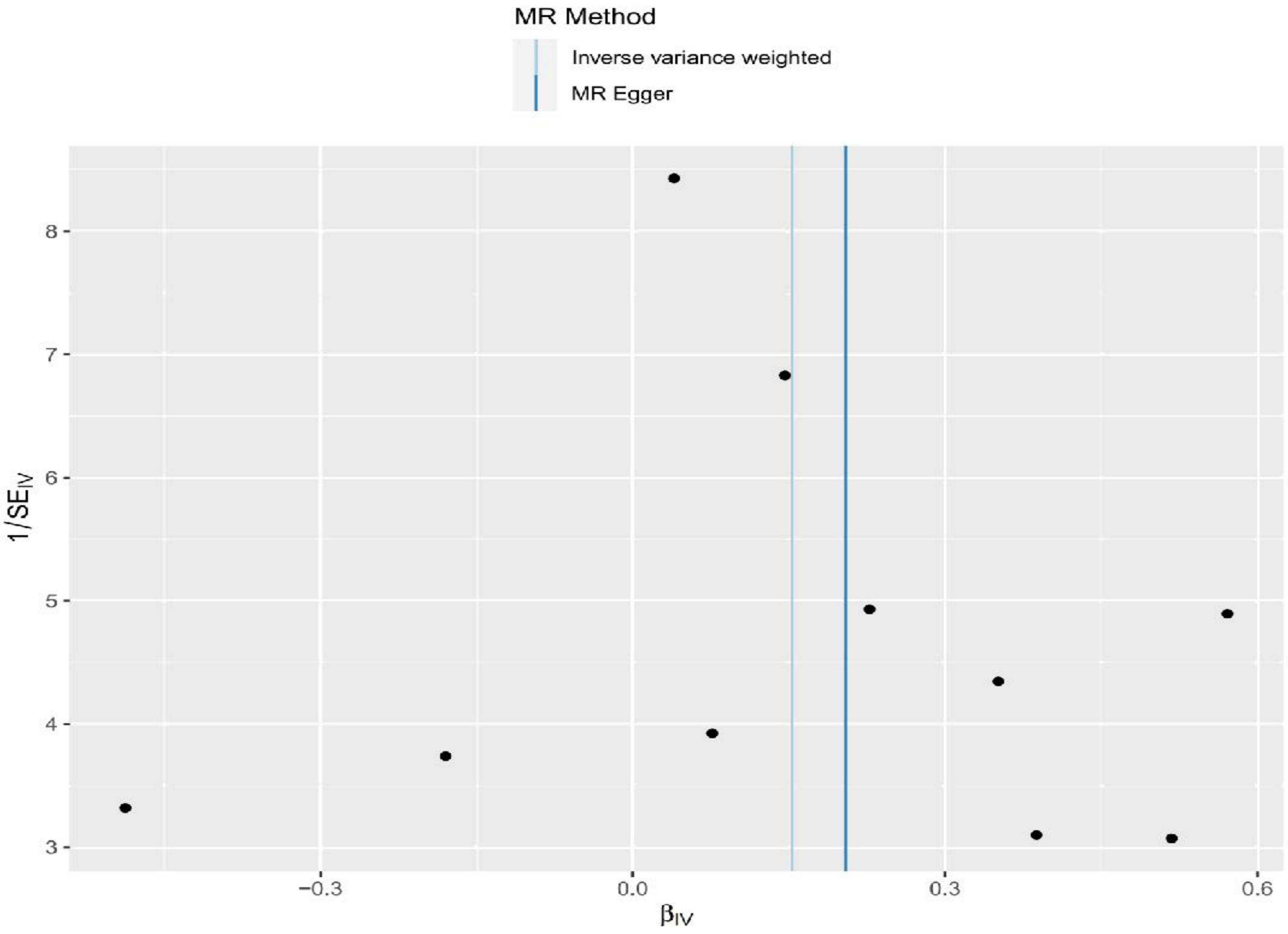

Supplementary Figure-8 Leave-one-out Analysis, Scatter Plot, Forest Plot, and Funnel Plot of All Glioma on Telomere Length

Supplementary Figure-8A Leave-one-out Analysis

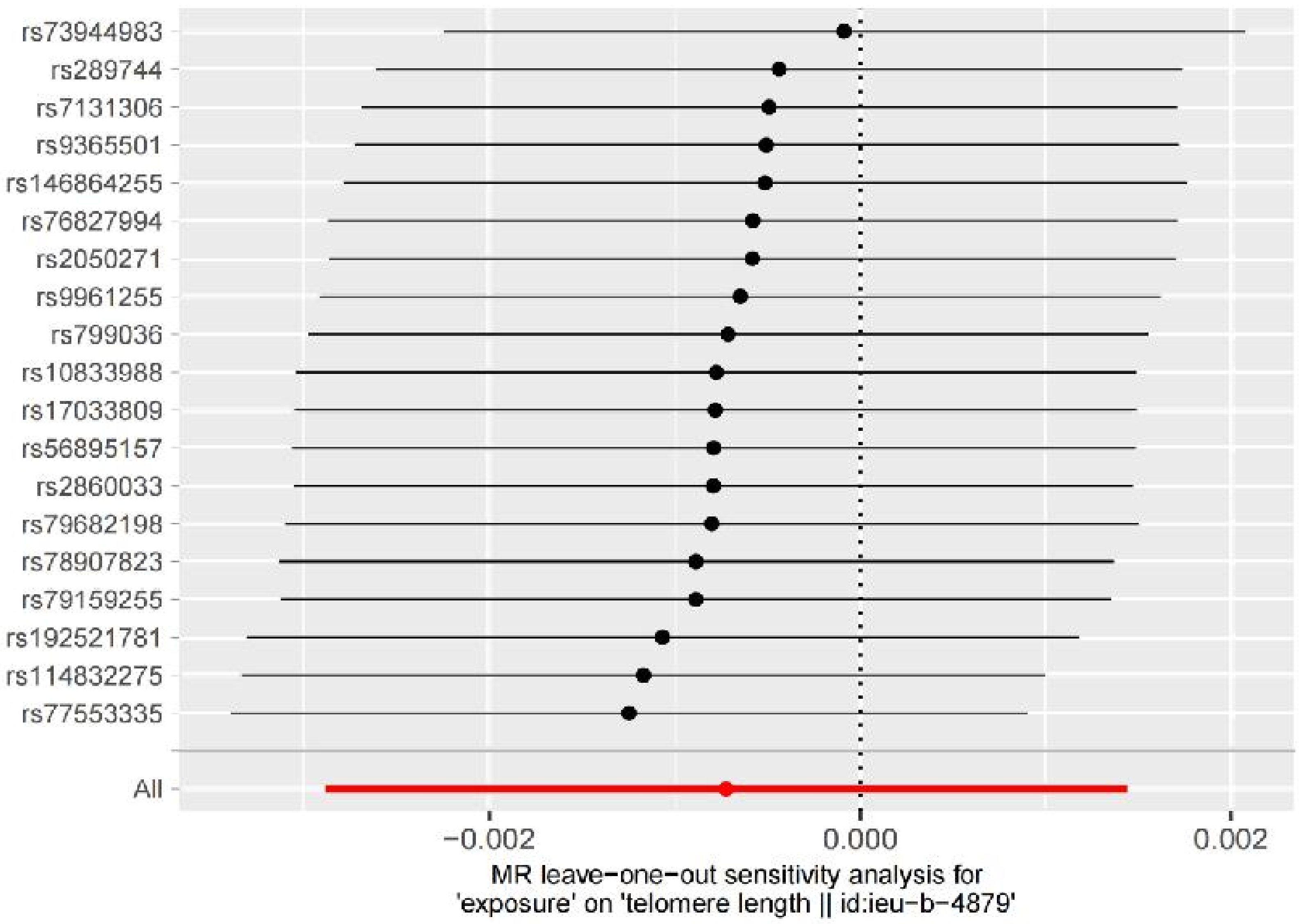

Supplementary Figure-8B Scatter

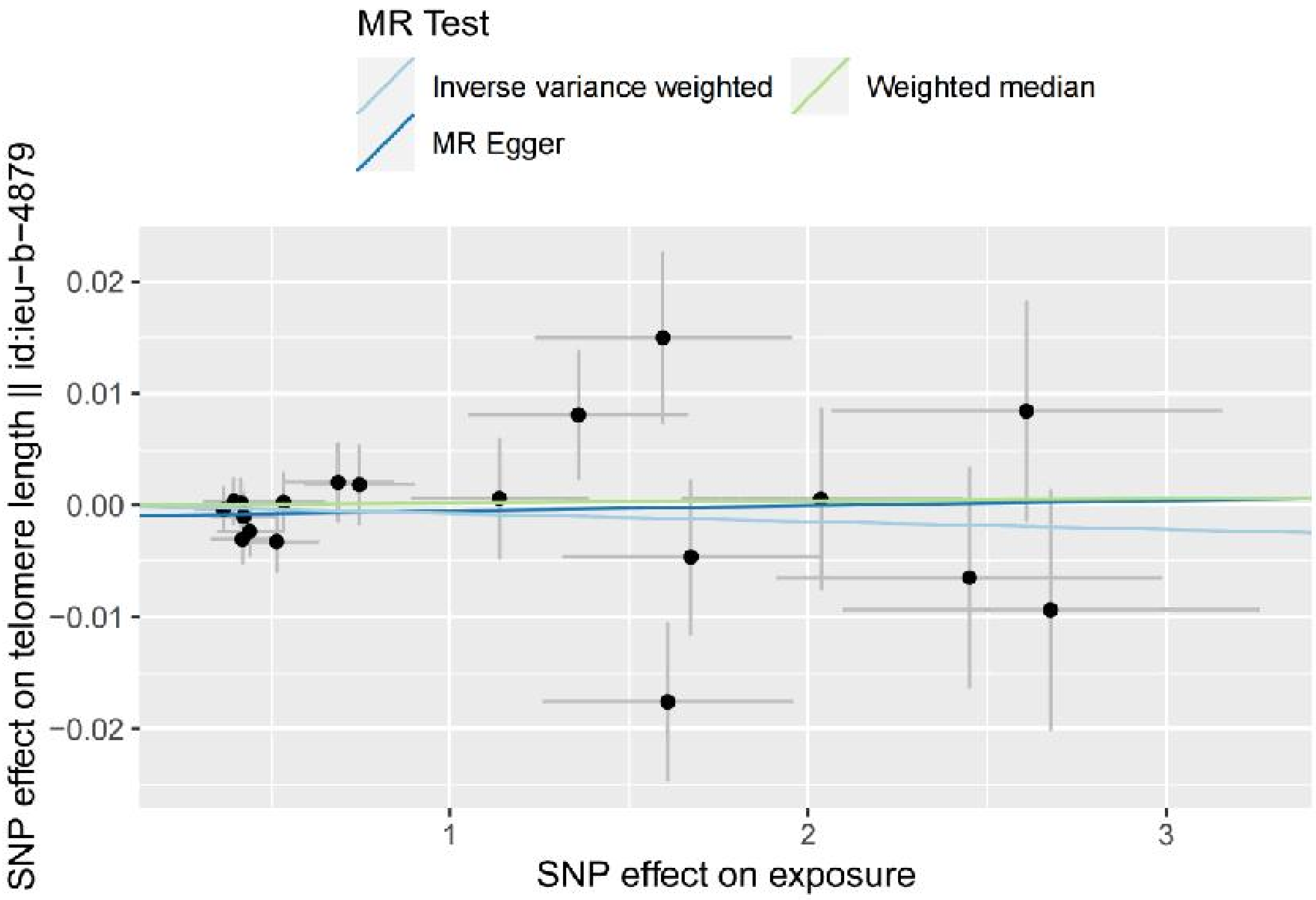

Supplementary Figure-8C Forest Plot

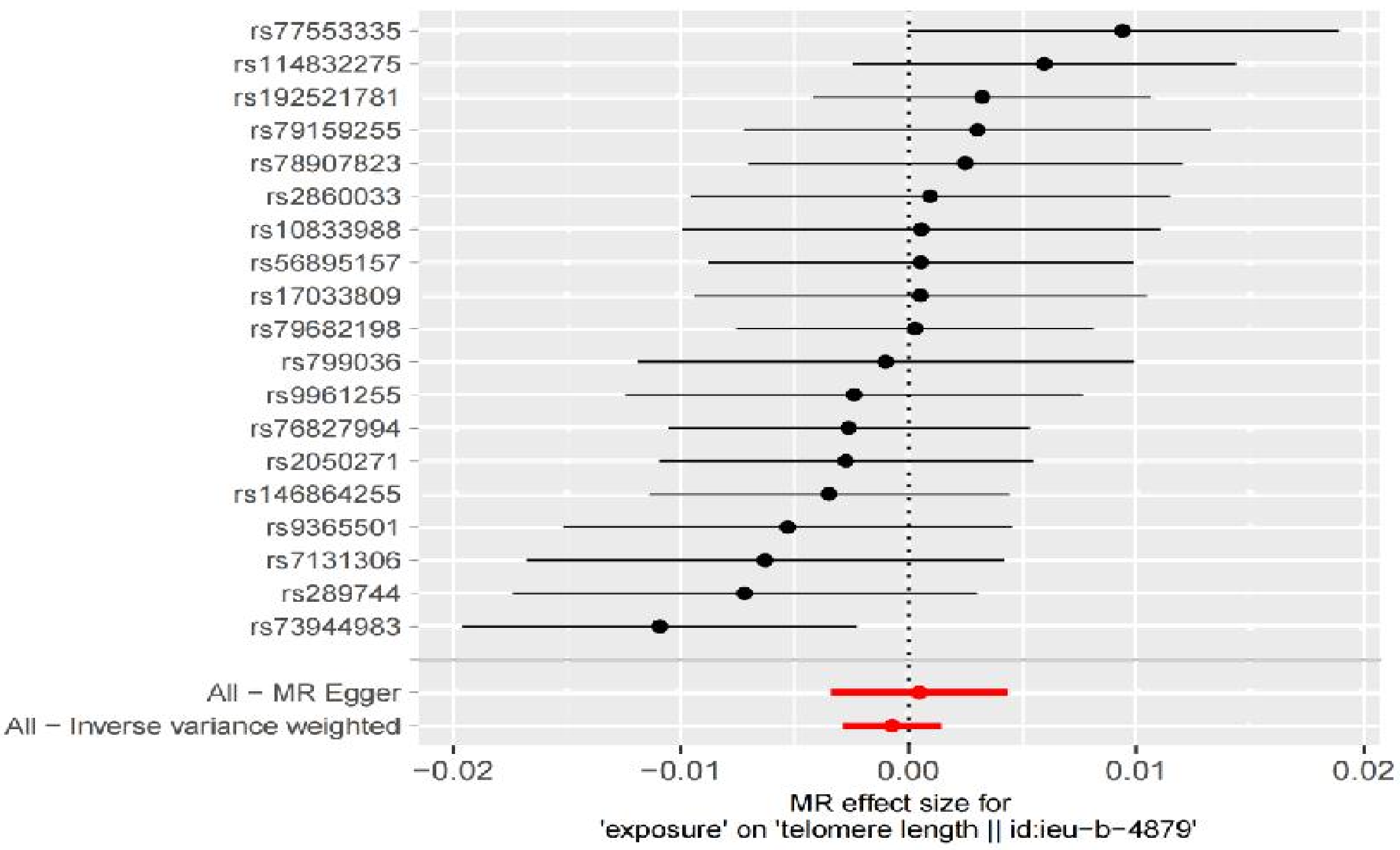

Supplementary Figure-8D Funnel Plot

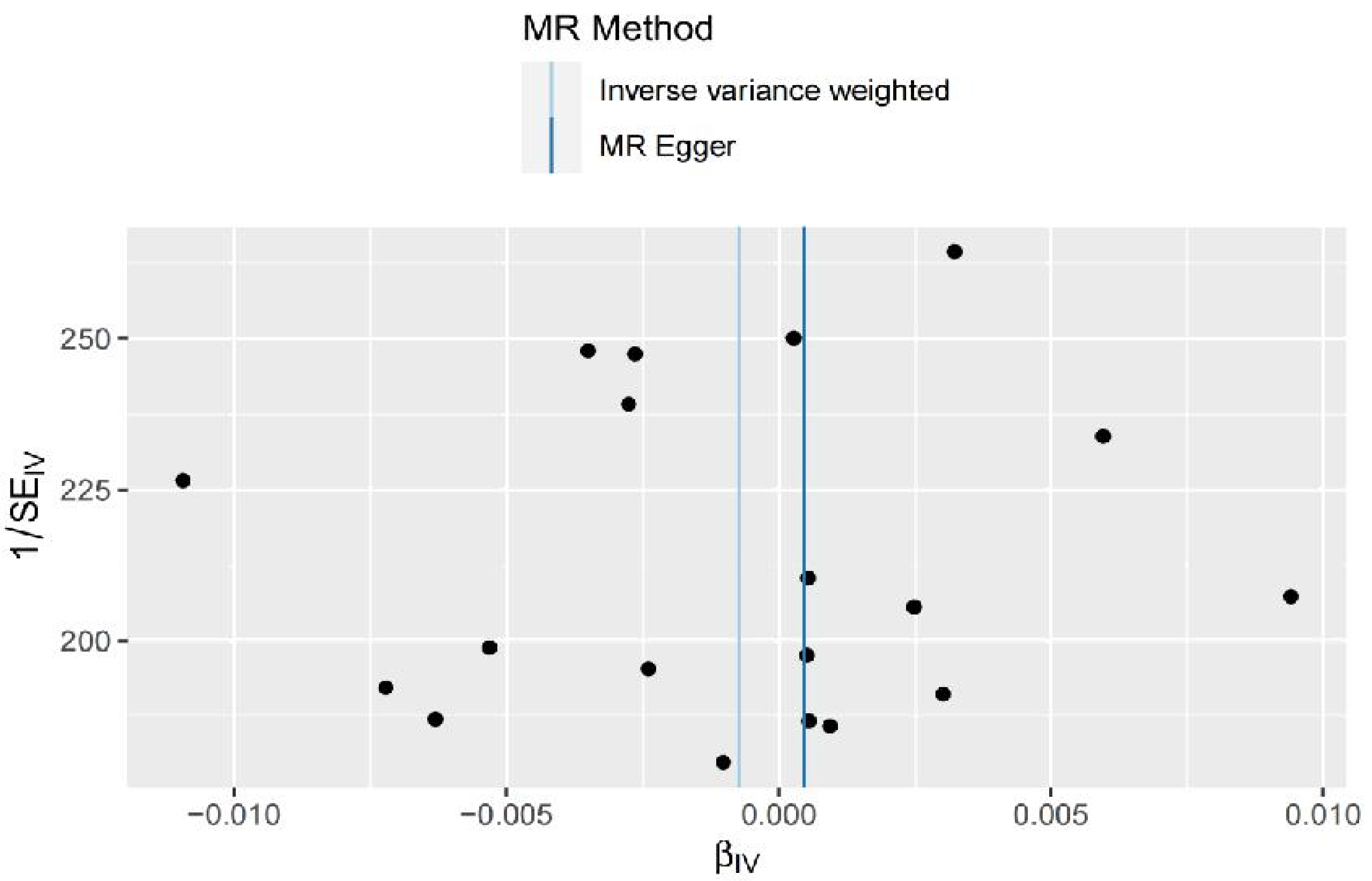

Supplementary Figure-9 Leave-one-out Analysis, Scatter Plot, Forest Plot, and Funnel Plot of Amyotrophic Lateral Sclerosis on Facial Aging

Supplementary Figure-9A Leave-one-out Analysis

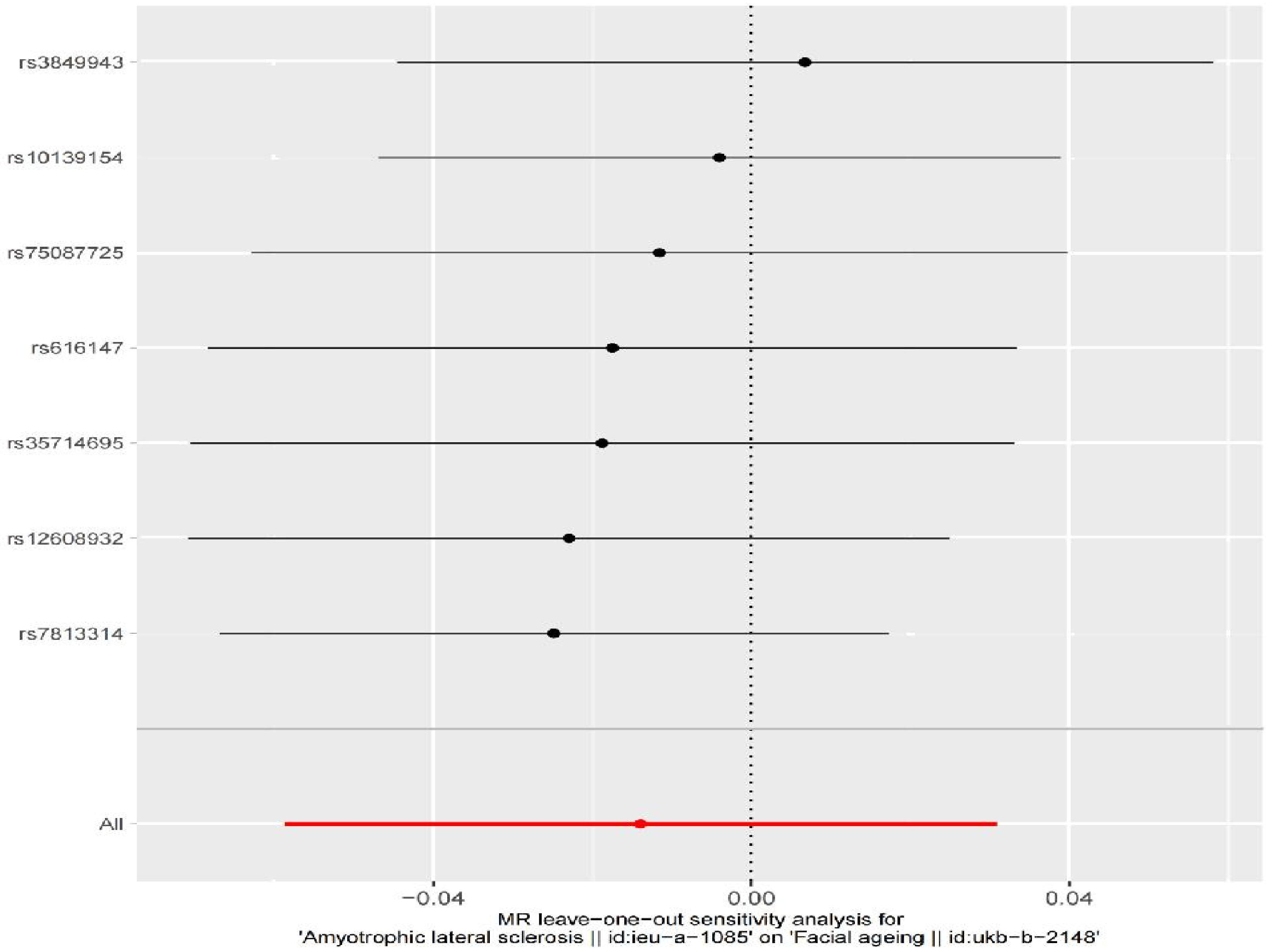

Supplementary Figure-9B Scatter

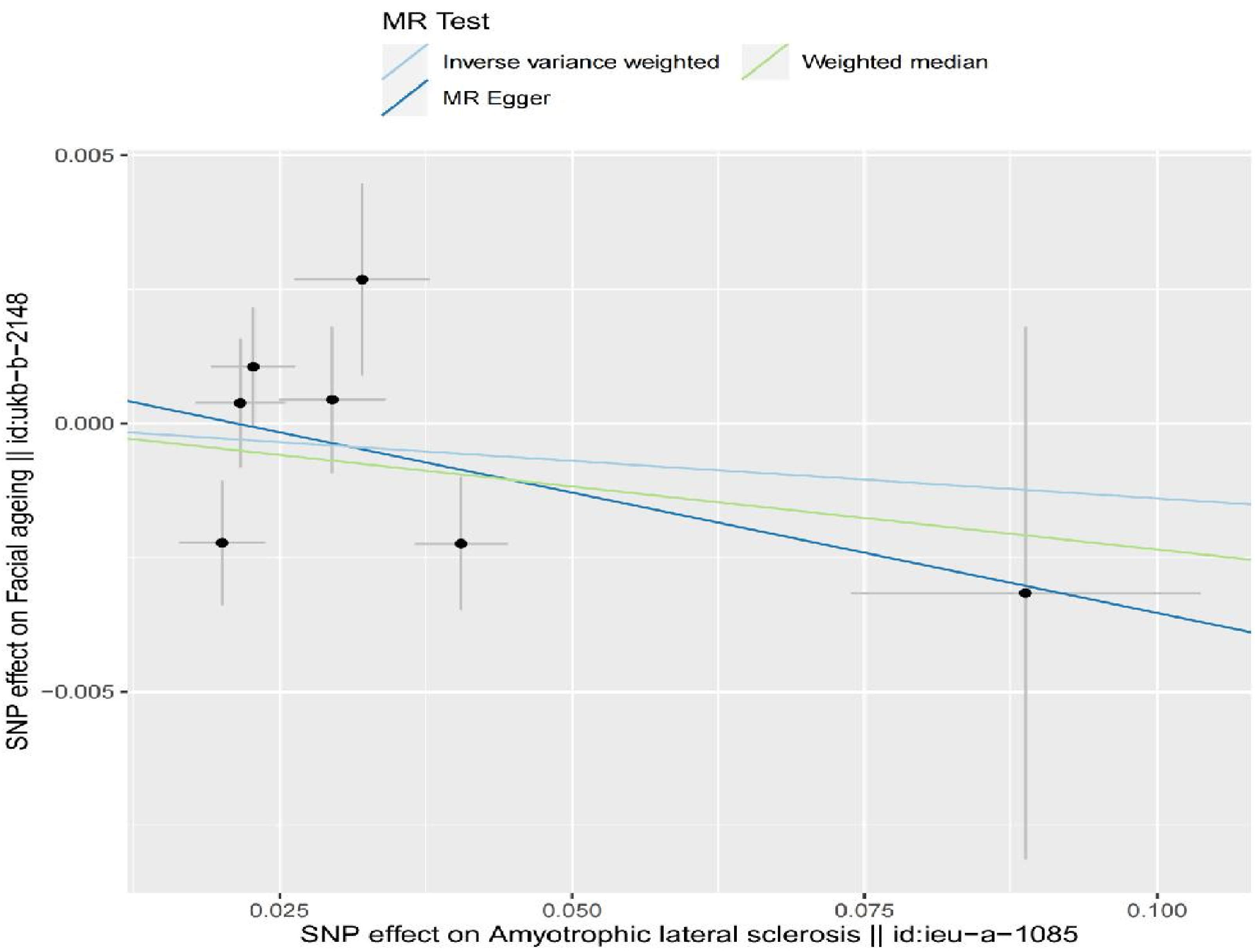

Supplementary Figure-9D Forest Plot

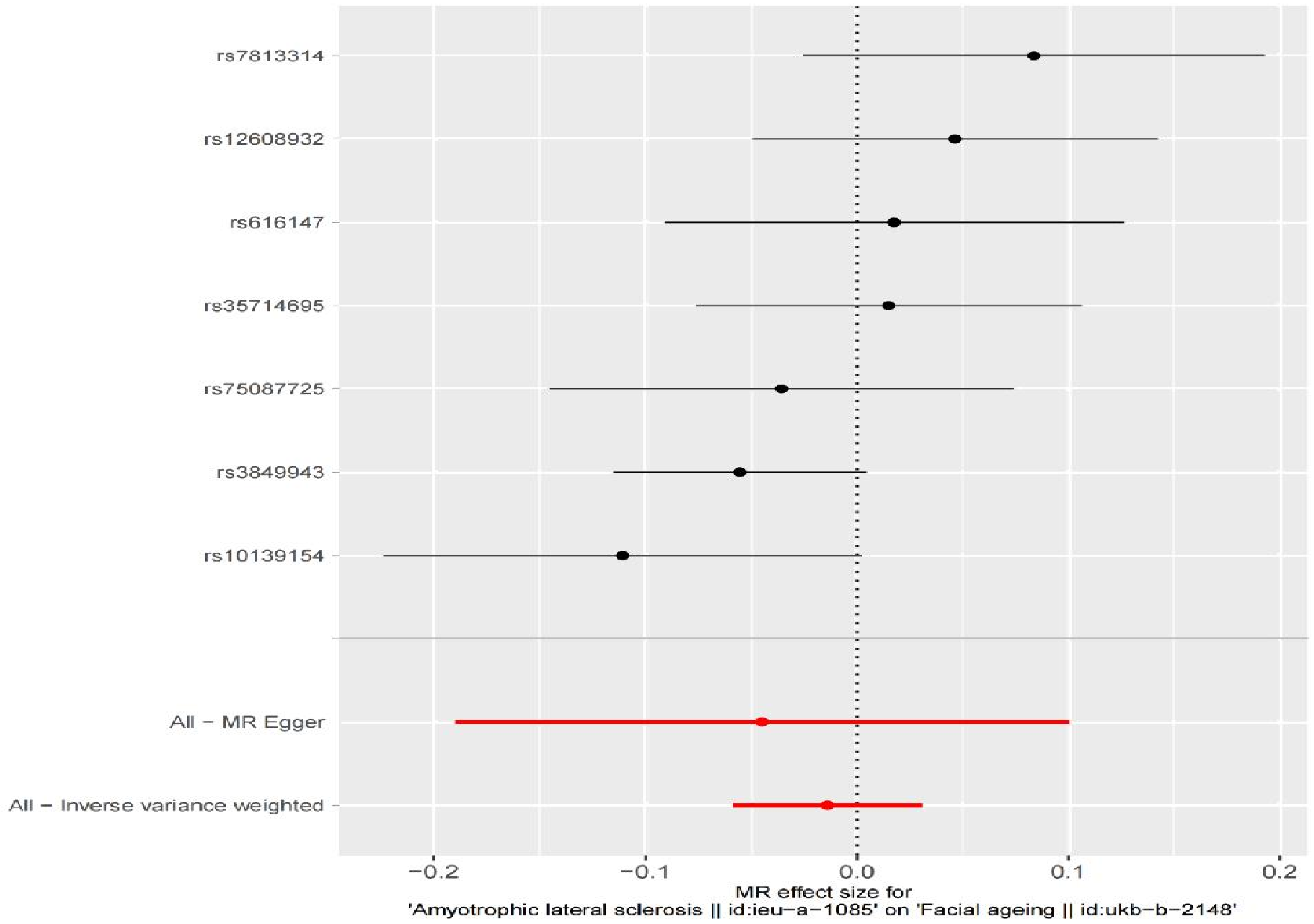

Supplementary Figure-9D Funnel Plot

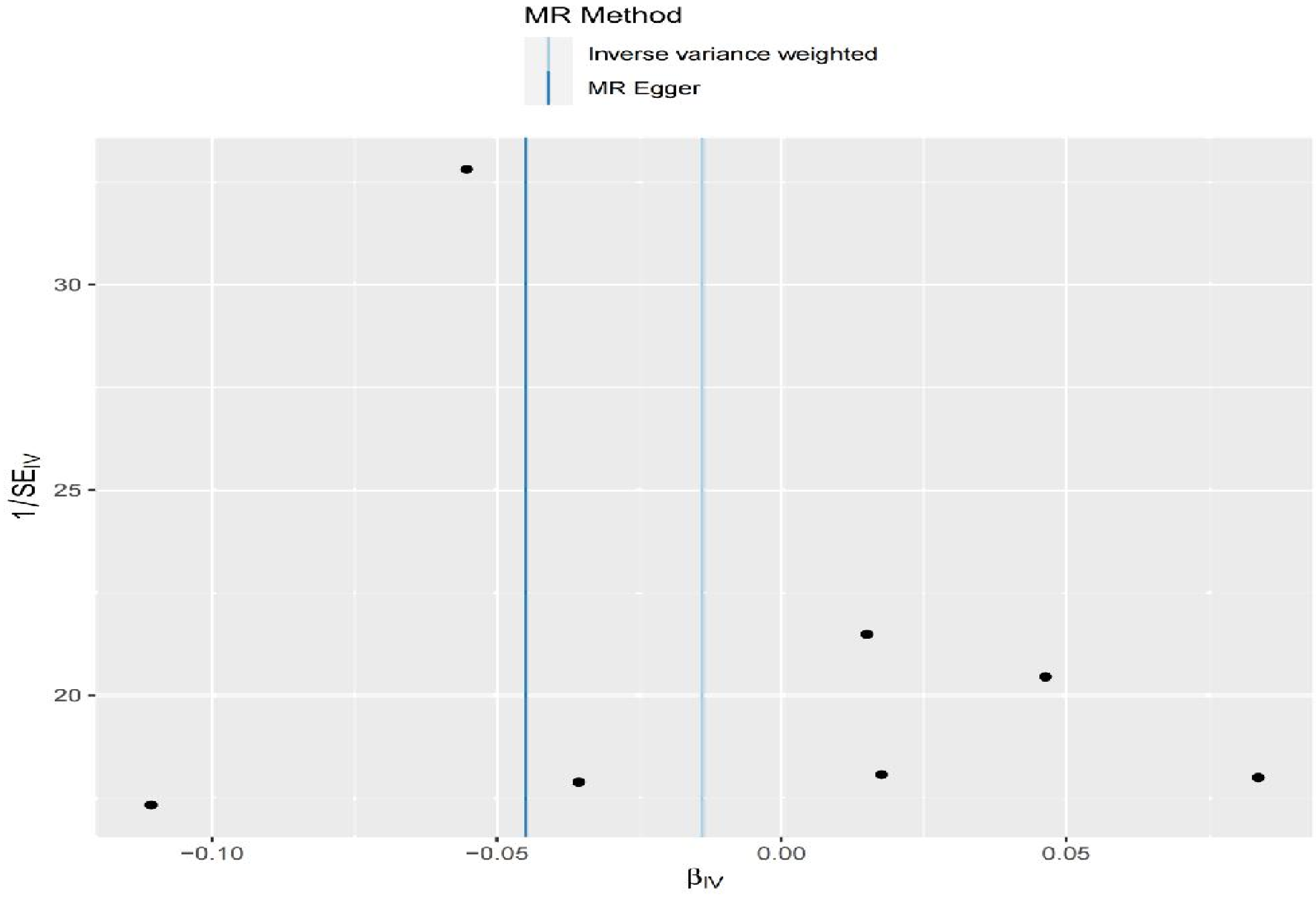

Supplementary Figure10 Leave-one-out Analysis, Scatter Plot, Forest Plot, and Funnel Plot of Amyotrophic Lateral Sclerosis on Frailty Index

Supplementary Figure-10A Leave-one-out Analysis

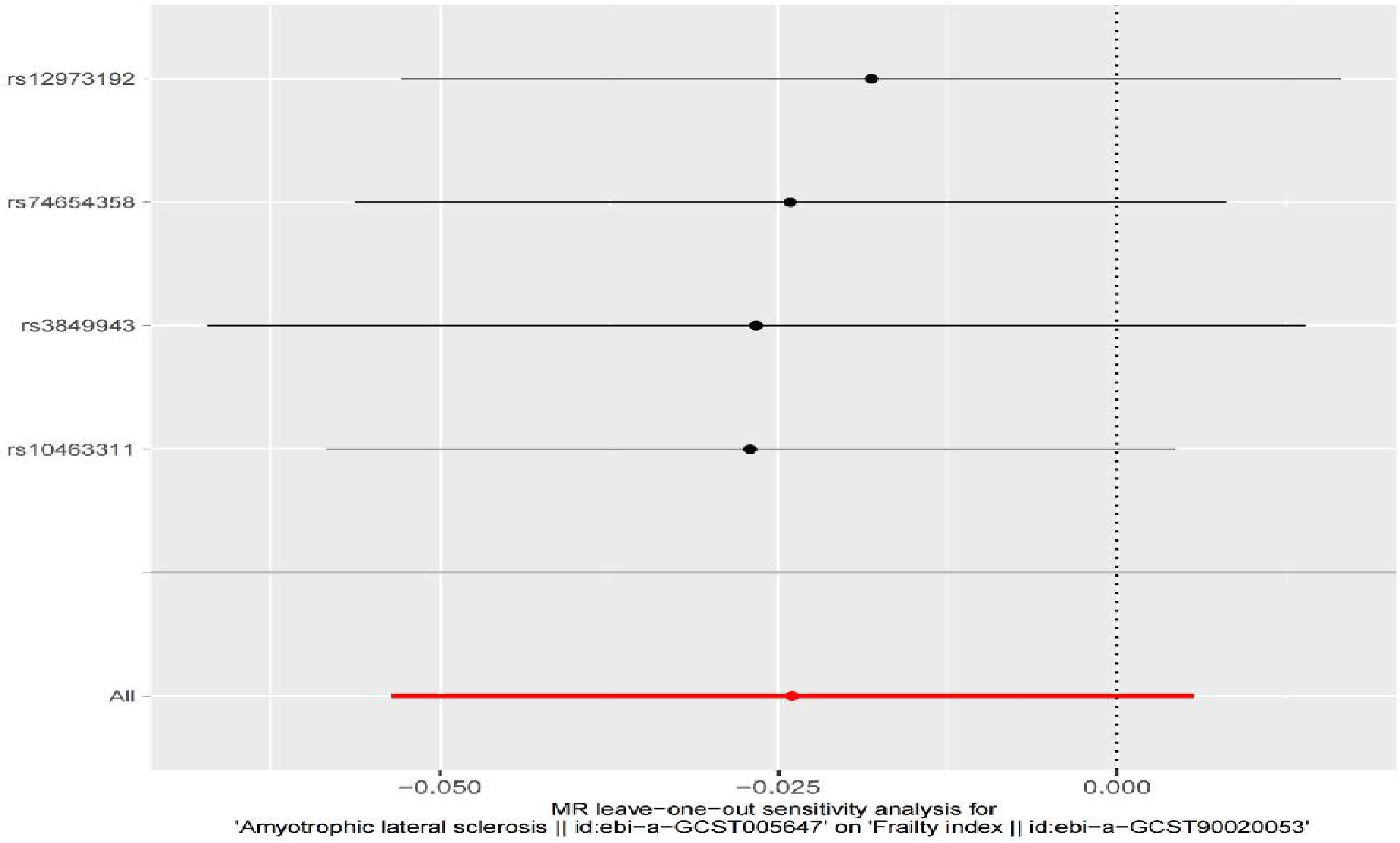

Supplementary Figure-10B Scatter

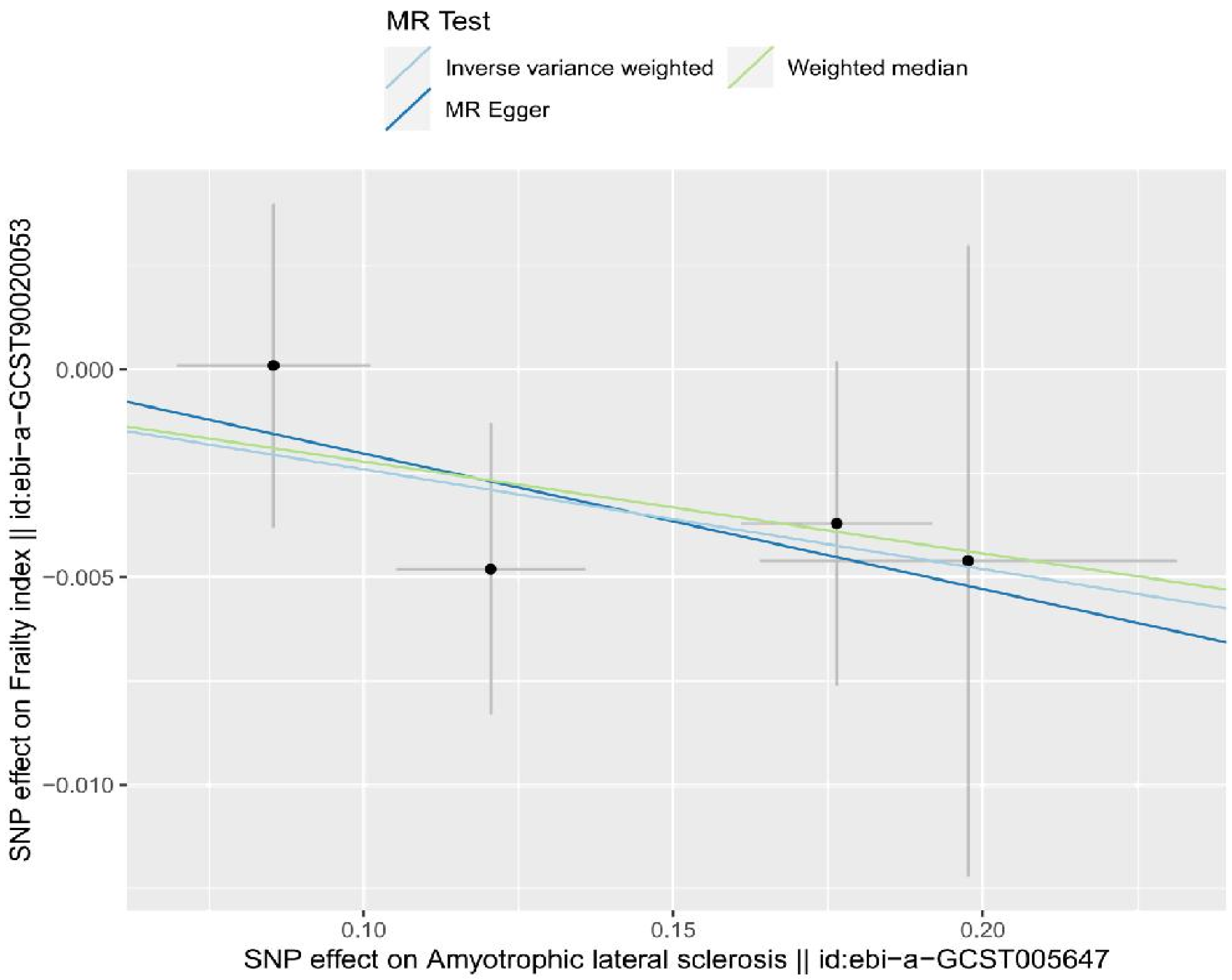

Supplementary Figure-10C Forest Plot

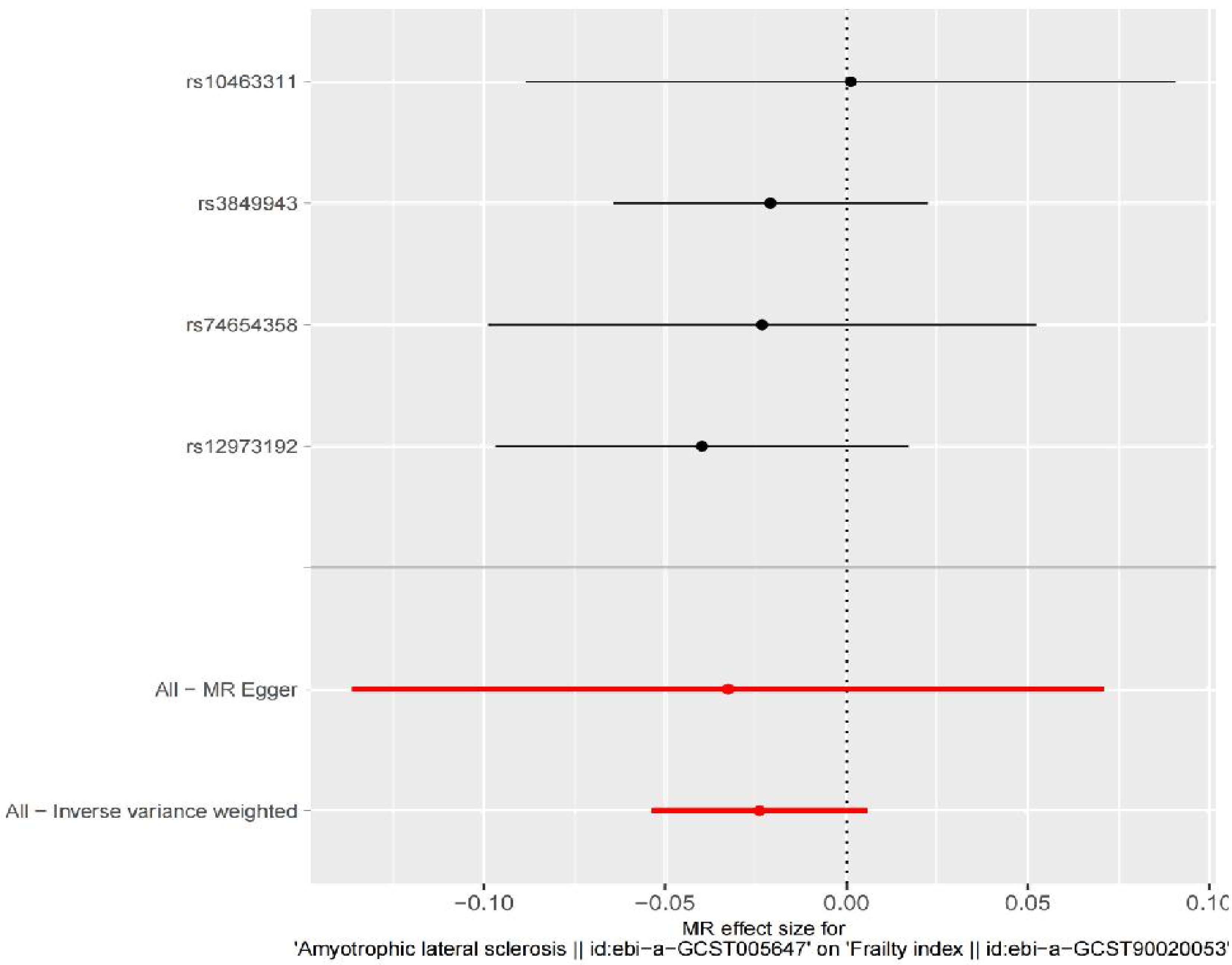

Supplementary Figure-10D Funnel Plot

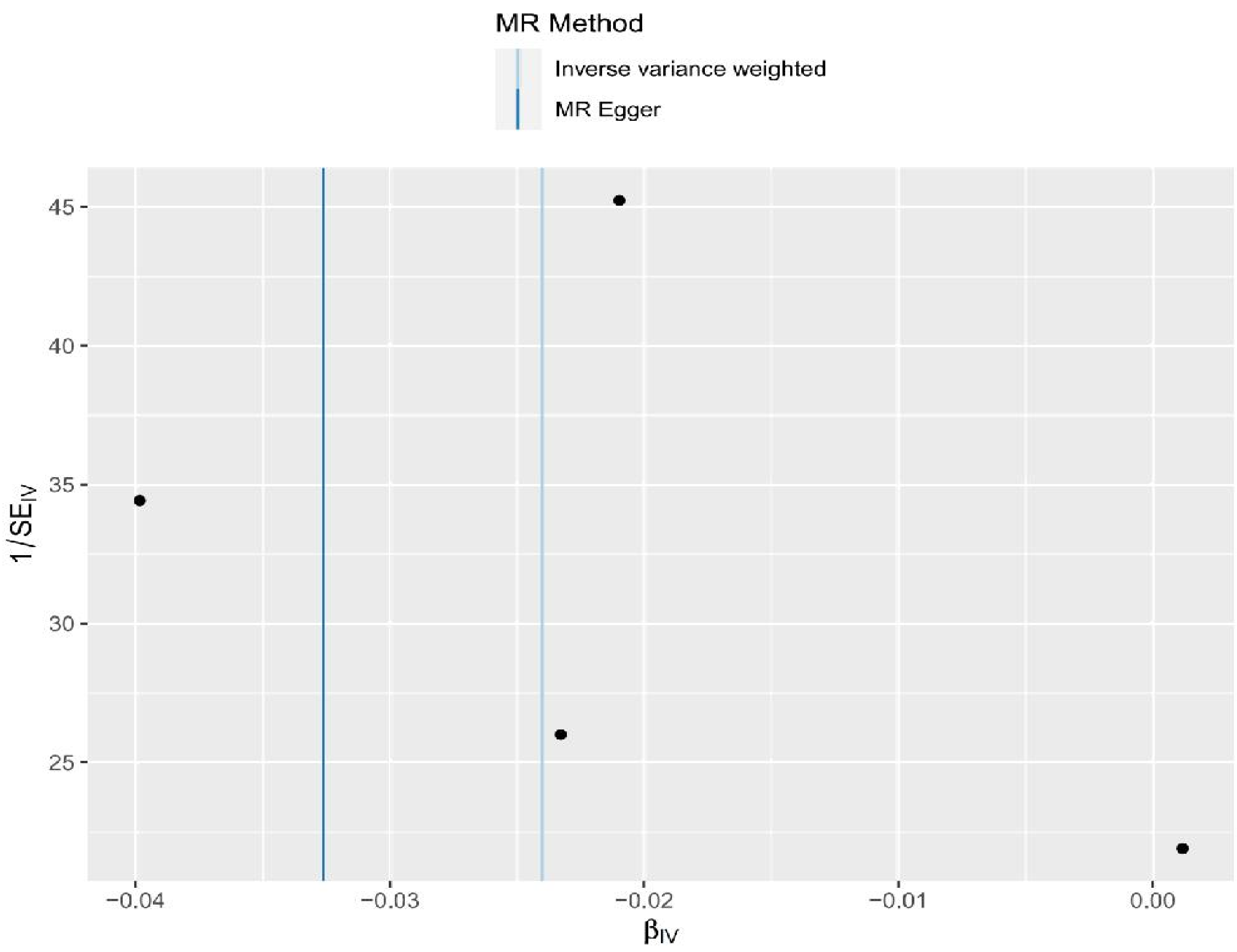

Supplementary Figure-11 Scatter Plot, Forest Plot, and Funnel Plot of Amyotrophic Lateral Sclerosis on DNA methylation GrimAge Acceleration

Supplementary Figure-11A Scatter

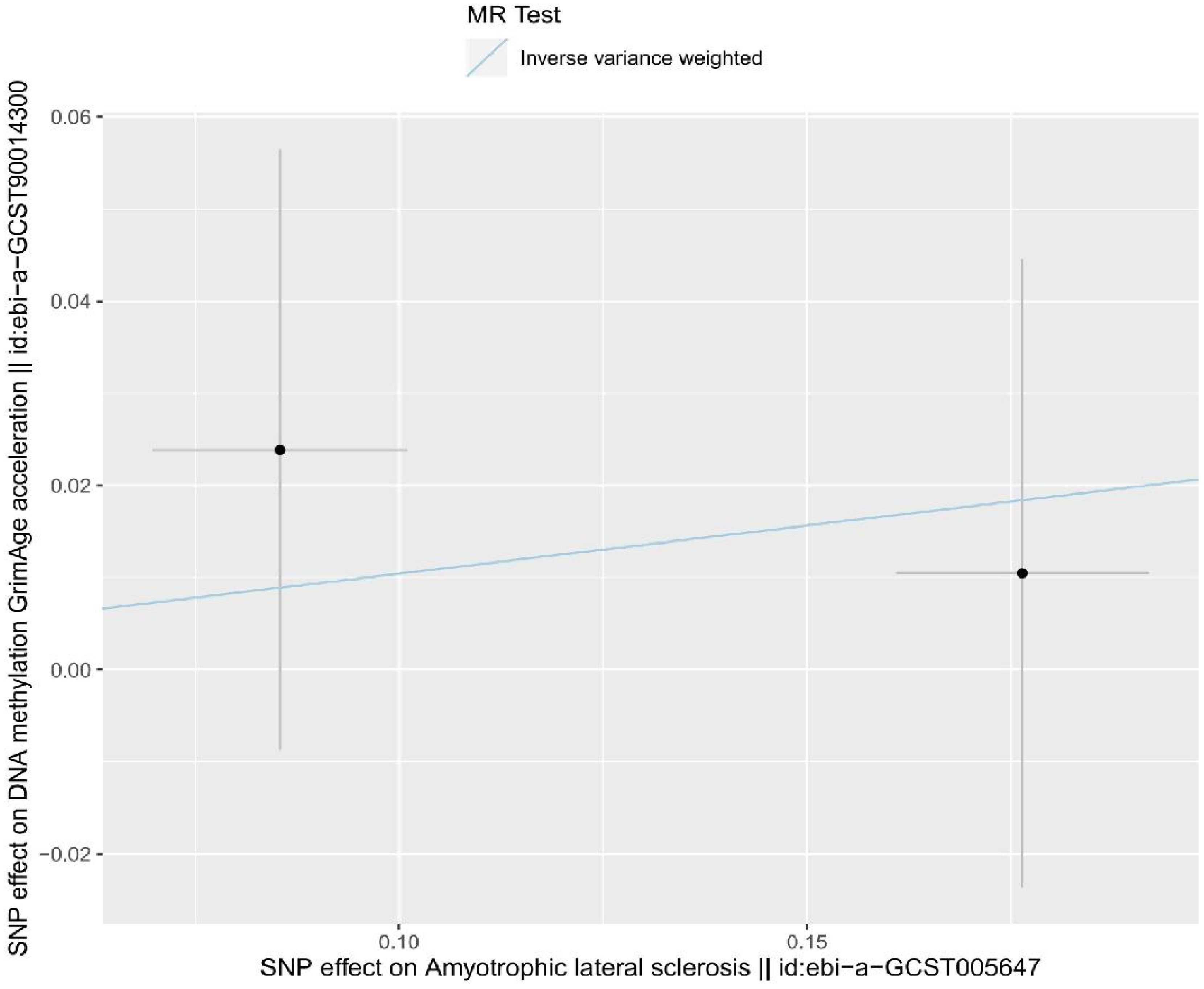

Supplementary Figure-11B Forest Plot

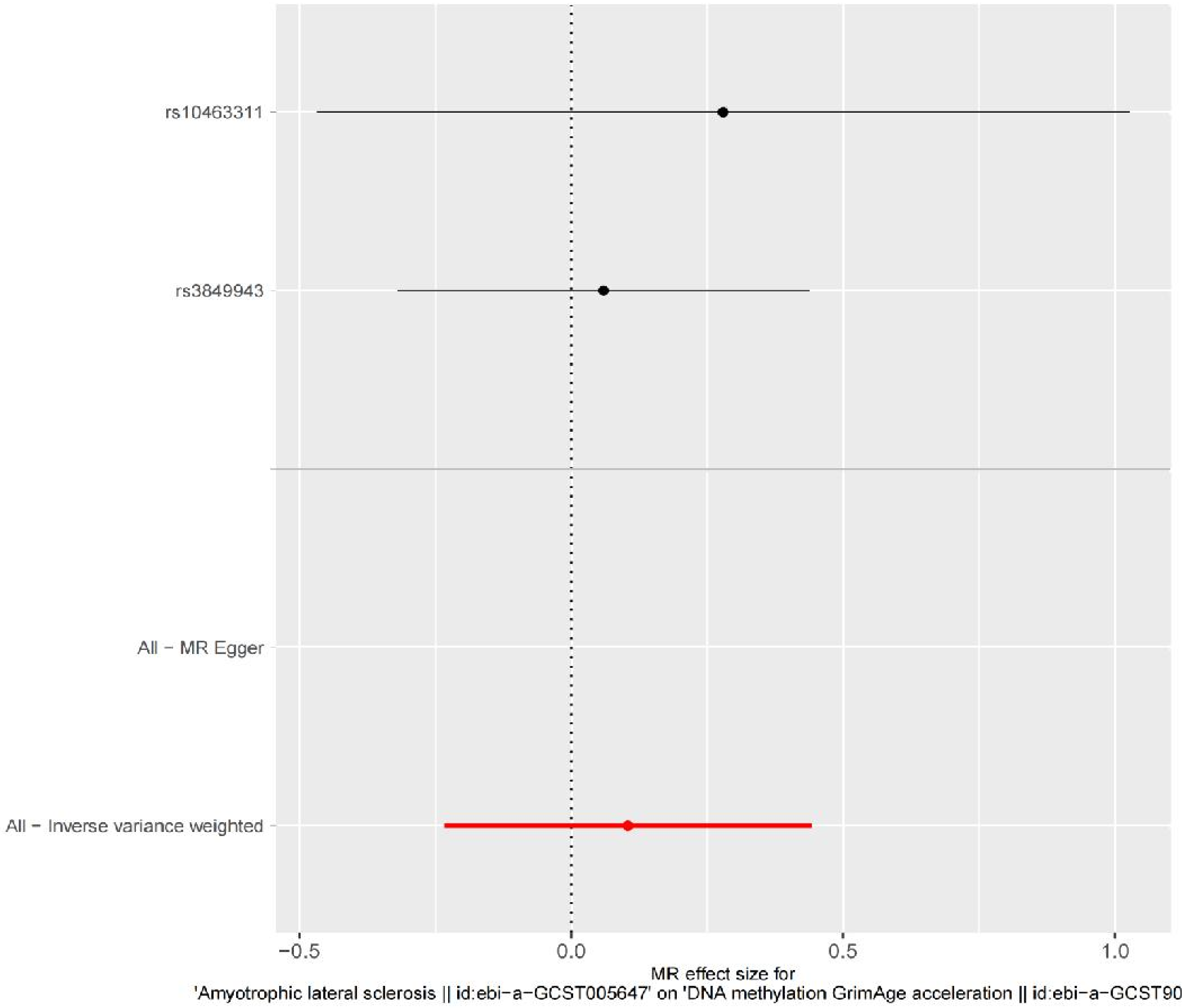

Supplementary Figure-11C Funnel Plot

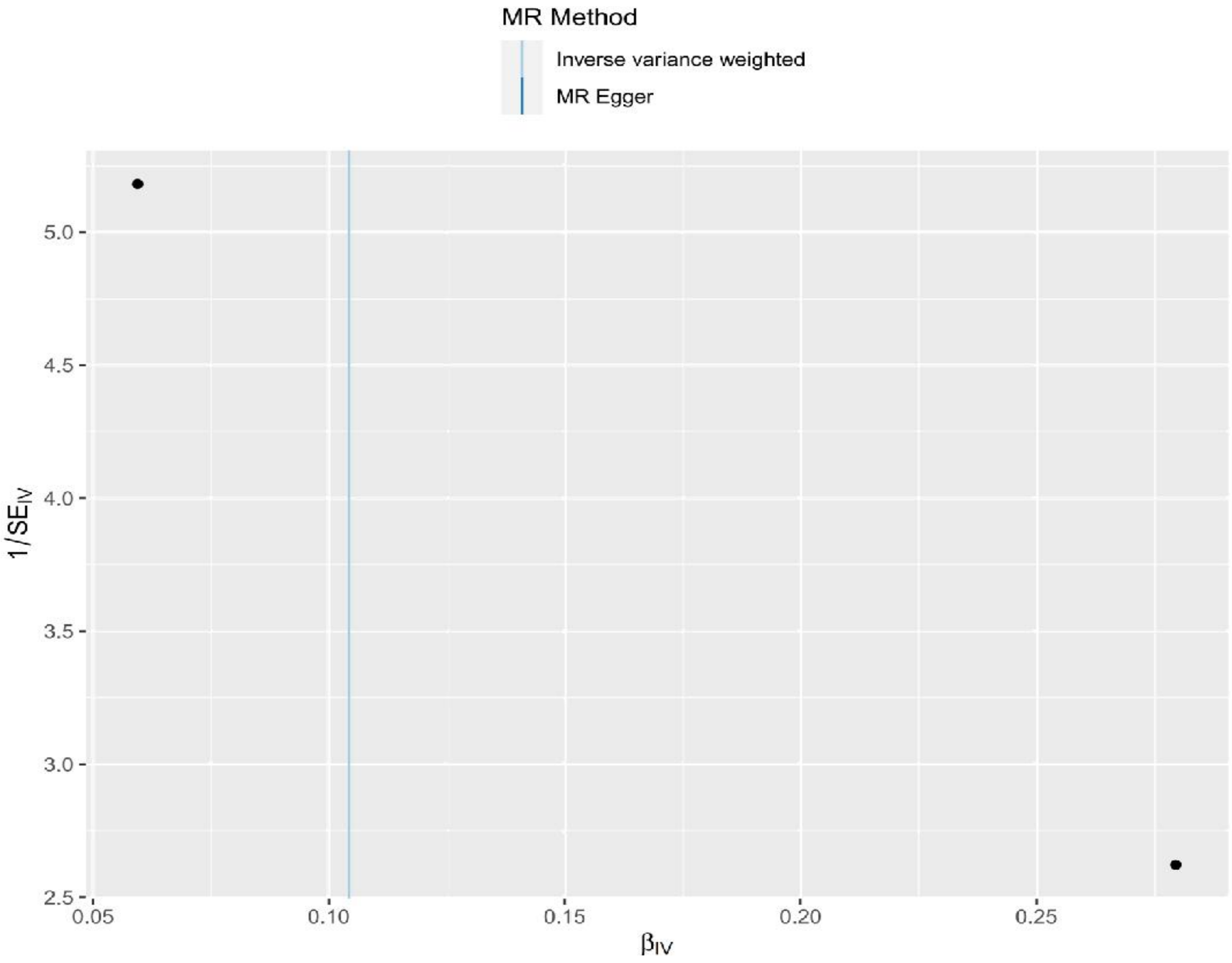

Supplementary Figure-12 Leave-one-out Analysis, Scatter Plot, Forest Plot, and Funnel Plot of Amyotrophic Lateral Sclerosis on Telomere Length

Supplementary Figure-8A Leave-one-out Analysis

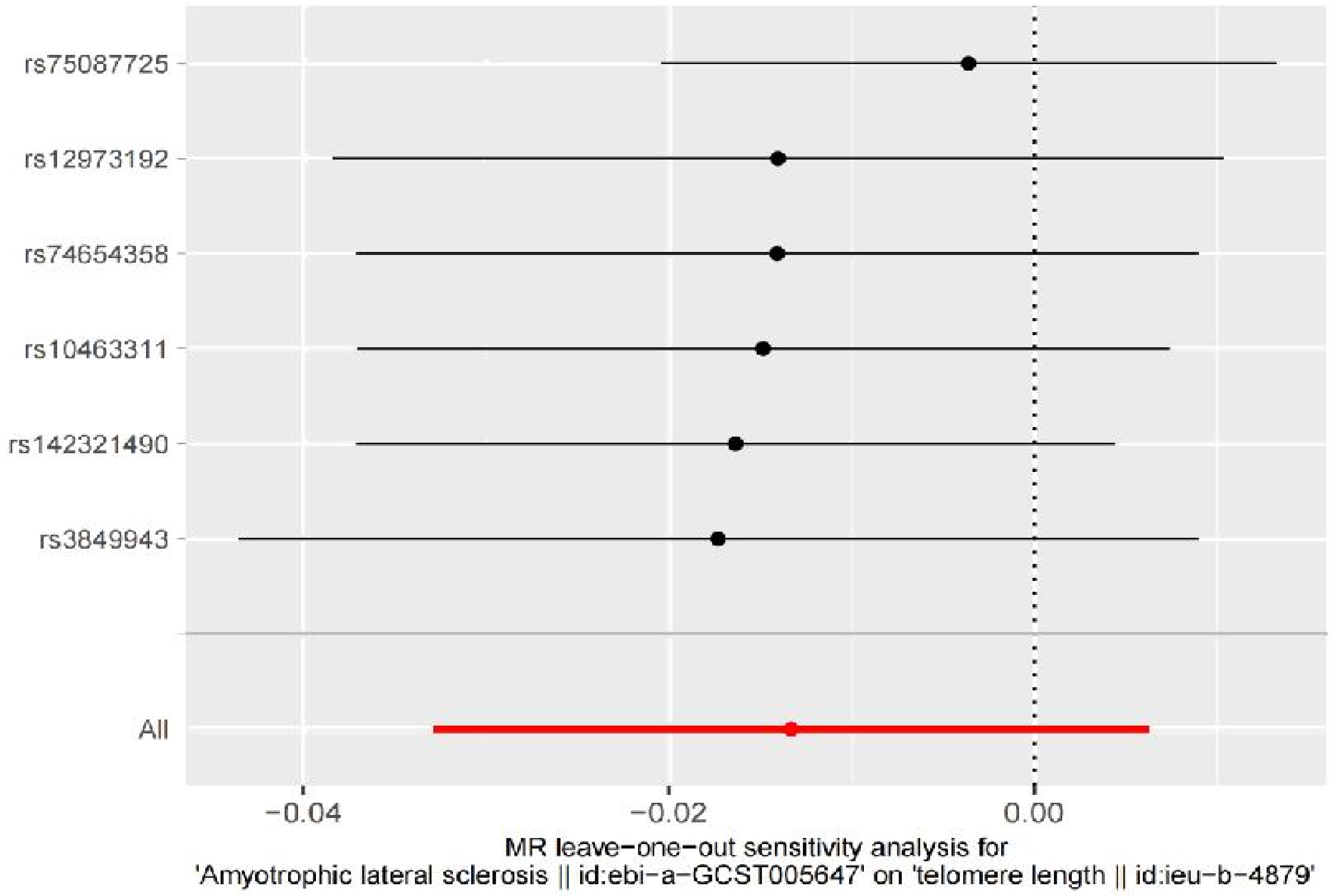

Supplementary Figure-12B Scatter

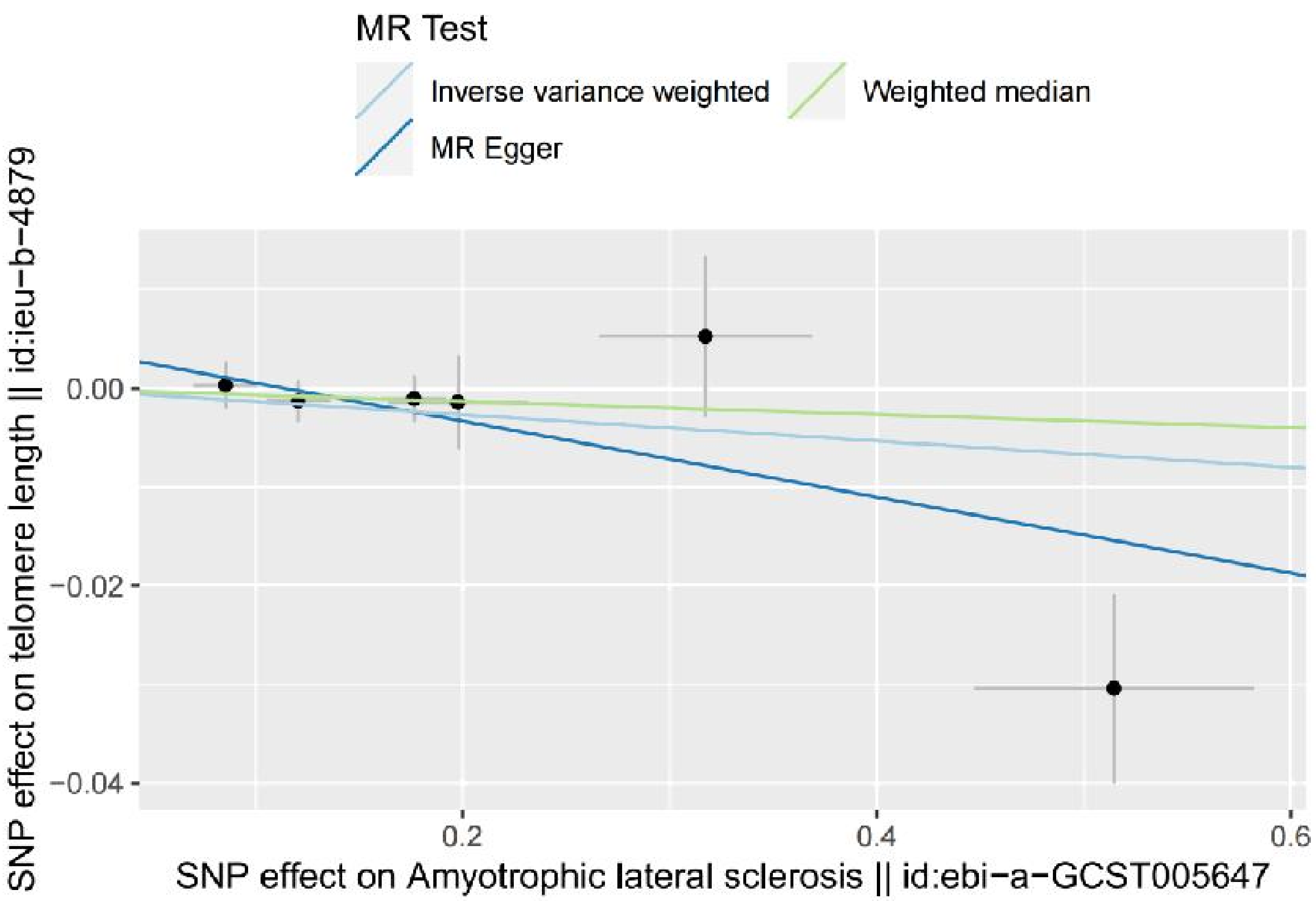

Supplementary Figure-12C Forest Plot

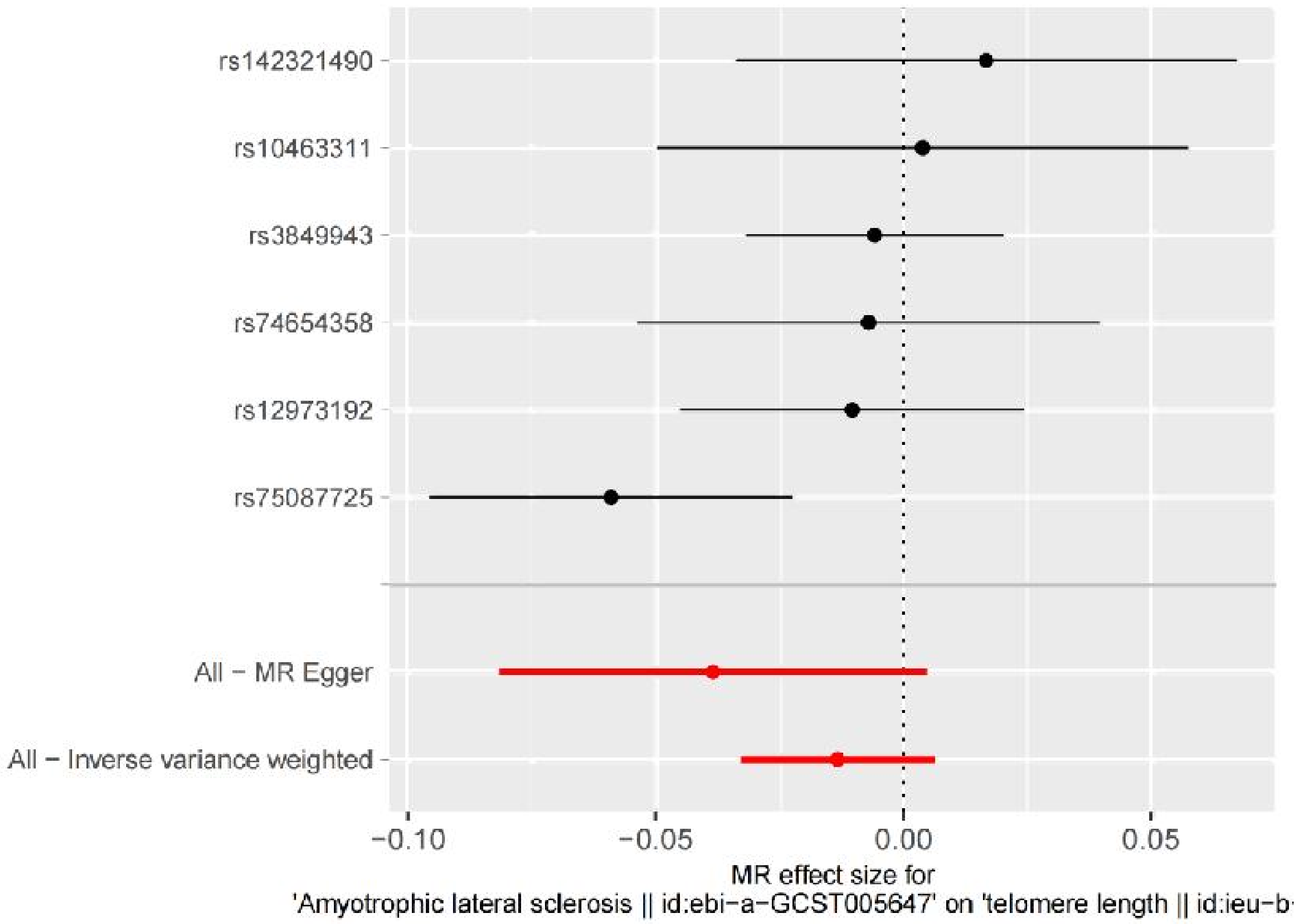

Supplementary Figure-12D Funnel Plot

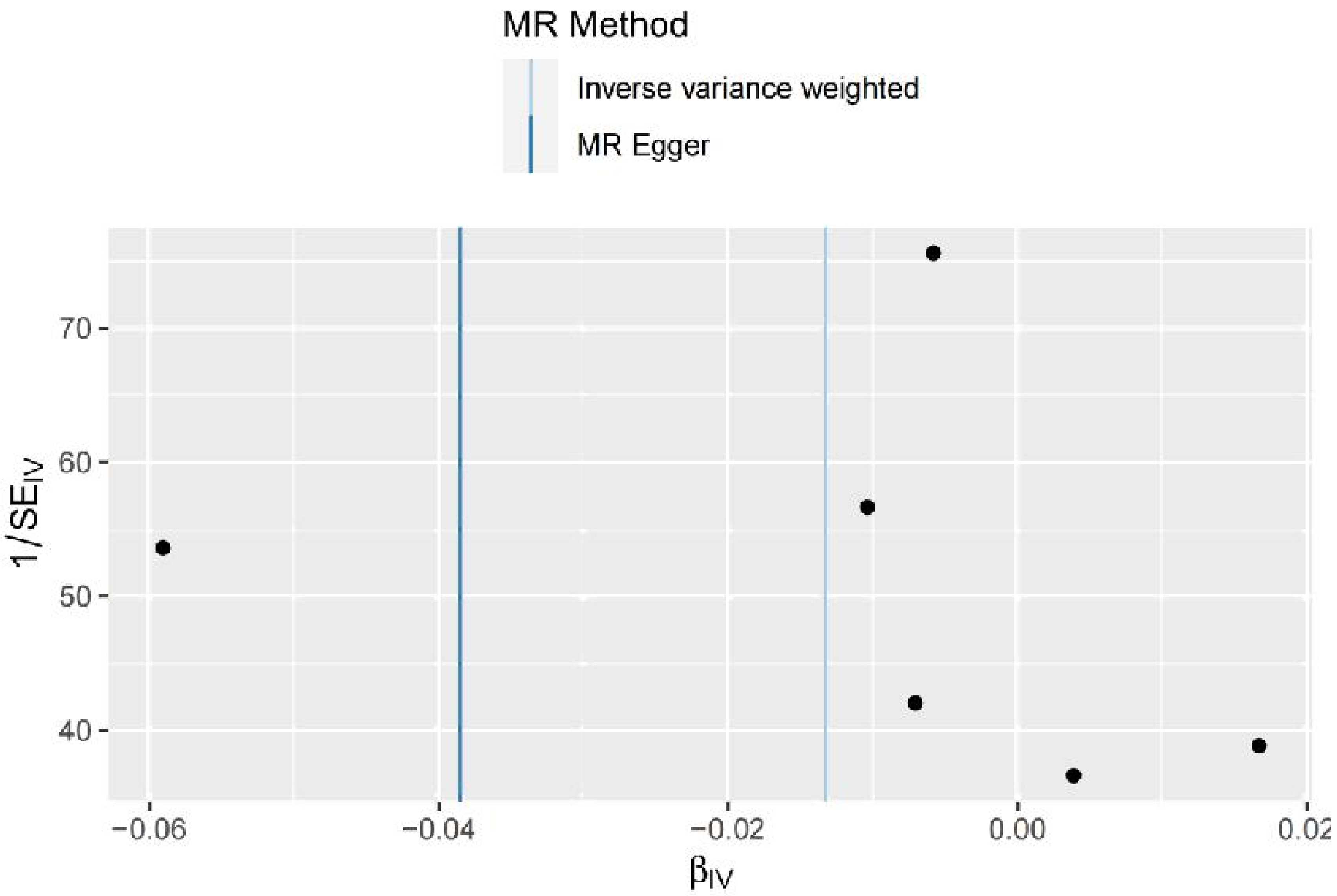

Supplementary Figure-13 Leave-one-out Analysis, Scatter Plot, Forest Plot, and Funnel Plot of Facial Aging on Alzheimer's Disease

Supplementary Figure-13A Leave-one-out Analysis

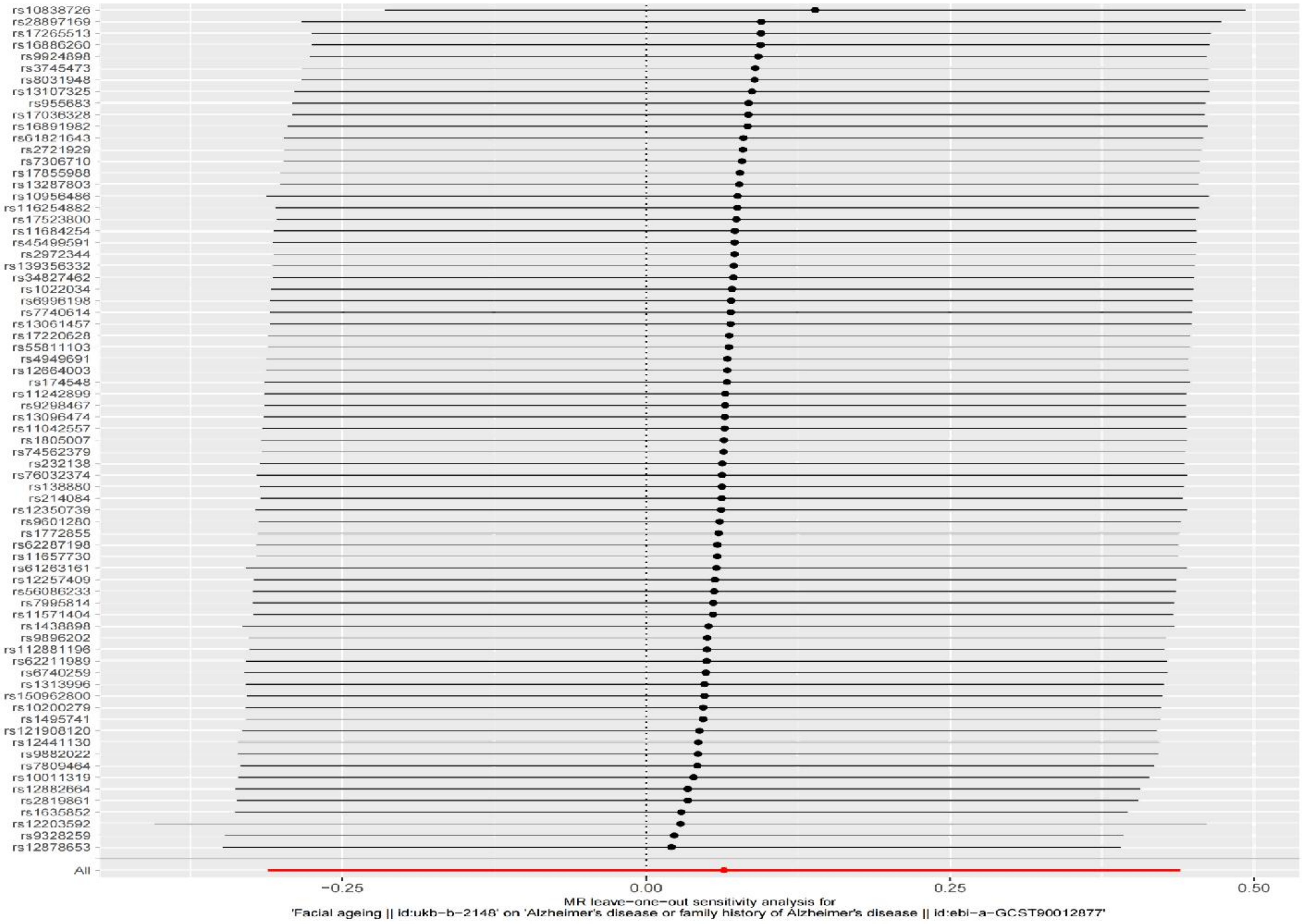

Supplementary Figure-13B Scatter

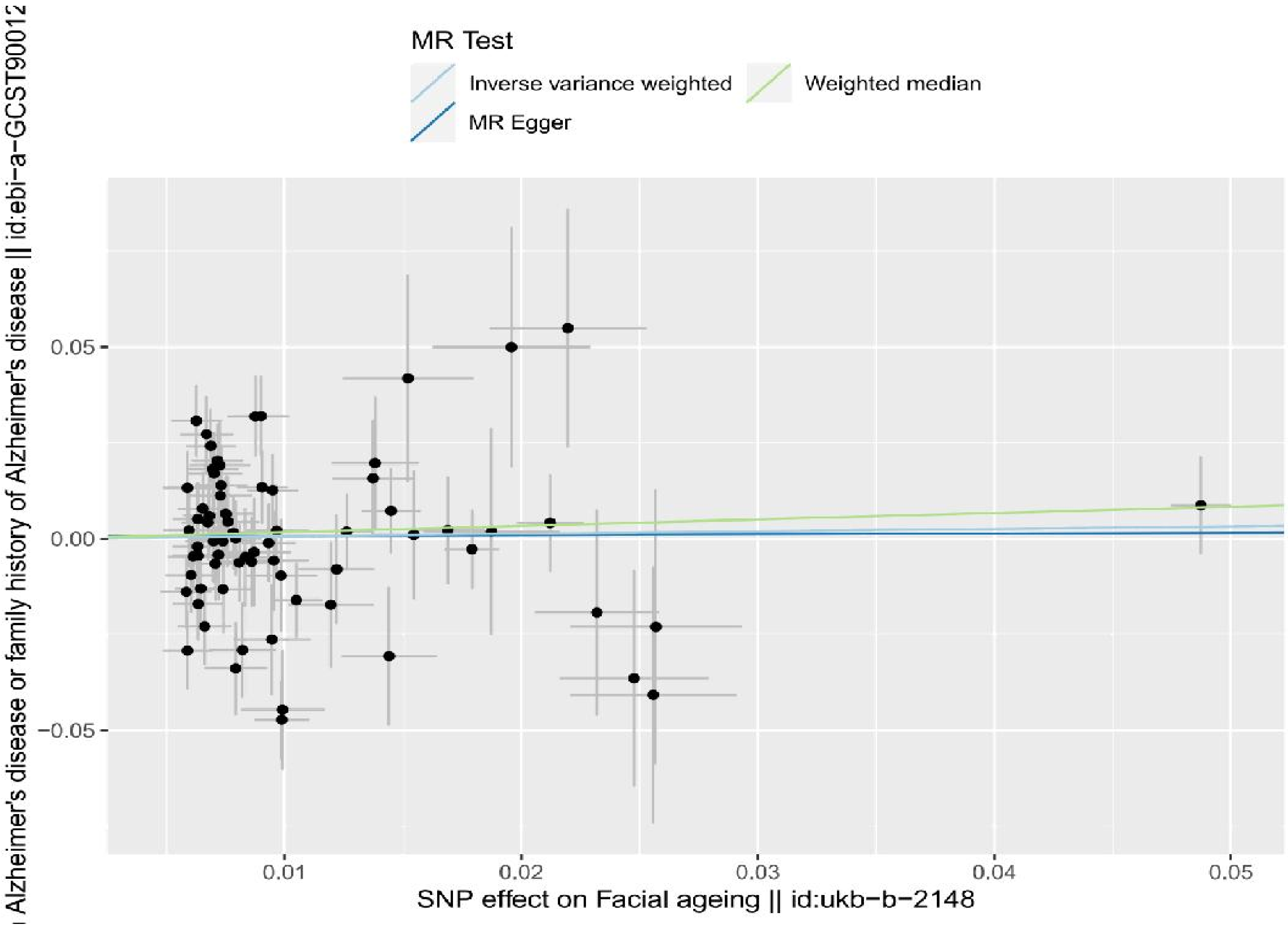

Supplementary Figure-13C Forest Plot

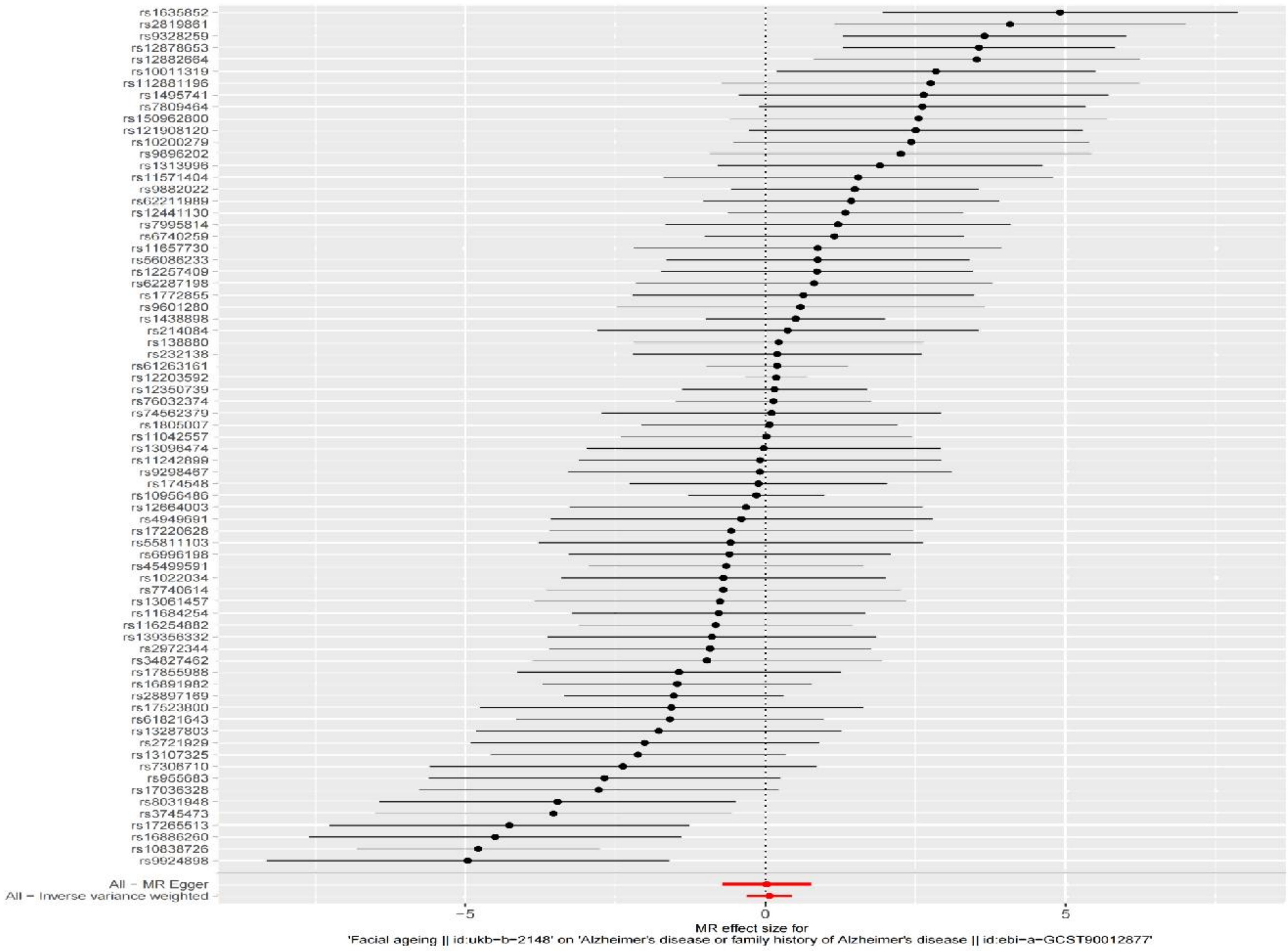

Supplementary Figure-13D Funnel Plot

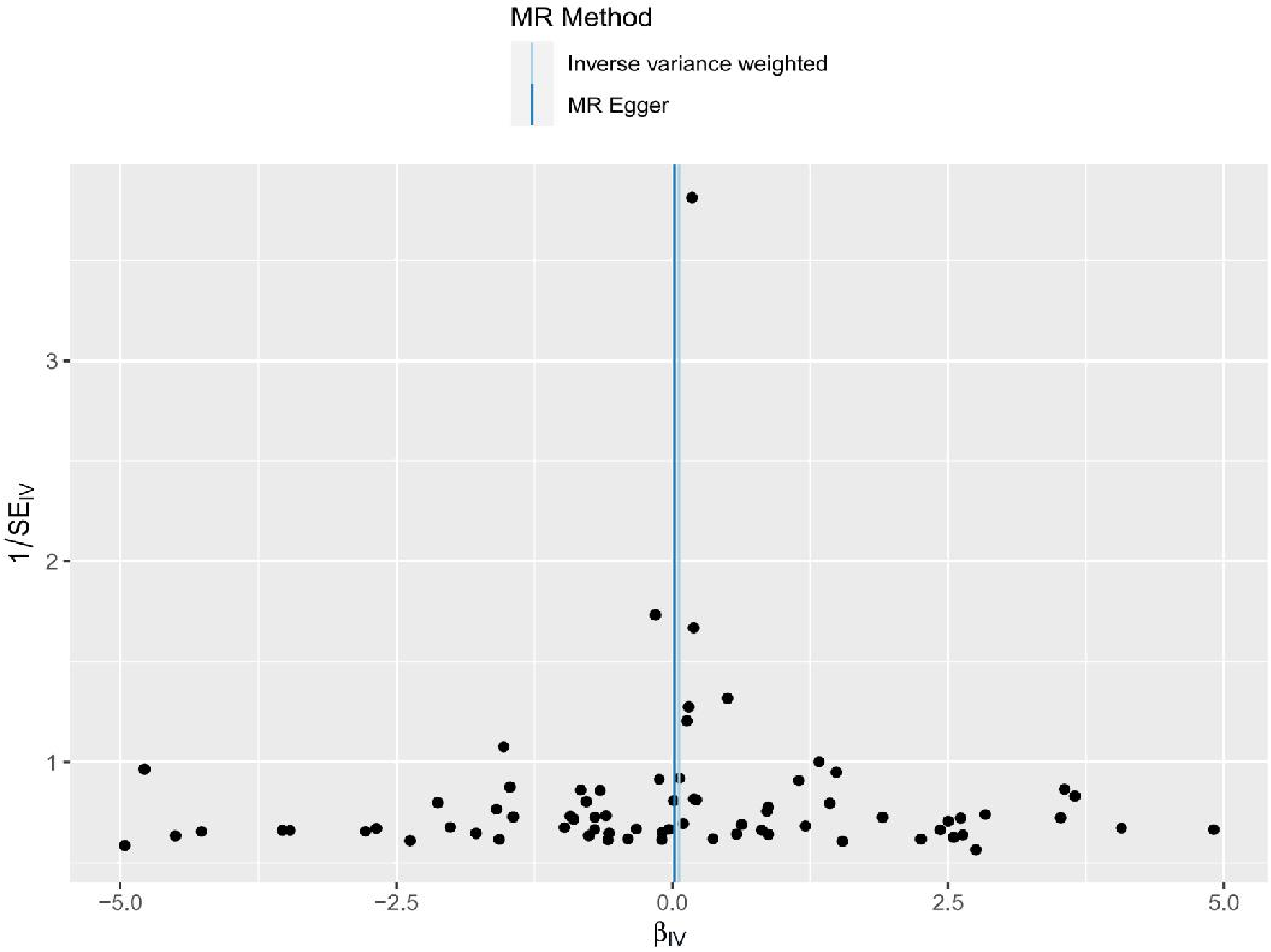

Supplementary Figure-14 Leave-one-out Analysis, Scatter Plot, Forest Plot, and Funnel Plot of Facial Aging on All Glioma

Supplementary Figure-14A Leave-one-out Analysis

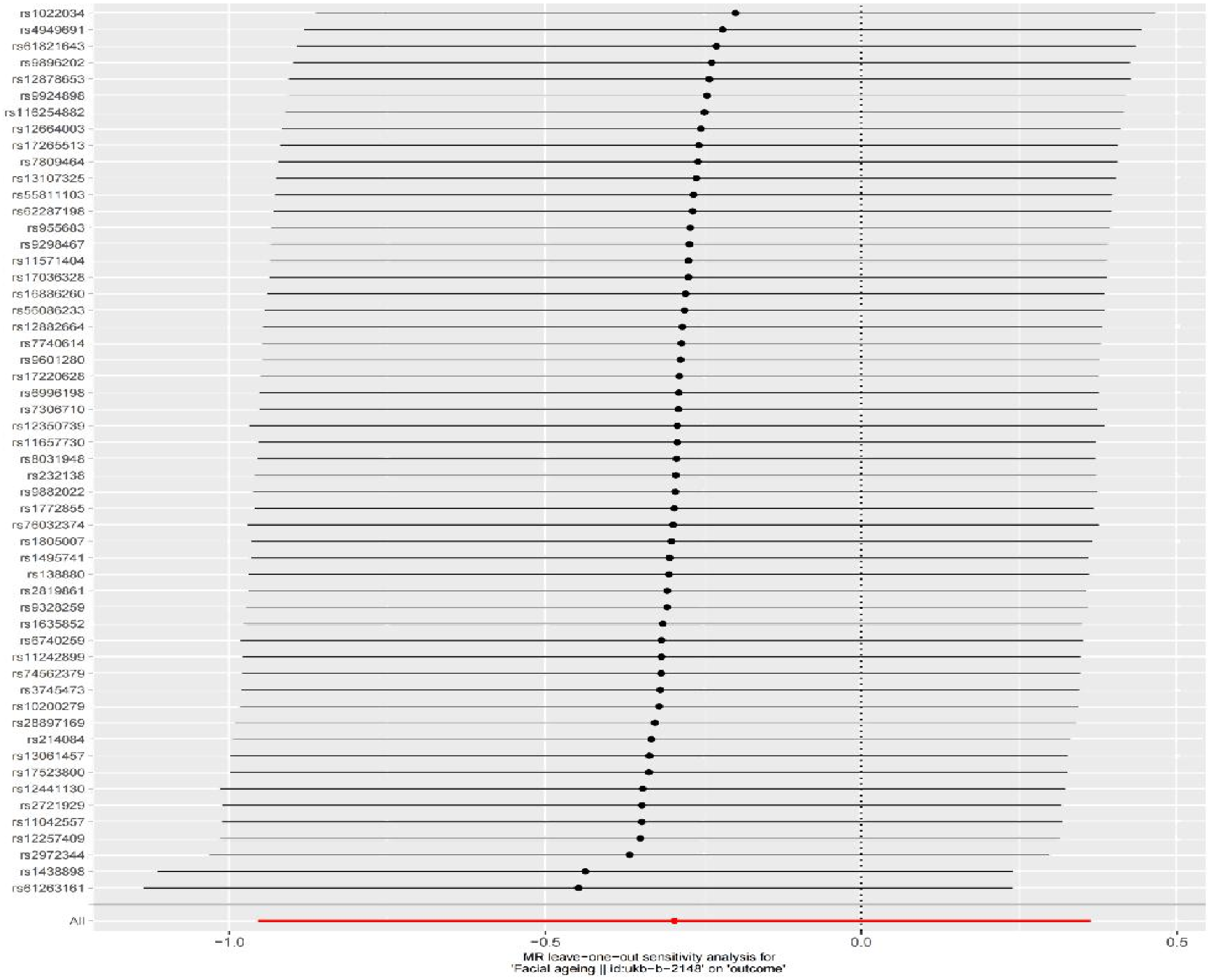

Supplementary Figure-14B Scatter

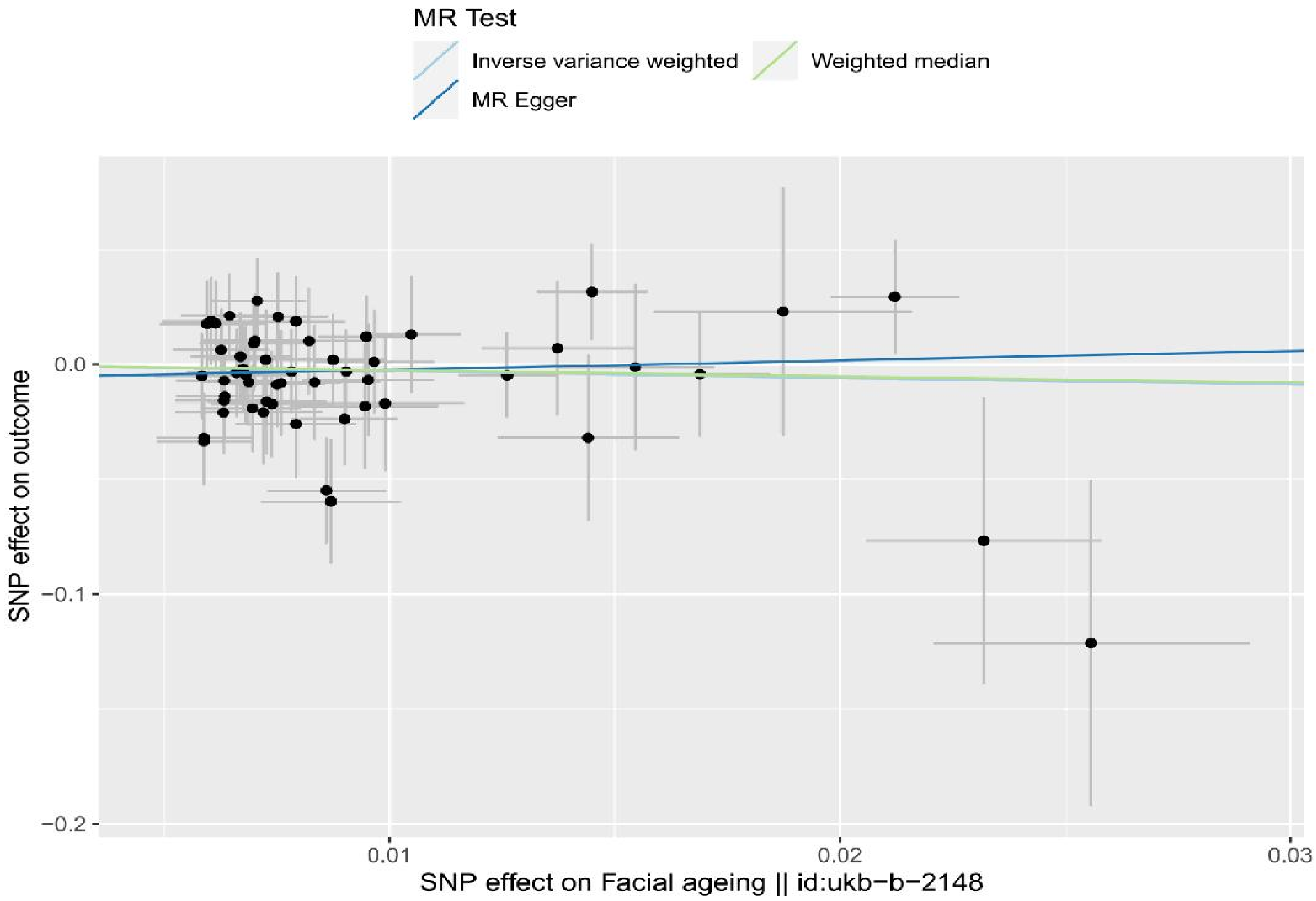

Supplementary Figure-14C Forest Plot

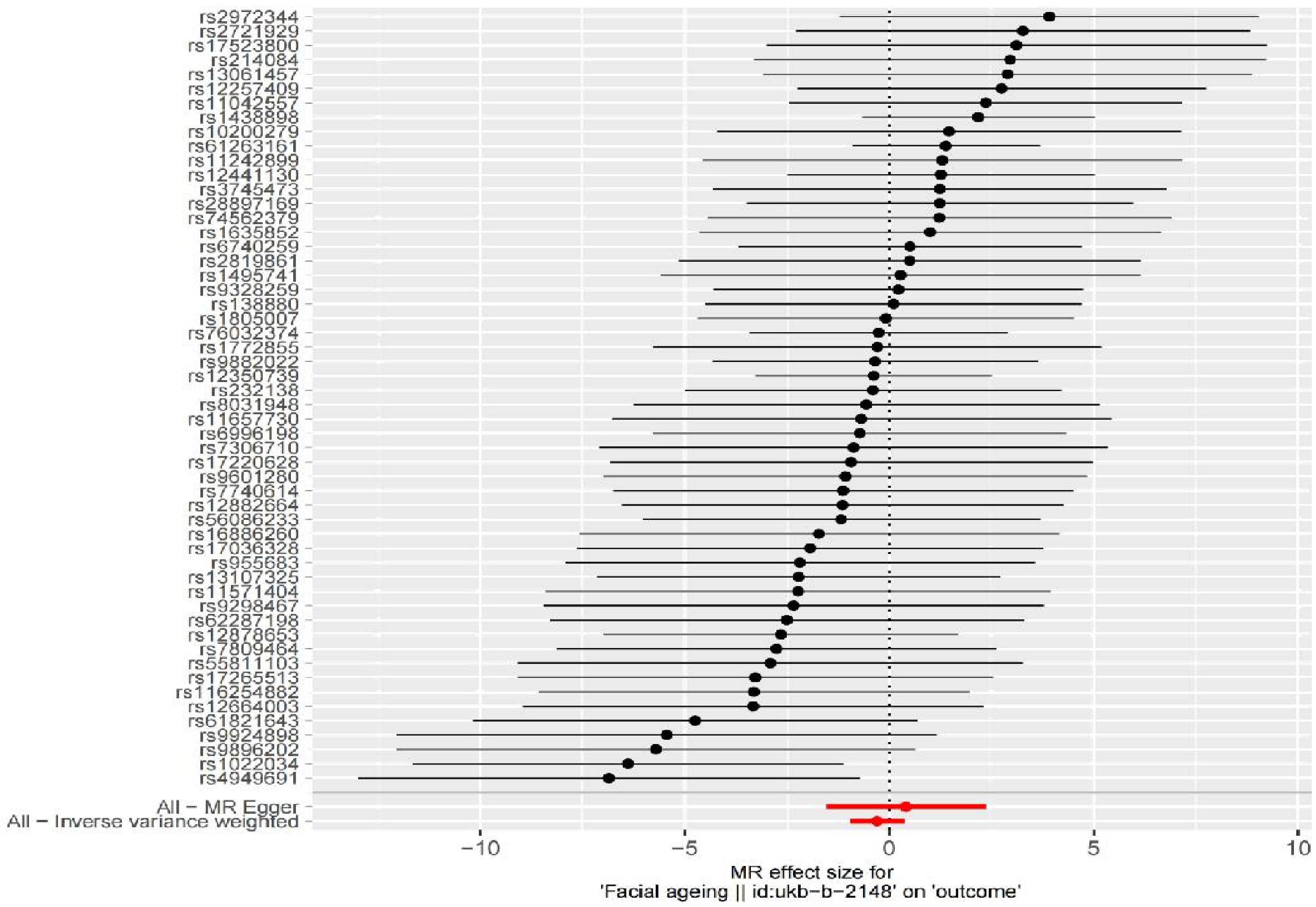

Supplementary Figure-14D Funnel Plot

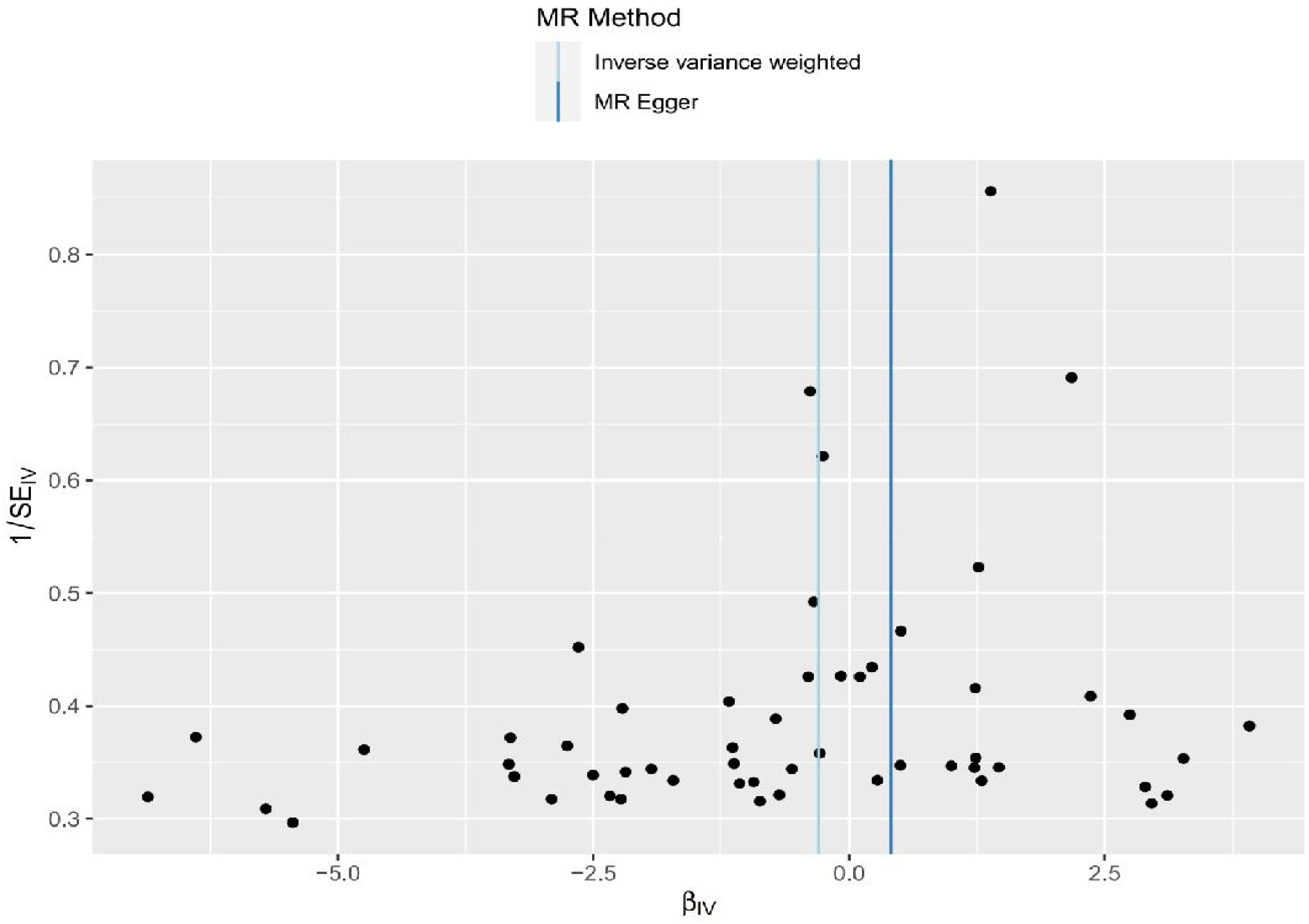

Supplementary Figure-15 Leave-one-out Analysis, Scatter Plot, Forest Plot, and Funnel Plot of Facial Aging on Amyotrophic Lateral Sclerosis

Supplementary Figure-15A Leave-one-out Analysis

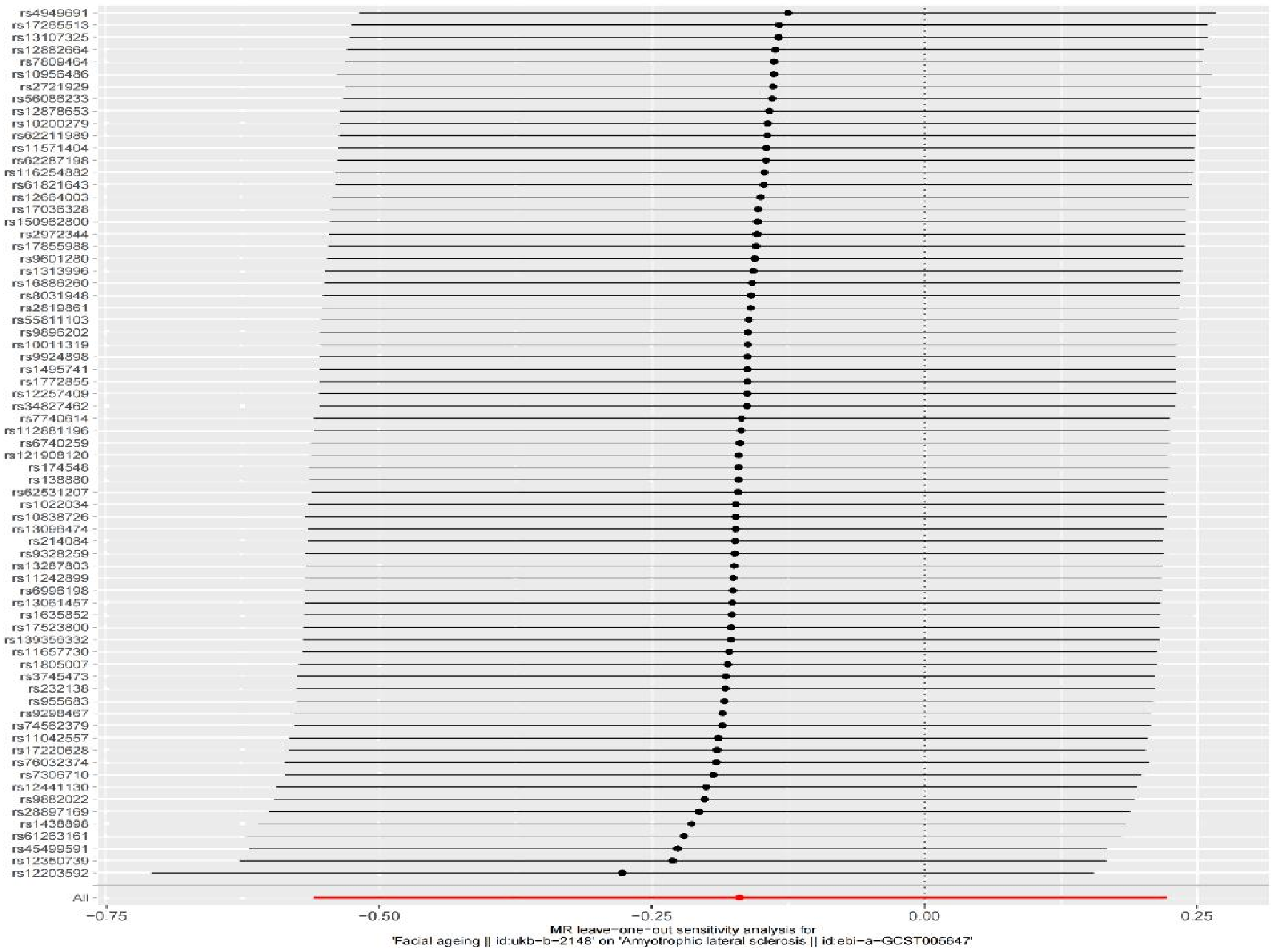

Supplementary Figure-15B Scatter

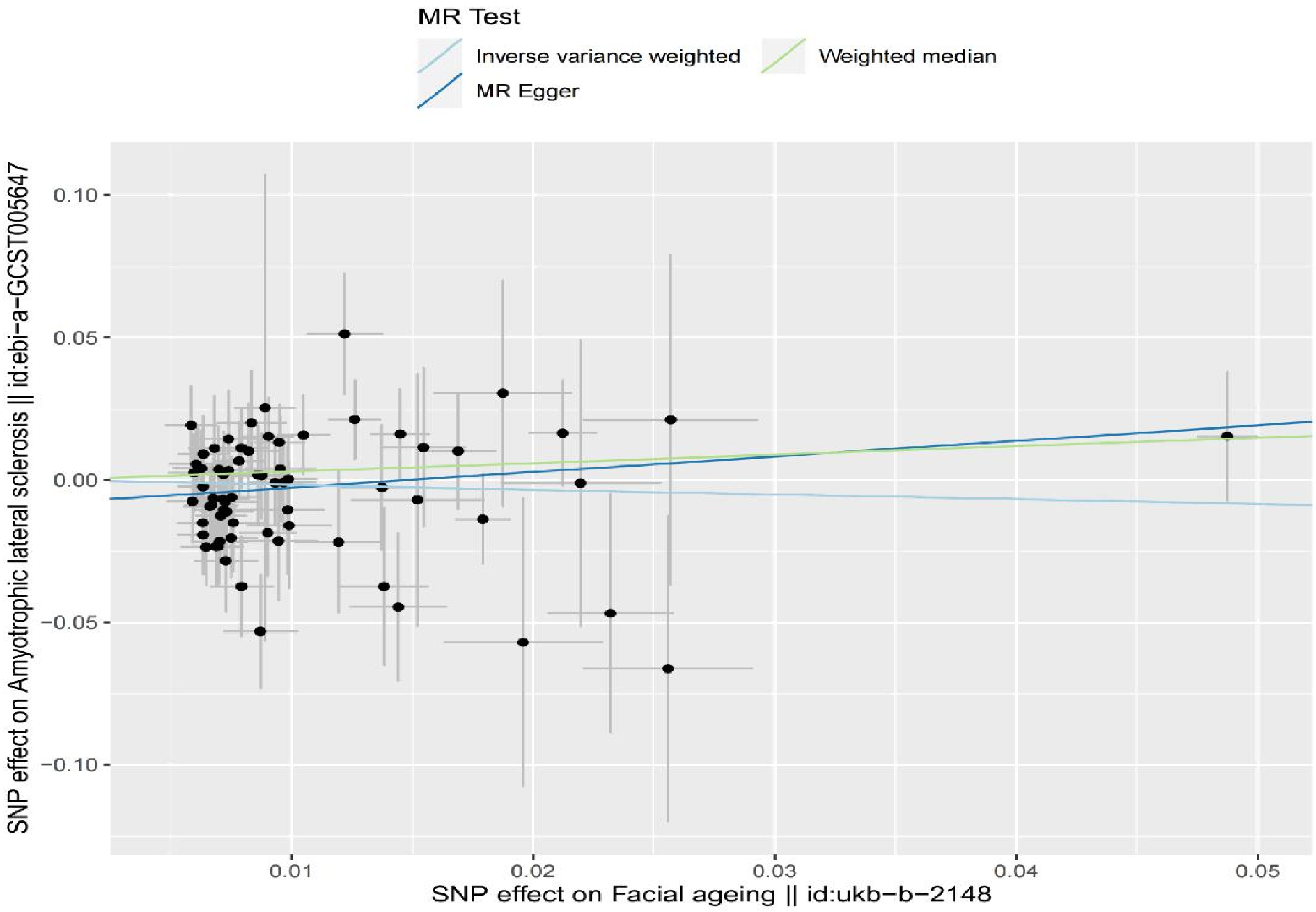

Supplementary Figure-15C Forest Plot

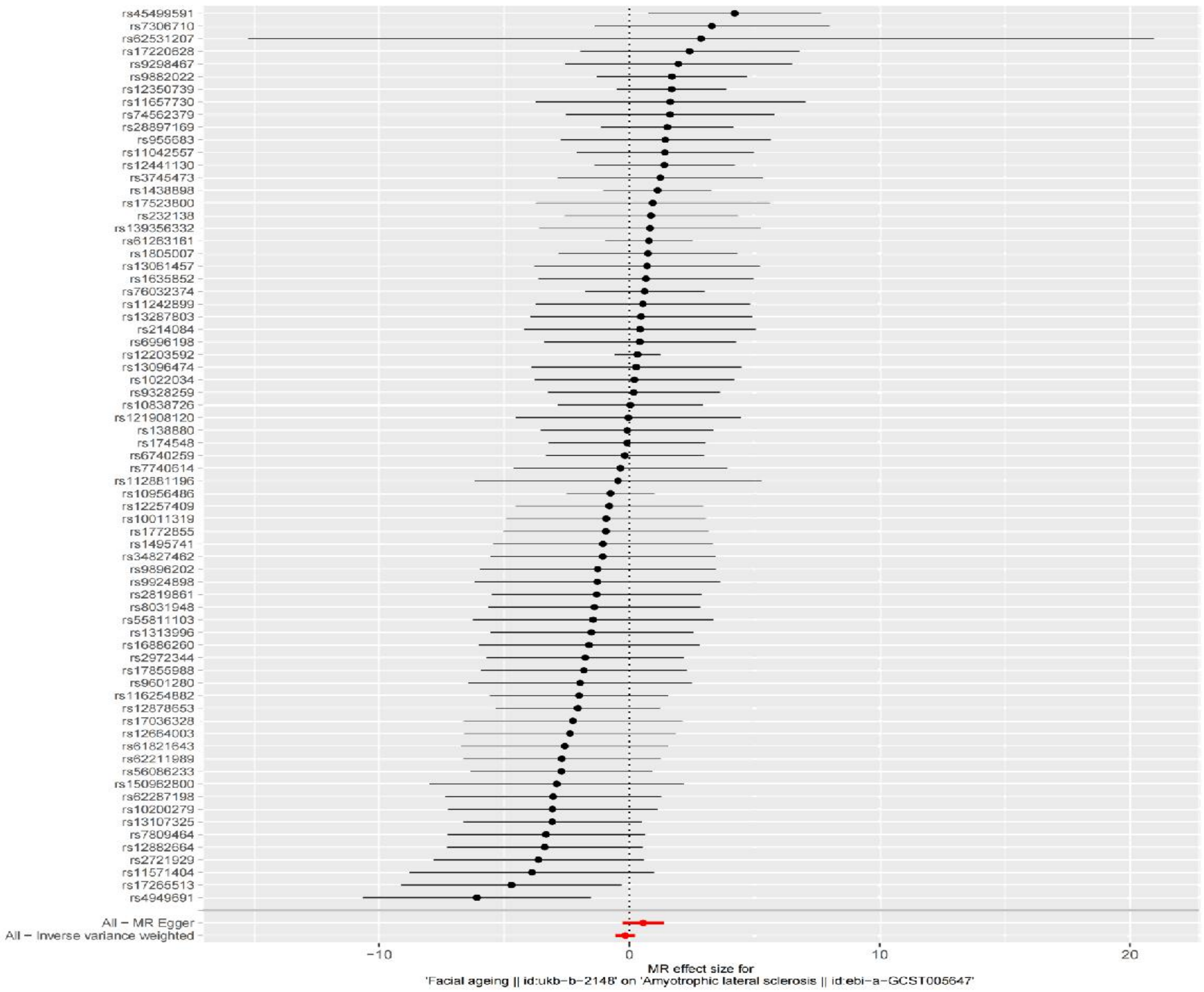

Supplementary Figure-15D Funnel Plot

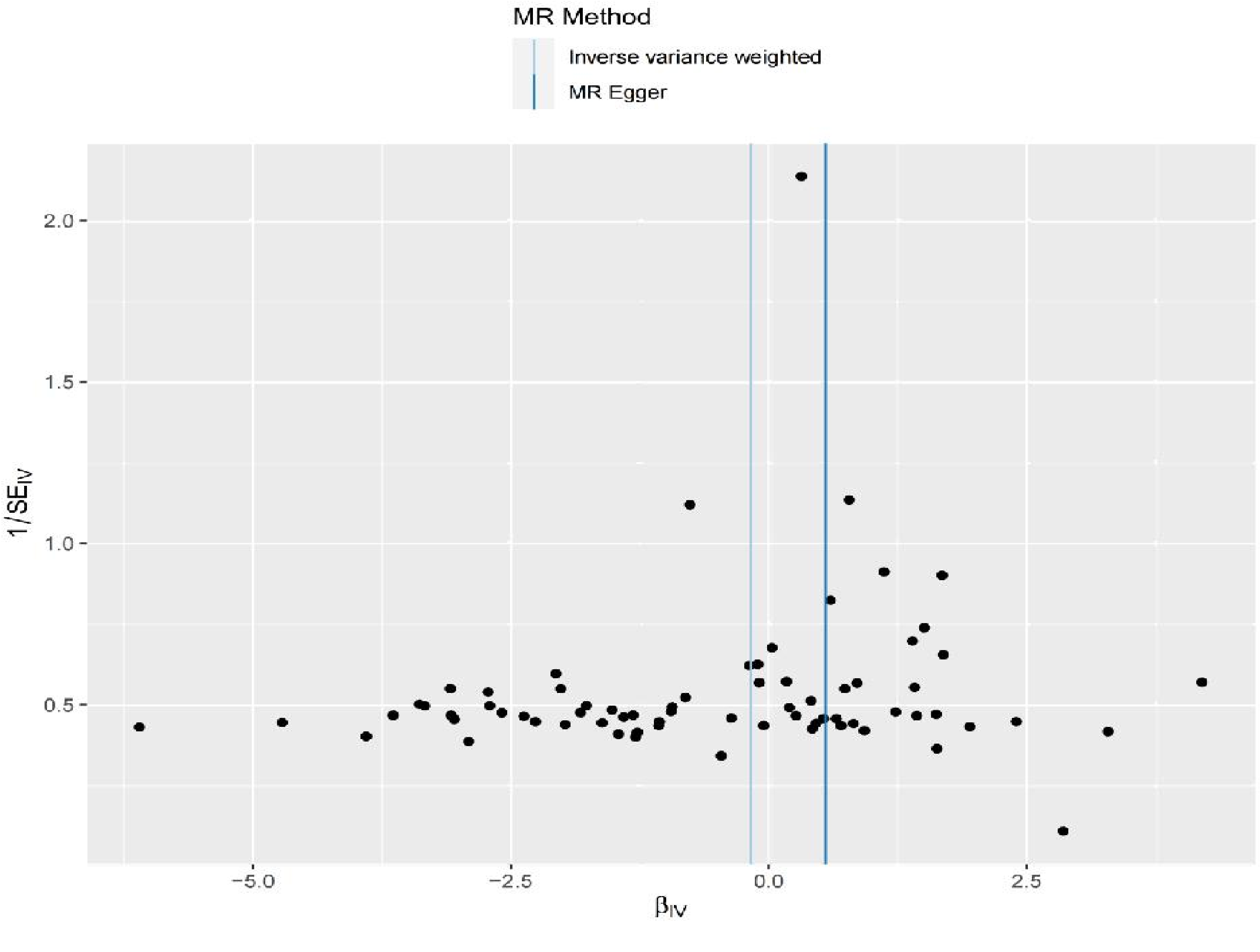

Supplementary Figure-16 Leave-one-out Analysis, Scatter Plot, Forest Plot, and Funnel Plot of Facial Aging on GBM

Supplementary Figure-16A Leave-one-out Analysis

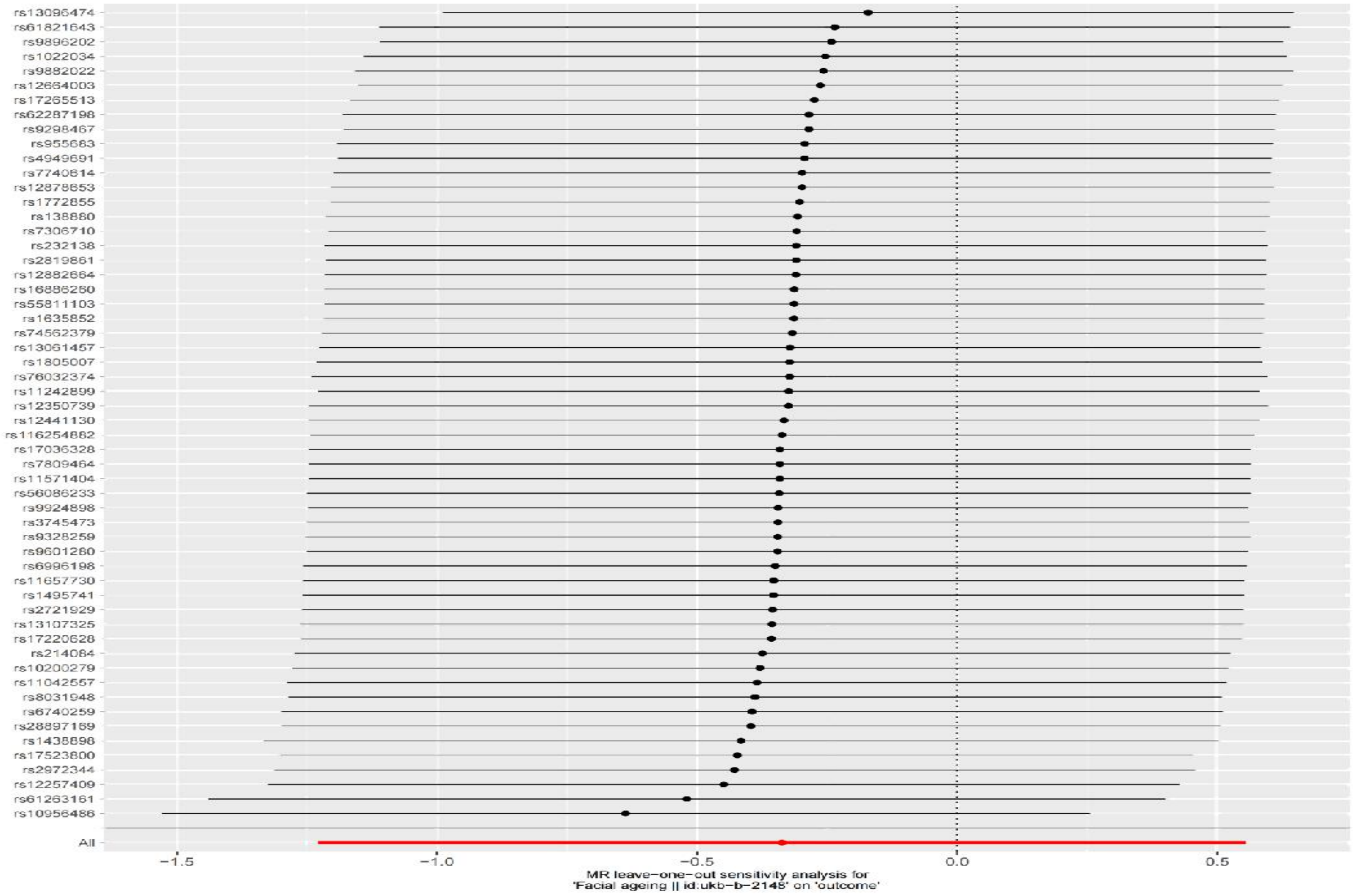

Supplementary Figure-16B Scatter

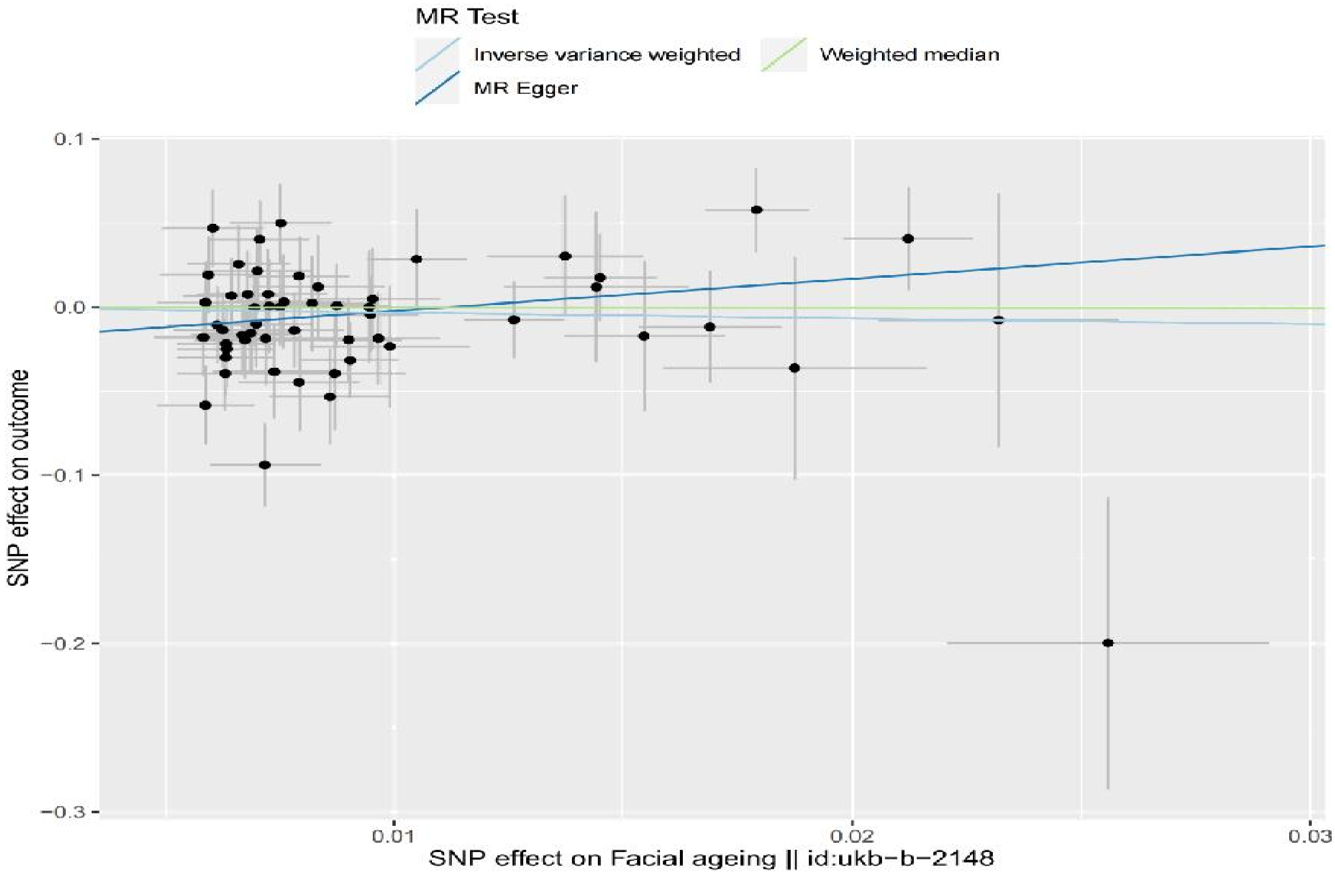

Supplementary Figure-16C Forest Plot

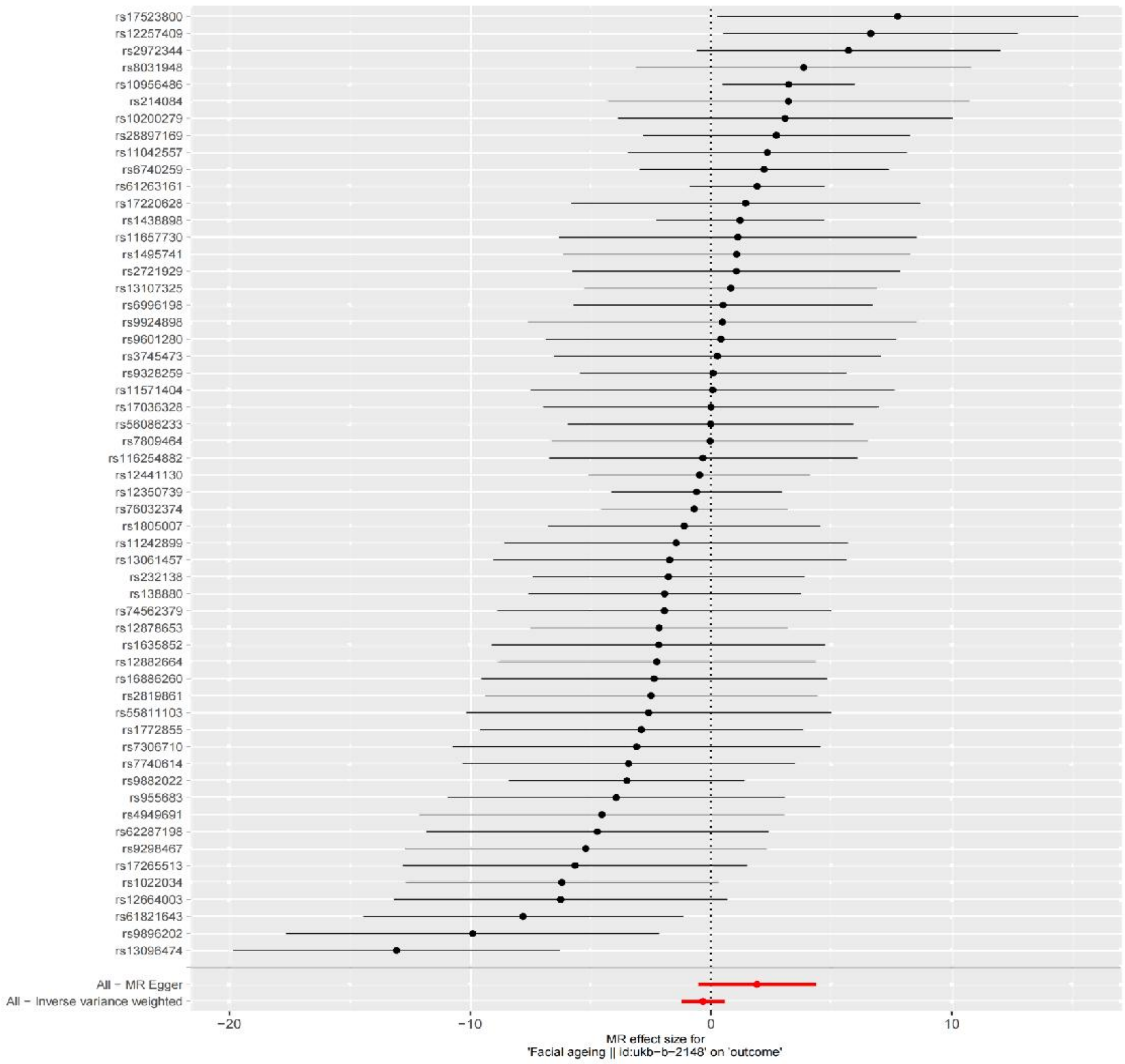

Supplementary Figure-16D Funnel Plot

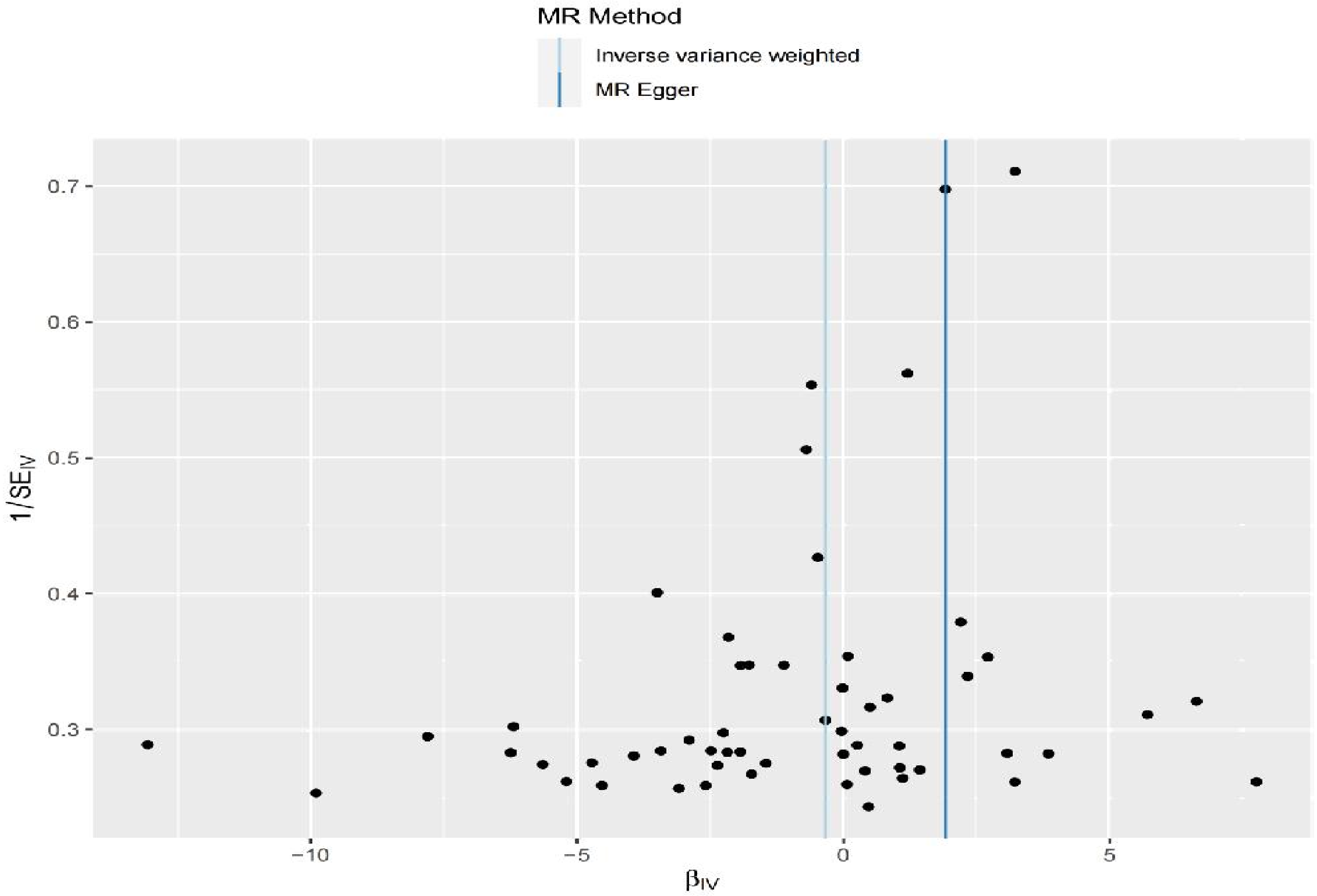

Supplementary Figure-17 Leave-one-out Analysis, Scatter Plot, Forest Plot, and Funnel Plot of Facial Aging on Meningioma

Supplementary Figure-17A Leave-one-out Analysis

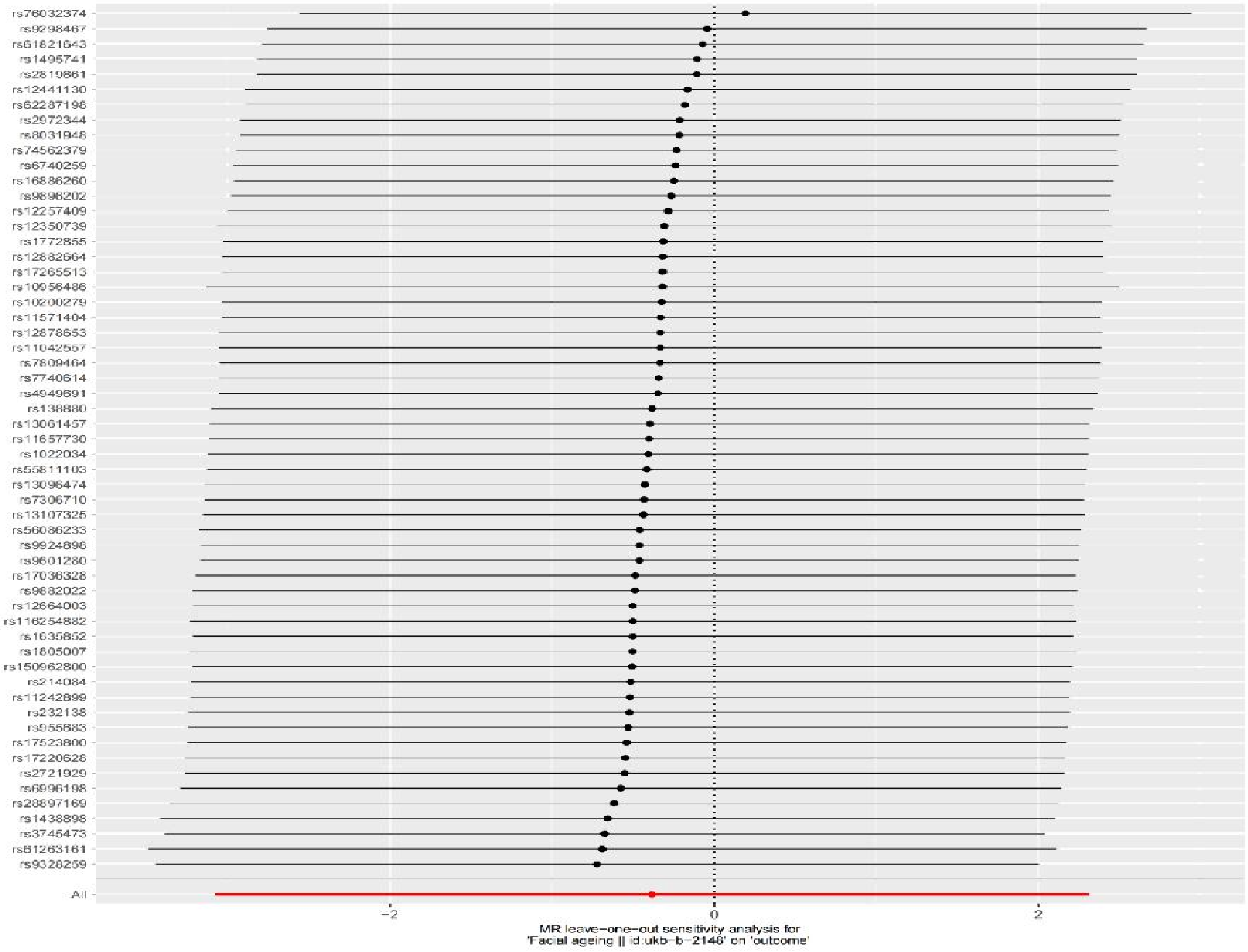

Supplementary Figure-17B Scatter

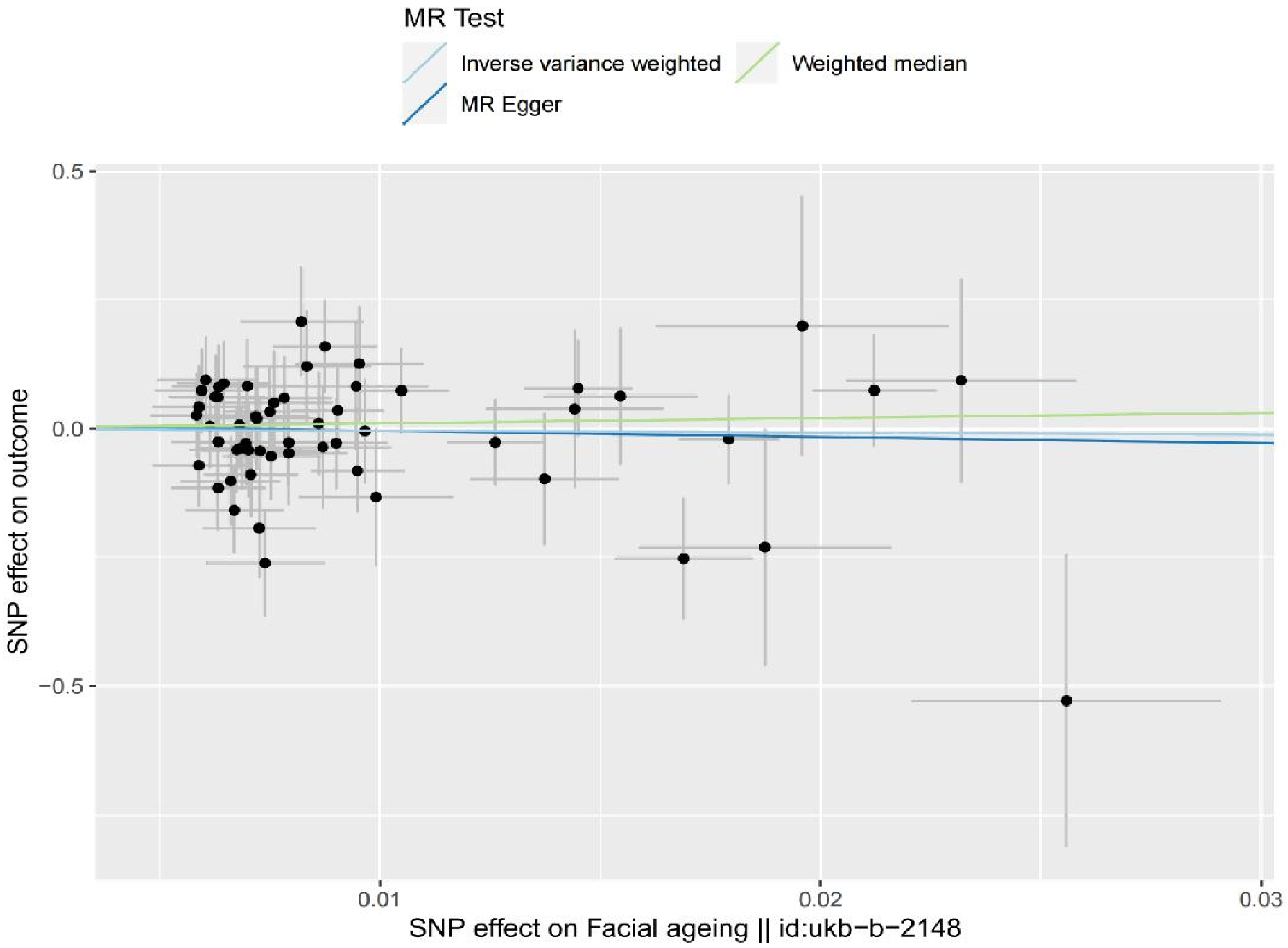

Supplementary Figure-17C Forest Plot

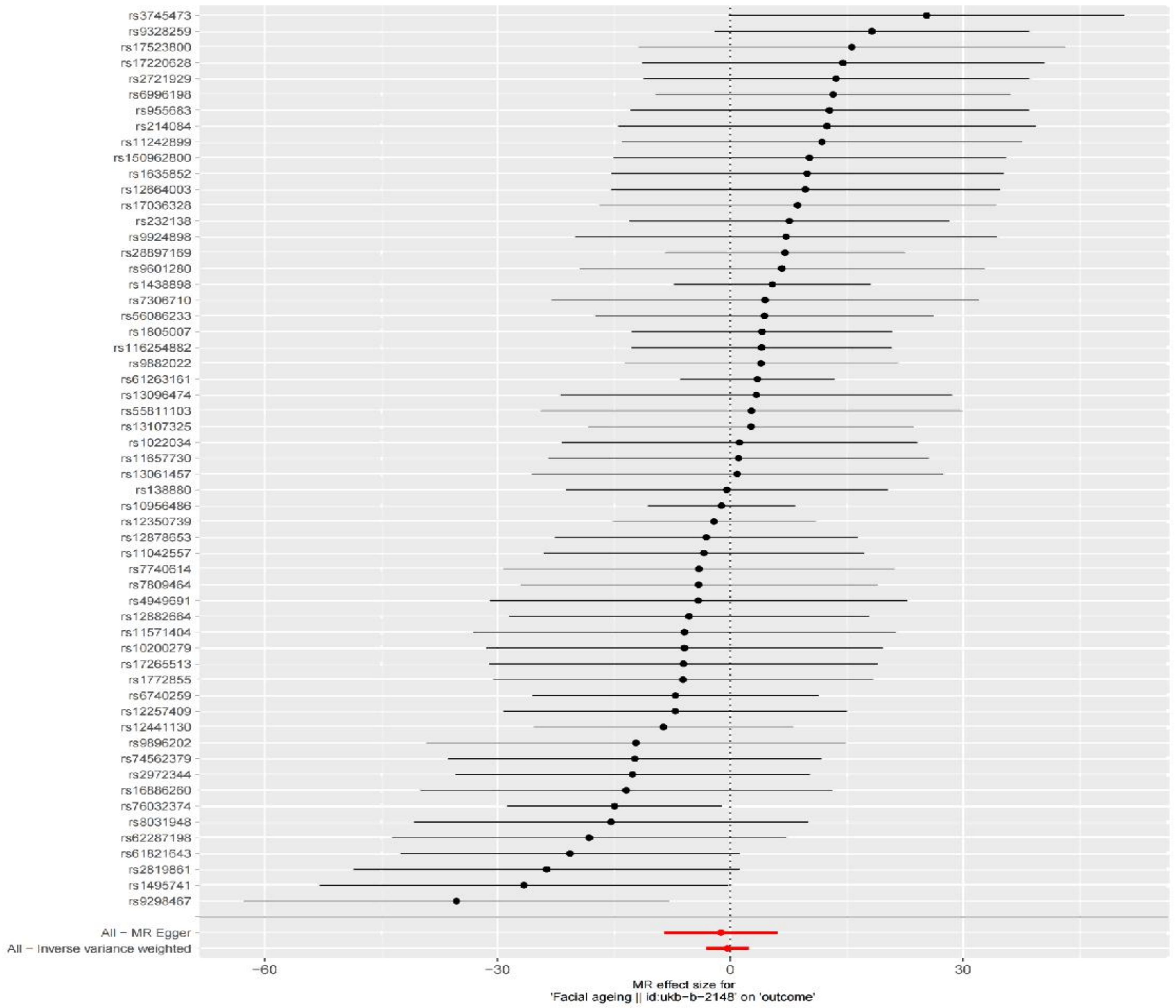

Supplementary Figure-17D Funnel Plot

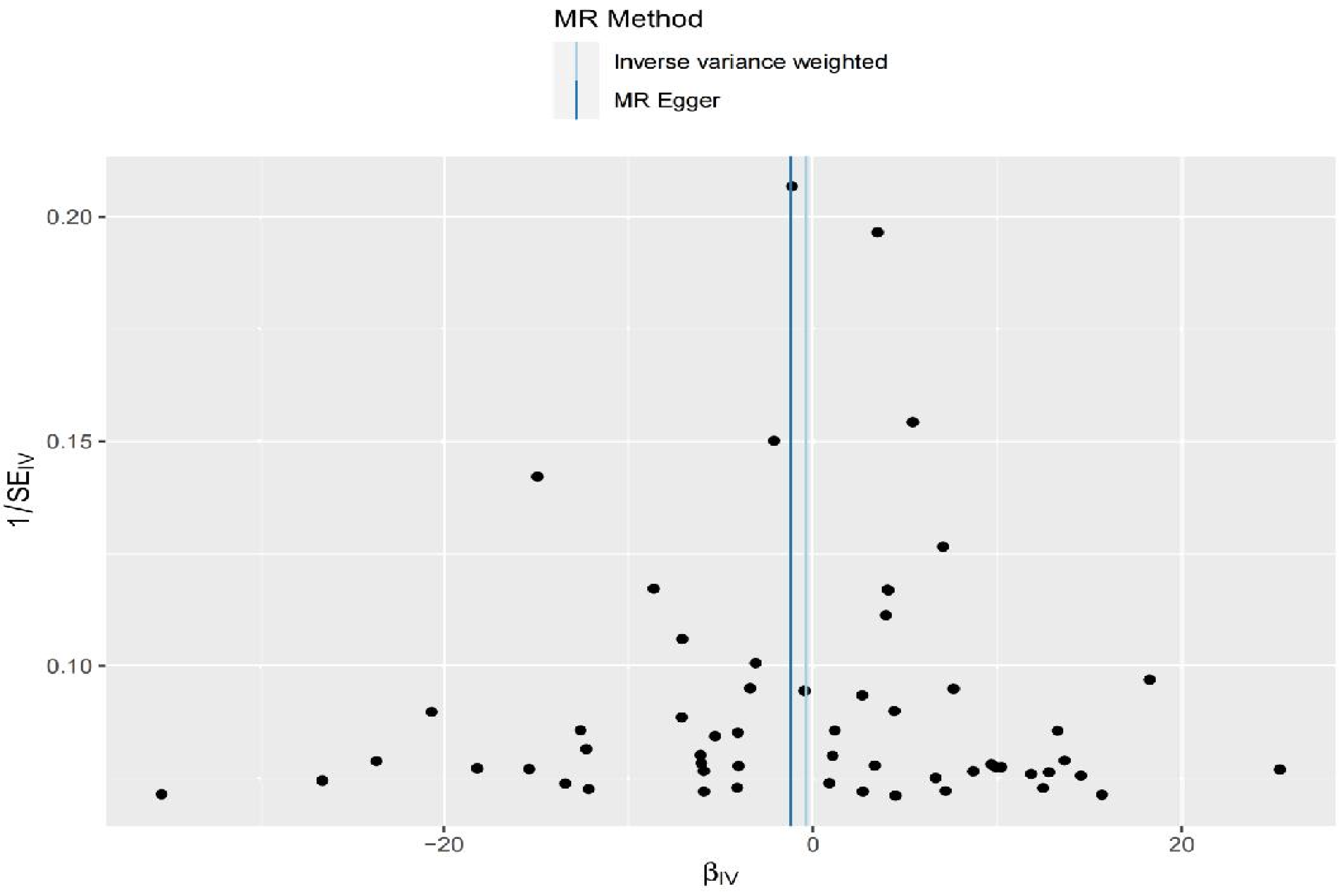

Supplementary Figure-18 Leave-one-out Analysis, Scatter Plot, Forest Plot, and Funnel Plot of Facial Aging on Non-GBM

Supplementary Figure-18A Leave-one-out Analysis

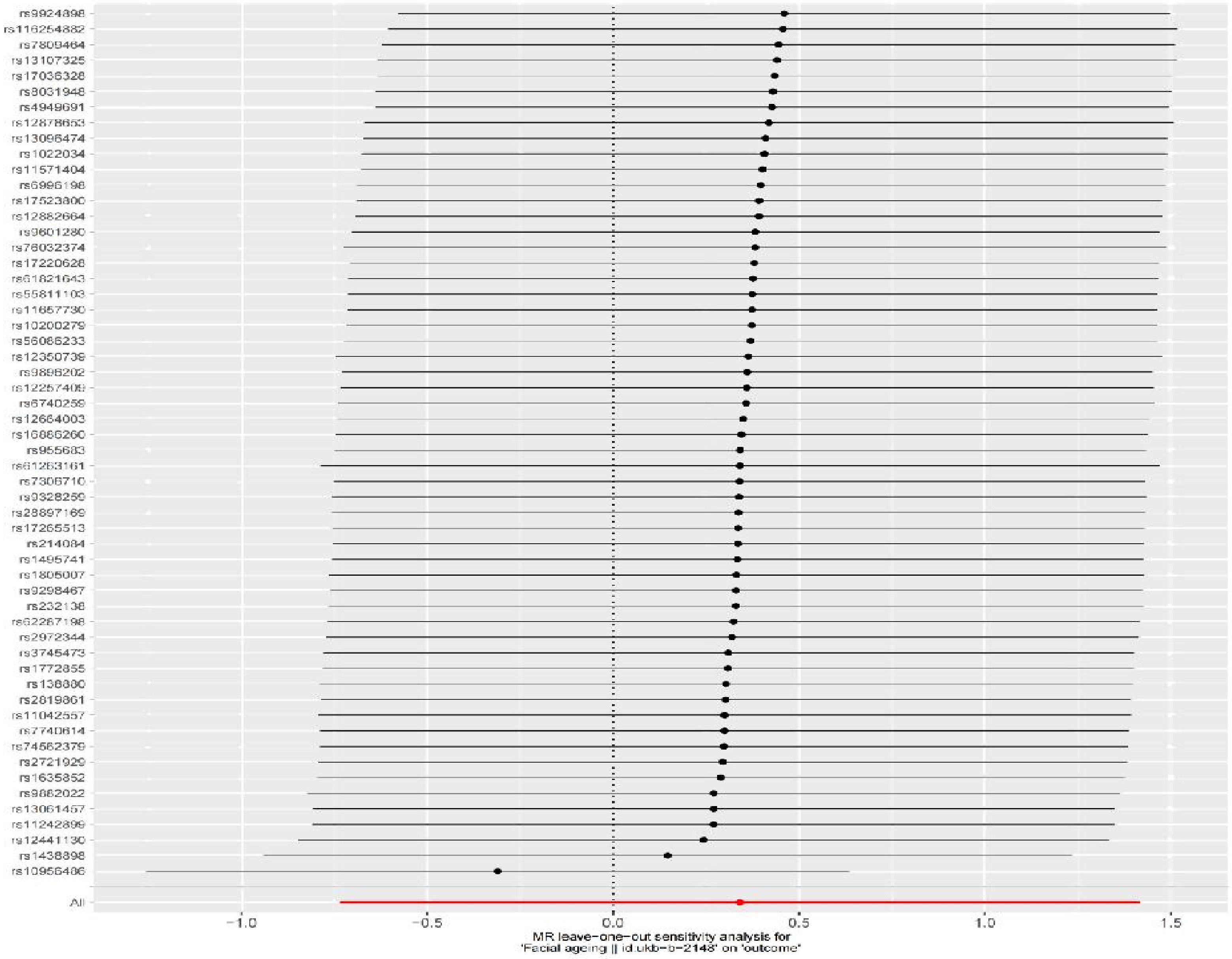

Supplementary Figure-18B Scatter

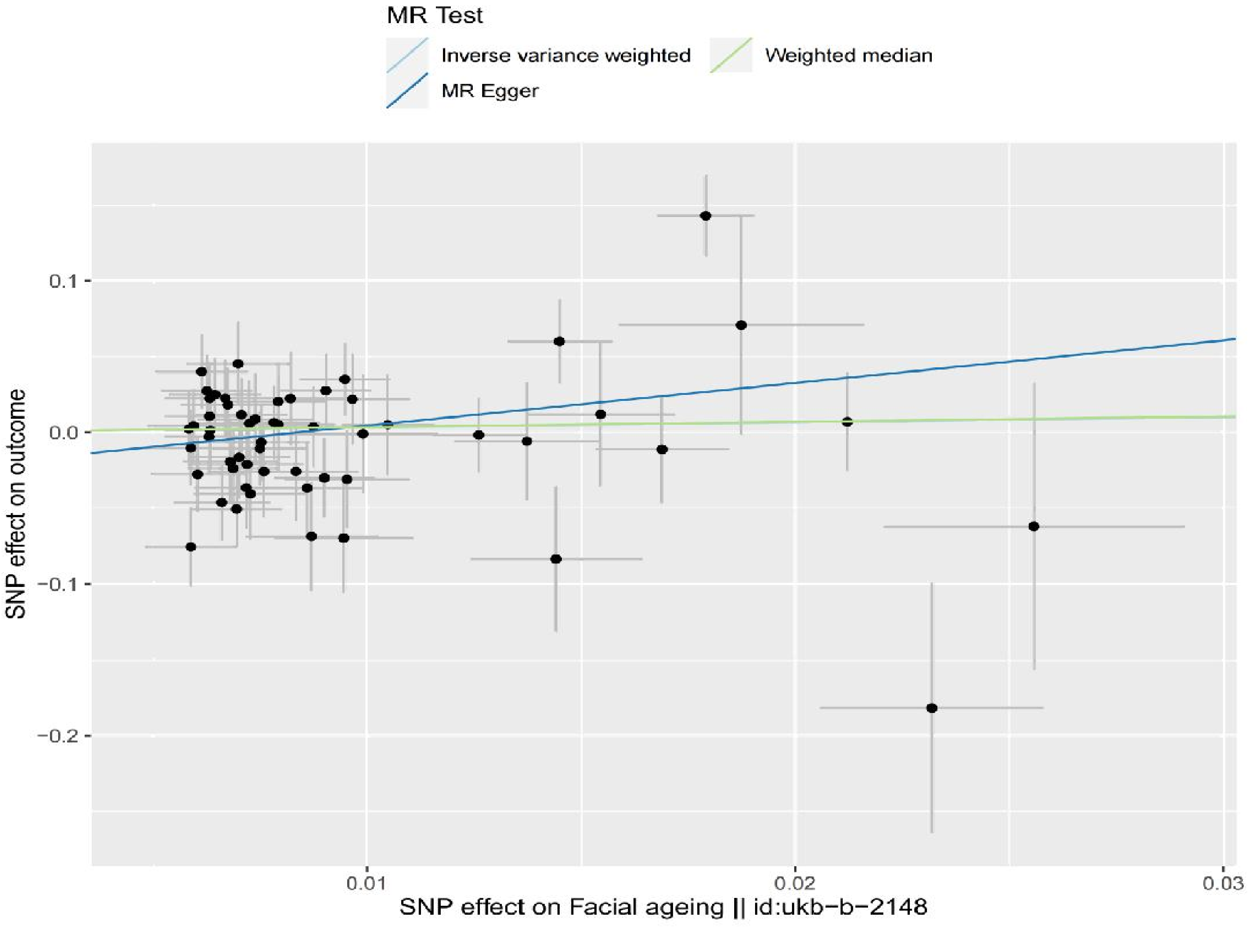

Supplementary Figure-18C Forest Plot

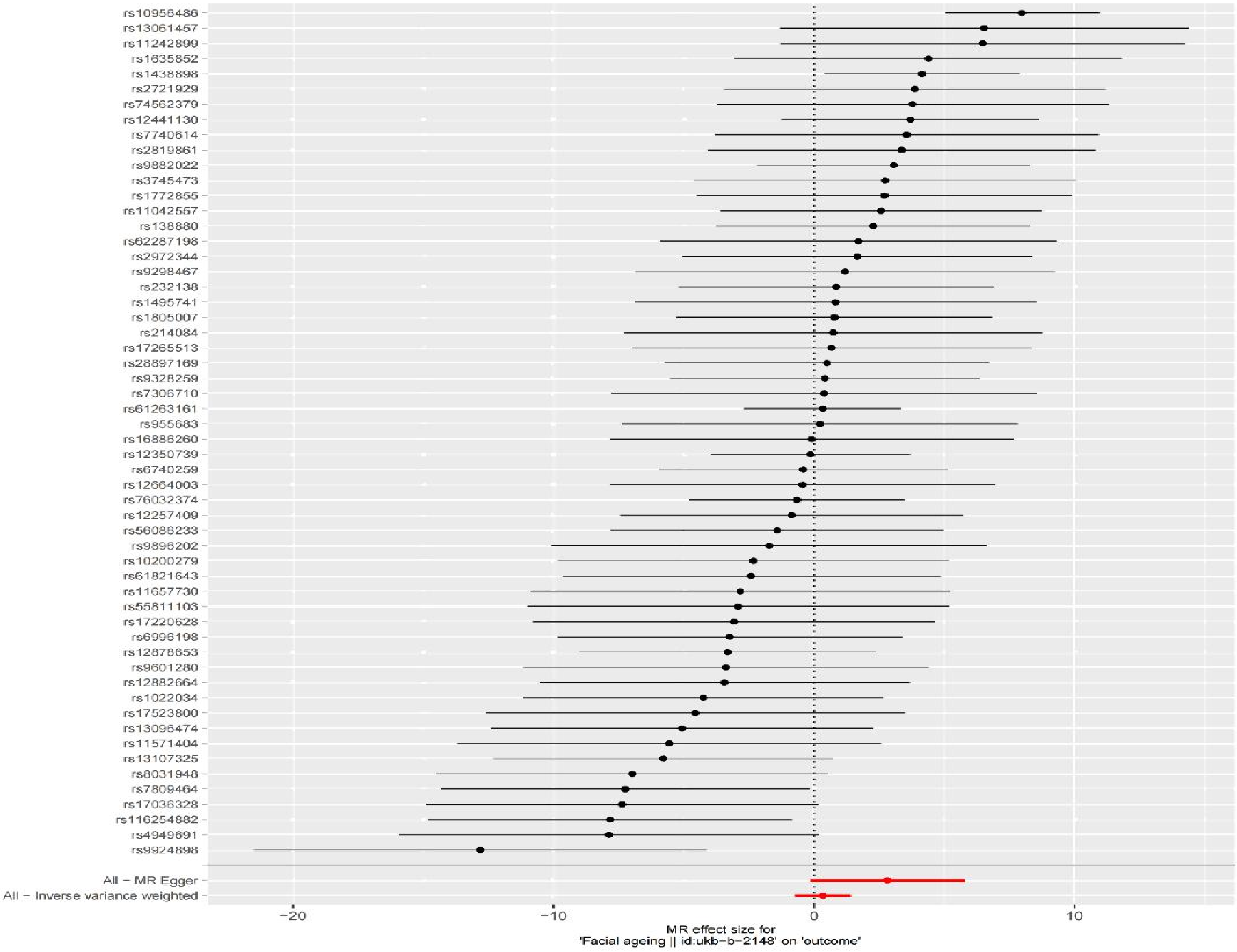

Supplementary Figure-18D Funnel Plot

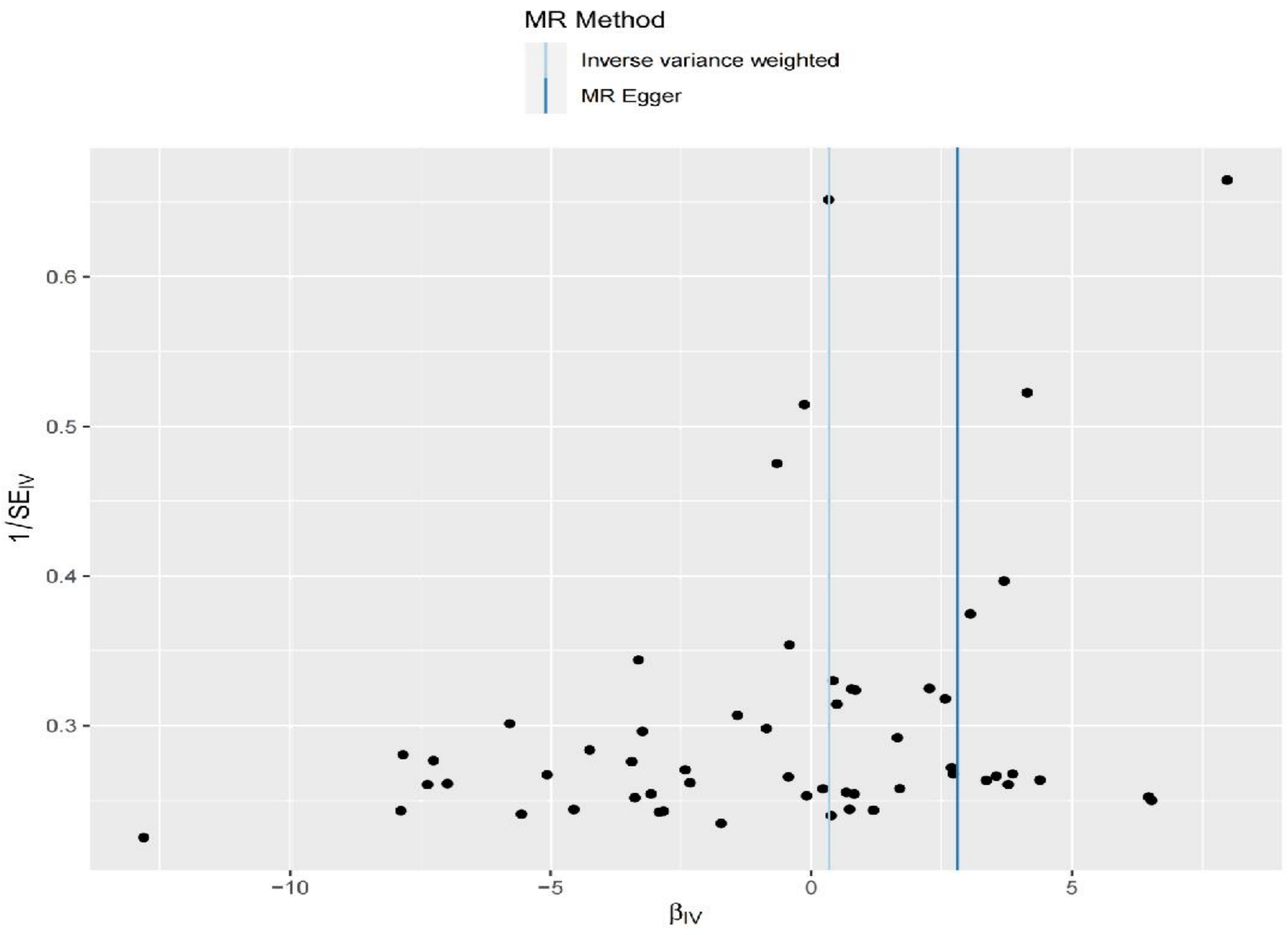

Supplementary Figure-19 Leave-one-out Analysis, Scatter Plot, Forest Plot, and Funnel Plot of Facial Aging on Parkinson

Supplementary Figure-19A Leave-one-out Analysis

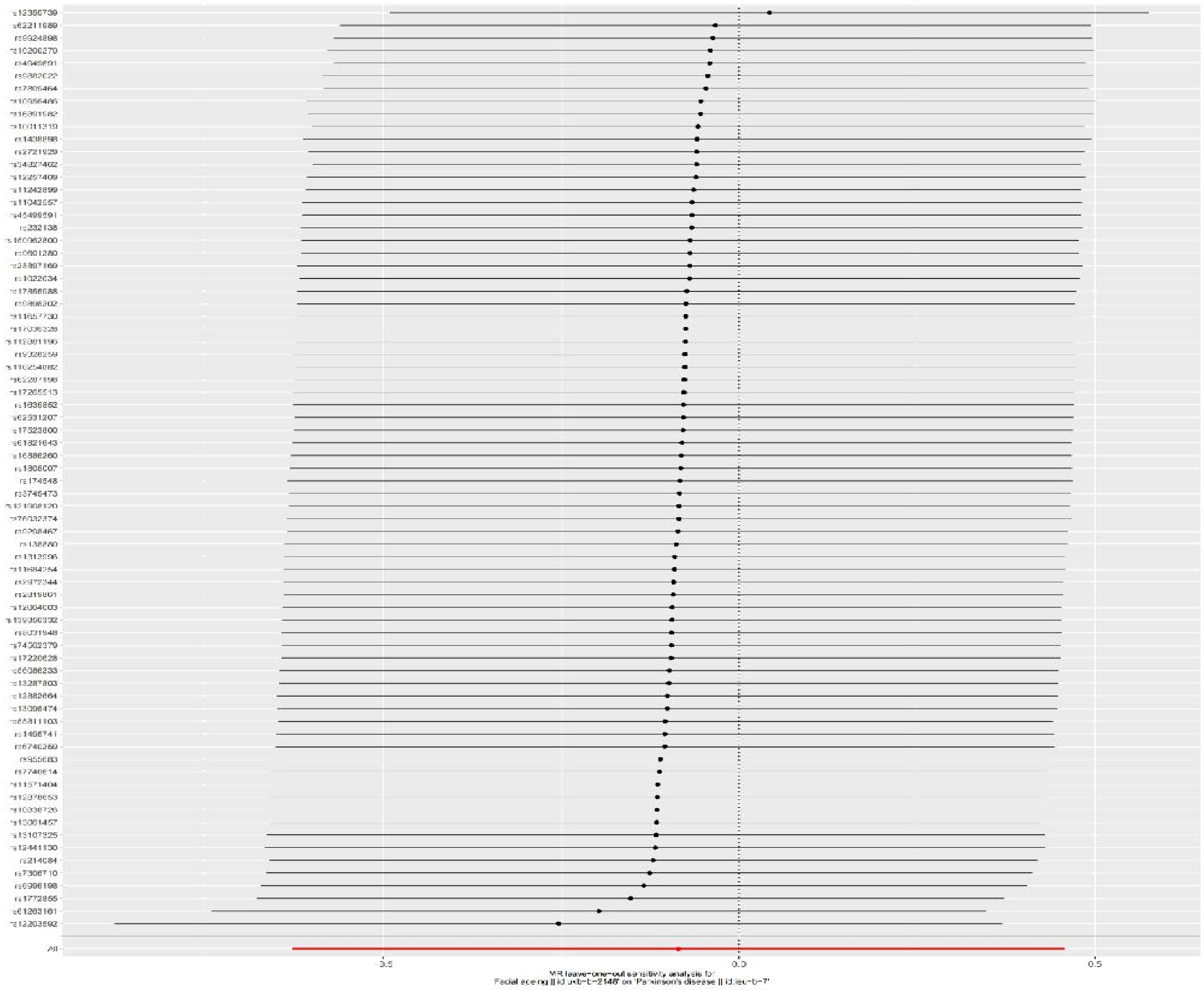

Supplementary Figure-19B Scatter

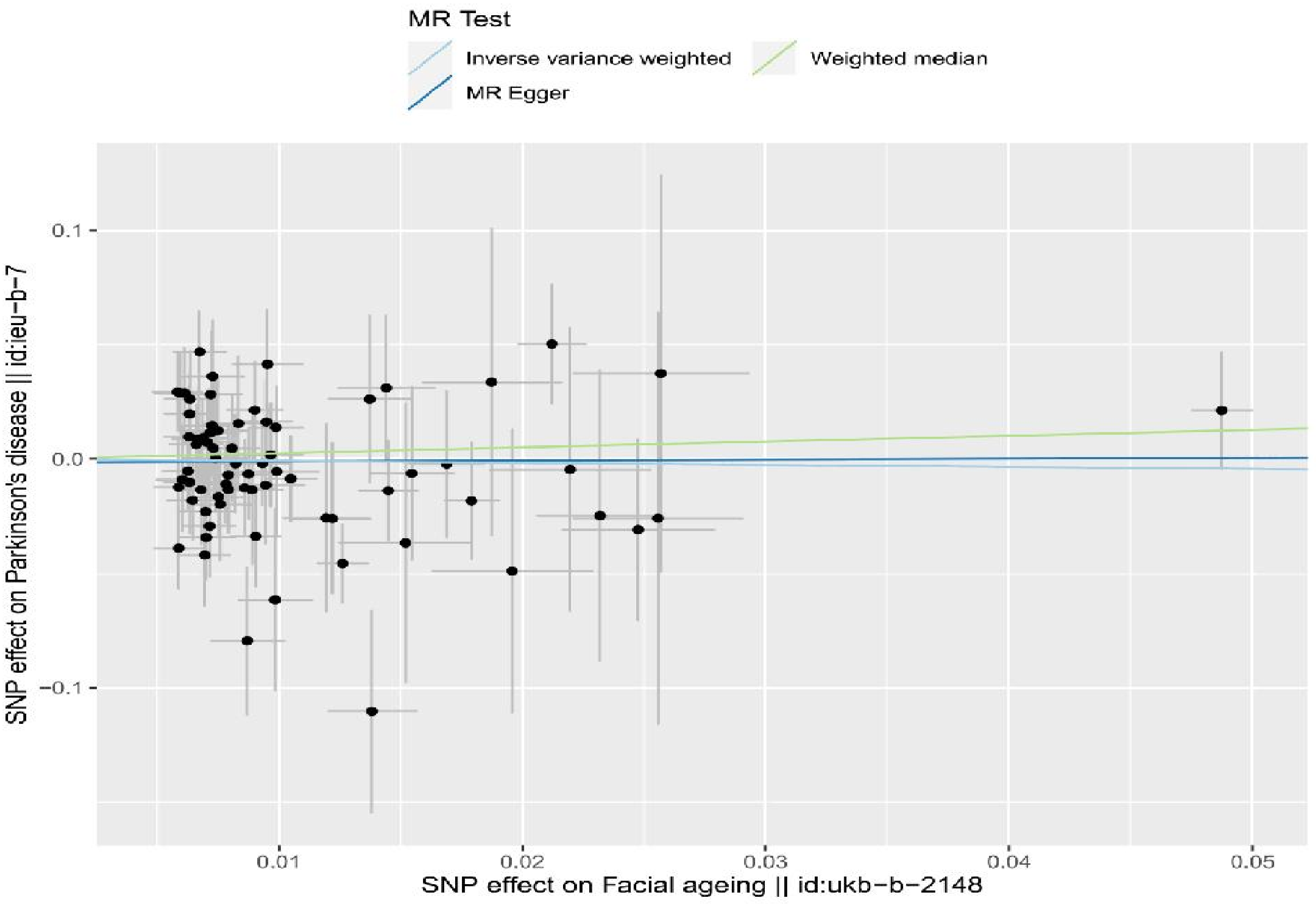

Supplementary Figure-19C Forest Plot

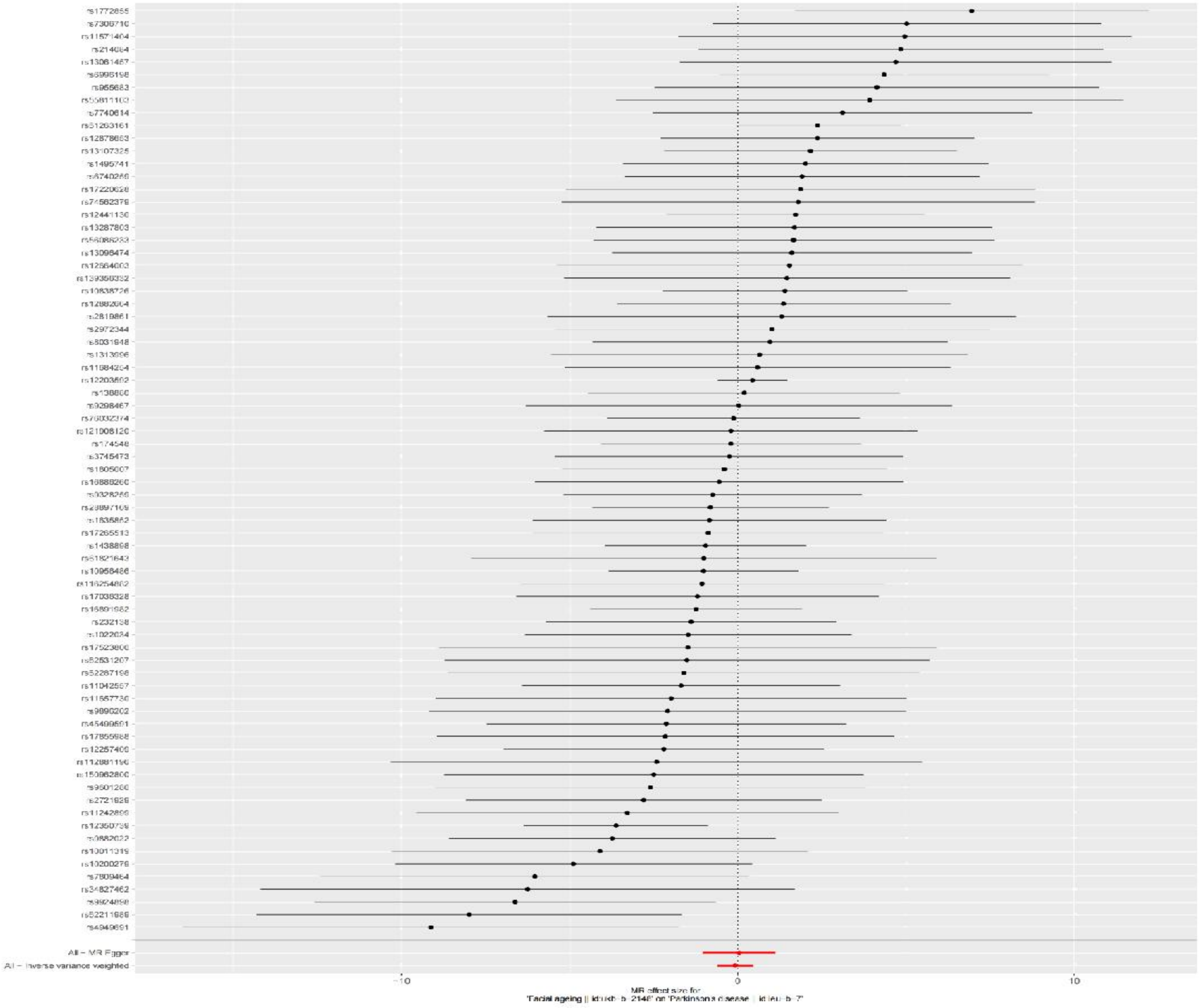

Supplementary Figure-19D Funnel Plot

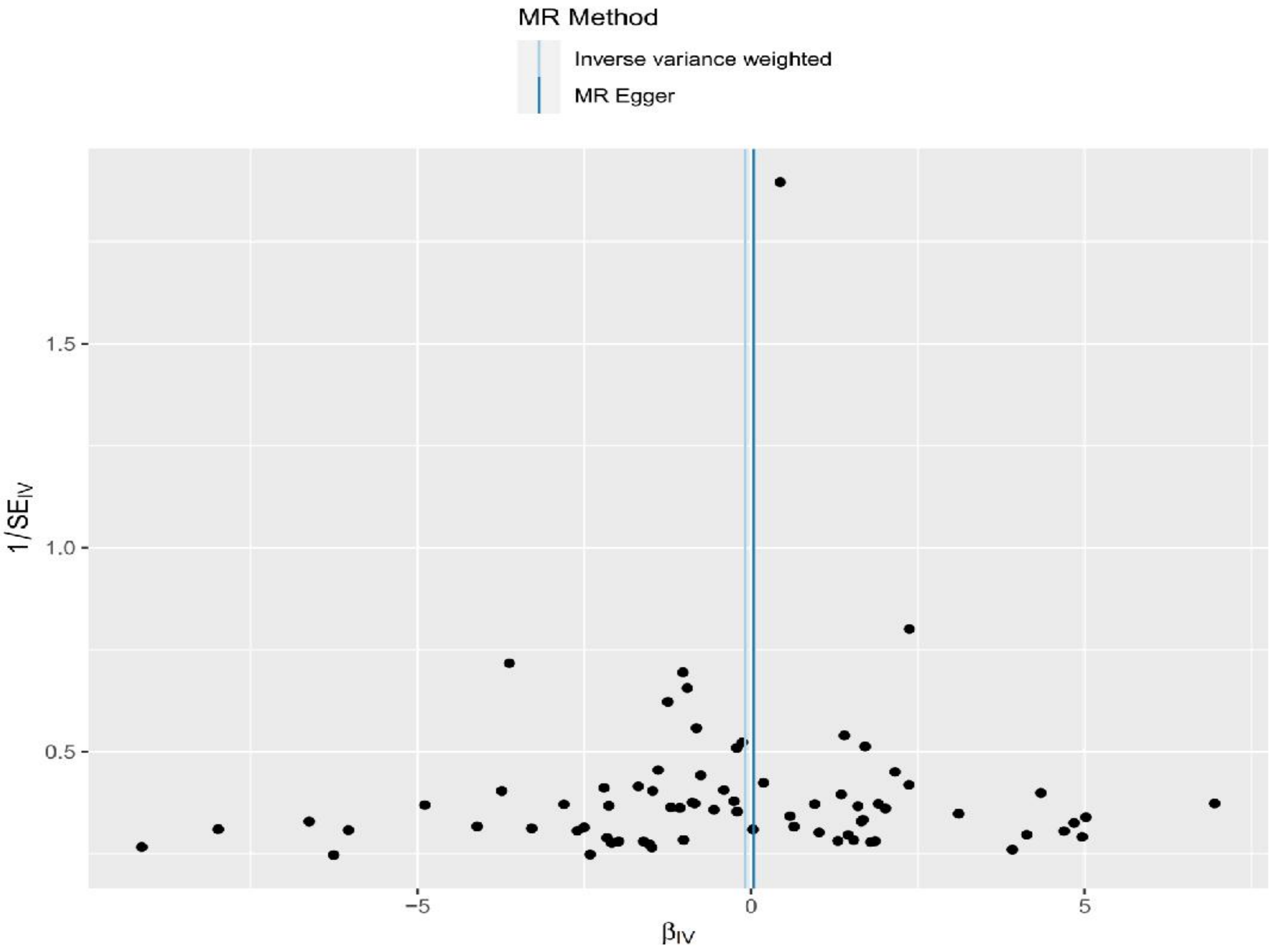

Supplementary Figure-20 Leave-one-out Analysis, Scatter Plot, Forest Plot, and Funnel Plot of Facial Aging on Vestibular Schwannomas

Supplementary Figure-20A Leave-one-out Analysis

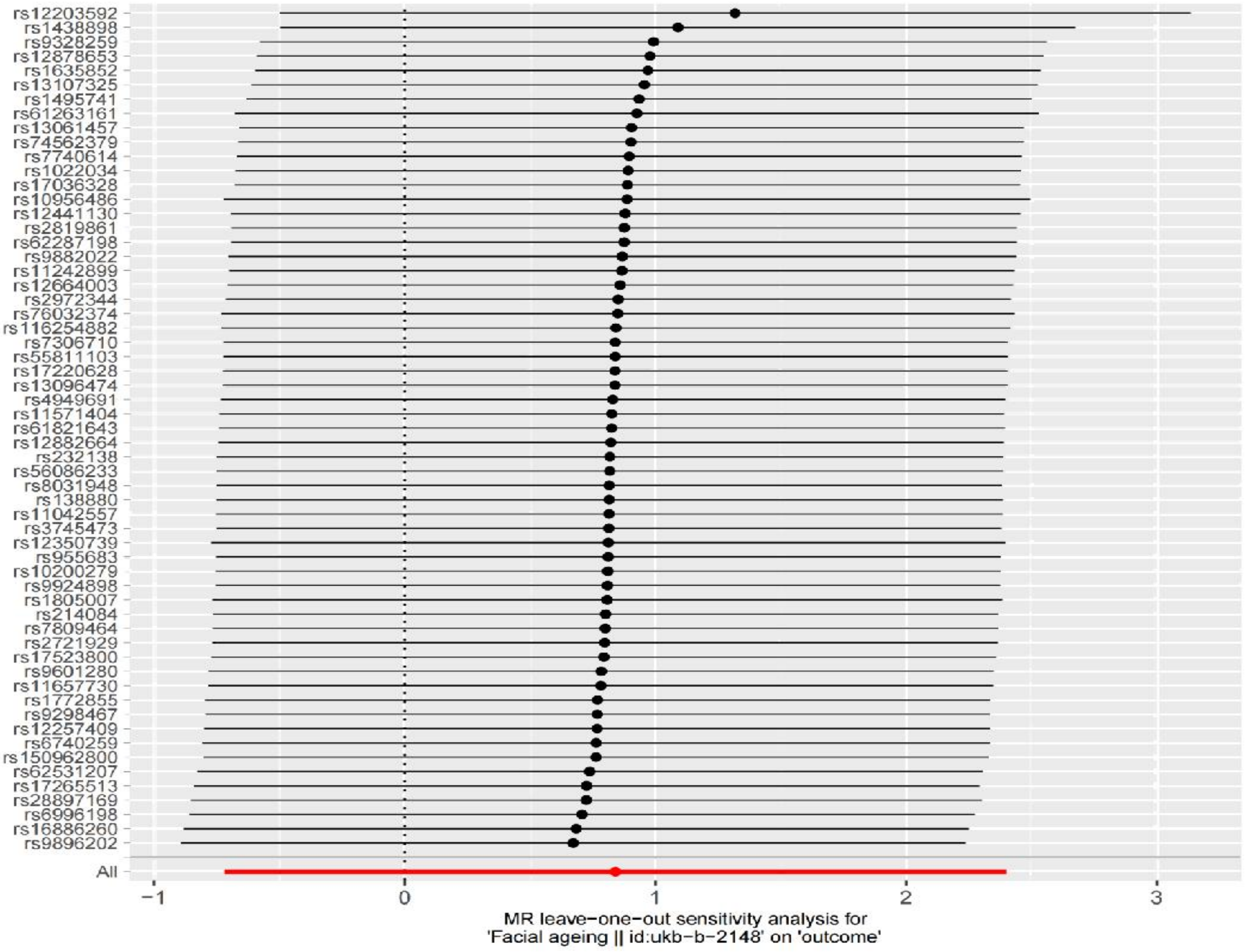

Supplementary Figure-20B Scatter

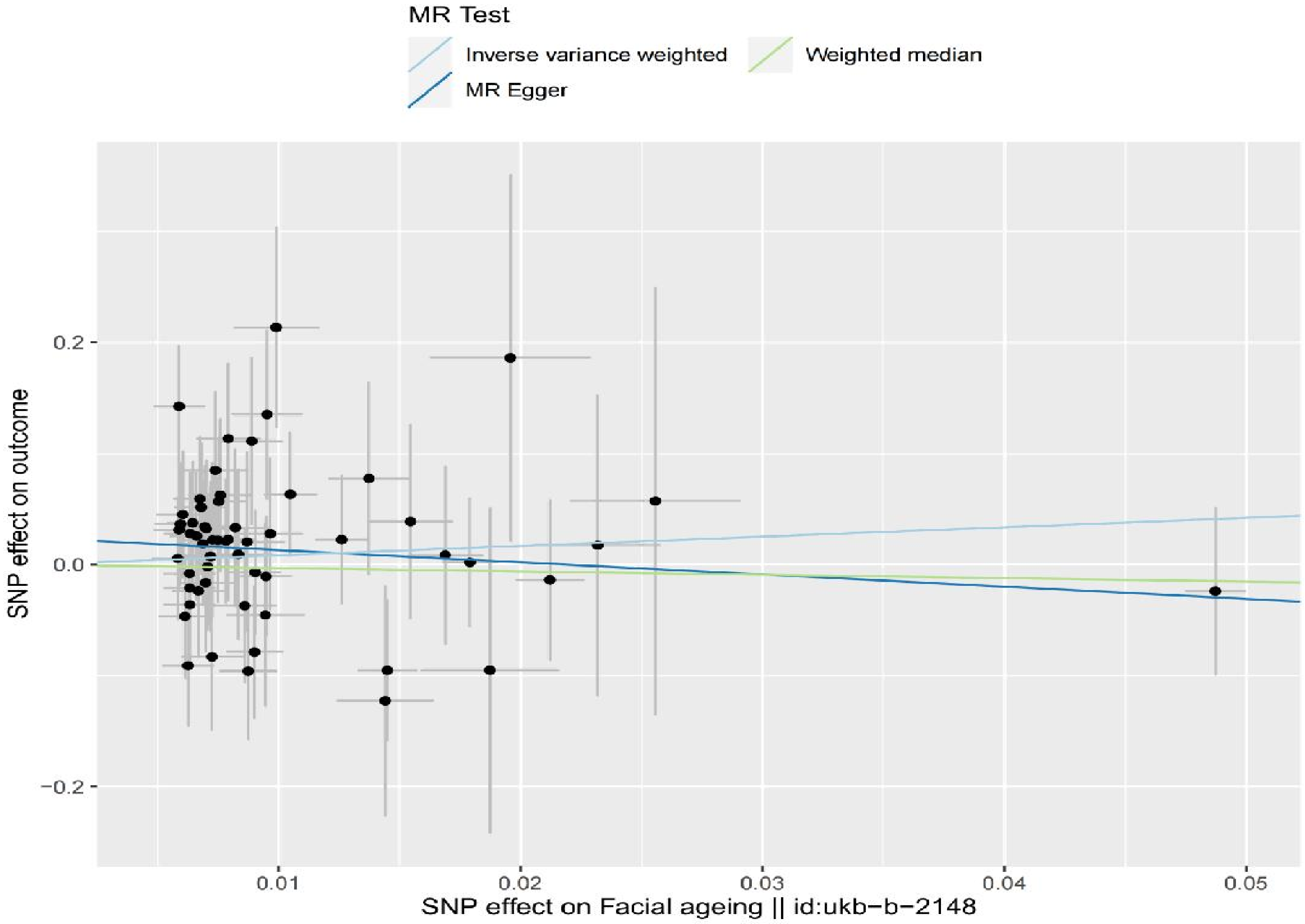

Supplementary Figure-20C Forest Plot

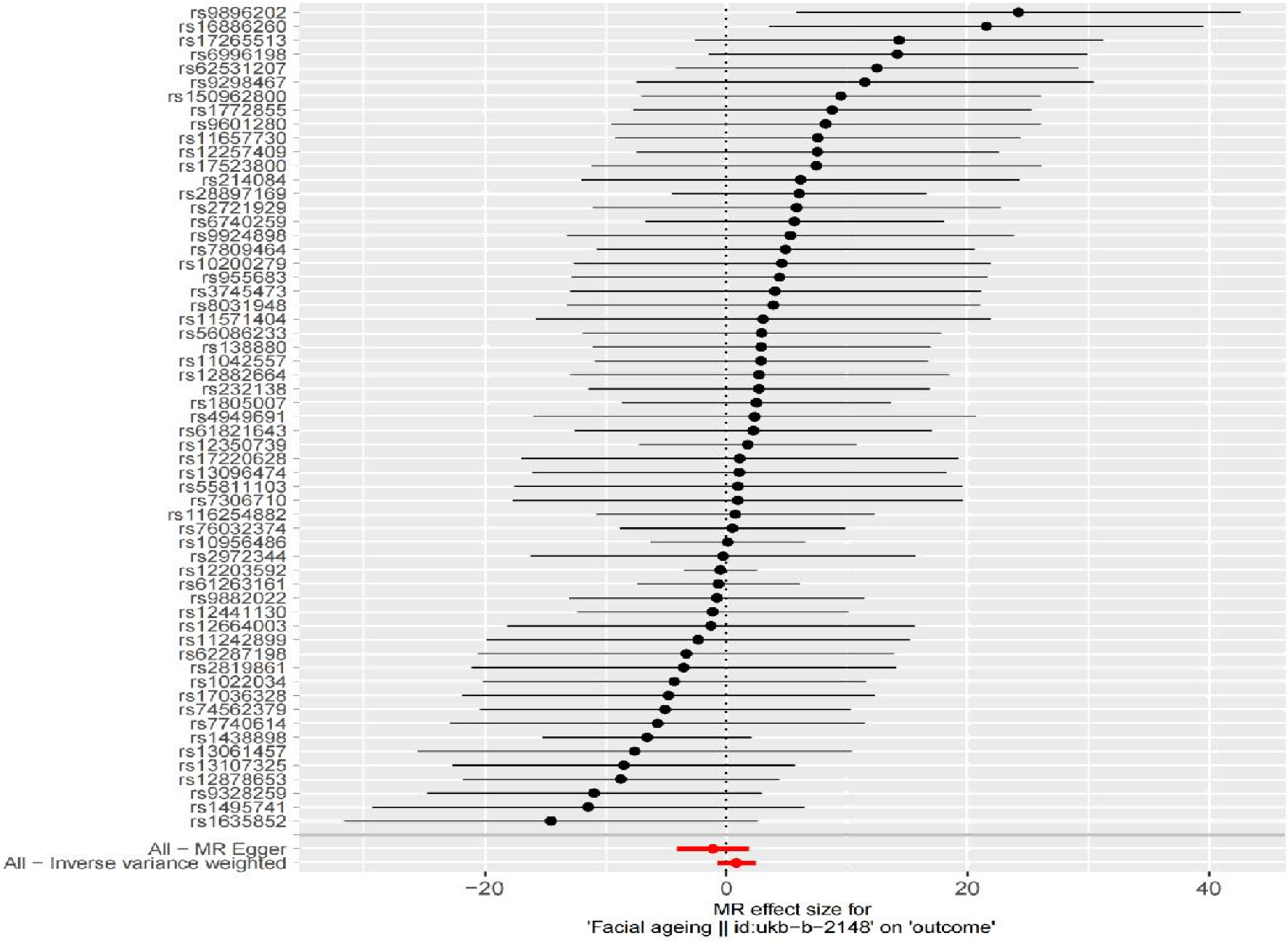

Supplementary Figure-20D Funnel Plot

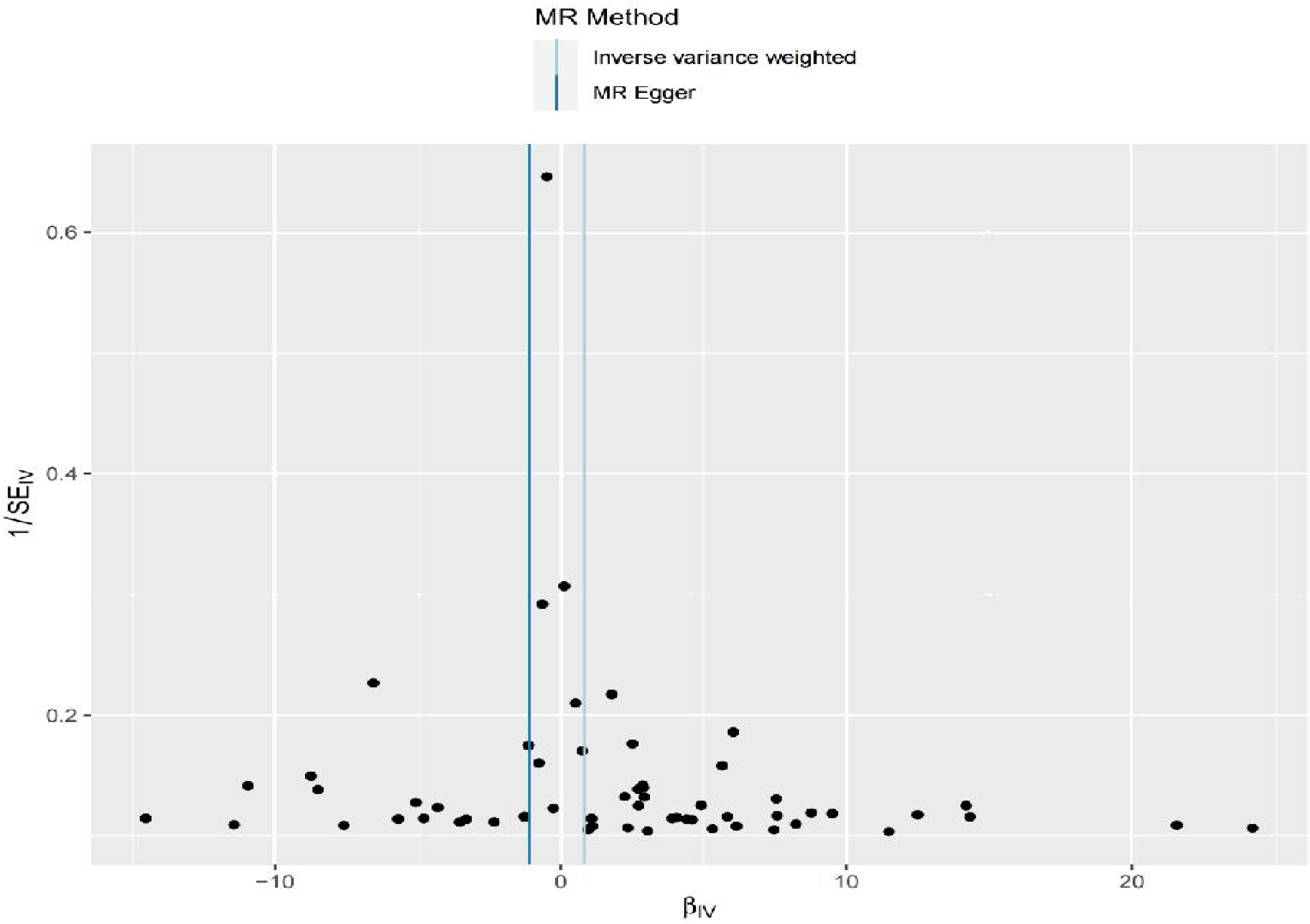

Supplementary Figure-21 Leave-one-out Analysis, Scatter Plot, Forest Plot, and Funnel Plot of Frailty Index on Alzheimer's Disease

Supplementary Figure-21A Leave-one-out Analysis

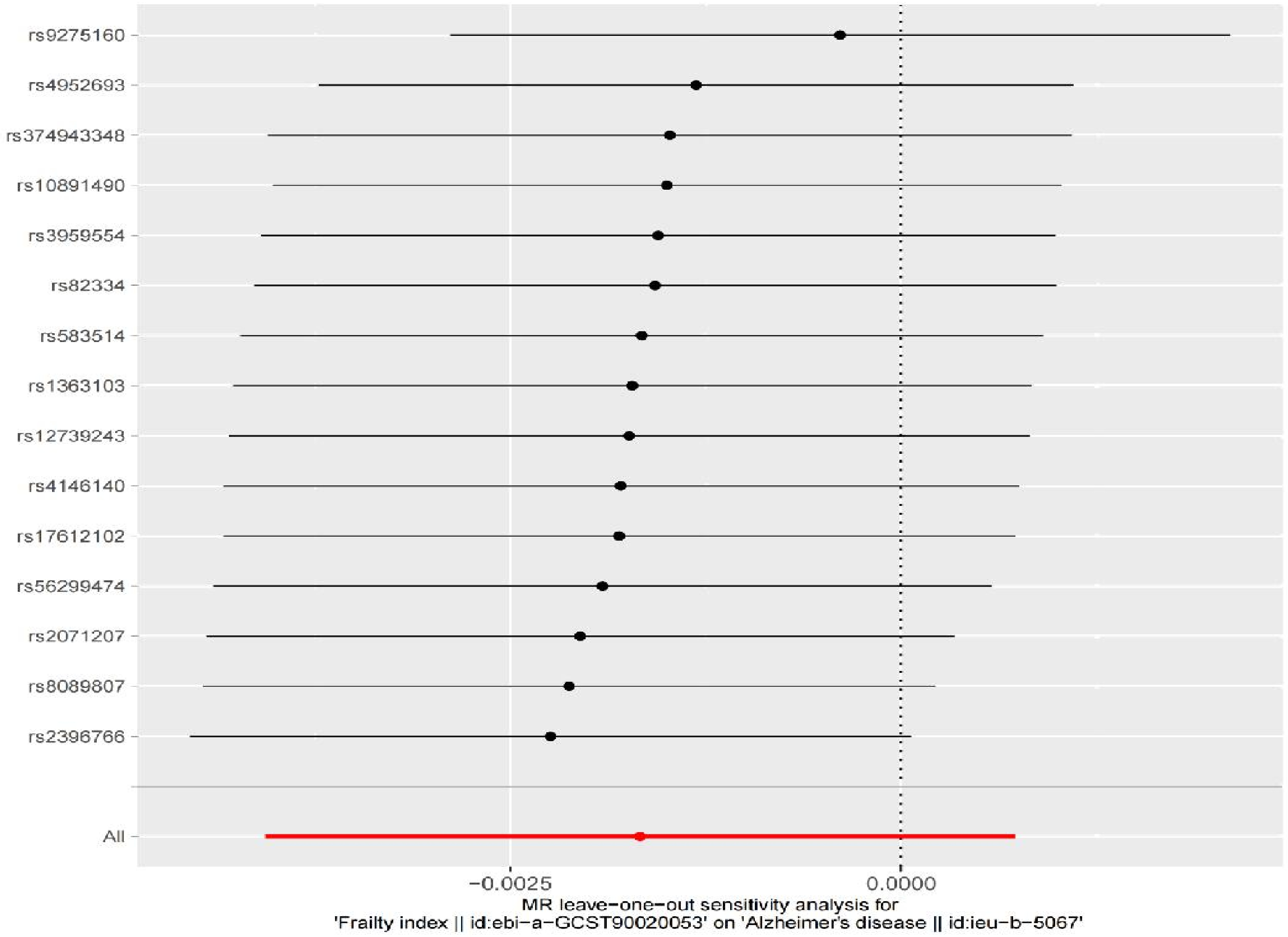

Supplementary Figure-21B Scatter

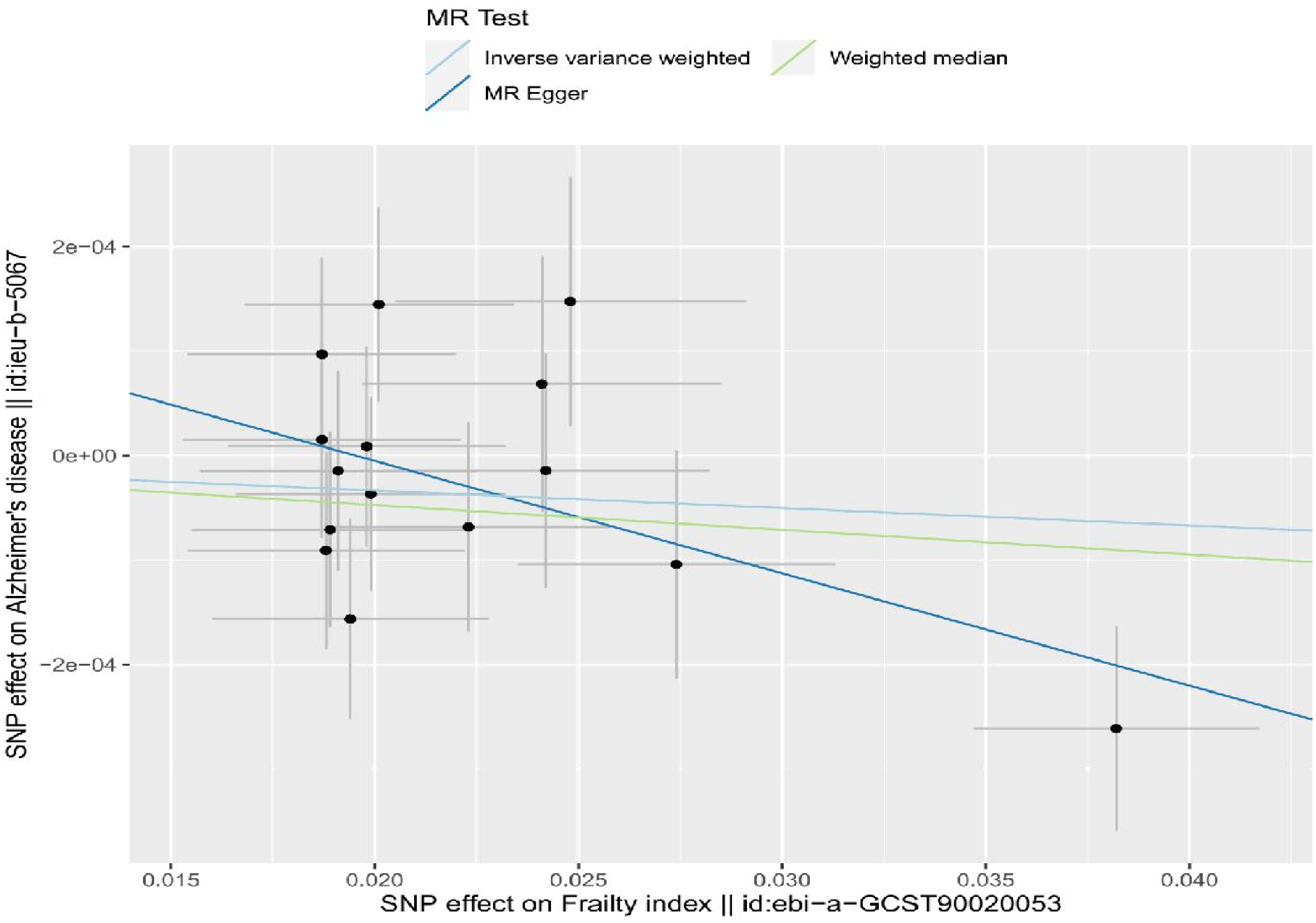

Supplementary Figure-21C Forest Plot

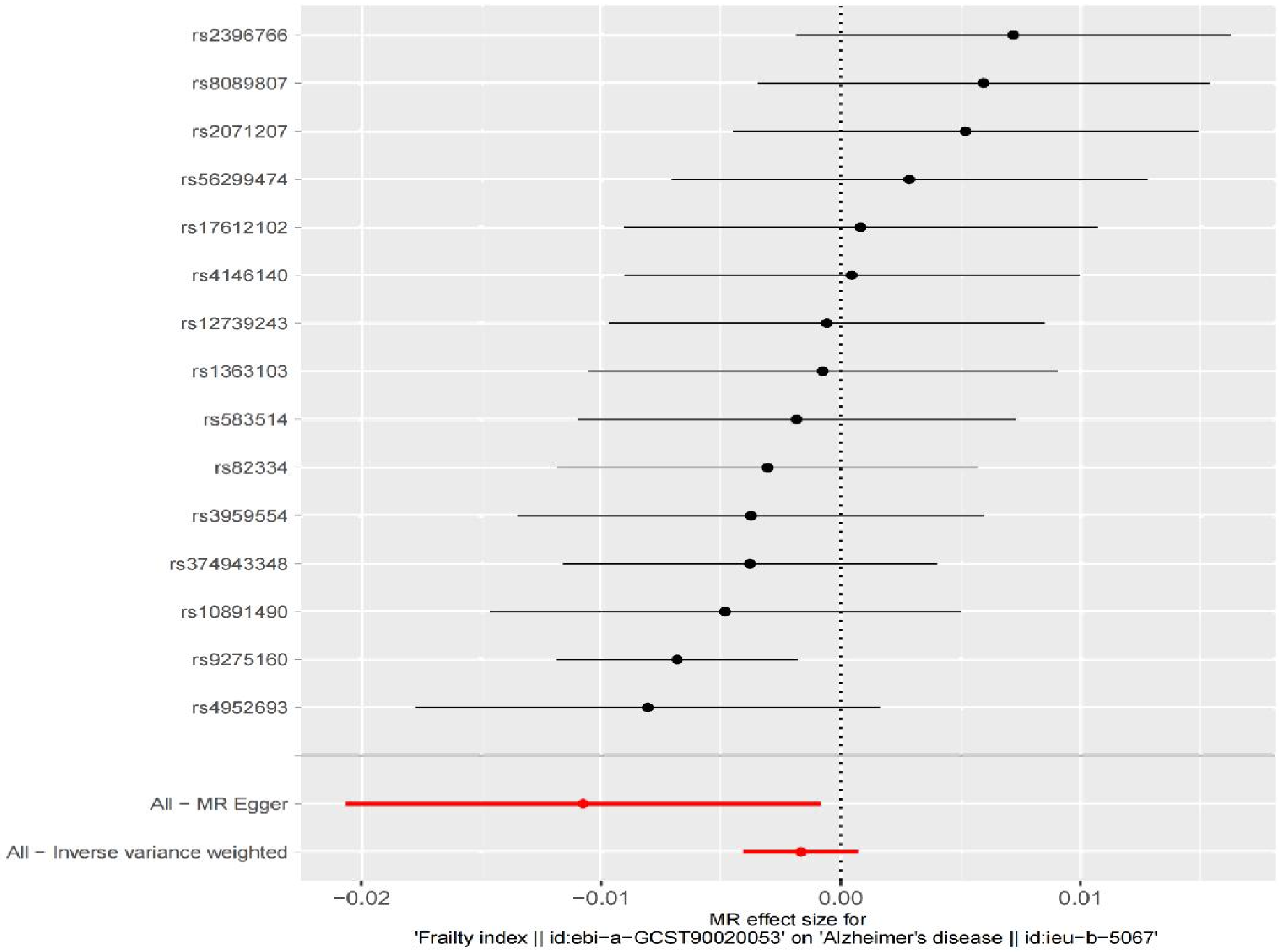

Supplementary Figure-21D Funnel Plot

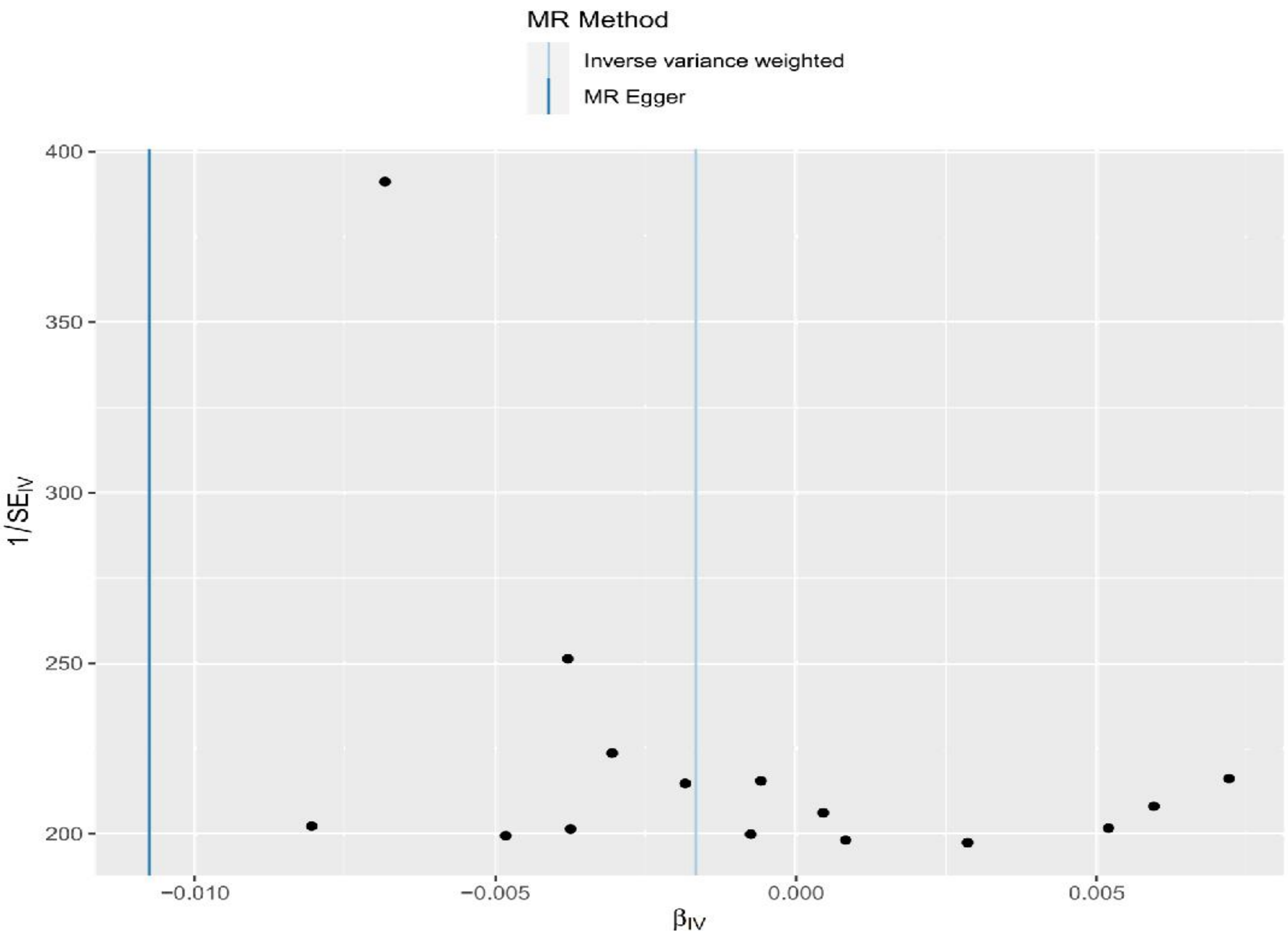

Supplementary Figure-22 Leave-one-out Analysis, Scatter Plot, Forest Plot, and Funnel Plot of Frailty Index on All Glioma

Supplementary Figure-22A Leave-one-out Analysis

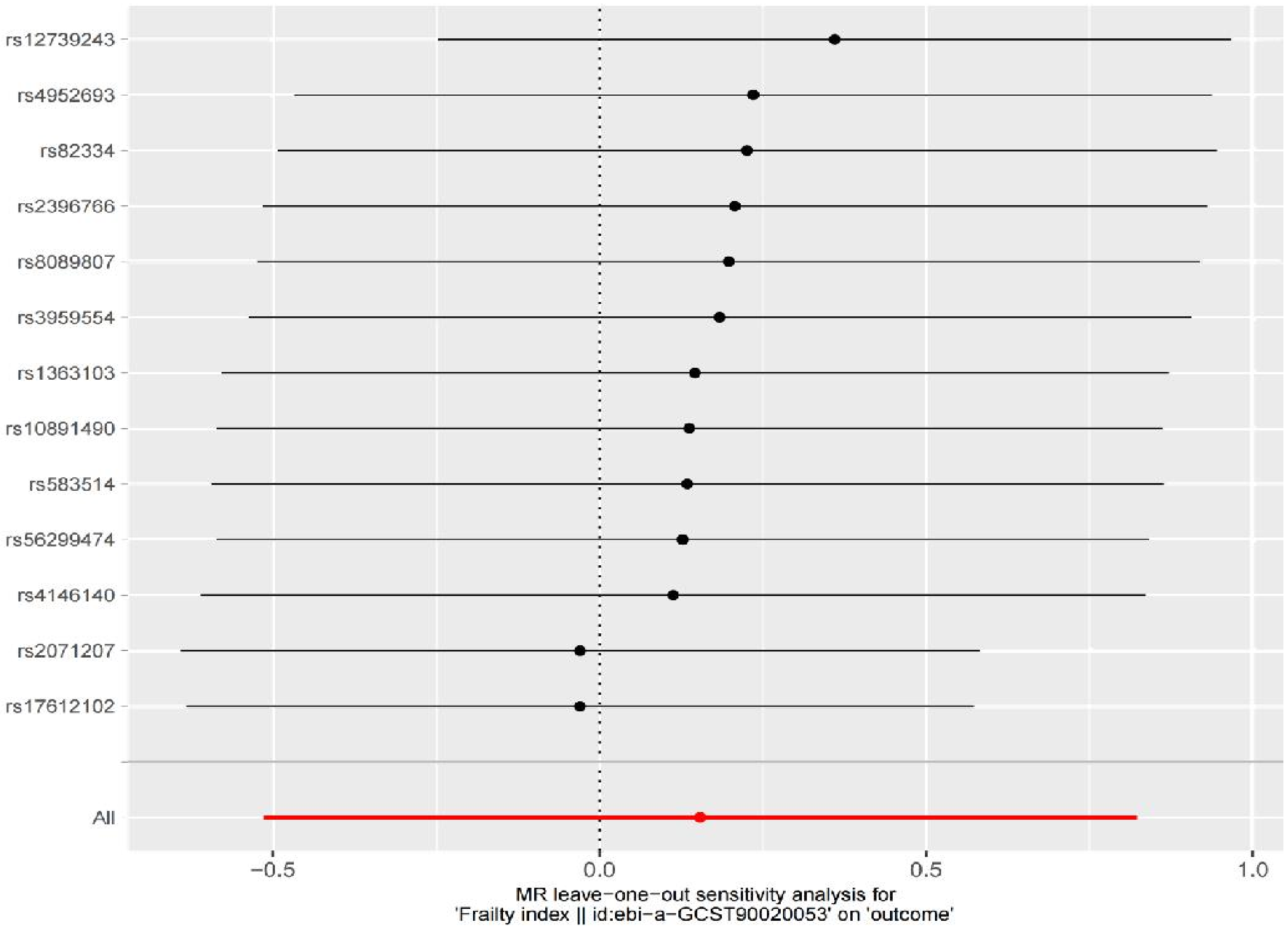

Supplementary Figure-22B Scatter

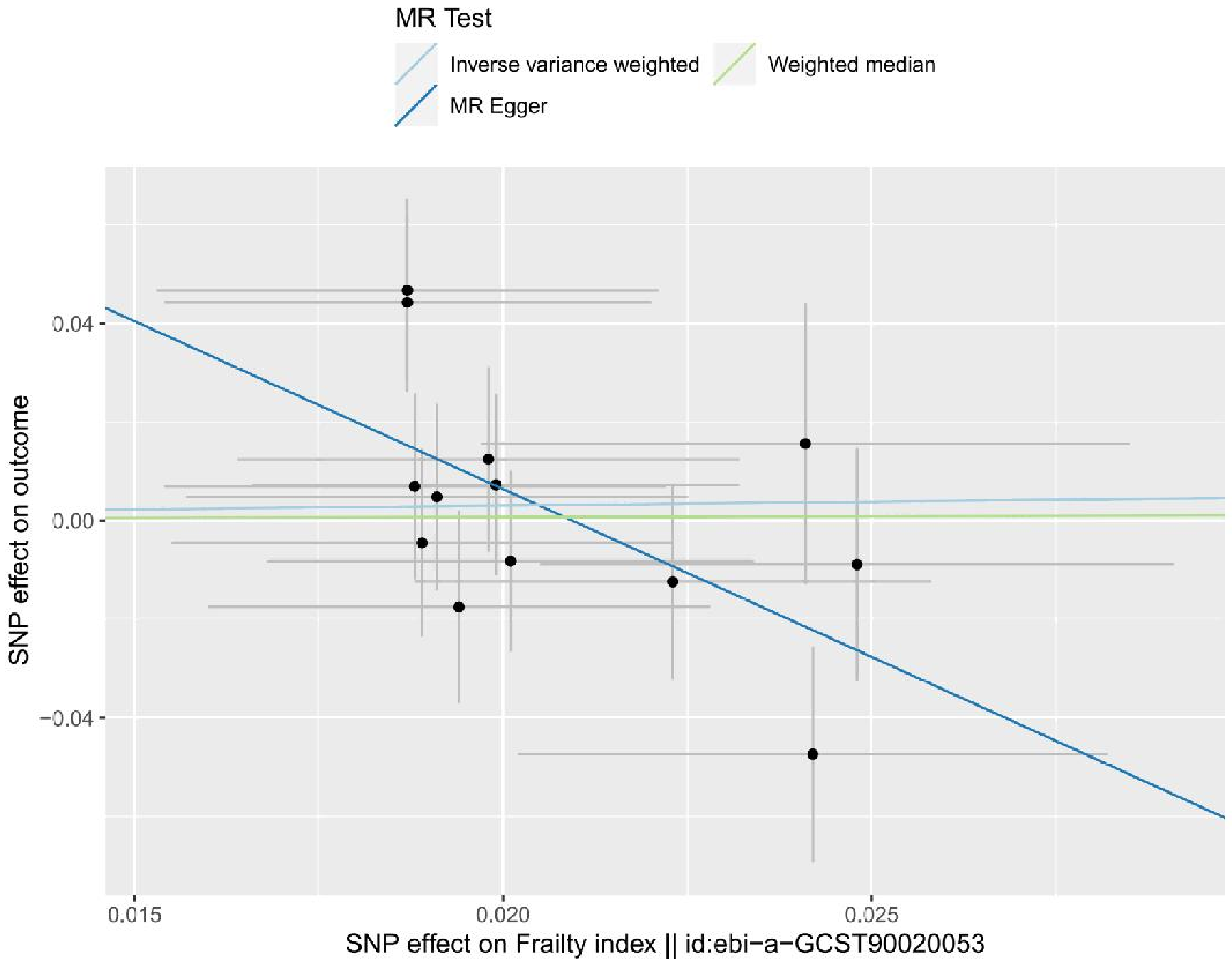

Supplementary Figure-22C Forest Plot

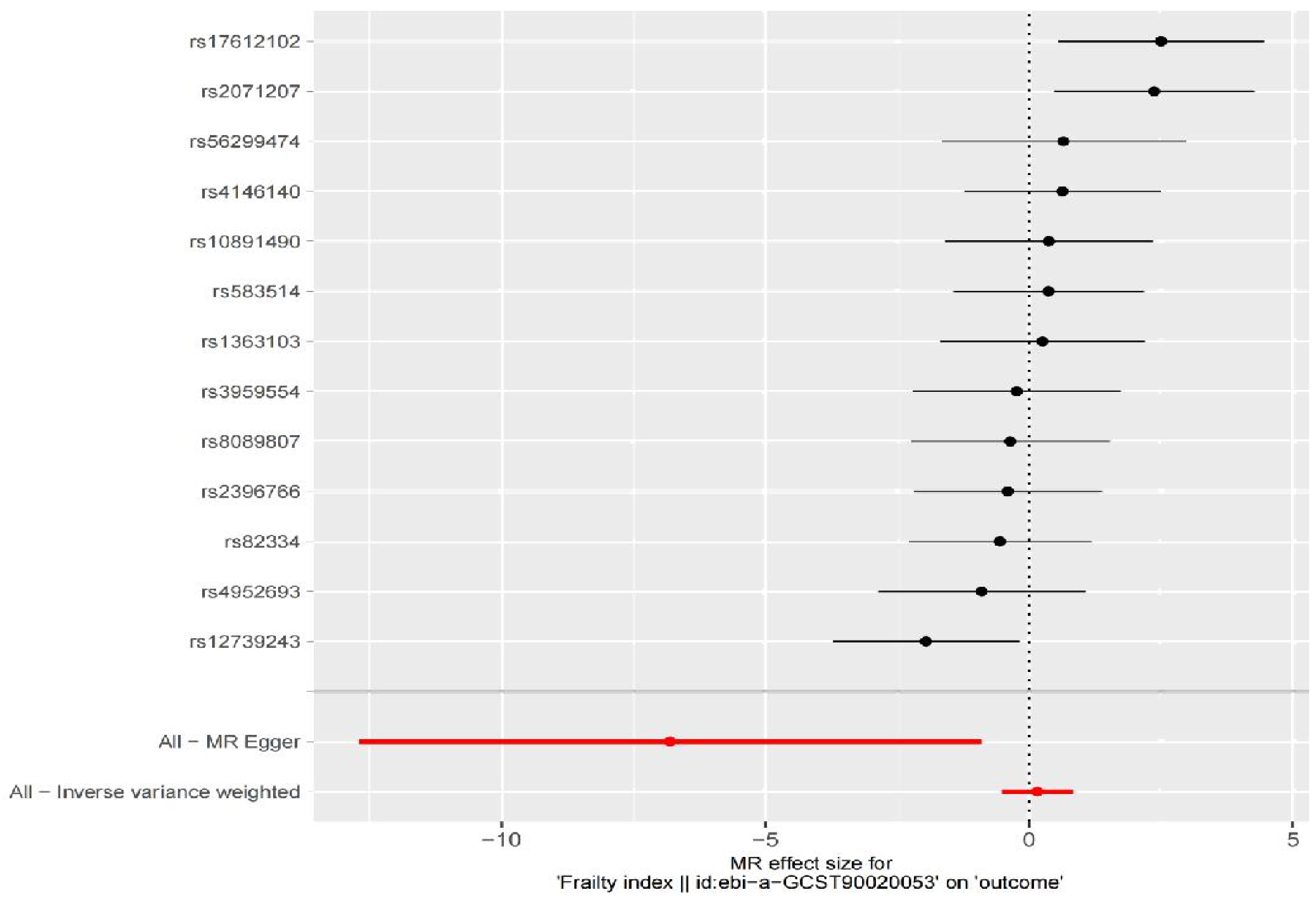

Supplementary Figure-22D Funnel Plot

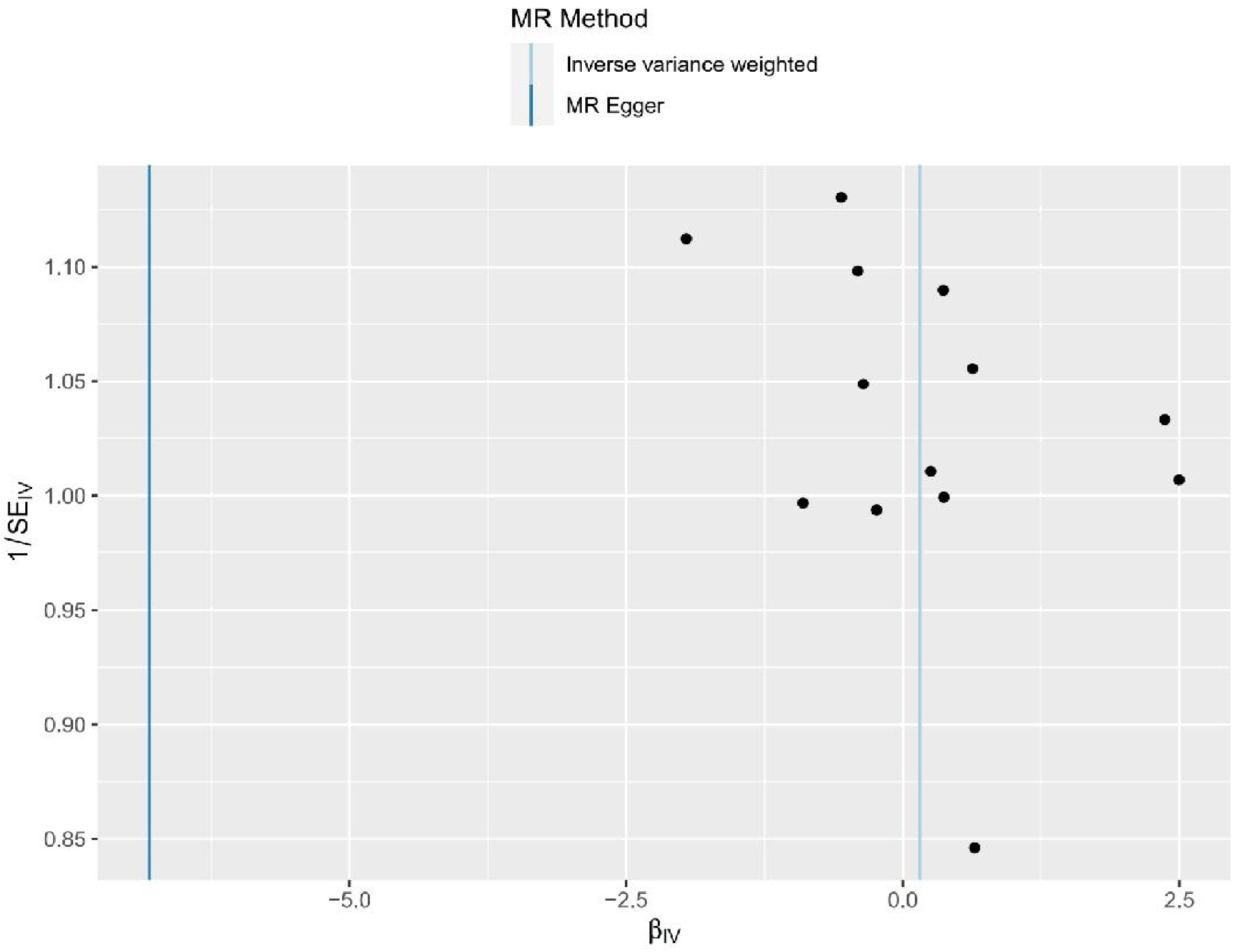

Supplementary Figure-23 Leave-one-out Analysis, Scatter Plot, Forest Plot, and Funnel Plot of Frailty Index on Amyotrophic Lateral Sclerosis

Supplementary Figure-23A Leave-one-out Analysis

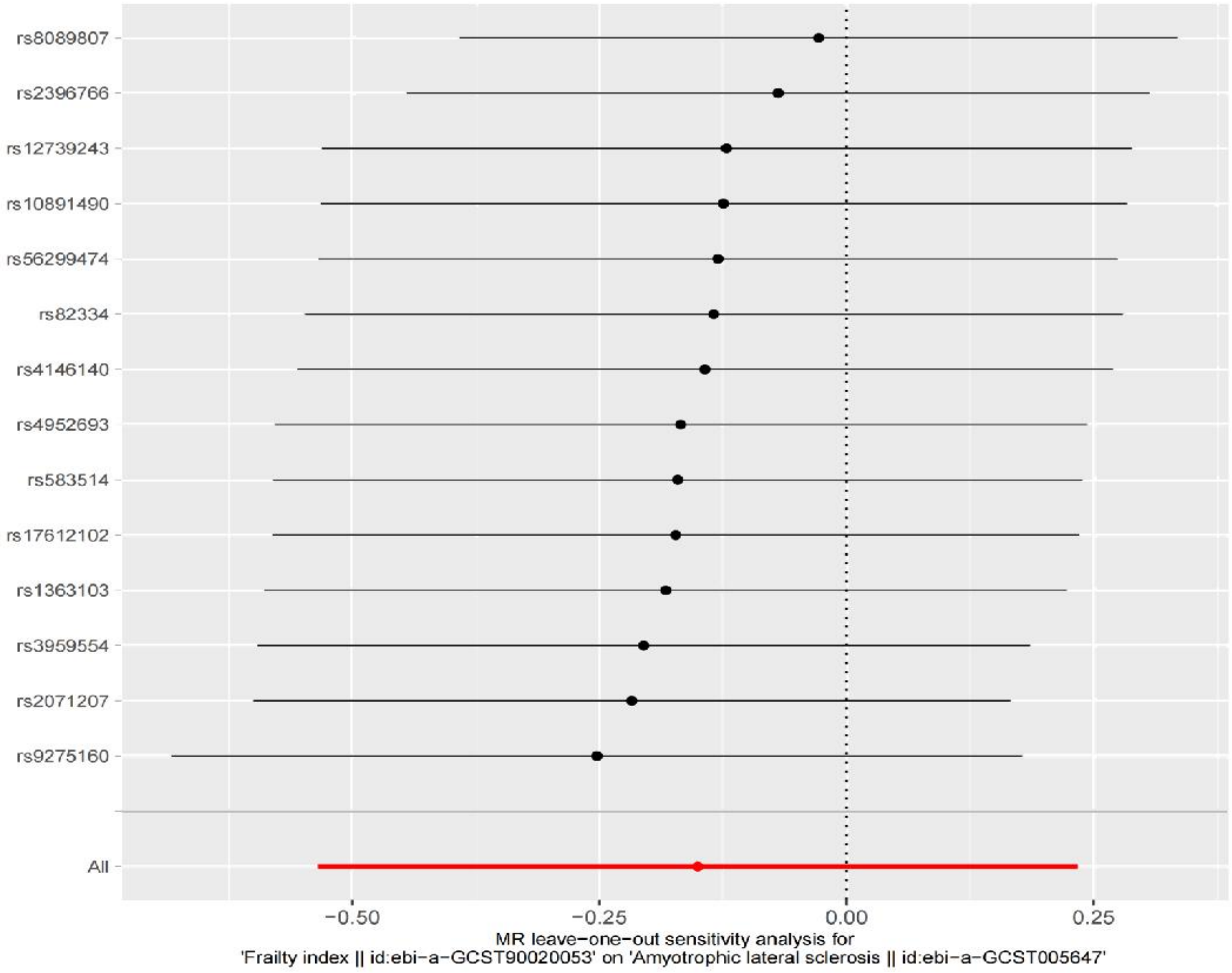

Supplementary Figure-23B Scatter

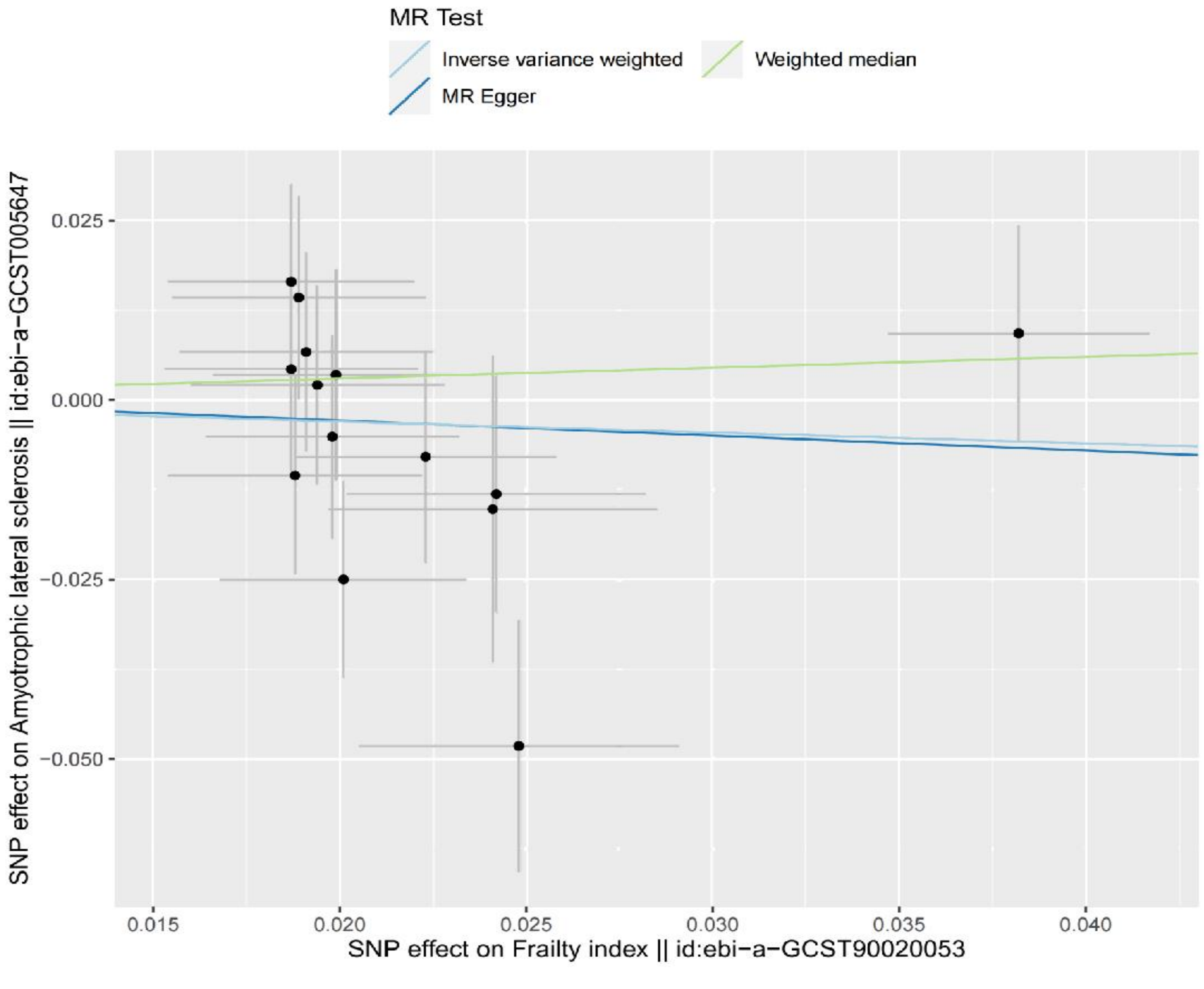

Supplementary Figure-23C Forest Plot

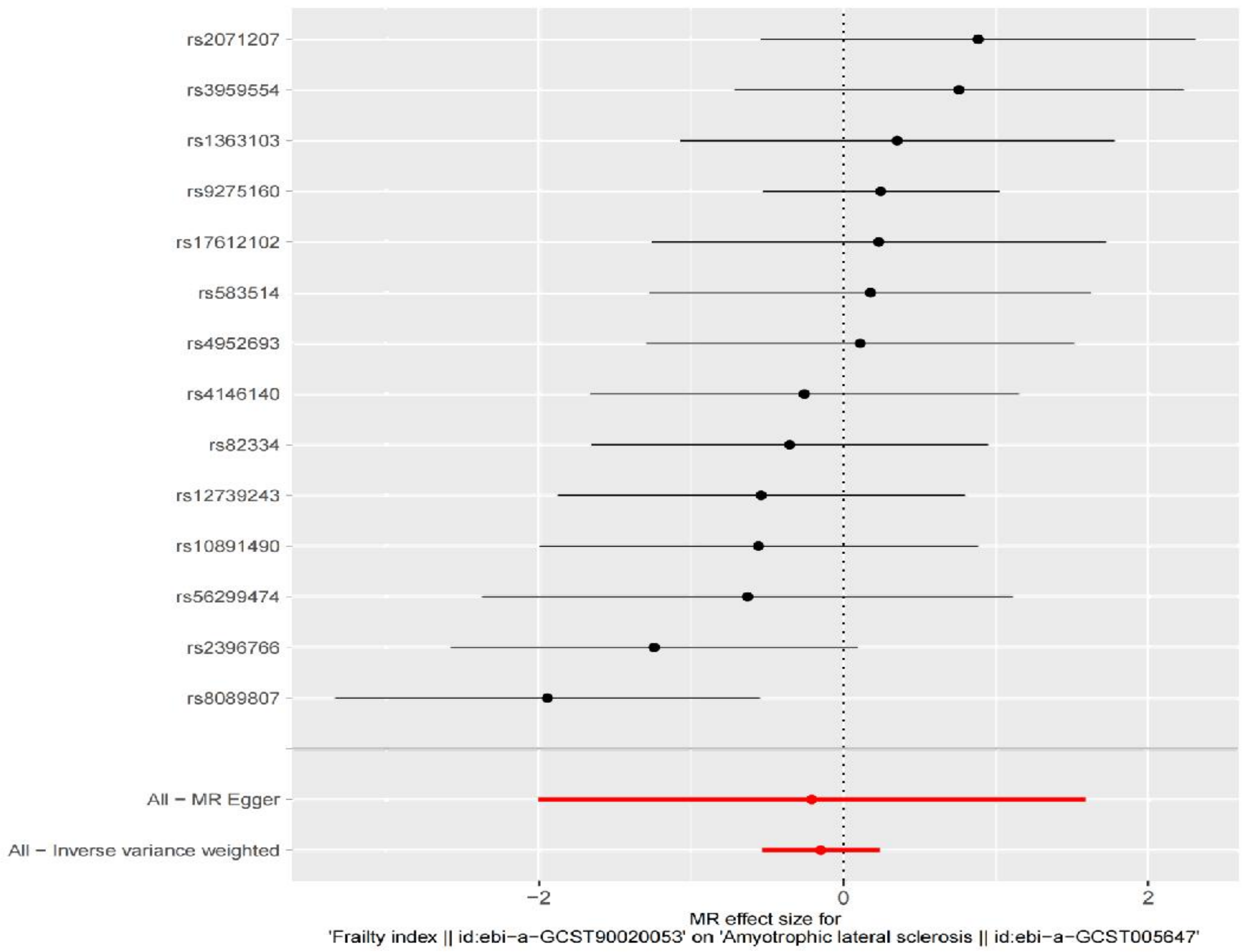

Supplementary Figure-23D Funnel Plot

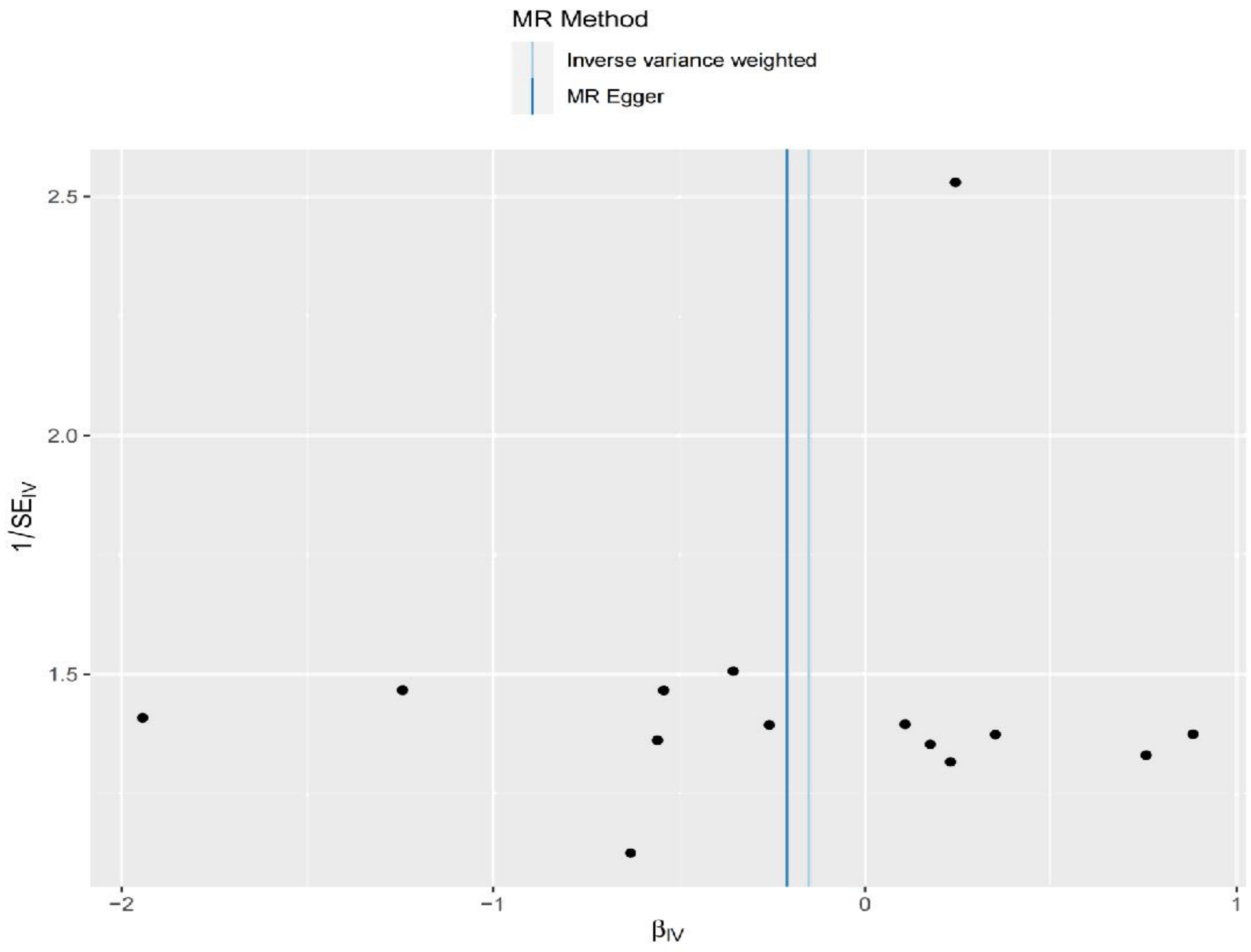

Supplementary Figure-24 Leave-one-out Analysis, Scatter Plot, Forest Plot, and Funnel Plot of Frailty Index on GBM

Supplementary Figure-24A Leave-one-out Analysis

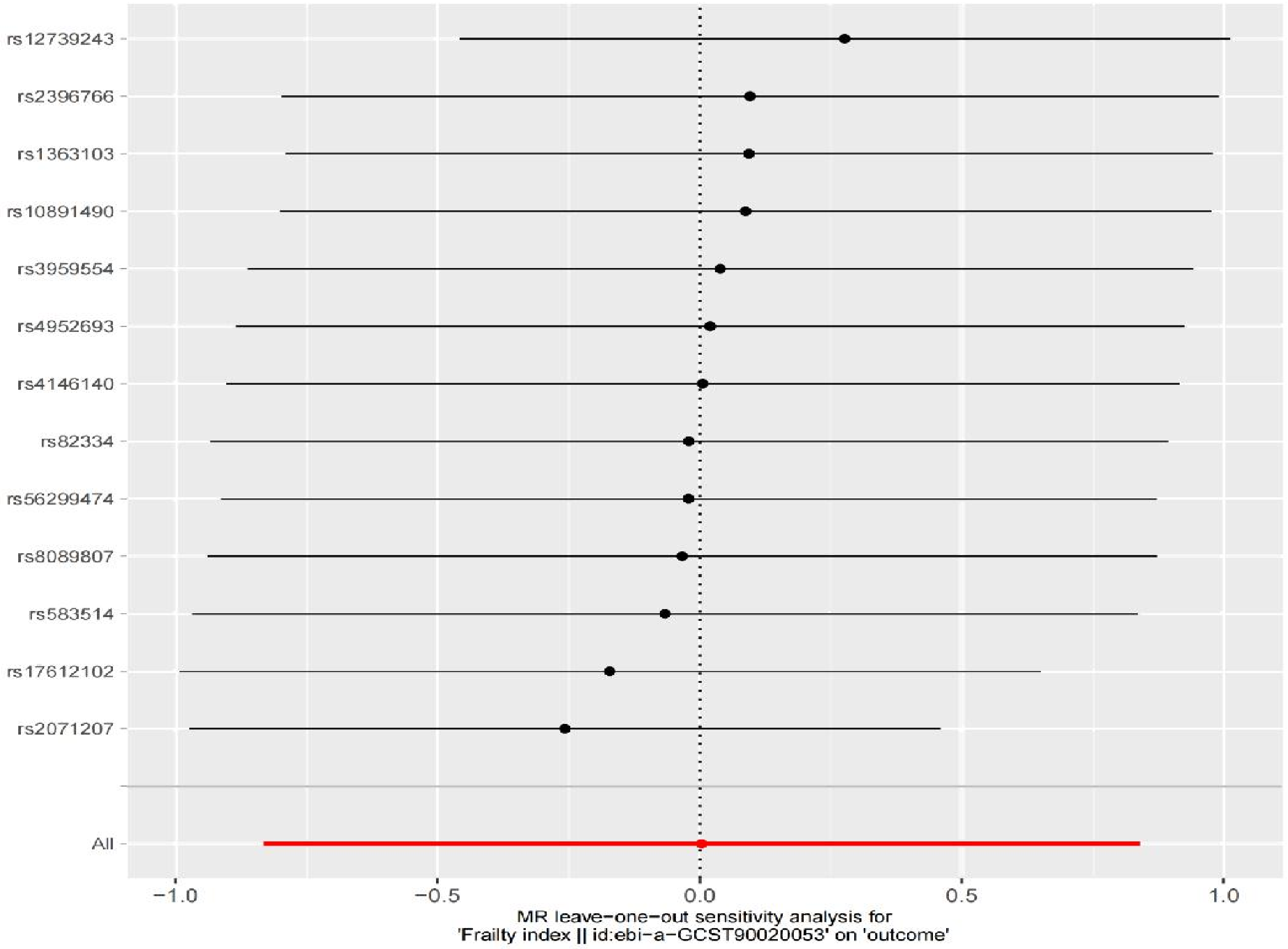

Supplementary Figure-24B Scatter

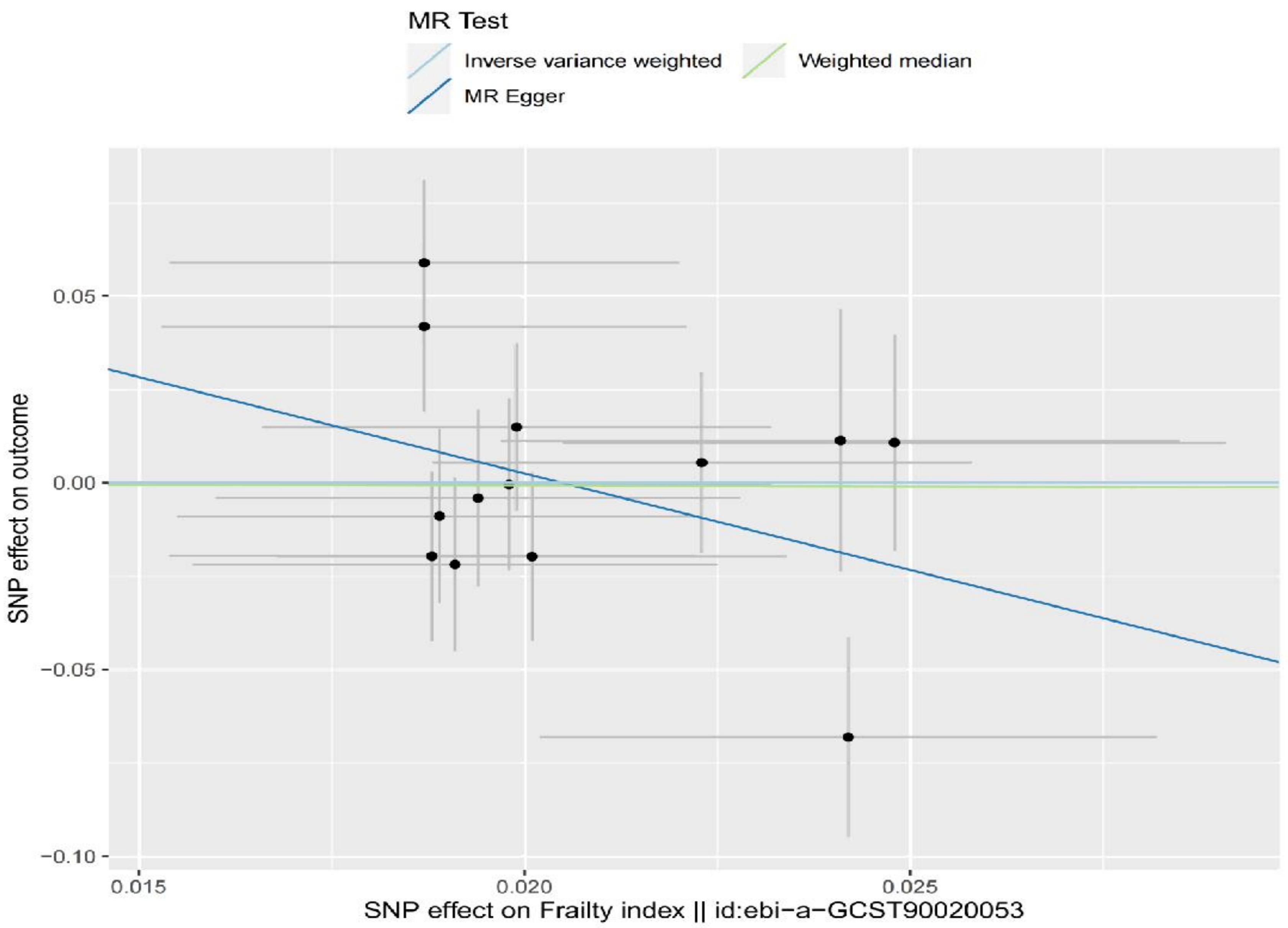

Supplementary Figure-24C Forest Plot

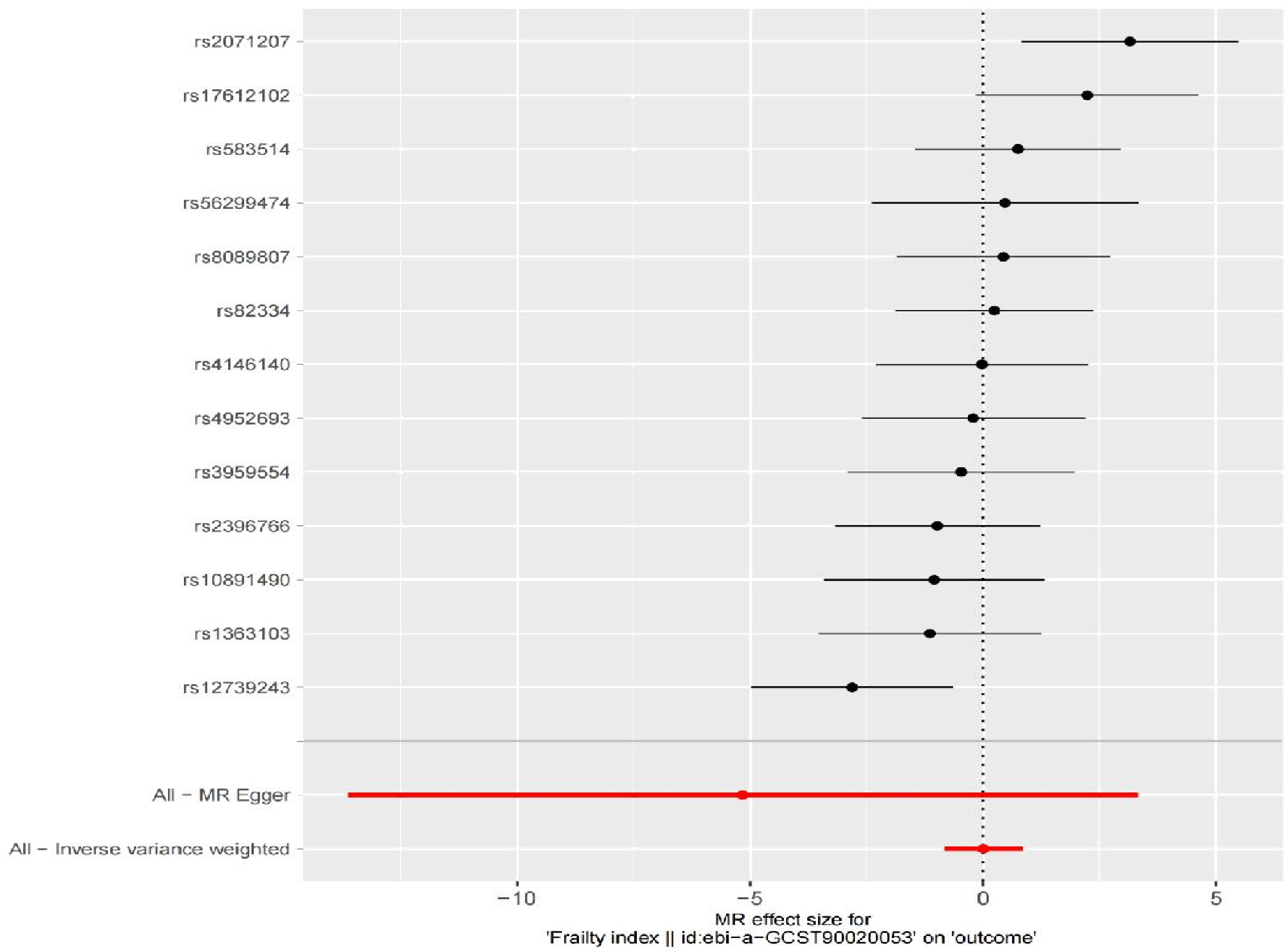

Supplementary Figure-24D Funnel Plot

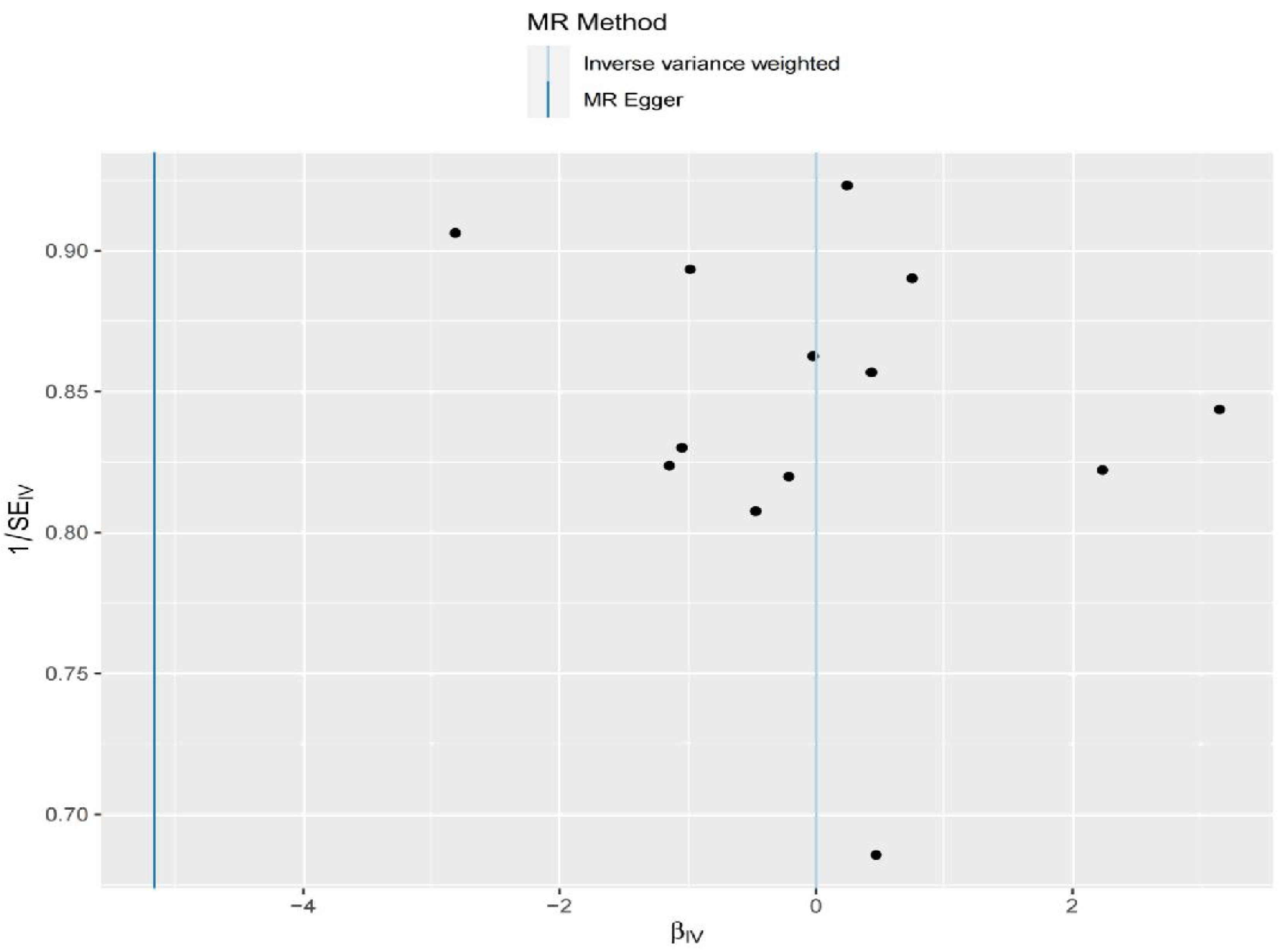

Supplementary Figure-25 Leave-one-out Analysis, Scatter Plot, Forest Plot, and Funnel Plot of Frailty Index on Meningioma

Supplementary Figure-25A Leave-one-out Analysis

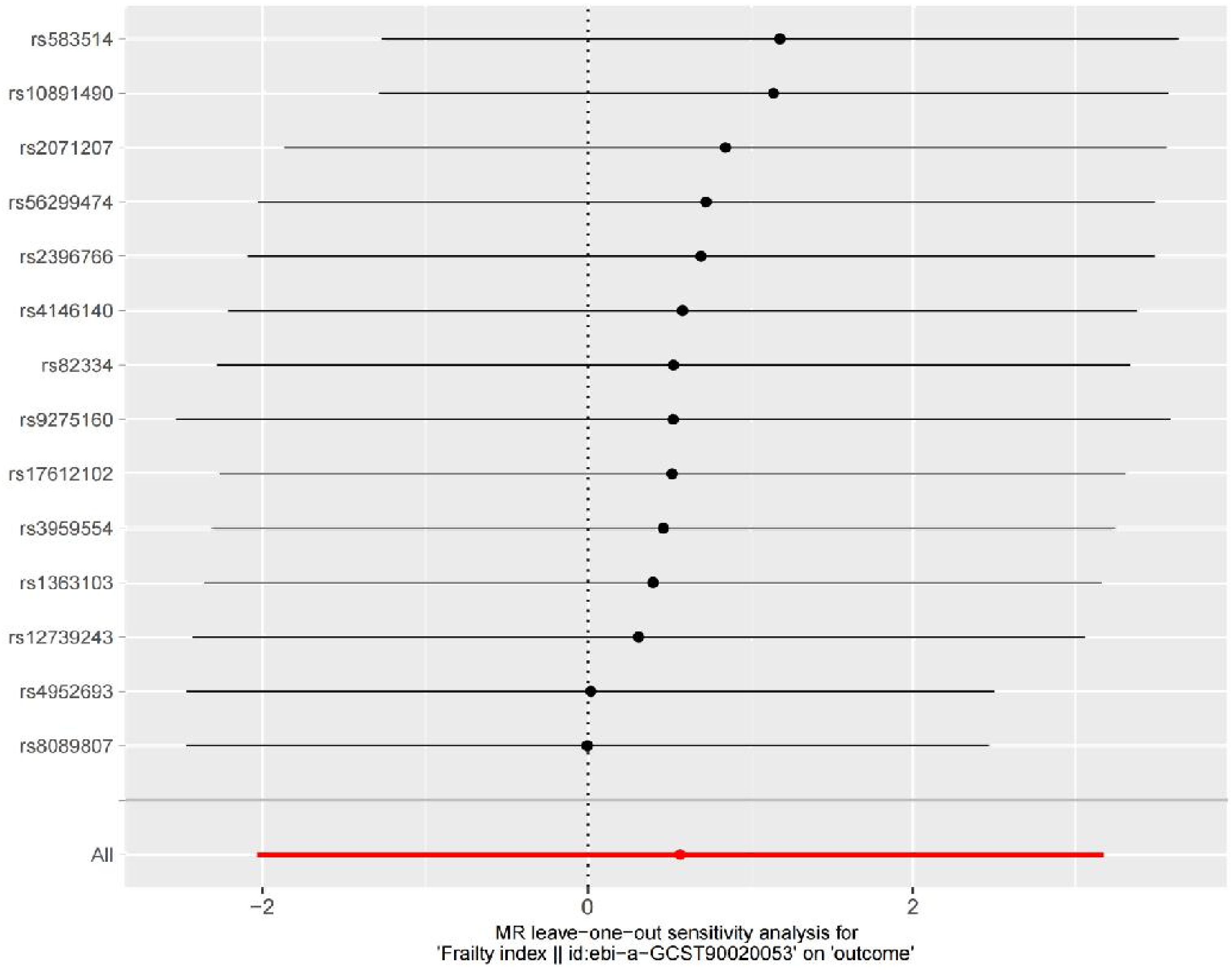

Supplementary Figure-25B Scatter

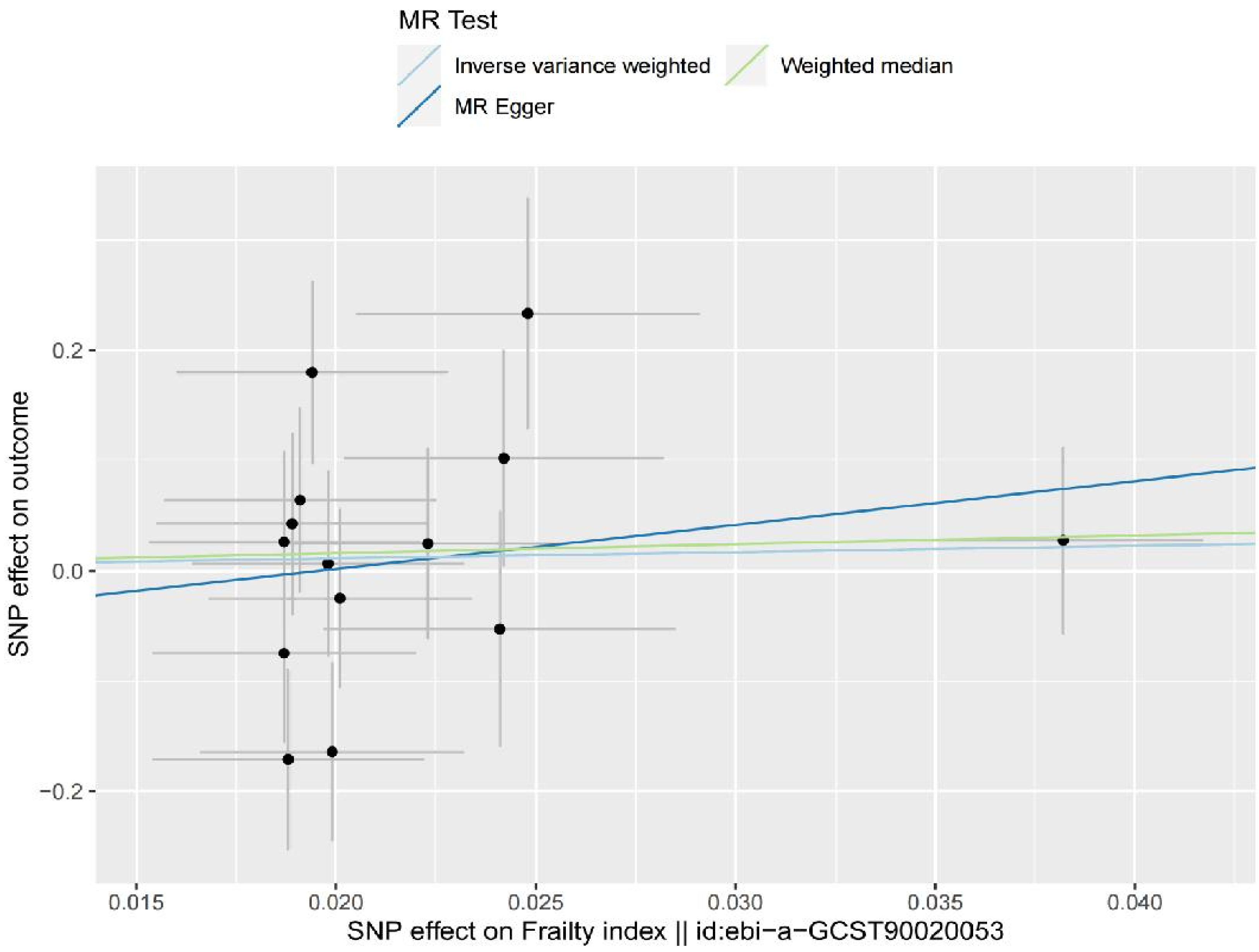

Supplementary Figure-25C Forest Plot

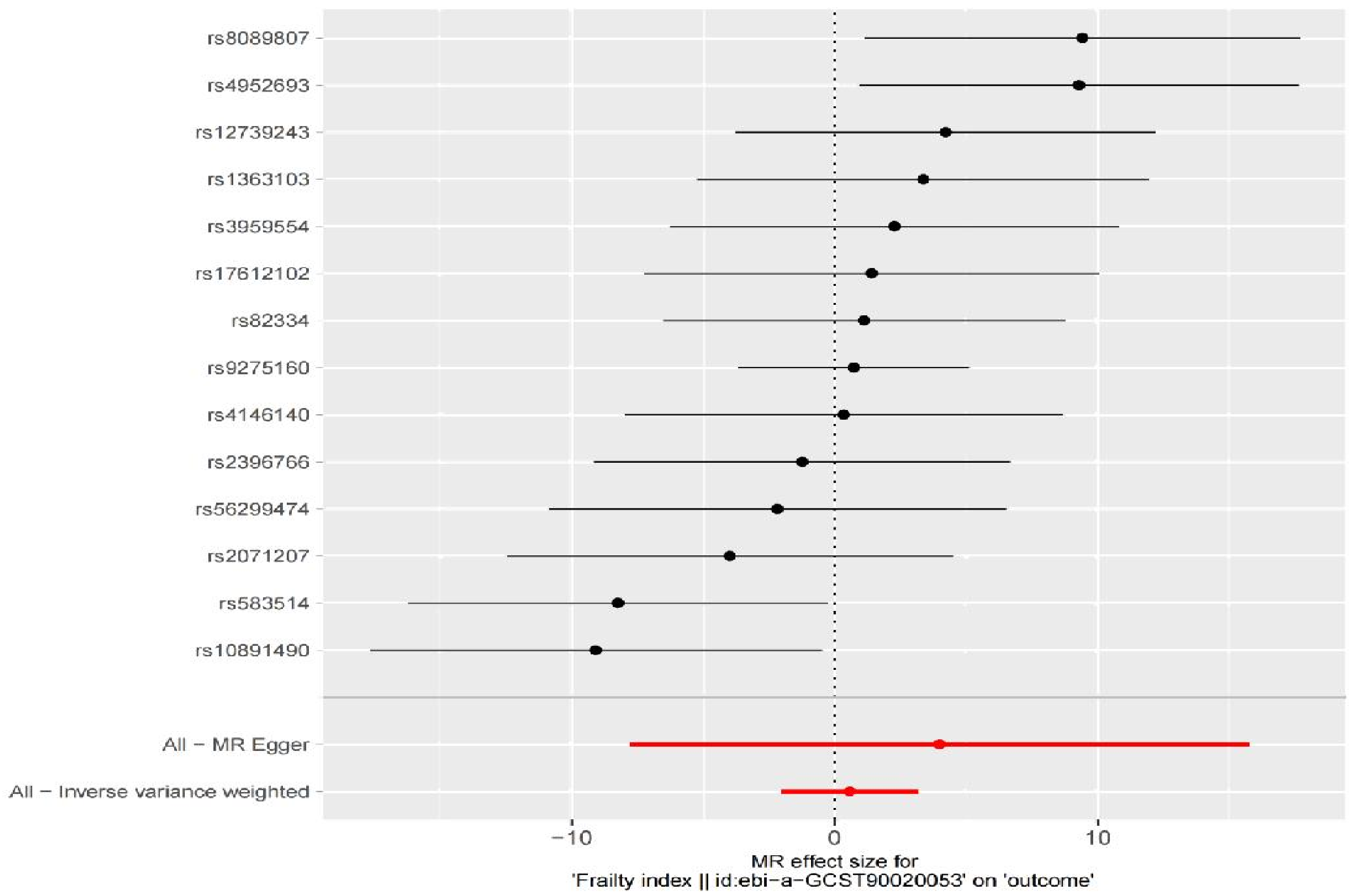

Supplementary Figure-25D Funnel Plot

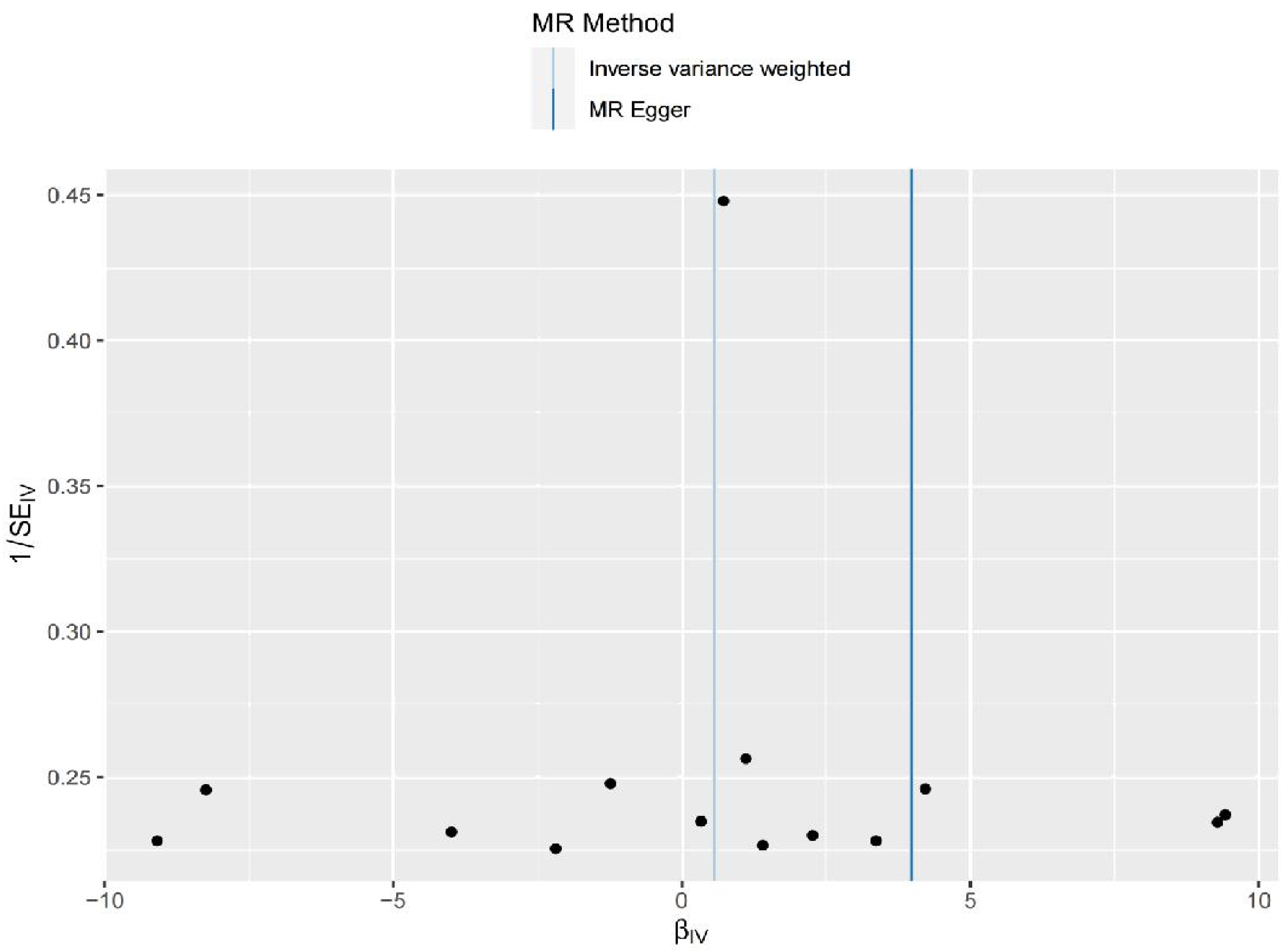

Supplementary Figure-26 Leave-one-out Analysis, Scatter Plot, Forest Plot, and Funnel Plot of Frailty Index on Non-GBM

Supplementary Figure-26A Leave-one-out Analysis

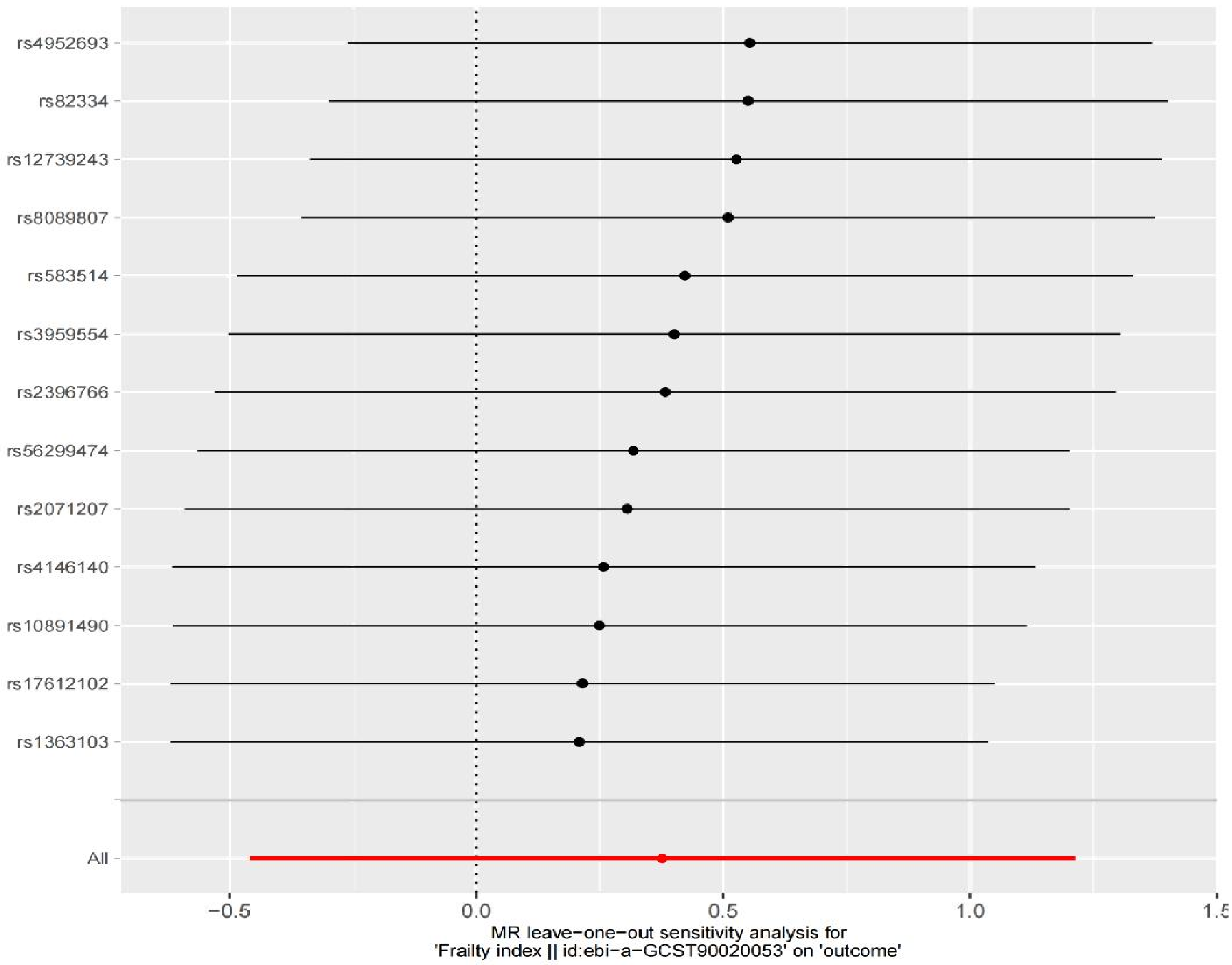

Supplementary Figure-26B Scatter

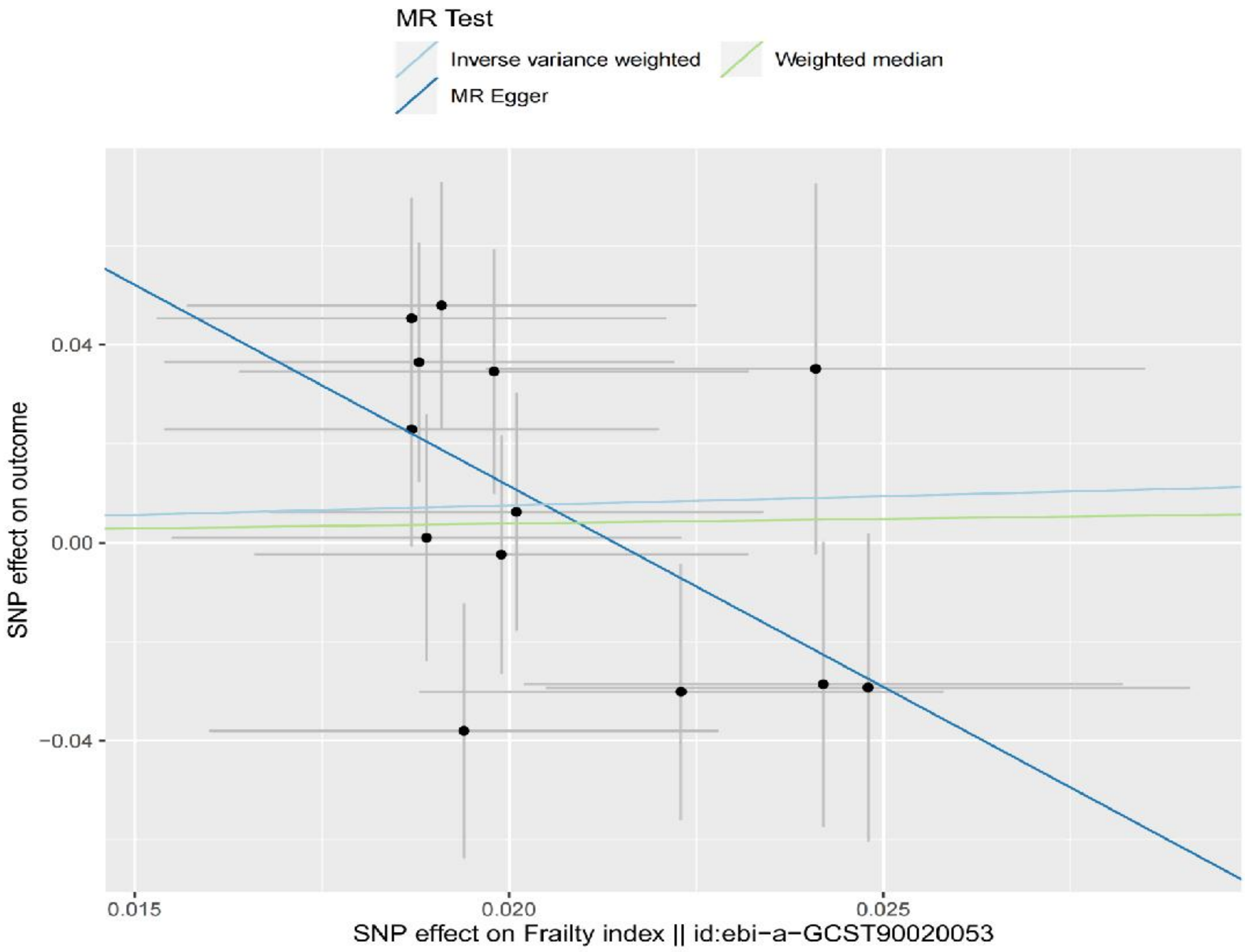

Supplementary Figure-26C Forest Plot

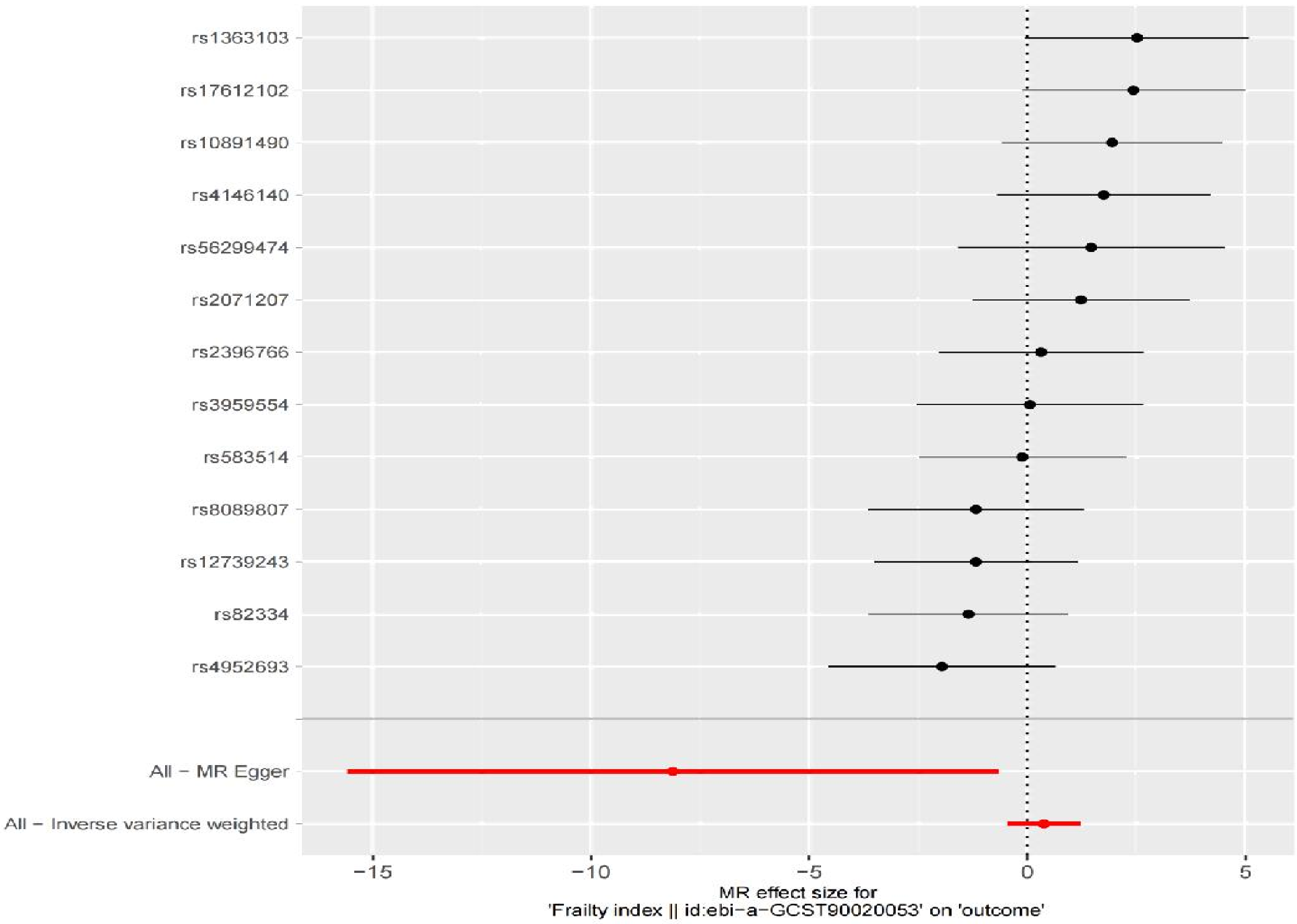

Supplementary Figure-26D Funnel Plot

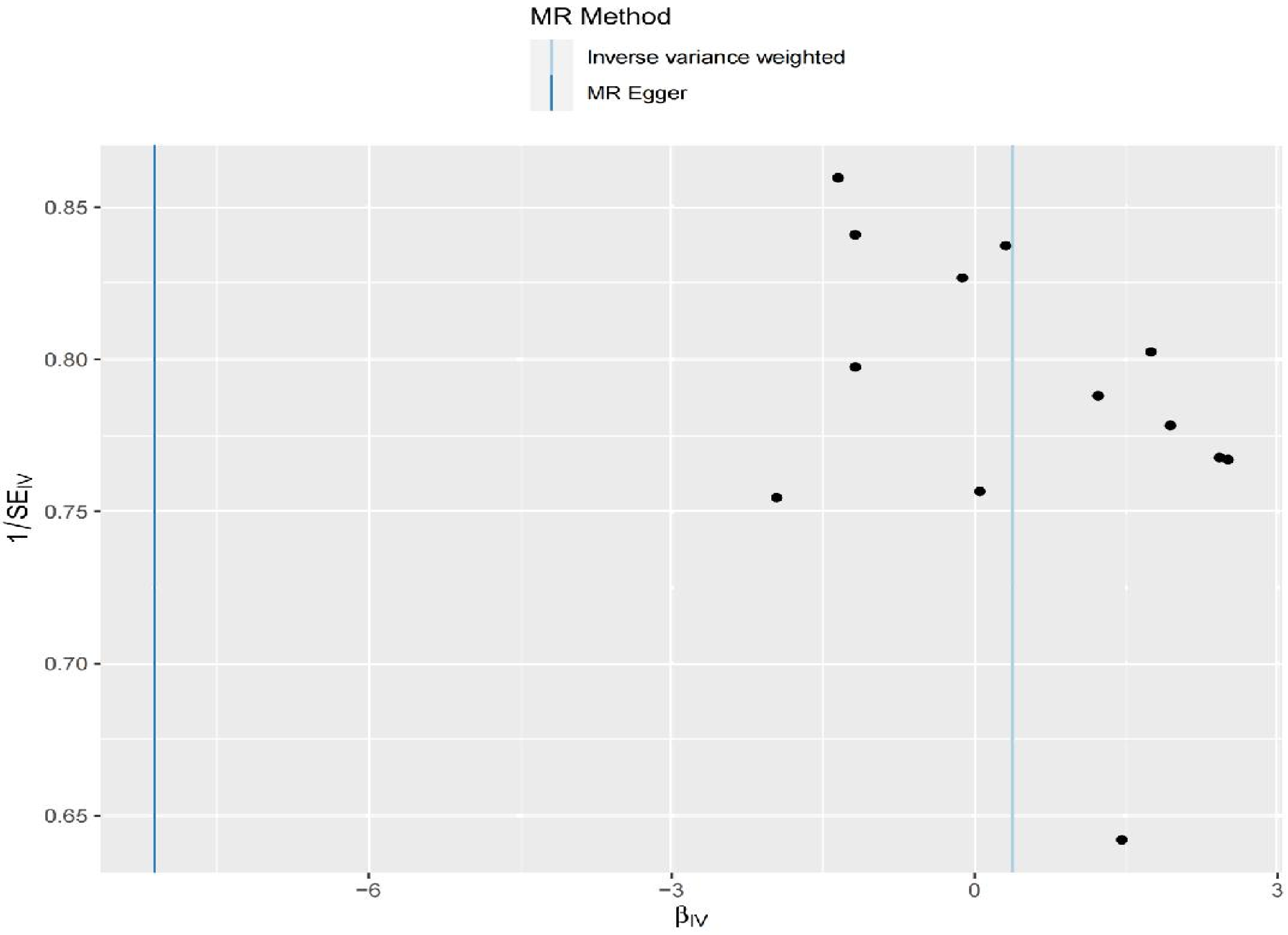

Supplementary Figure-27 Leave-one-out Analysis, Scatter Plot, Forest Plot, and Funnel Plot of Frailty Index on Parkinson

Supplementary Figure-27A Leave-one-out Analysis

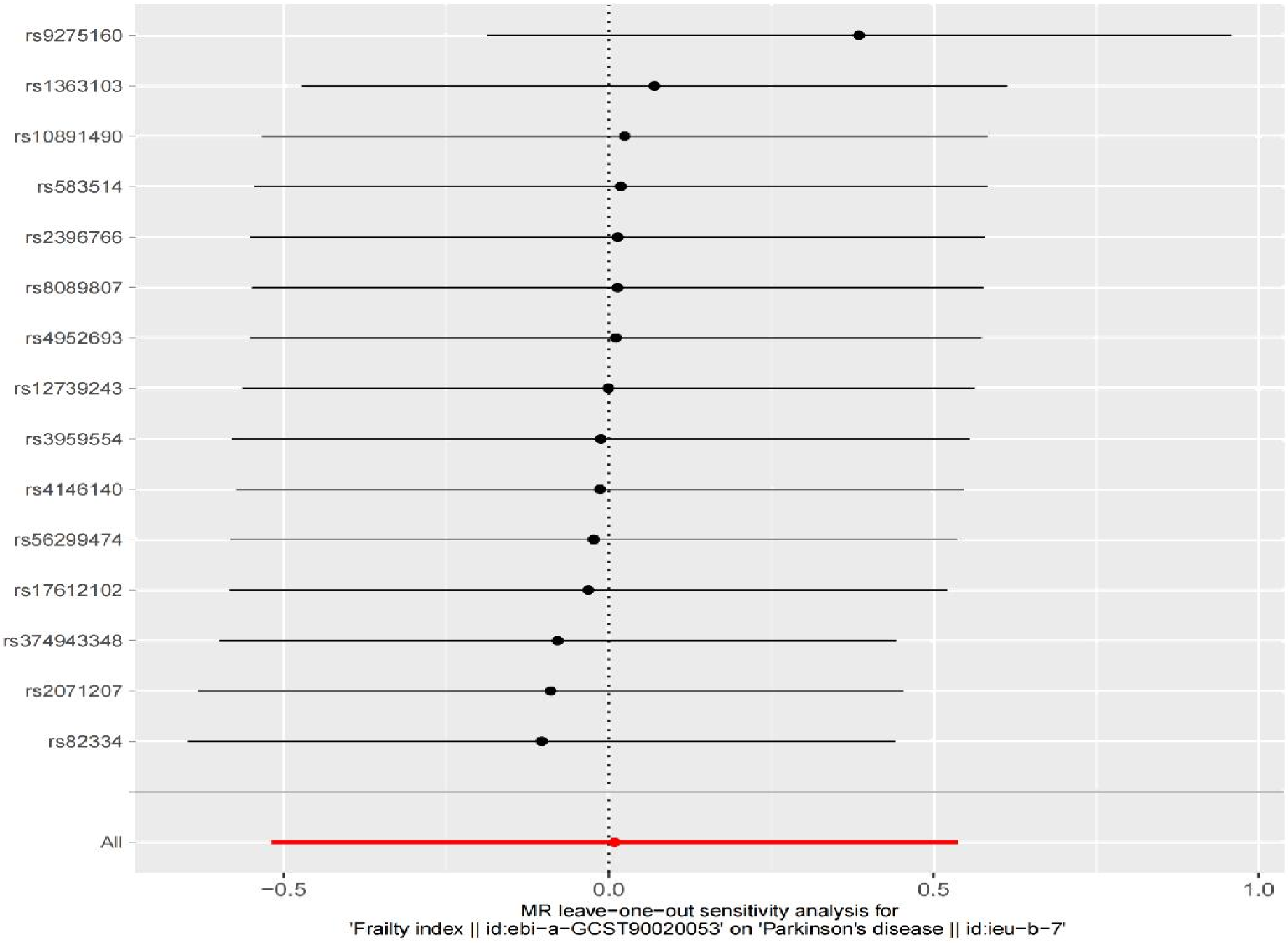

Supplementary Figure-27B Scatter

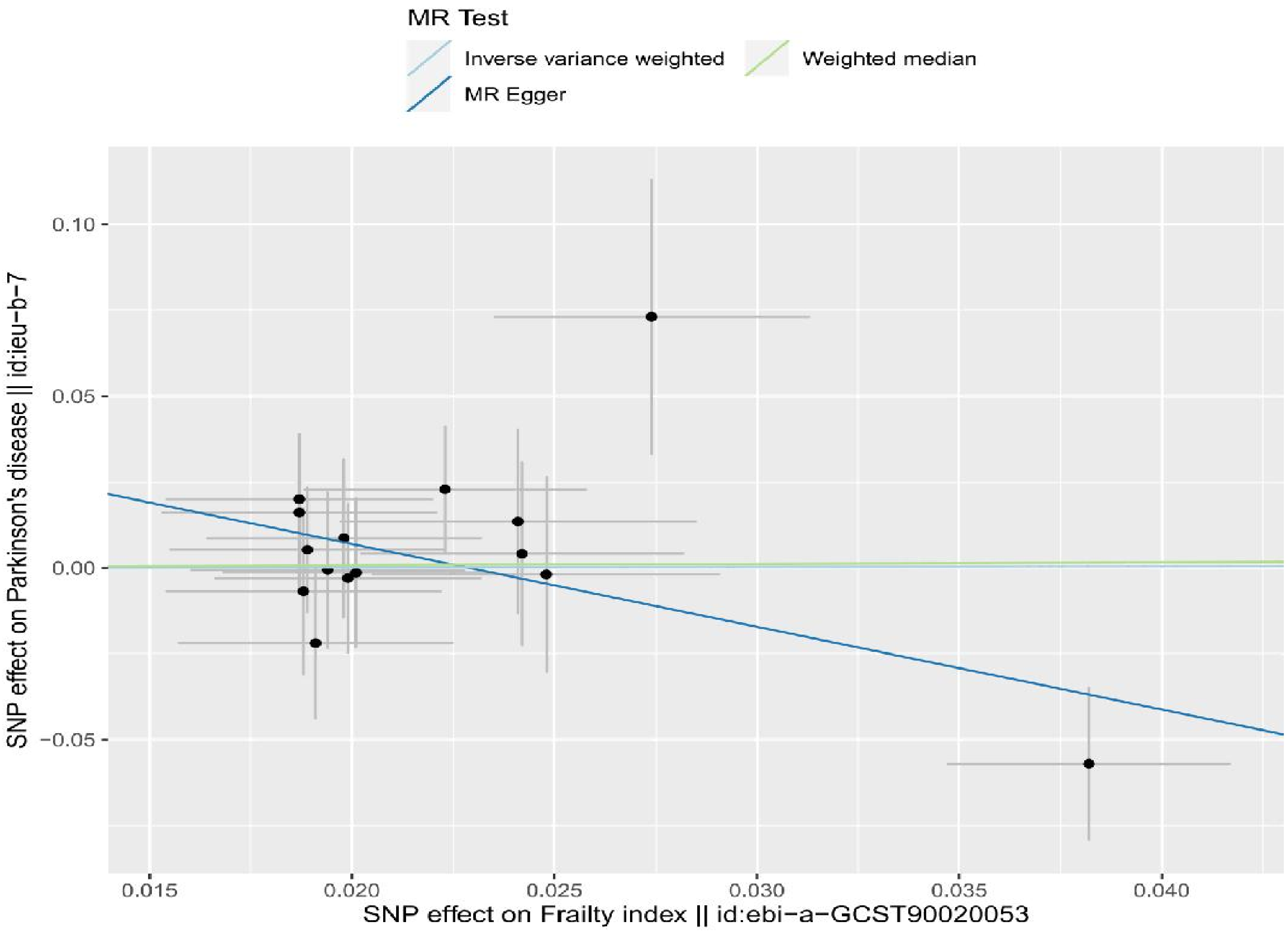

Supplementary Figure-27C Forest Plot

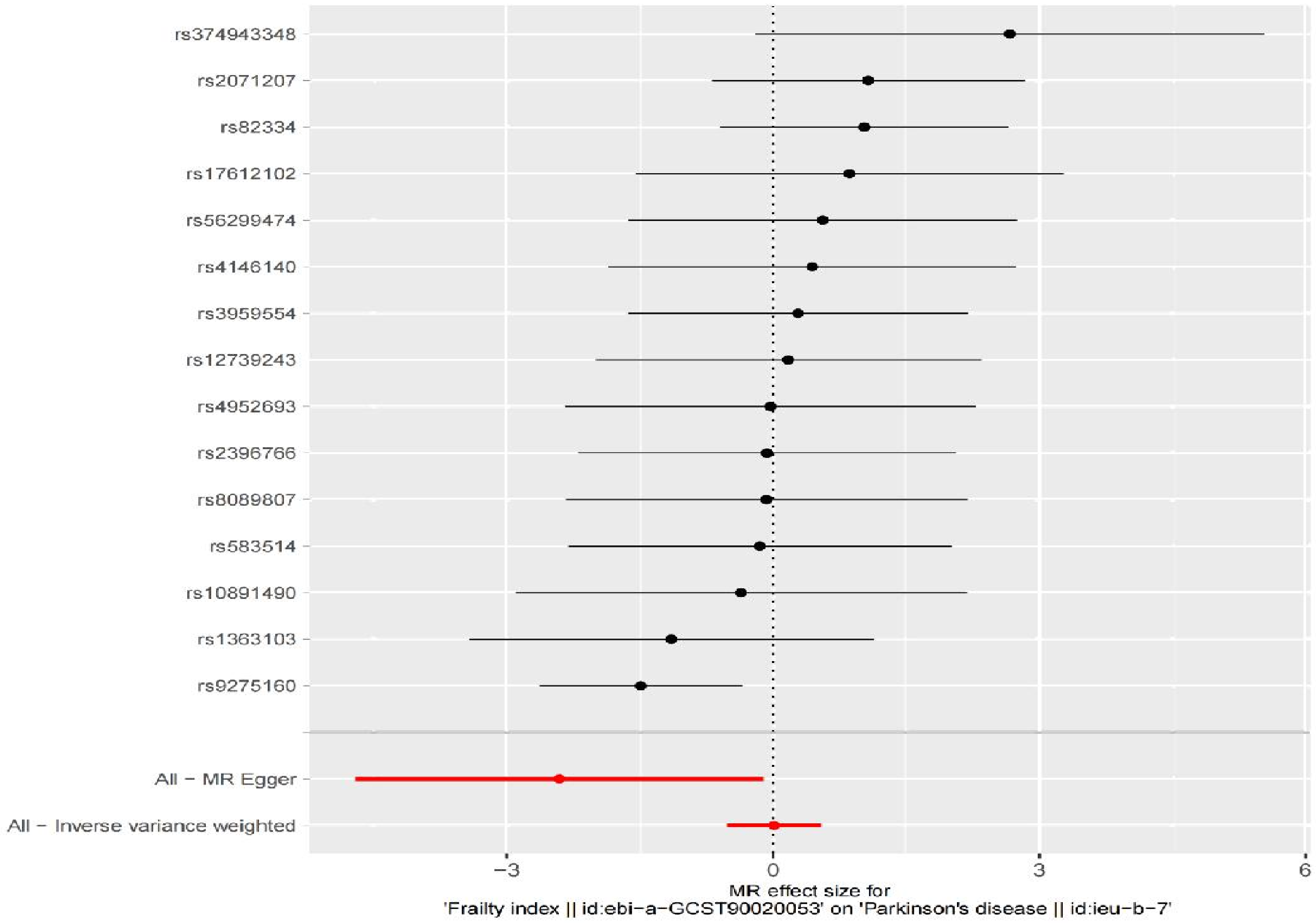

Supplementary Figure-27D Funnel Plot

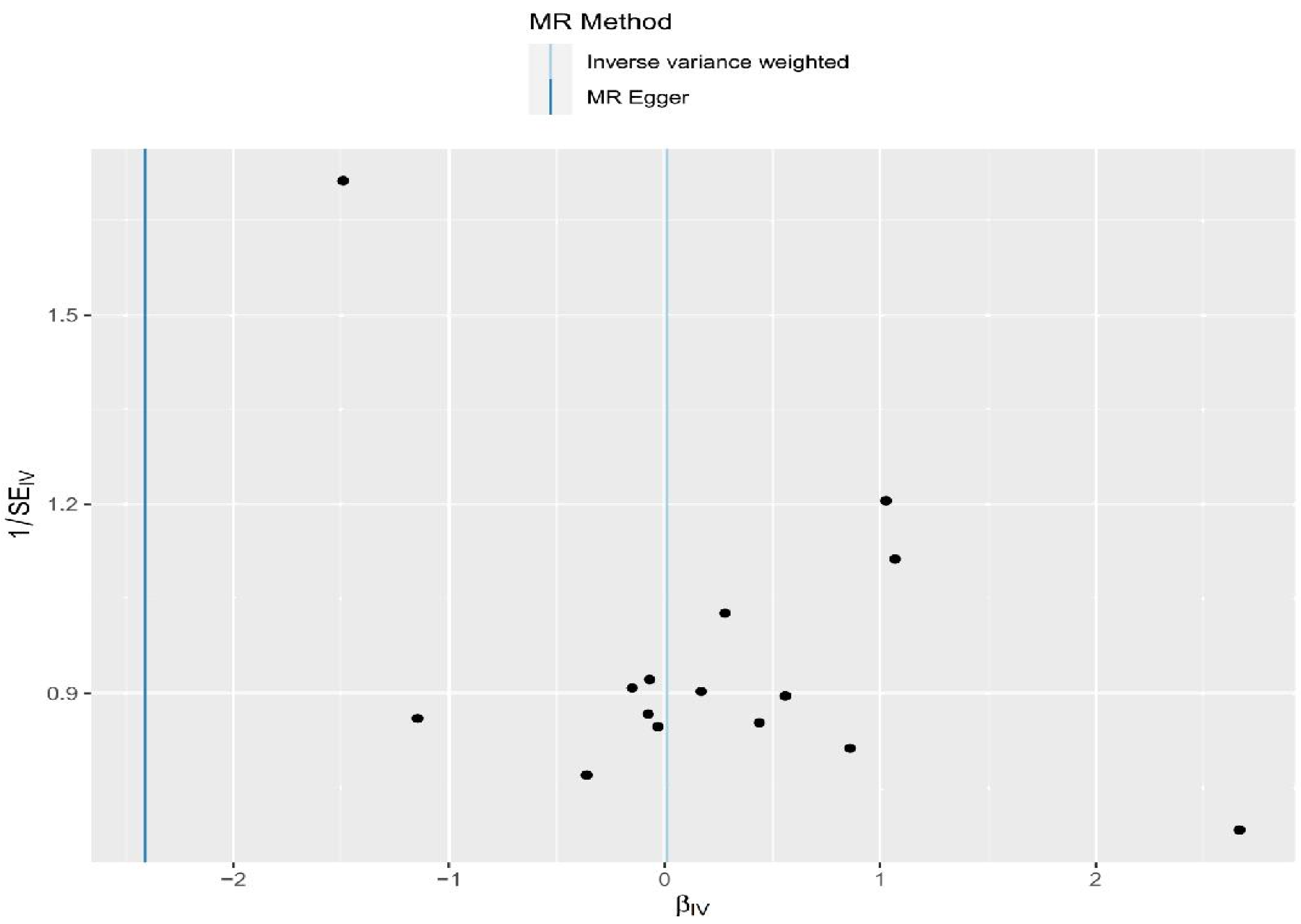

Supplementary Figure-28 Leave-one-out Analysis, Scatter Plot, Forest Plot, and Funnel Plot of Frailty Index on Vestibular Schwannomas

Supplementary Figure-28A Leave-one-out Analysis

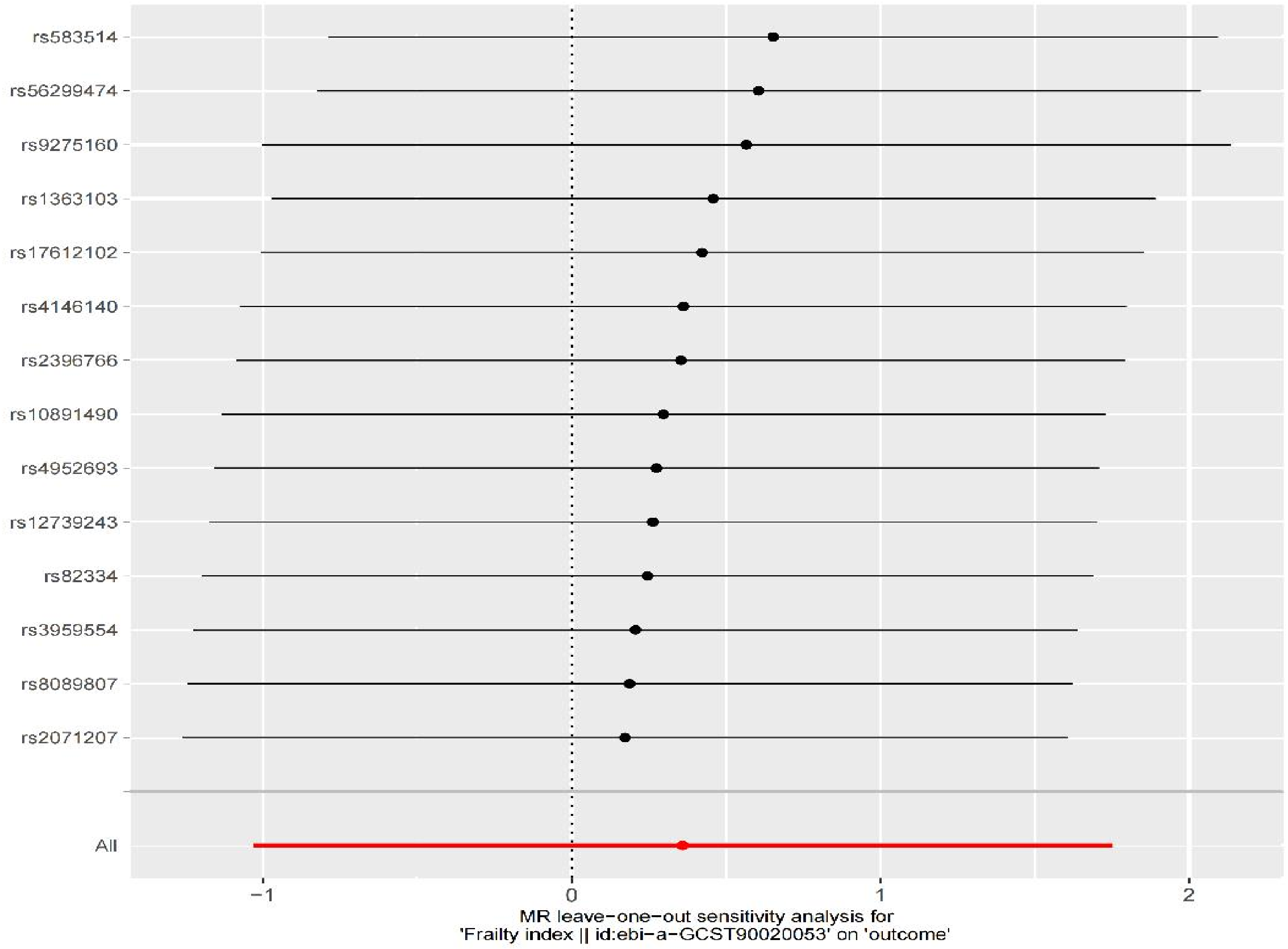

Supplementary Figure-28B Scatter

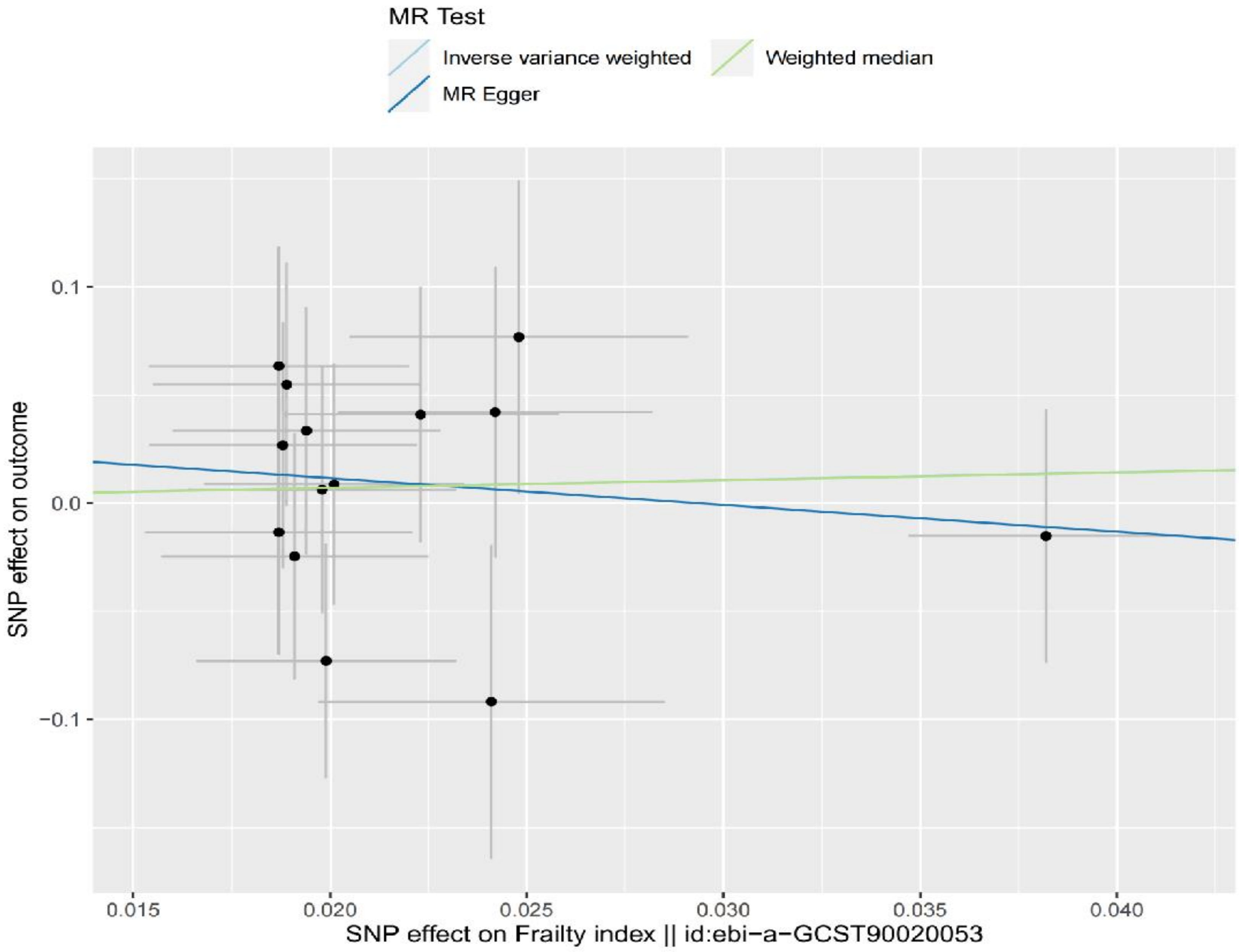

Supplementary Figure-28C Forest Plot

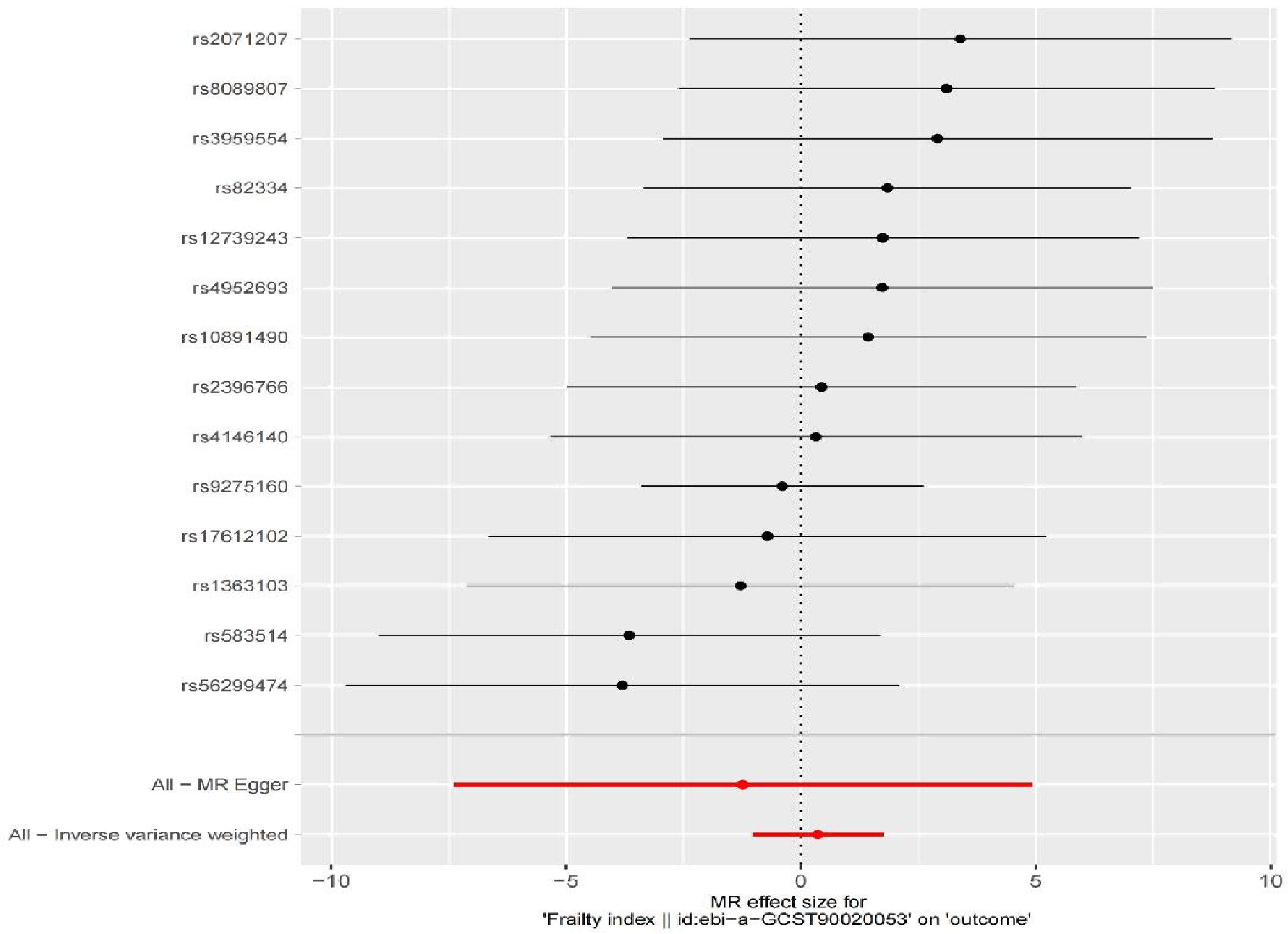

Supplementary Figure-28D Funnel Plot

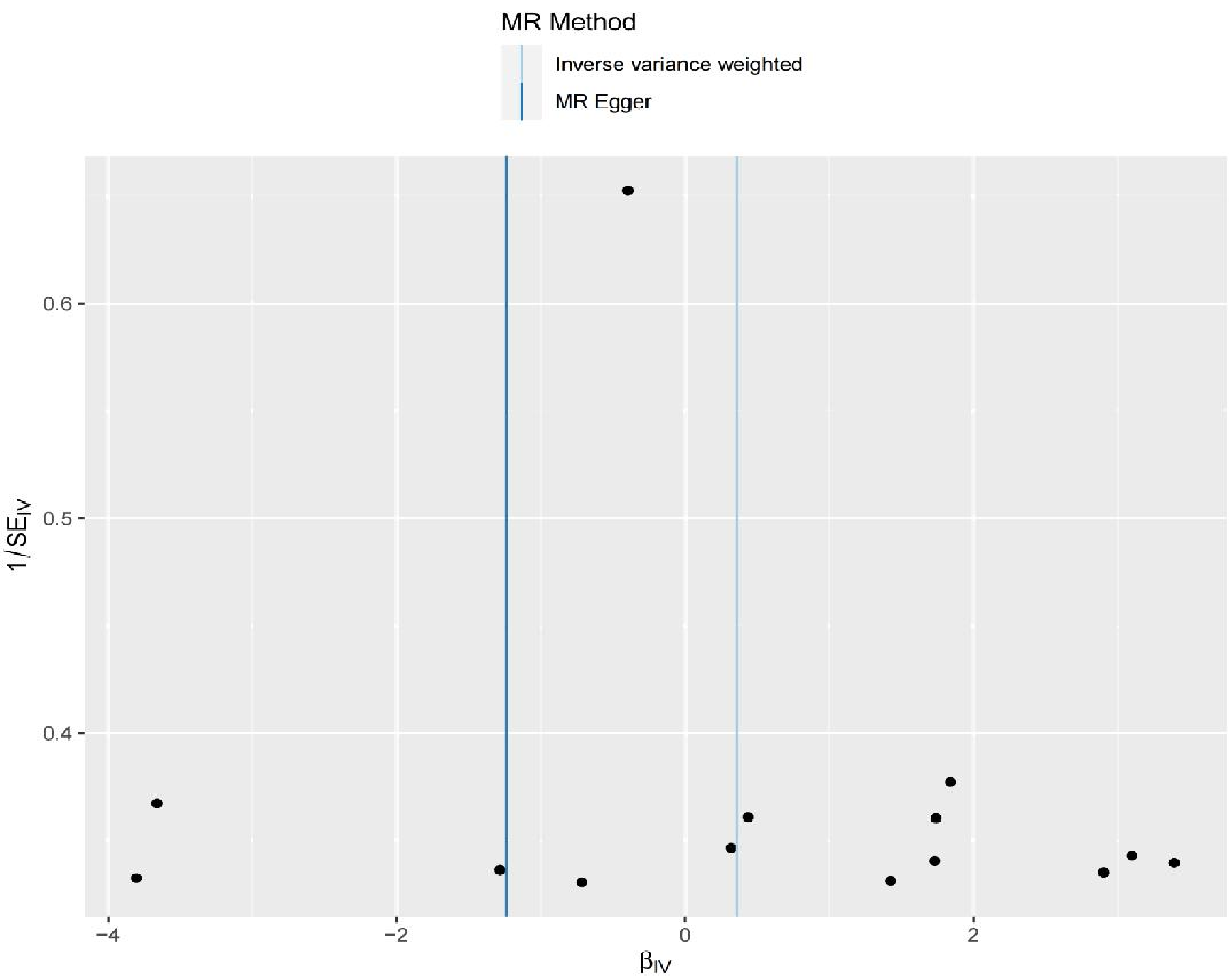

Supplementary Figure-29 Leave-one-out Analysis, Scatter Plot, Forest Plot, and Funnel Plot of GBM on Facial Ageing

Supplementary Figure-29A Leave-one-out Analysis

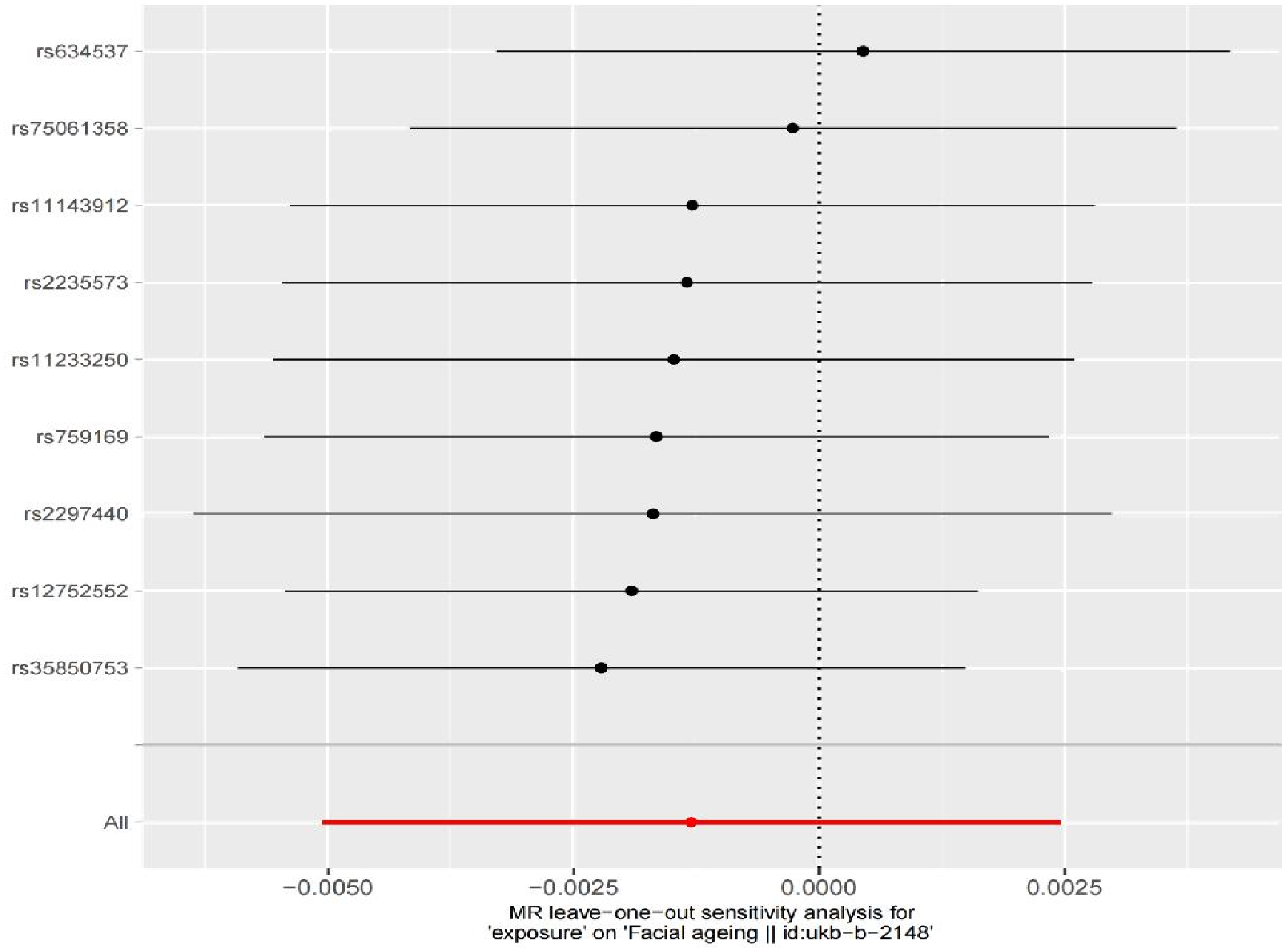

Supplementary Figure-29B Scatter

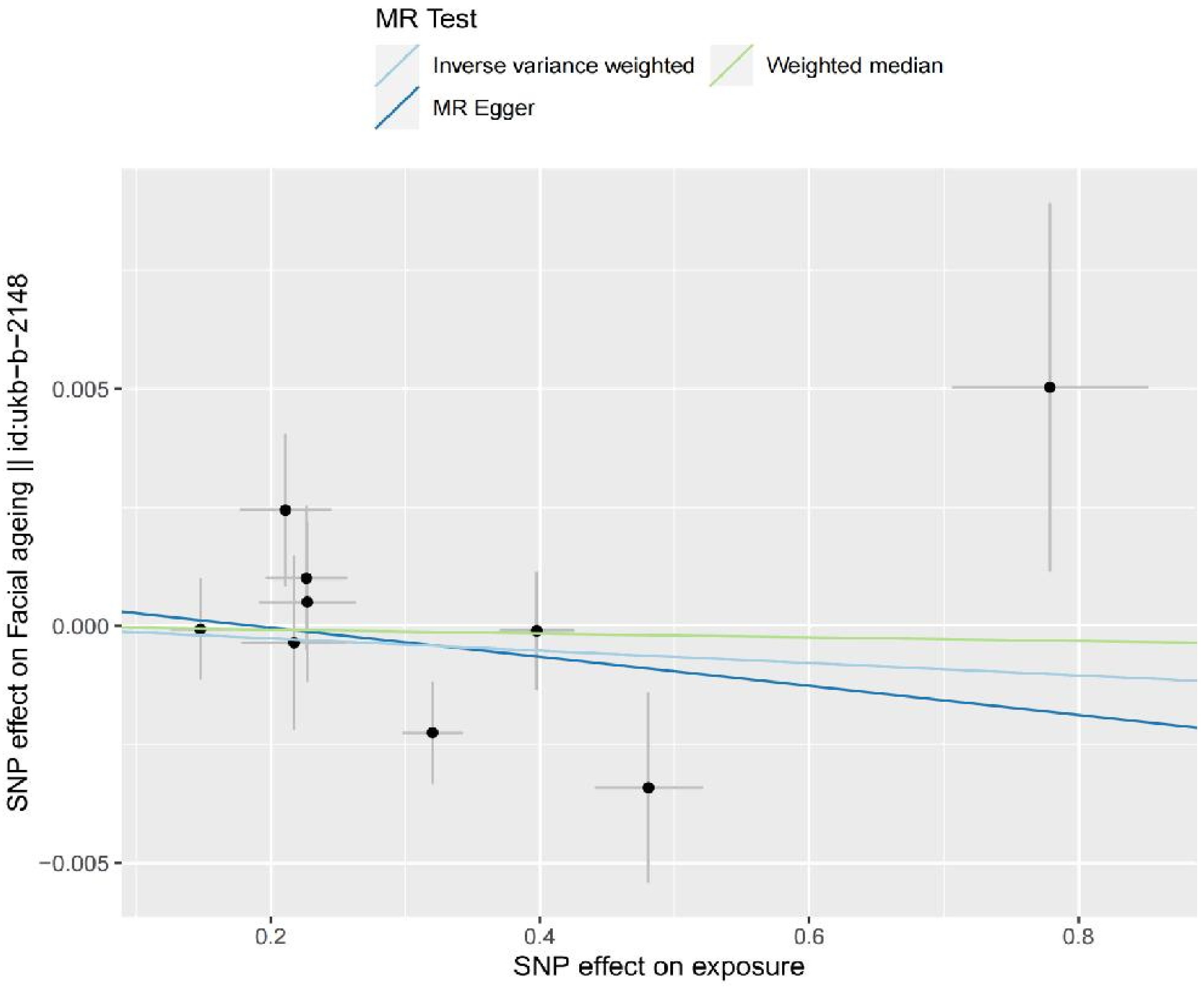

Supplementary Figure-29C Forest Plot

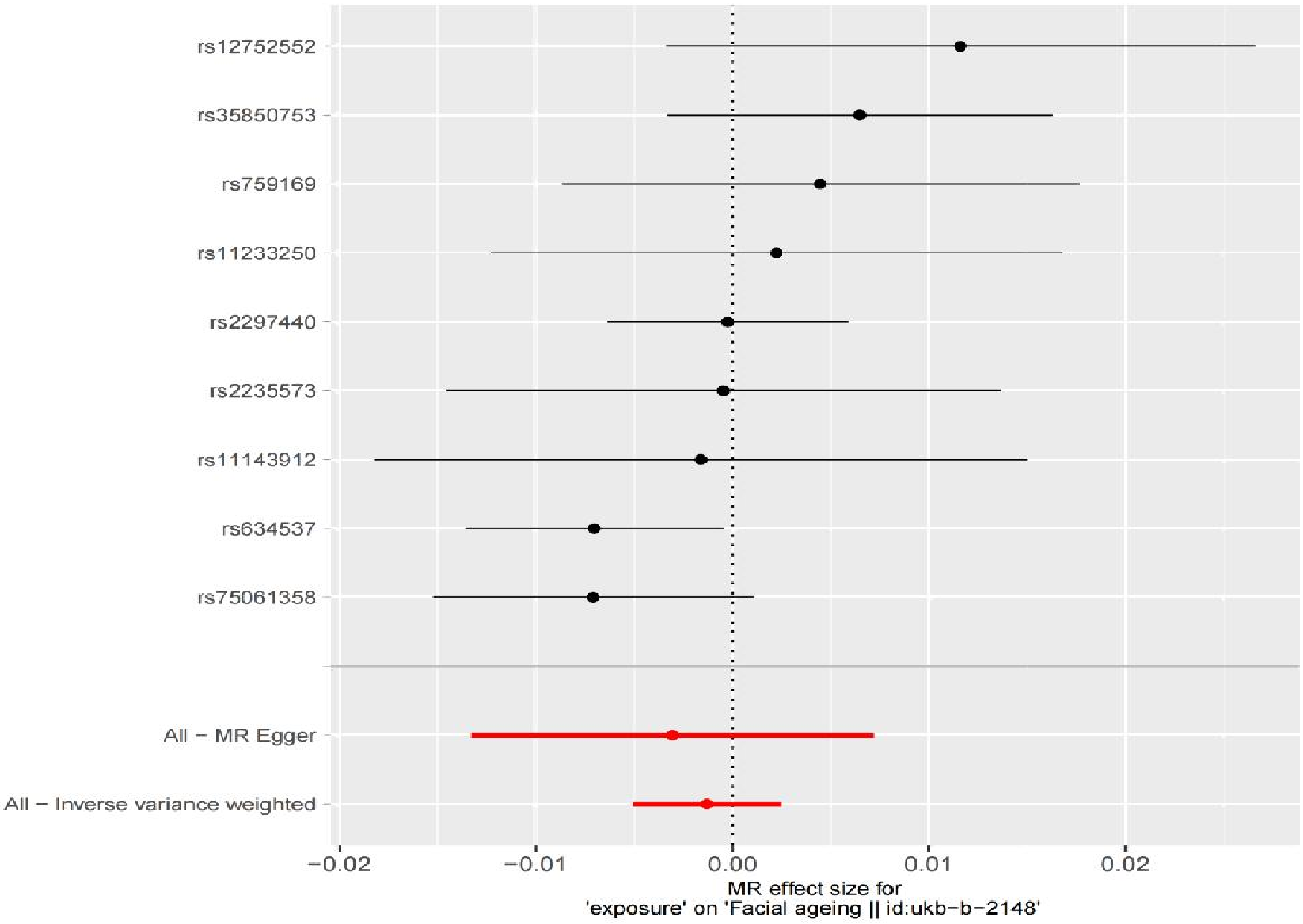

Supplementary Figure-29D Funnel Plot

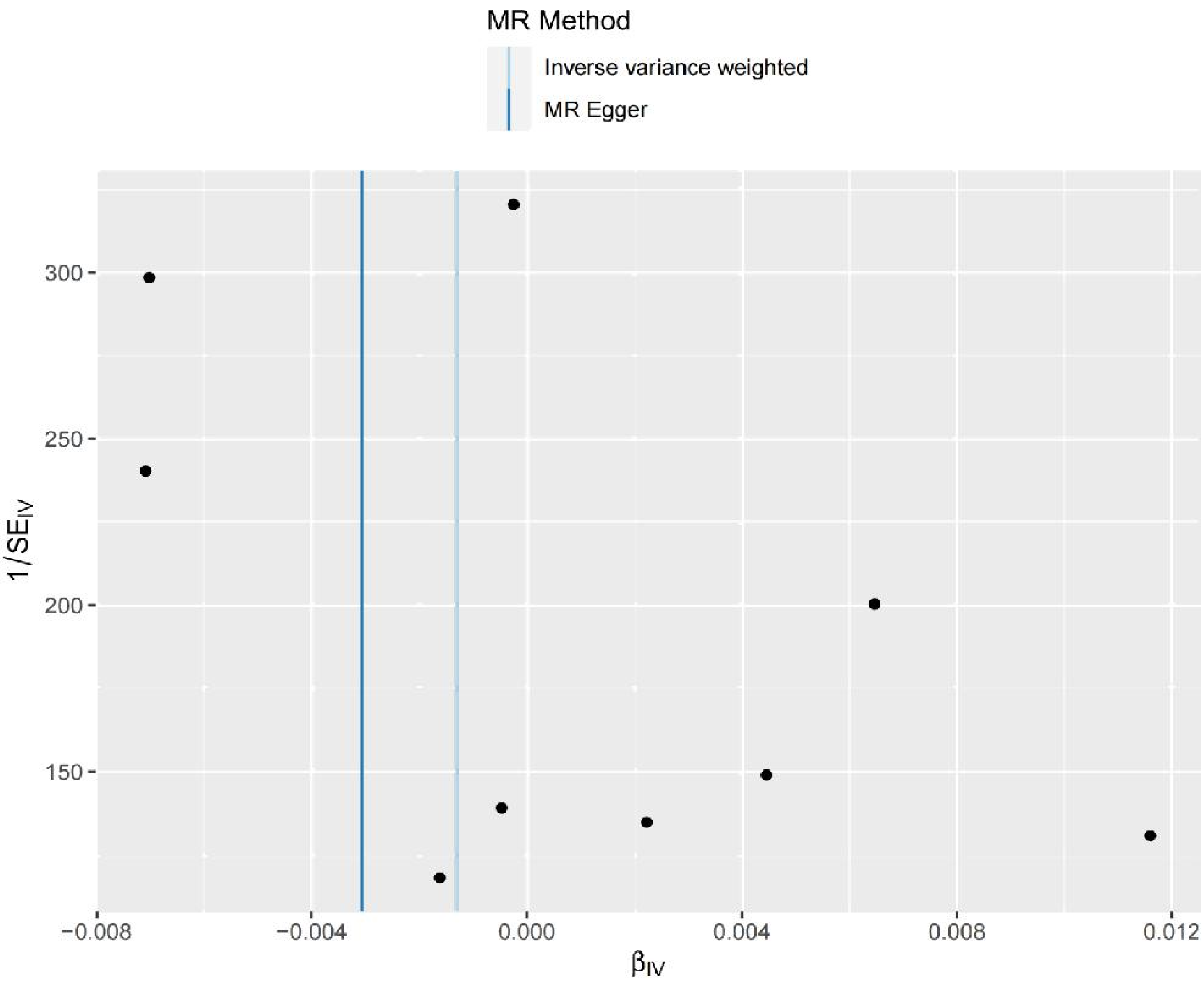

Supplementary Figure-30Leave-one-out Analysis, Scatter Plot, Forest Plot, and Funnel Plot of GBM on Frailty Index

Supplementary Figure-30A Leave-one-out Analysis

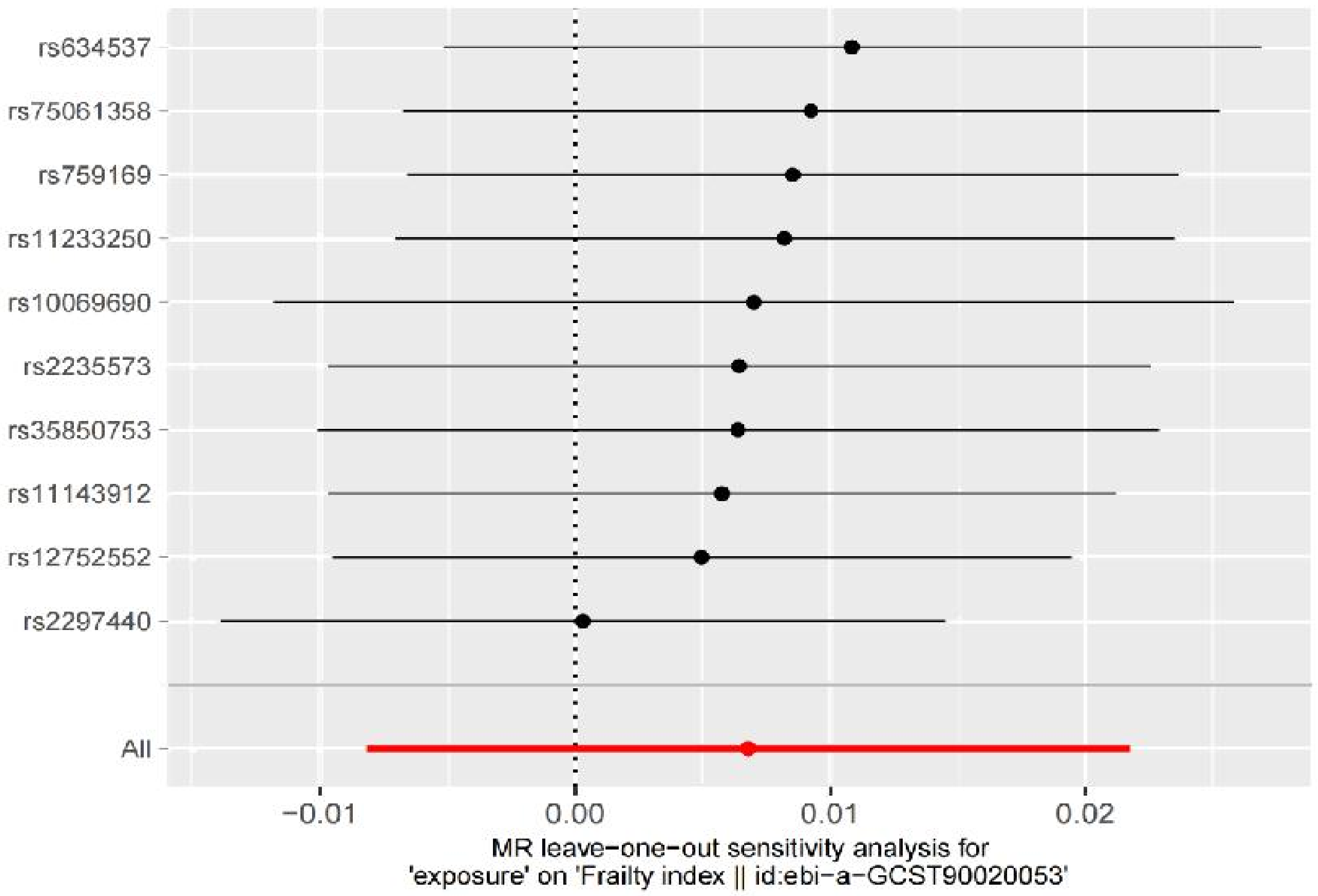

Supplementary Figure-30B Scatter

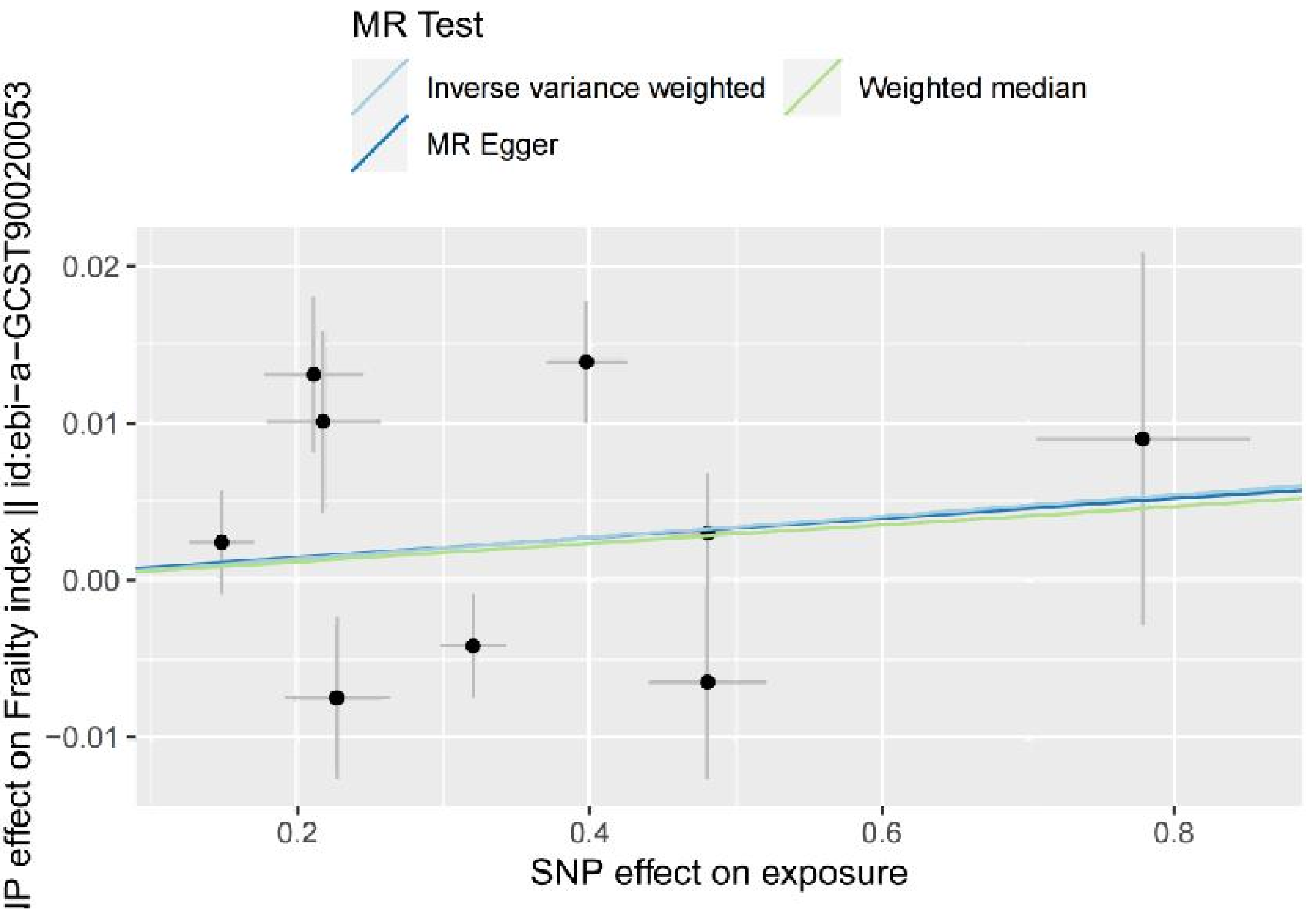

Supplementary Figure-30C Forest Plot

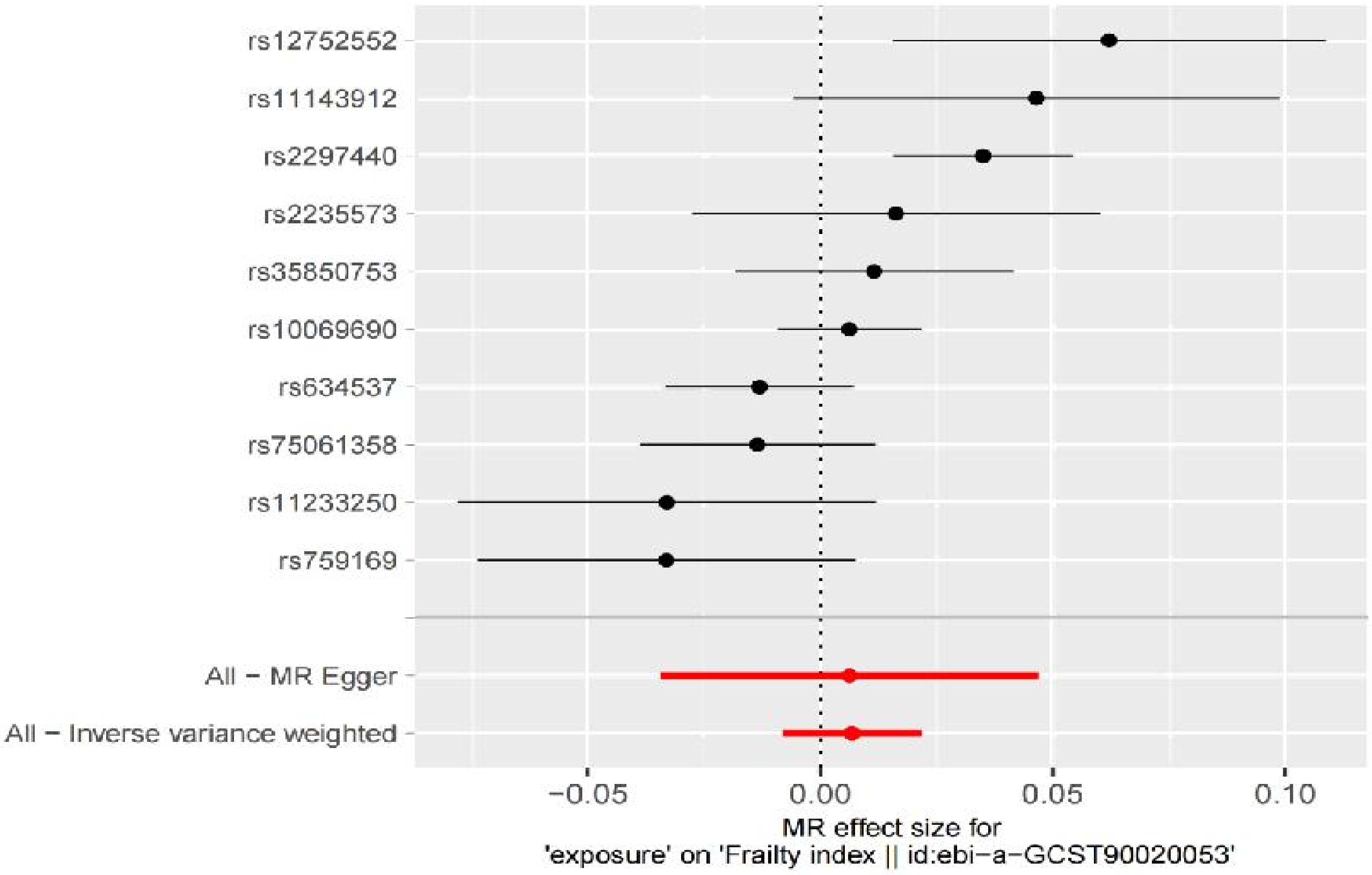

Supplementary Figure-30D Funnel Plot

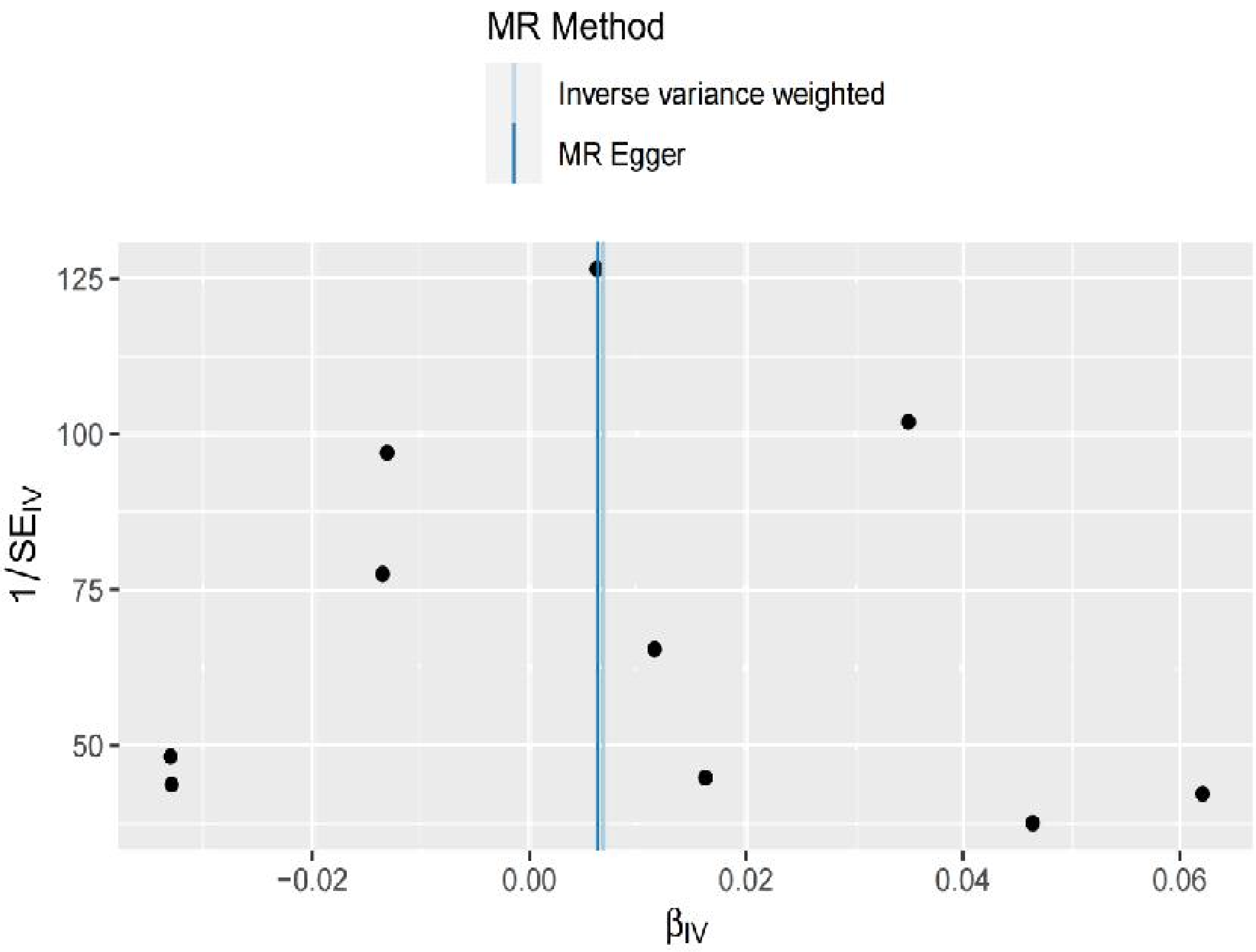

Supplementary Figure-31 Leave-one-out Analysis, Scatter Plot, Forest Plot, and Funnel Plot of GBM on DNA methylation GrimAge Acceleration

Supplementary Figure-31A Leave-one-out Analysis

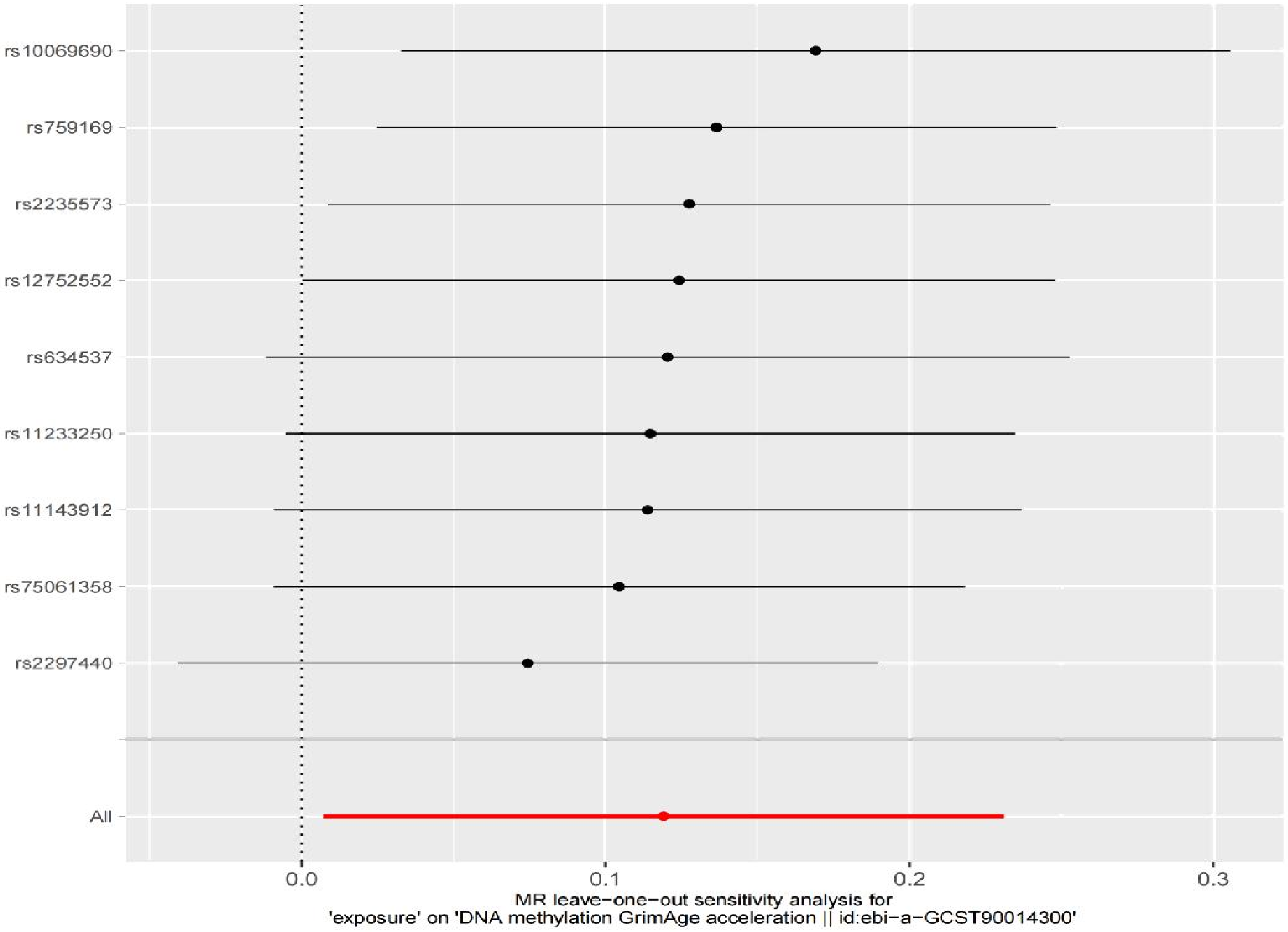

Supplementary Figure-31B Scatter

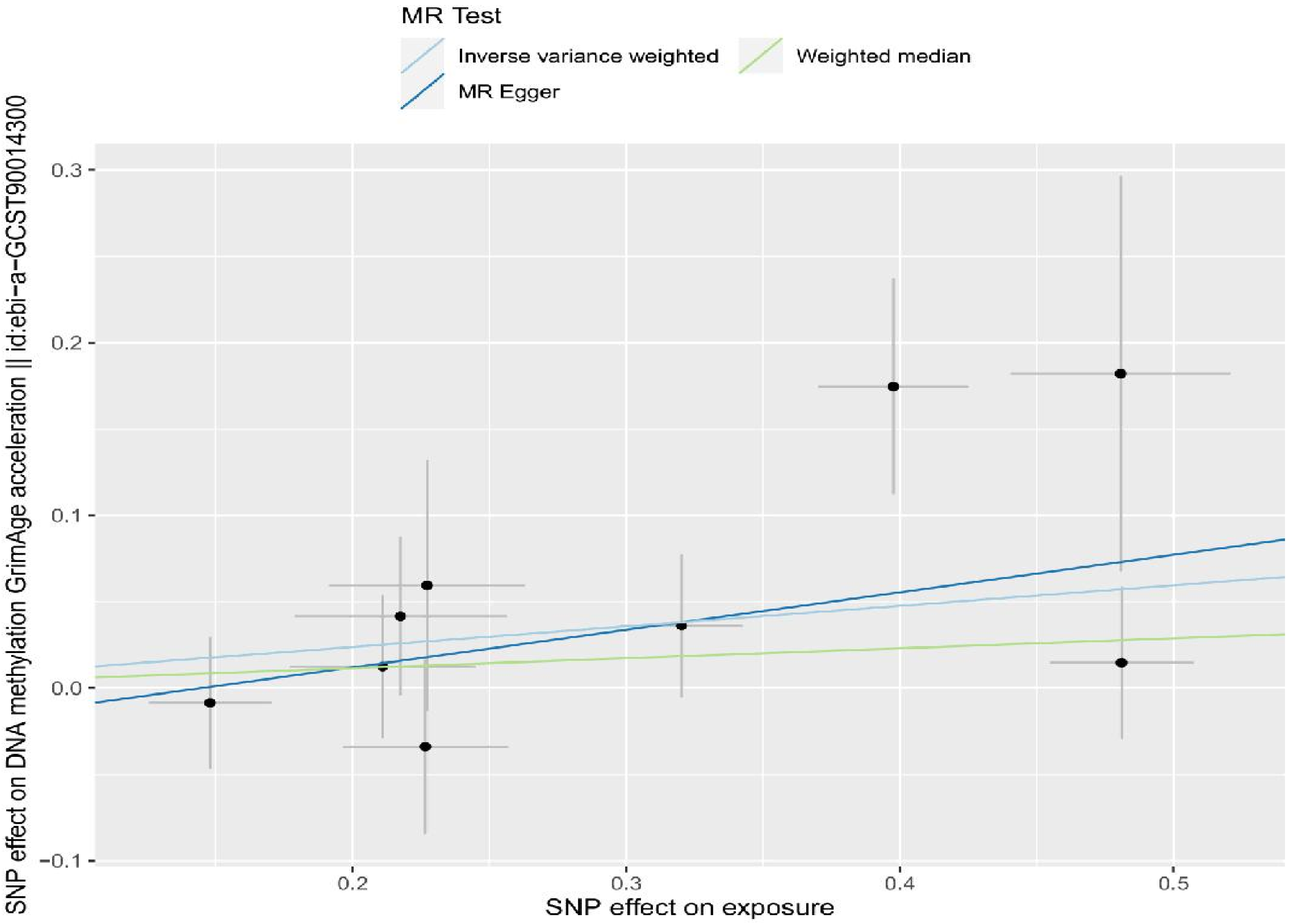

Supplementary Figure-31C Forest Plot

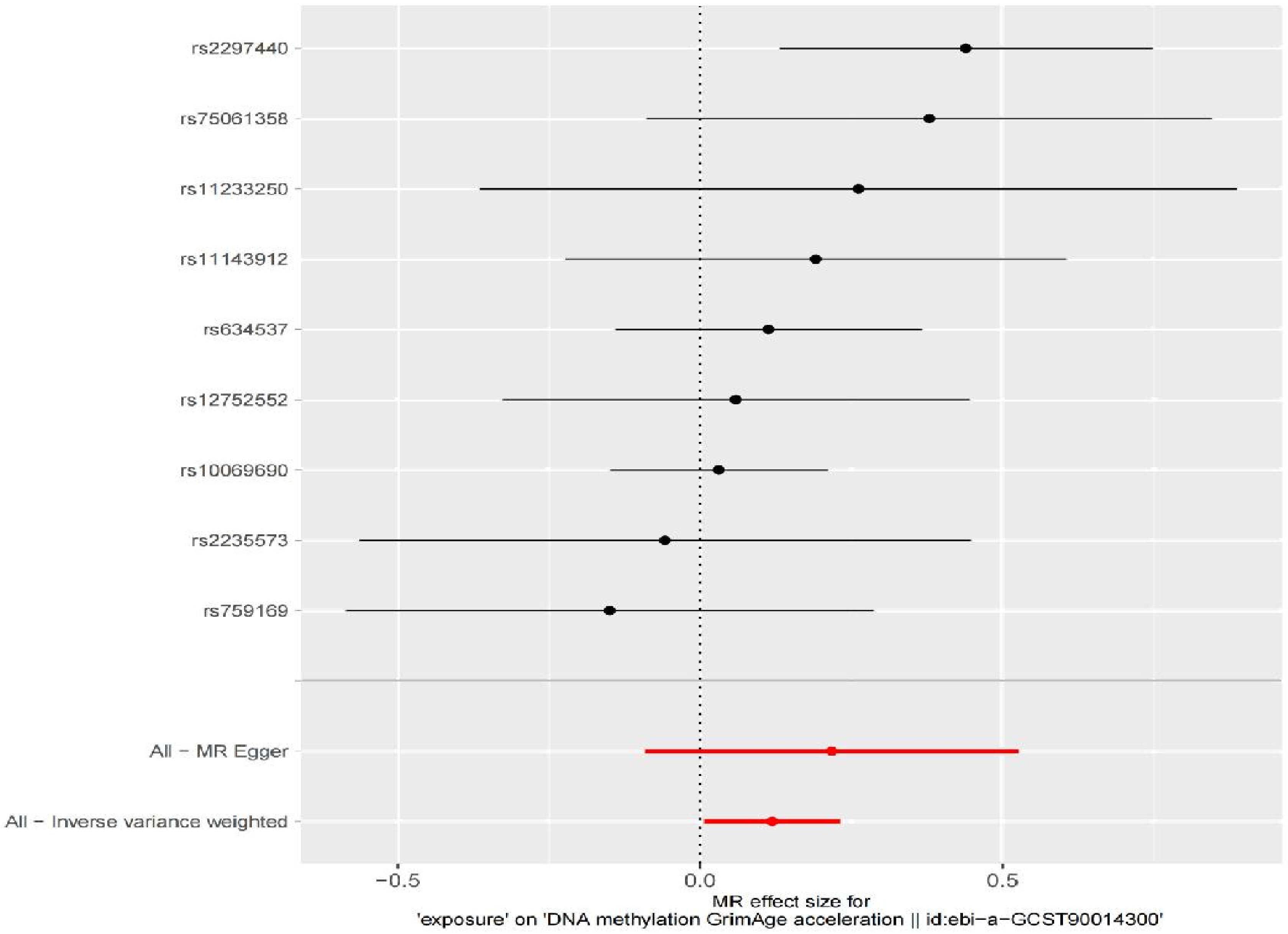

Supplementary Figure-31D Funnel Plot

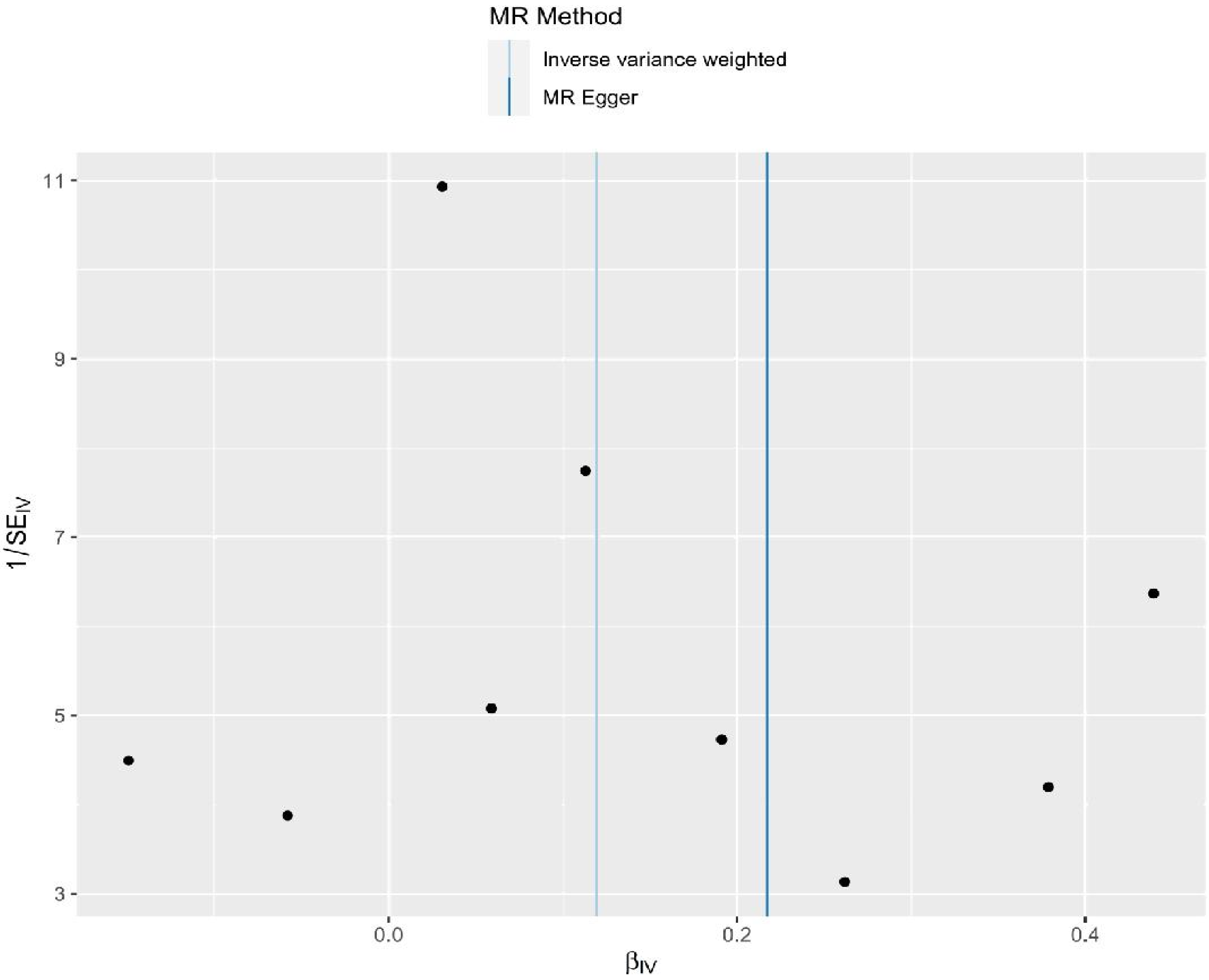

Supplementary Figure-32 Leave-one-out Analysis, Scatter Plot, Forest Plot, and Funnel Plot of GBM on Telomere Length

Supplementary Figure-32A Leave-one-out Analysis

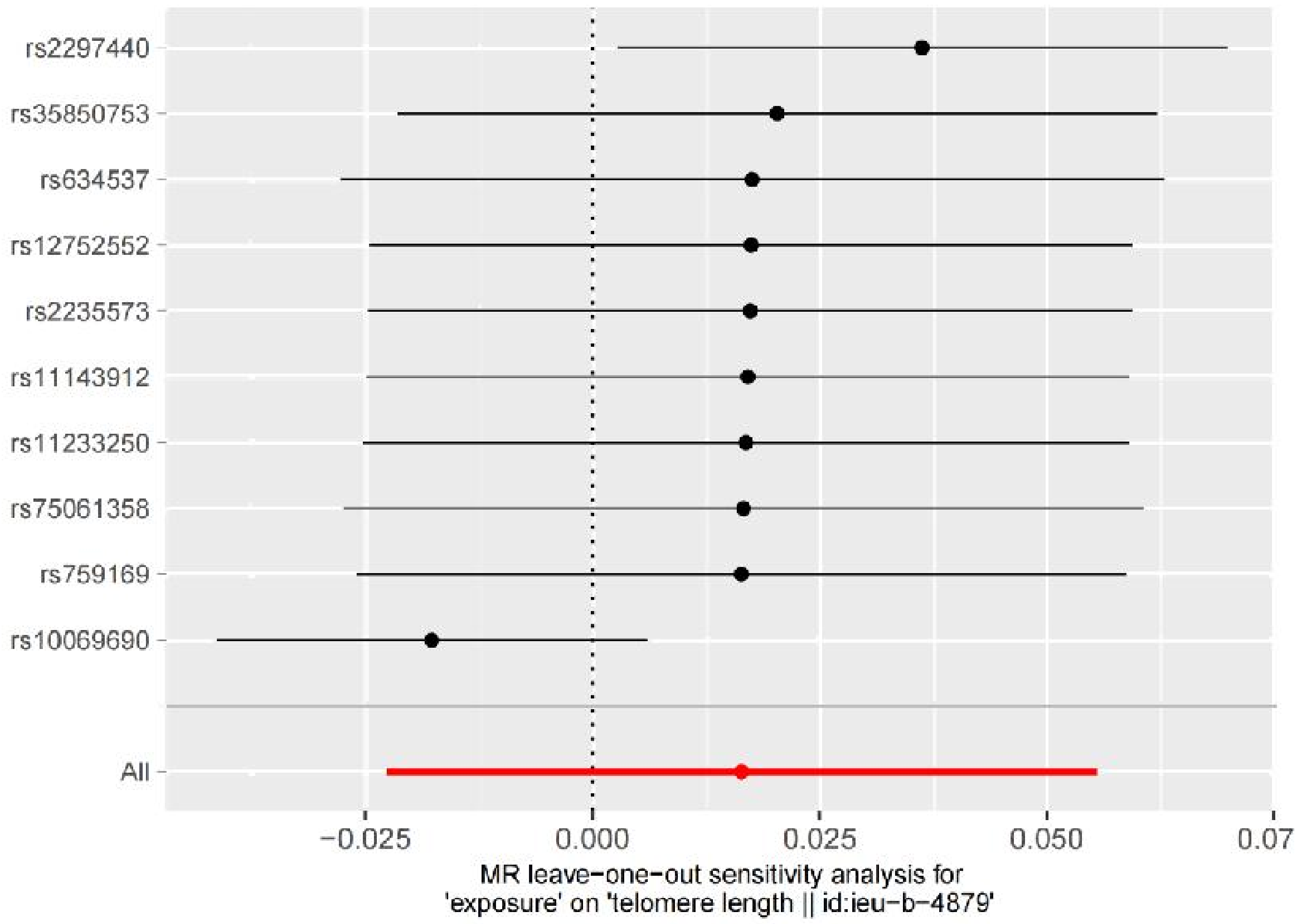

Supplementary Figure-32B Scatter

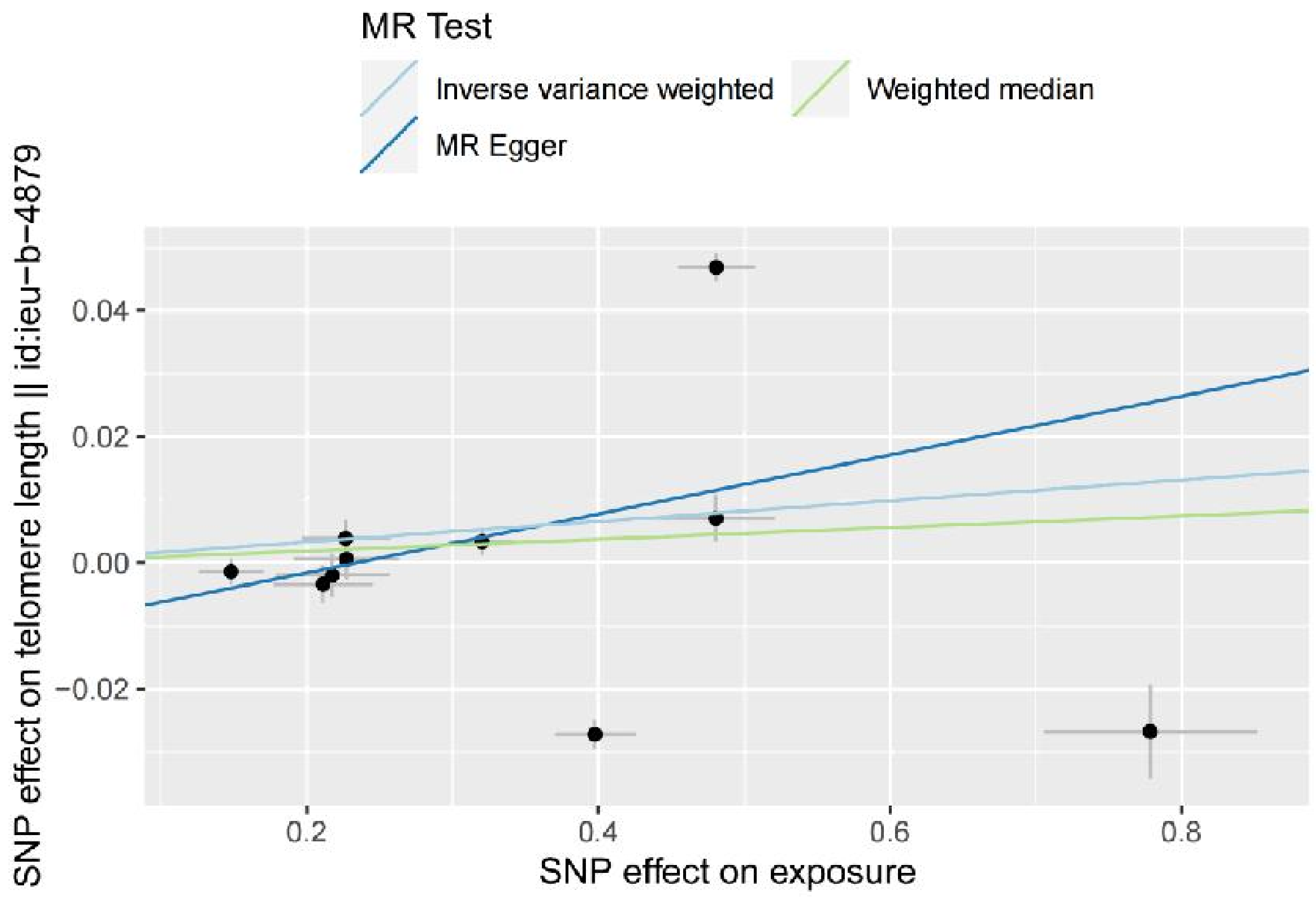

Supplementary Figure-32C Forest Plot

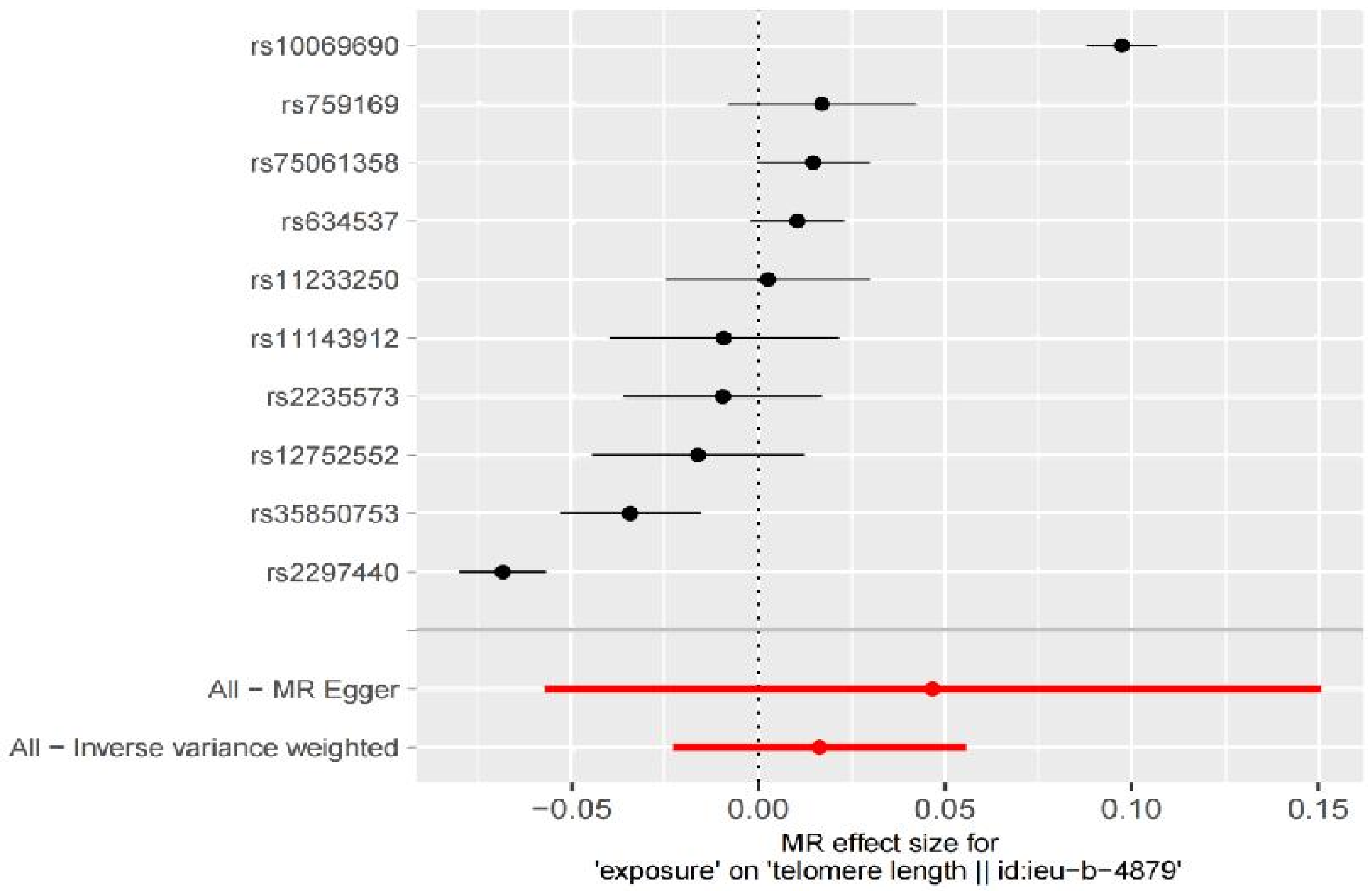

Supplementary Figure-32D Funnel Plot

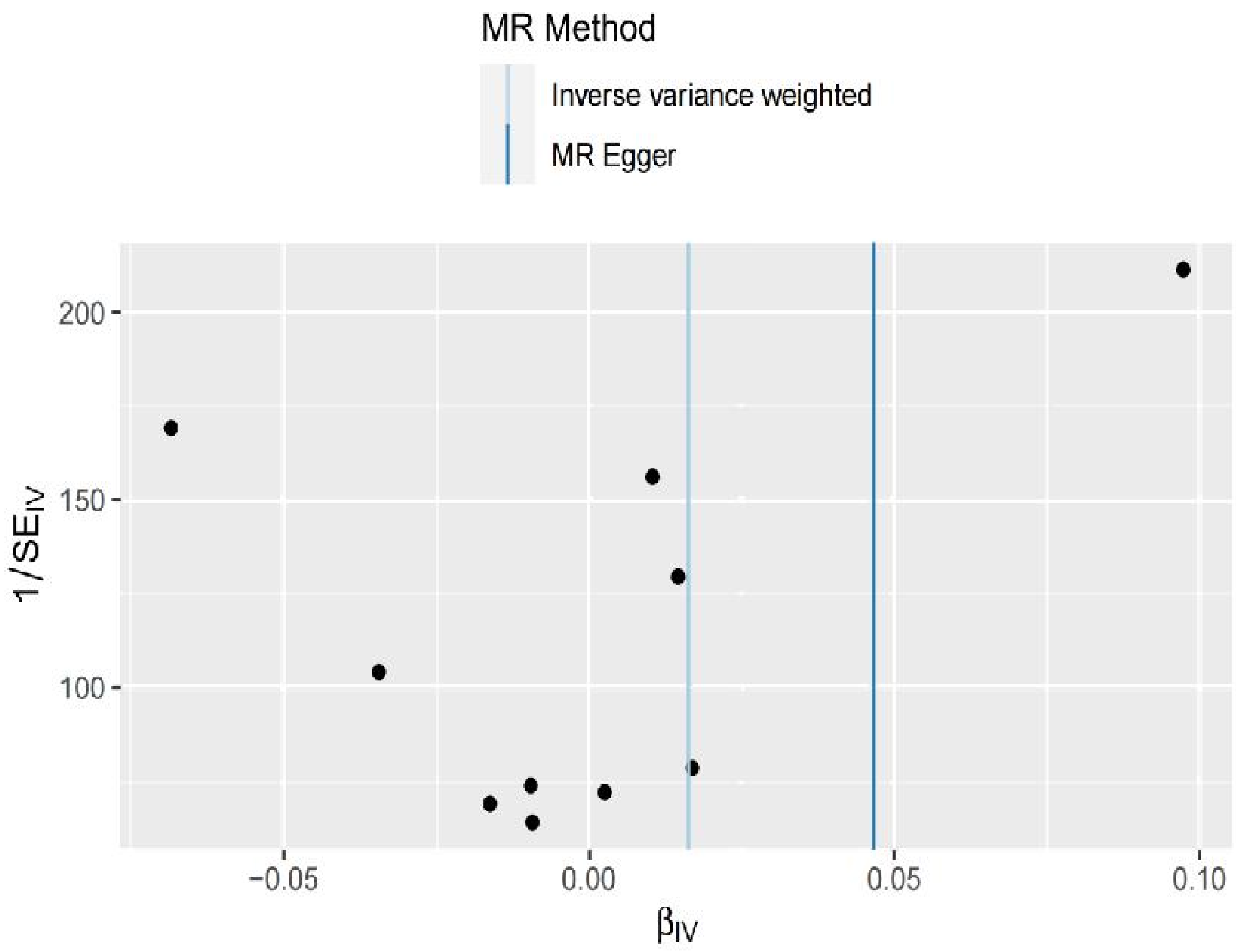

Supplementary Figure-33 Leave-one-out Analysis, Scatter Plot, Forest Plot, and Funnel Plot of DNA methylation GrimAge Acceleration on Alzheimer's Disease

Supplementary Figure-33A Leave-one-out Analysis

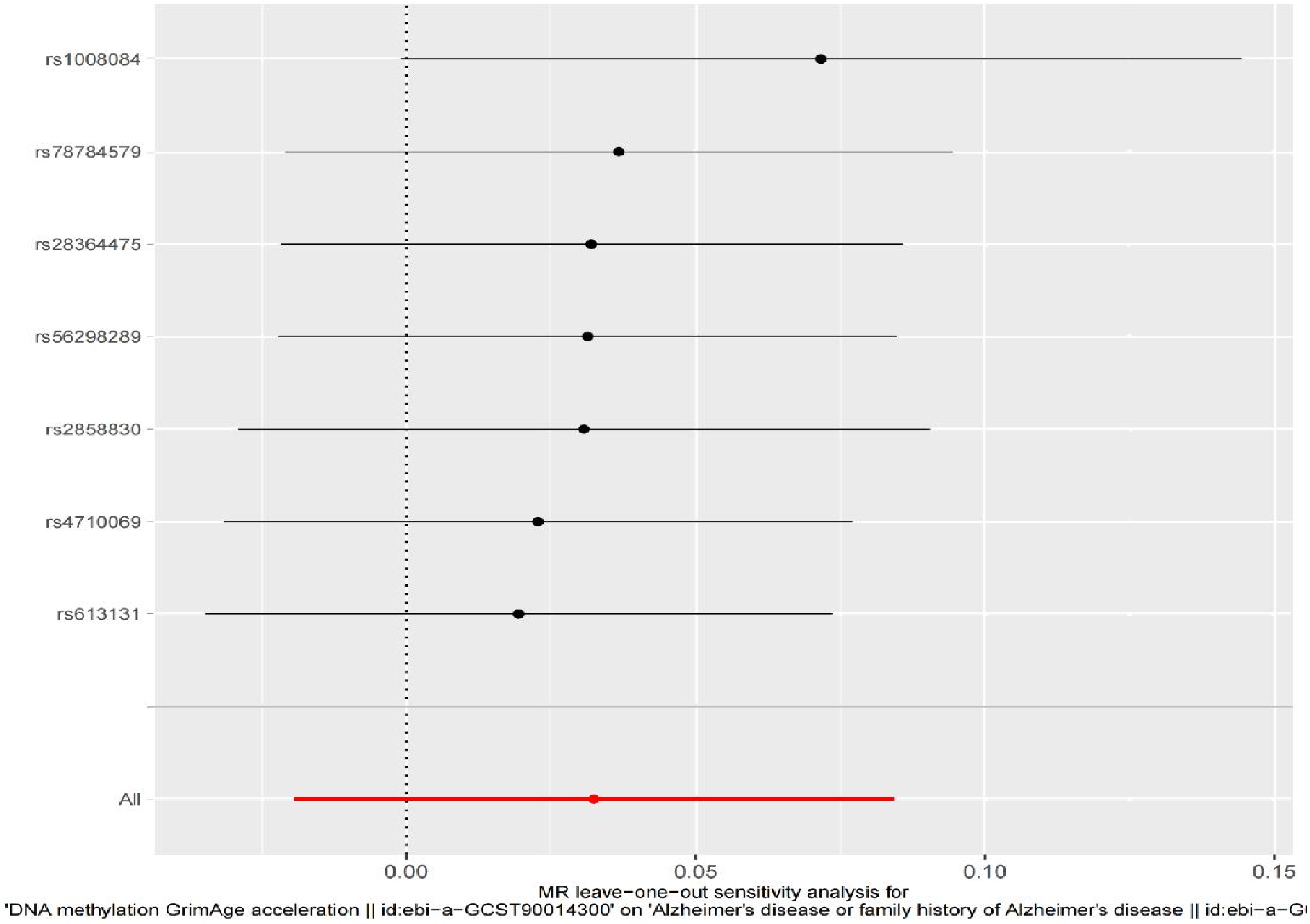

Supplementary Figure-33B Scatter

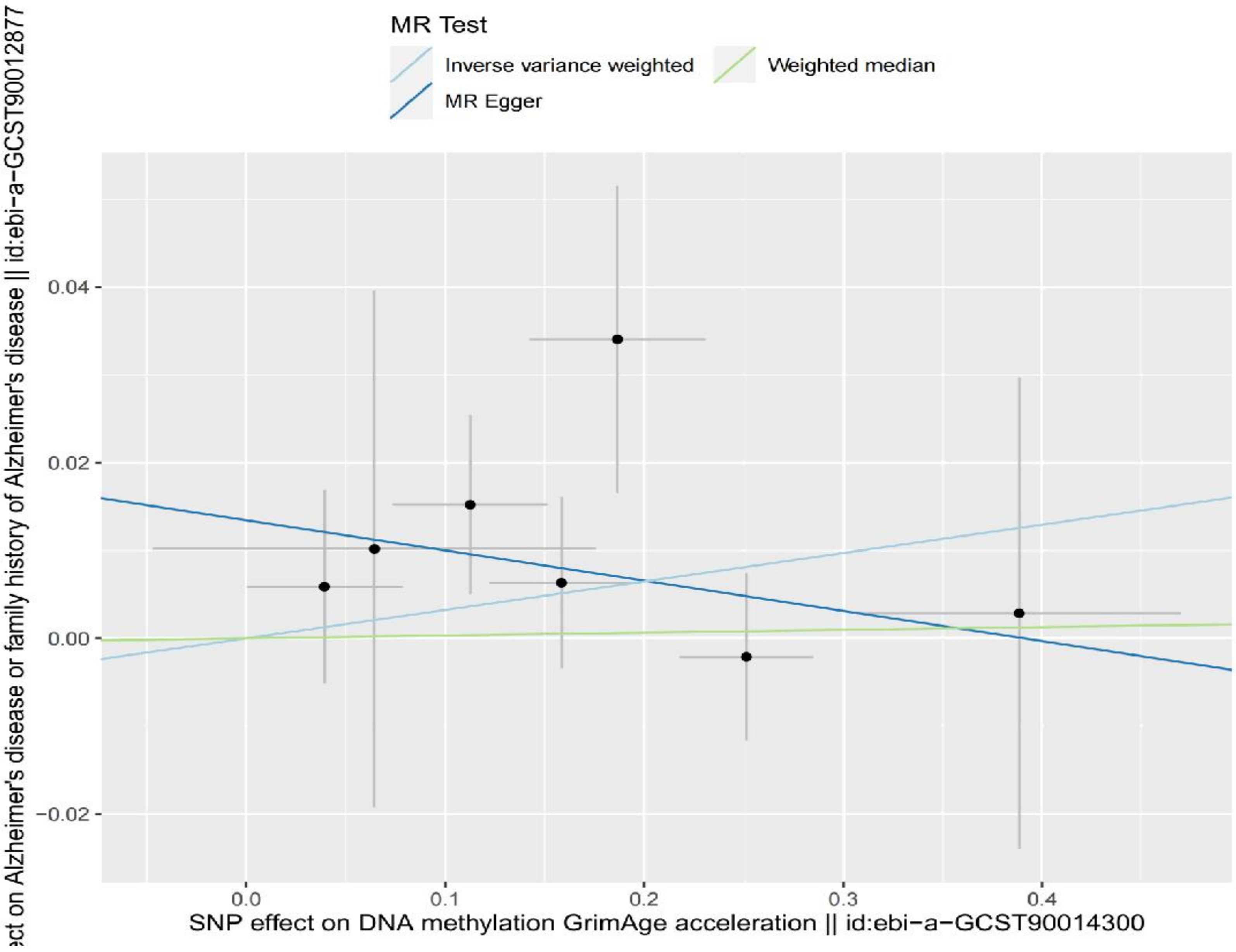

Supplementary Figure-33C Forest Plot

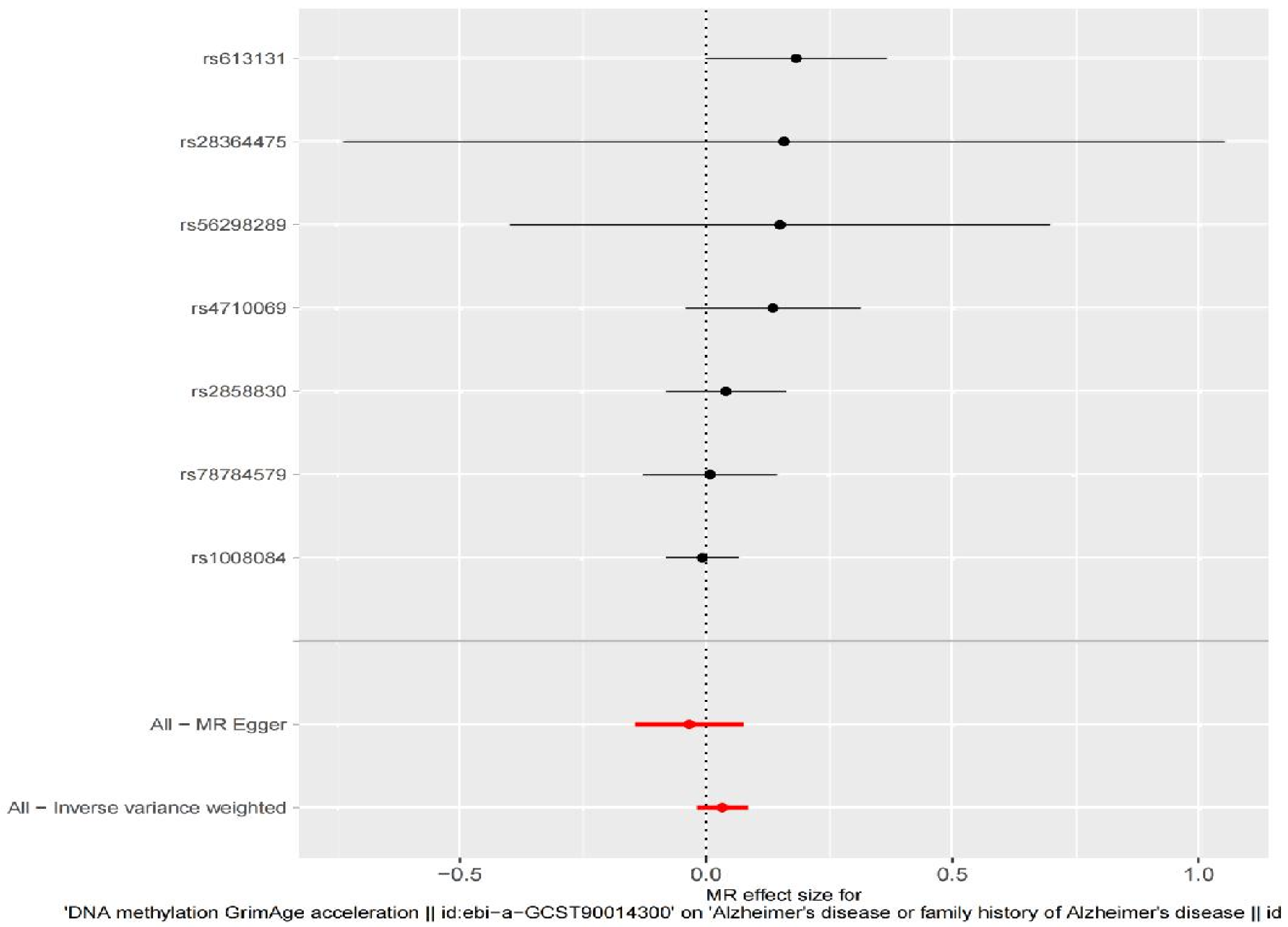

Supplementary Figure-33D Funnel Plot

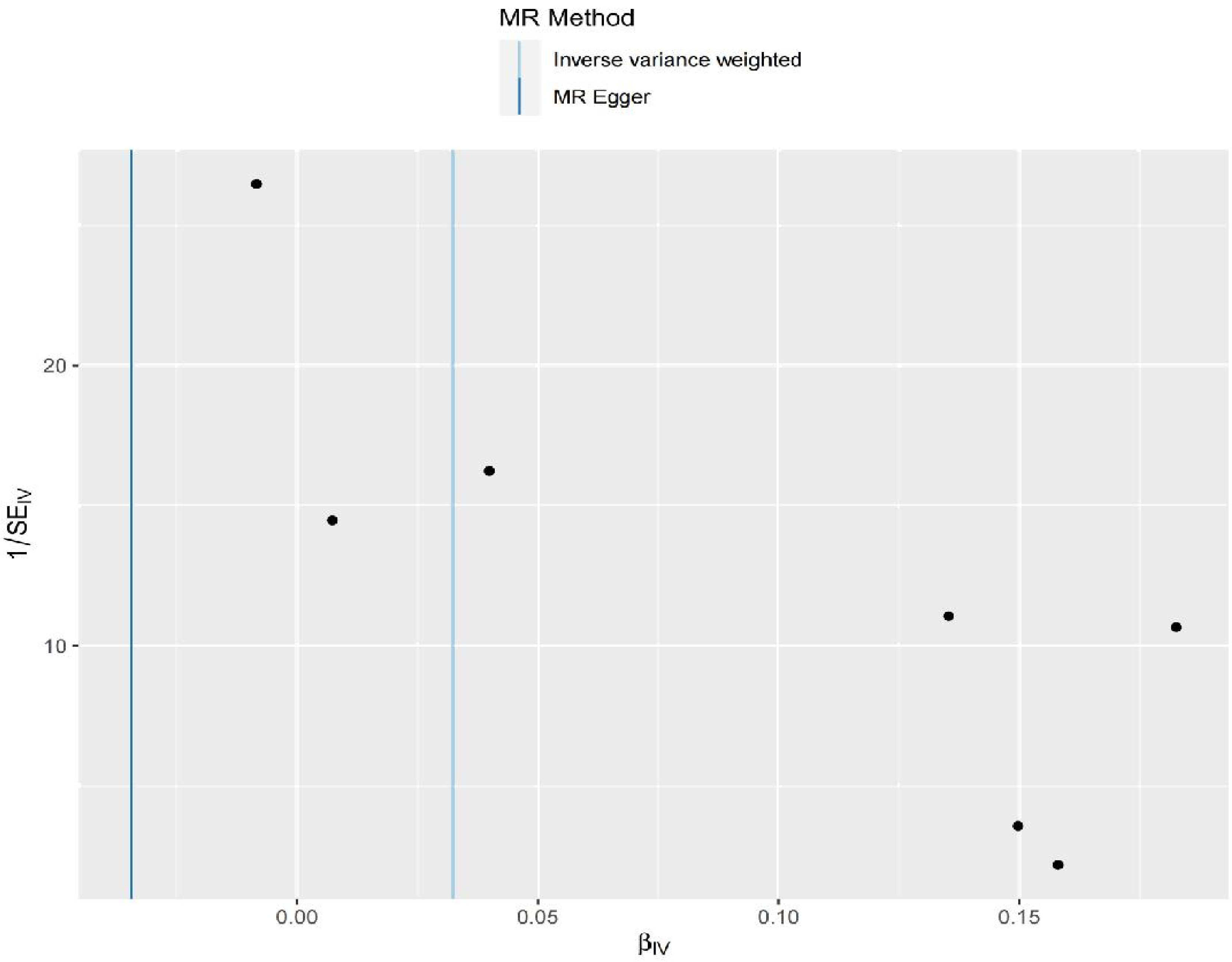

Supplementary Figure-34 Leave-one-out Analysis, Scatter Plot, Forest Plot, and Funnel Plot of DNA methylation GrimAge Acceleration on All Glioma

Supplementary Figure-34A Leave-one-out Analysis

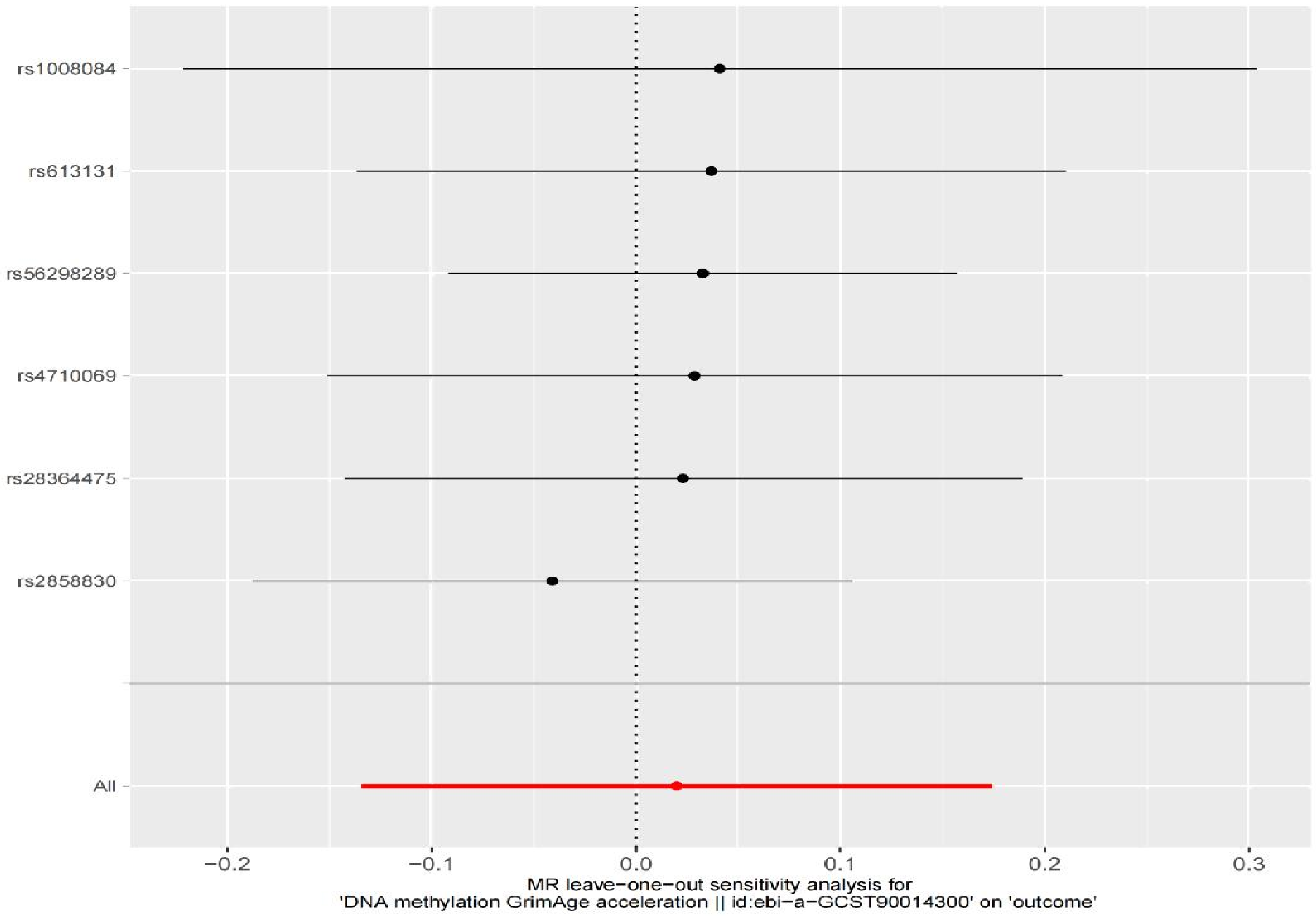

Supplementary Figure-34B Scatter

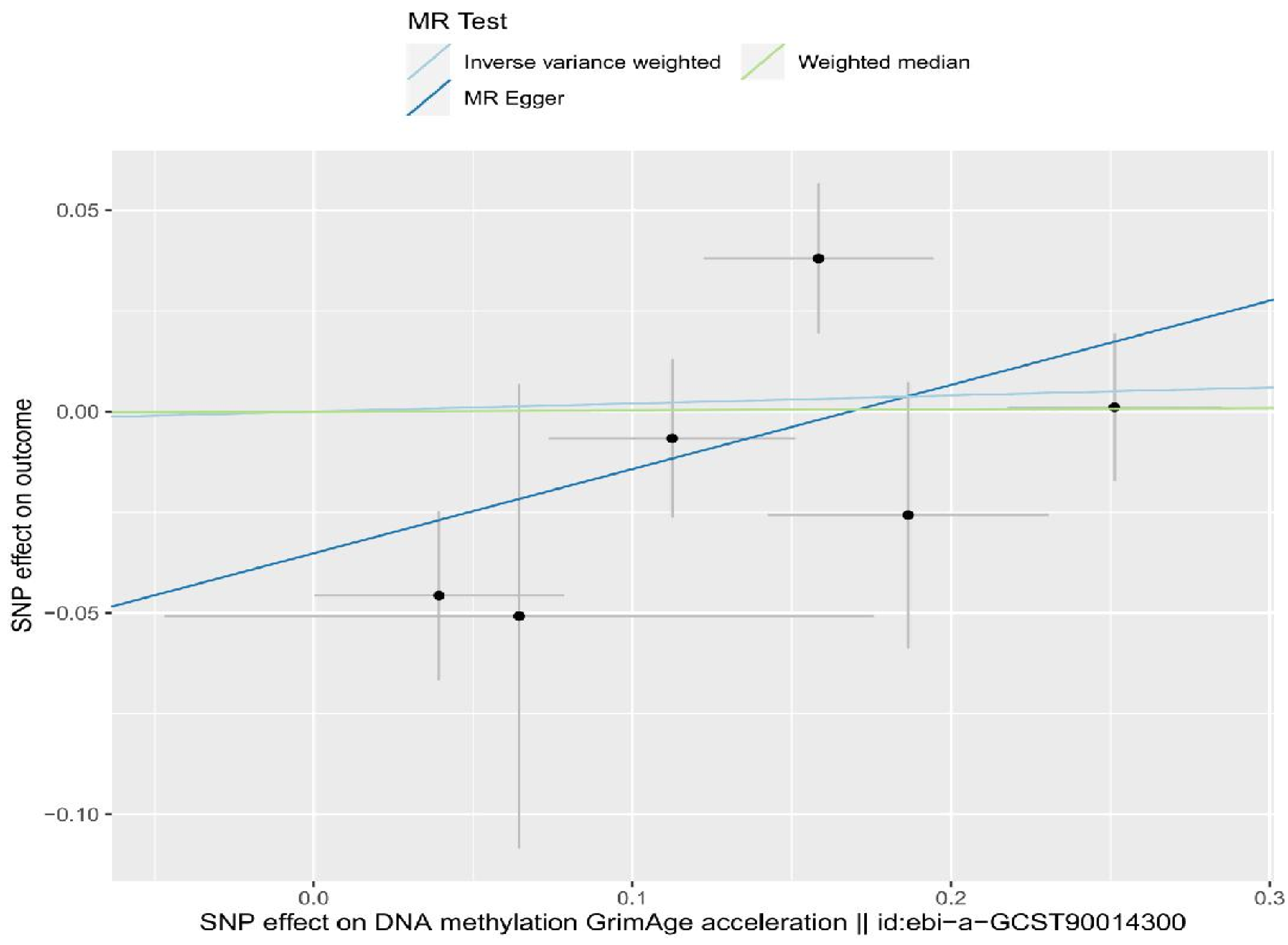

Supplementary Figure-34C Forest Plot

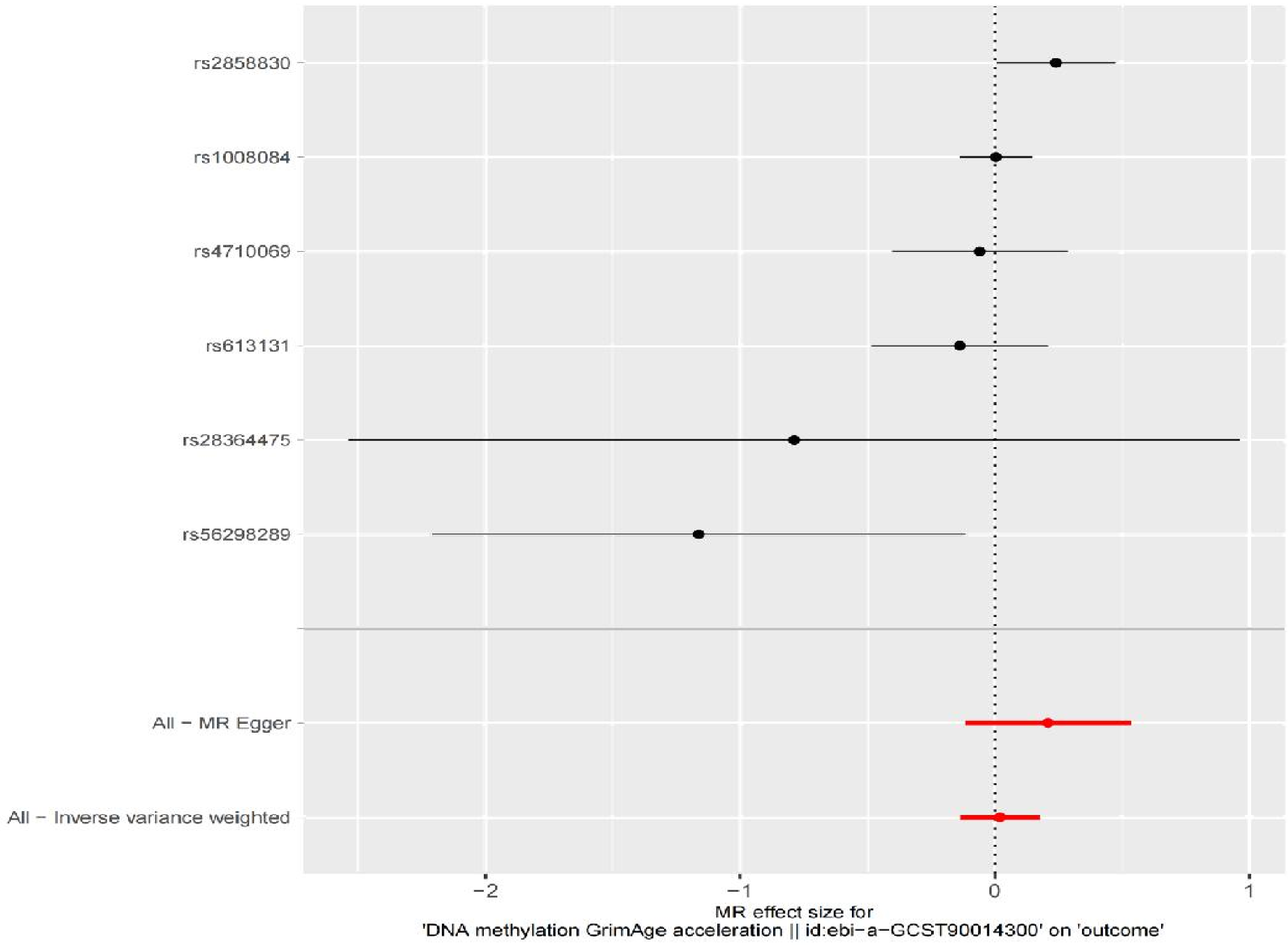

Supplementary Figure-34D Funnel Plot

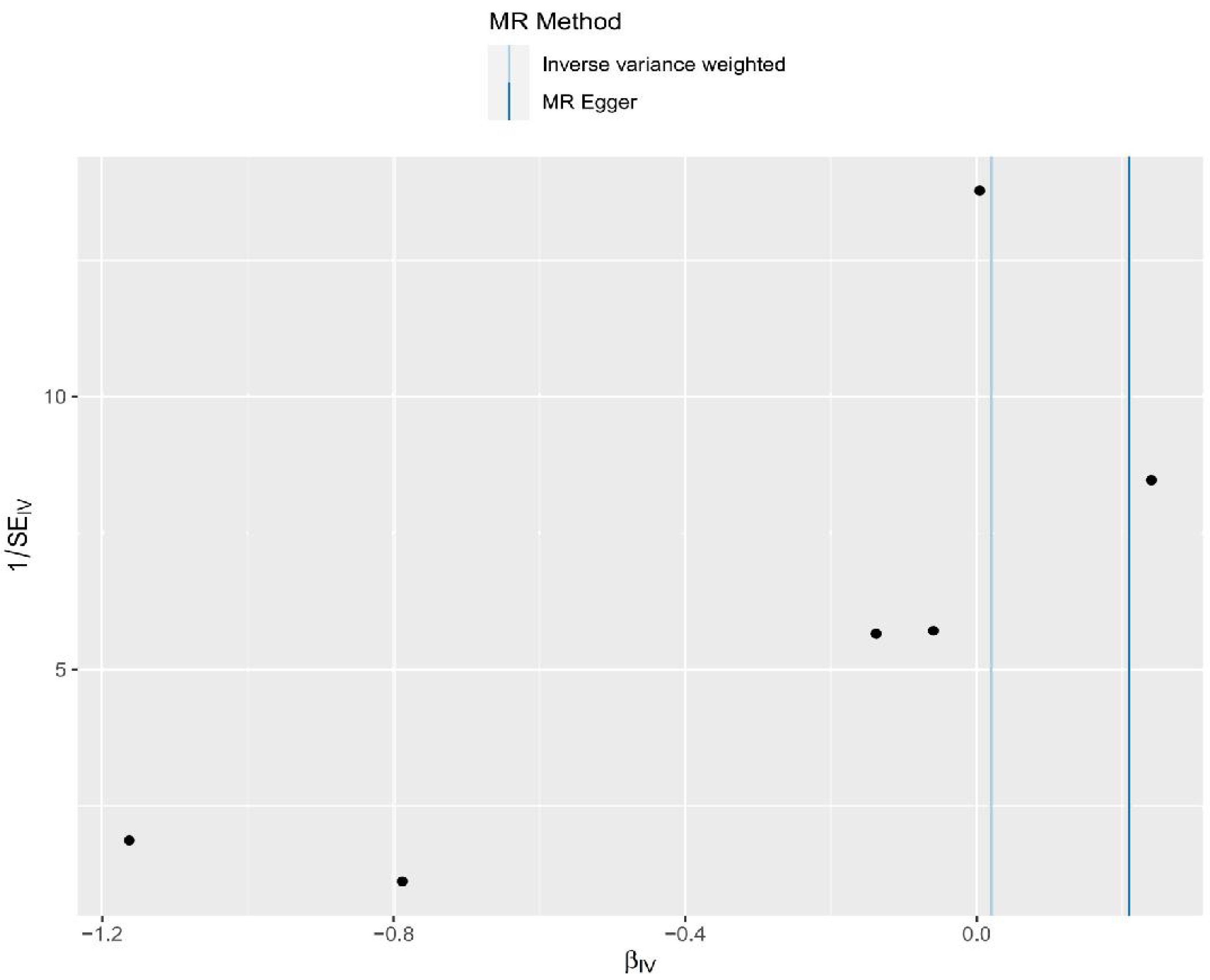

Supplementary Figure-35 Leave-one-out Analysis, Scatter Plot, Forest Plot, and Funnel Plot of DNA methylation GrimAge Acceleration on Amyotrophic Lateral Sclerosis

Supplementary Figure-35A Leave-one-out Analysis

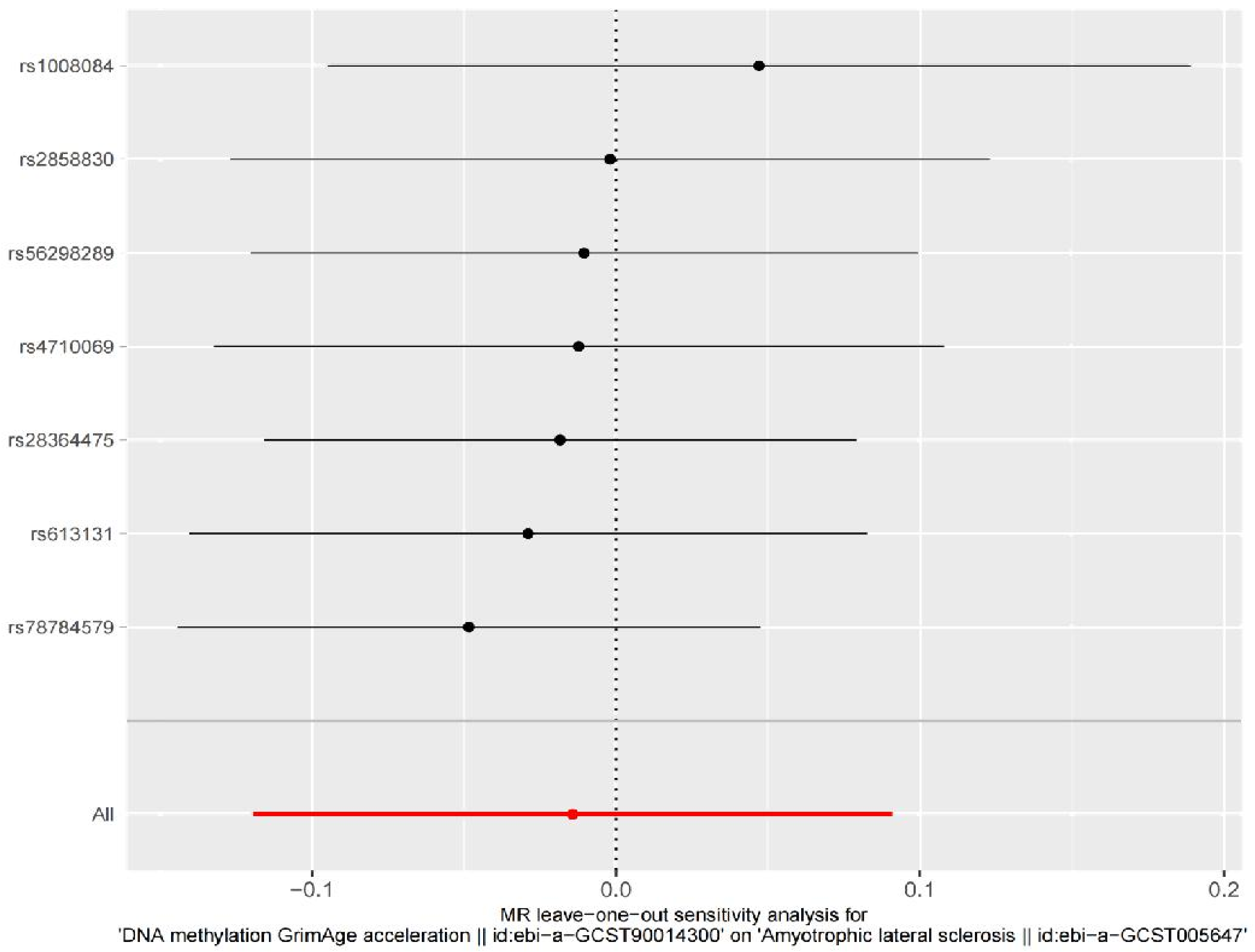

Supplementary Figure-35B Scatter

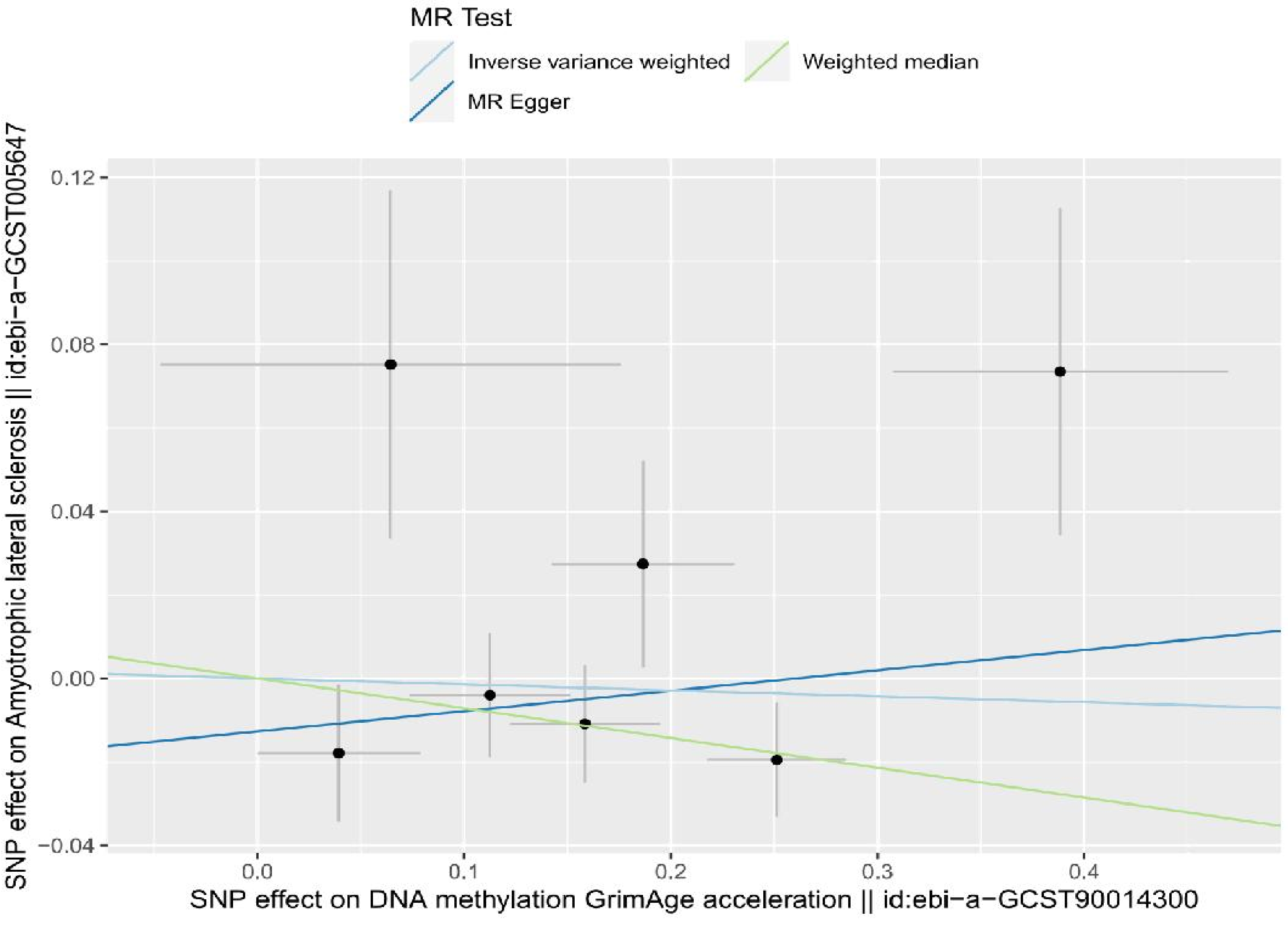

Supplementary Figure-35C Forest Plot

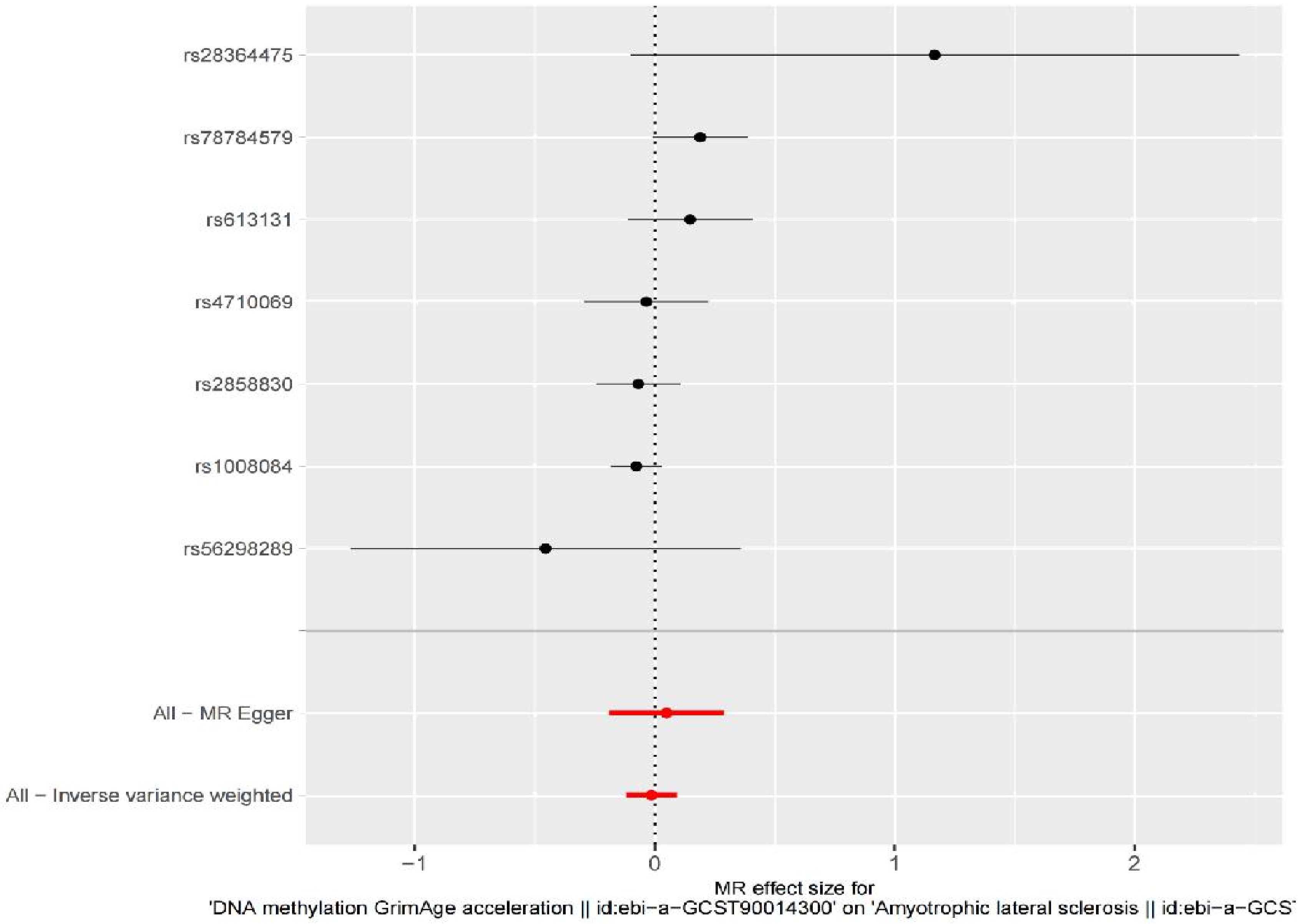

Supplementary Figure-35D Funnel Plot

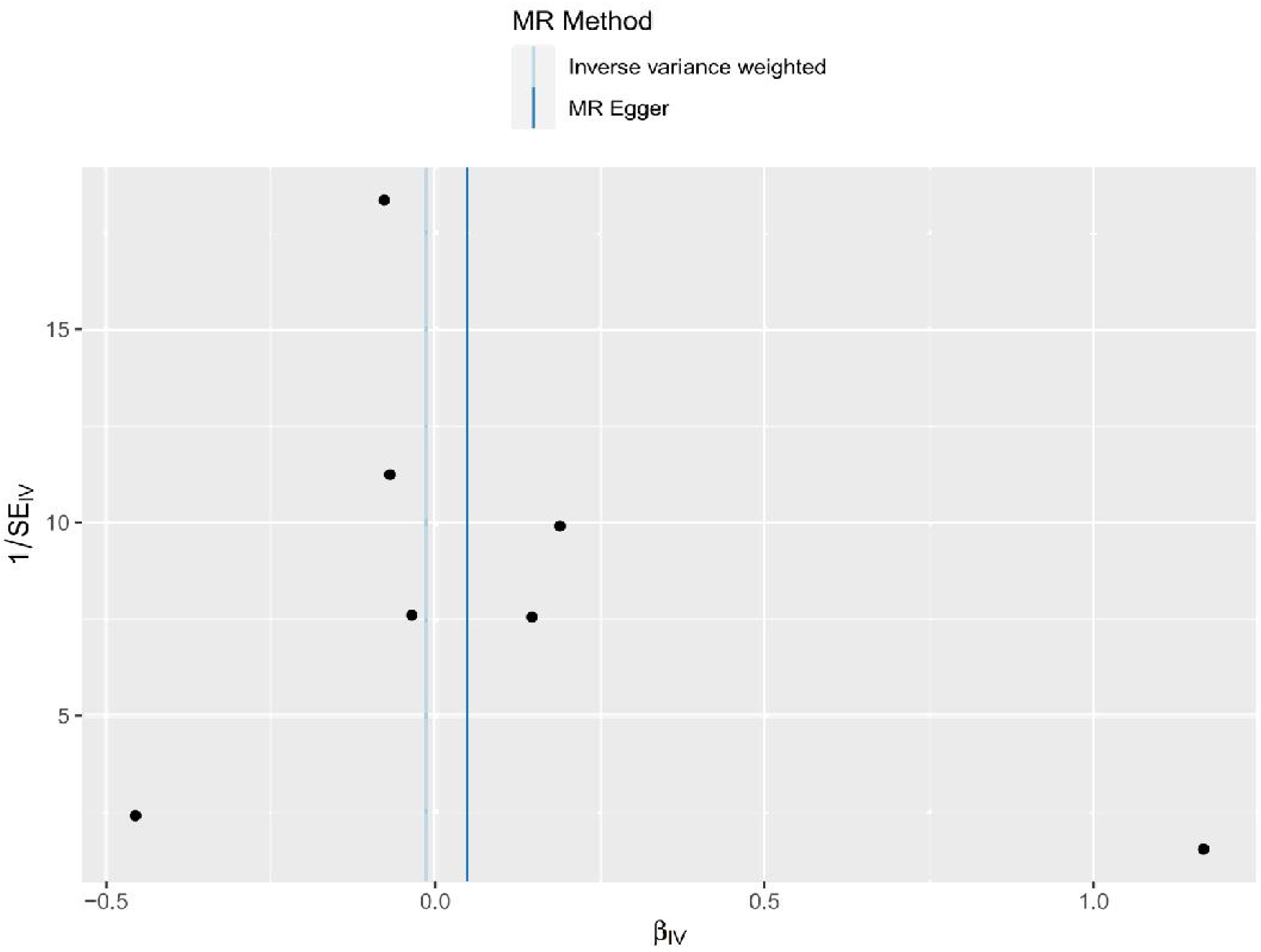

Supplementary Figure-36 Leave-one-out Analysis, Scatter Plot, Forest Plot, and Funnel Plot of DNA methylation GrimAge Acceleration on GBM

Supplementary Figure-36A Leave-one-out Analysis

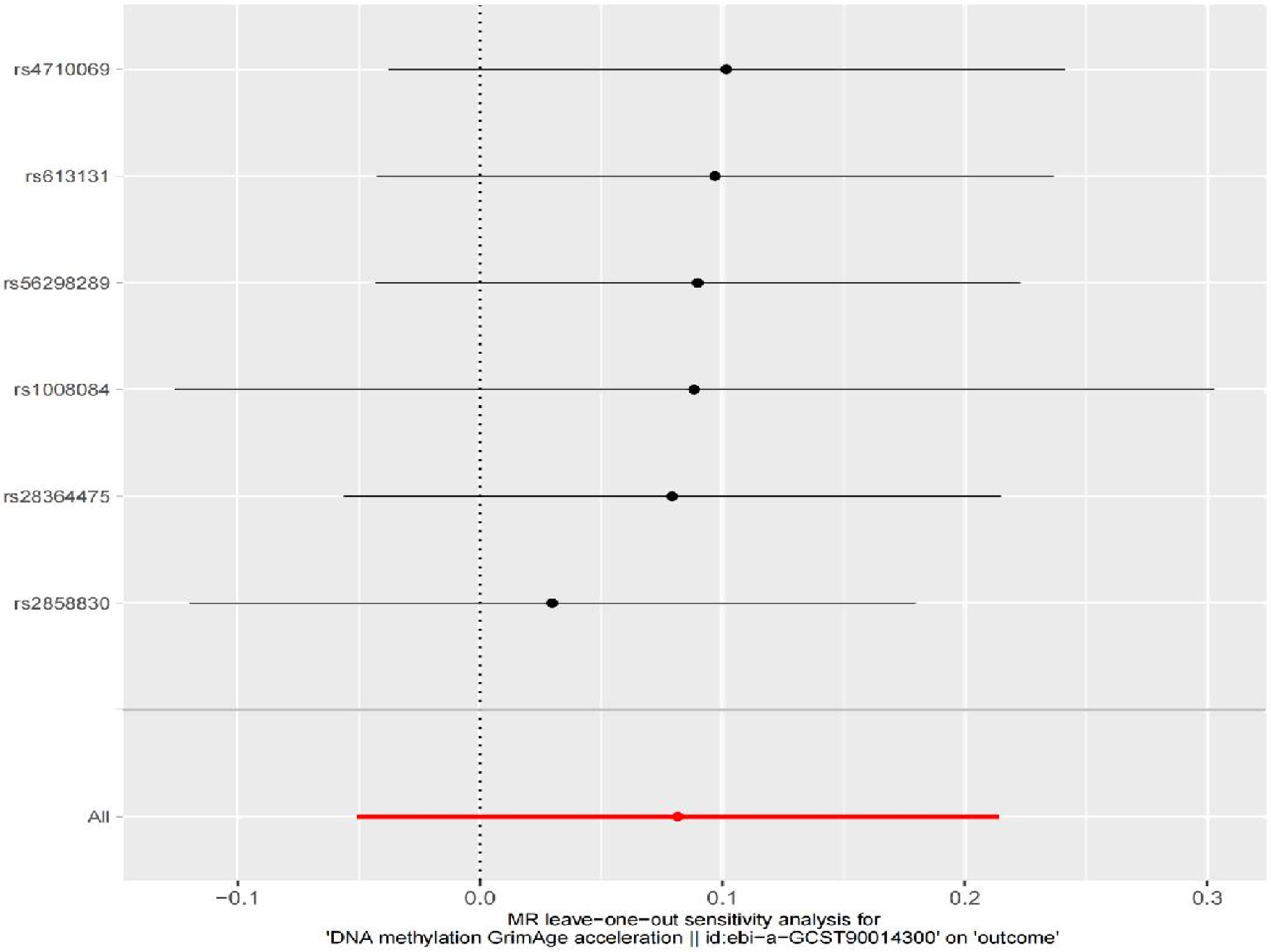

Supplementary Figure-36B Scatter

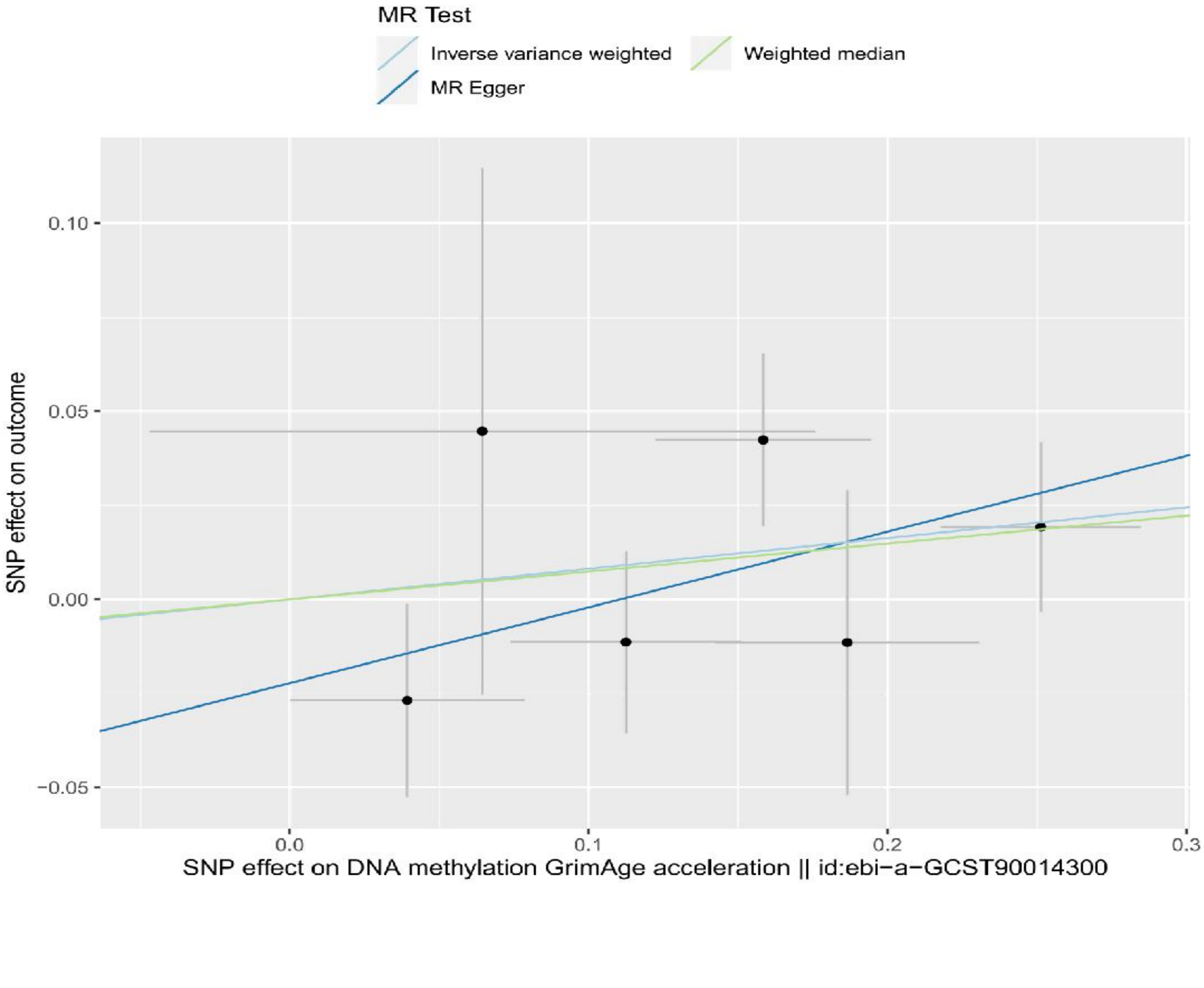

Supplementary Figure-36C Forest Plot

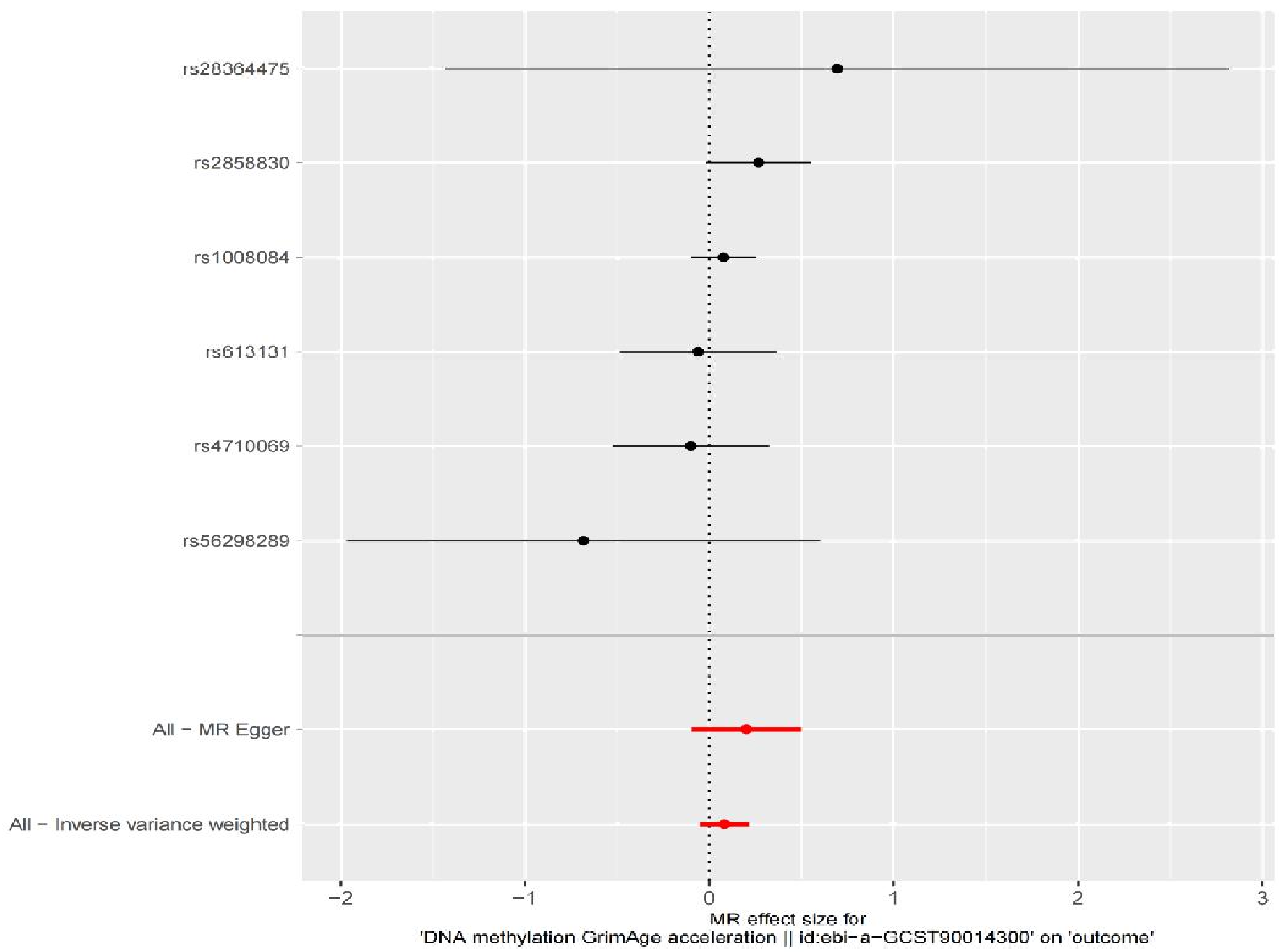

Supplementary Figure-36D Funnel Plot

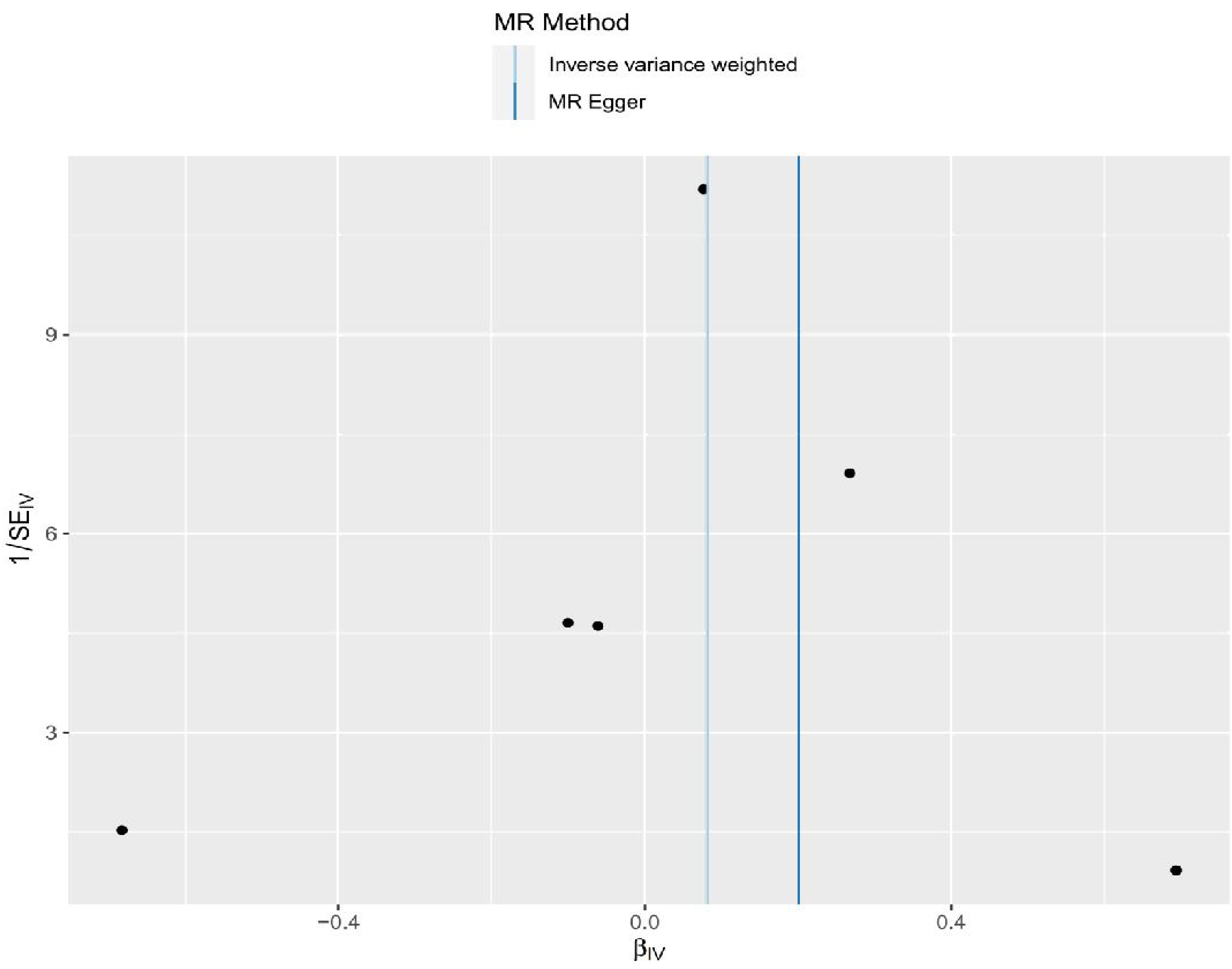

Supplementary Figure-37 Leave-one-out Analysis, Scatter Plot, Forest Plot, and Funnel Plot of DNA methylation GrimAge Acceleration on Meningioma

Supplementary Figure-37A Leave-one-out Analysis

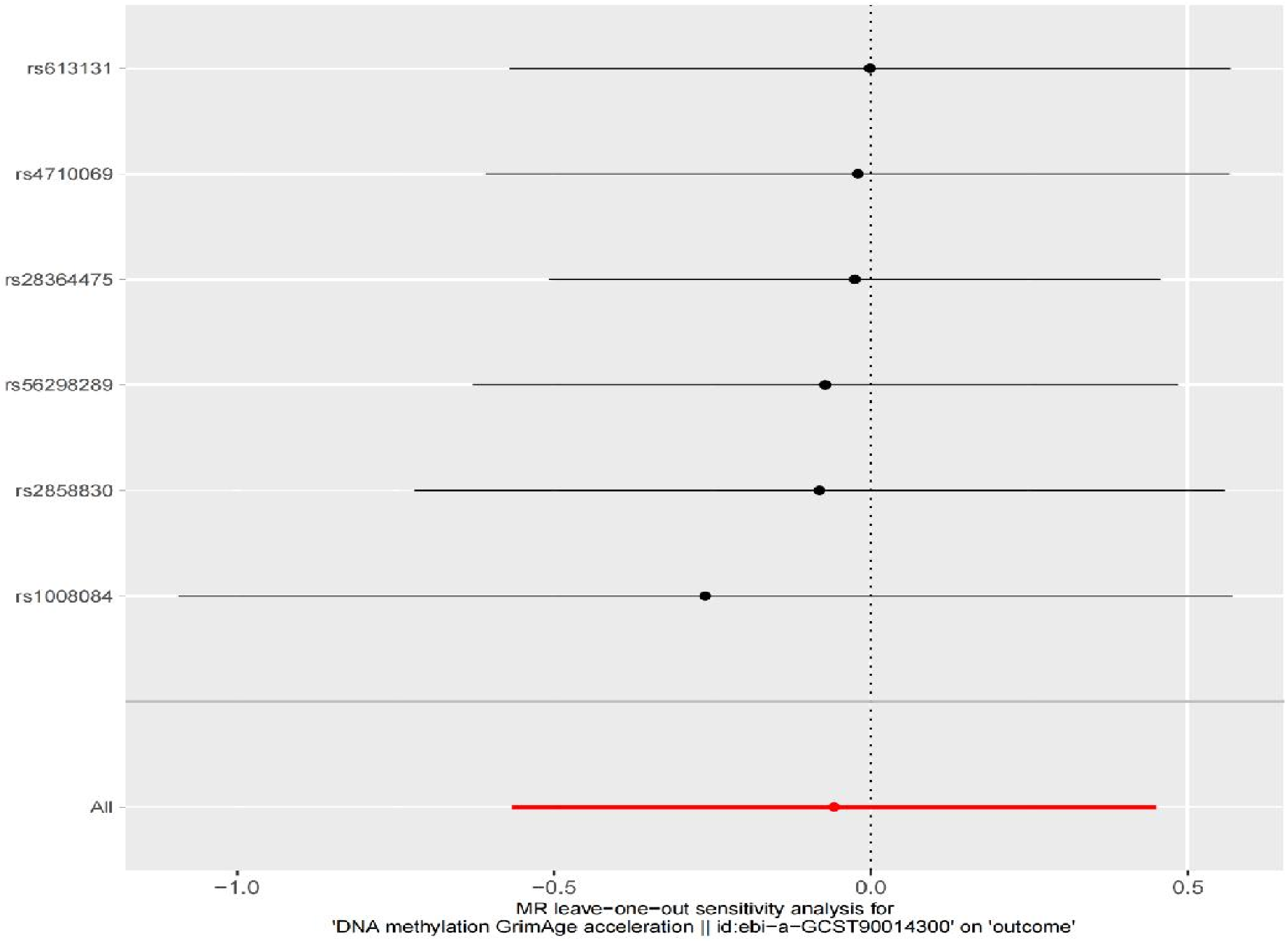

Supplementary Figure-37B Scatter

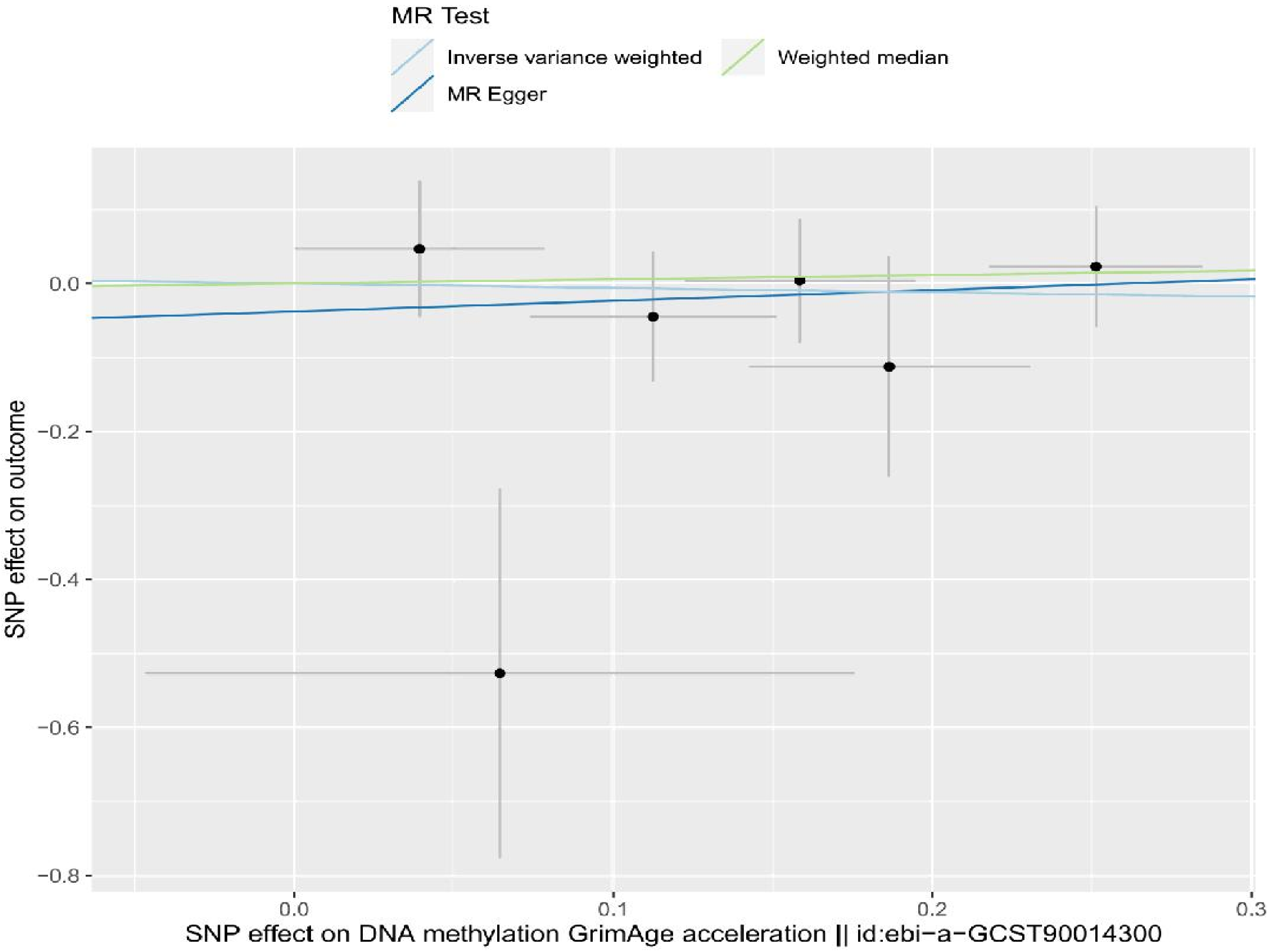

Supplementary Figure-37C Forest Plot

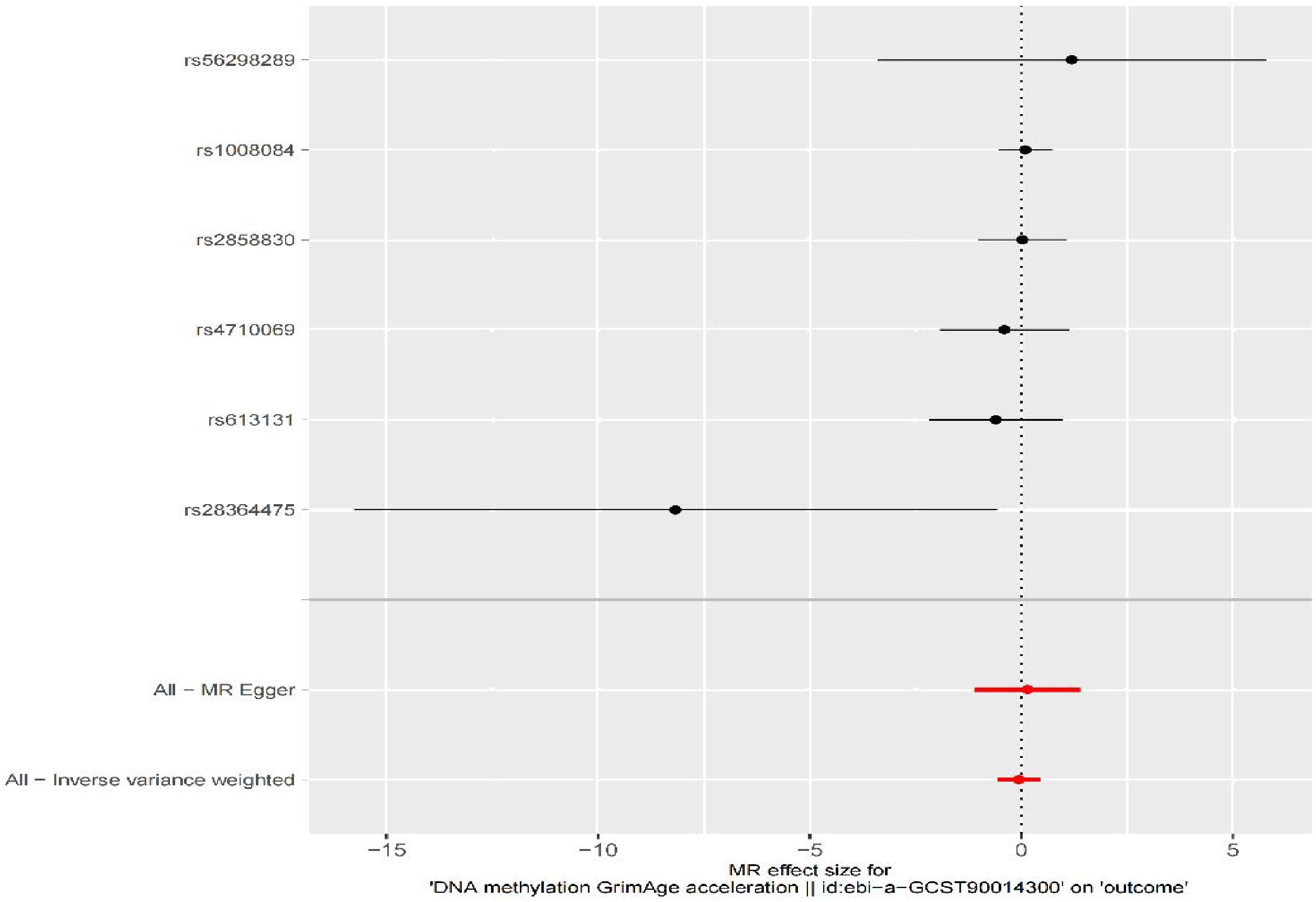

Supplementary Figure-37D Funnel Plot

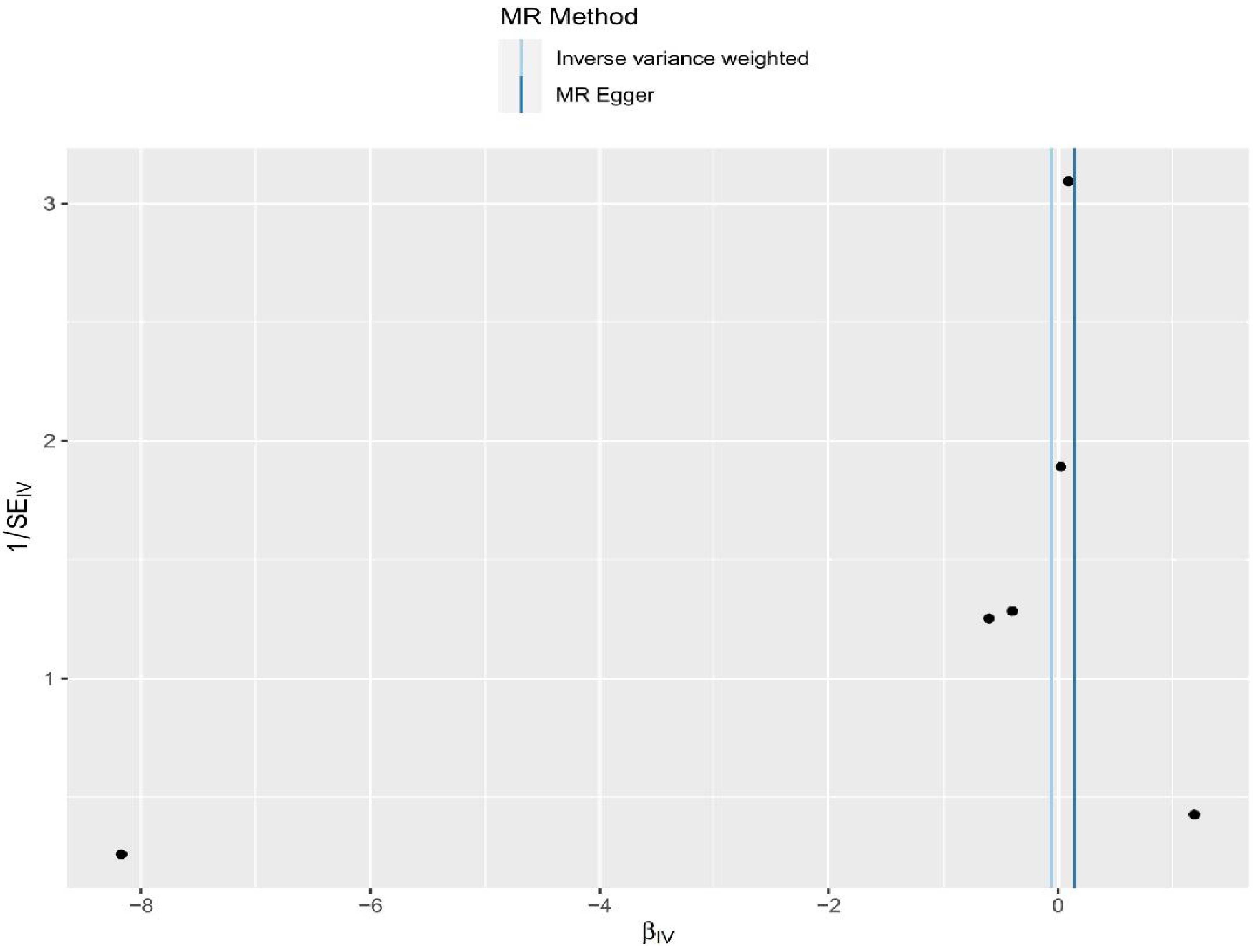

Supplementary Figure-38 Leave-one-out Analysis, Scatter Plot, Forest Plot, and Funnel Plot of DNA methylation GrimAge Acceleration on Non-GBM

Supplementary Figure-38A Leave-one-out Analysis

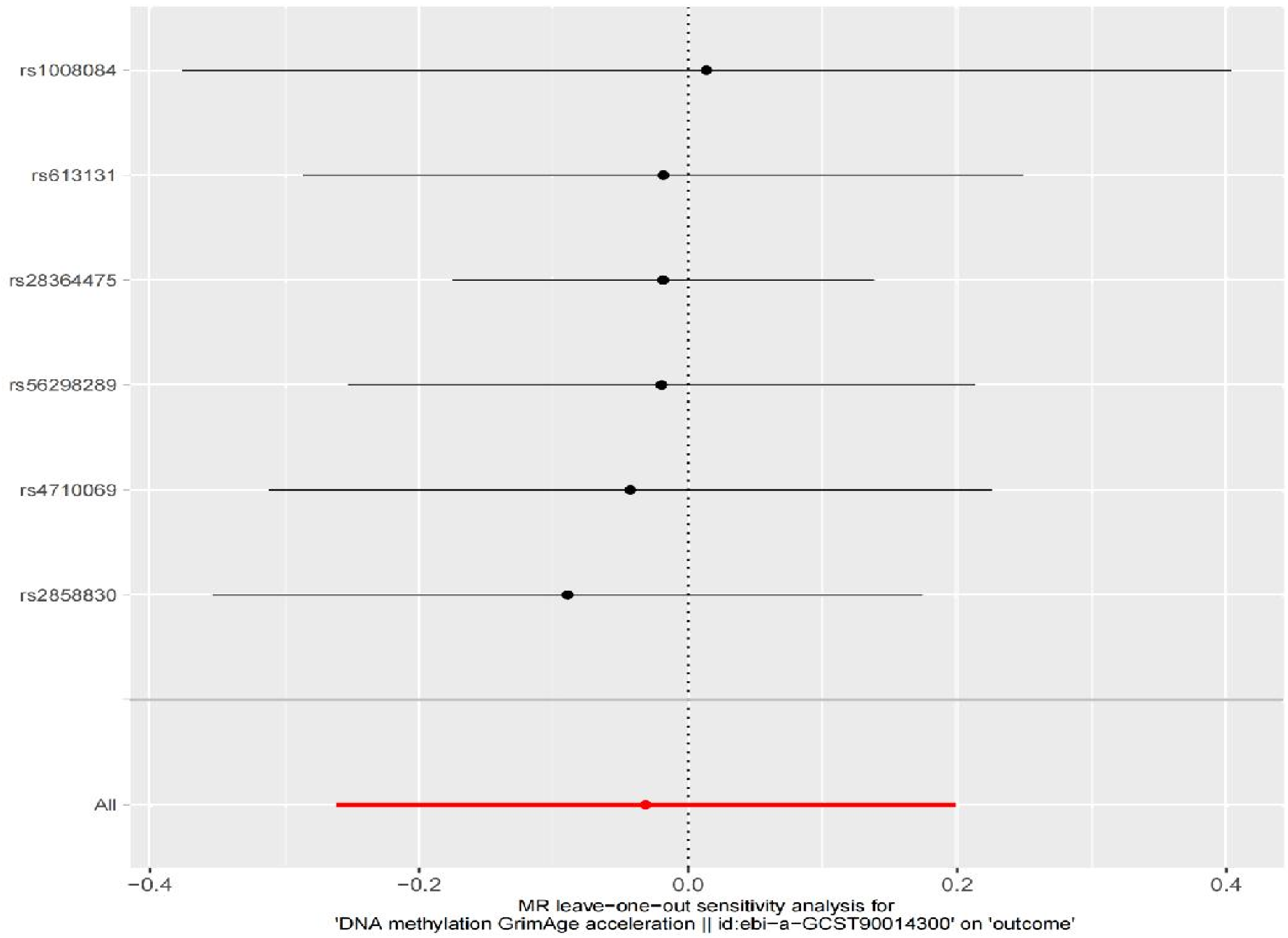

Supplementary Figure-38B Scatter

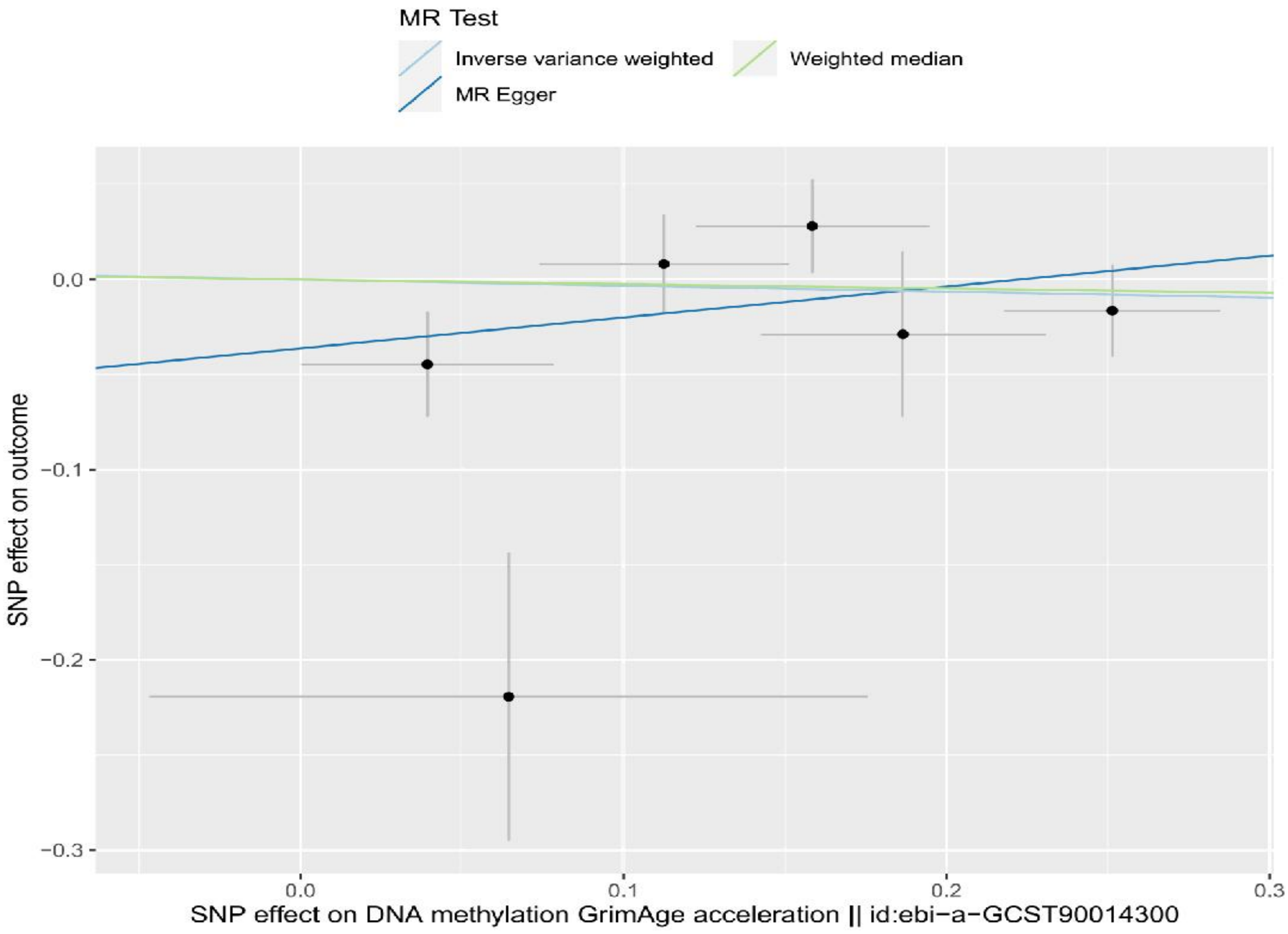

Supplementary Figure-38C Forest Plot

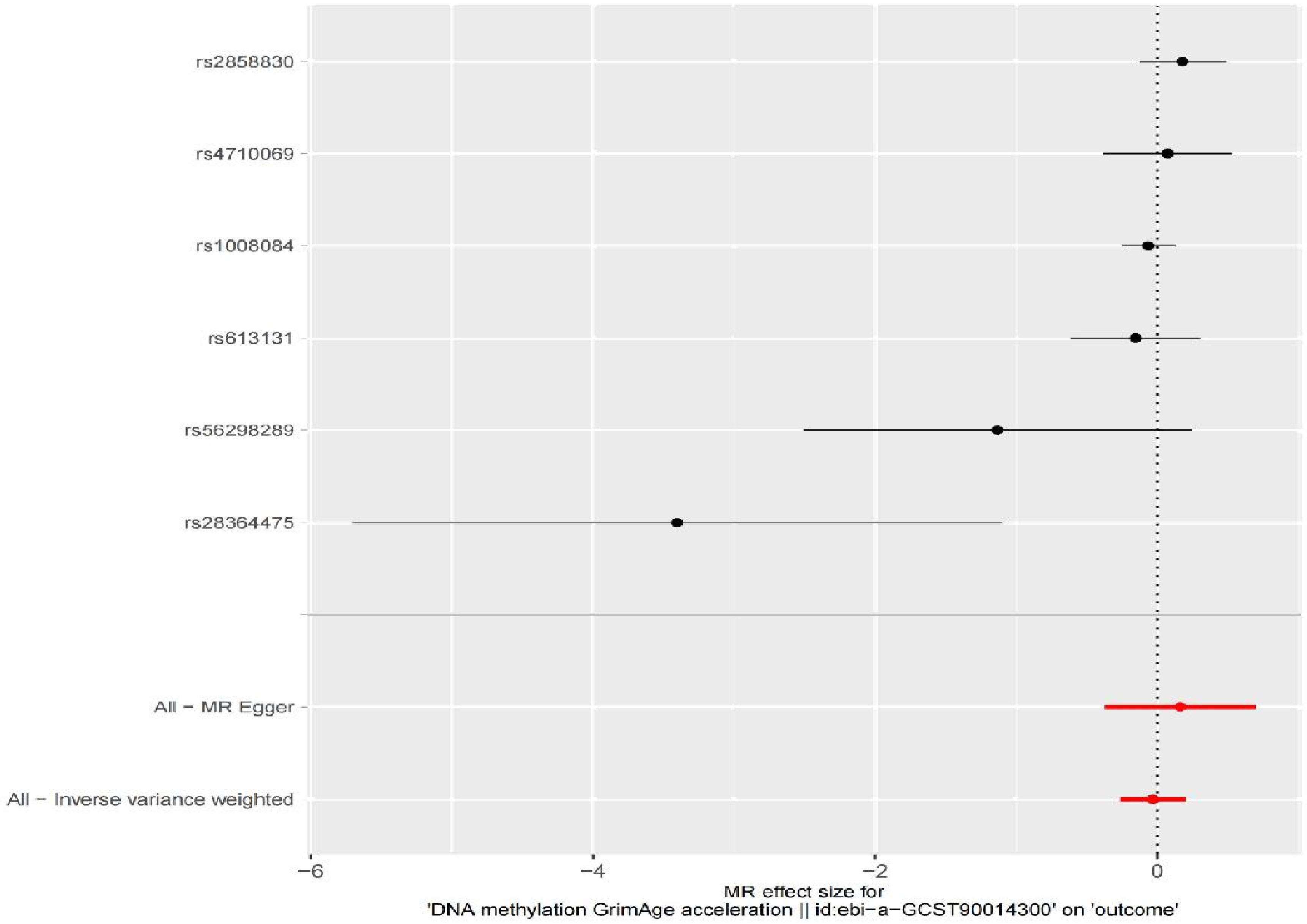

Supplementary Figure-38D Funnel Plot

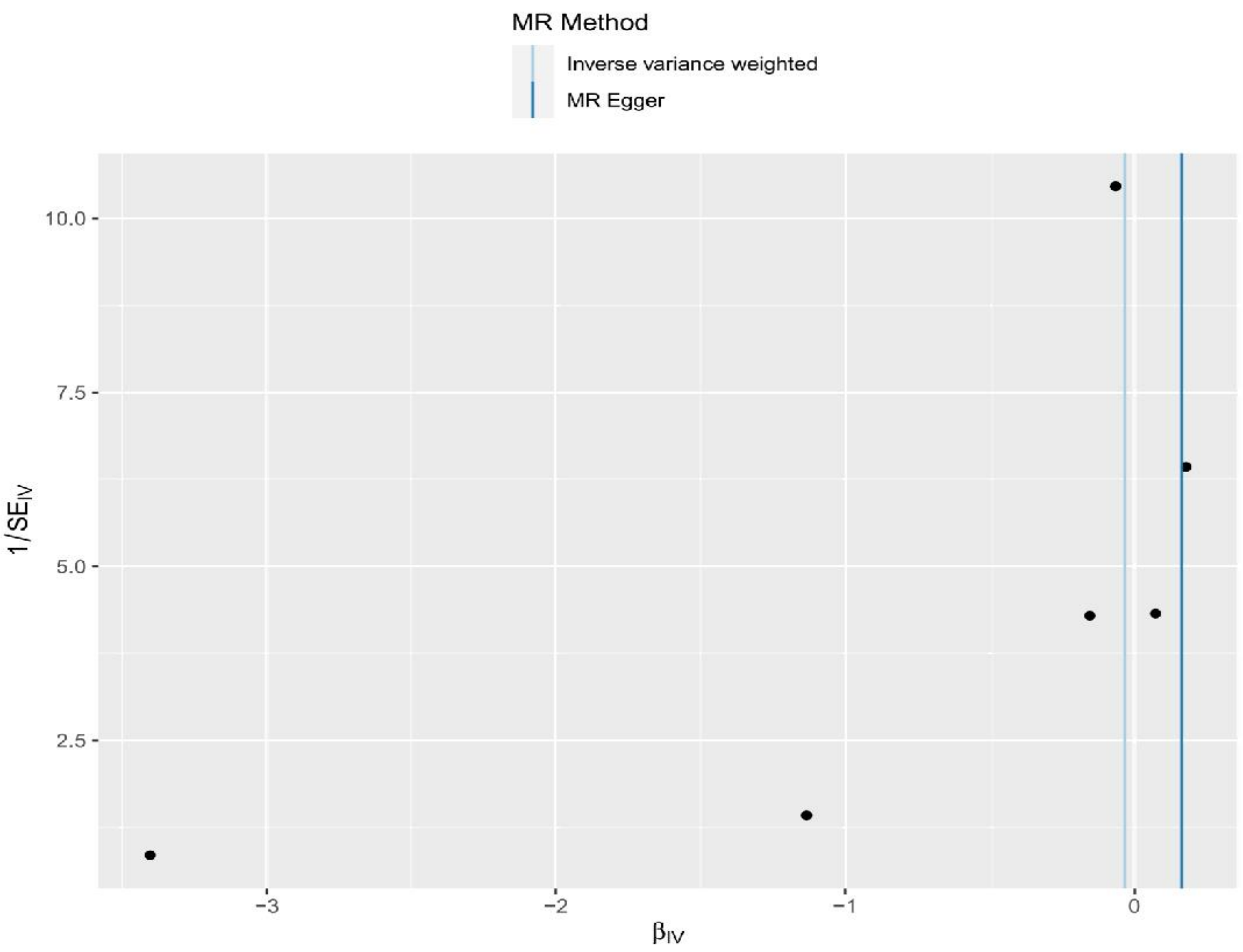

Supplementary Figure-39 Leave-one-out Analysis, Scatter Plot, Forest Plot, and Funnel Plot of DNA methylation GrimAge Acceleration on Parkinson

Supplementary Figure-39A Leave-one-out Analysis

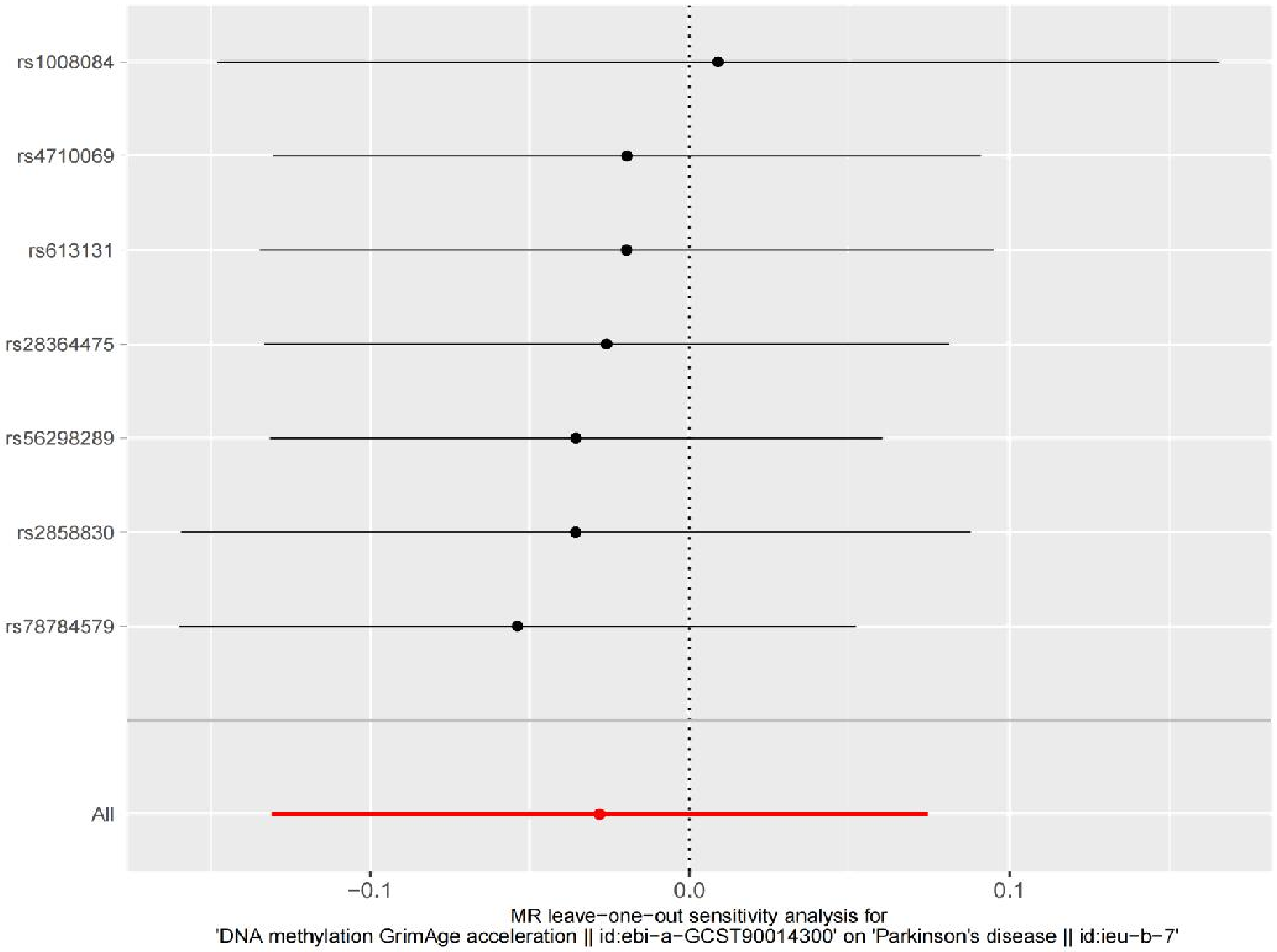

Supplementary Figure-39B Scatter

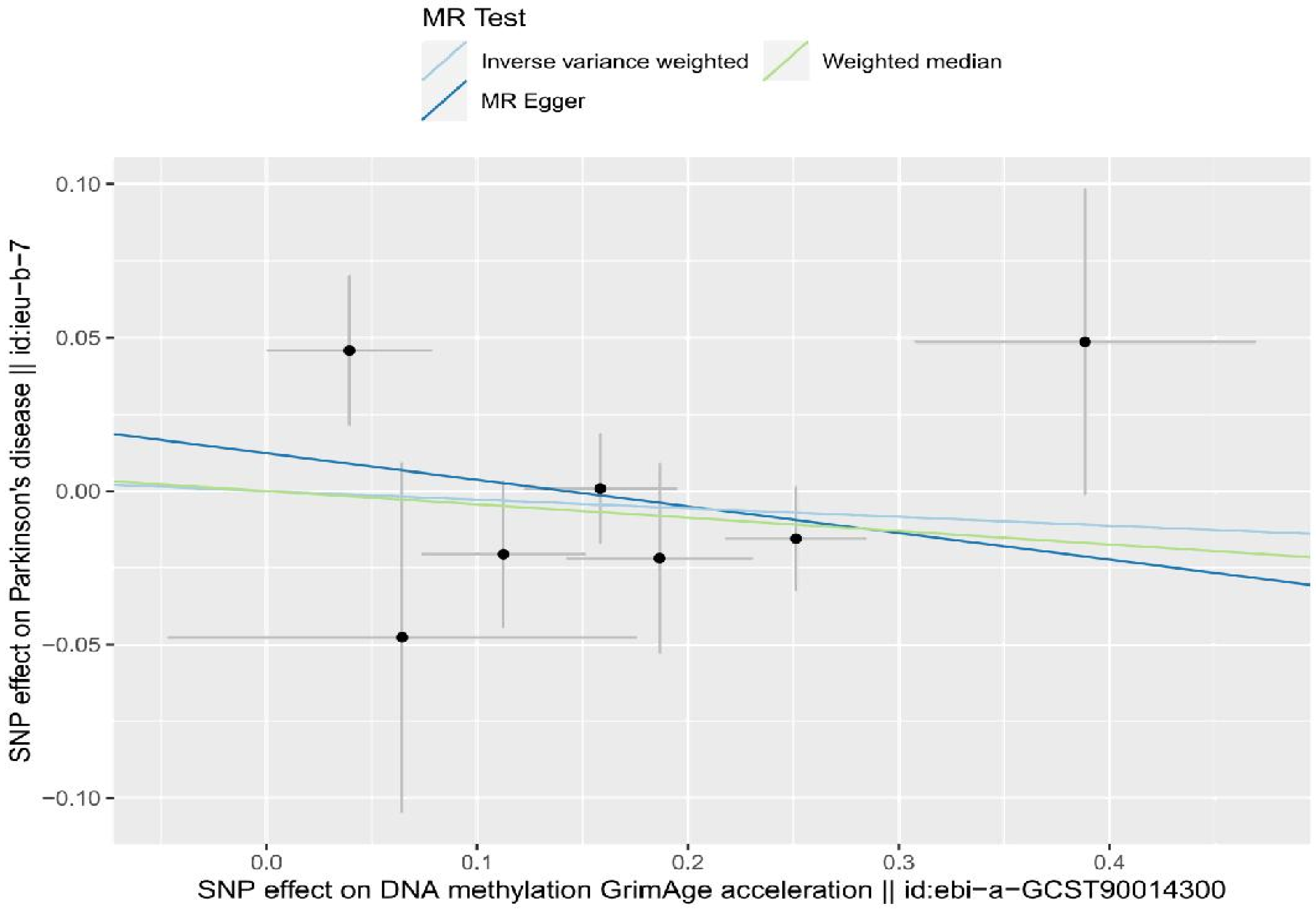

Supplementary Figure-39C Forest Plot

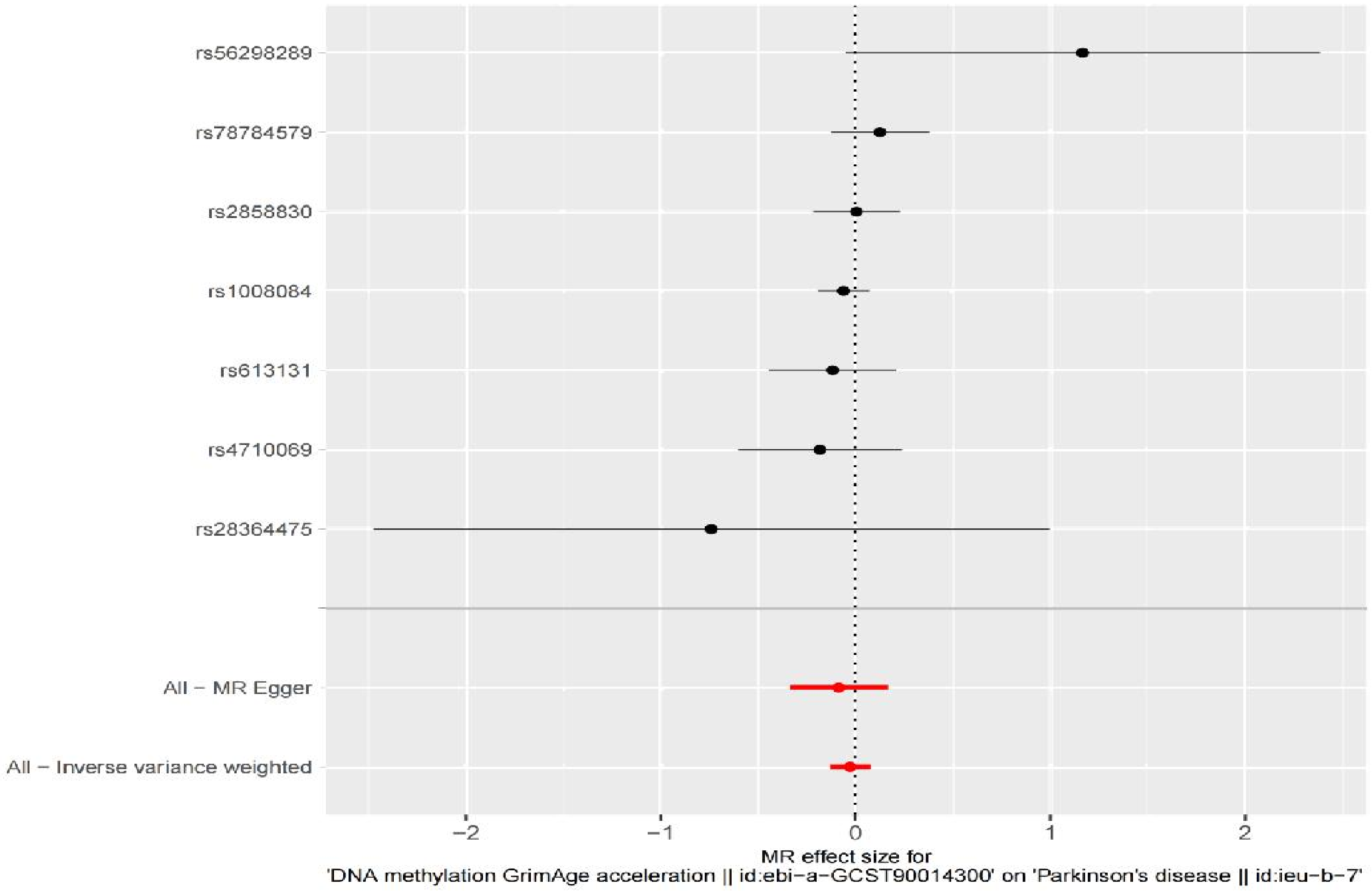

Supplementary Figure-39D Funnel Plot

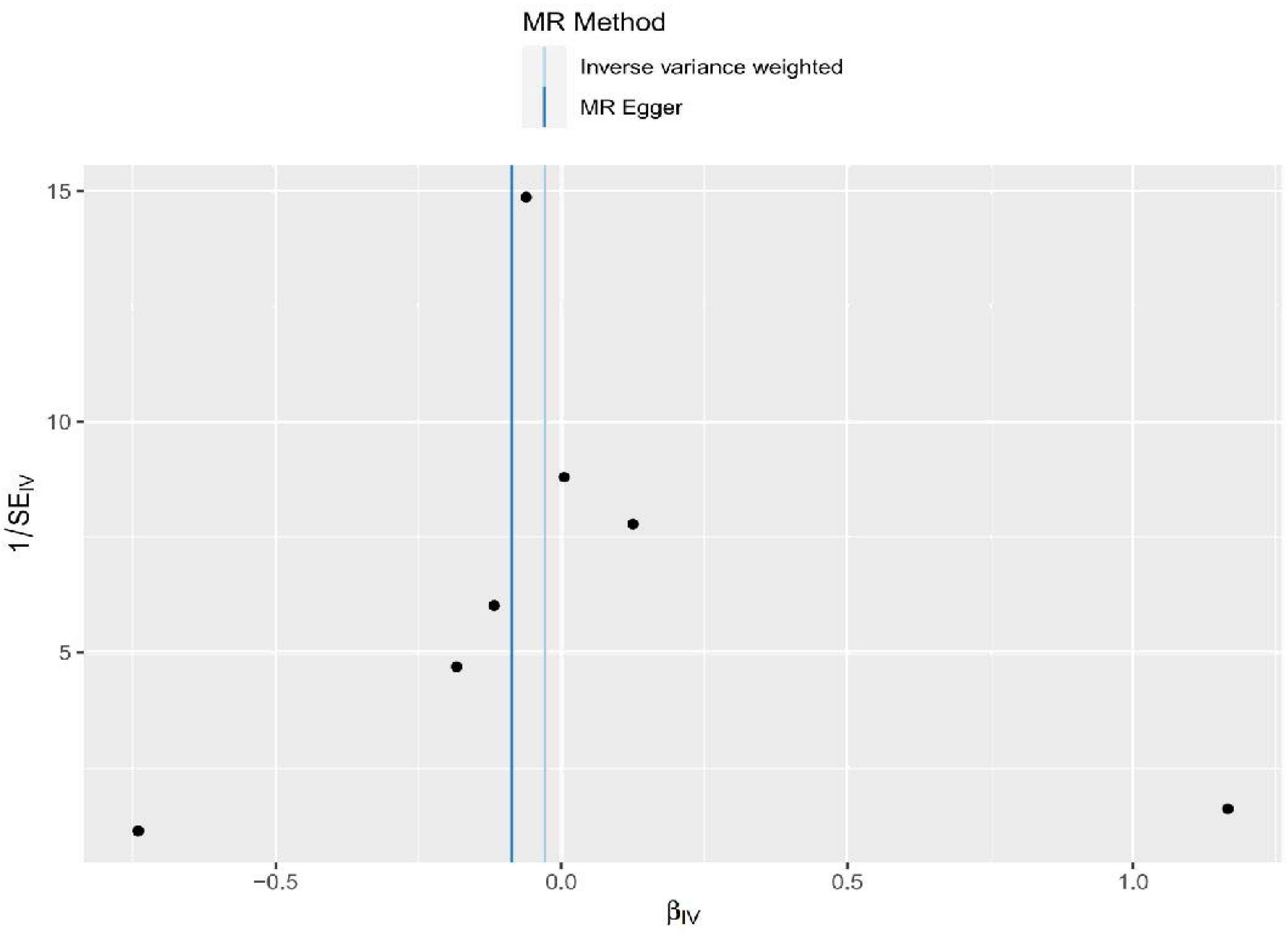

Supplementary Figure-40 Leave-one-out Analysis, Scatter Plot, Forest Plot, and Funnel Plot of DNA methylation GrimAge Acceleration on Vestibular Schwannomas

Supplementary Figure-40A Leave-one-out Analysis

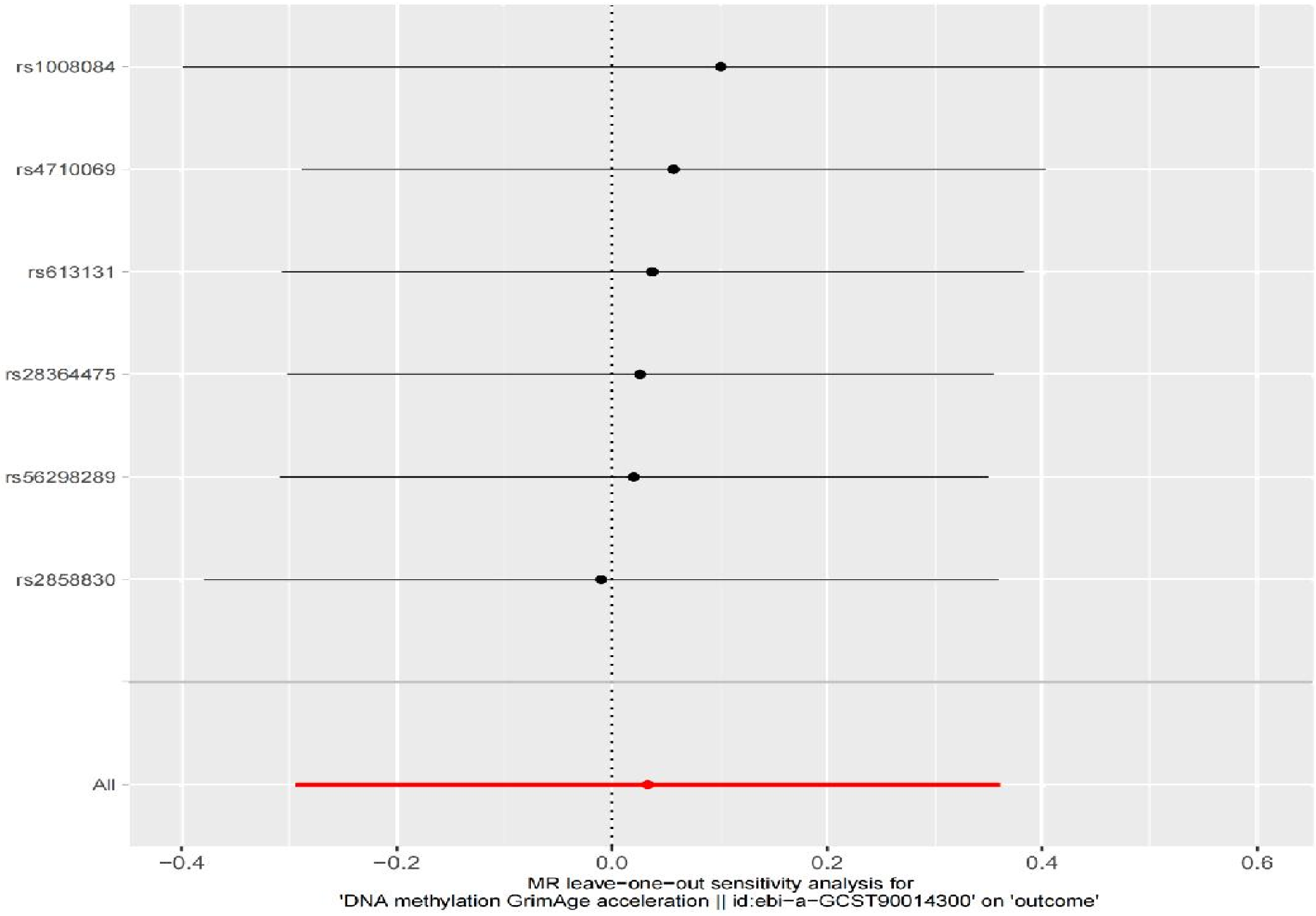

Supplementary Figure-40B Scatter

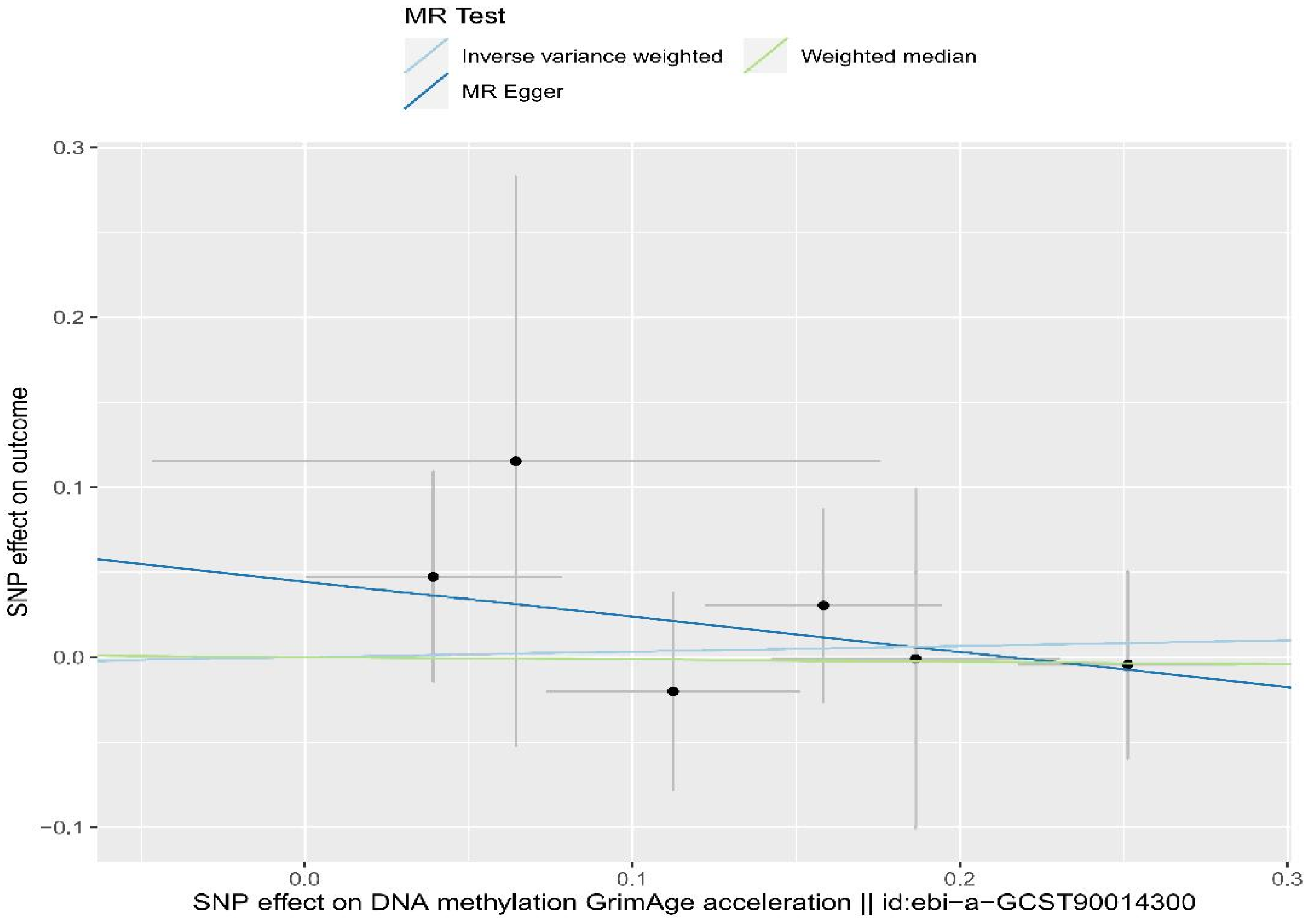

Supplementary Figure-40C Forest Plot

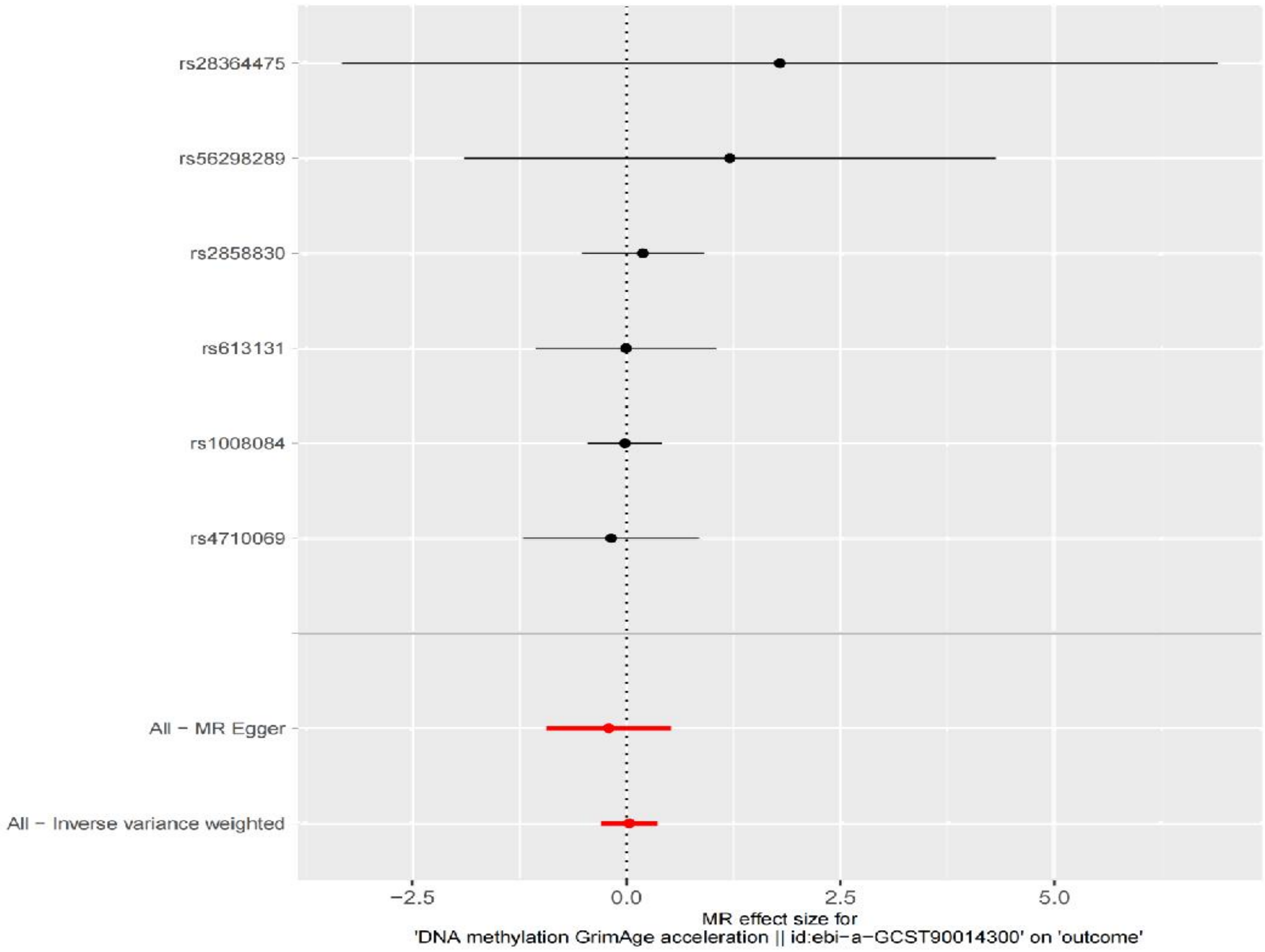

Supplementary Figure-40D Funnel Plot

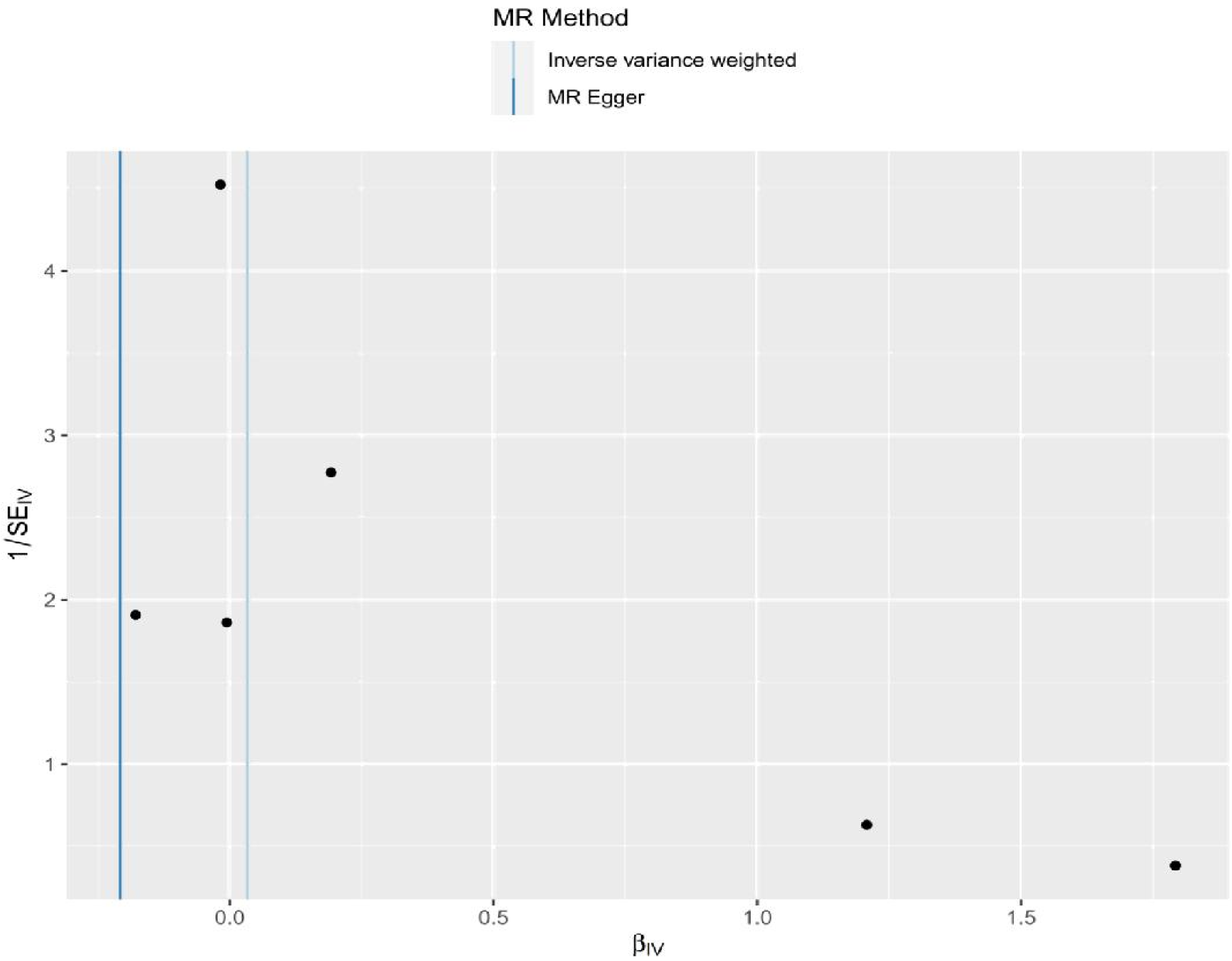

Supplementary Figure-41 Leave-one-out Analysis, Scatter Plot, Forest Plot, and Funnel Plot of Meningioma on Facial Ageing

Supplementary Figure-41A Leave-one-out Analysis

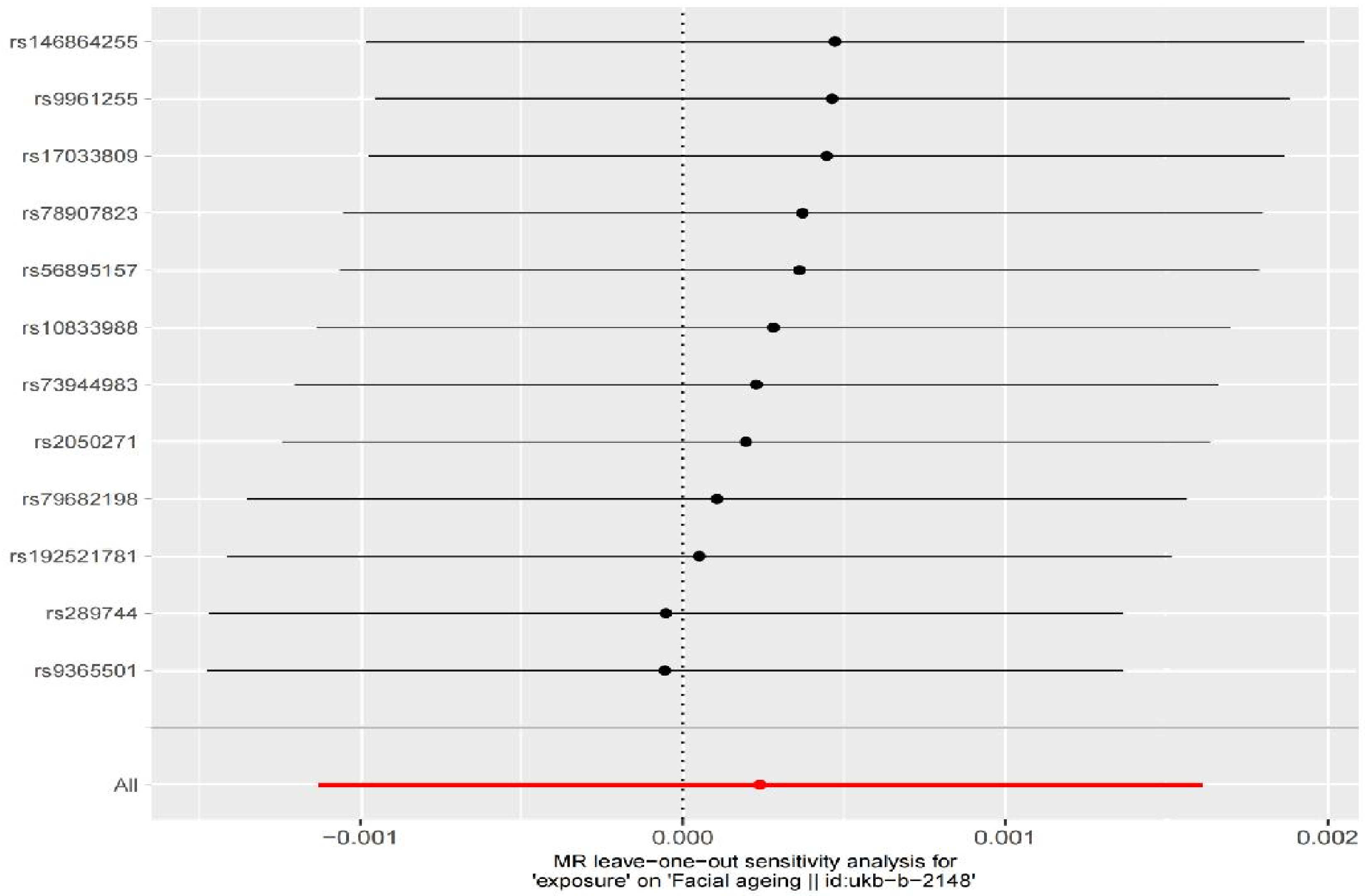

Supplementary Figure-41B Scatter

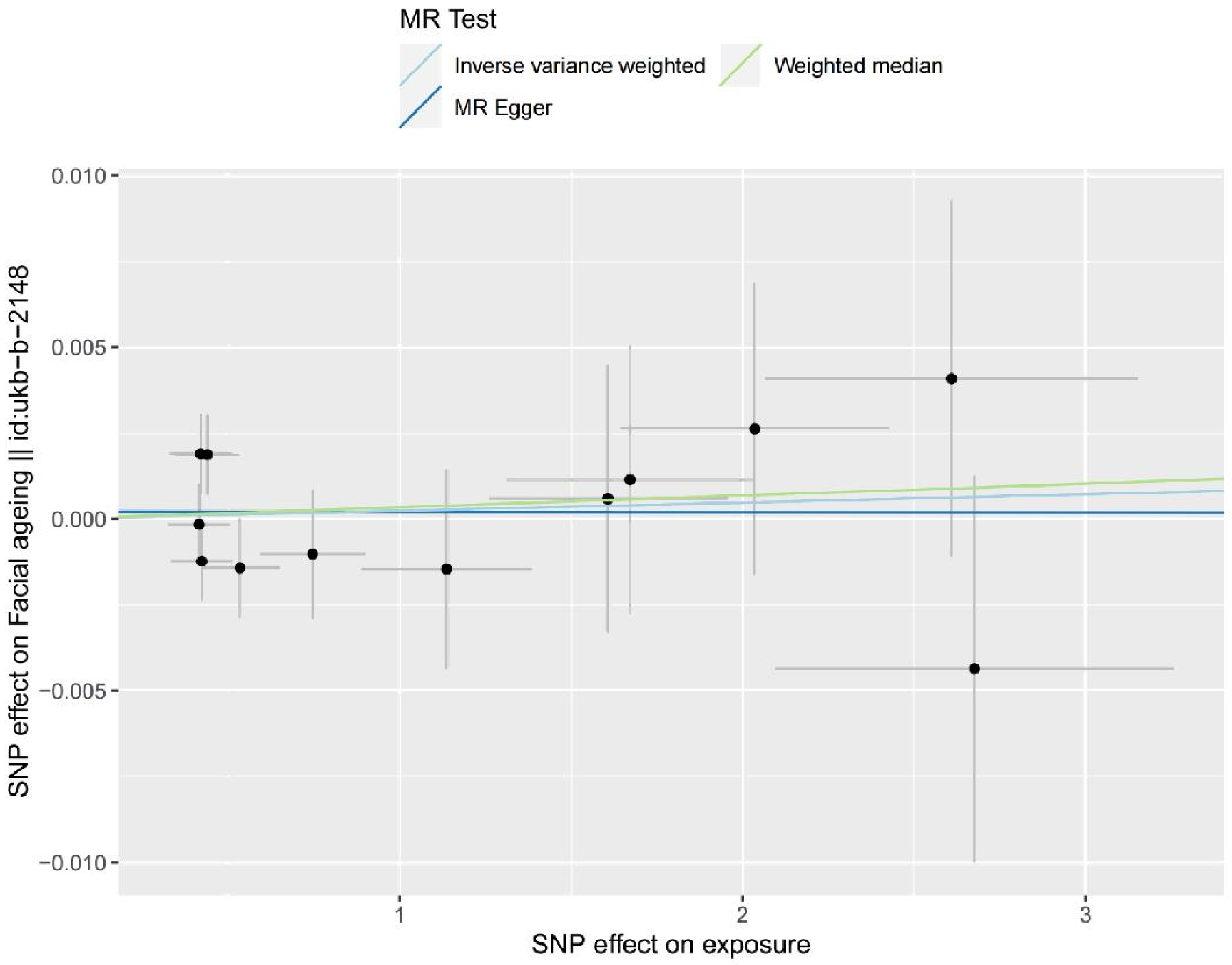

Supplementary Figure-41C Forest Plot

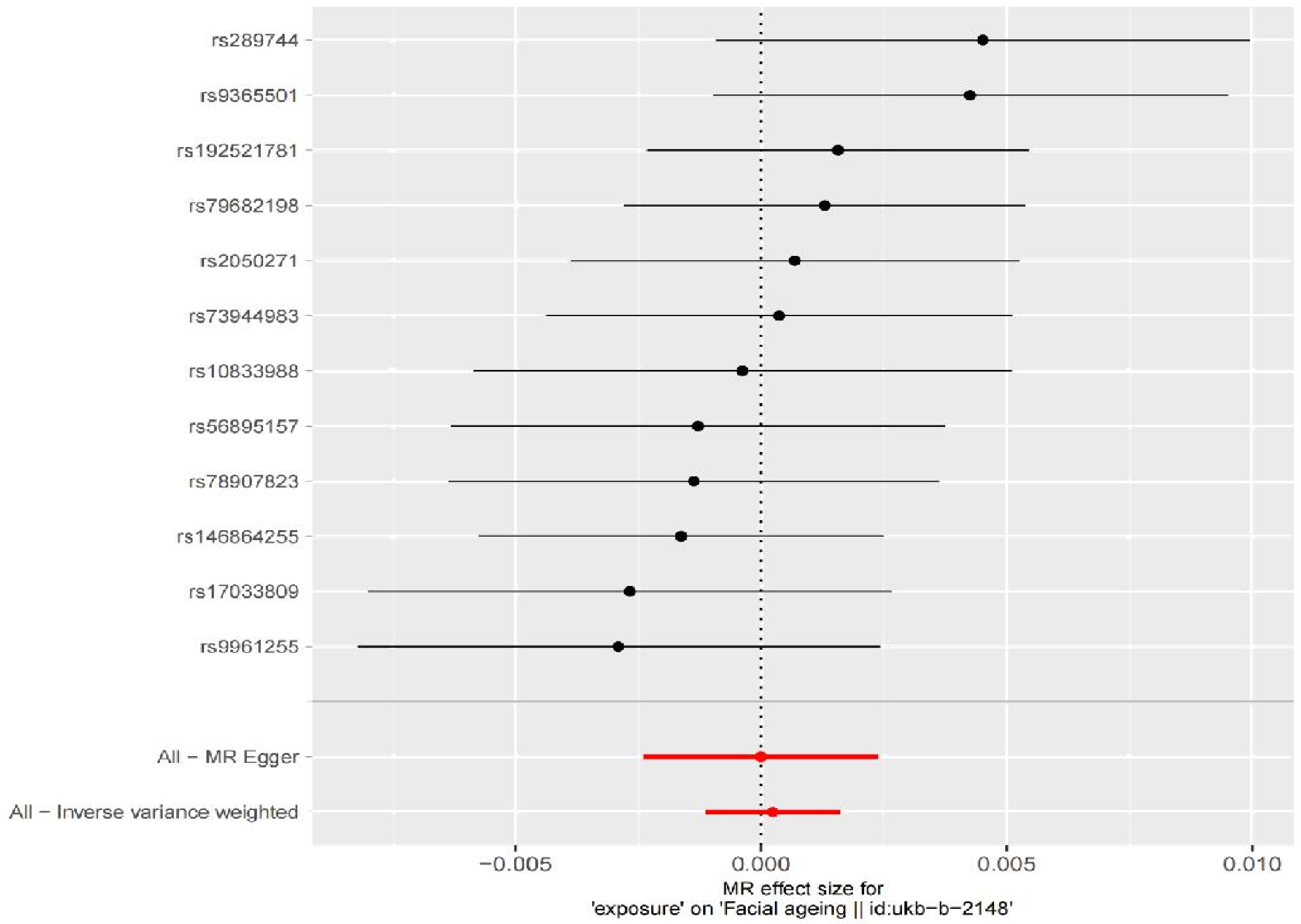

Supplementary Figure-41D Funnel Plot

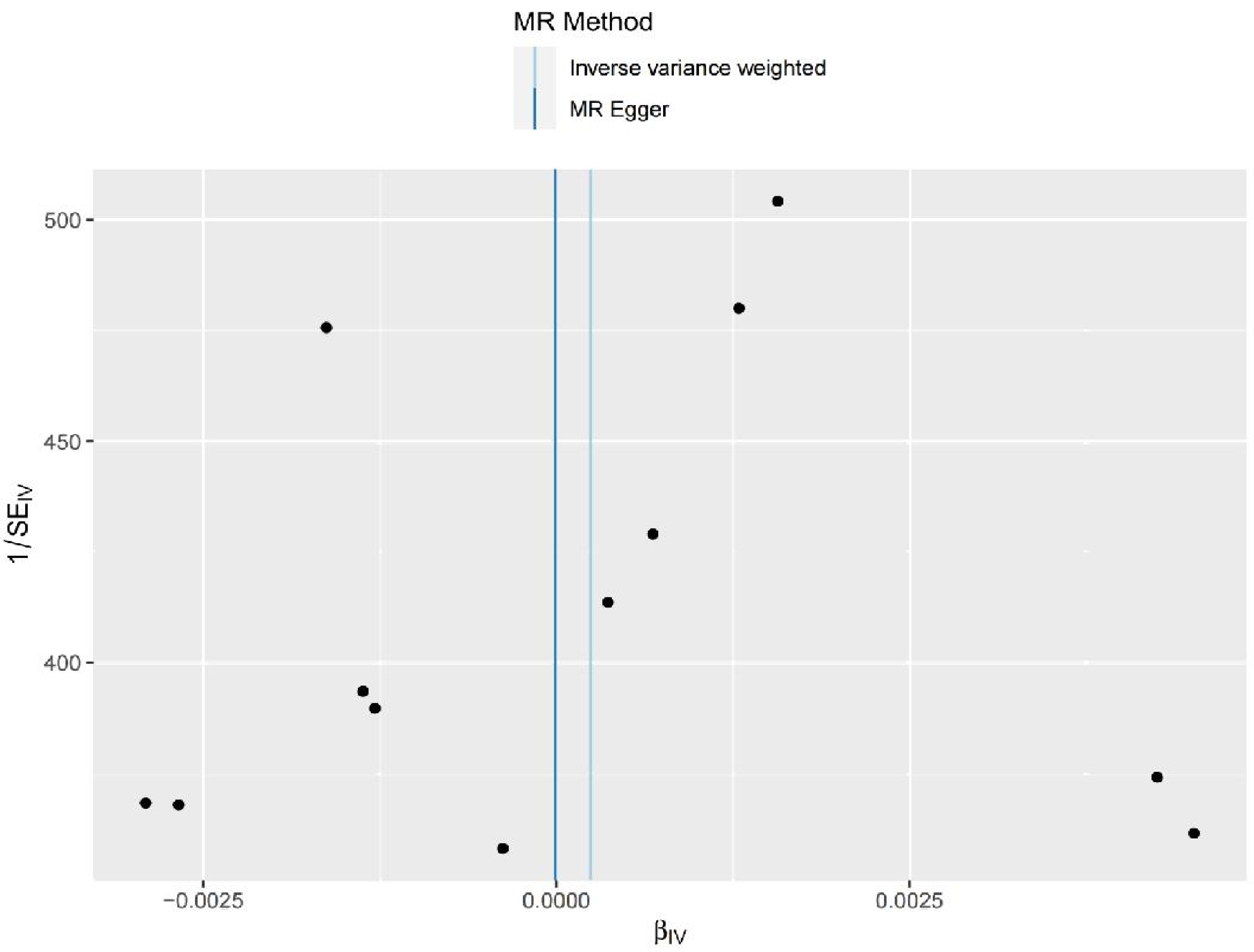

Supplementary Figure-42Leave-one-out Analysis, Scatter Plot, Forest Plot, and Funnel Plot of Meningioma on Frailty Index

Supplementary Figure-42A Leave-one-out Analysis

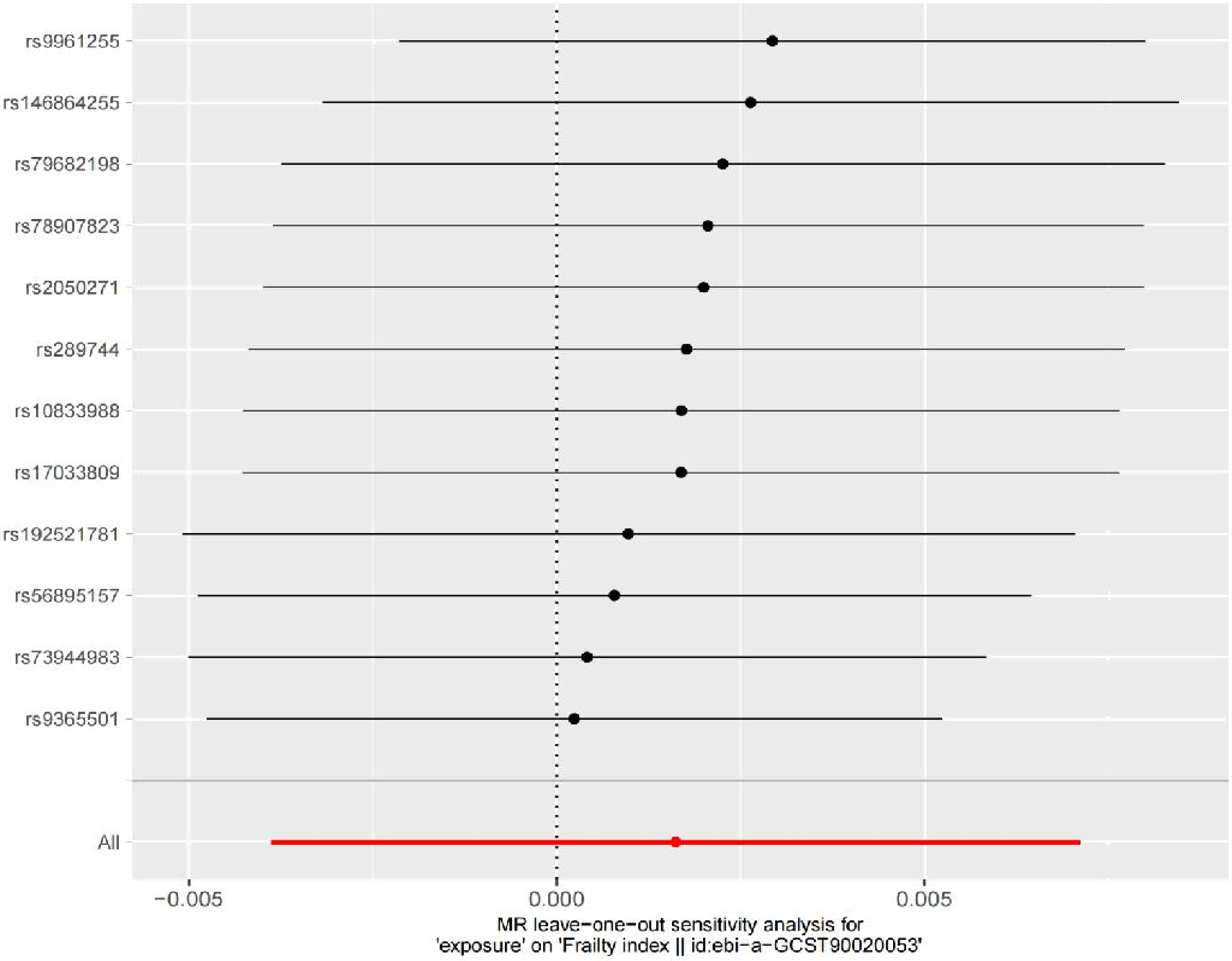

Supplementary Figure-42B Scatter

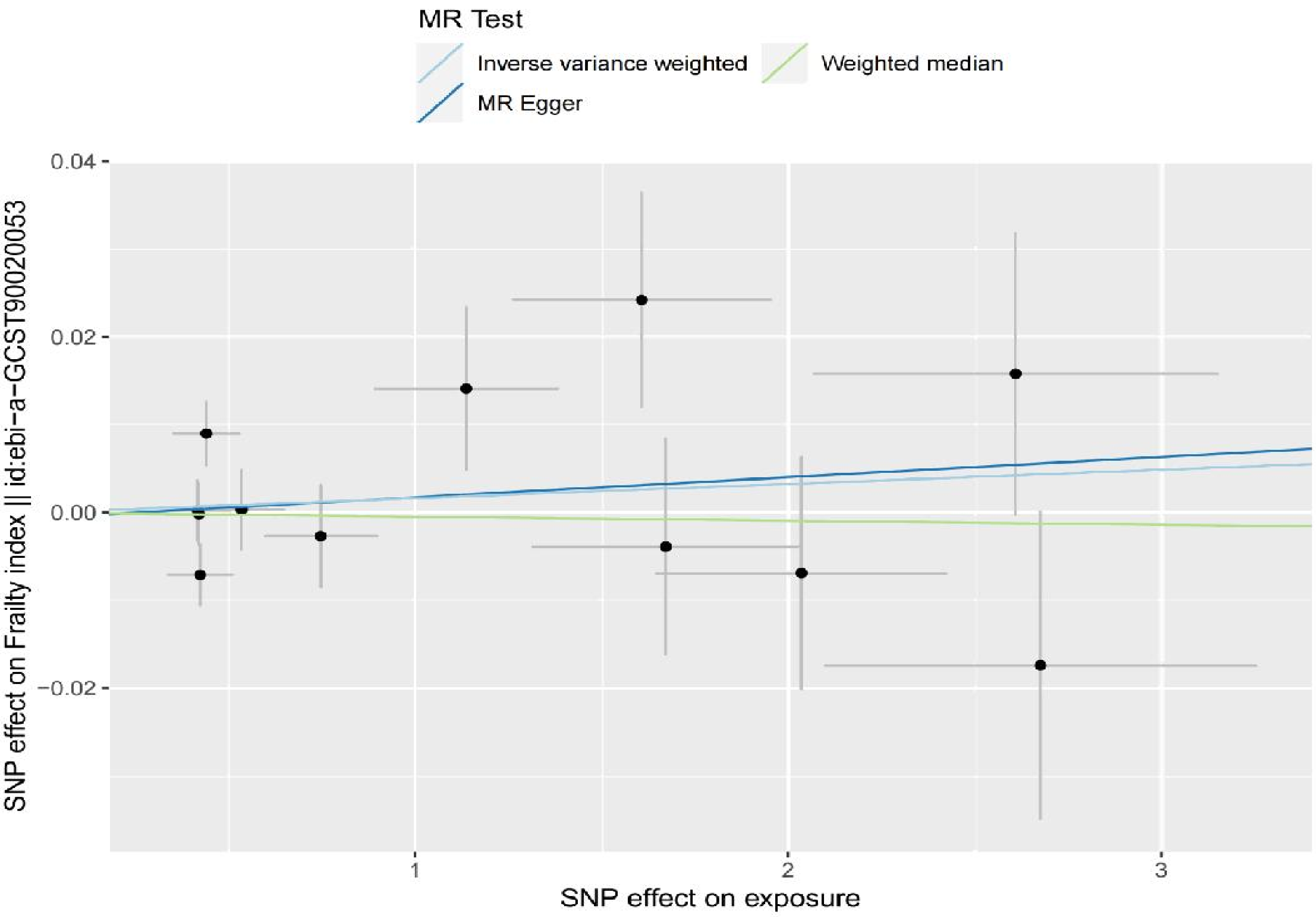

Supplementary Figure-42C Forest Plot

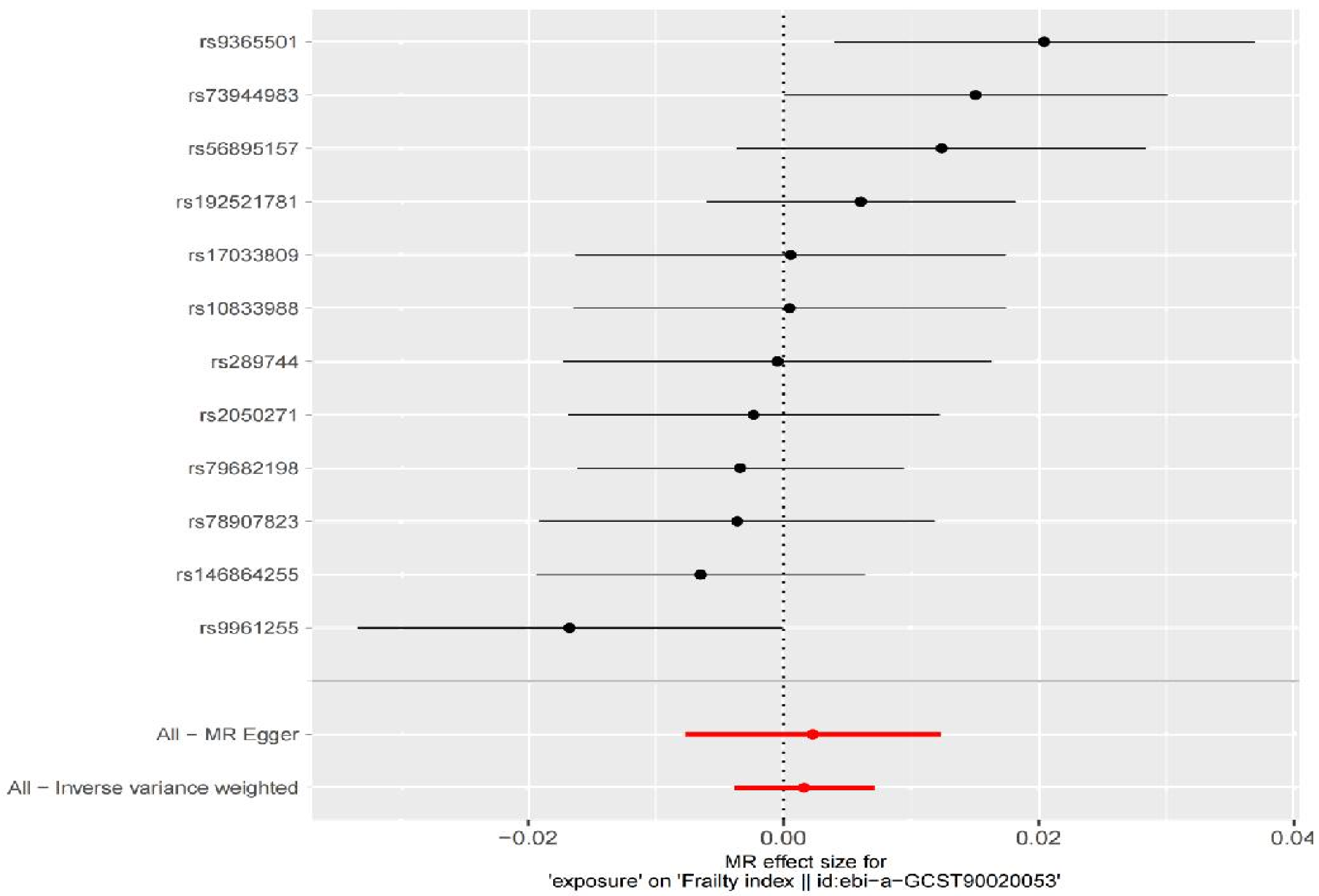

Supplementary Figure-42D Funnel Plot

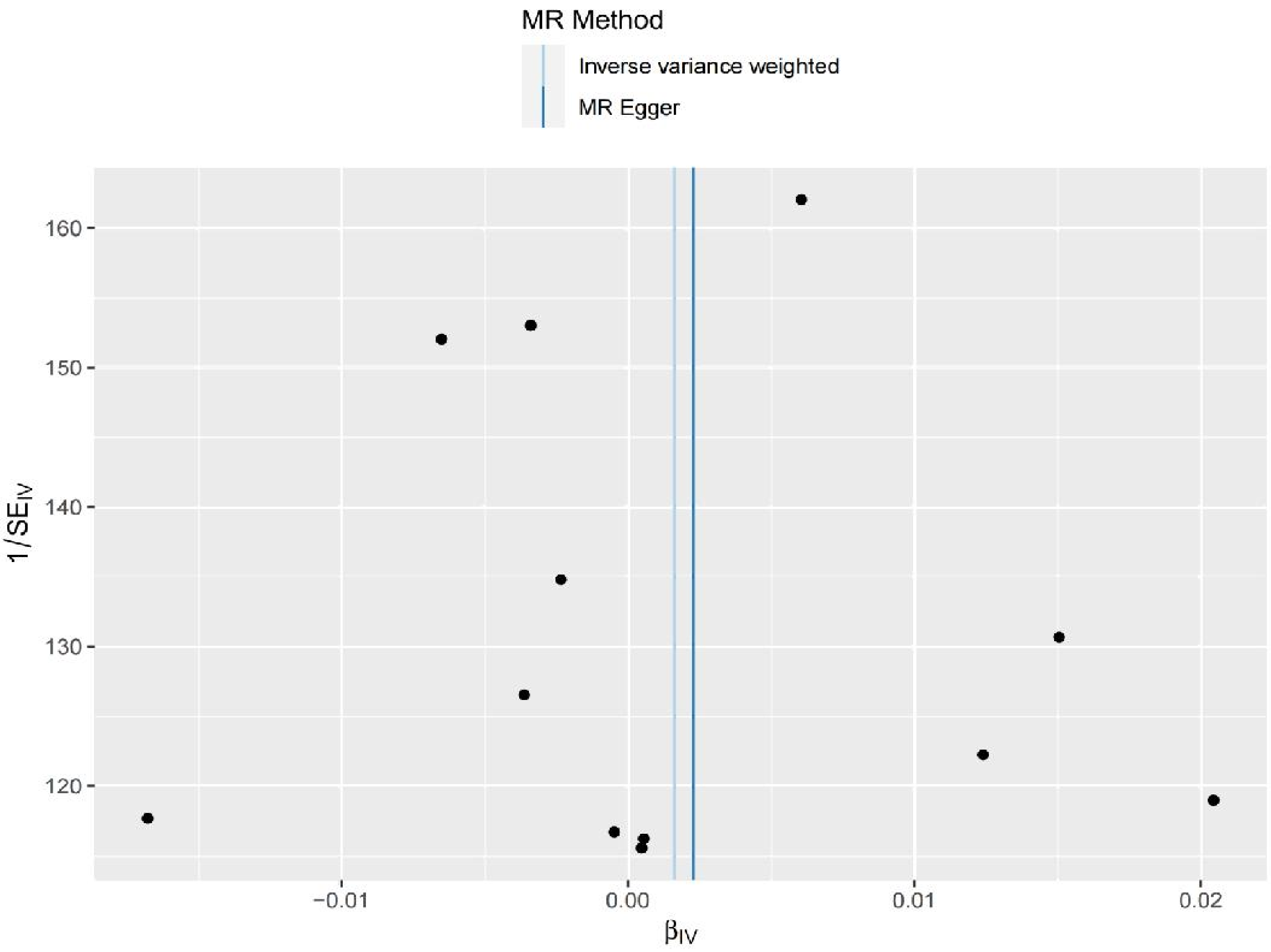

Supplementary Figure-43 Leave-one-out Analysis, Scatter Plot, Forest Plot, and Funnel Plot of Meningioma on DNA methylation GrimAge Acceleration

Supplementary Figure-43A Leave-one-out Analysis

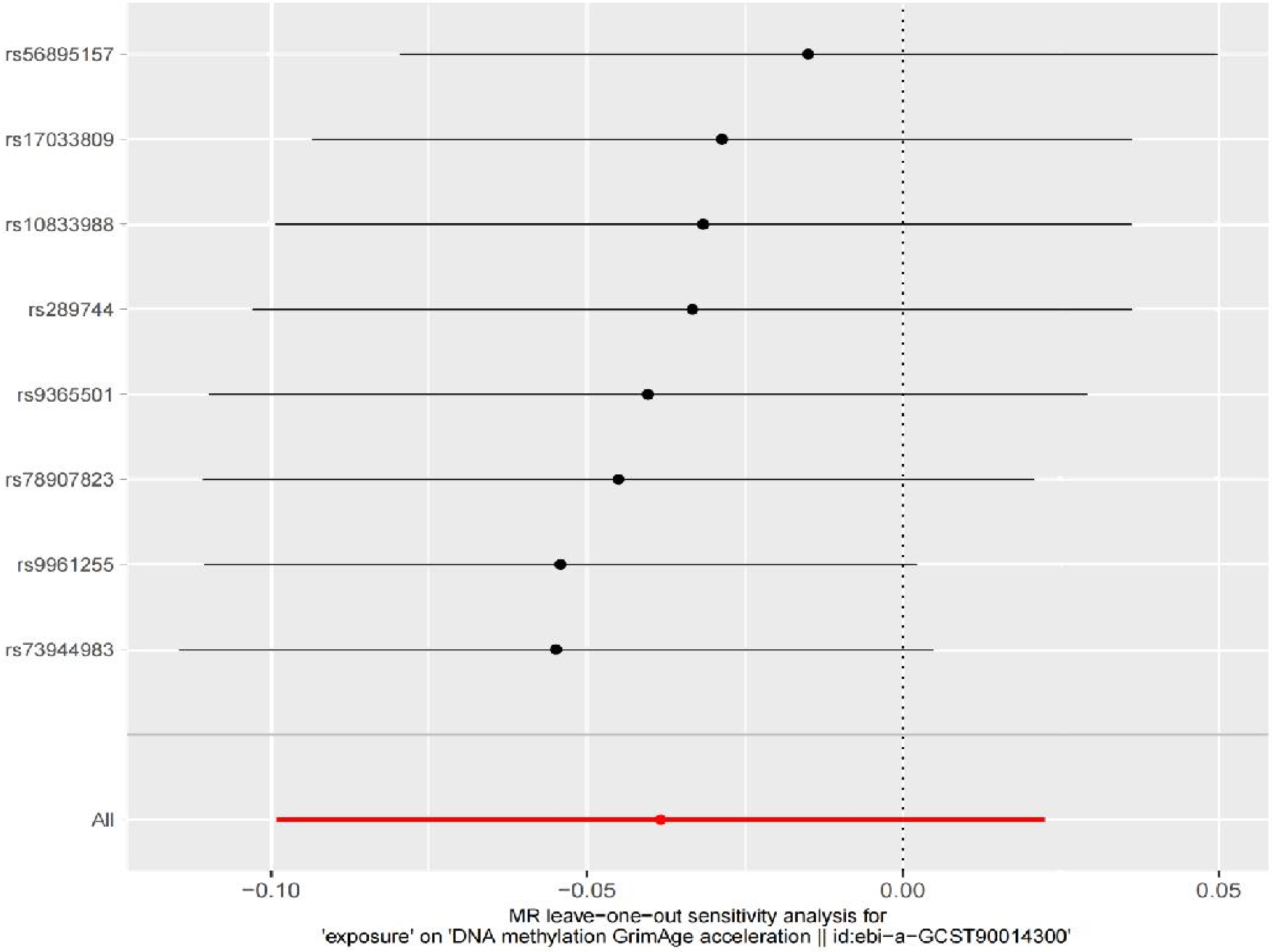

Supplementary Figure-43B Scatter

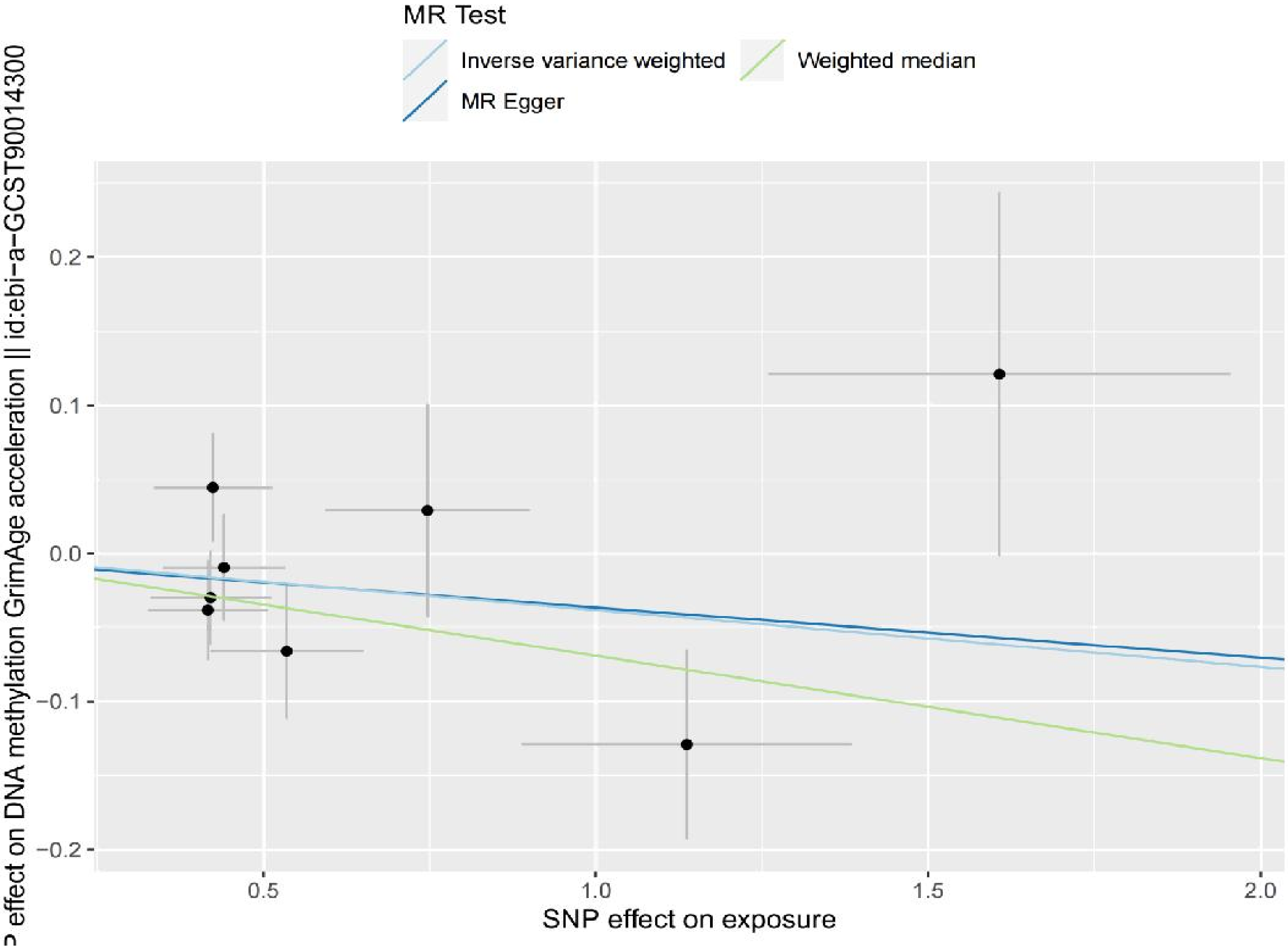

Supplementary Figure-43C Forest Plot

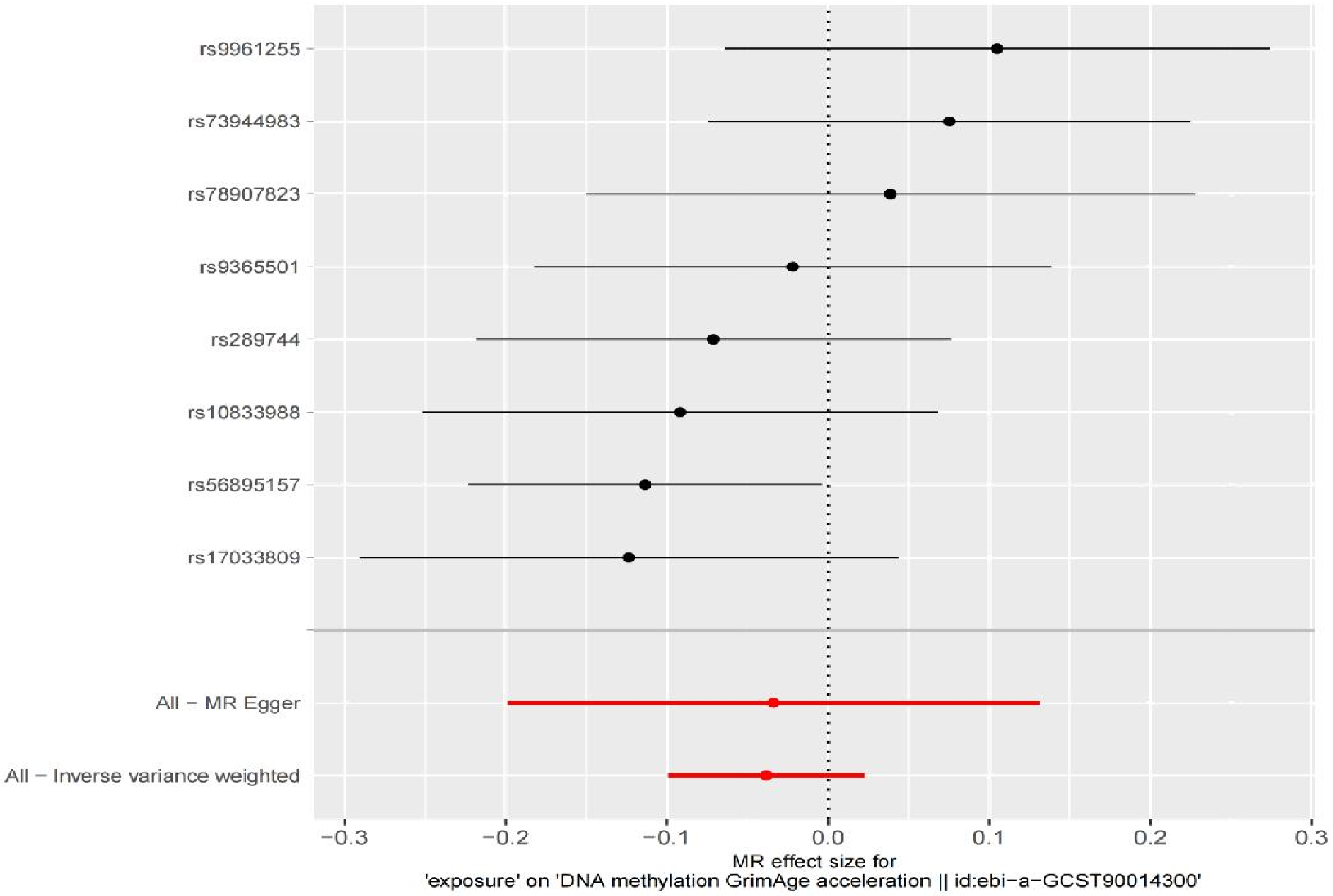

Supplementary Figure-43D Funnel Plot

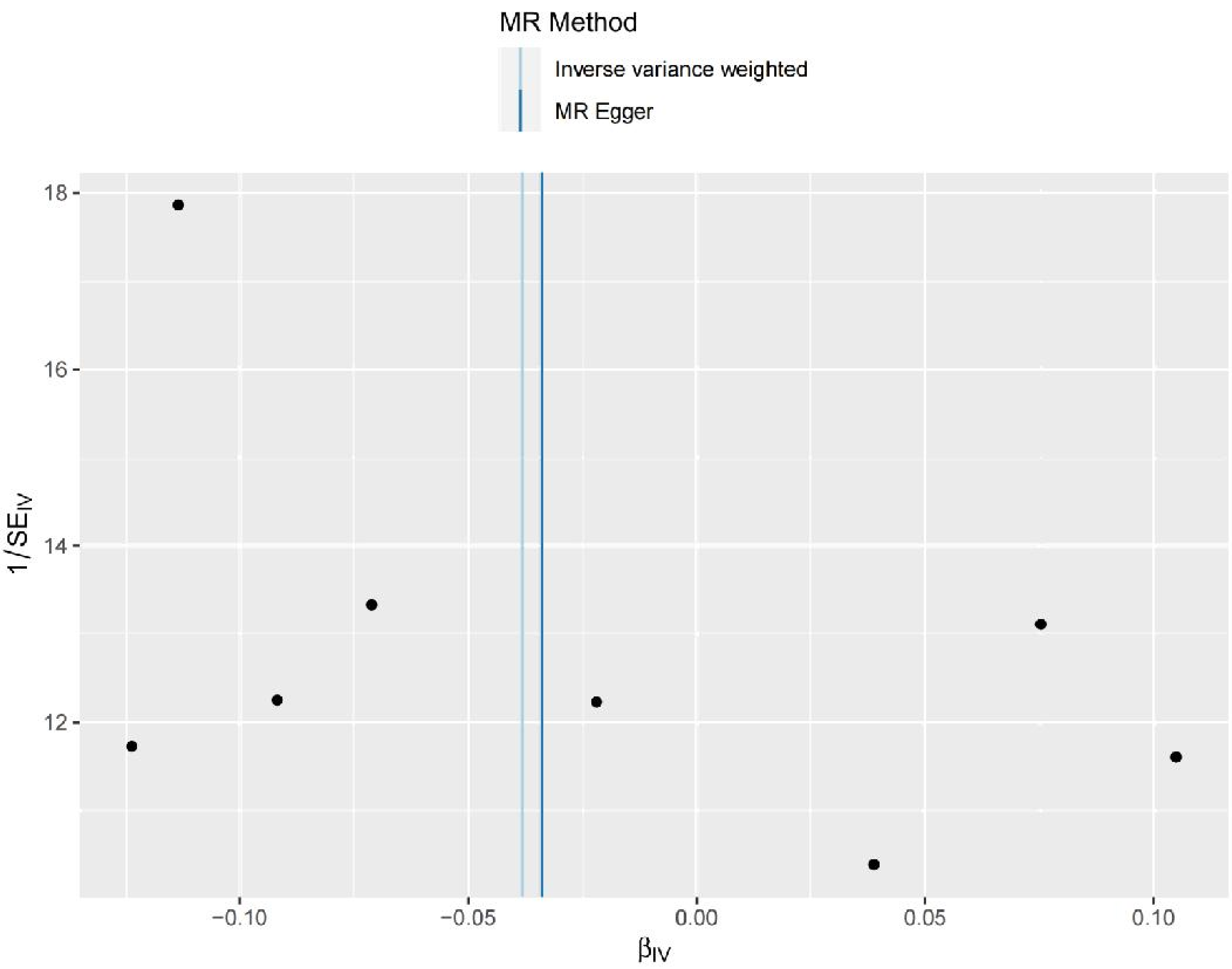

Supplementary Figure-44 Leave-one-out Analysis, Scatter Plot, Forest Plot, and Funnel Plot of Meningioma on Telomere Length

Supplementary Figure-44A Leave-one-out Analysis

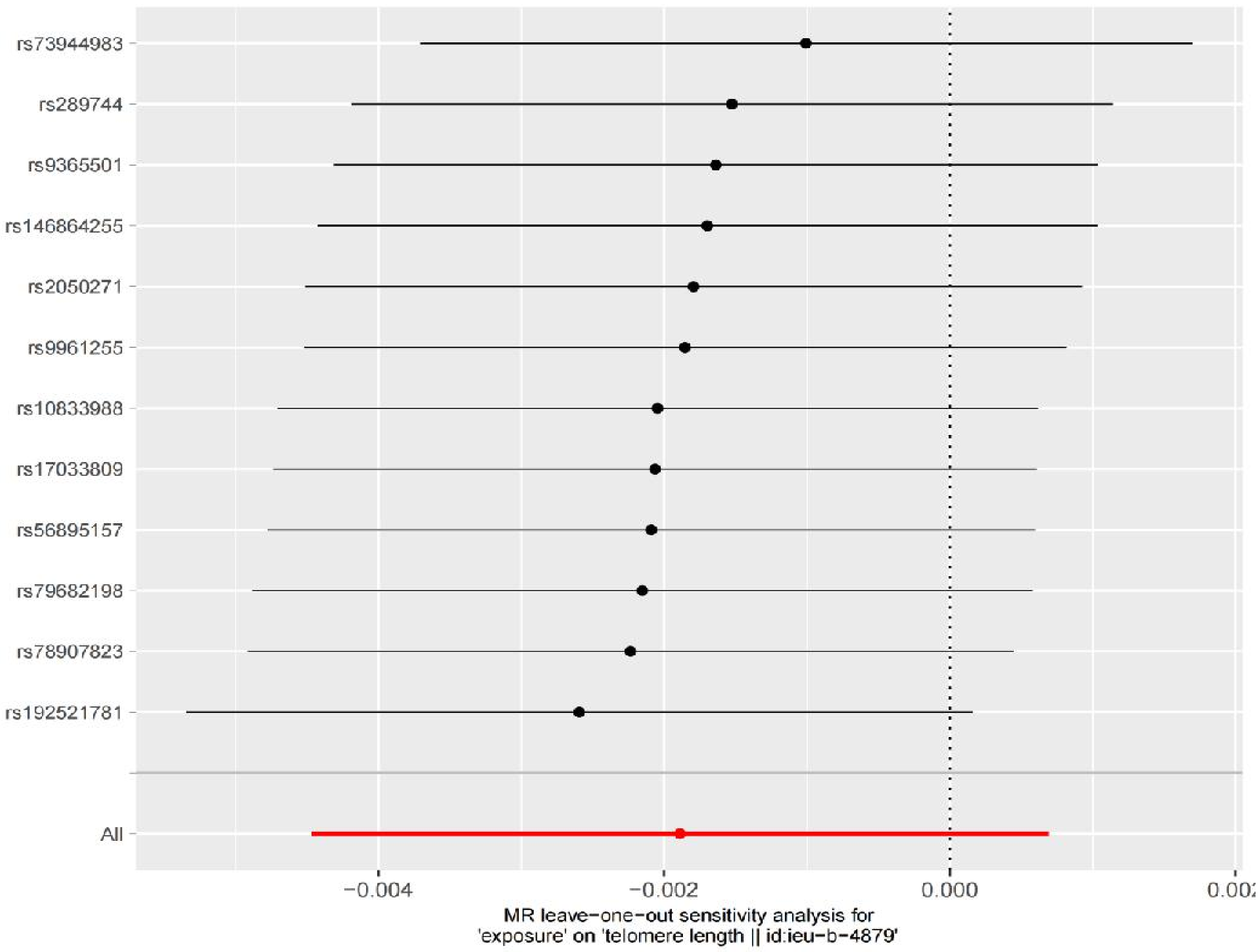

Supplementary Figure-44B Scatter

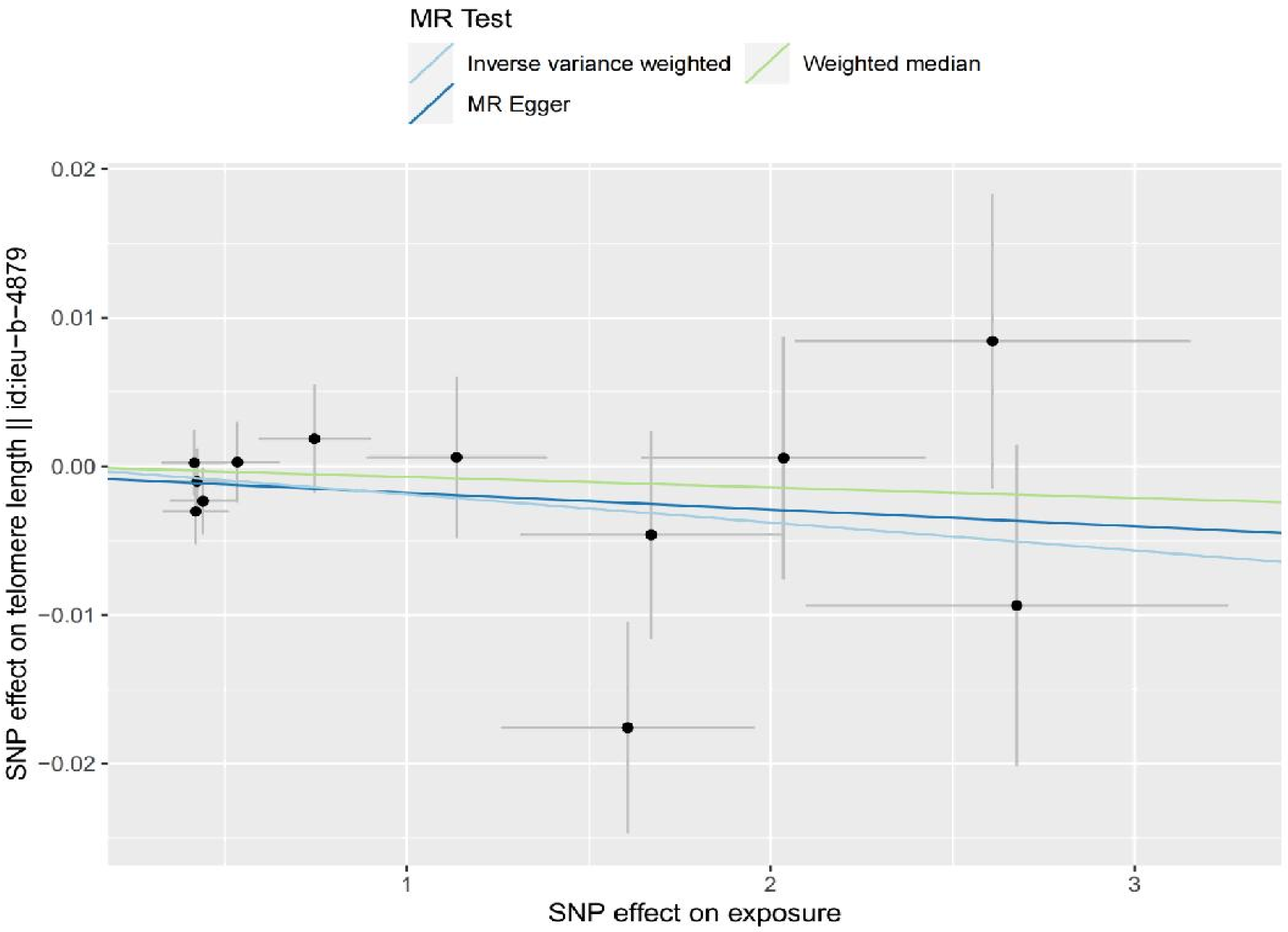

Supplementary Figure-44C Forest Plot

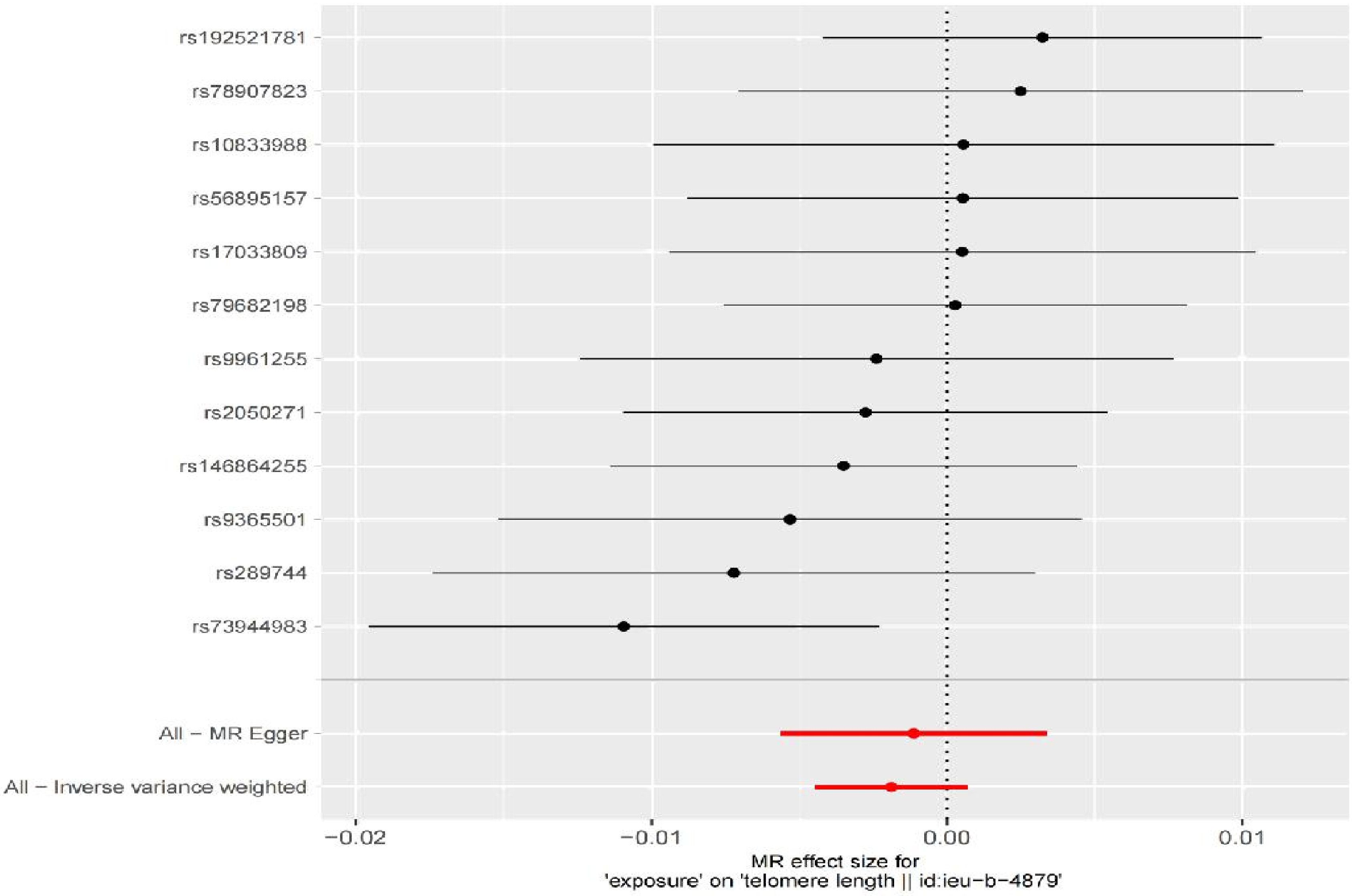

e

Supplementary Figure-44D Funnel Plot

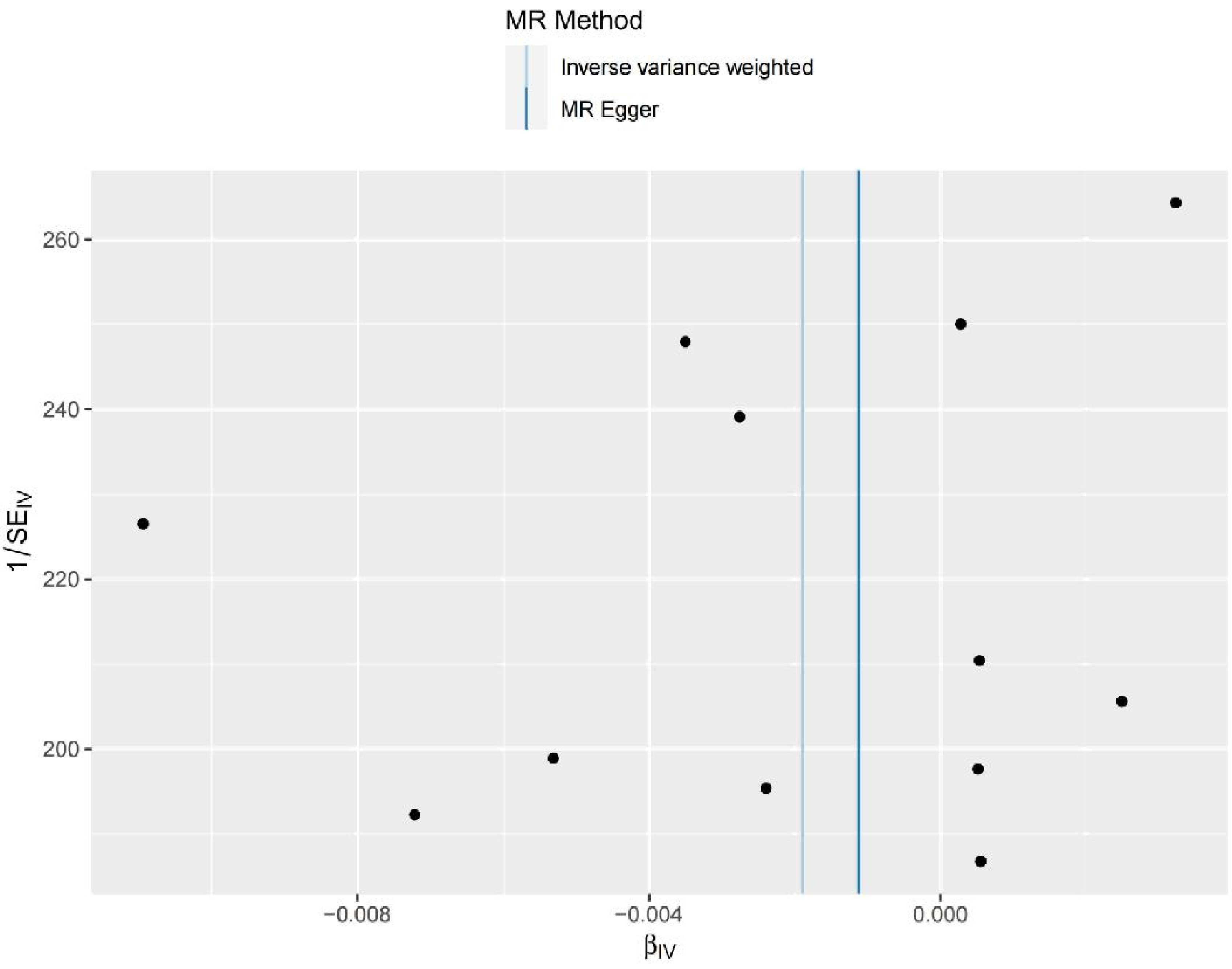

Supplementary Figure-45 Leave-one-out Analysis, Scatter Plot, Forest Plot, and Funnel Plot of Non-GBM on Facial Ageing

Supplementary Figure-45A Leave-one-out Analysis

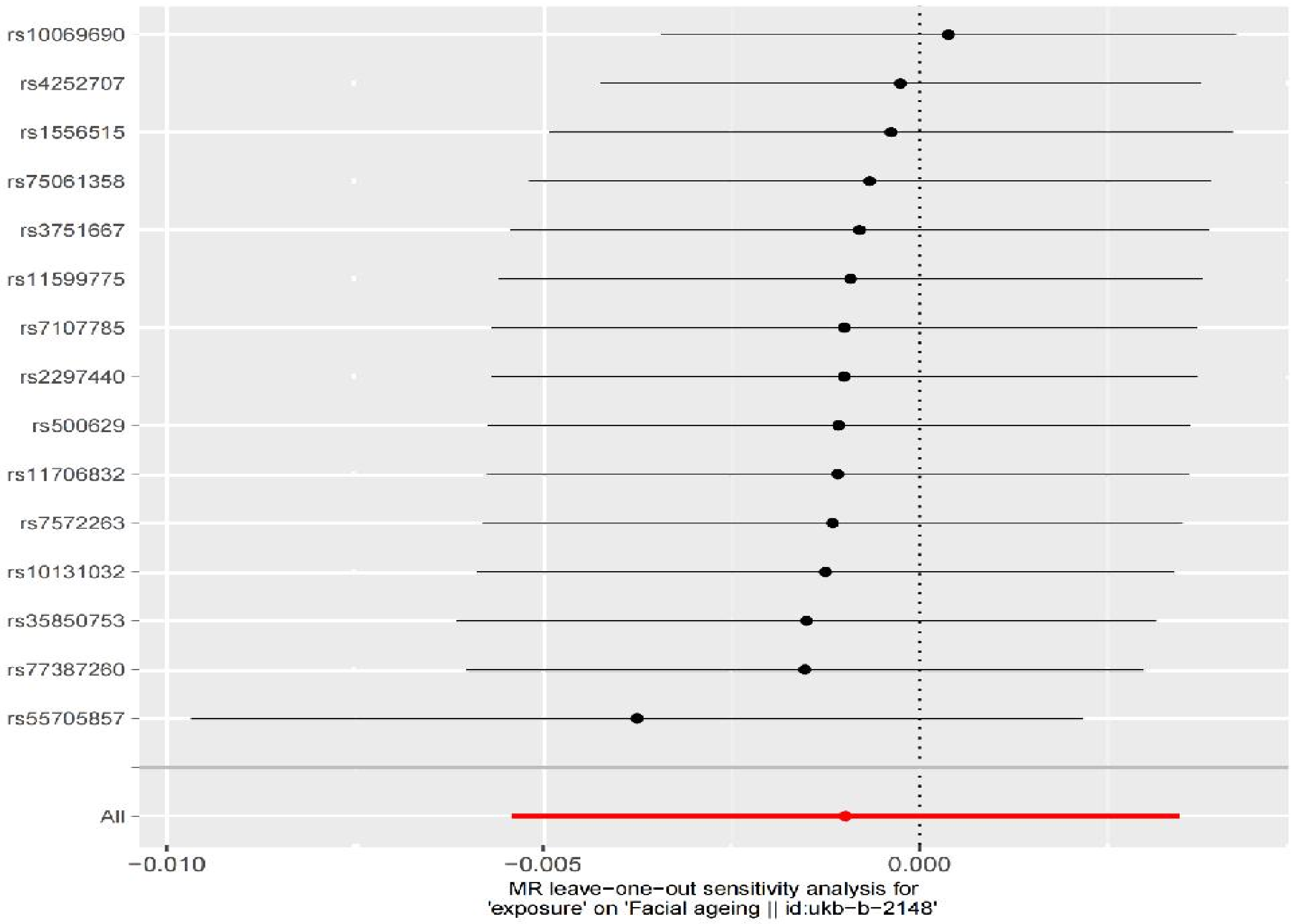

Supplementary Figure-45B Scatter

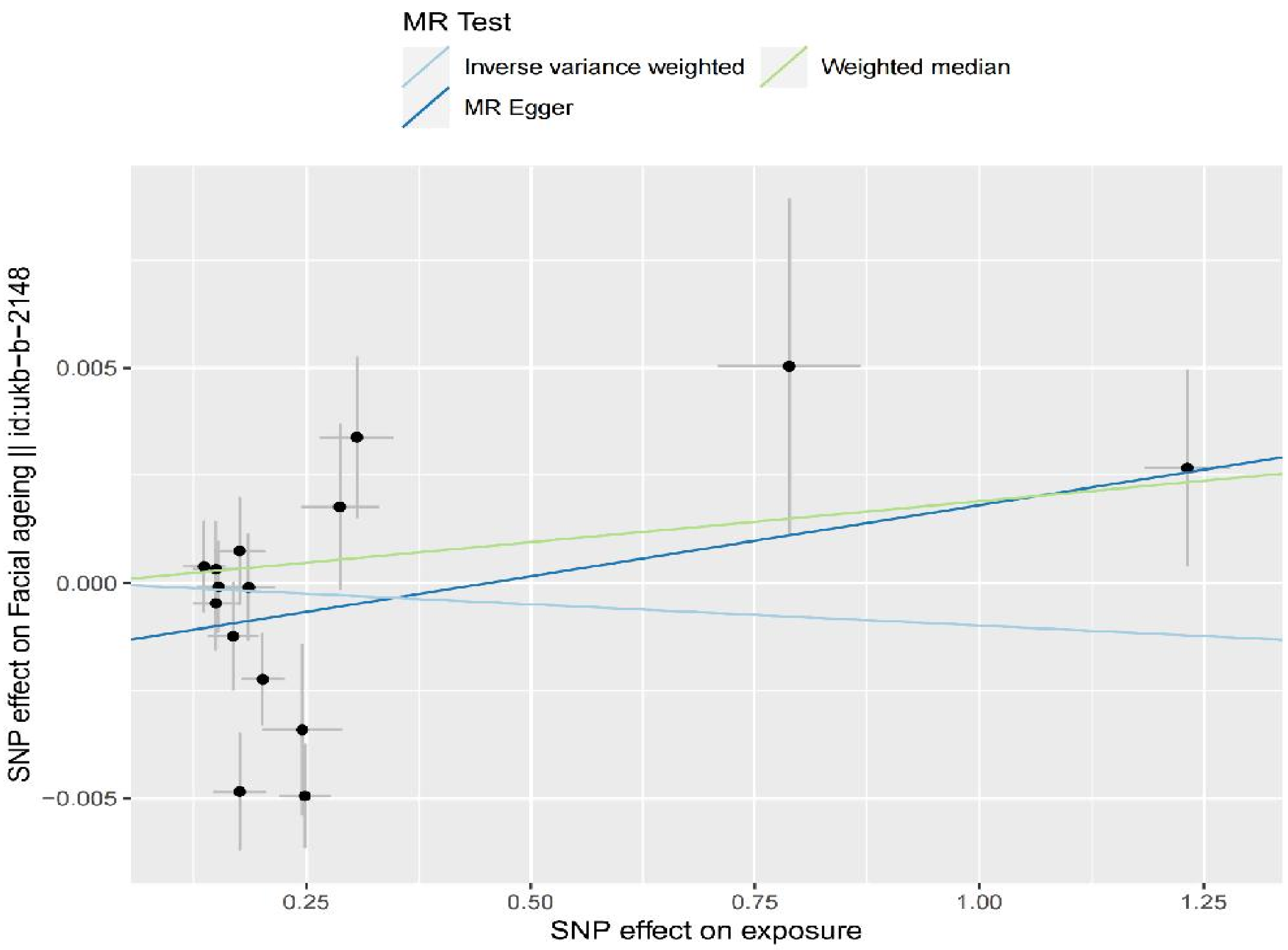

Supplementary Figure-45C Forest Plot

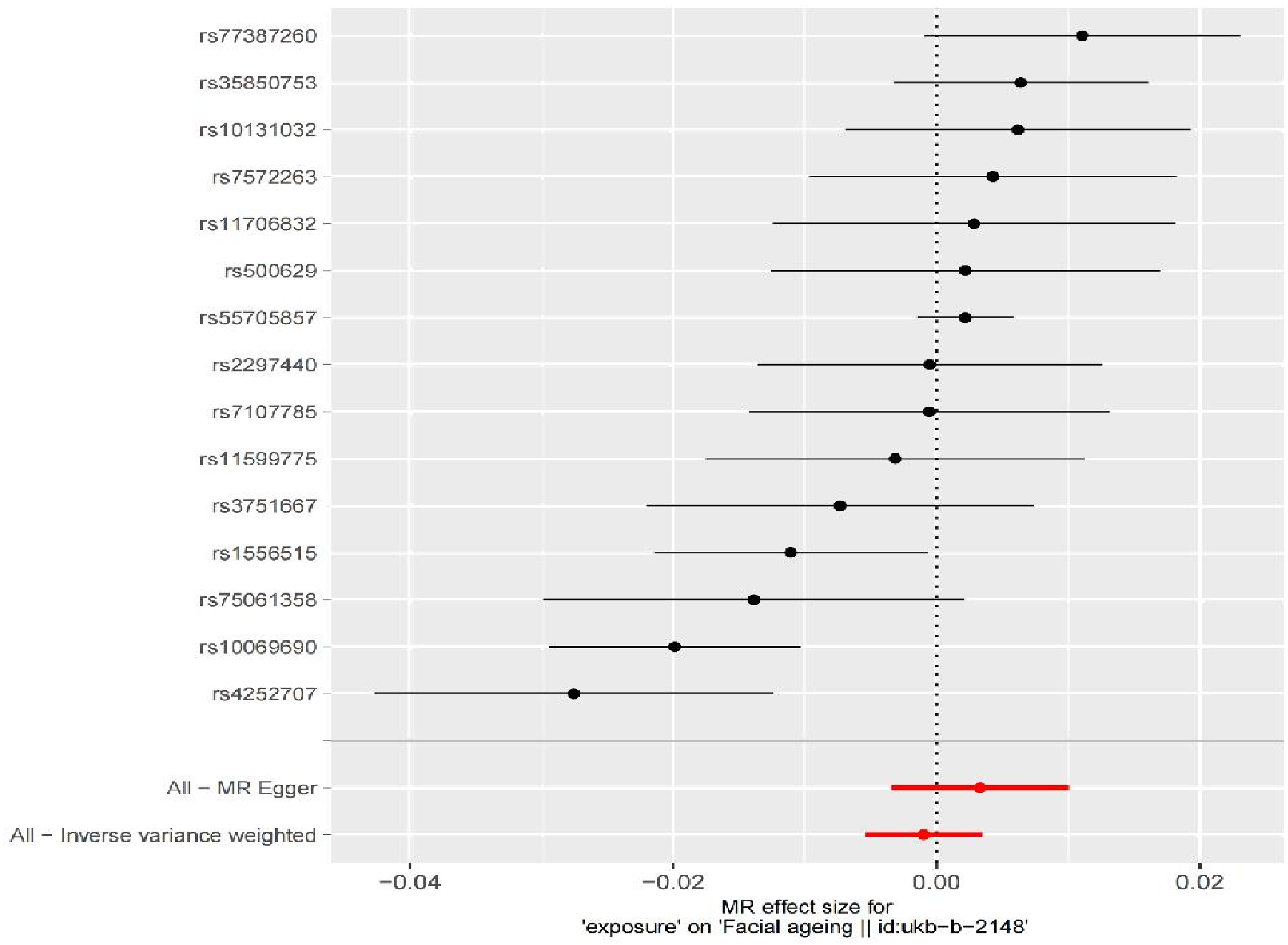

Supplementary Figure-45D Funnel Plot

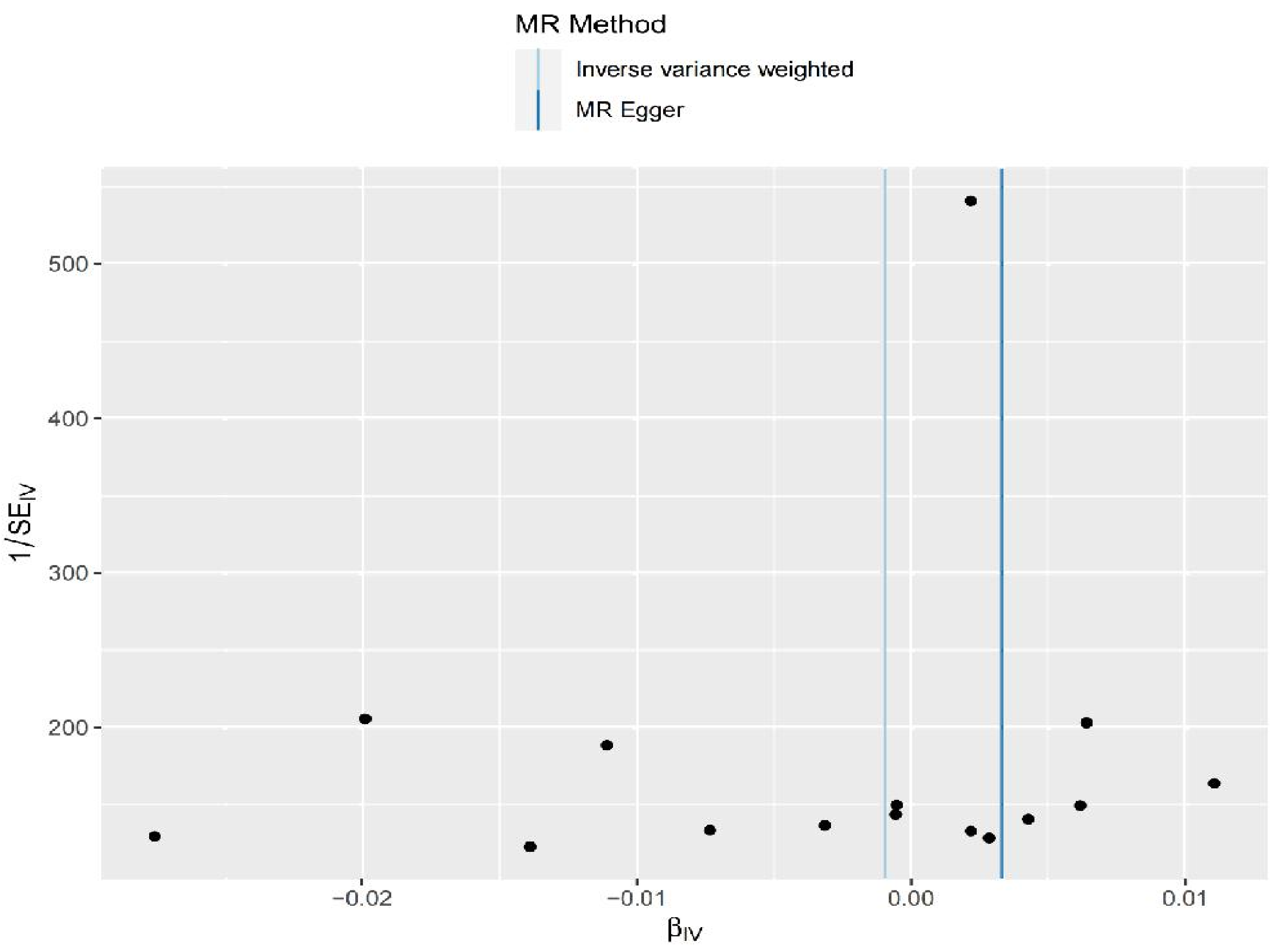

Supplementary Figure-46Leave-one-out Analysis, Scatter Plot, Forest Plot, and Funnel Plot of Non-GBM on Frailty Index

Supplementary Figure-46A Leave-one-out Analysis

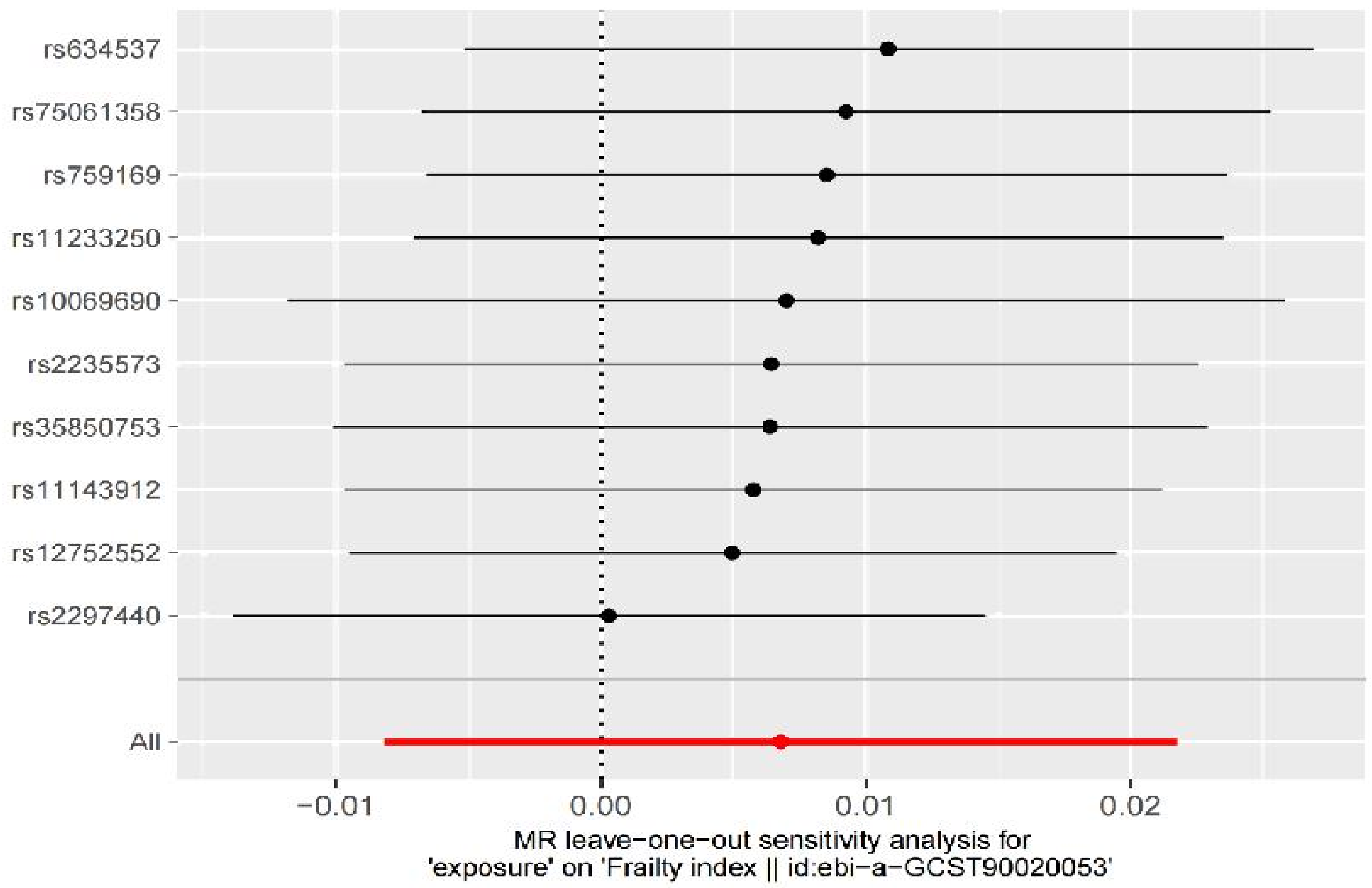

Supplementary Figure-46B Scatter

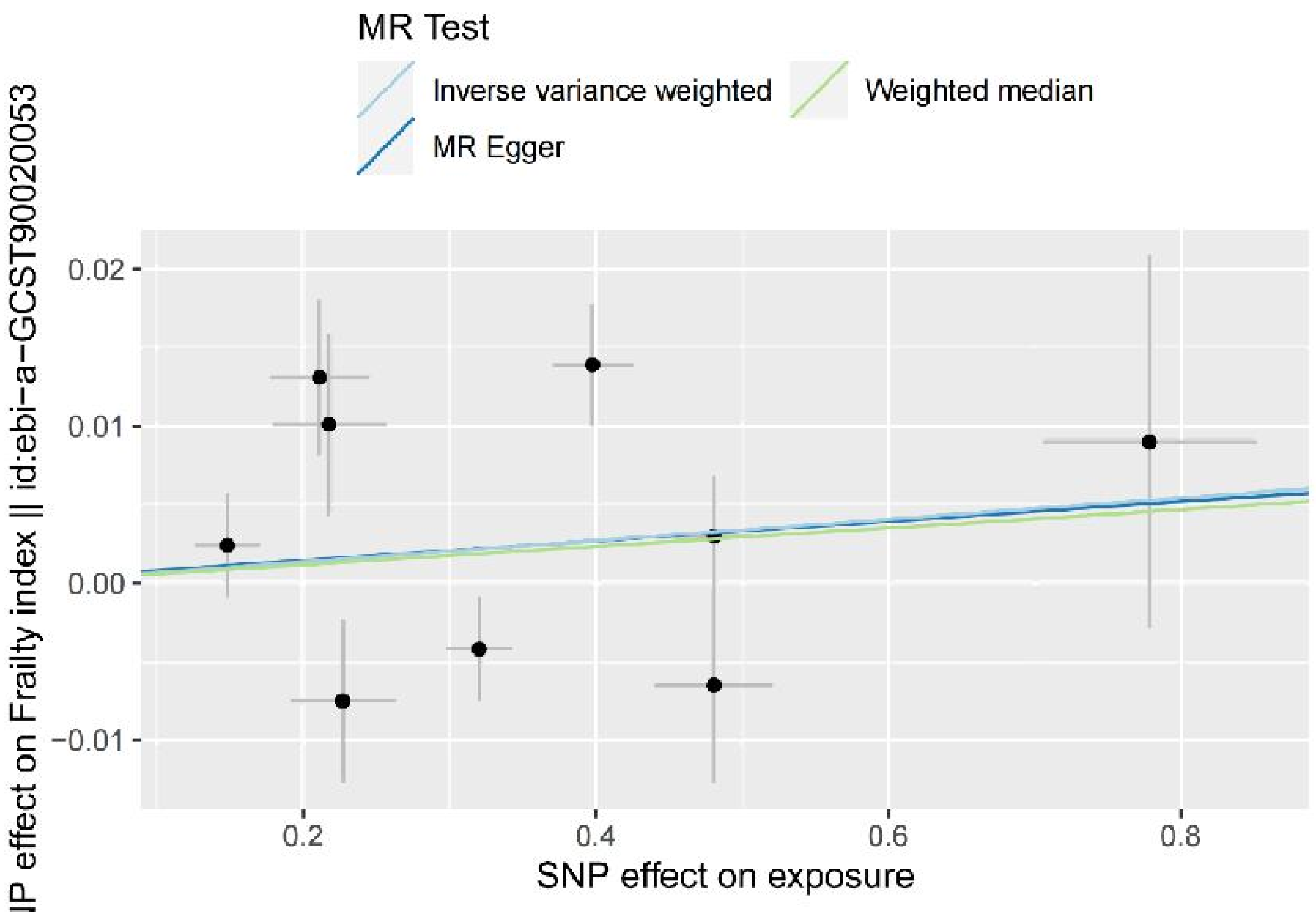

Supplementary Figure-46C Forest Plot

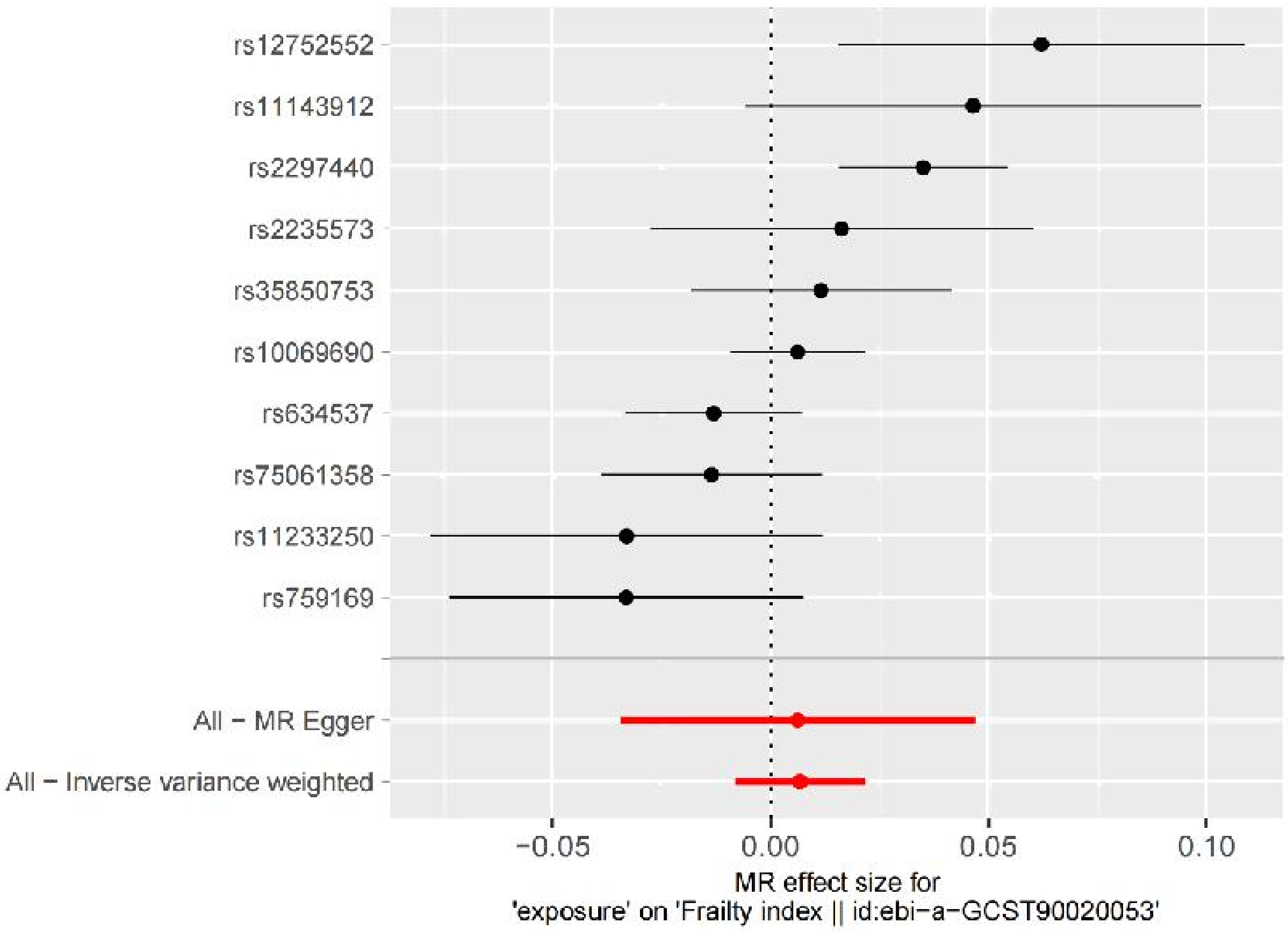

Supplementary Figure-46D Funnel Plot

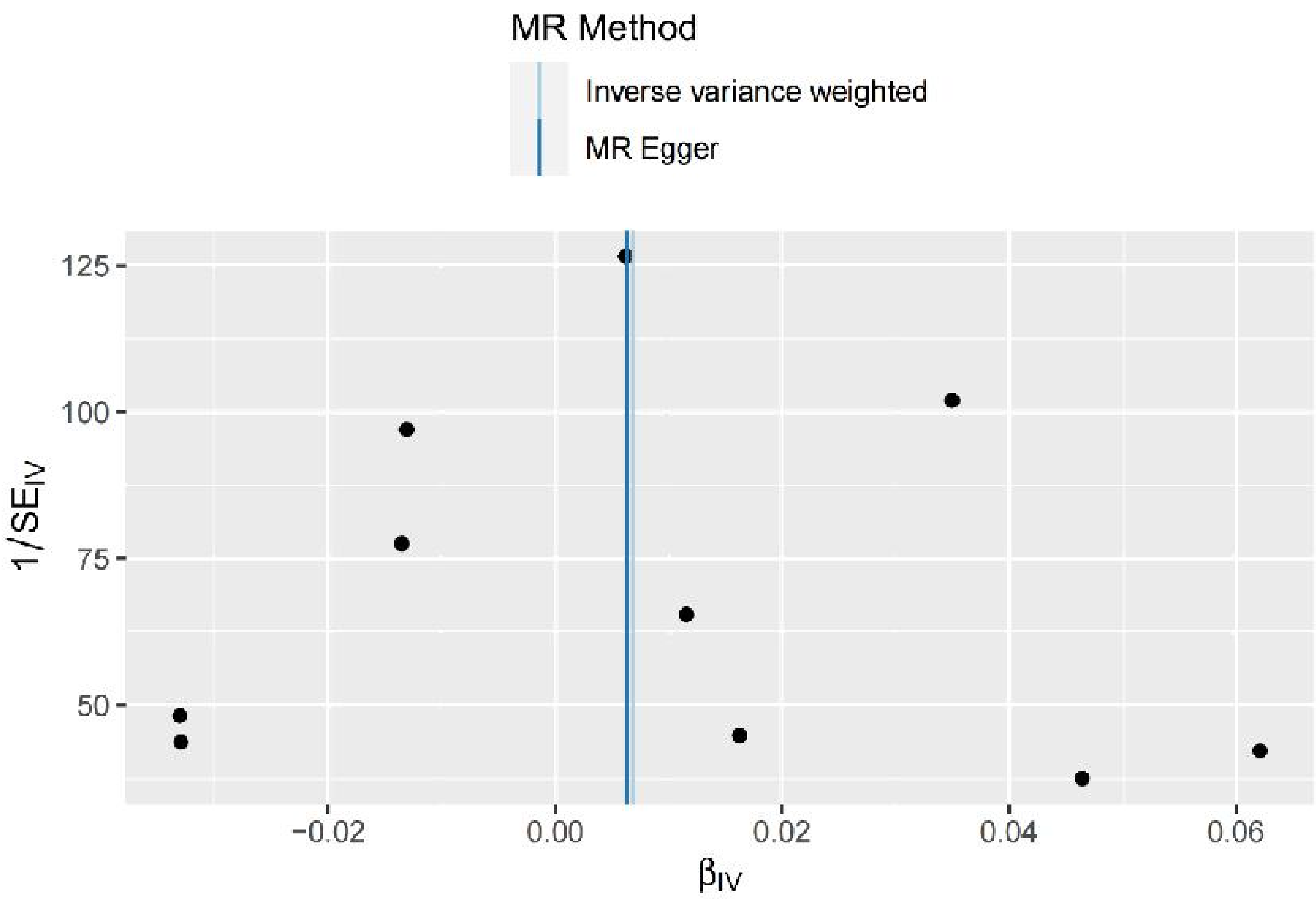

Supplementary Figure-47 Leave-one-out Analysis, Scatter Plot, Forest Plot, and Funnel Plot of Non-GBM on DNA methylation GrimAge Acceleration

Supplementary Figure-47A Leave-one-out Analysis

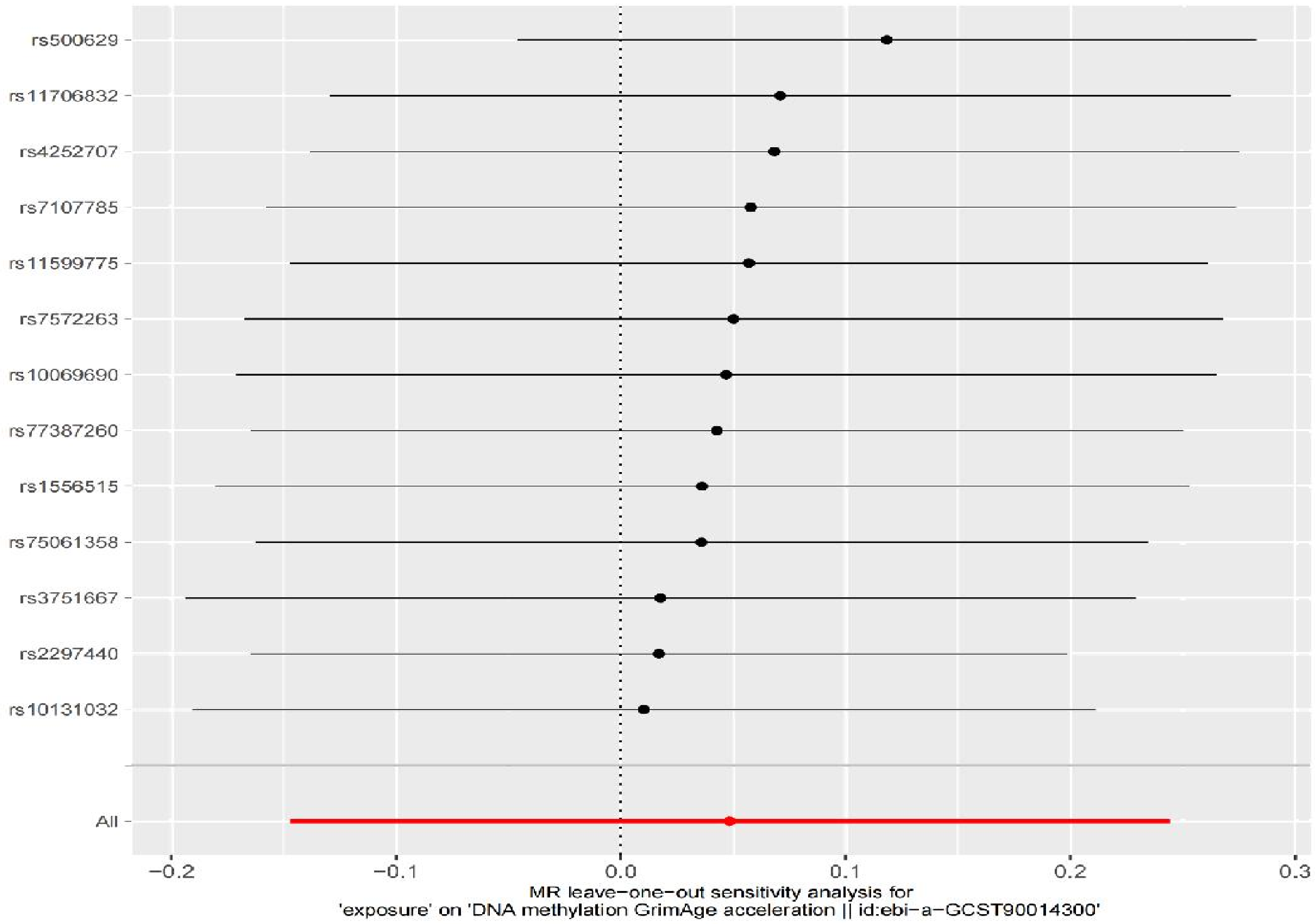

Supplementary Figure-47B Scatter

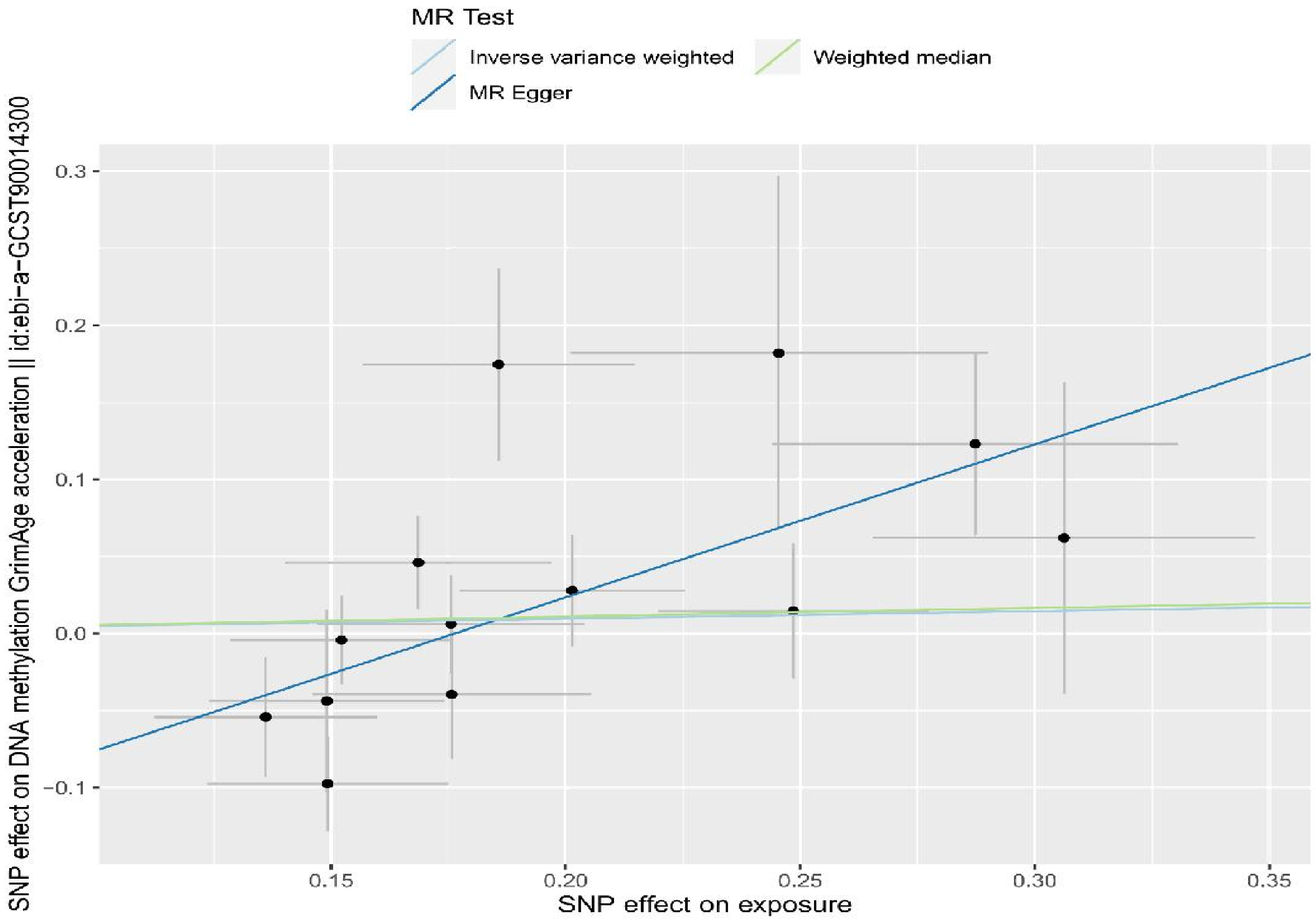

Supplementary Figure-47C Forest Plot

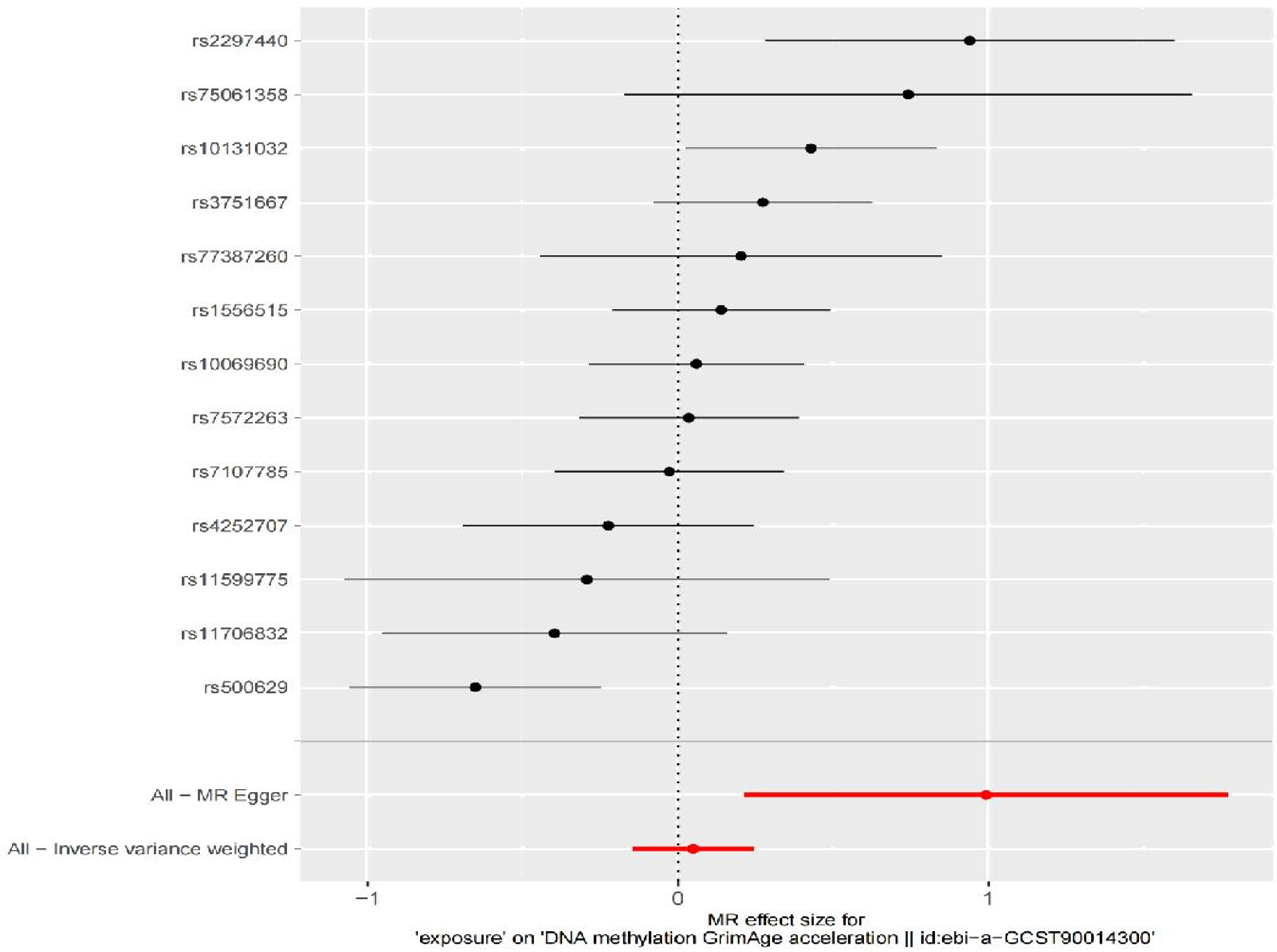

Supplementary Figure-47D Funnel Plot

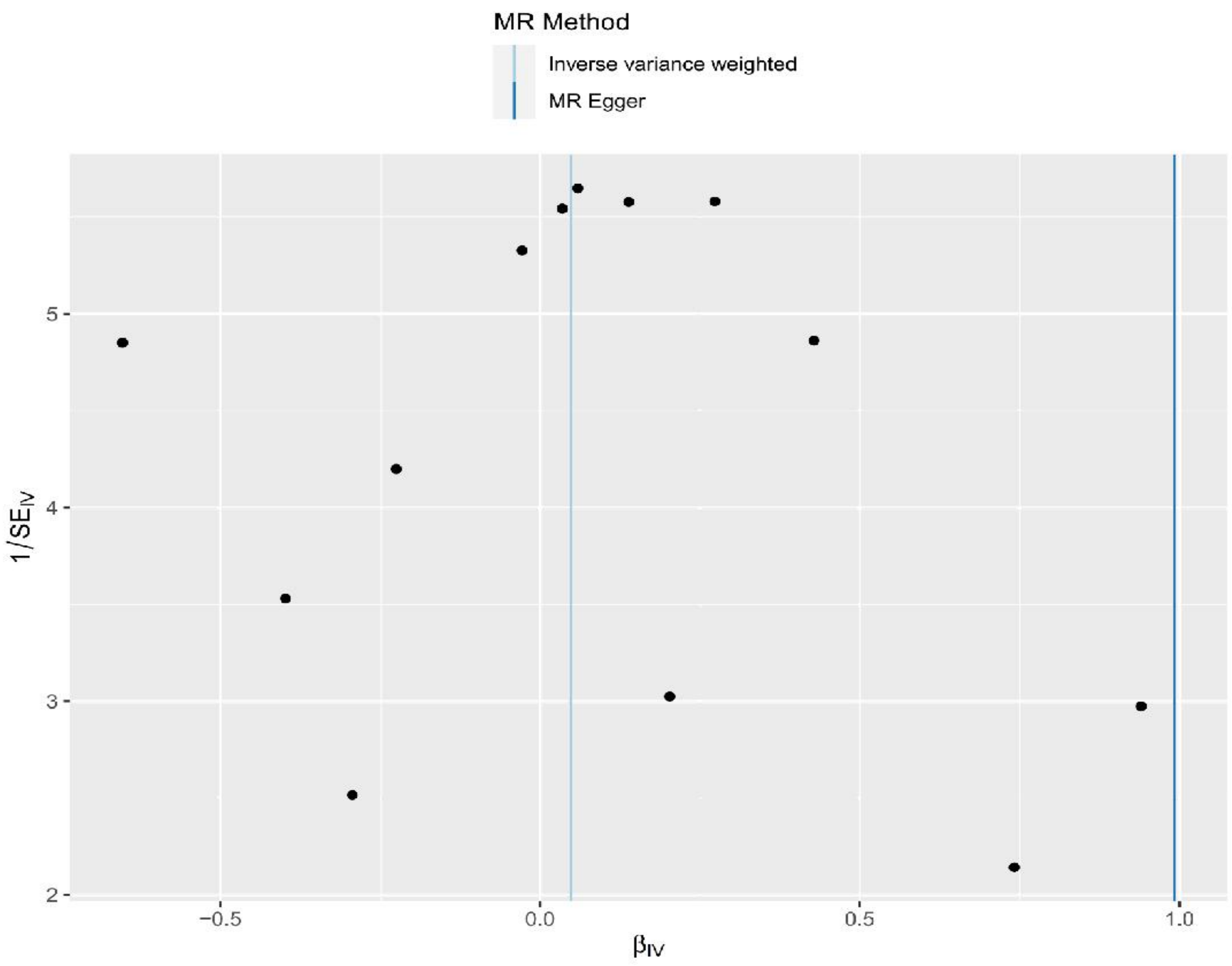

Supplementary Figure-48 Leave-one-out Analysis, Scatter Plot, Forest Plot, and Funnel Plot of Non-GBM on Telomere Length

Supplementary Figure-48A Leave-one-out Analysis

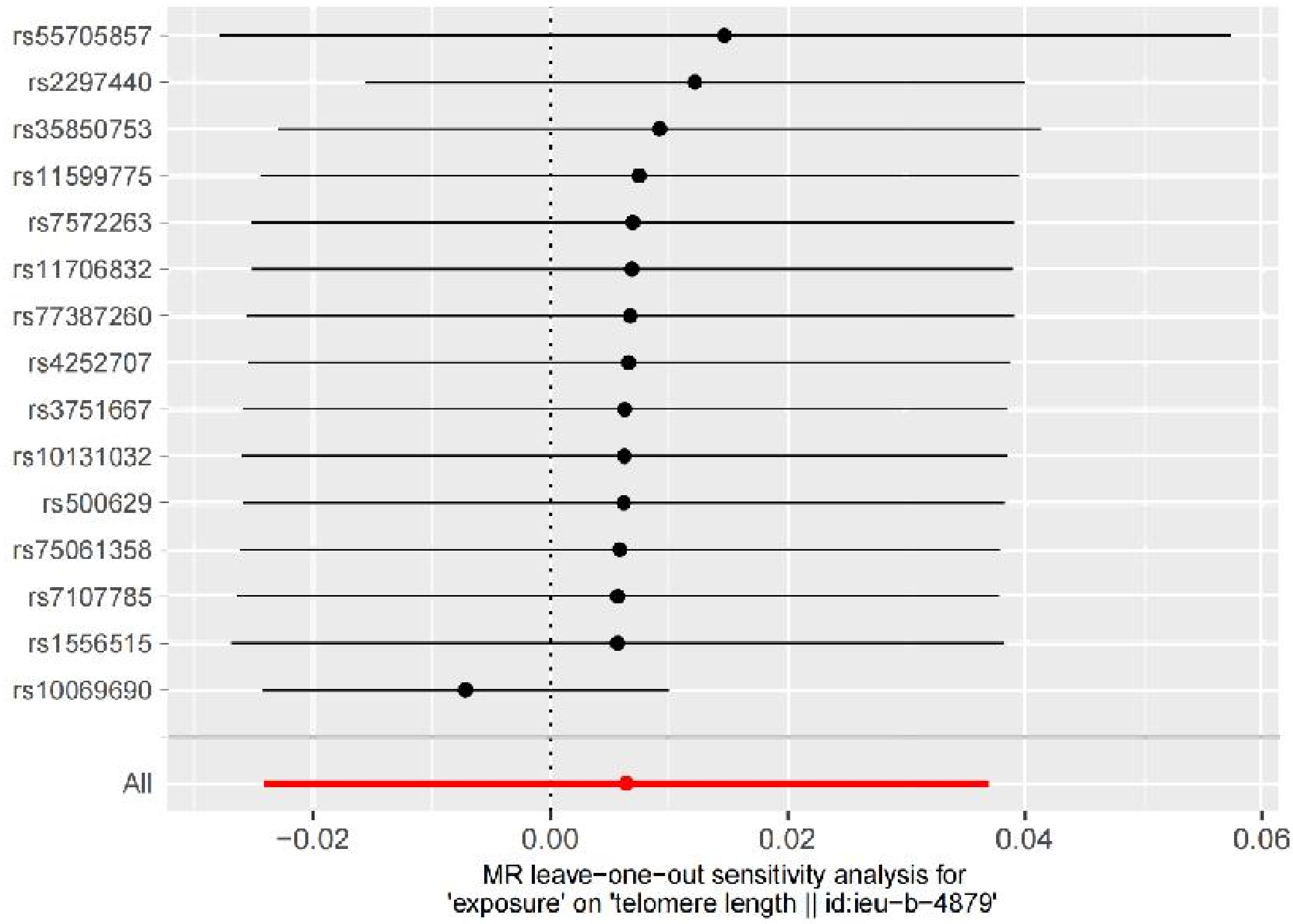

Supplementary Figure-48B Scatter

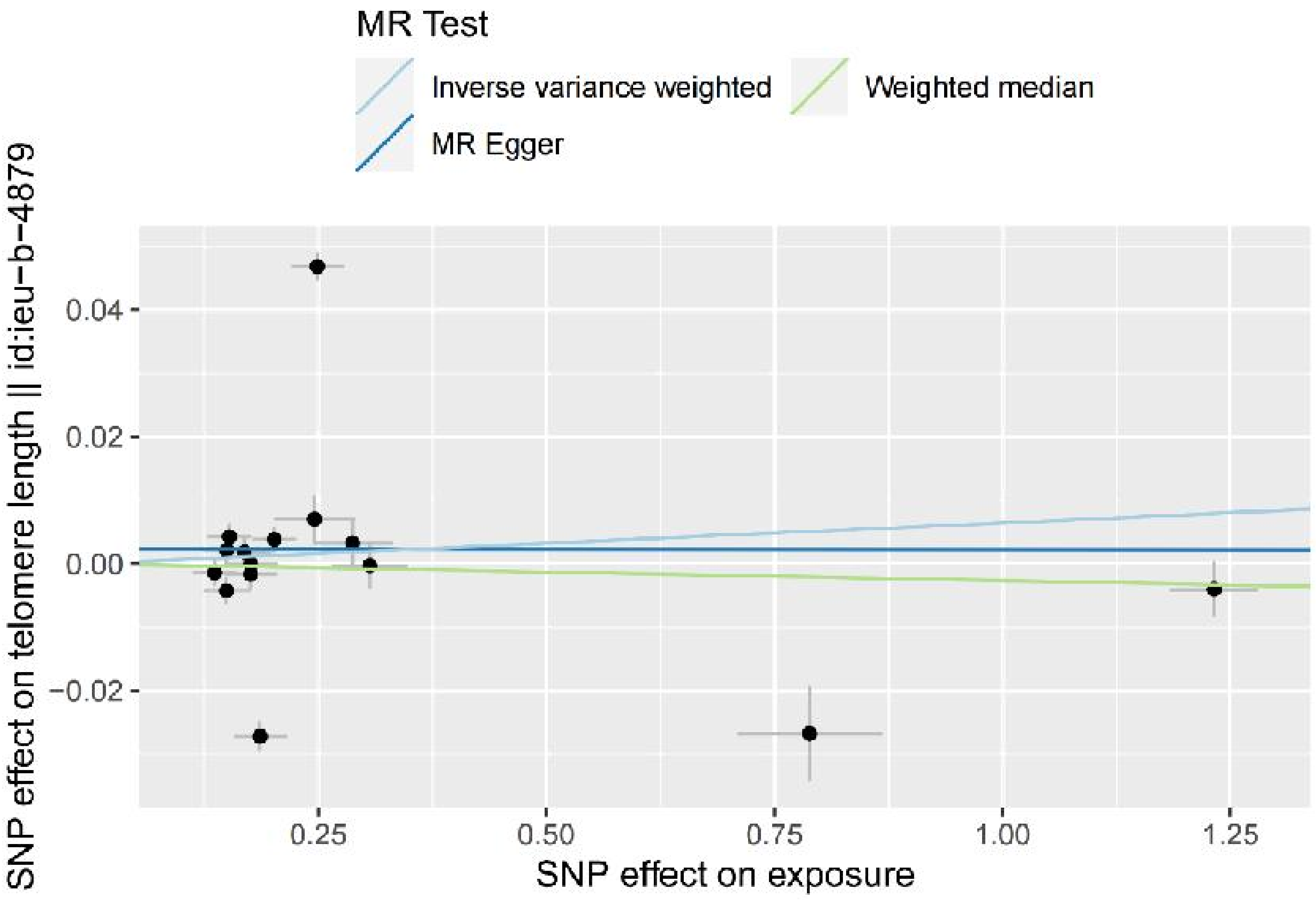

Supplementary Figure-48C Forest Plot

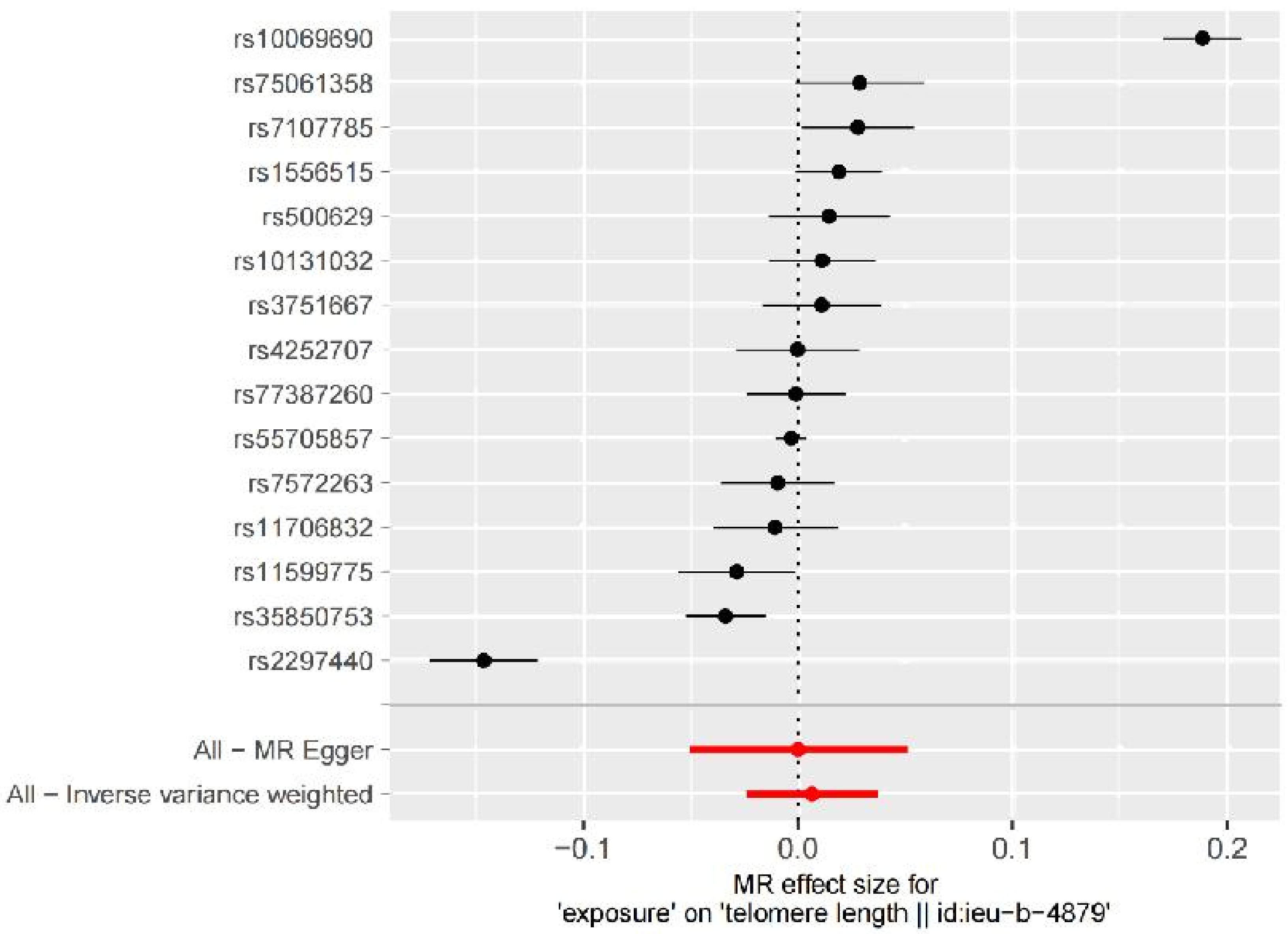

Supplementary Figure-48D Funnel Plot

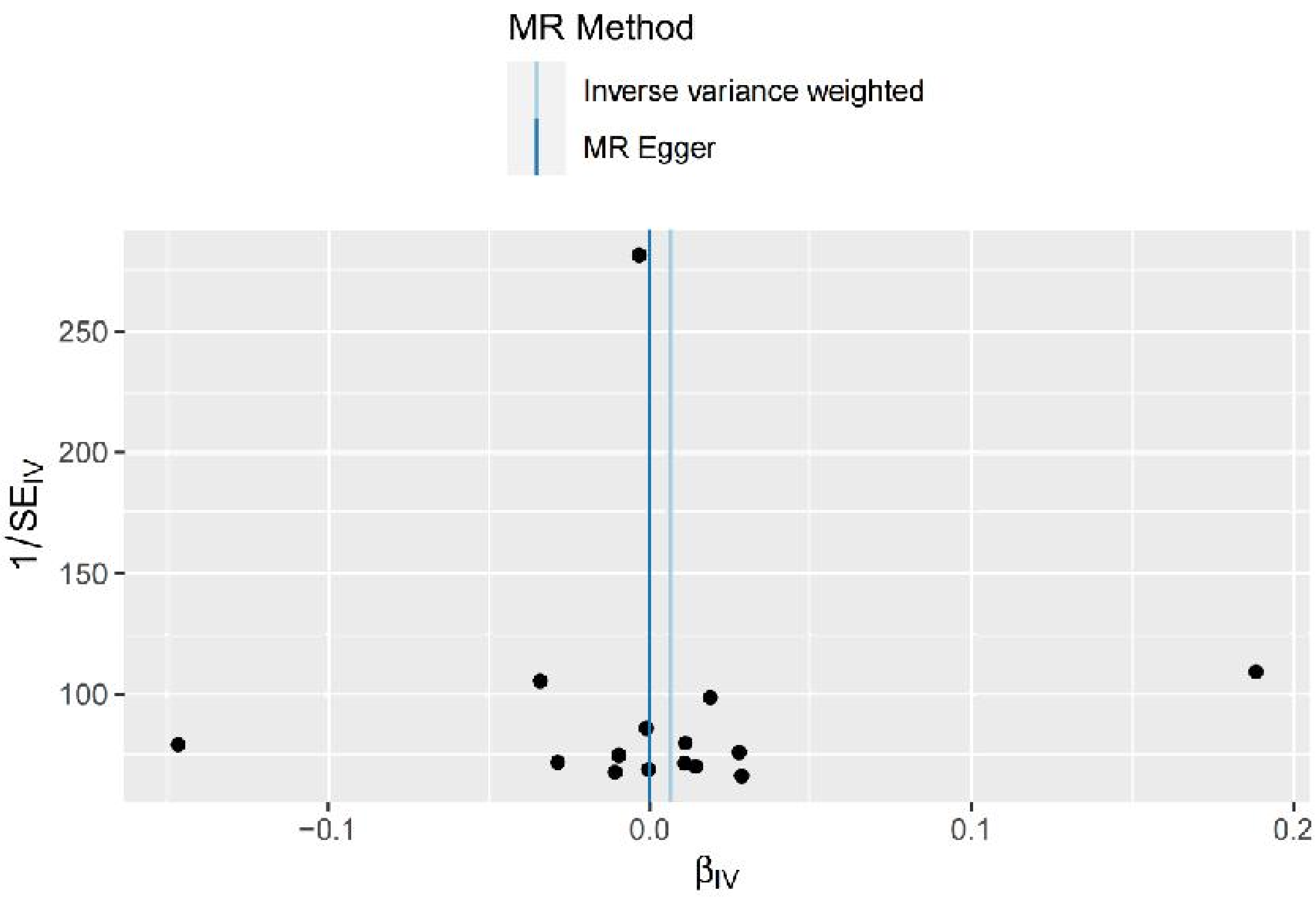

Supplementary Figure-49 Leave-one-out Analysis, Scatter Plot, Forest Plot, and Funnel Plot of Parkinson's Disease on Facial Ageing

Supplementary Figure-49A Leave-one-out Analysis

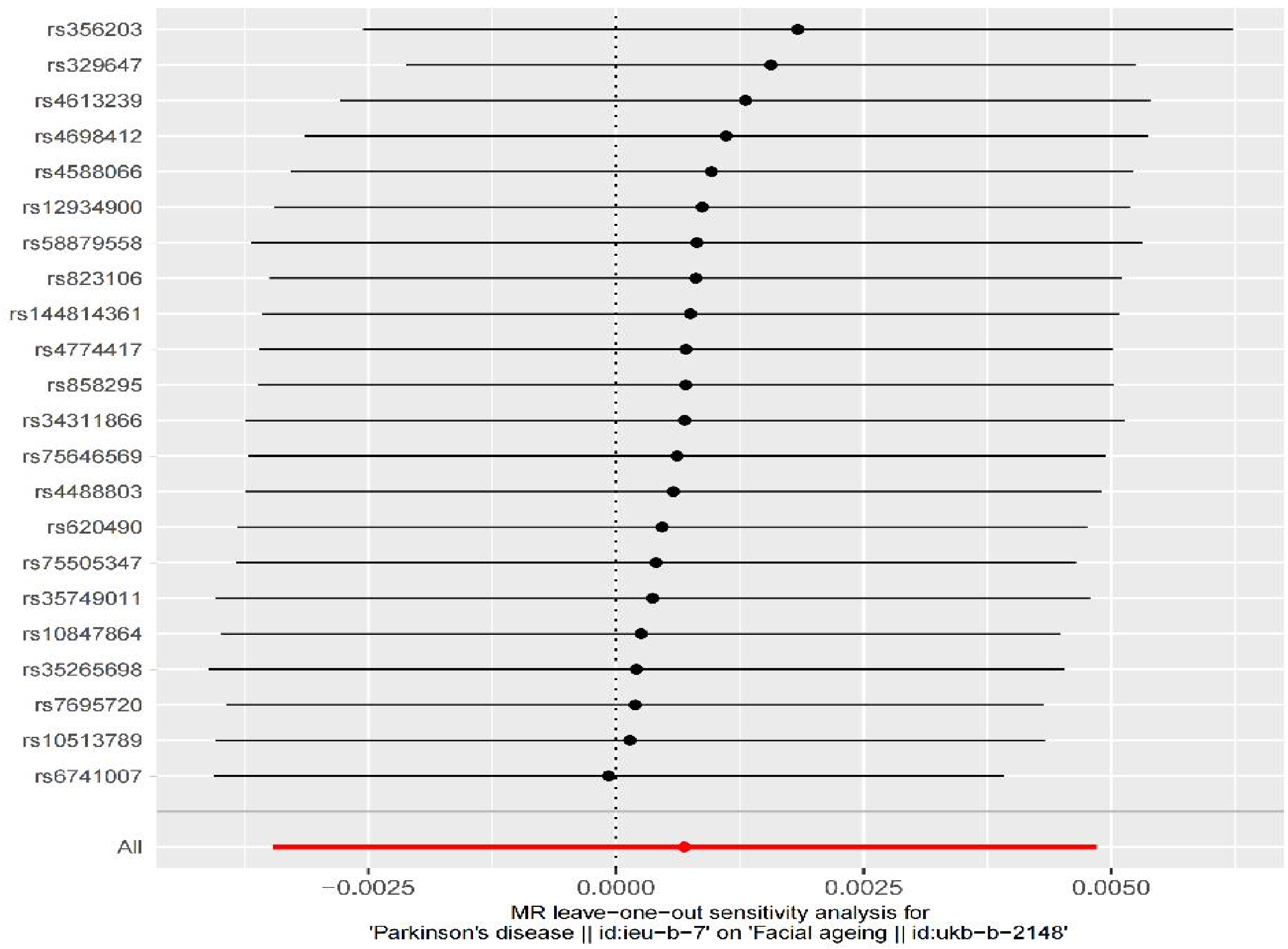

Supplementary Figure-49B Scatter

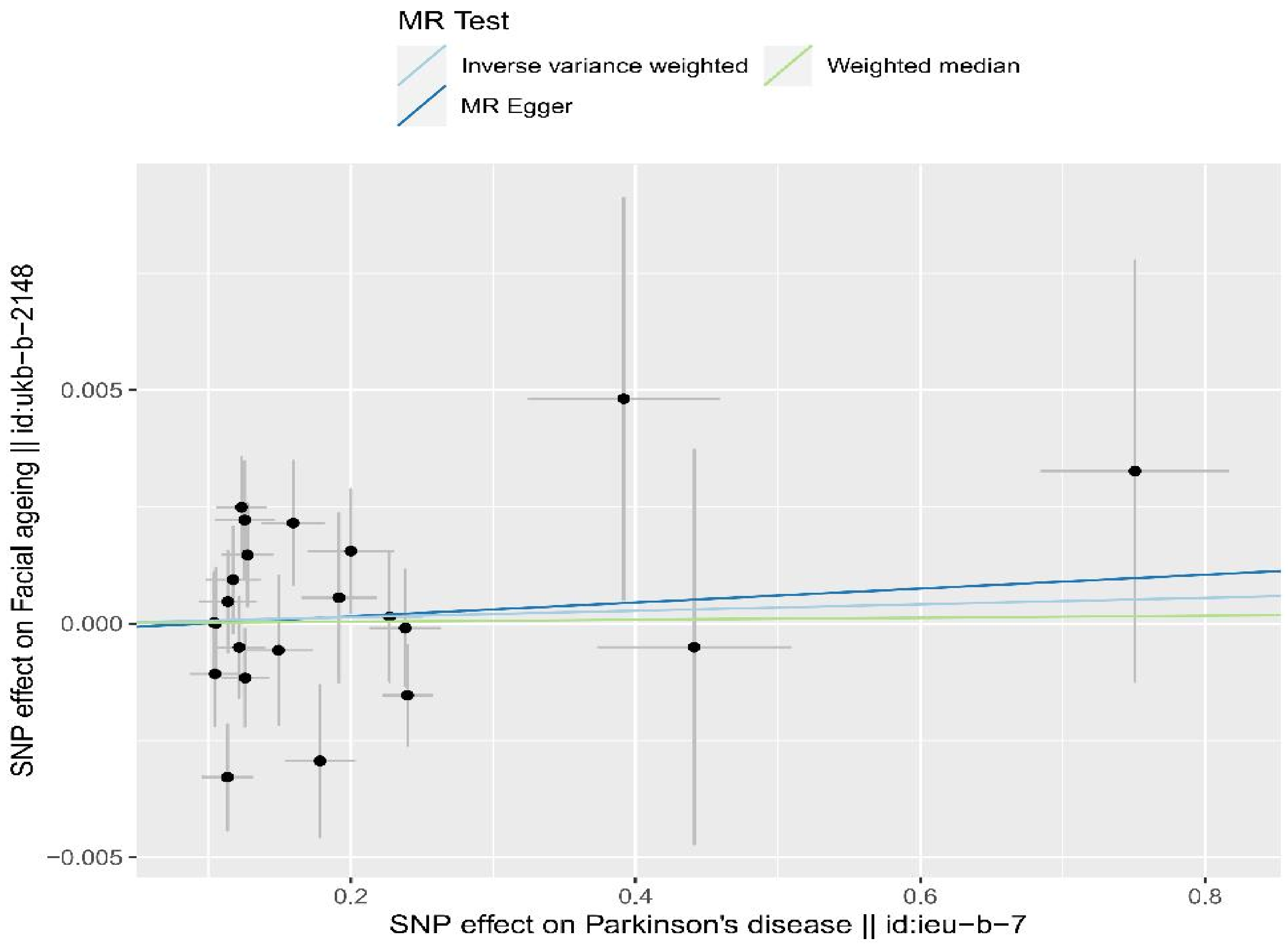

Supplementary Figure-49C Forest Plot

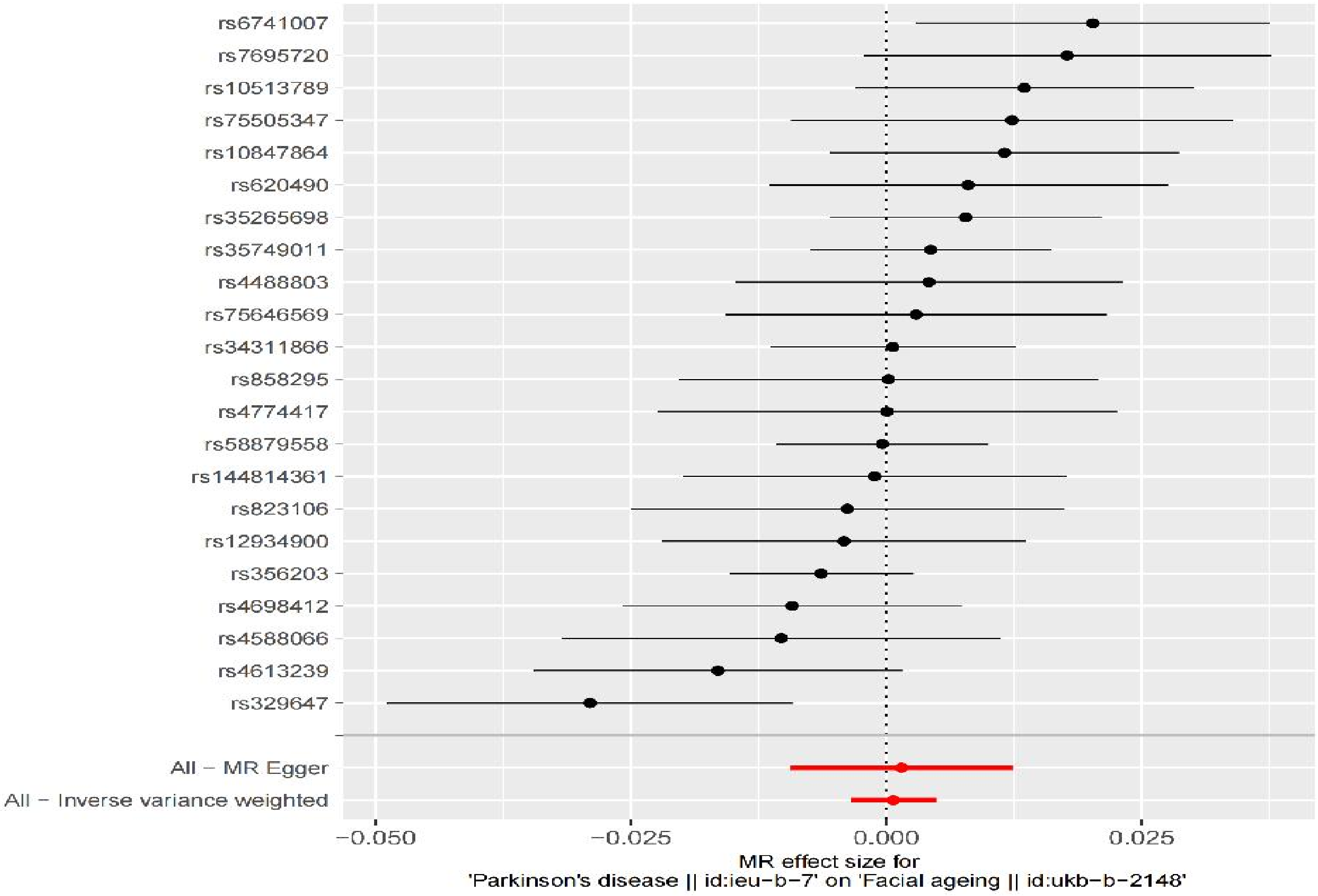

Supplementary Figure-49D Funnel Plot

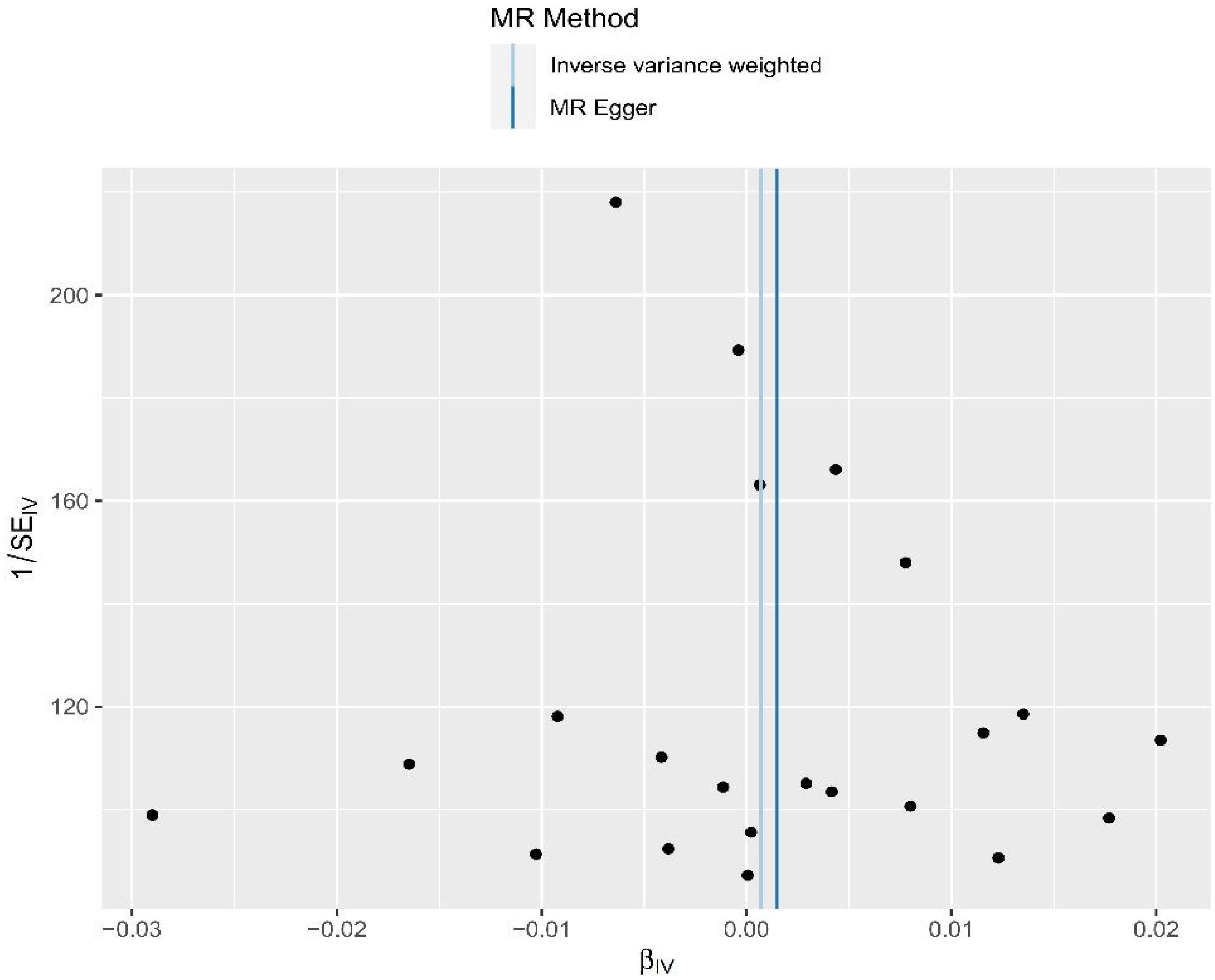

Supplementary Figure-50 Leave-one-out Analysis, Scatter Plot, Forest Plot, and Funnel Plot of Parkinson's Disease on Frailty Index

Supplementary Figure-50A Leave-one-out Analysis

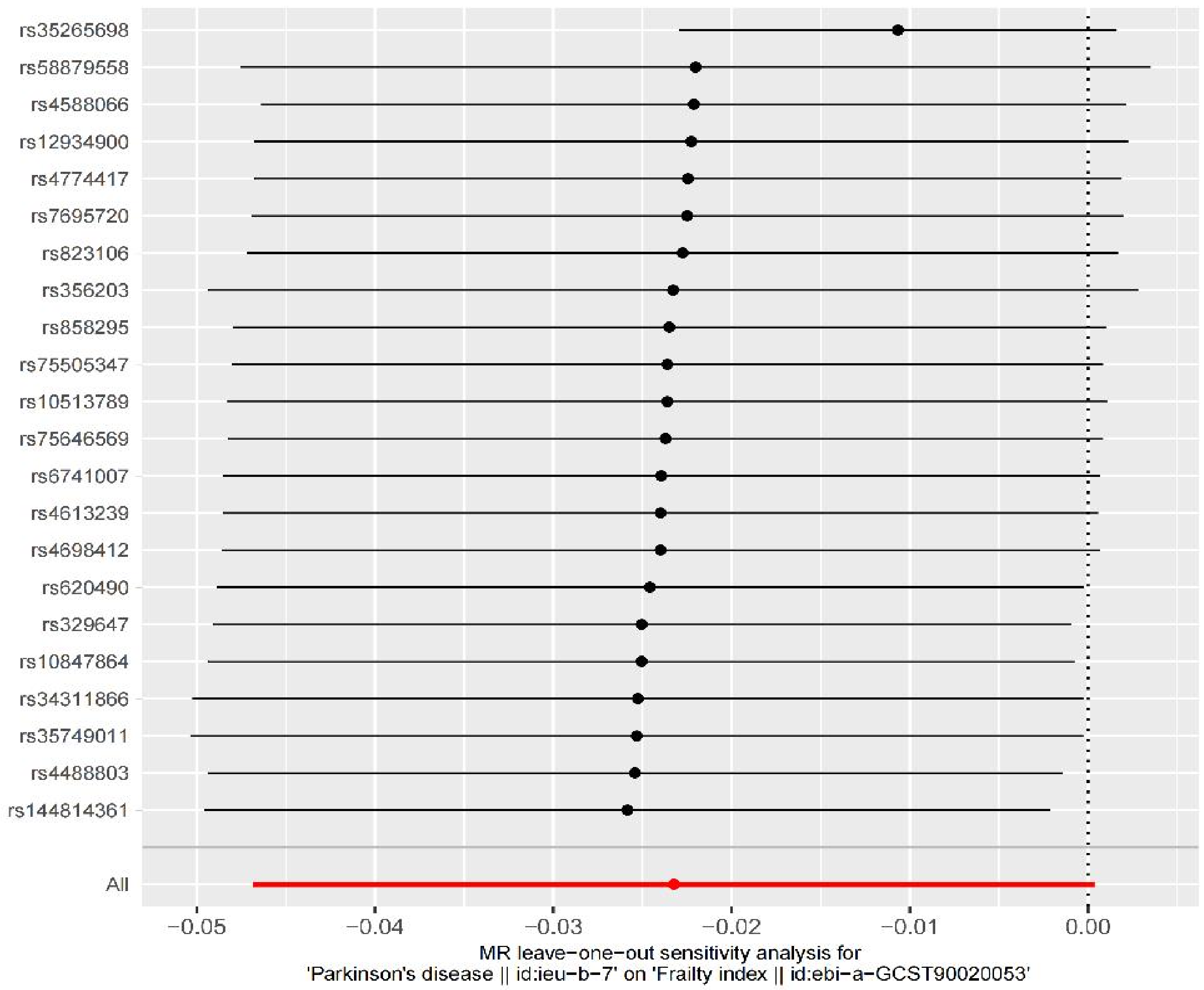

Supplementary Figure-50B Scatter

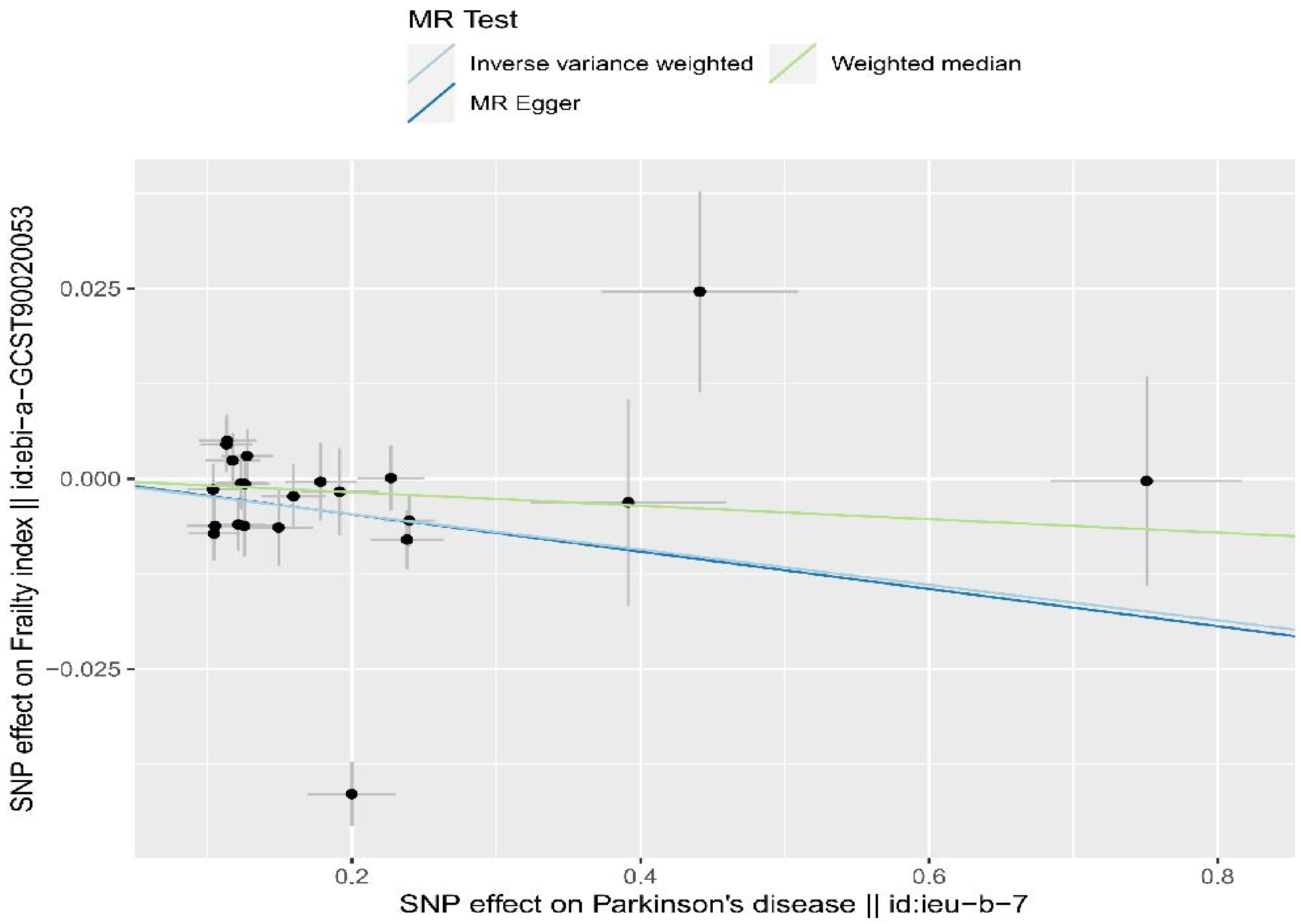

Supplementary Figure-50C Forest Plot

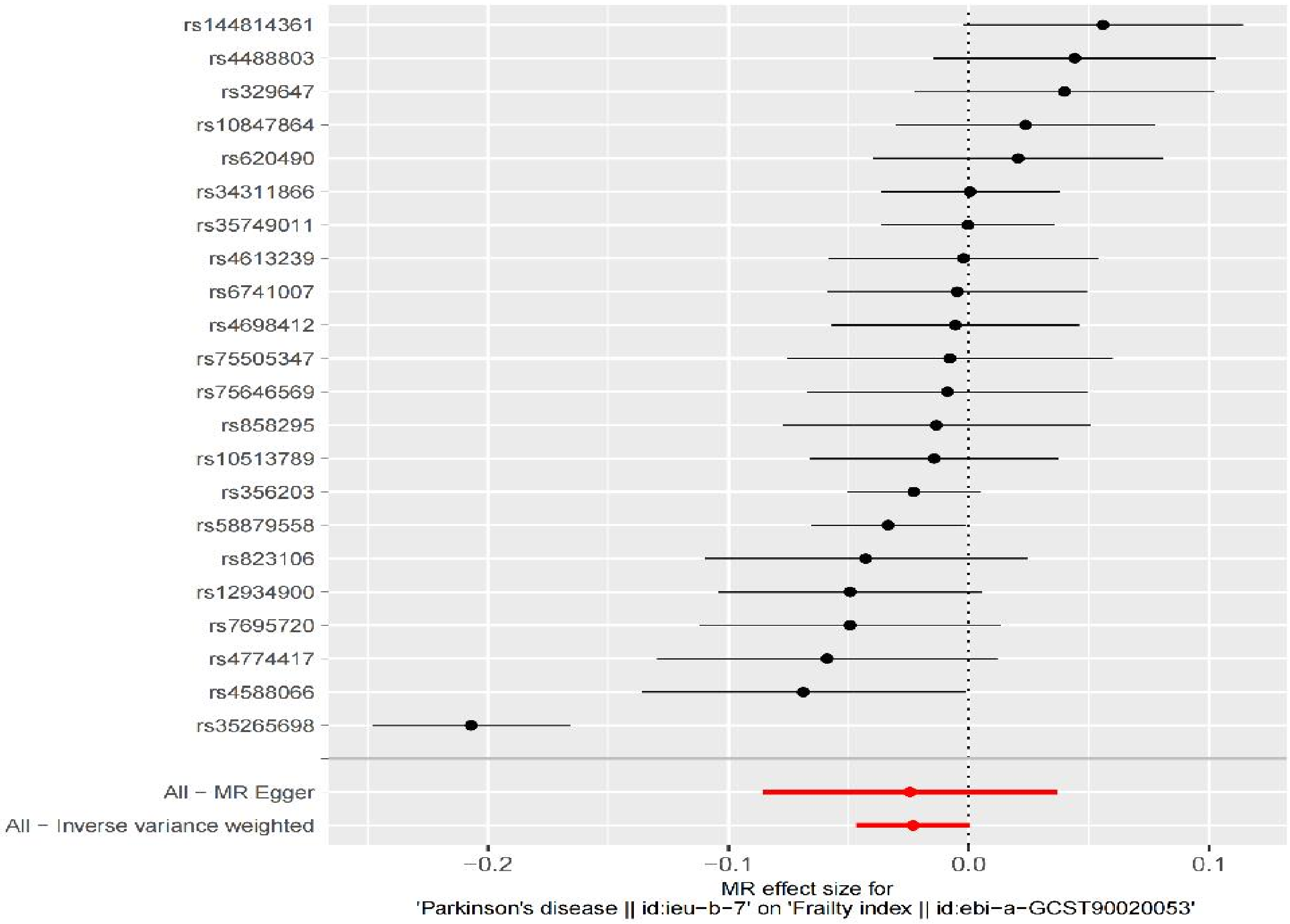

Supplementary Figure-50D Funnel Plot

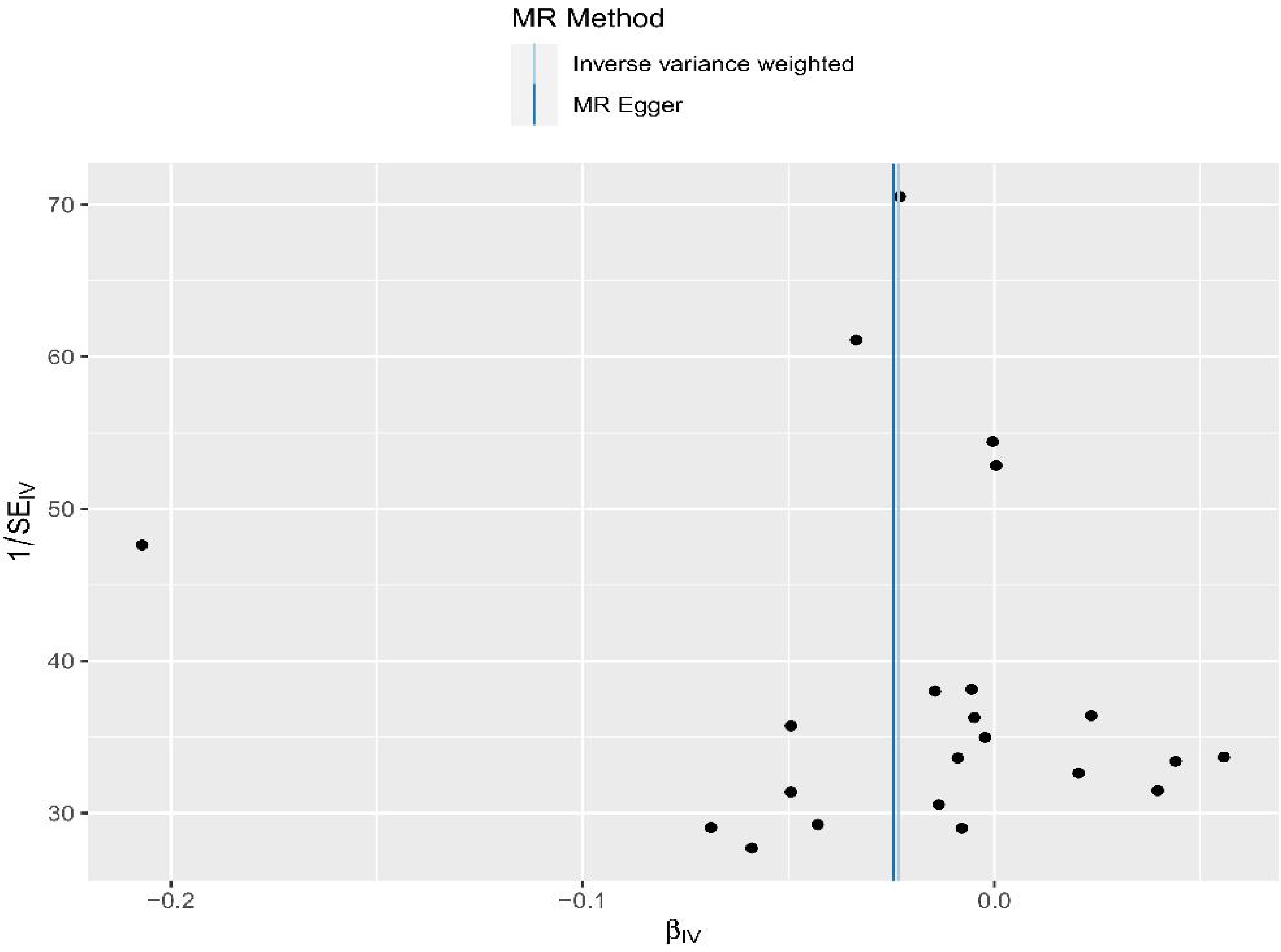

Supplementary Figure-51 Leave-one-out Analysis, Scatter Plot, Forest Plot, and Funnel Plot of Parkinson's Disease on DNA methylation GrimAge acceleration

Supplementary Figure-51A Leave-one-out Analysis

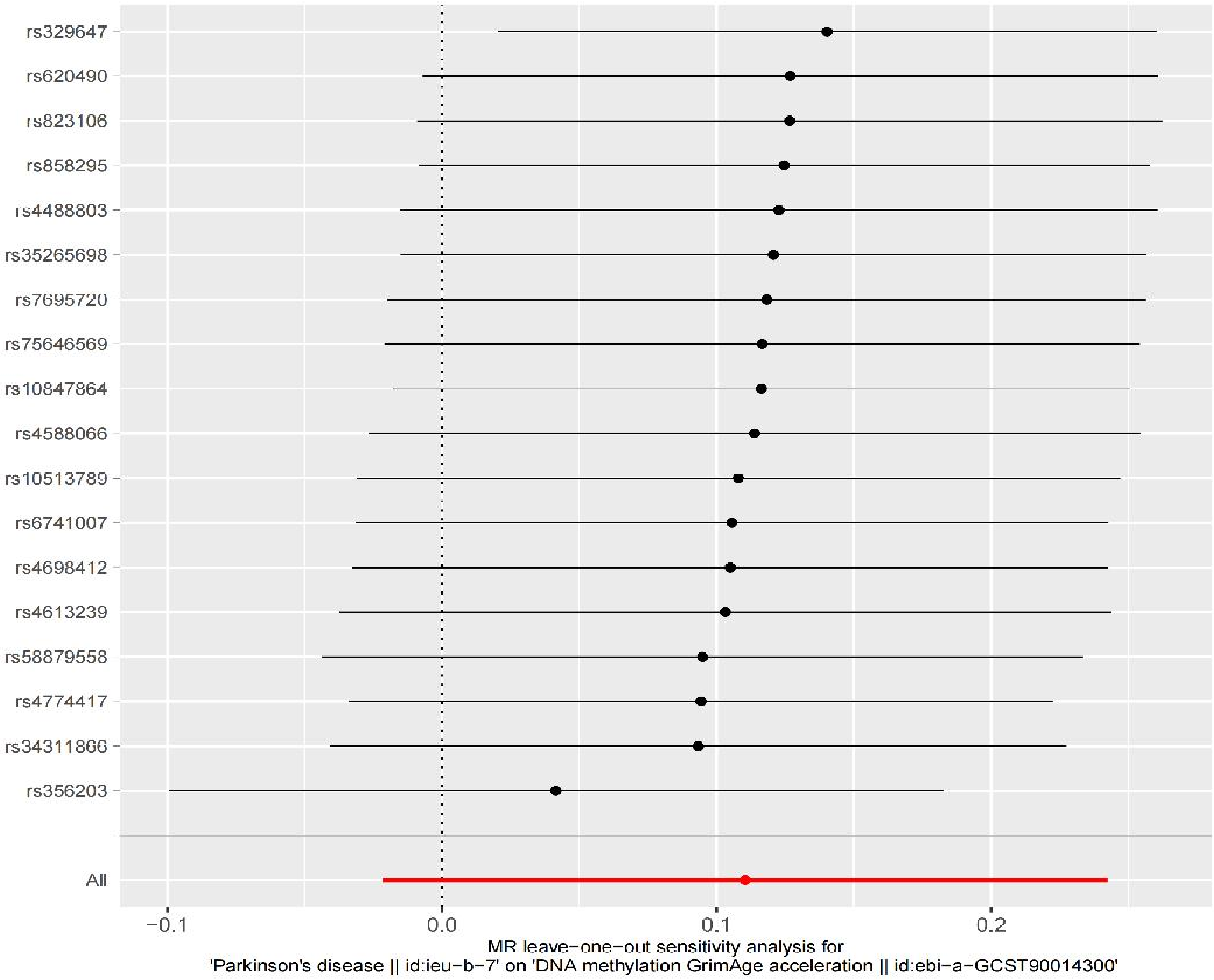

Supplementary Figure-51B Scatter

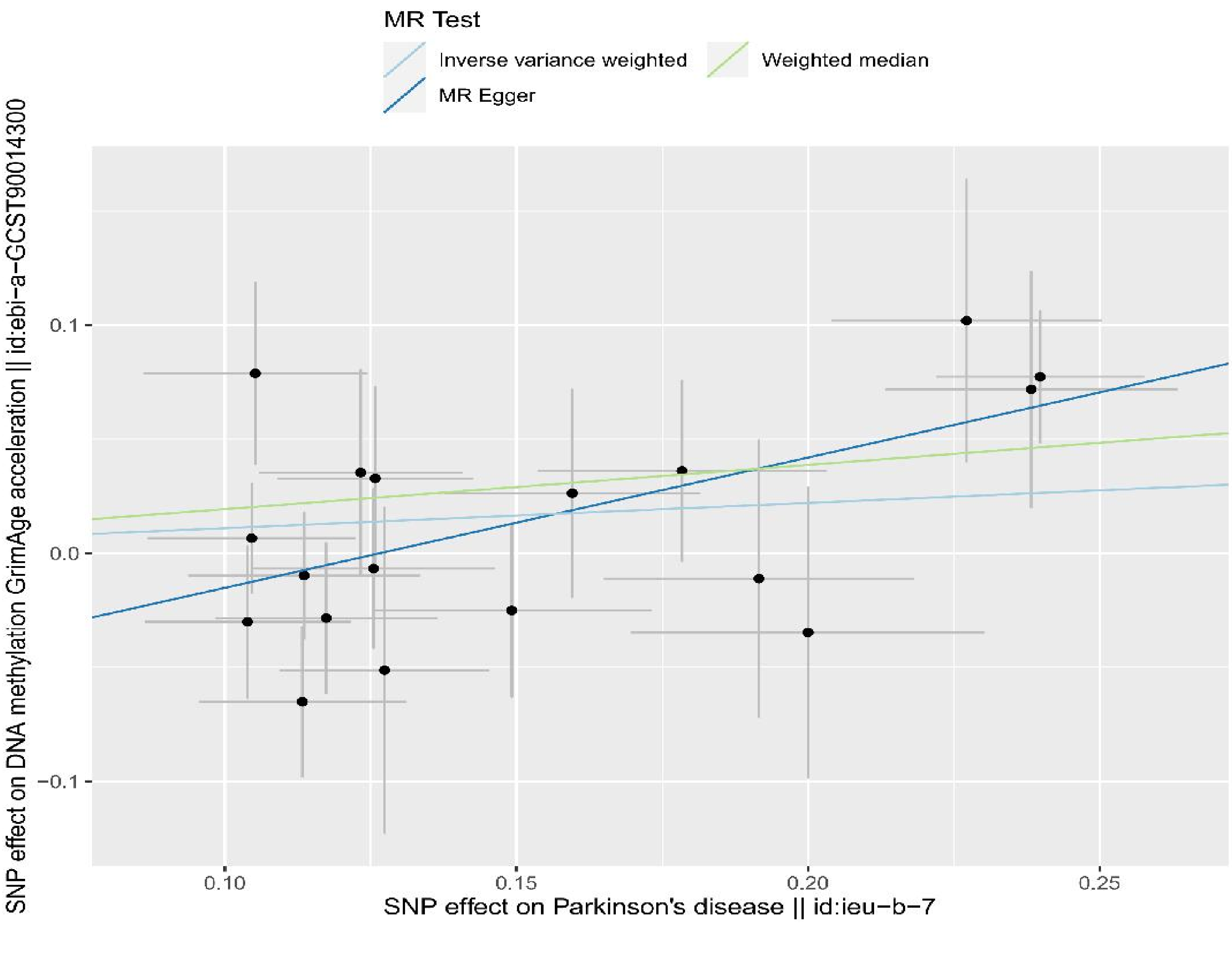

Supplementary Figure-51C Forest Plot

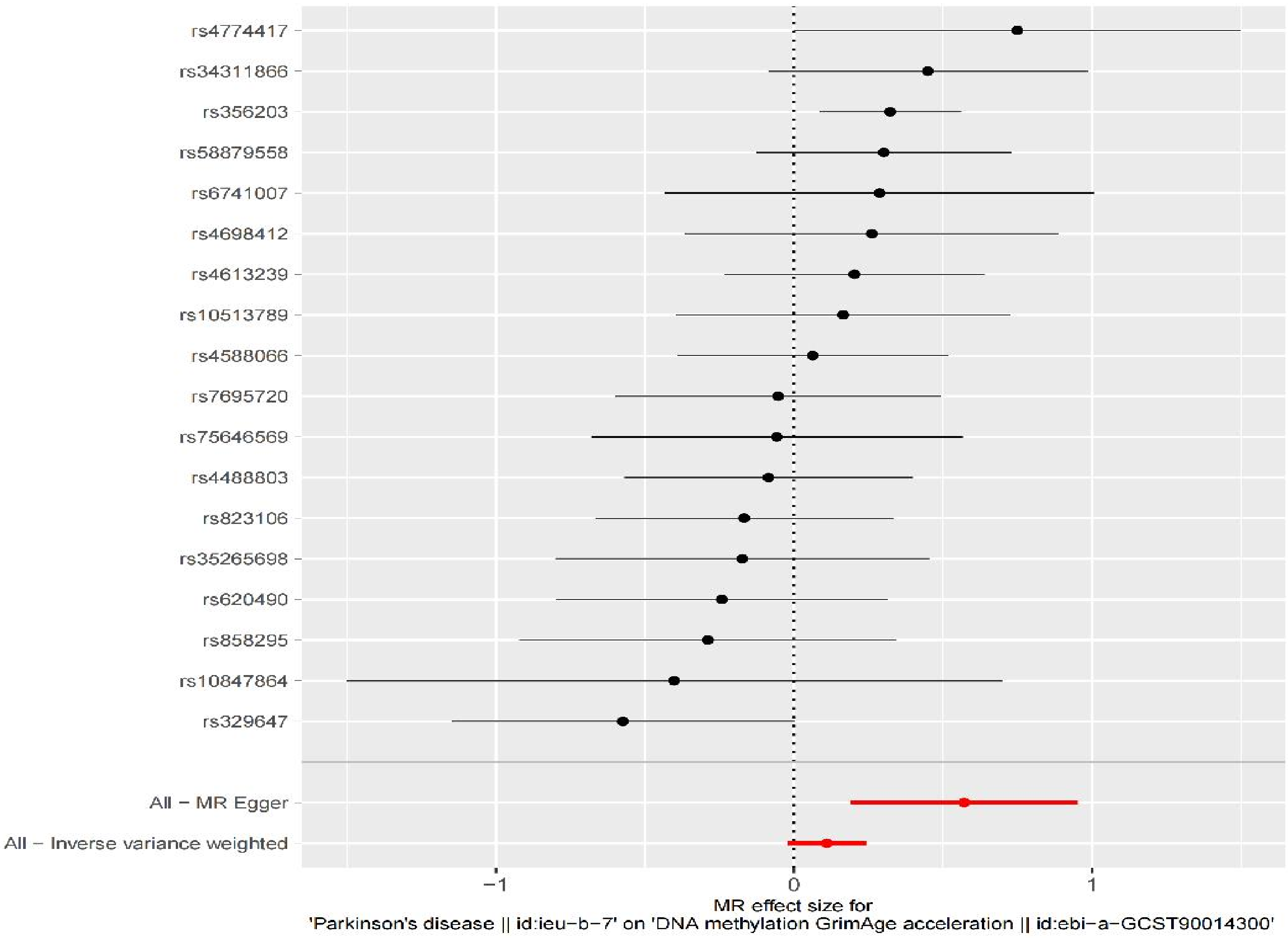

Supplementary Figure-51D Funnel Plot

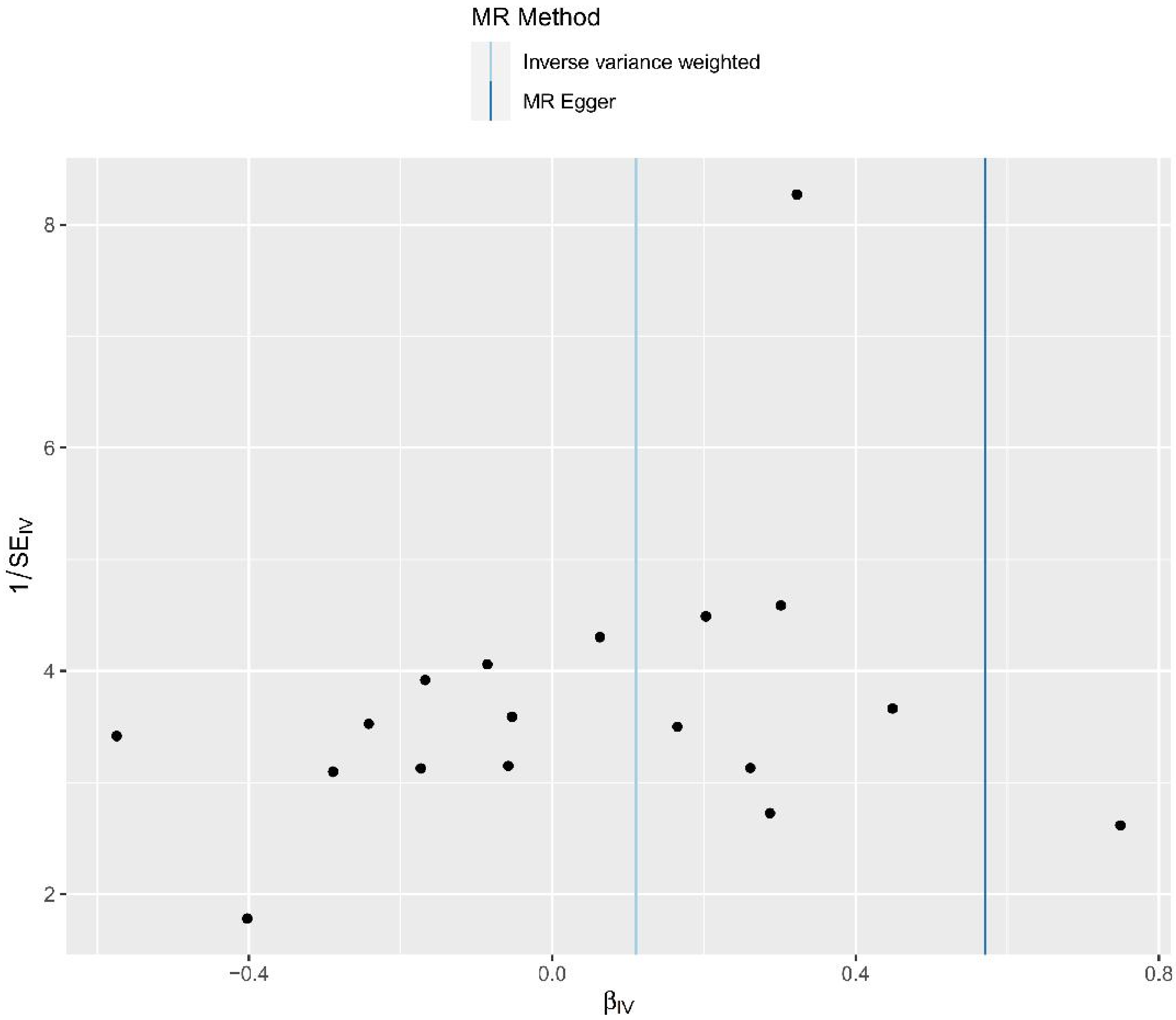

Supplementary Figure-52 Leave-one-out Analysis, Scatter Plot, Forest Plot, and Funnel Plot of Parkinson's Disease on Telomere length

Supplementary Figure-52A Leave-one-out Analysis

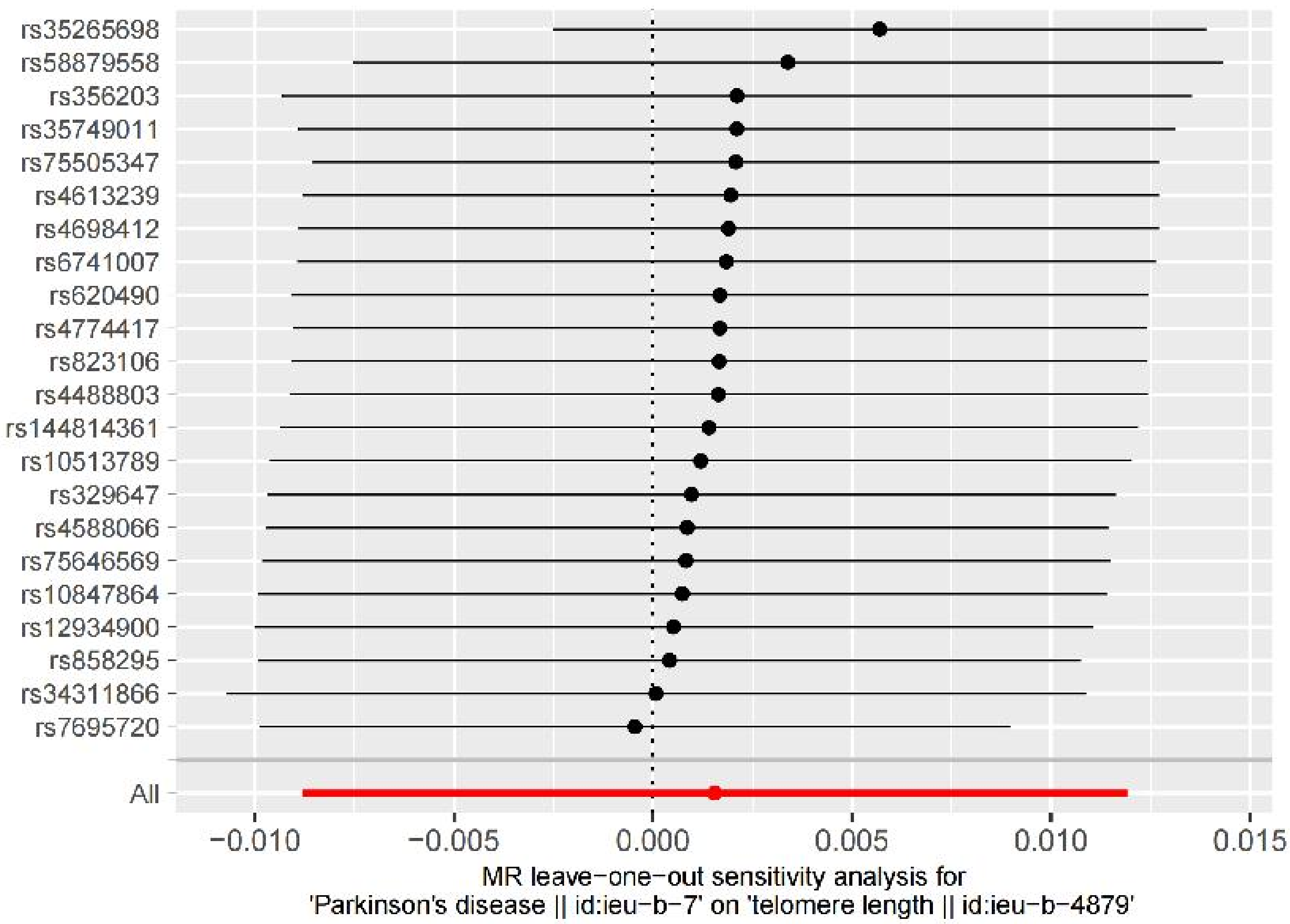

Supplementary Figure-52B Scatter

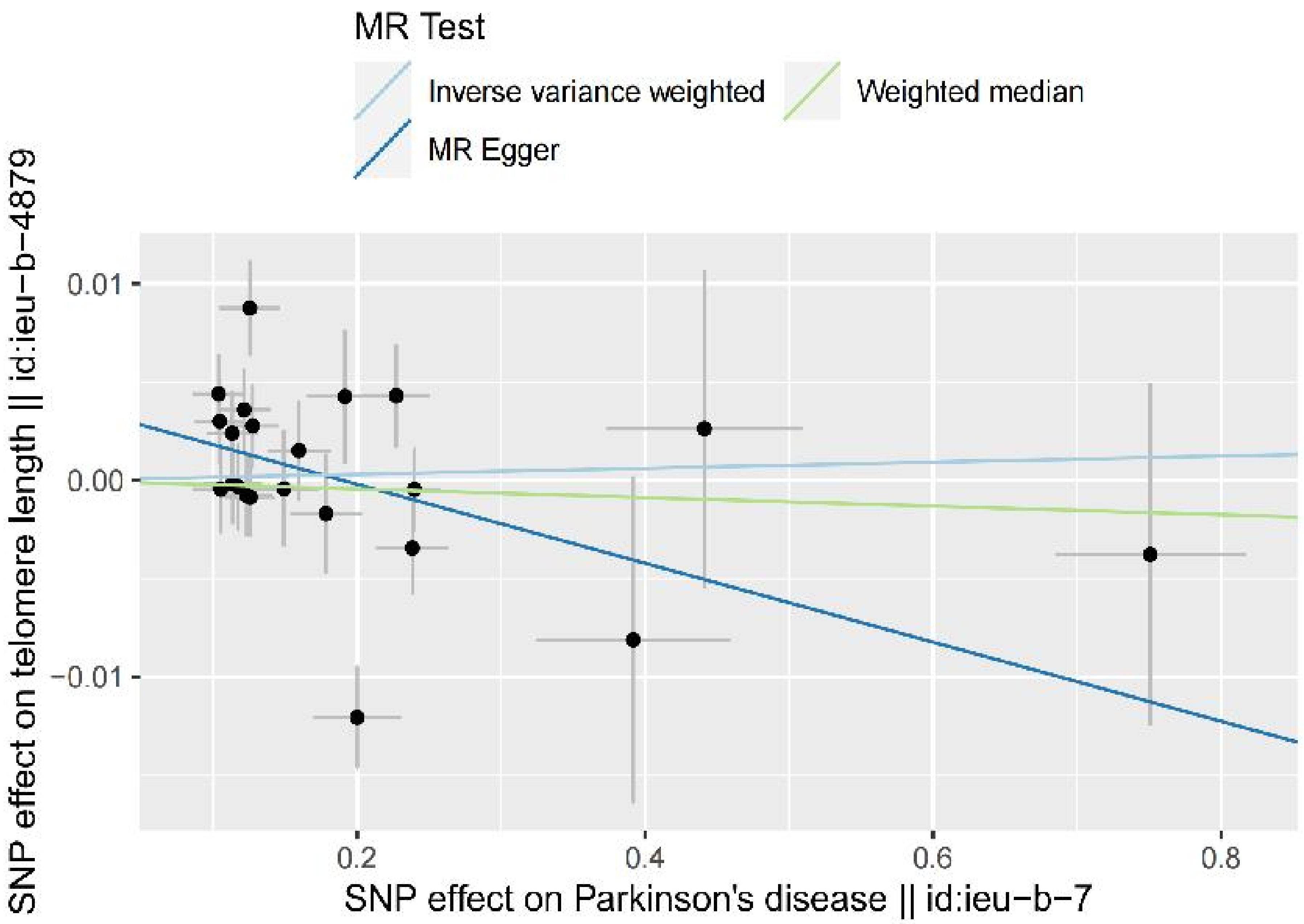

Supplementary Figure-52C Forest Plot

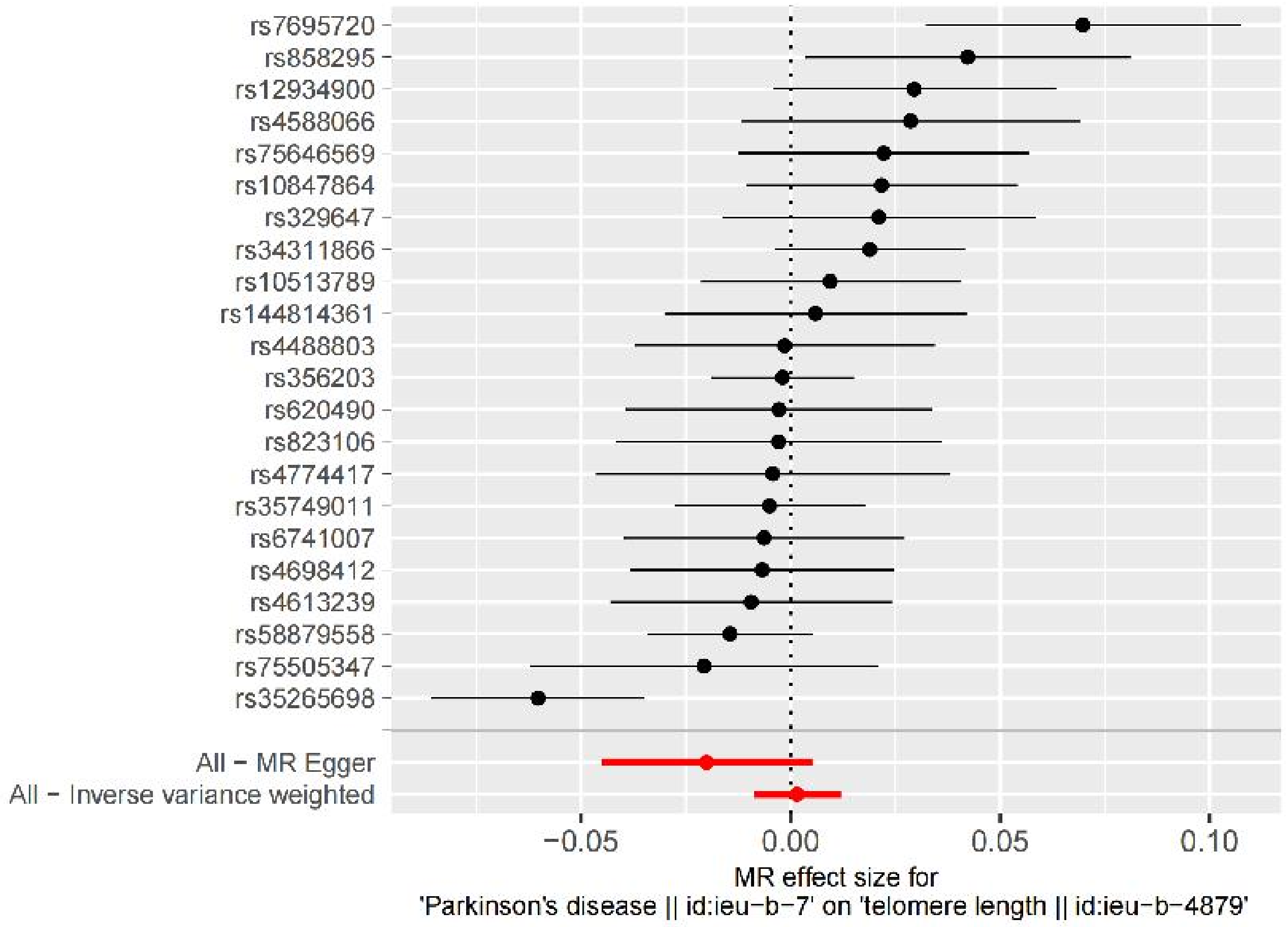

Supplementary Figure-52D Funnel Plot

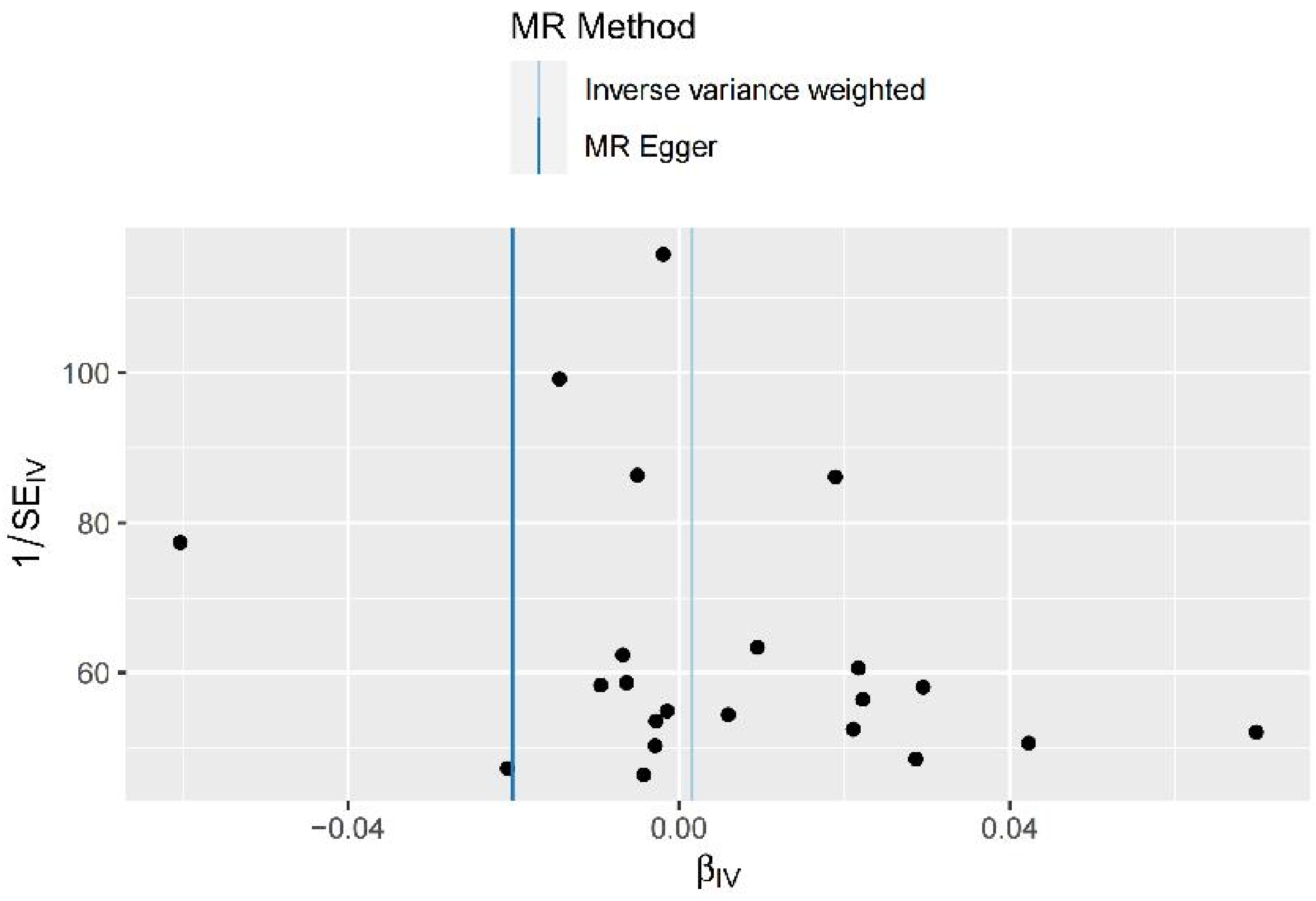

Supplementary Figure-53 Leave-one-out Analysis, Scatter Plot, Forest Plot, and Funnel Plot of telomere length on Alzheimer's disease

Supplementary Figure-53A Leave-one-out Analysis

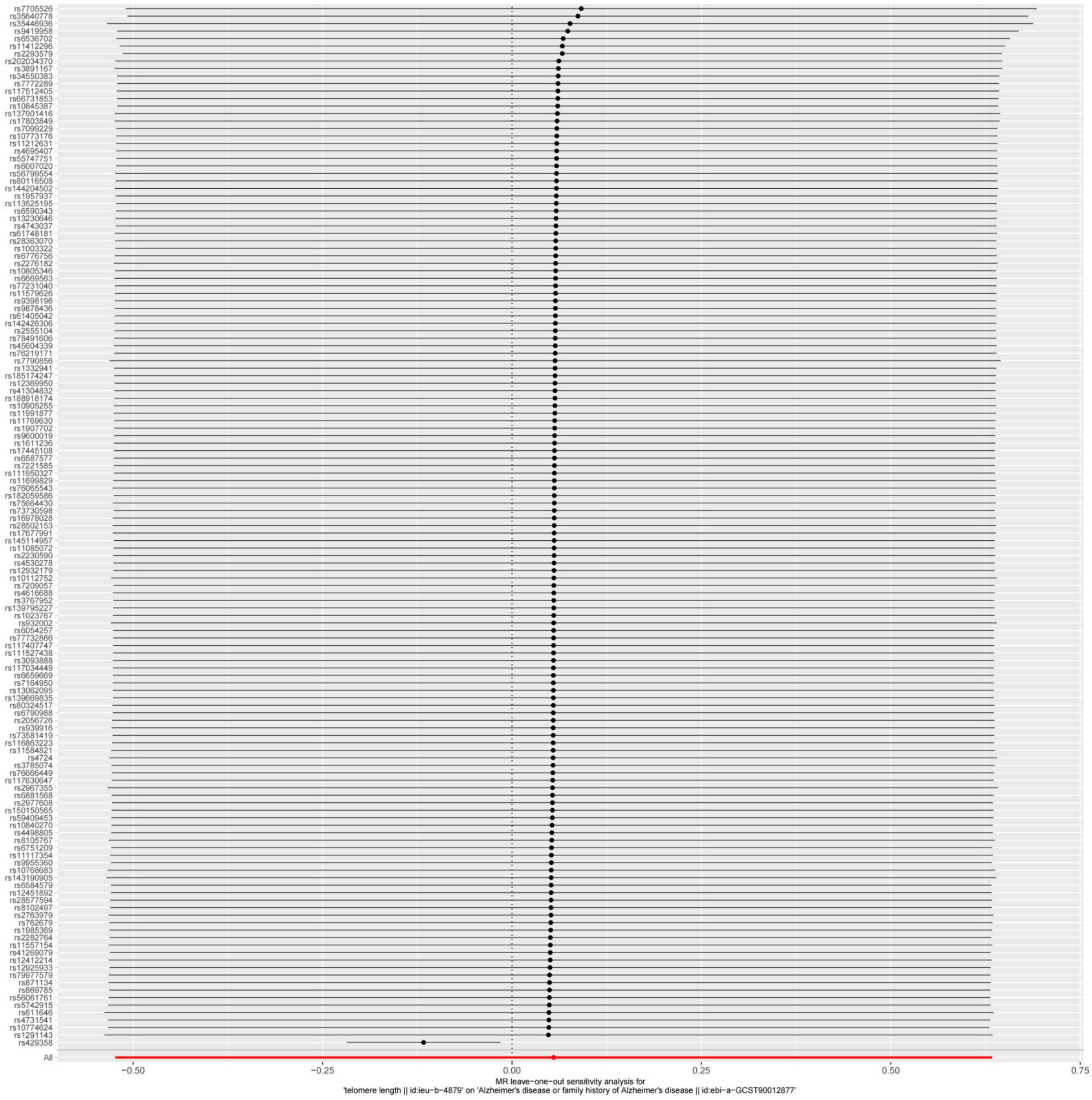

Supplementary Figure-53B Scatter

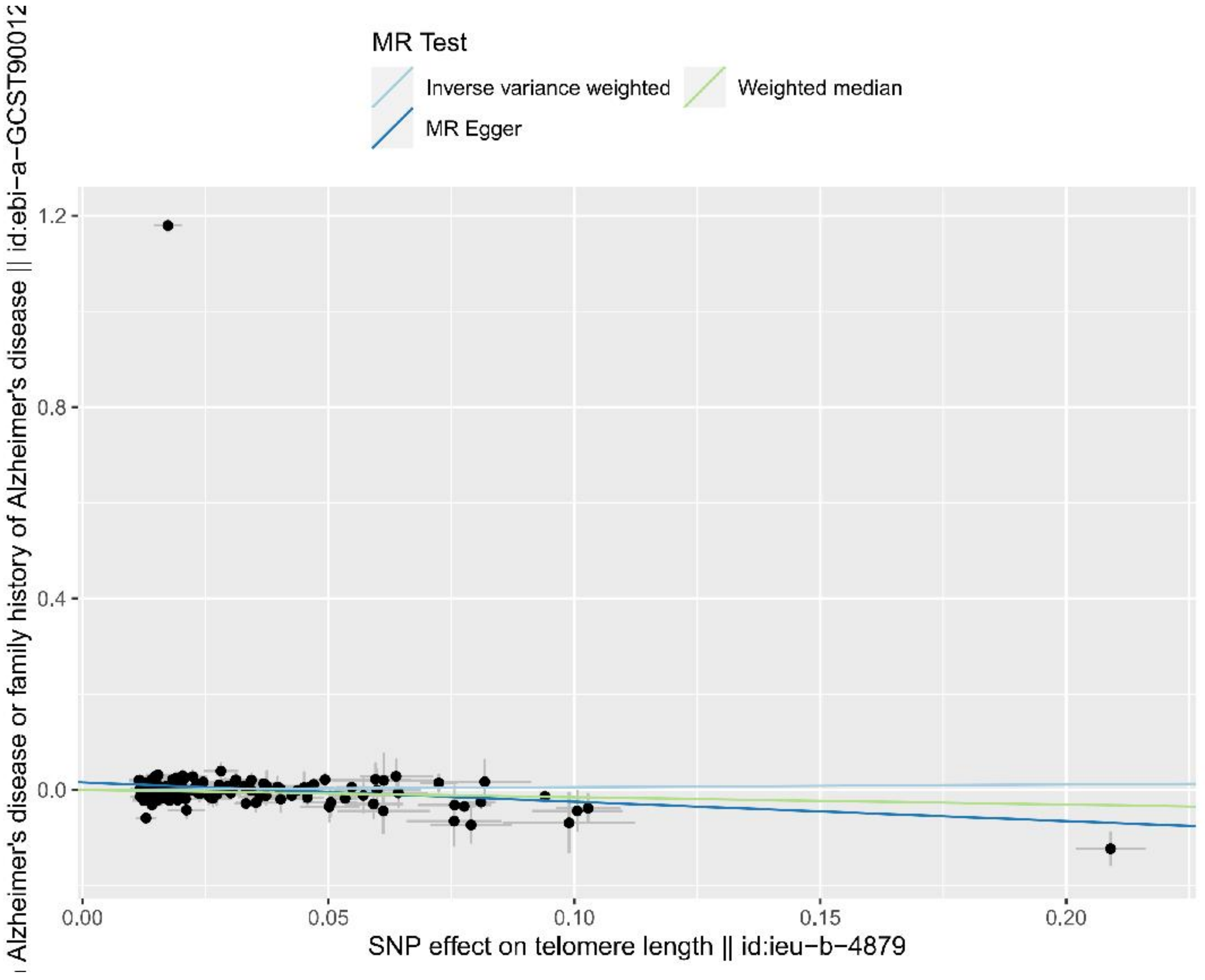

Supplementary Figure-53C Forest Plot

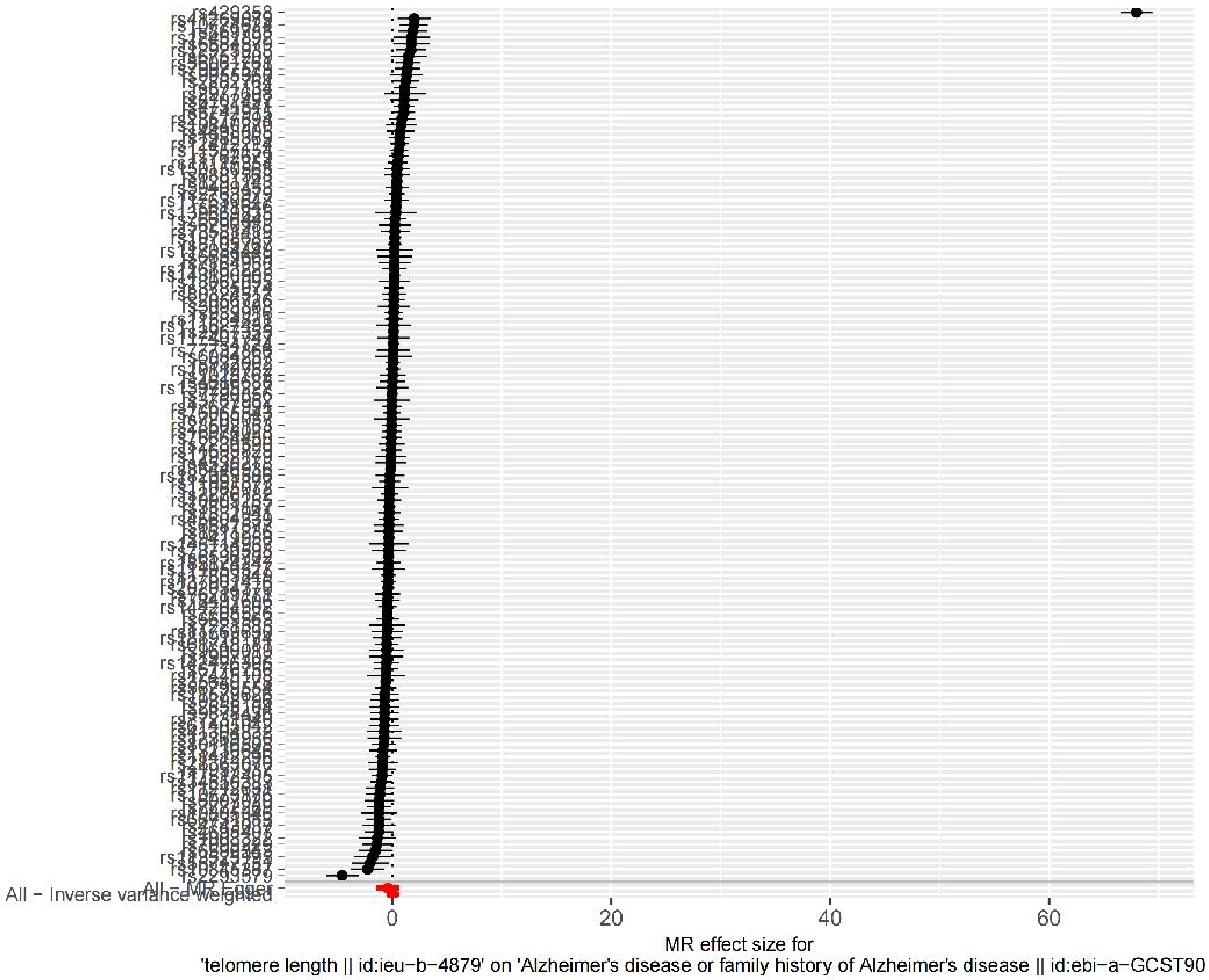

Supplementary Figure-53D Funnel Plot

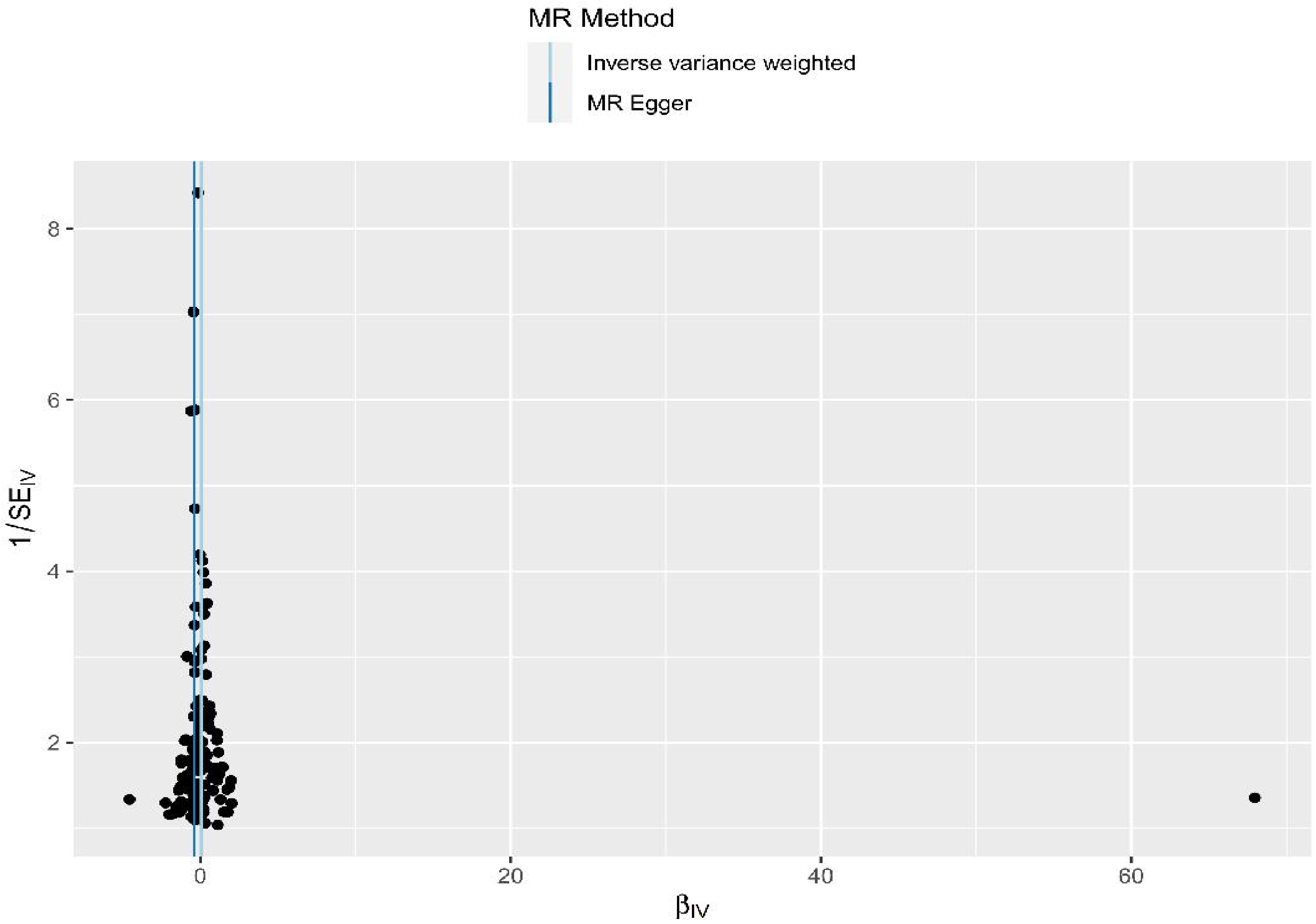

Supplementary Figure-54 Leave-one-out Analysis, Scatter Plot, Forest Plot, and Funnel Plot of telomere length on Alzheimer's disease (Corrected)

Supplementary Figure-54A Leave-one-out Analysis

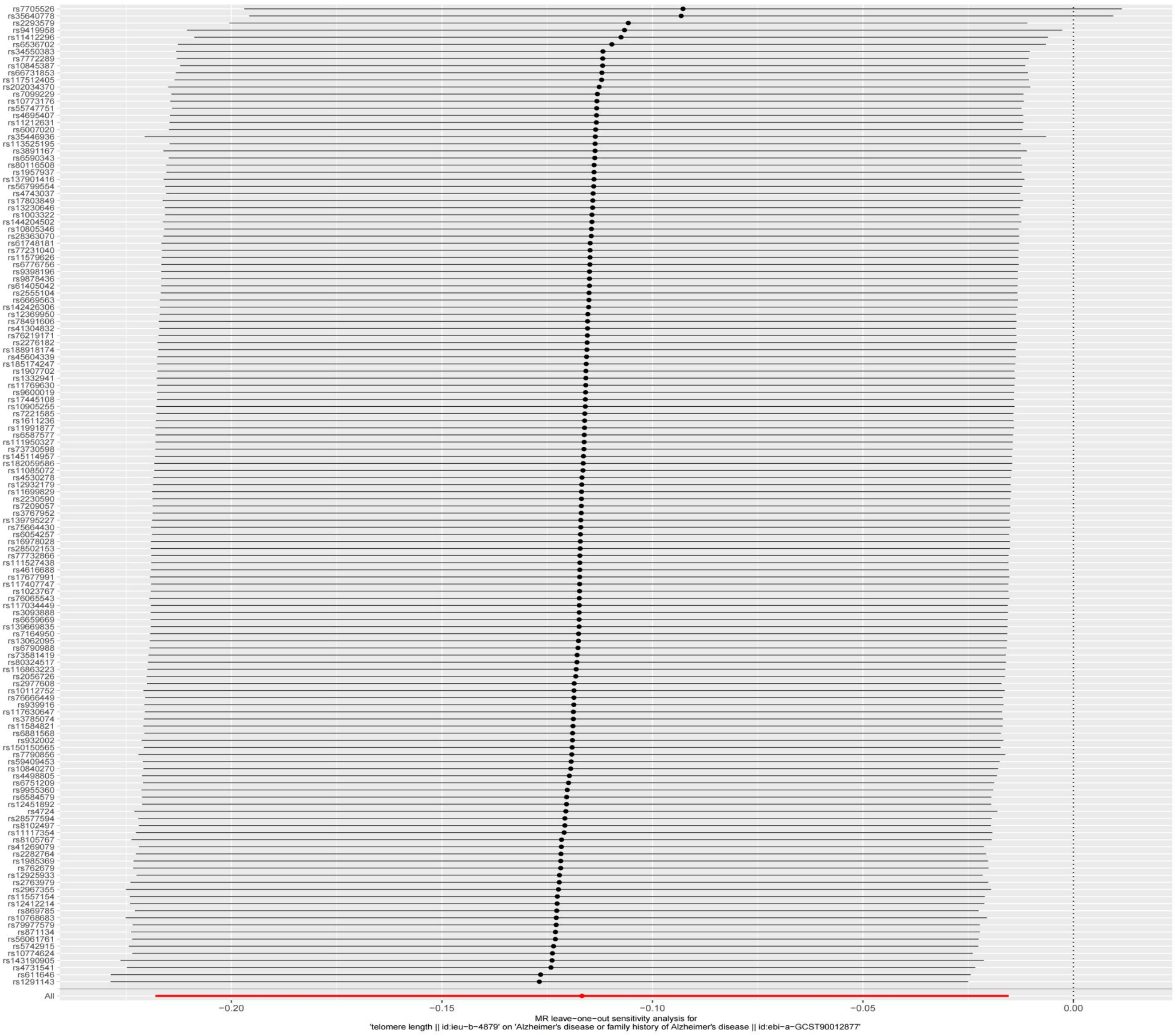

Supplementary Figure-54B Scatter

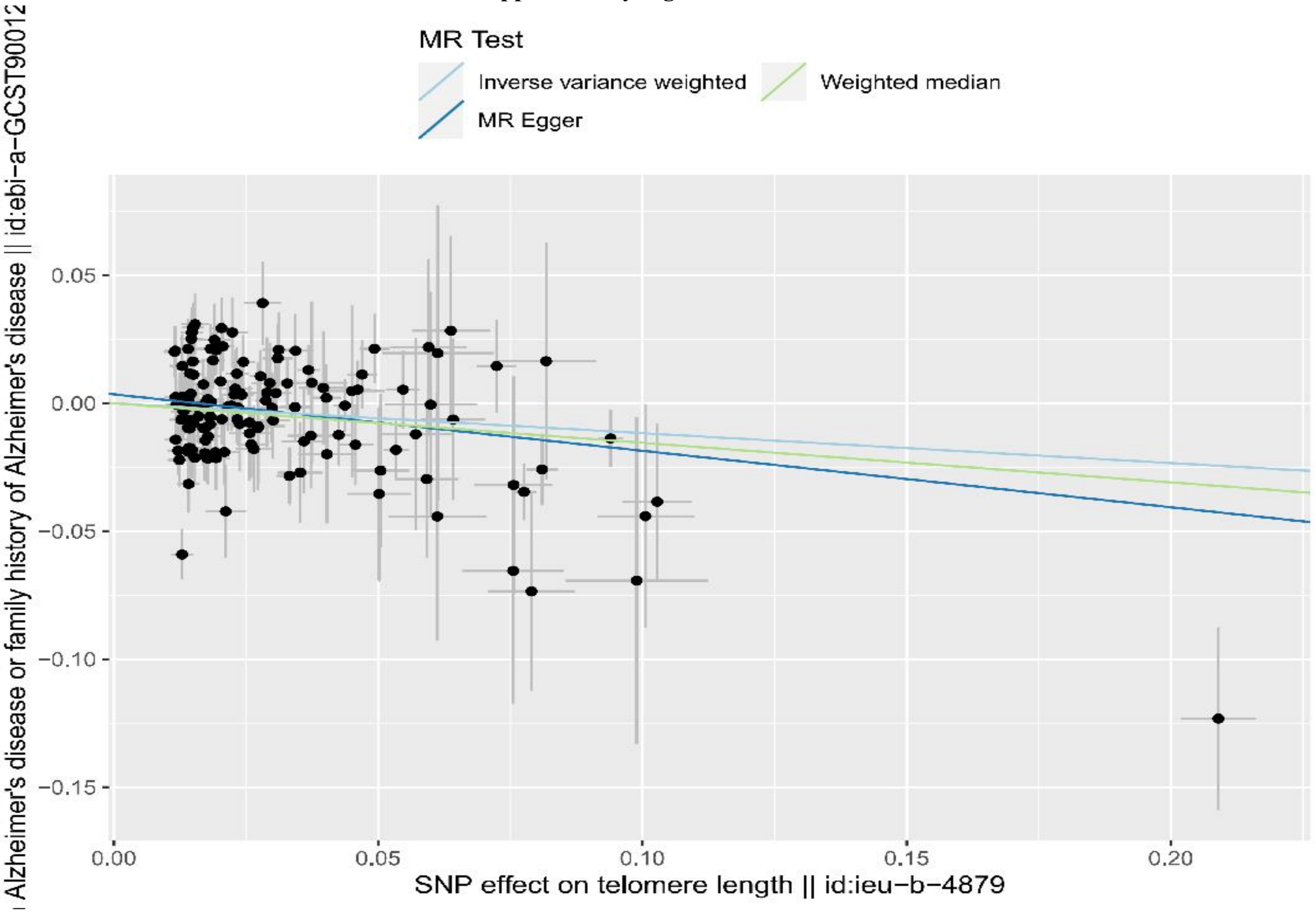

Supplementary Figure-54C Forest Plot

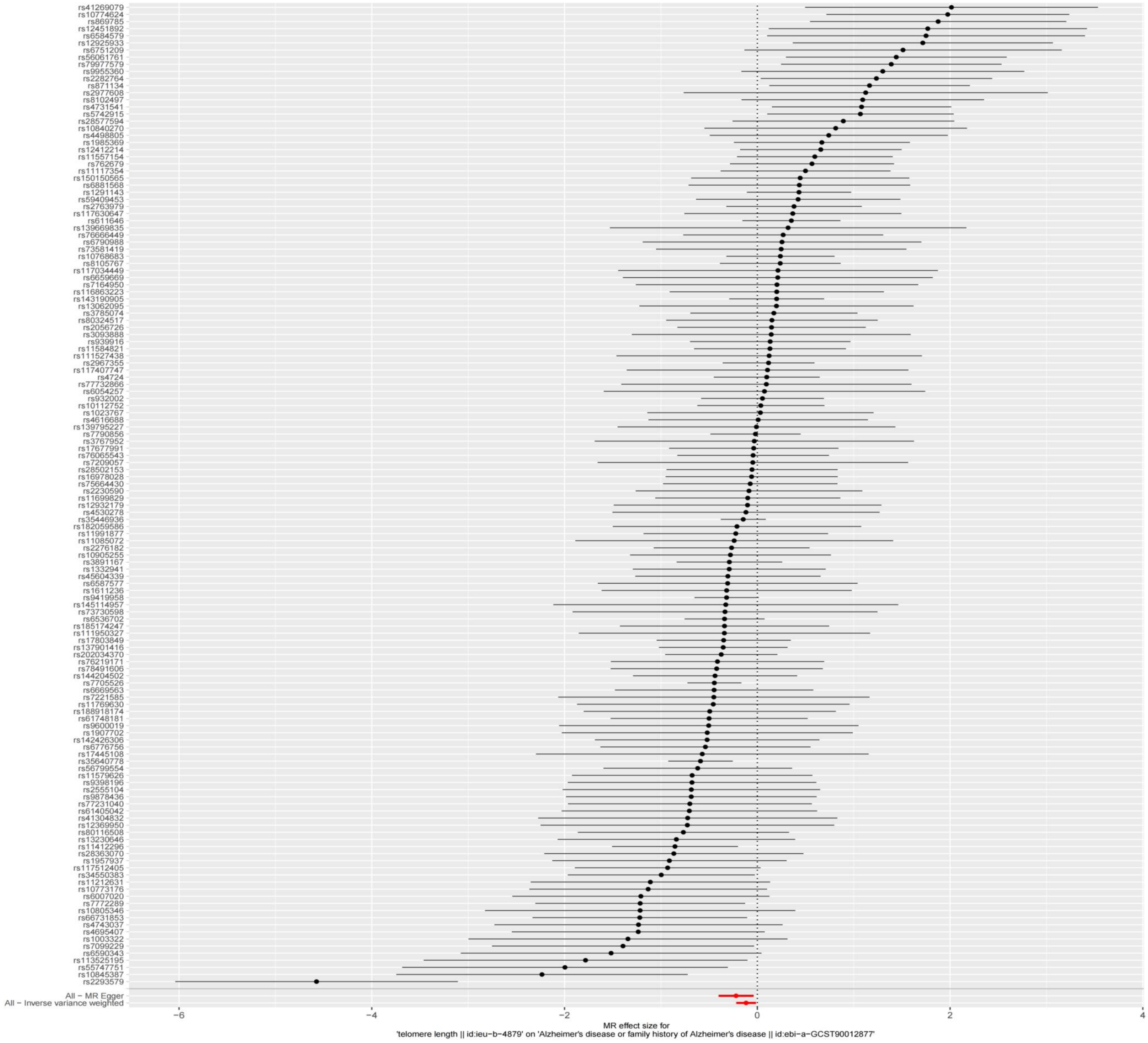

Supplementary Figure-54D Funnel Plot

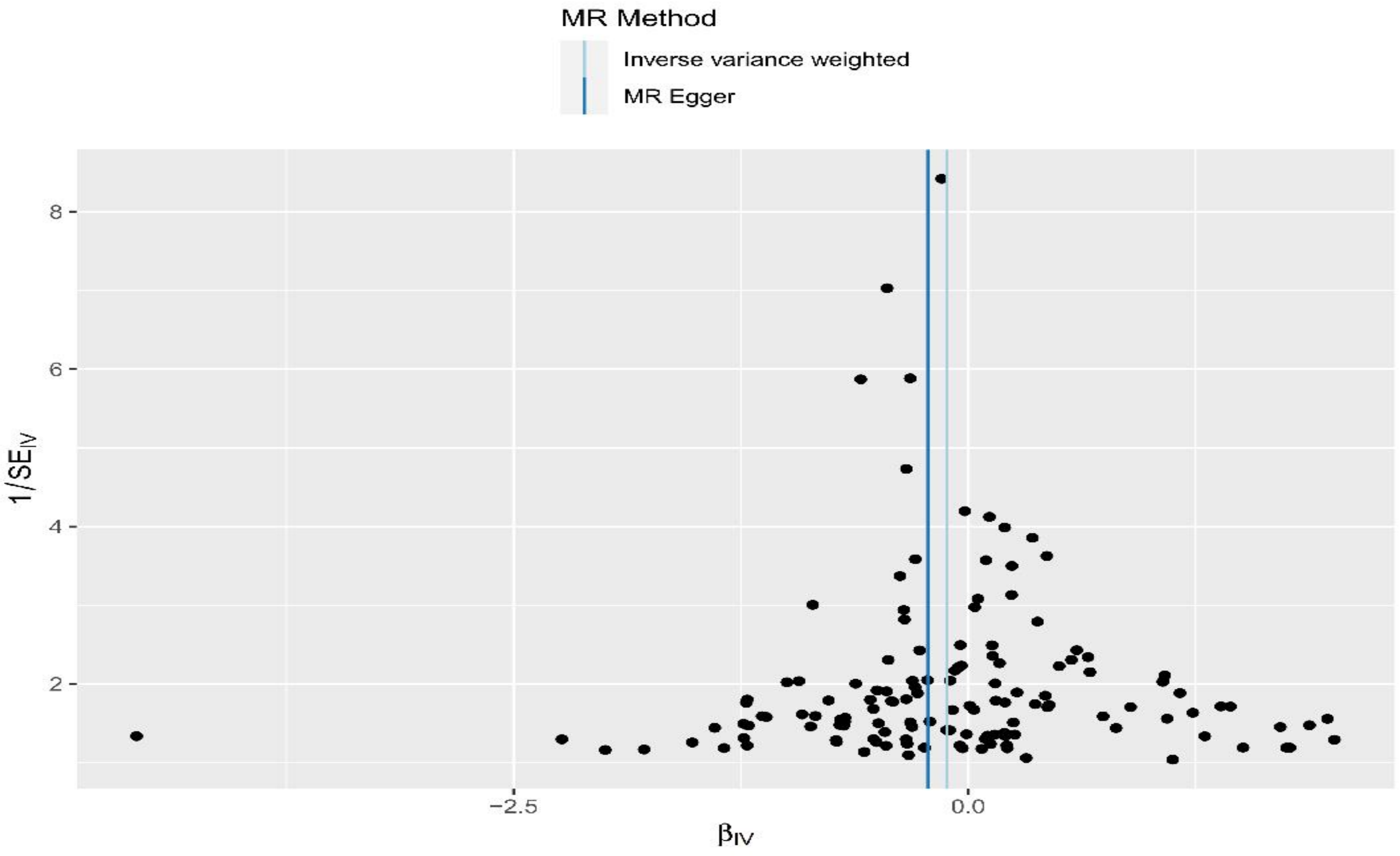

Supplementary Figure-55 Leave-one-out Analysis, Scatter Plot, Forest Plot, and Funnel Plot of telomere length on All Glioma

Supplementary Figure-55A Leave-one-out Analysis

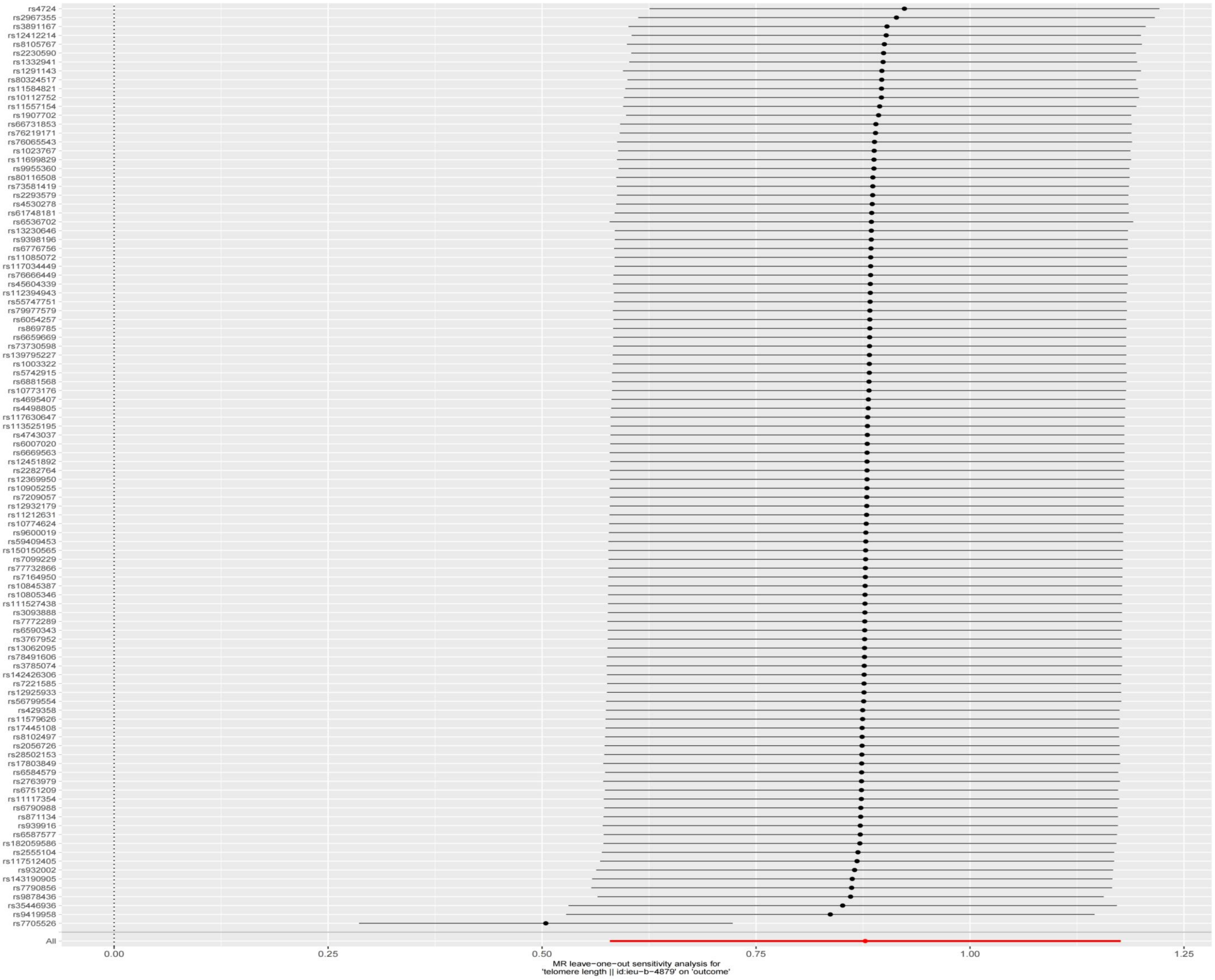

Supplementary Figure-55B Scatter

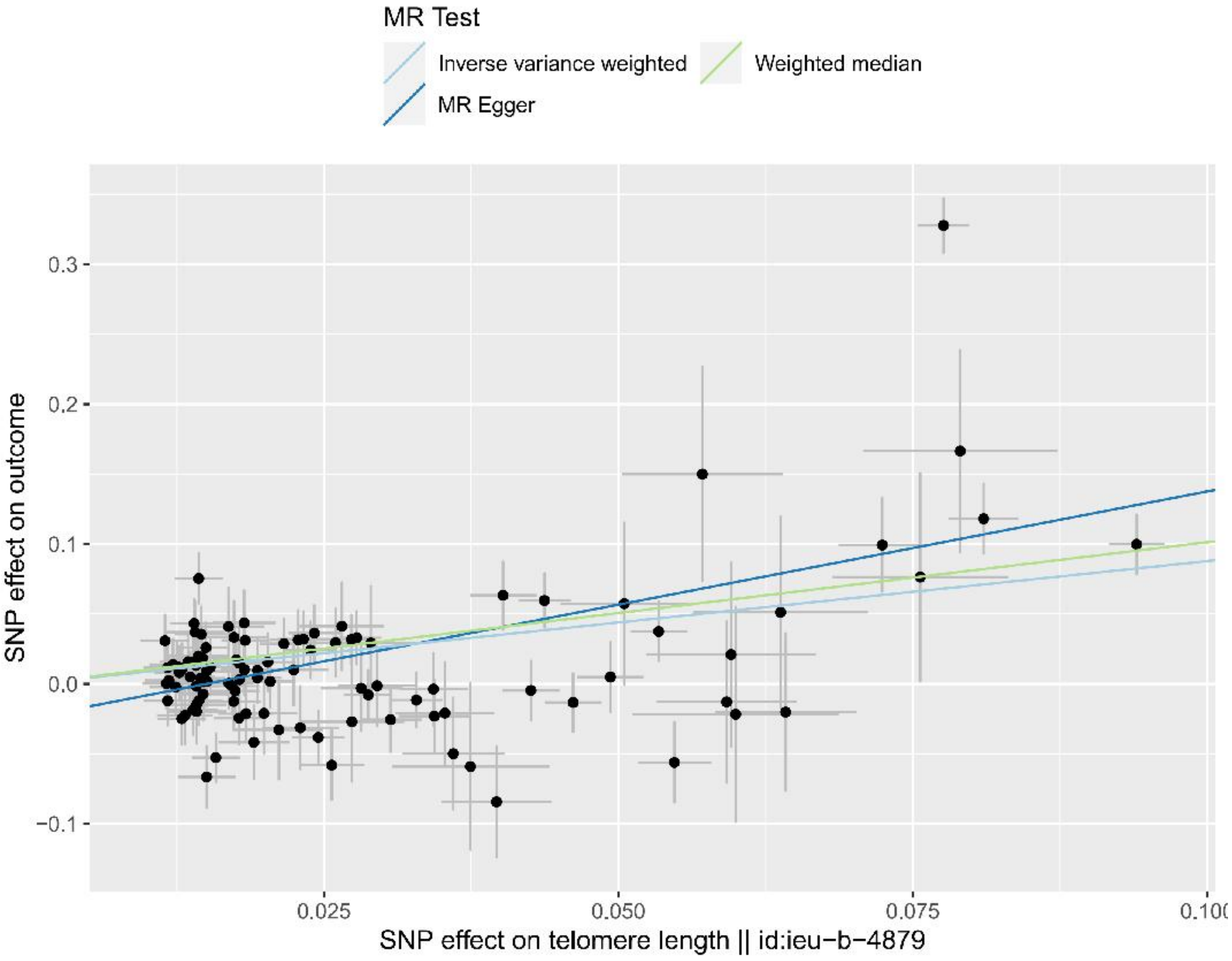

Supplementary Figure-55C Forest Plot

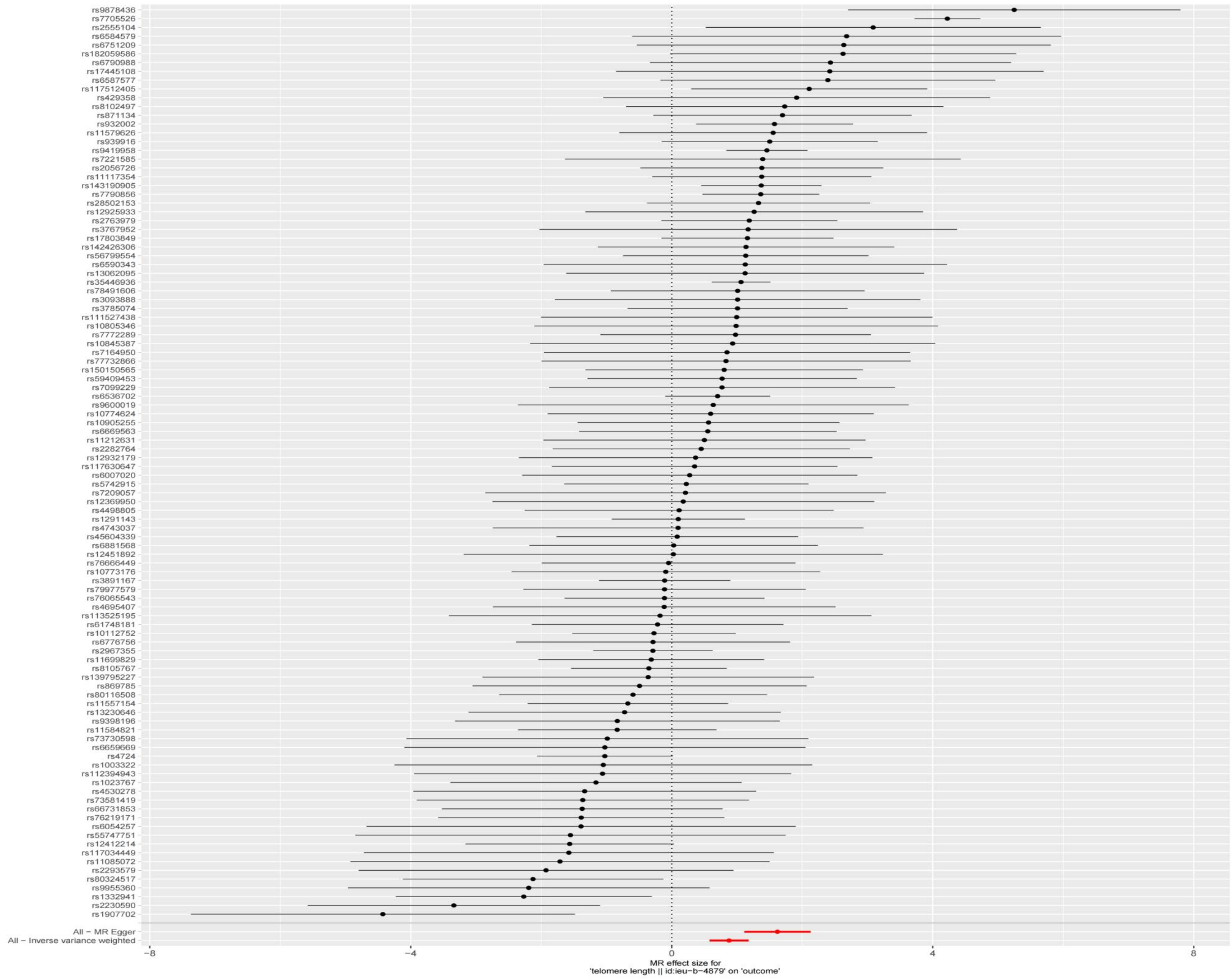

Supplementary Figure-55D Funnel Plot

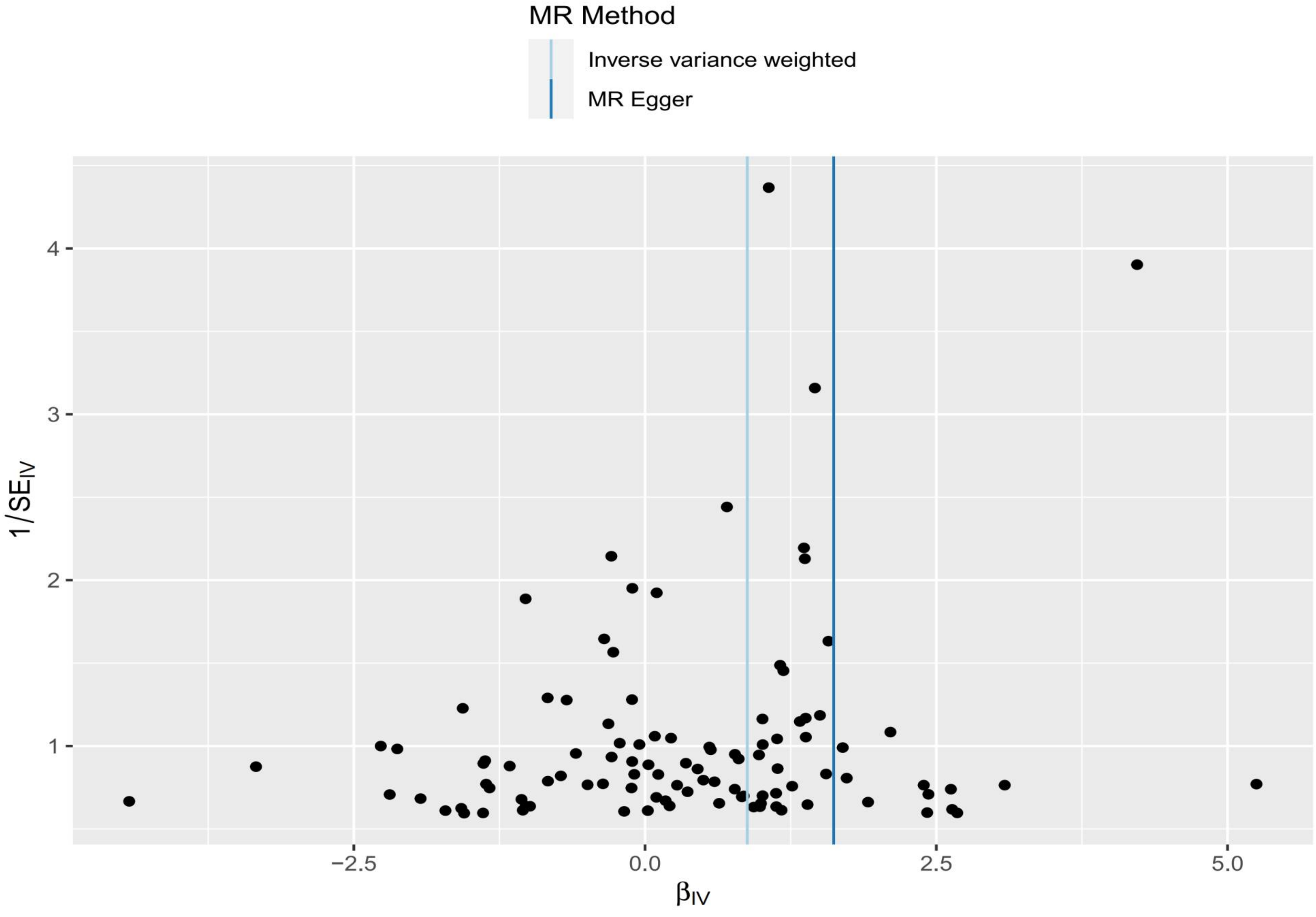

Supplementary Figure-56 Leave-one-out Analysis, Scatter Plot, Forest Plot, and Funnel Plot of Telomere length on Amyotrophic Lateral Sclerosis

Supplementary Figure-56A Leave-one-out Analysis

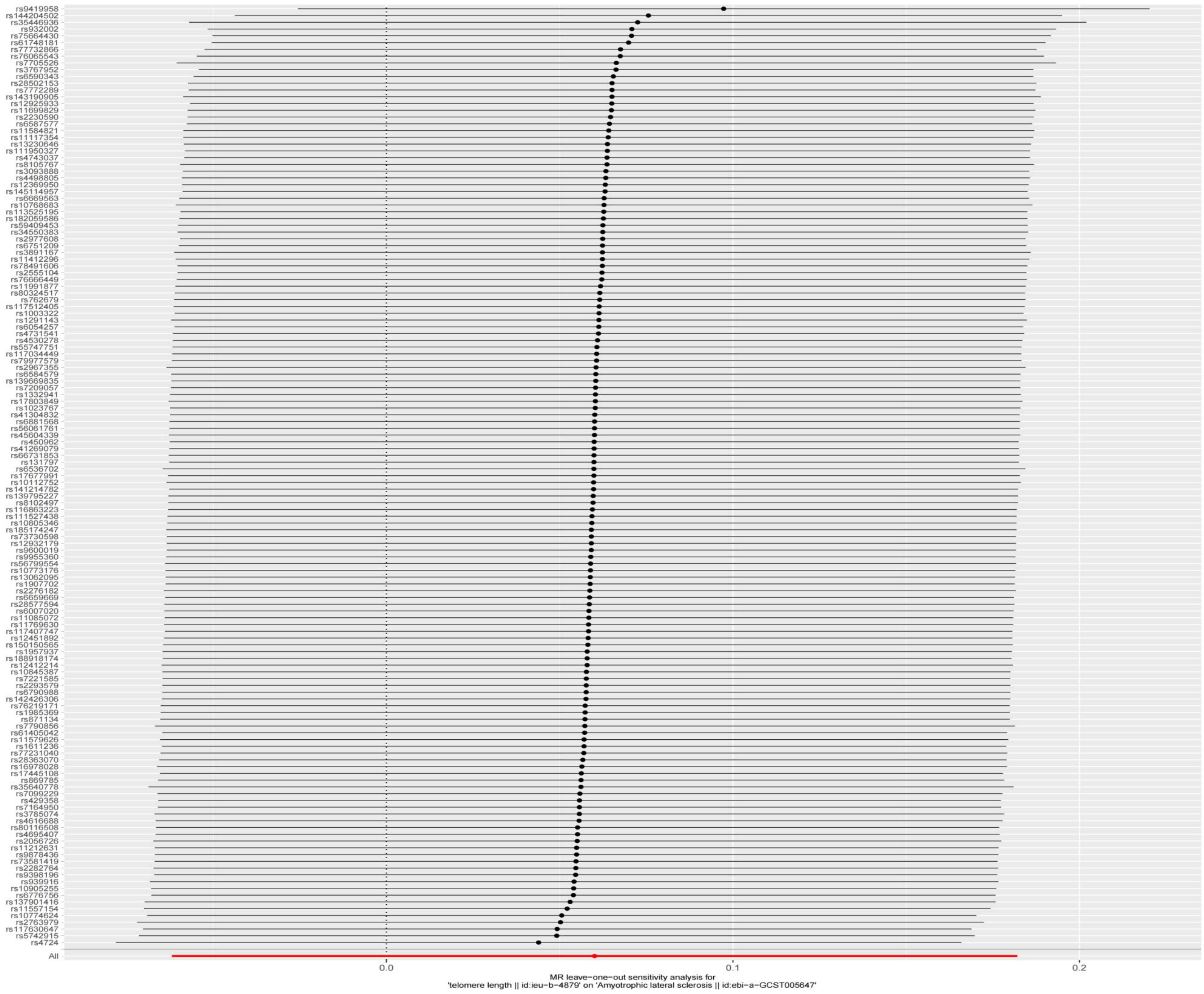

Supplementary Figure-56B Scatter

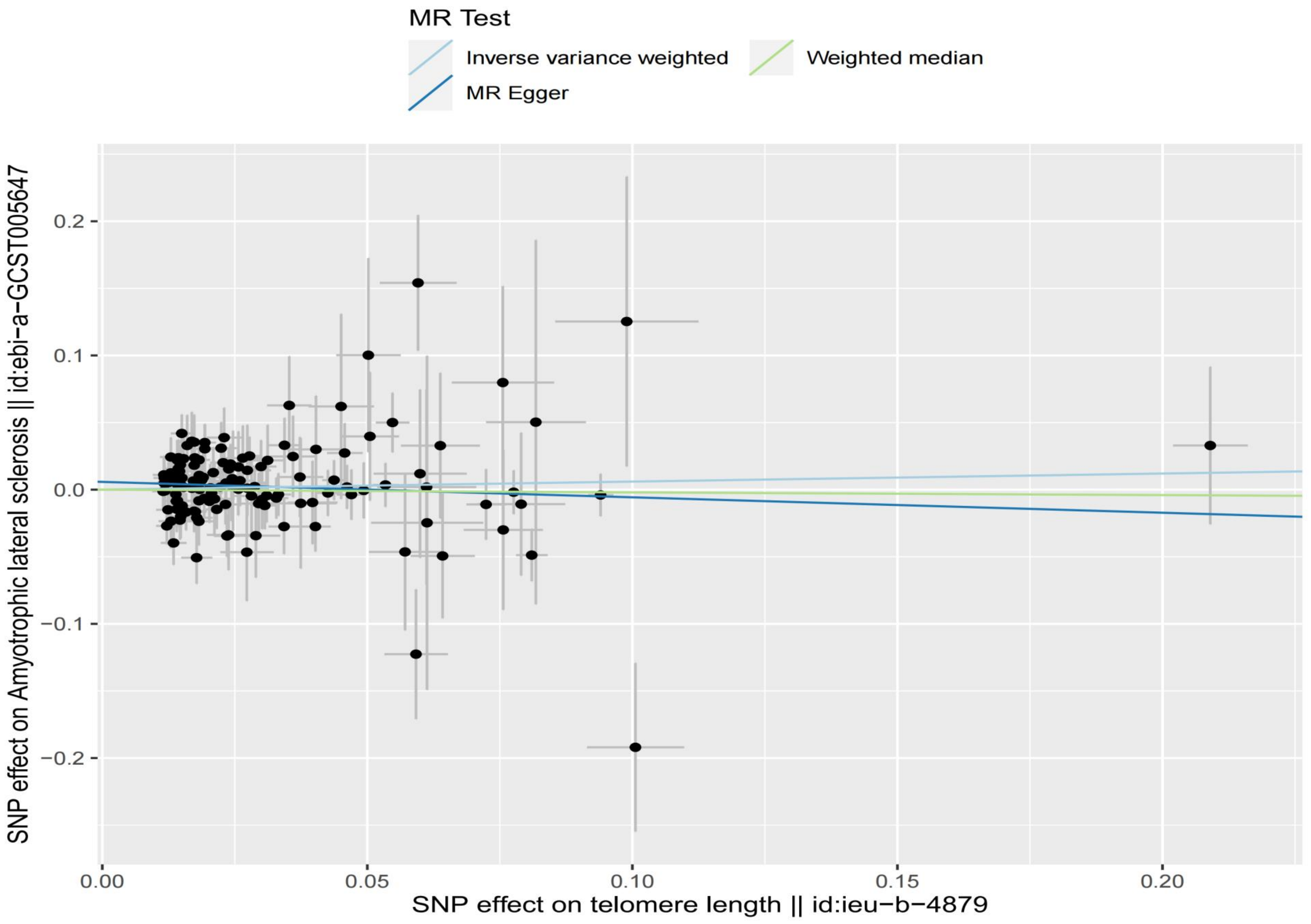

Supplementary Figure-56C Forest Plot

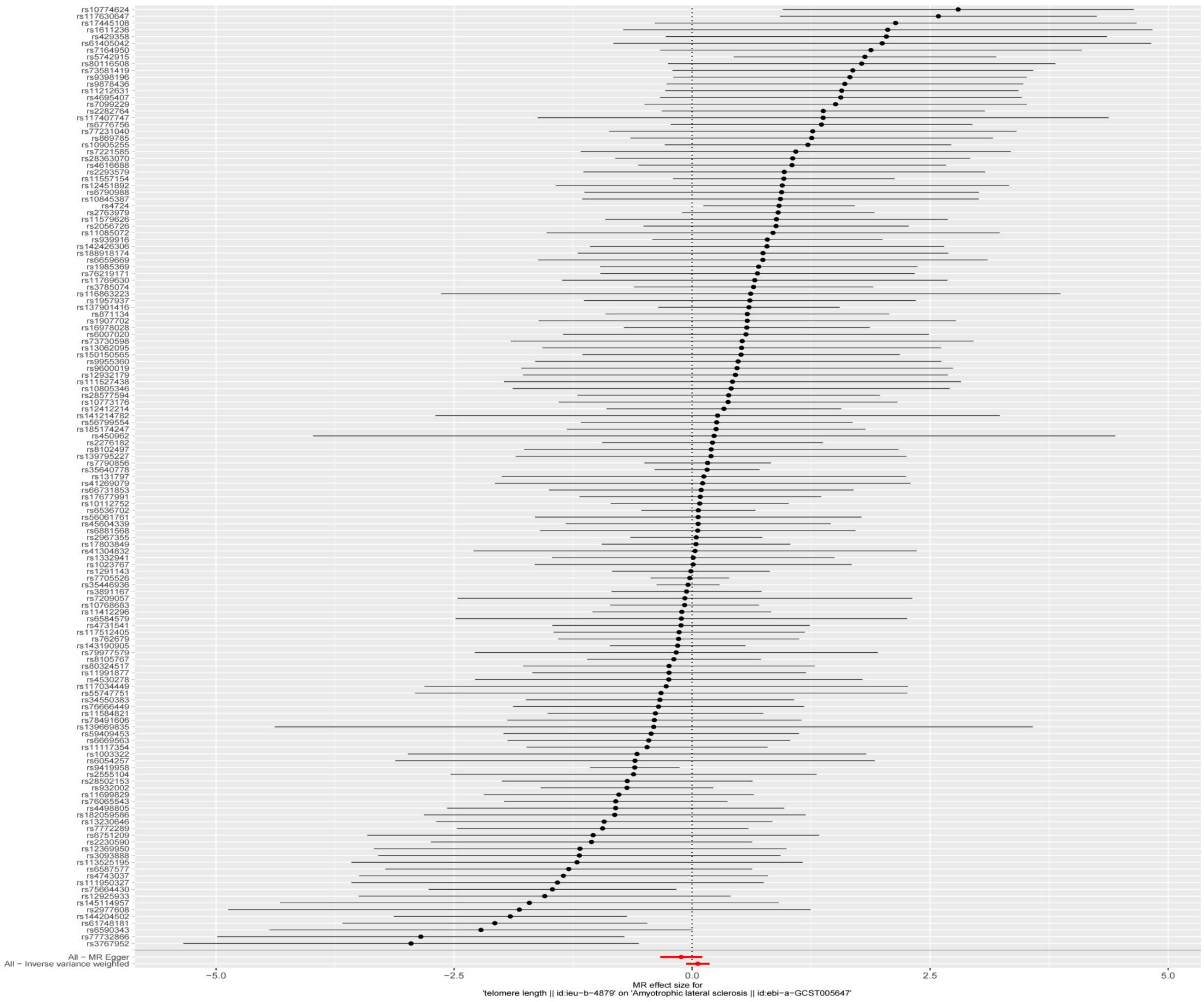

Supplementary Figure-56D Funnel Plot

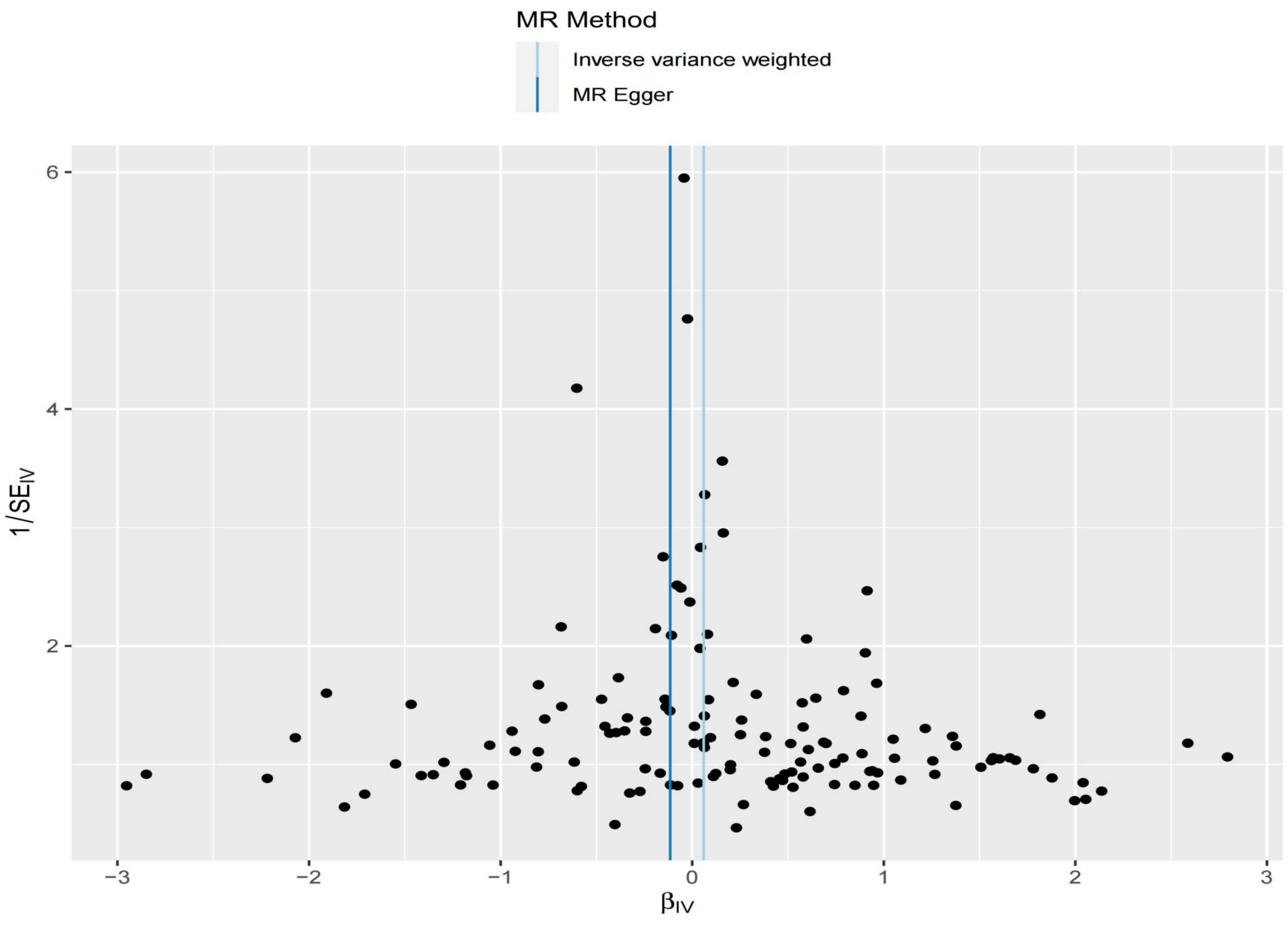

Supplementary Figure-57 Leave-one-out Analysis, Scatter Plot, Forest Plot, and Funnel Plot of Telomere length on GBM

Supplementary Figure-57A Leave-one-out Analysis

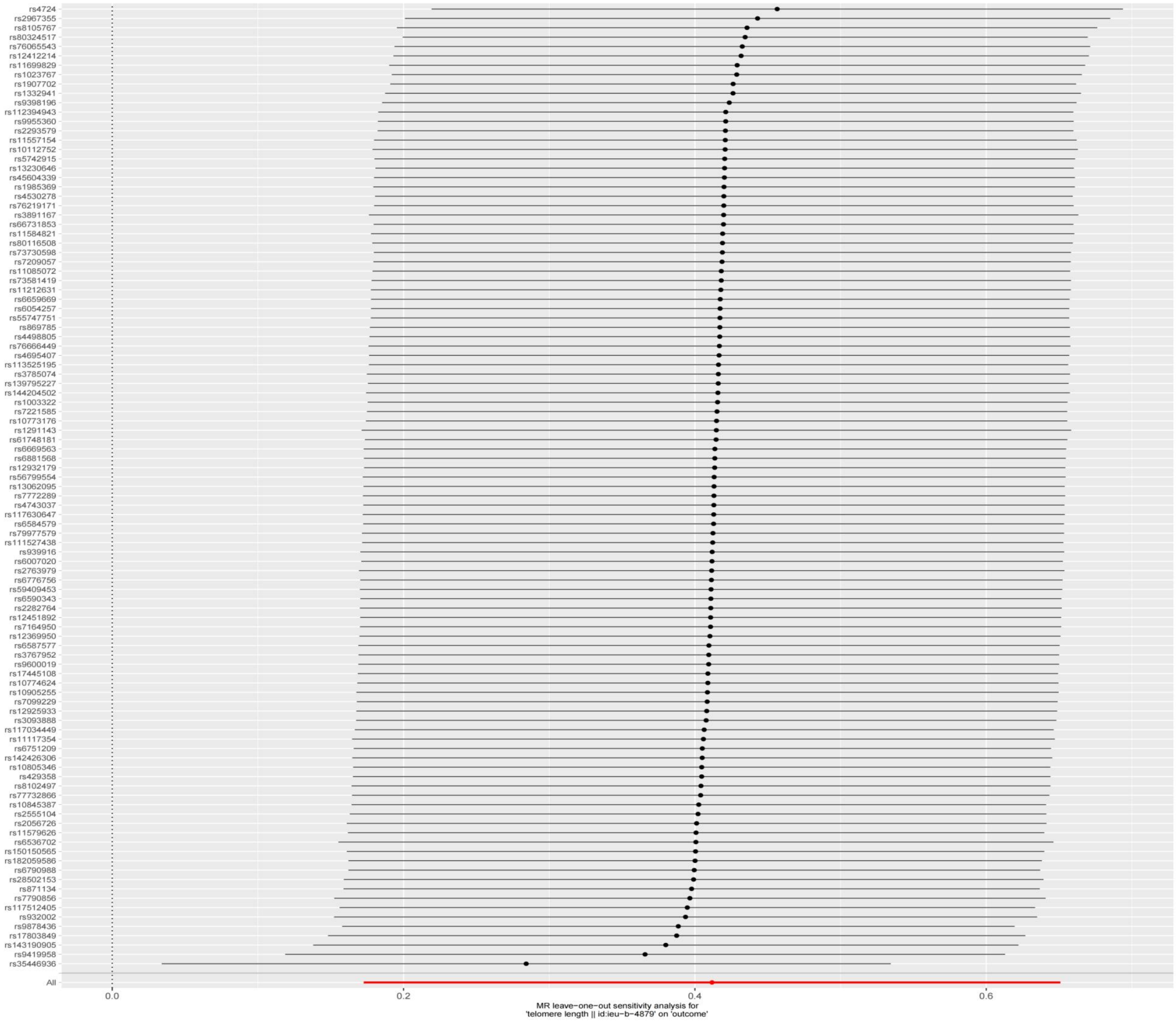

Supplementary Figure-57B Scatter

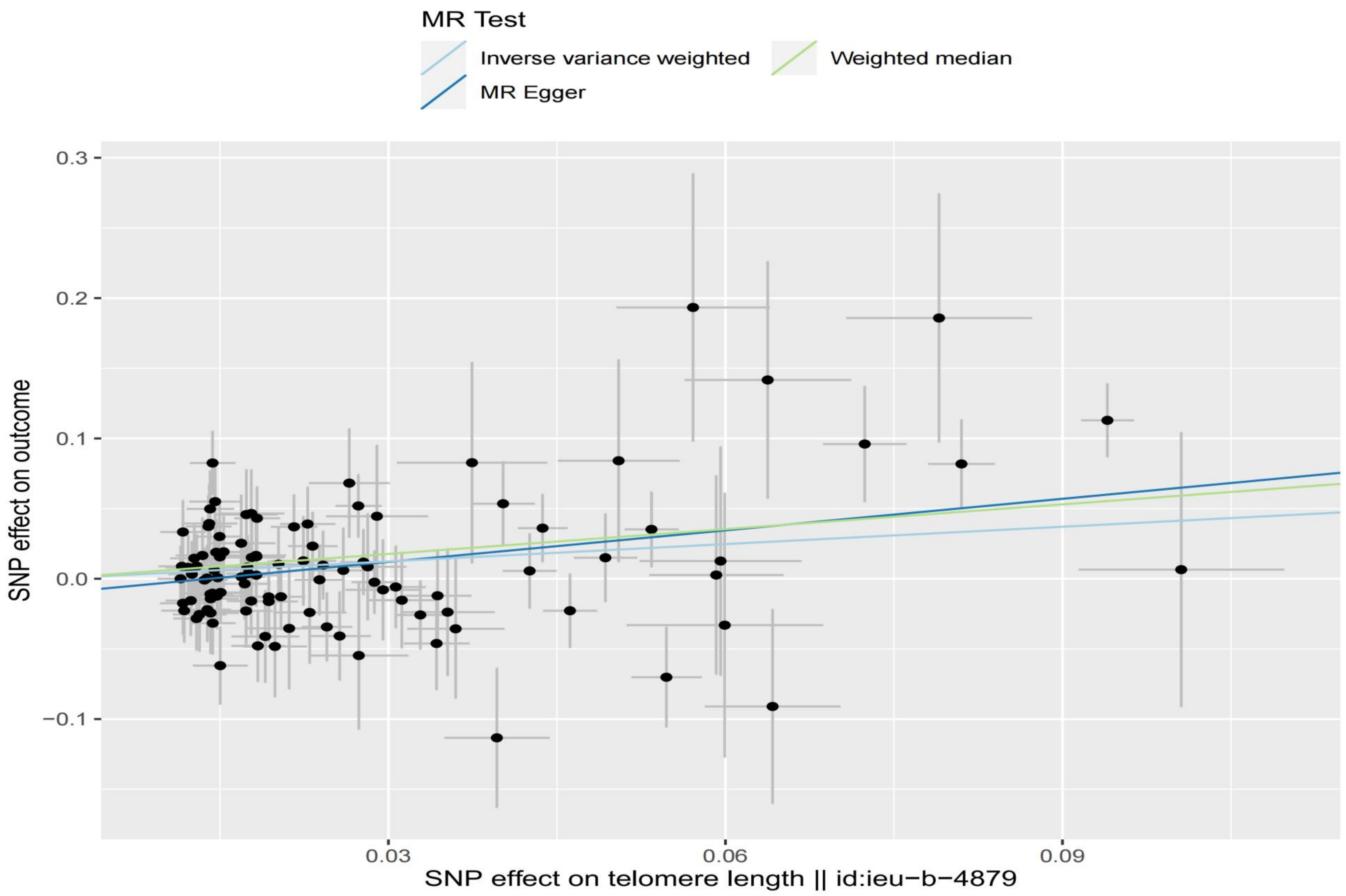

Supplementary Figure-57C Forest Plot

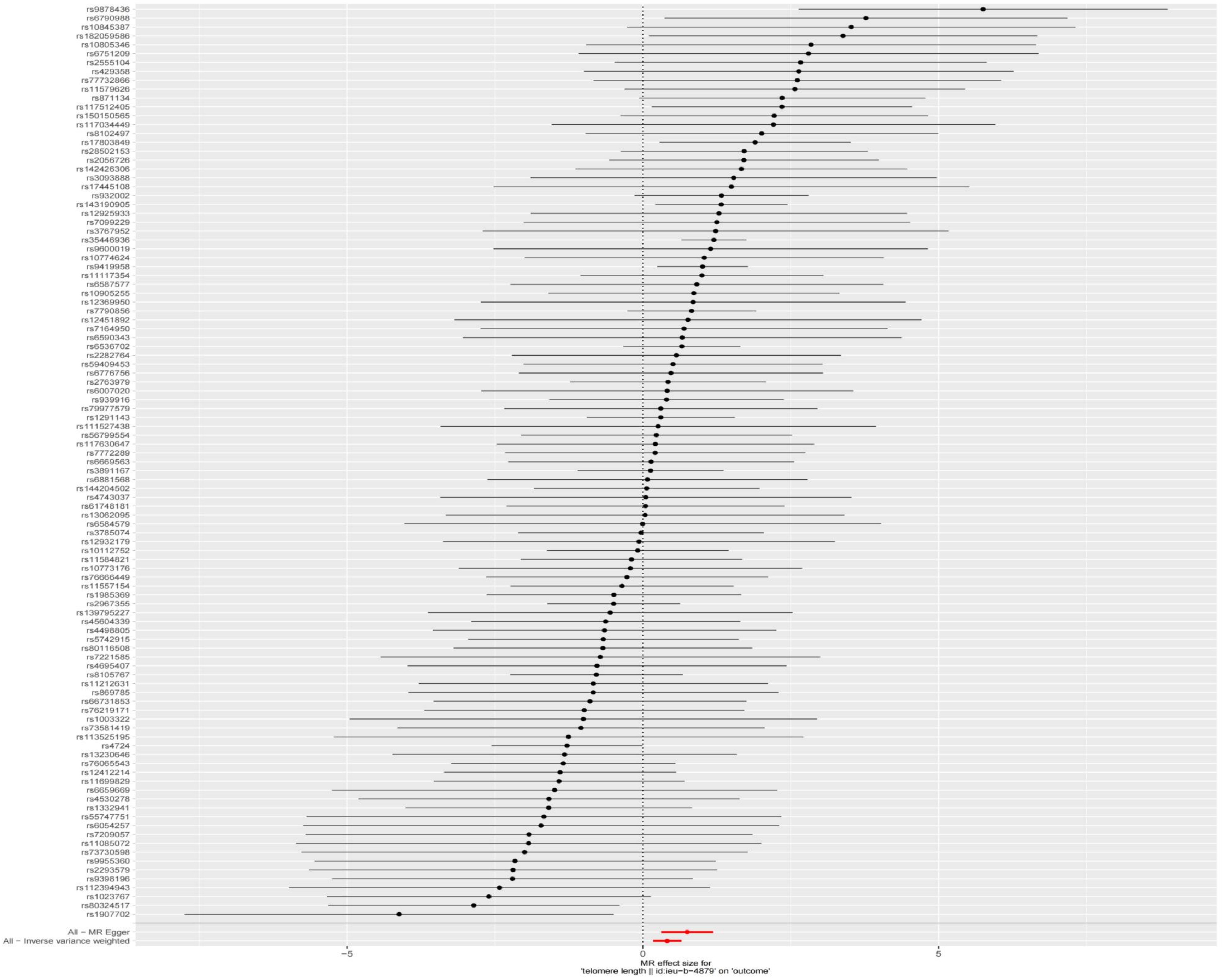

Supplementary Figure-57D Funnel Plot

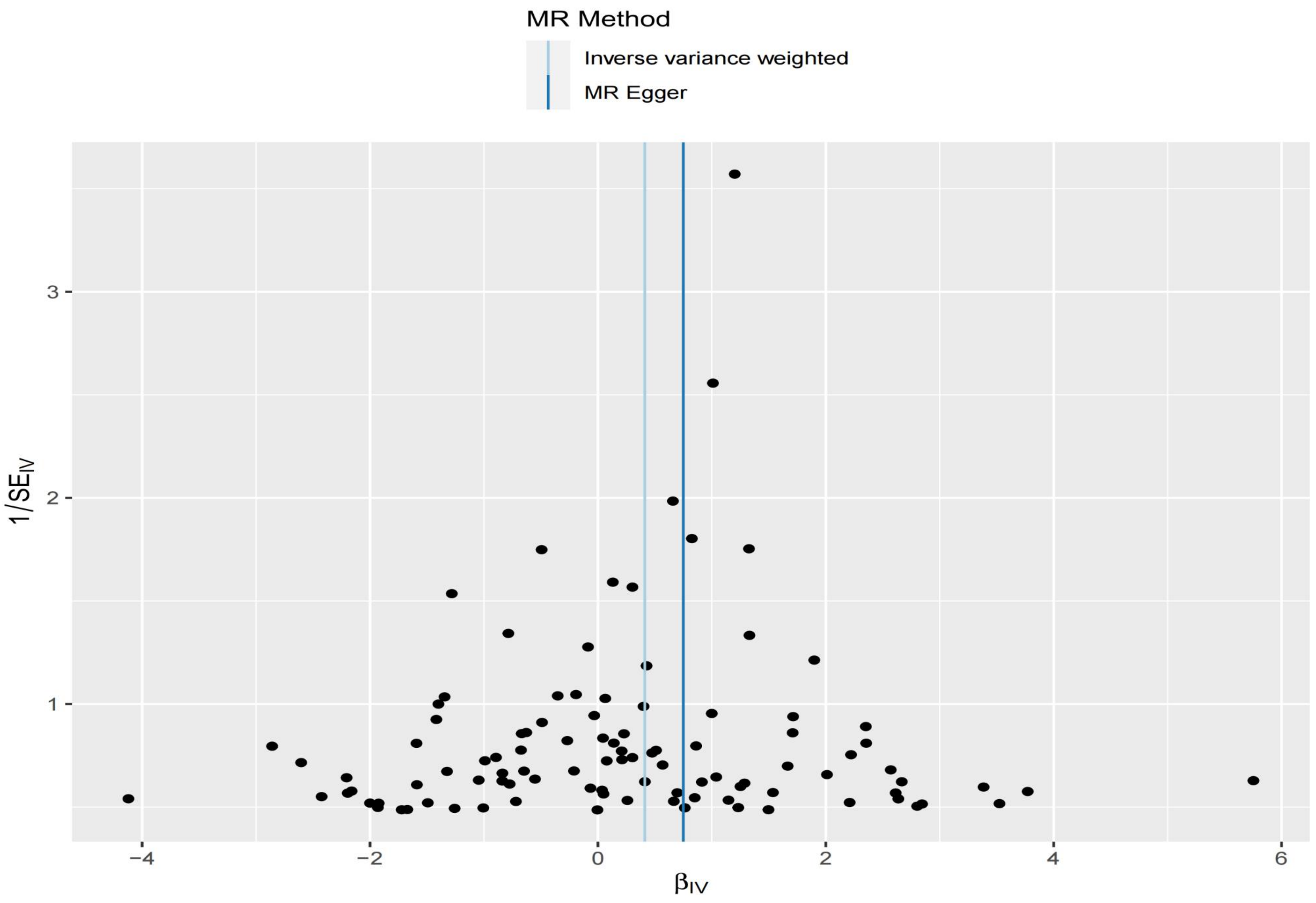

Supplementary Figure-58 Leave-one-out Analysis, Scatter Plot, Forest Plot, and Funnel Plot of Telomere length on Meningioma

Supplementary Figure-58A Leave-one-out Analysis

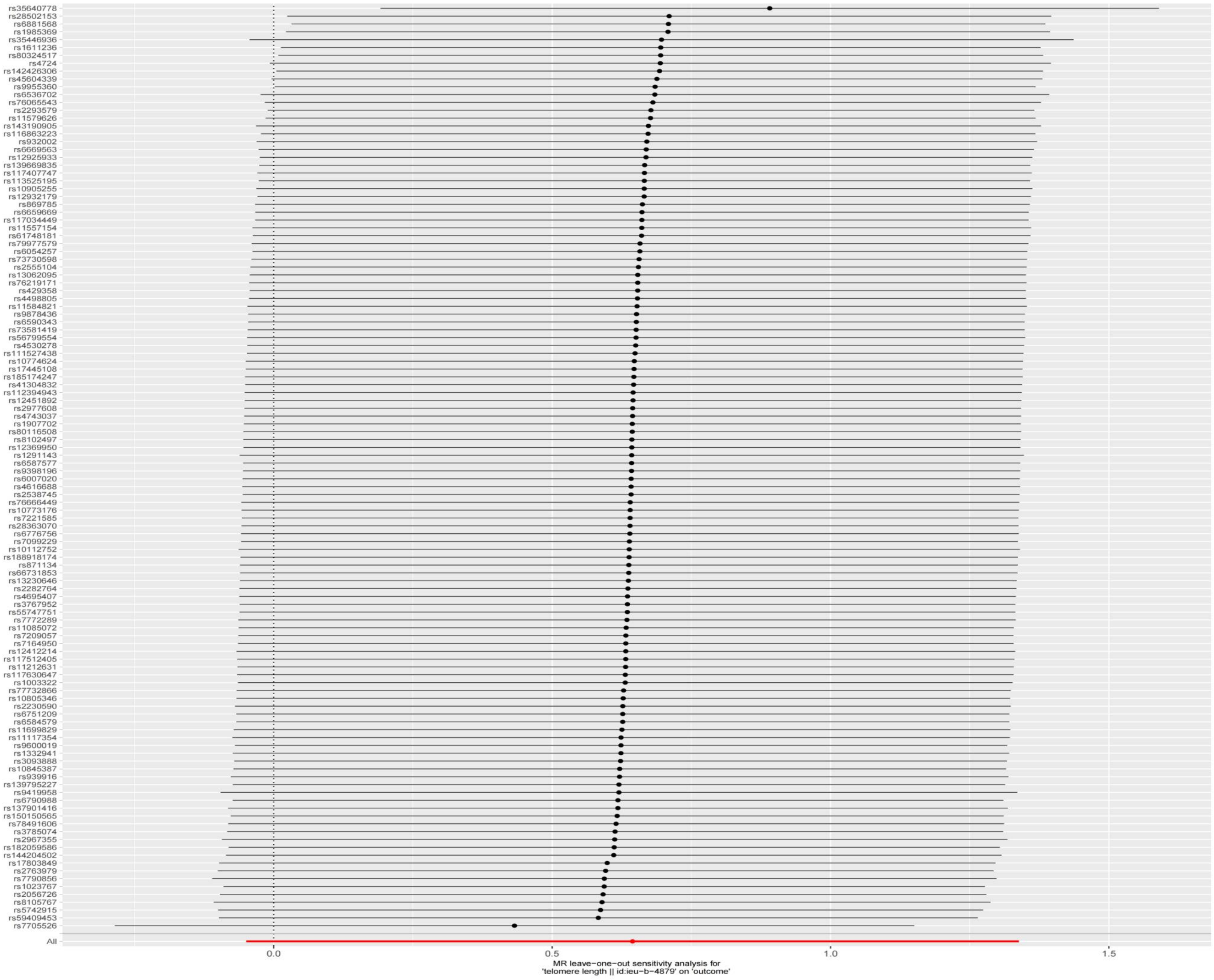

Supplementary Figure-58B Scatter

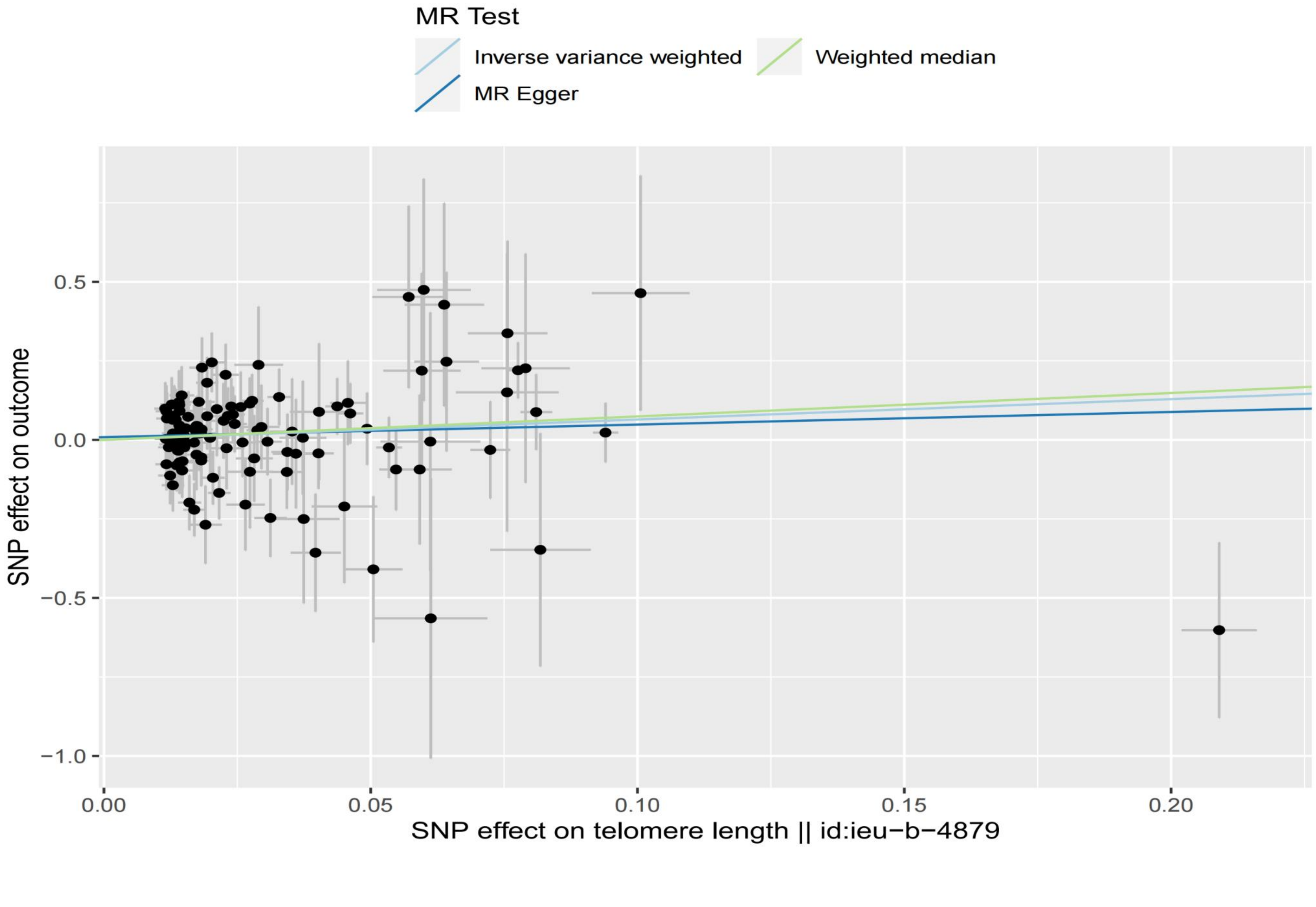

Supplementary Figure-58C Forest Plot

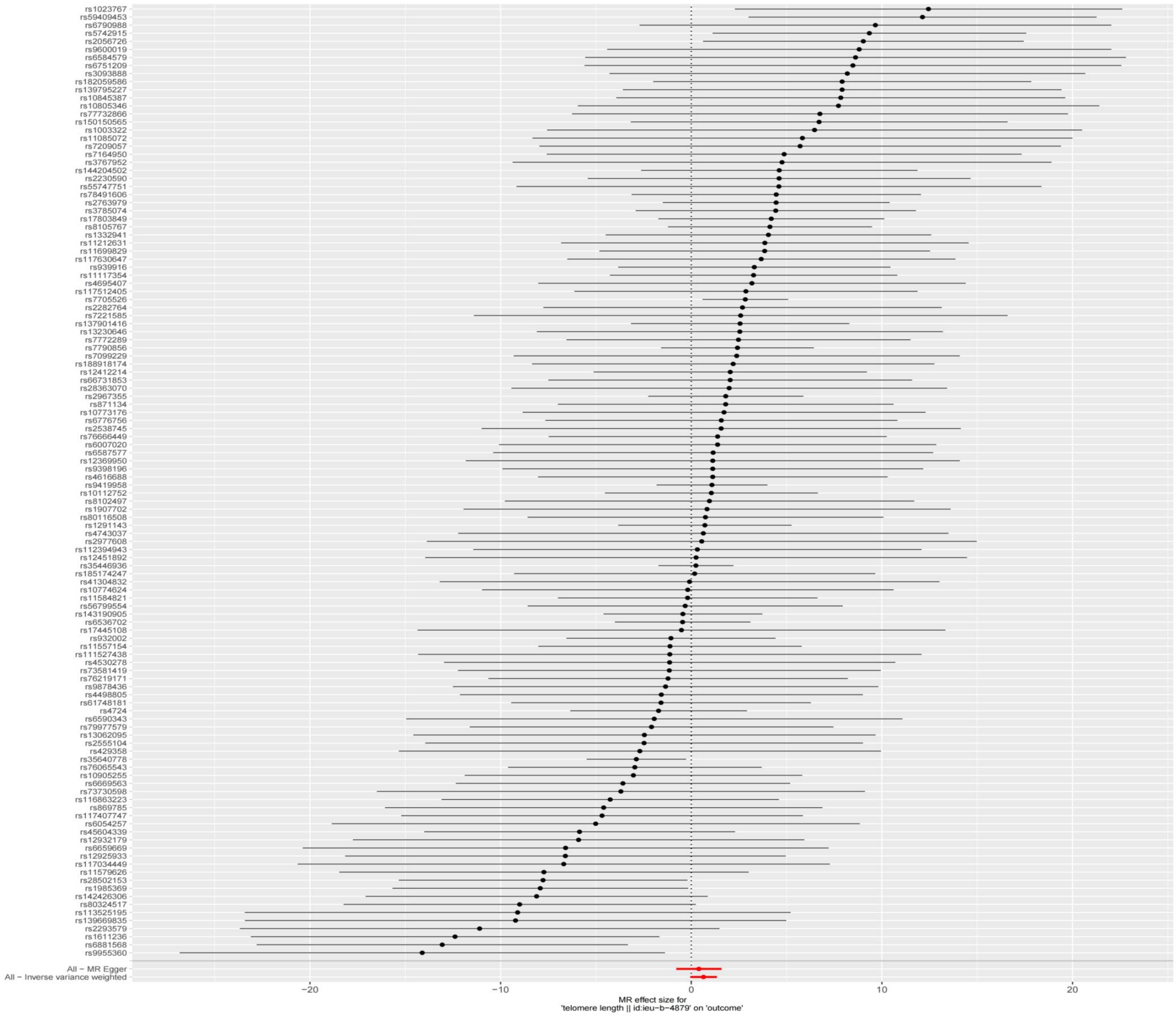

Supplementary Figure-58D Funnel Plot

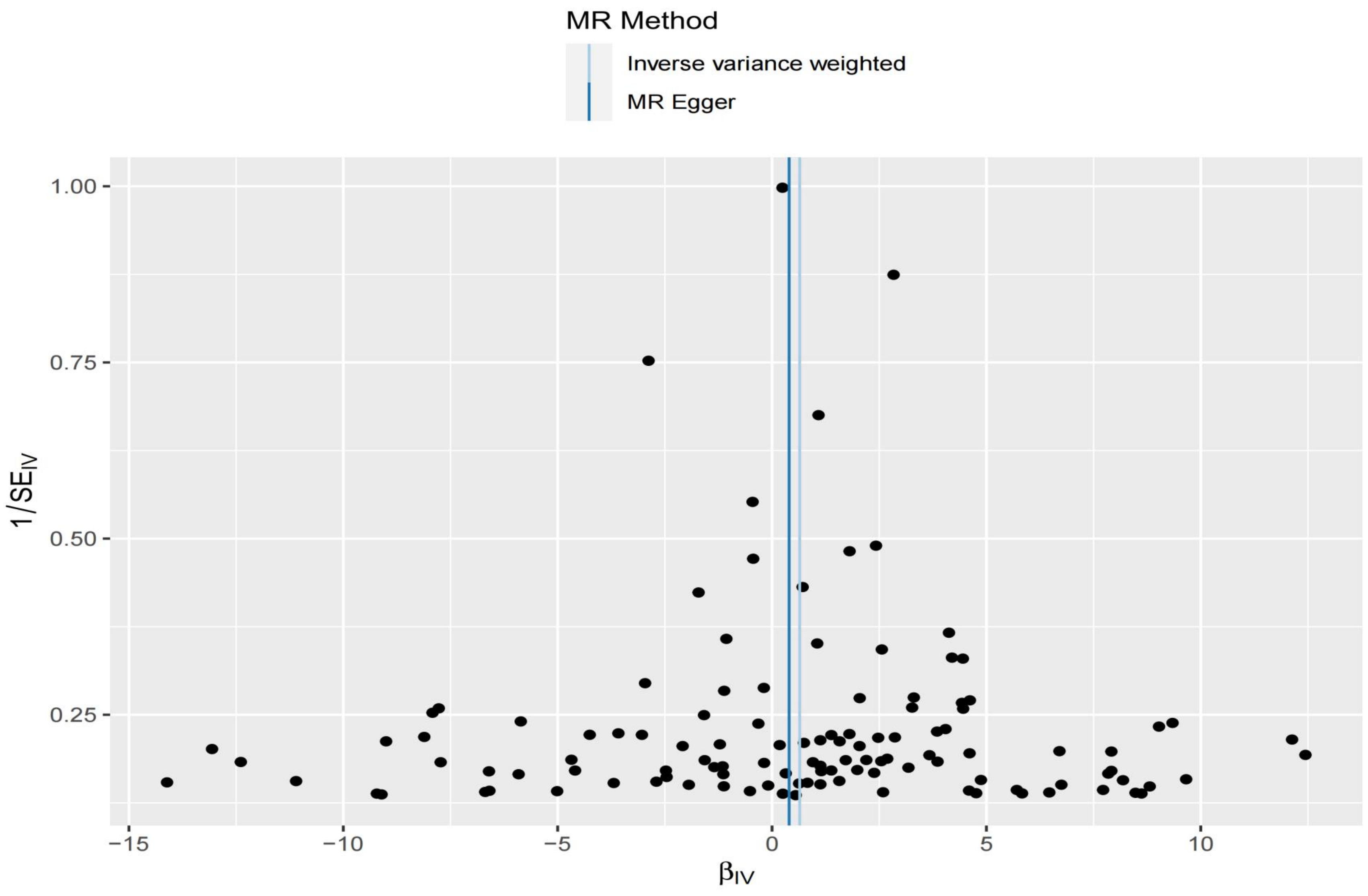

Supplementary Figure-59 Leave-one-out Analysis, Scatter Plot, Forest Plot, and Funnel Plot of Telomere length on Non GBM

Supplementary Figure-59A Leave-one-out Analysis

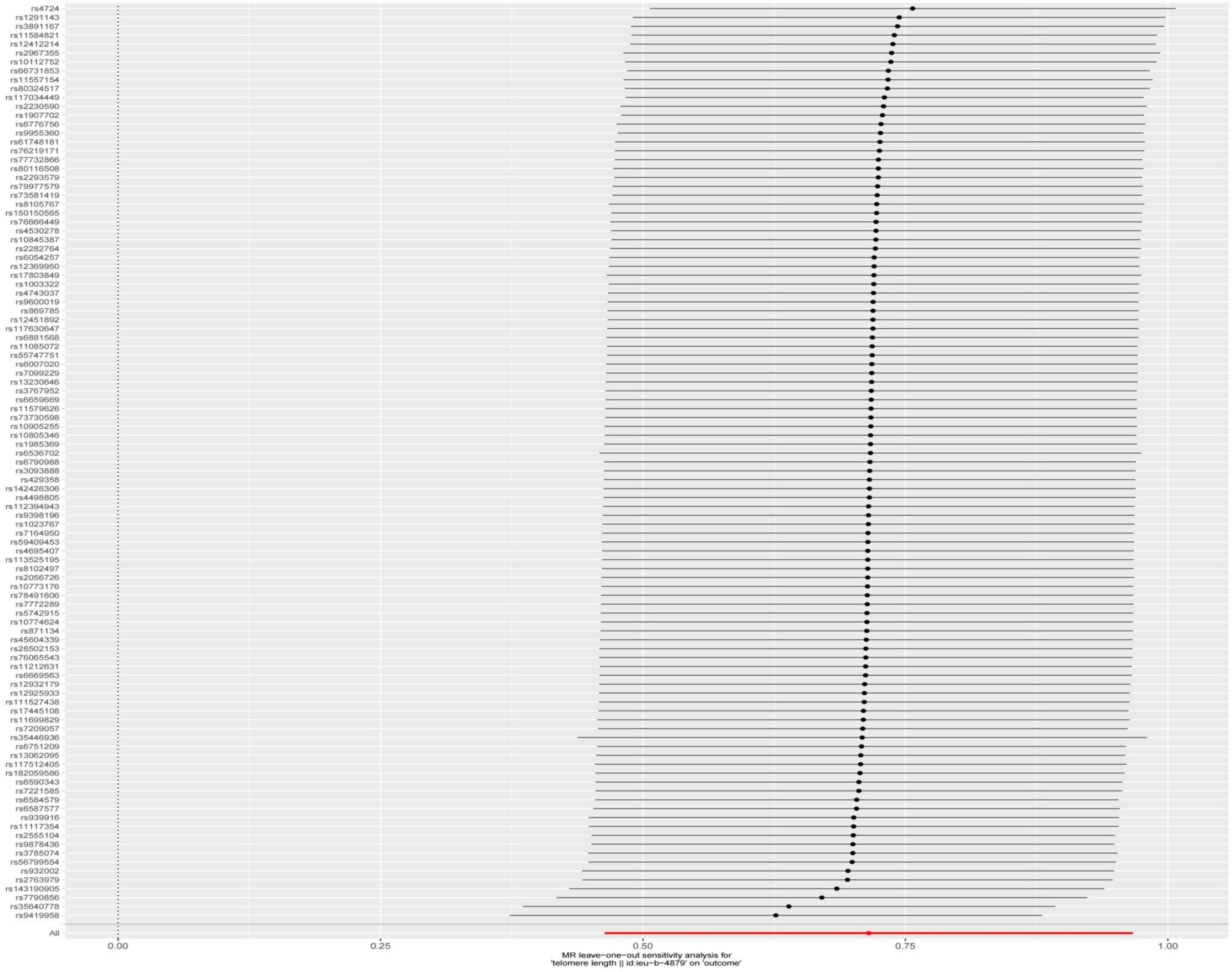

Supplementary Figure-59B Scatter

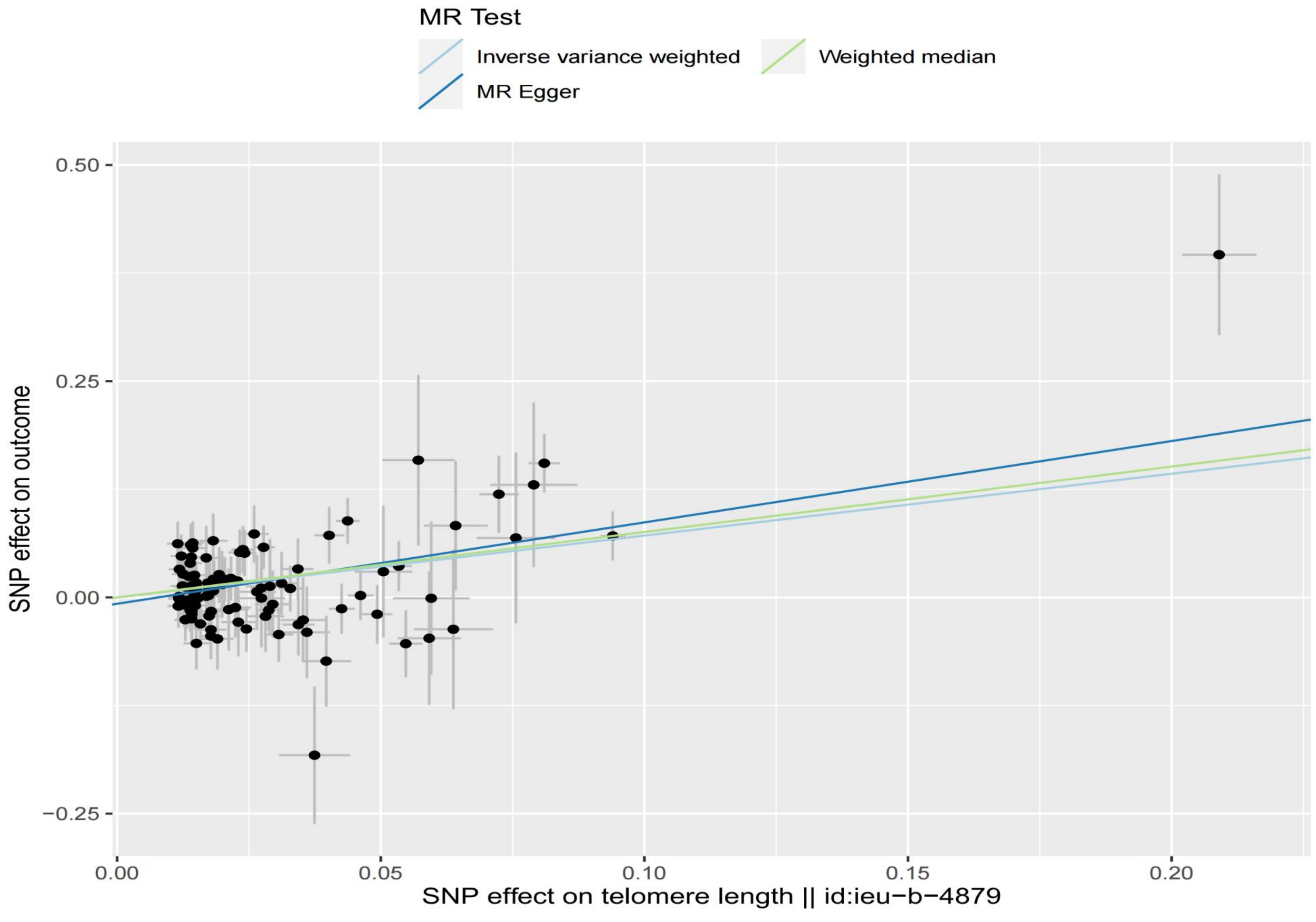

Supplementary Figure-59C Forest Plot

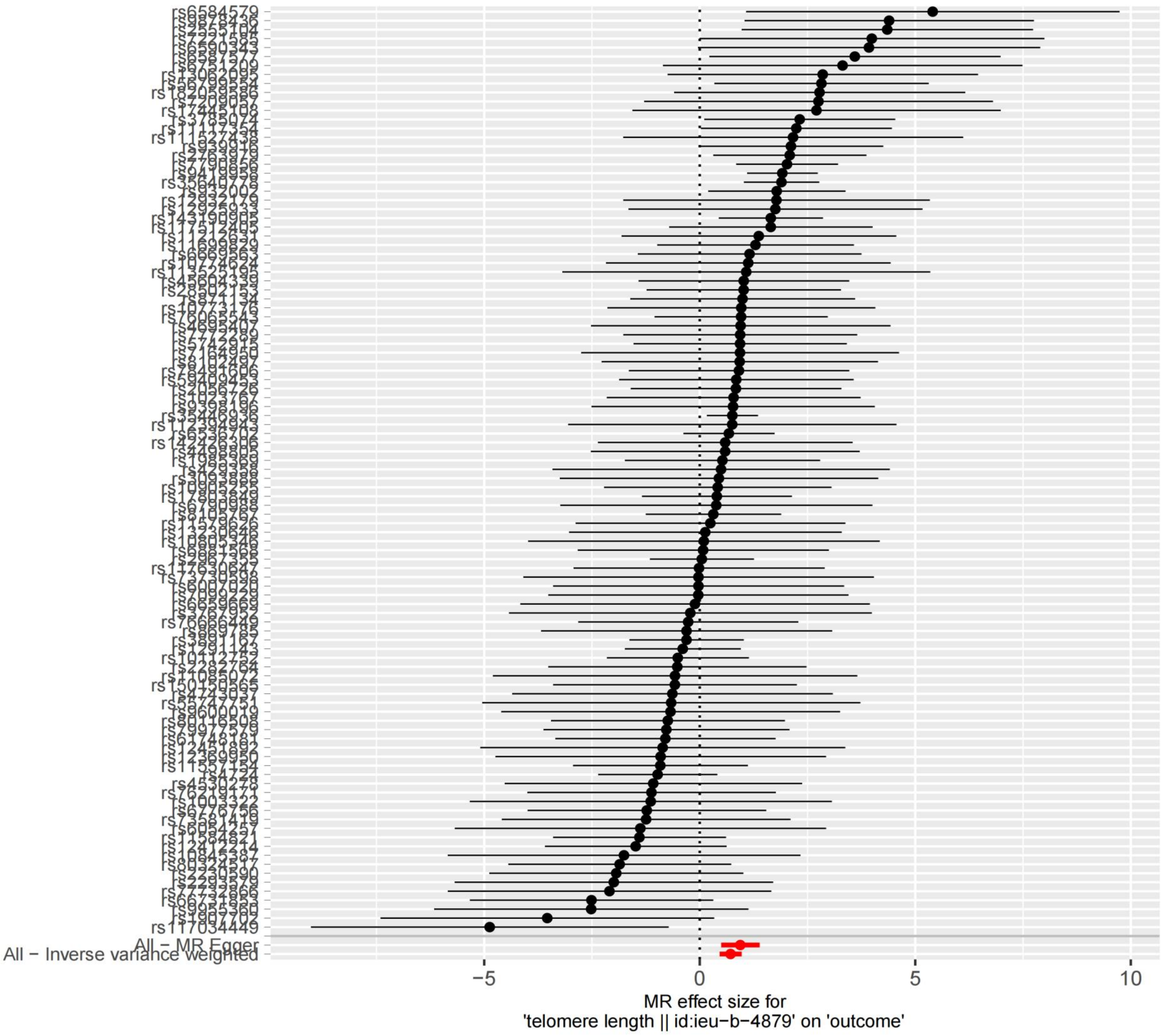

Supplementary Figure-59D Funnel Plot

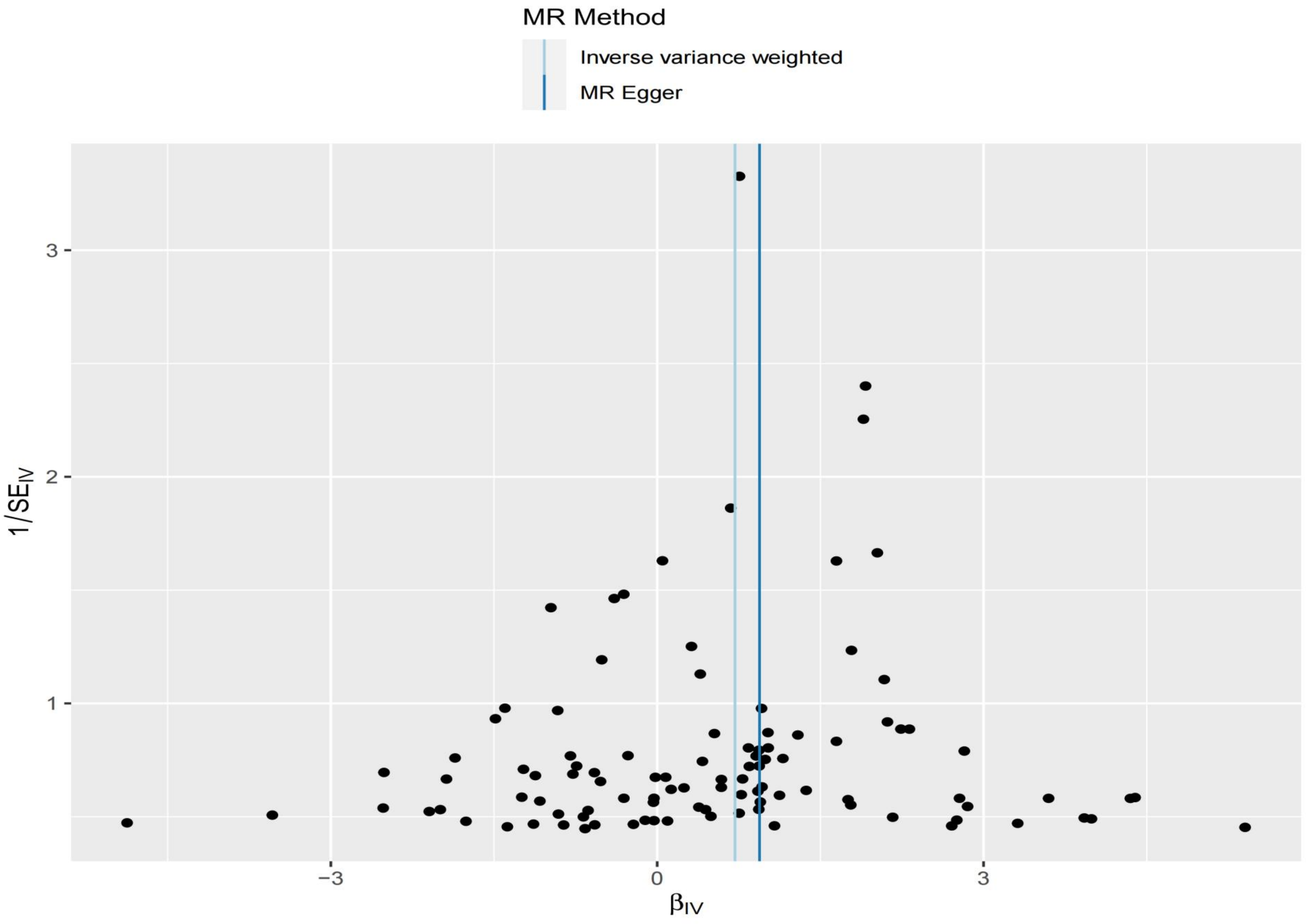

Supplementary Figure-60 Leave-one-out Analysis, Scatter Plot, Forest Plot, and Funnel Plot of Telomere length on Parkinson's Disease

Supplementary Figure-60A Leave-one-out Analysis

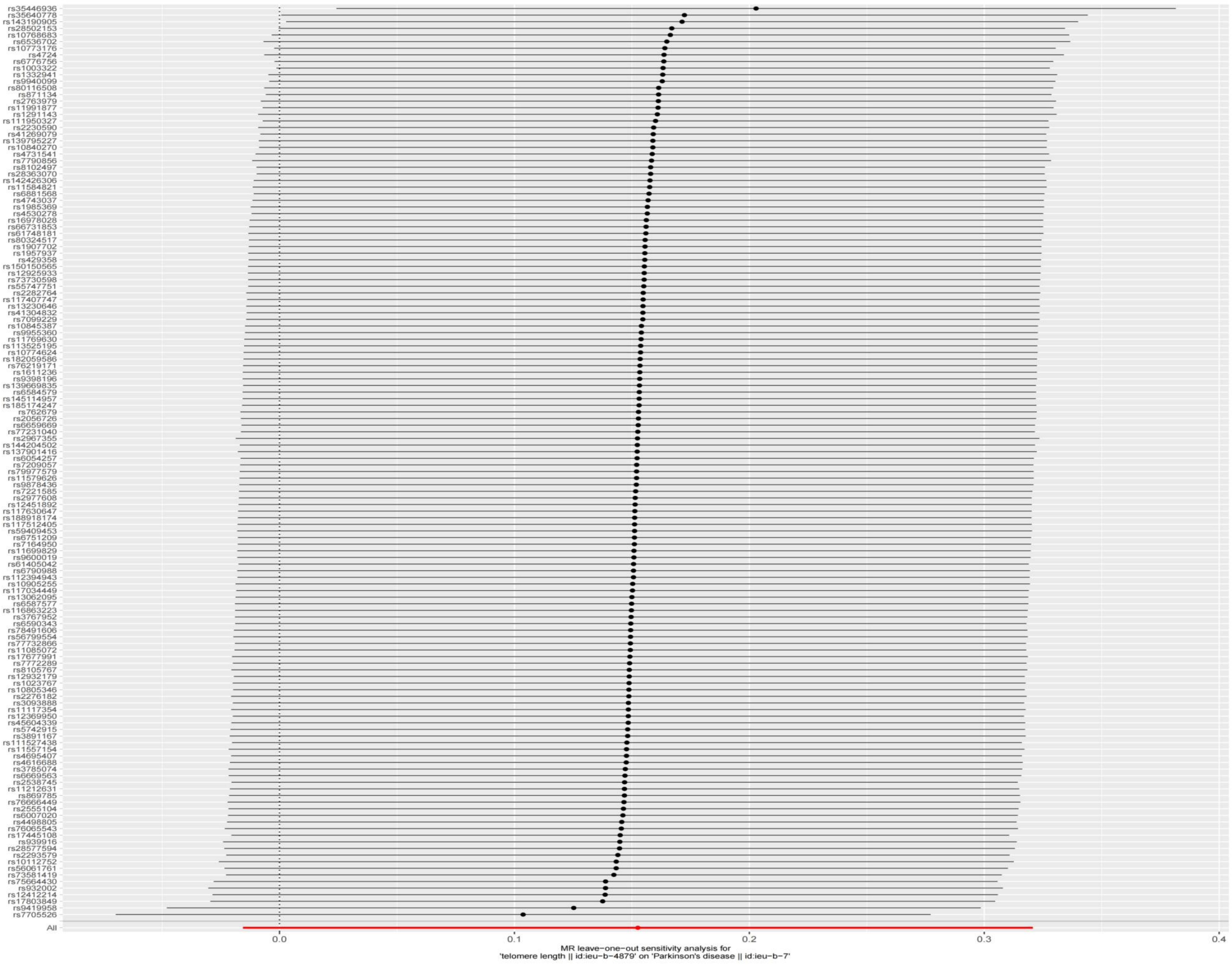

Supplementary Figure-60B Scatter

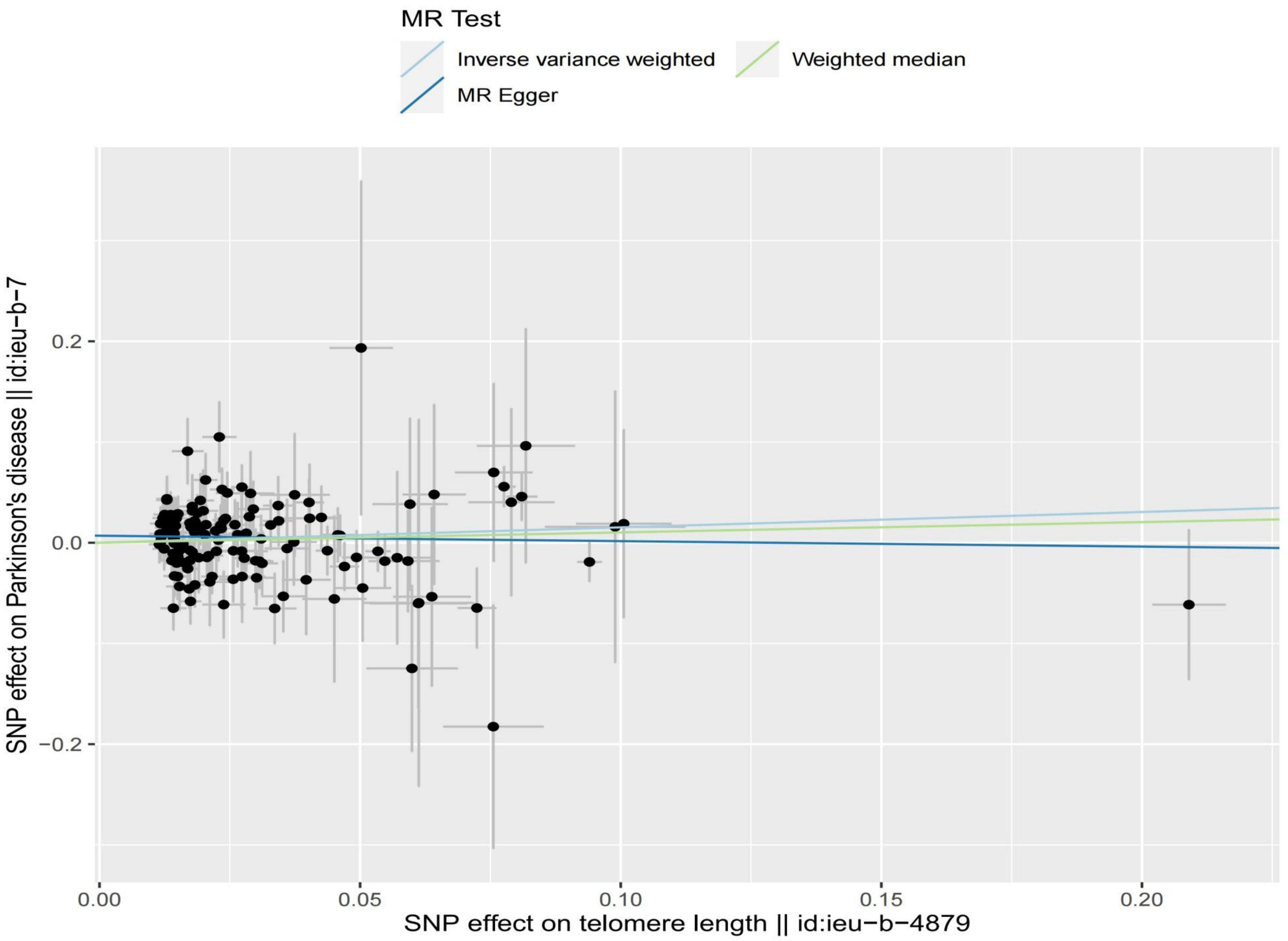

Supplementary Figure-60C Forest Plot

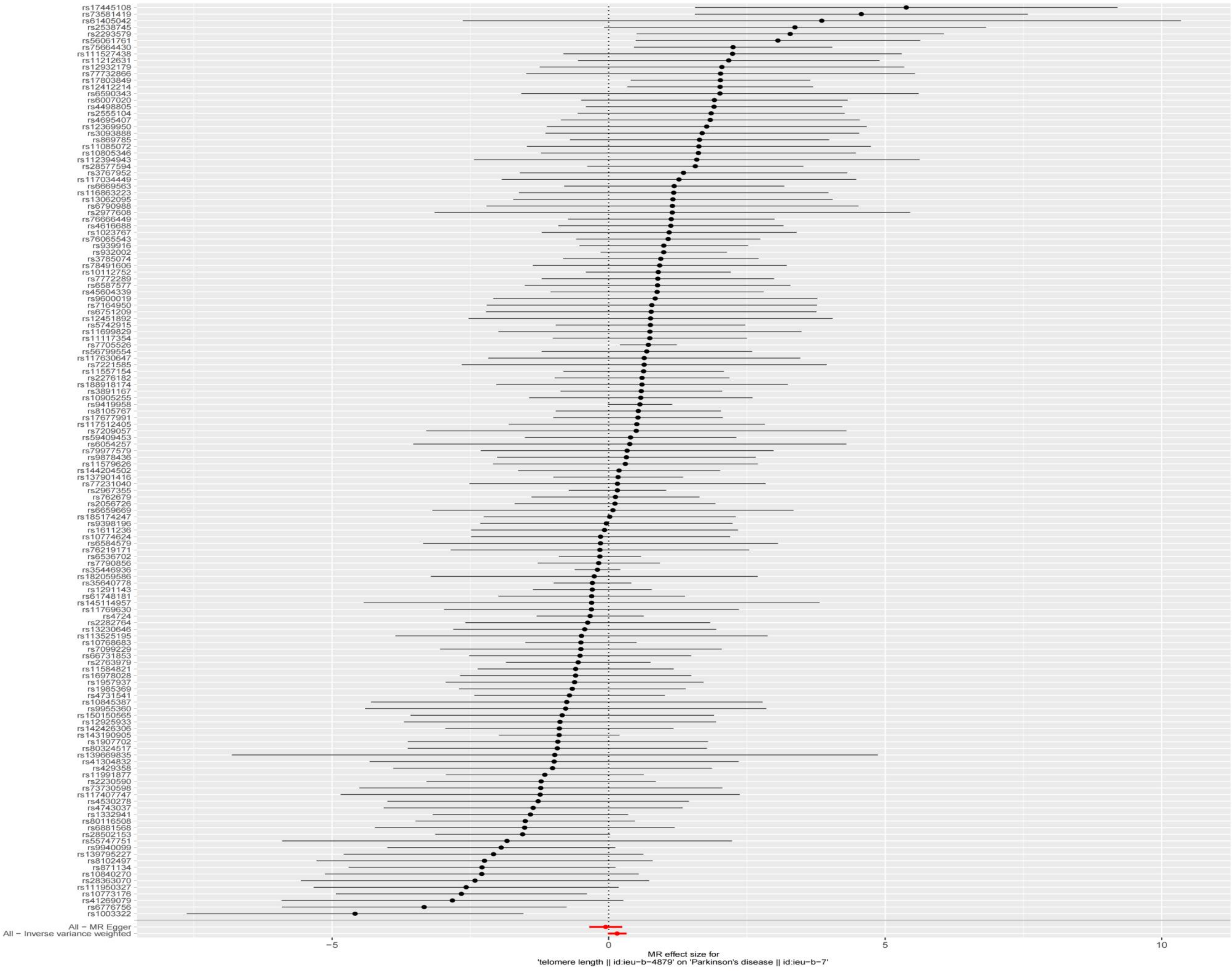

Supplementary Figure-60D Funnel Plot

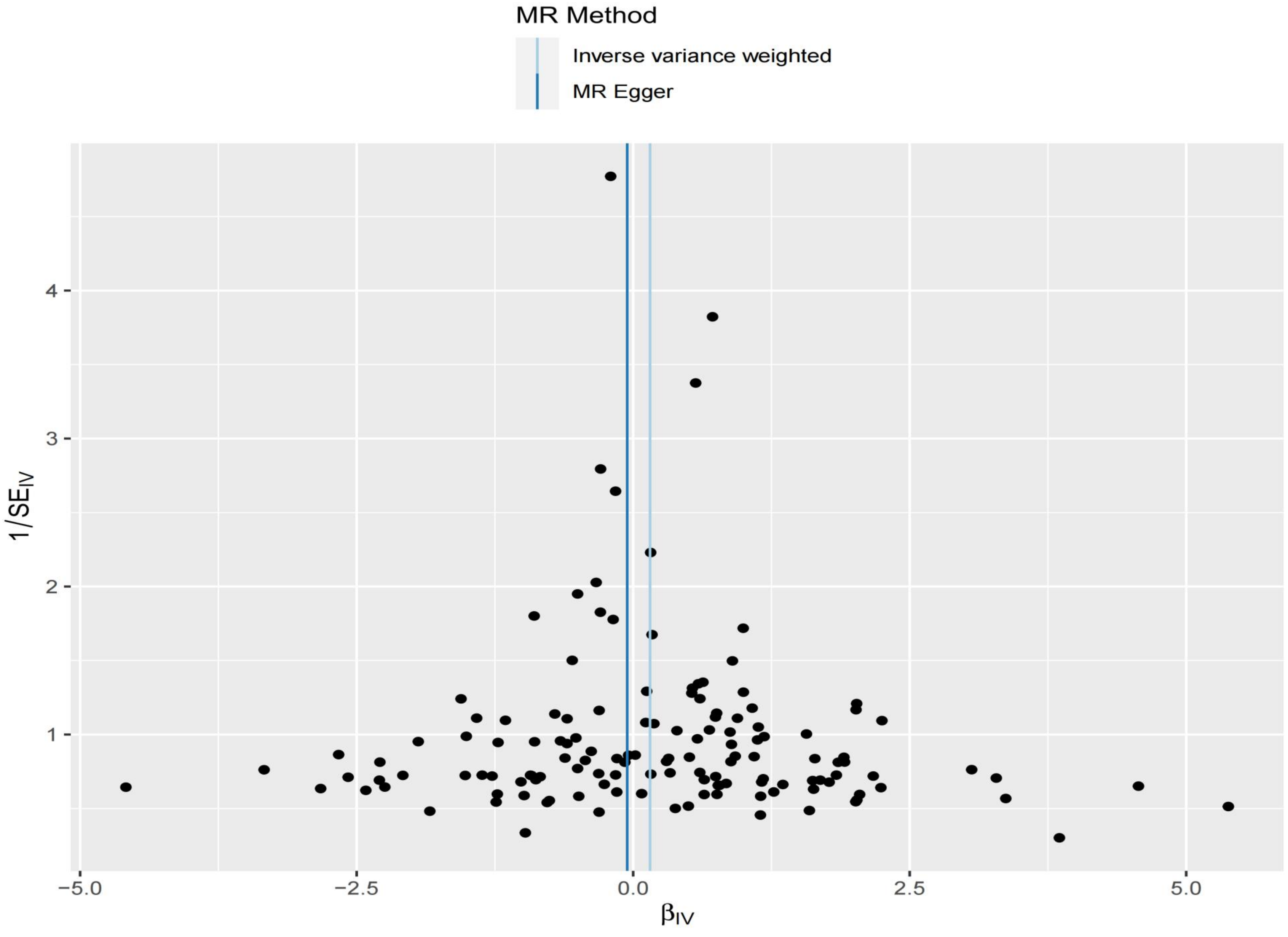

Supplementary Figure-61 Leave-one-out Analysis, Scatter Plot, Forest Plot, and Funnel Plot of Telomere length on Vestibular Schwannomas

Supplementary Figure-61A Leave-one-out Analysis

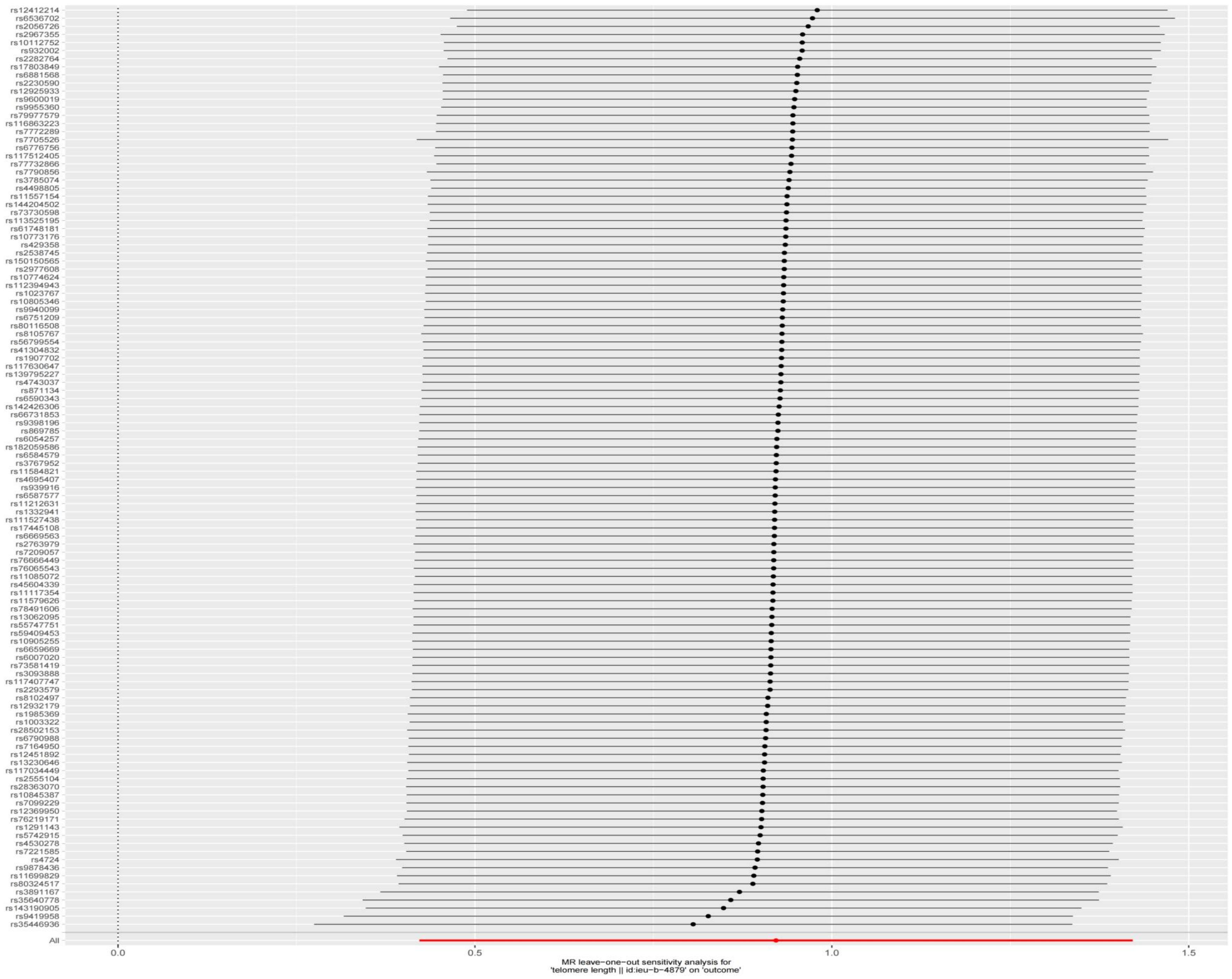

Supplementary Figure-61B Scatter

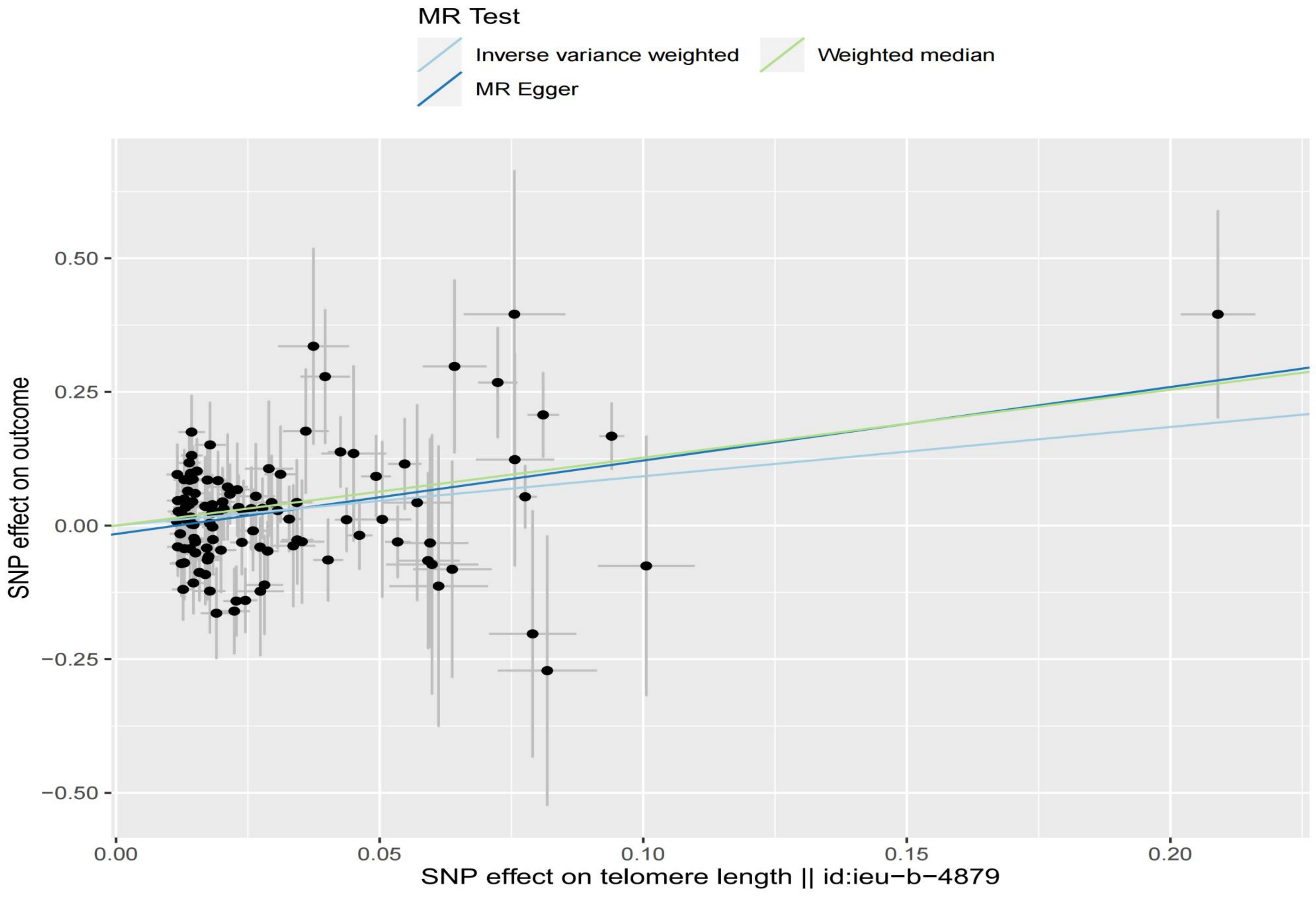

Supplementary Figure-61C Forest Plot

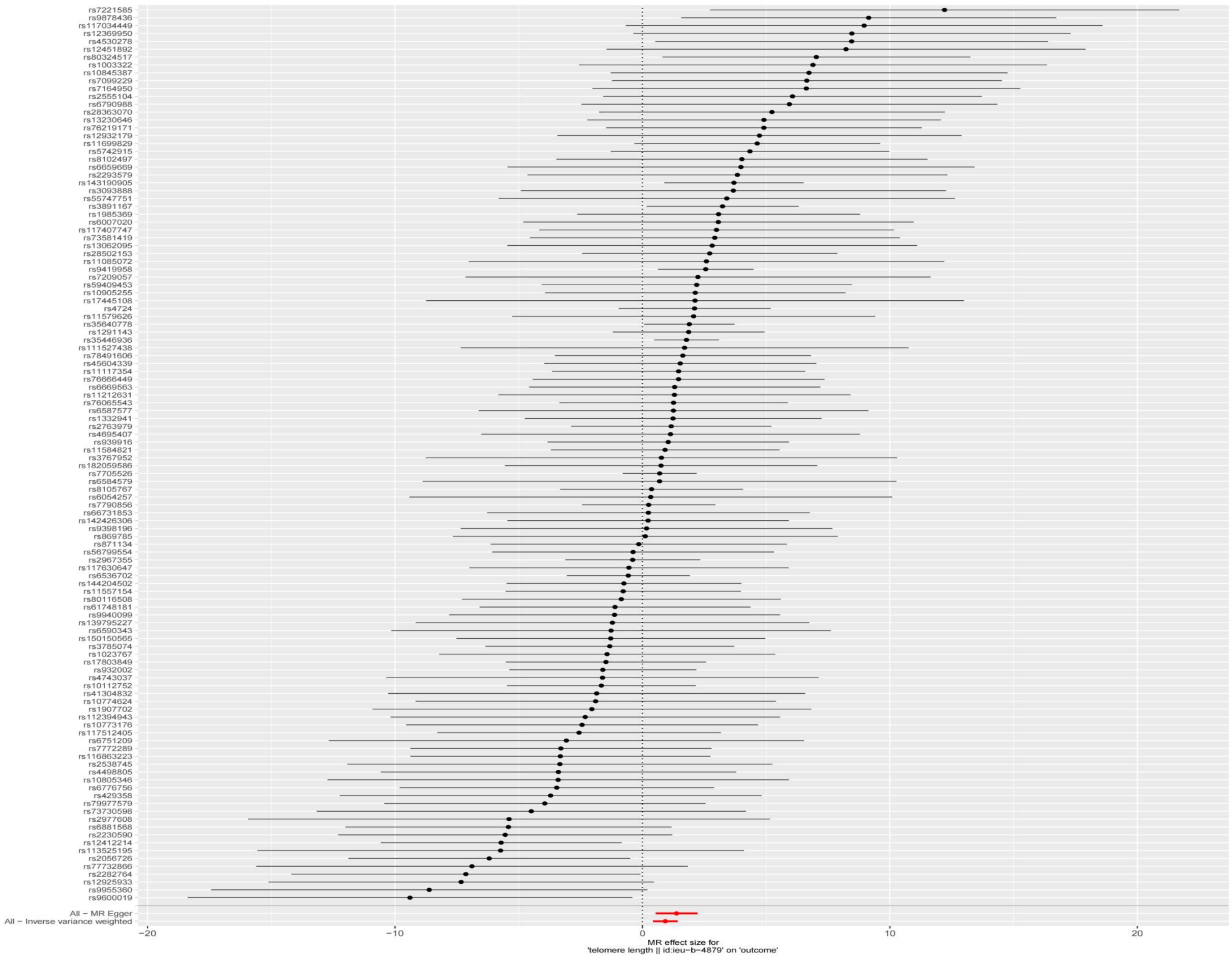

Supplementary Figure-61D Funnel Plot

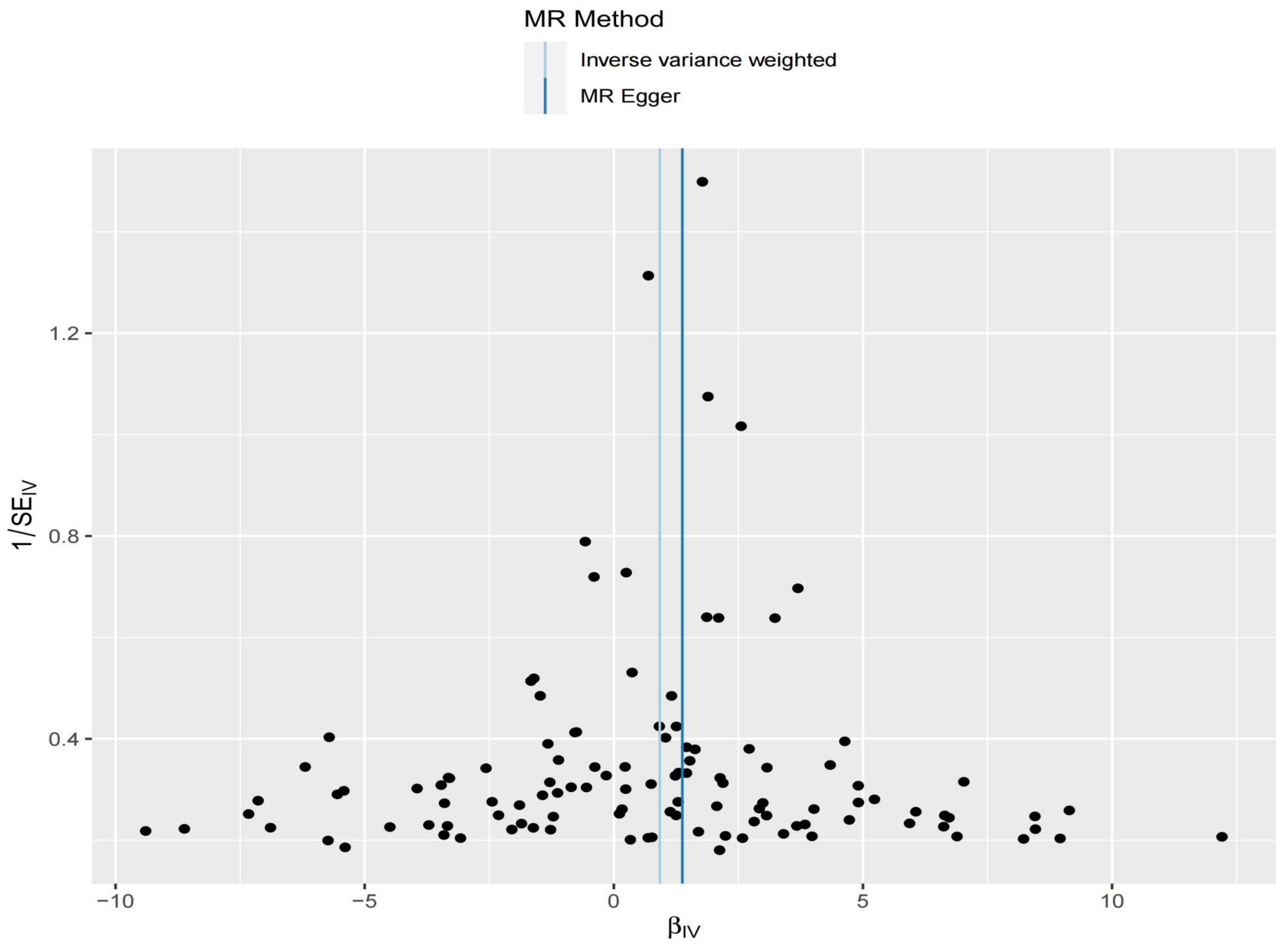

Supplementary Figure-62 Leave-one-out Analysis, Scatter Plot, Forest Plot, and Funnel Plot of Vestibular Schwannomas on Facial Ageing

Supplementary Figure-62A Leave-one-out Analysis

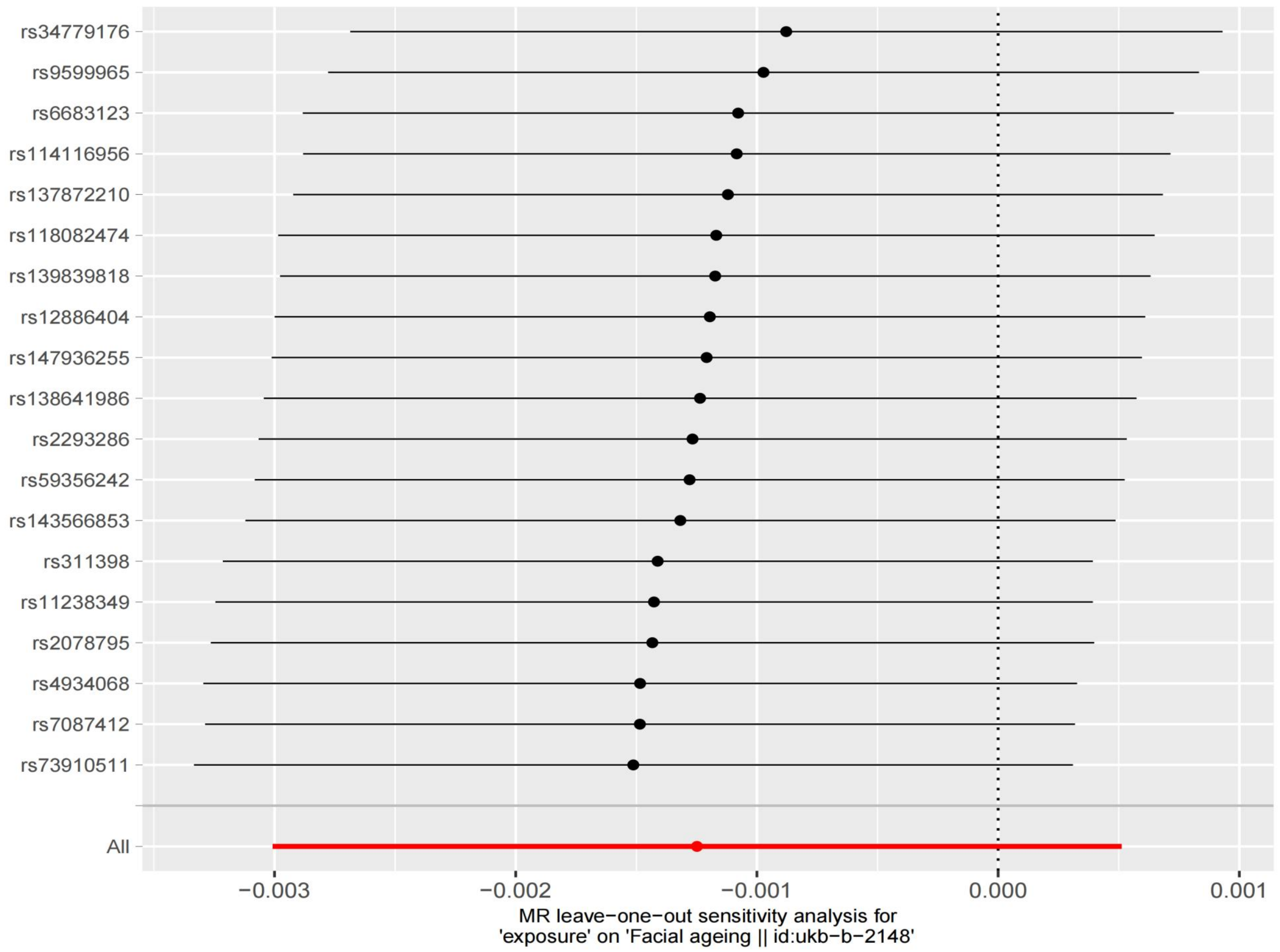

Supplementary Figure-62B Scatter

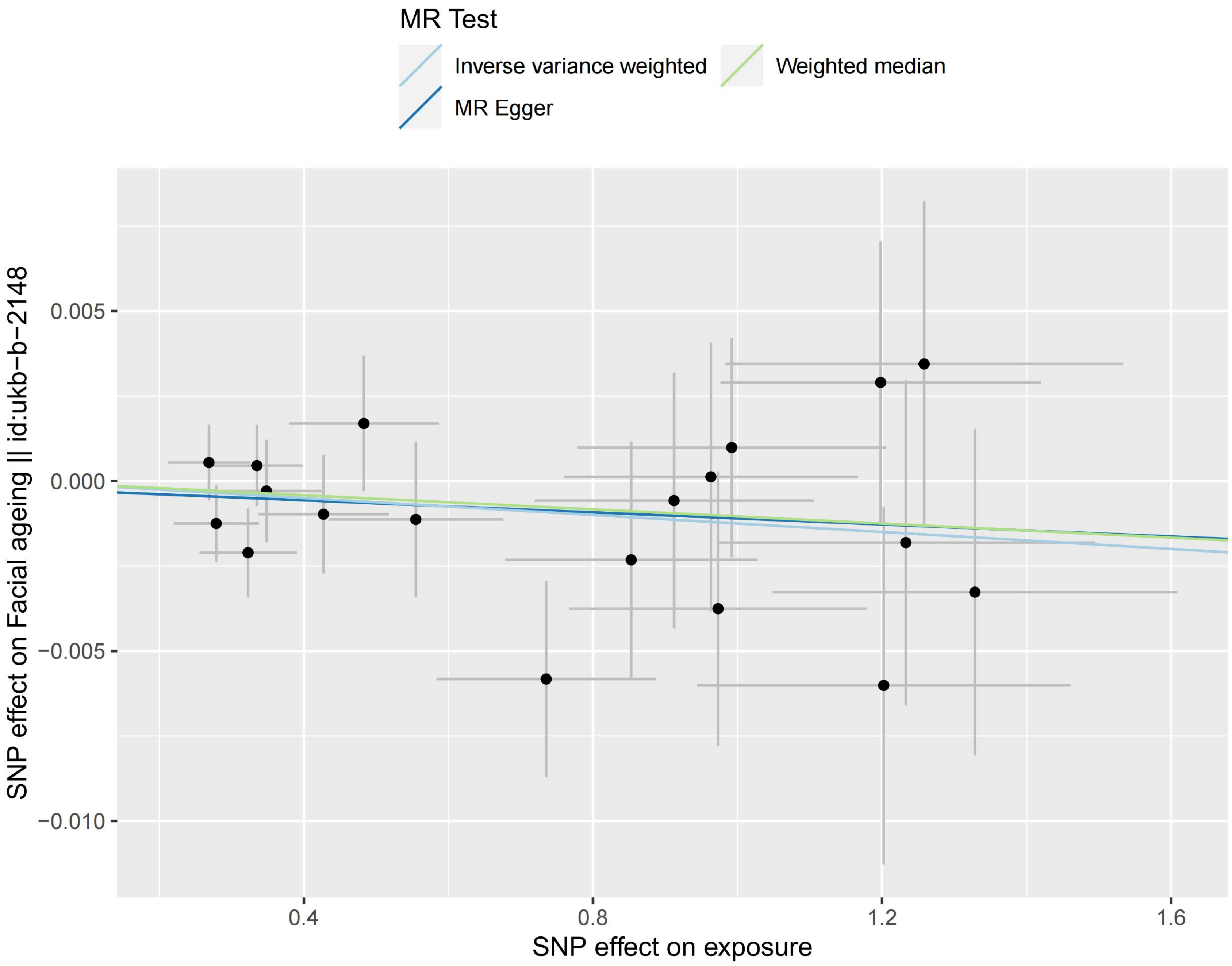

Supplementary Figure-62C Forest Plot

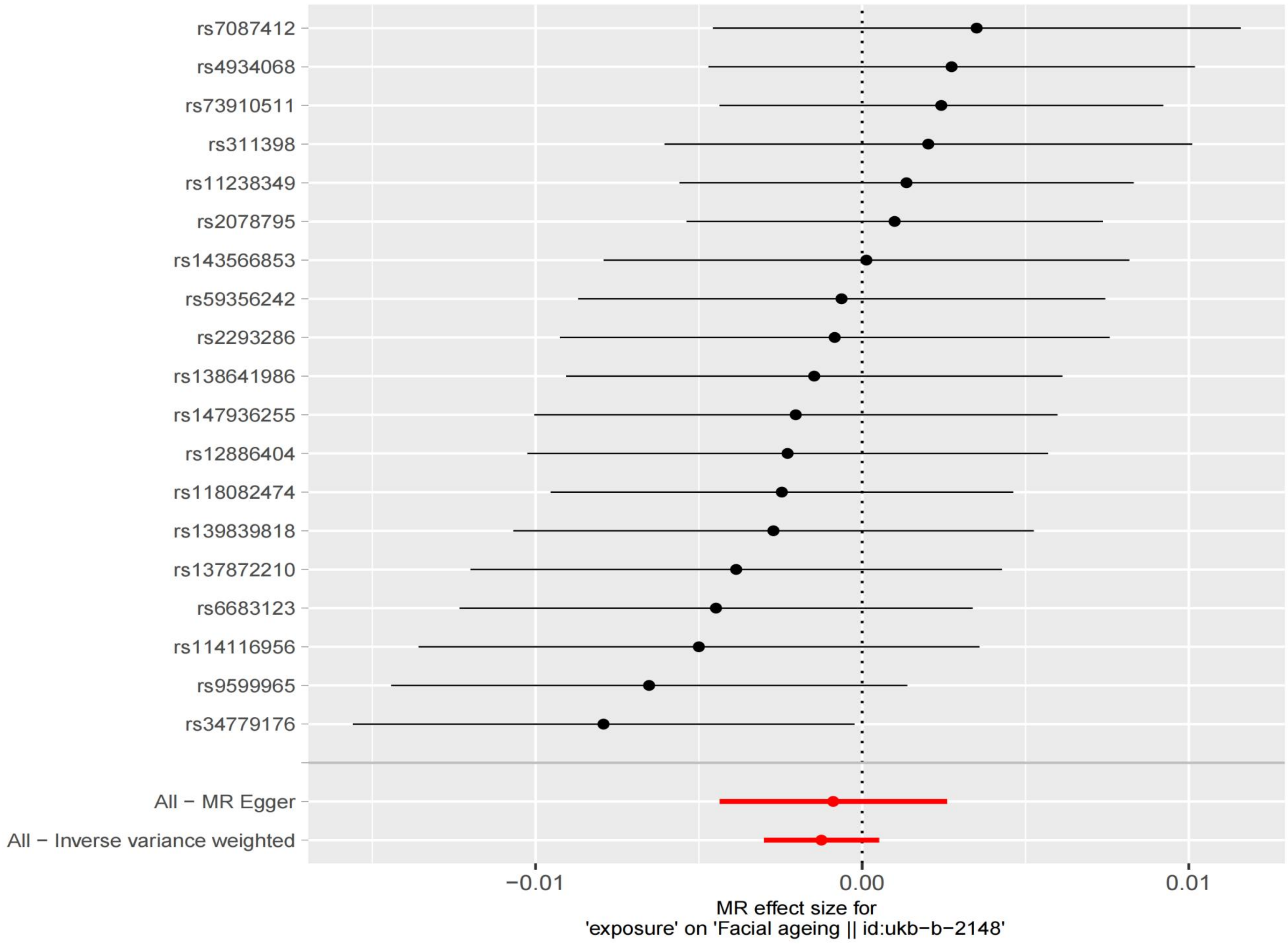

Supplementary Figure-62D Funnel Plot

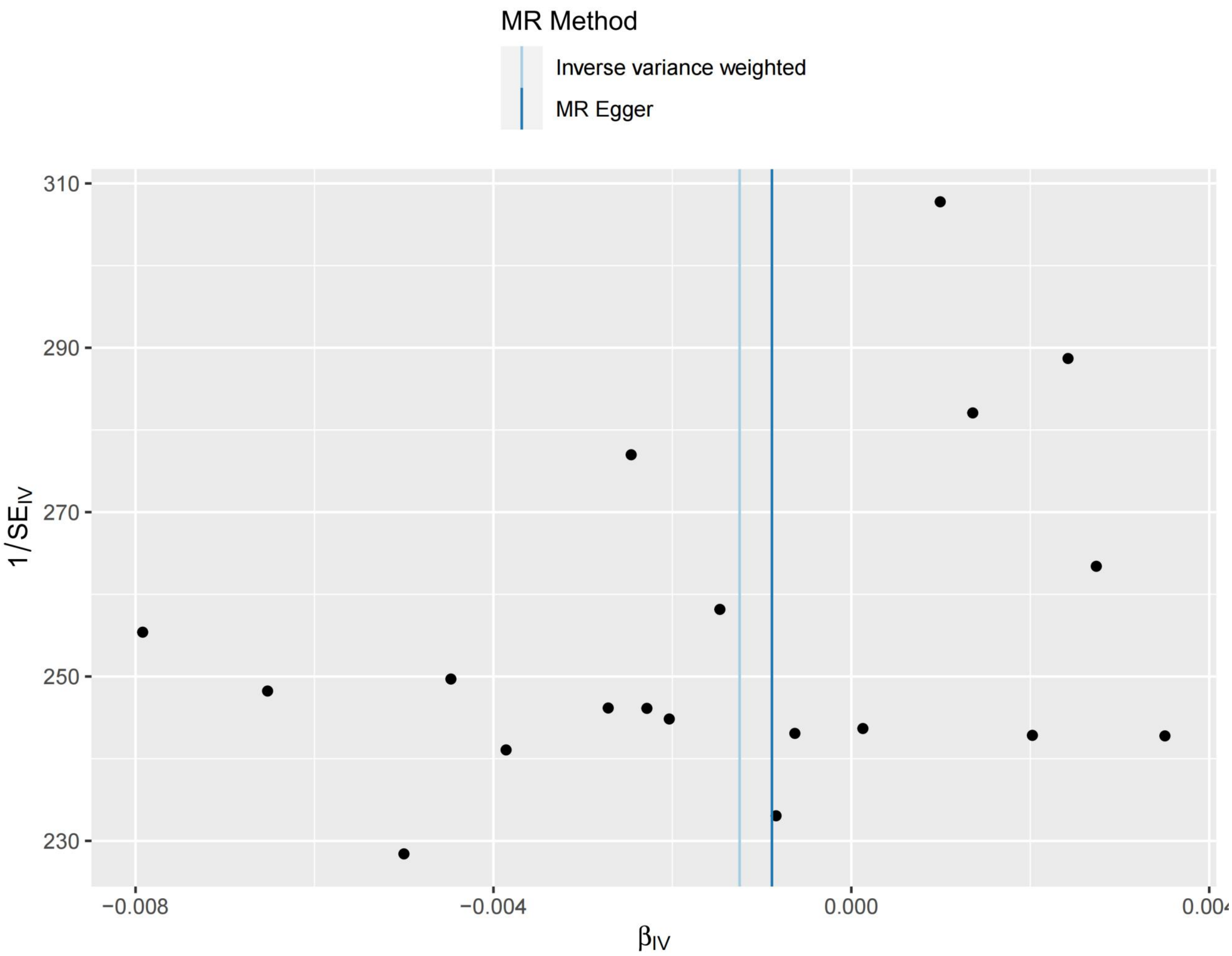

Supplementary Figure-63 Leave-one-out Analysis, Scatter Plot, Forest Plot, and Funnel Plot of Vestibular Schwannomas on DNA Methylation GrimAge Acceleration

Supplementary Figure-63A Leave-one-out Analysis

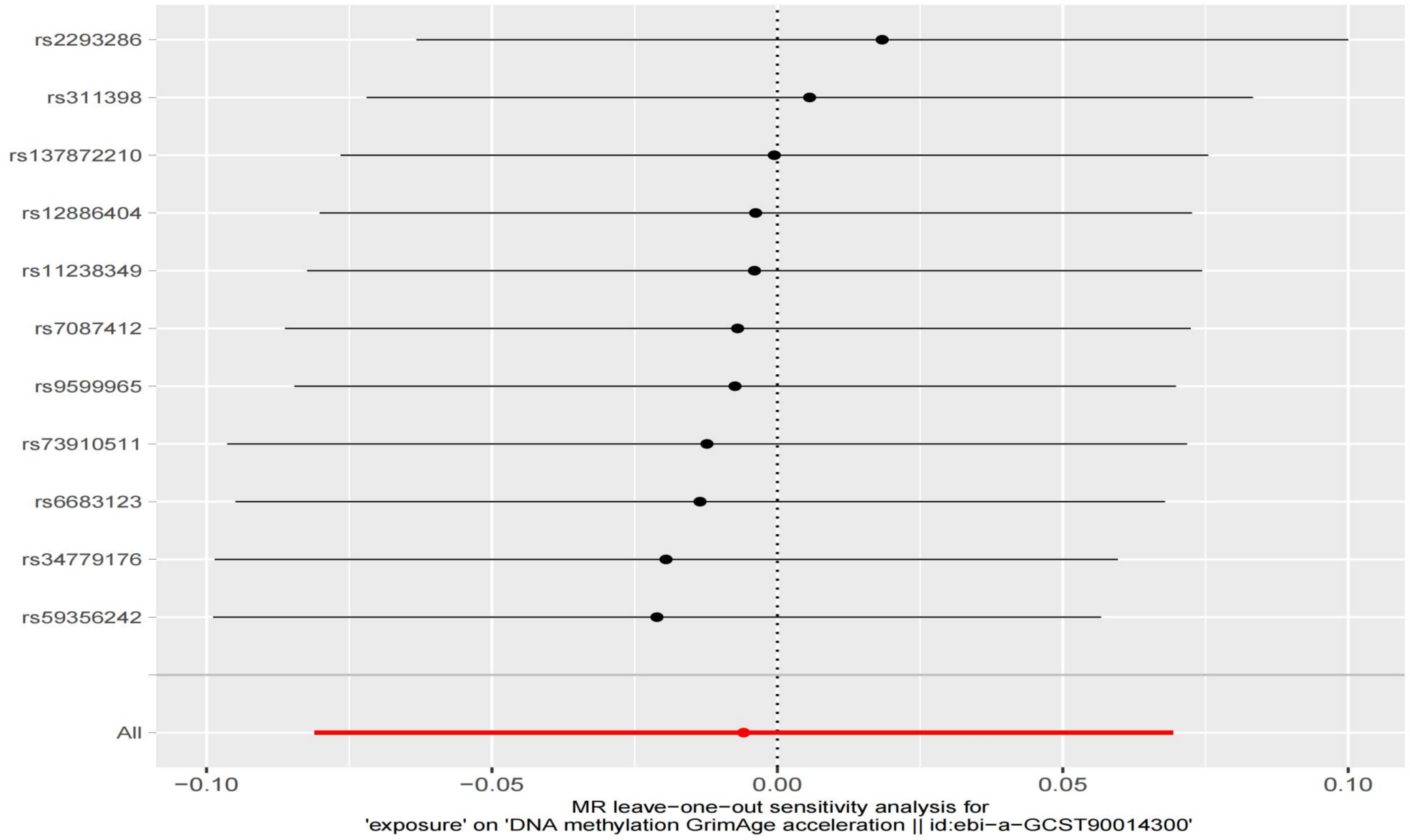

Supplementary Figure-63B Scatter

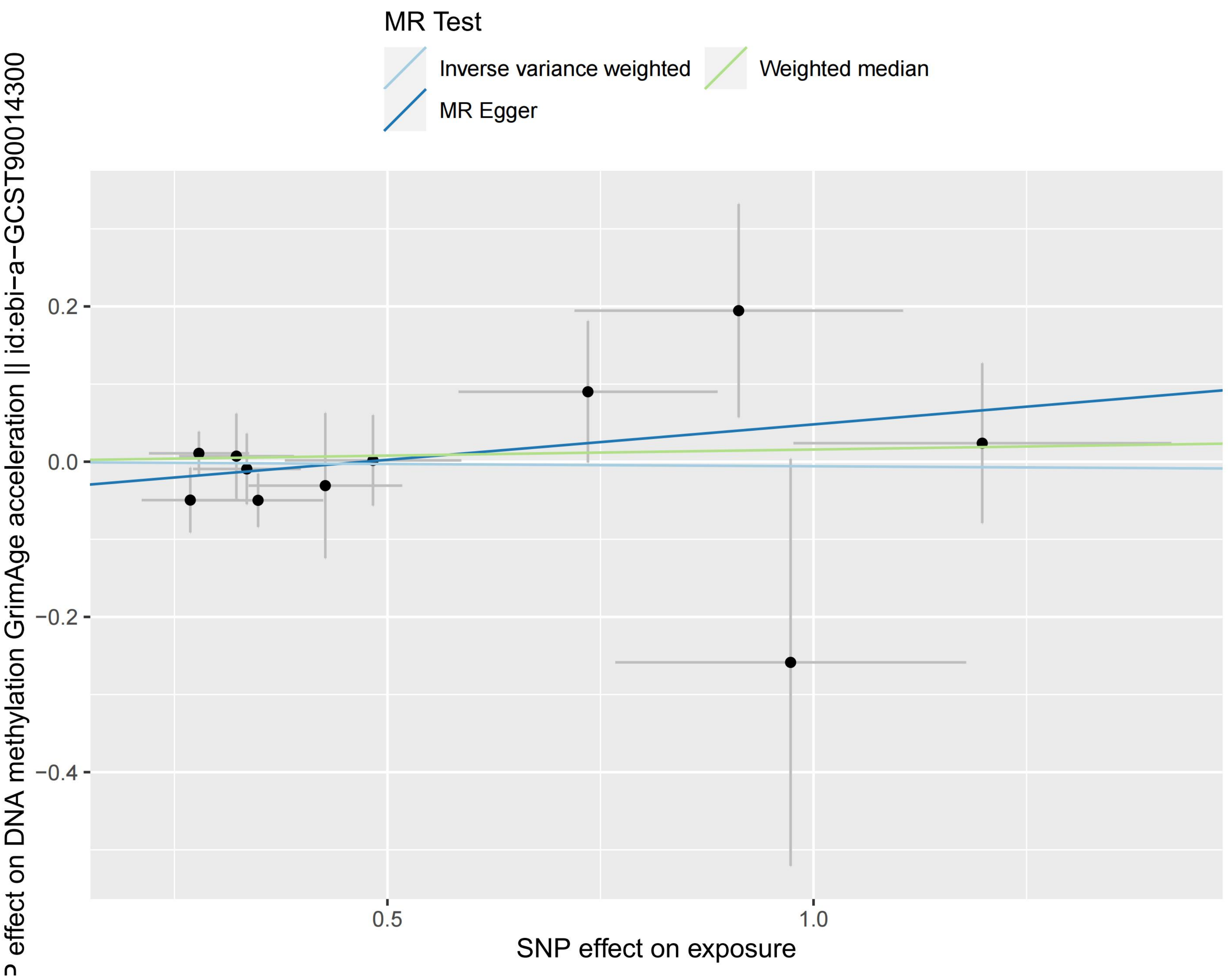

Supplementary Figure-63C Forest Plot

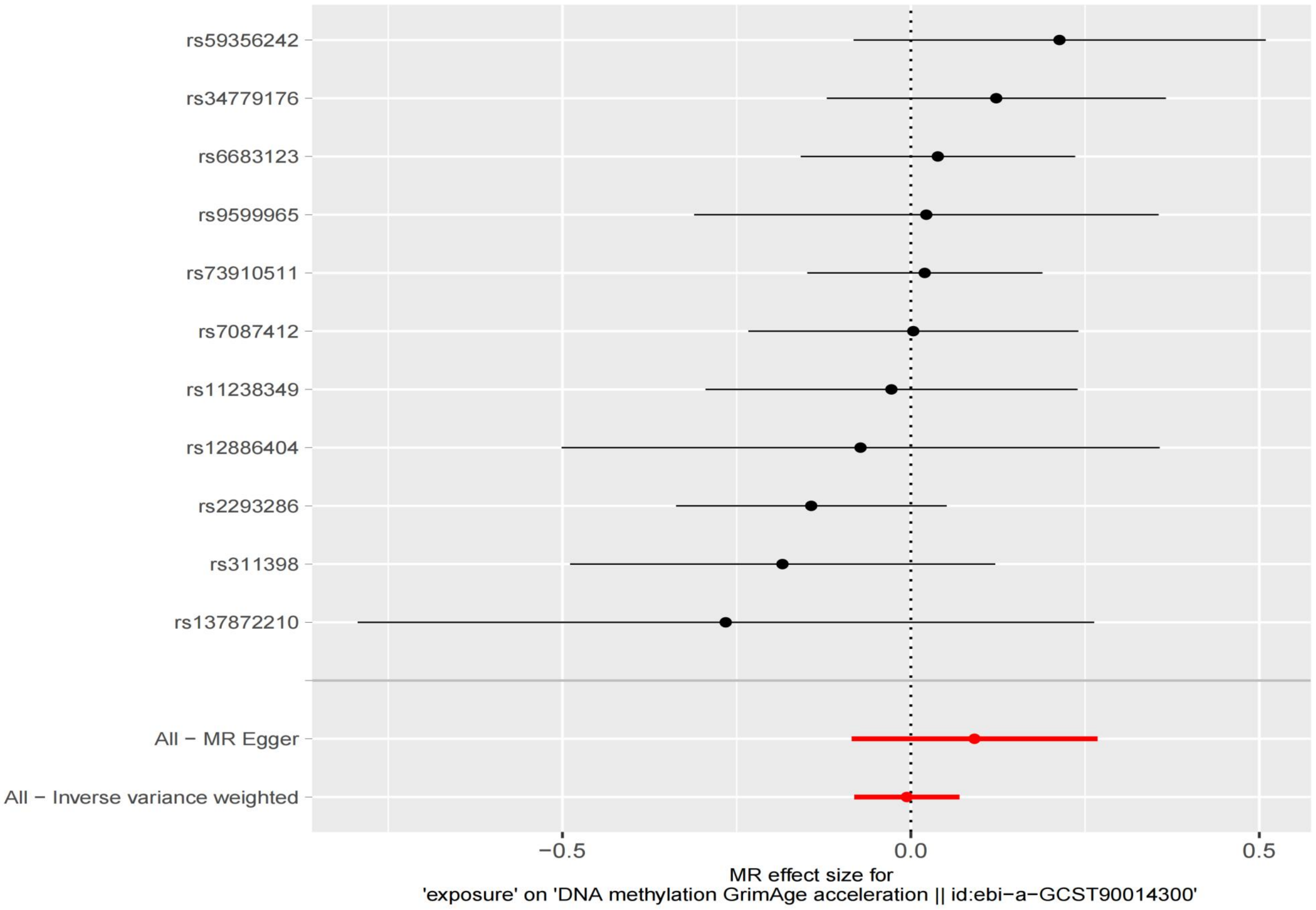

Supplementary Figure-63D Funnel Plot

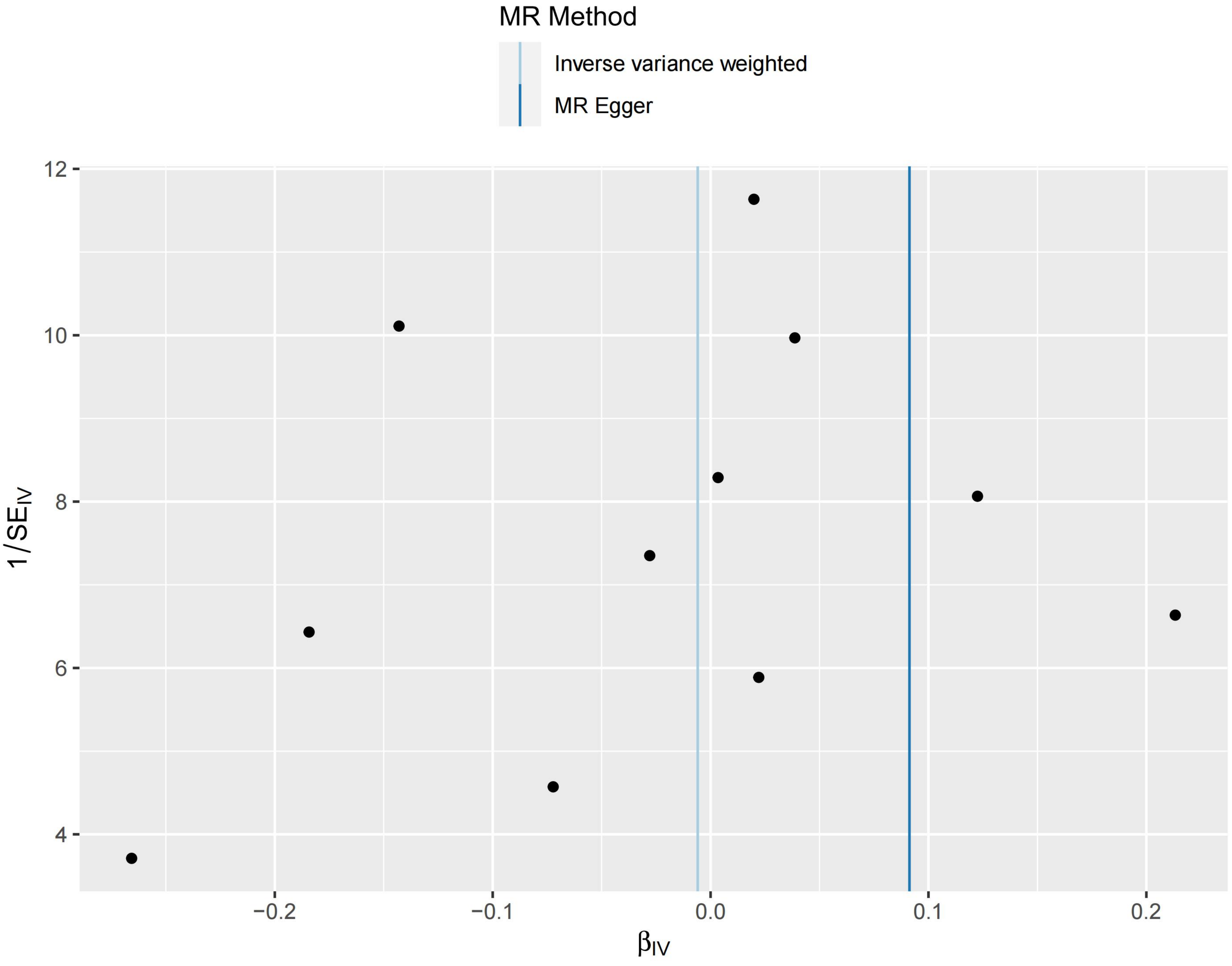

Supplementary Figure-64 Leave-one-out Analysis, Scatter Plot, Forest Plot, and Funnel Plot of Vestibular Schwannomas on Telomere length

Supplementary Figure-64A Leave-one-out Analysis

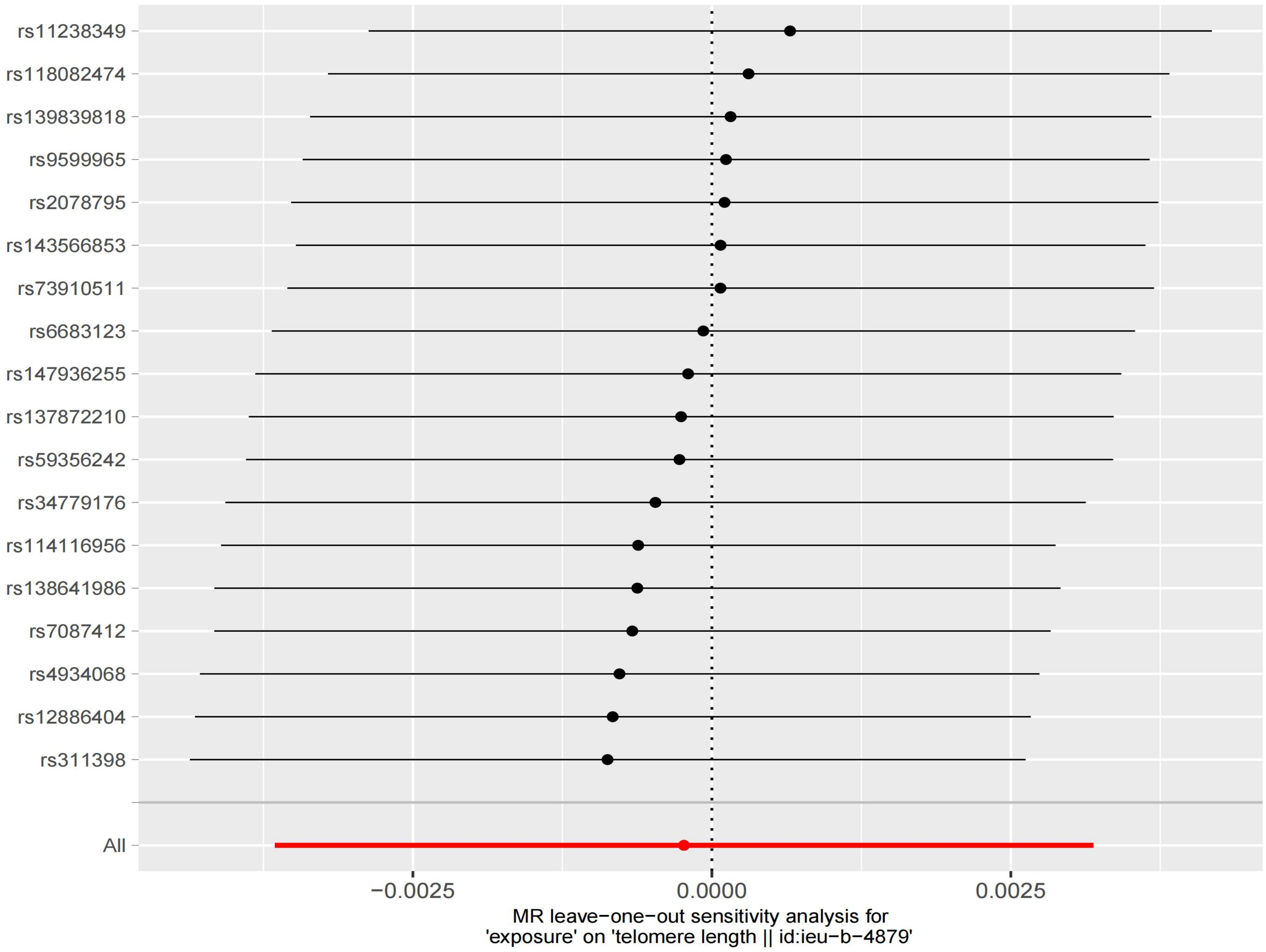

Supplementary Figure-64B Scatter

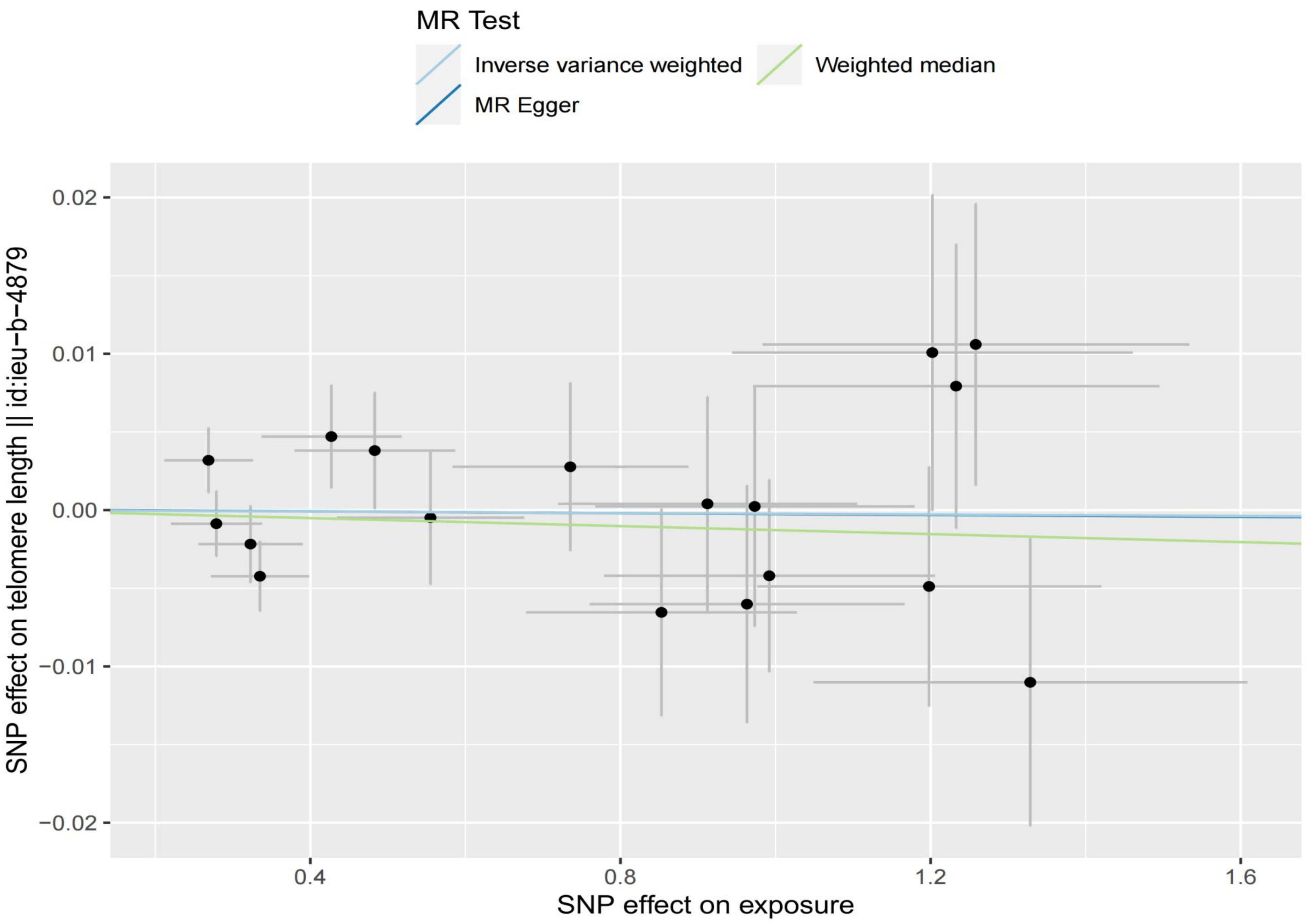

Supplementary Figure-64C Forest Plot

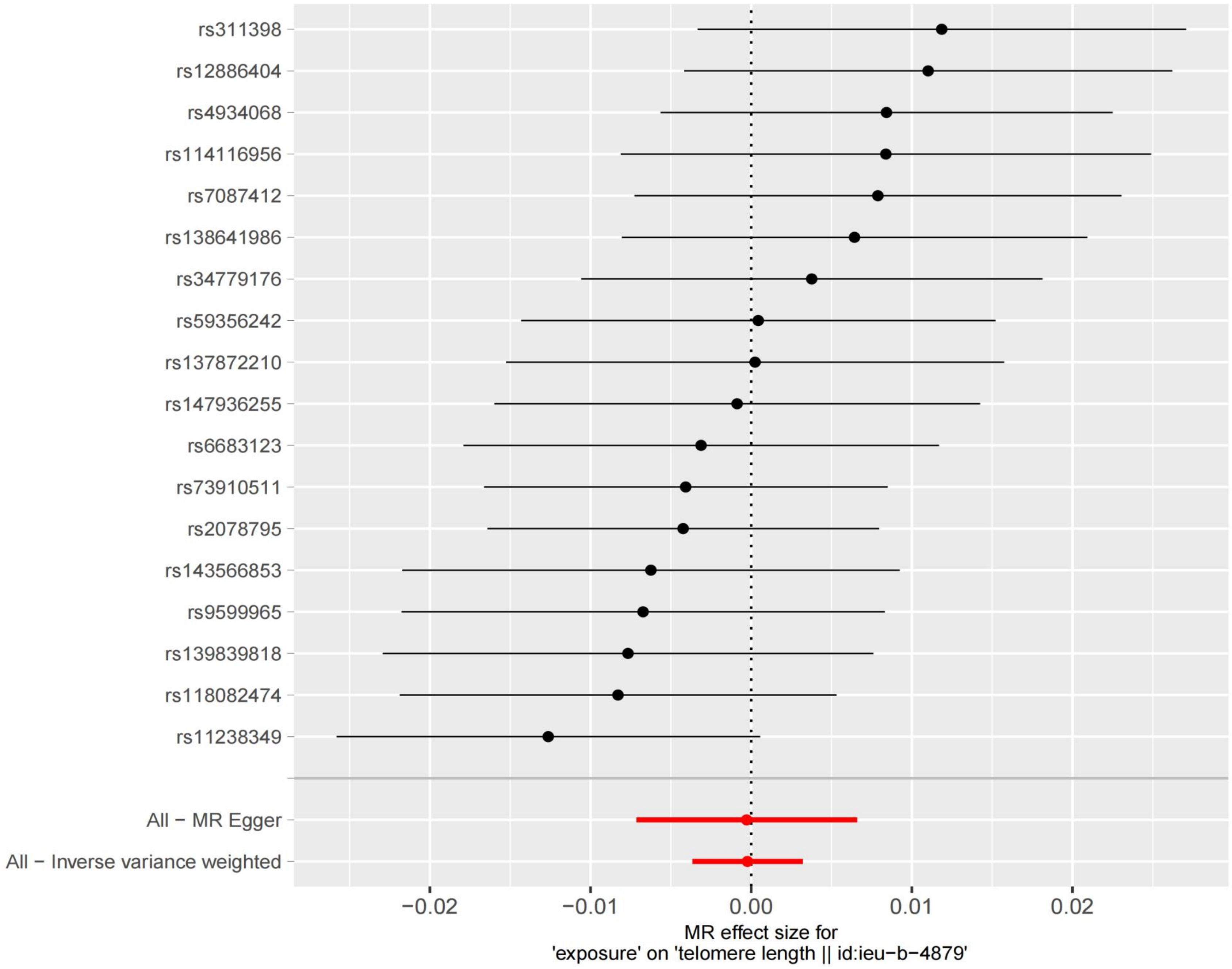

Supplementary Figure-64D Funnel Plot

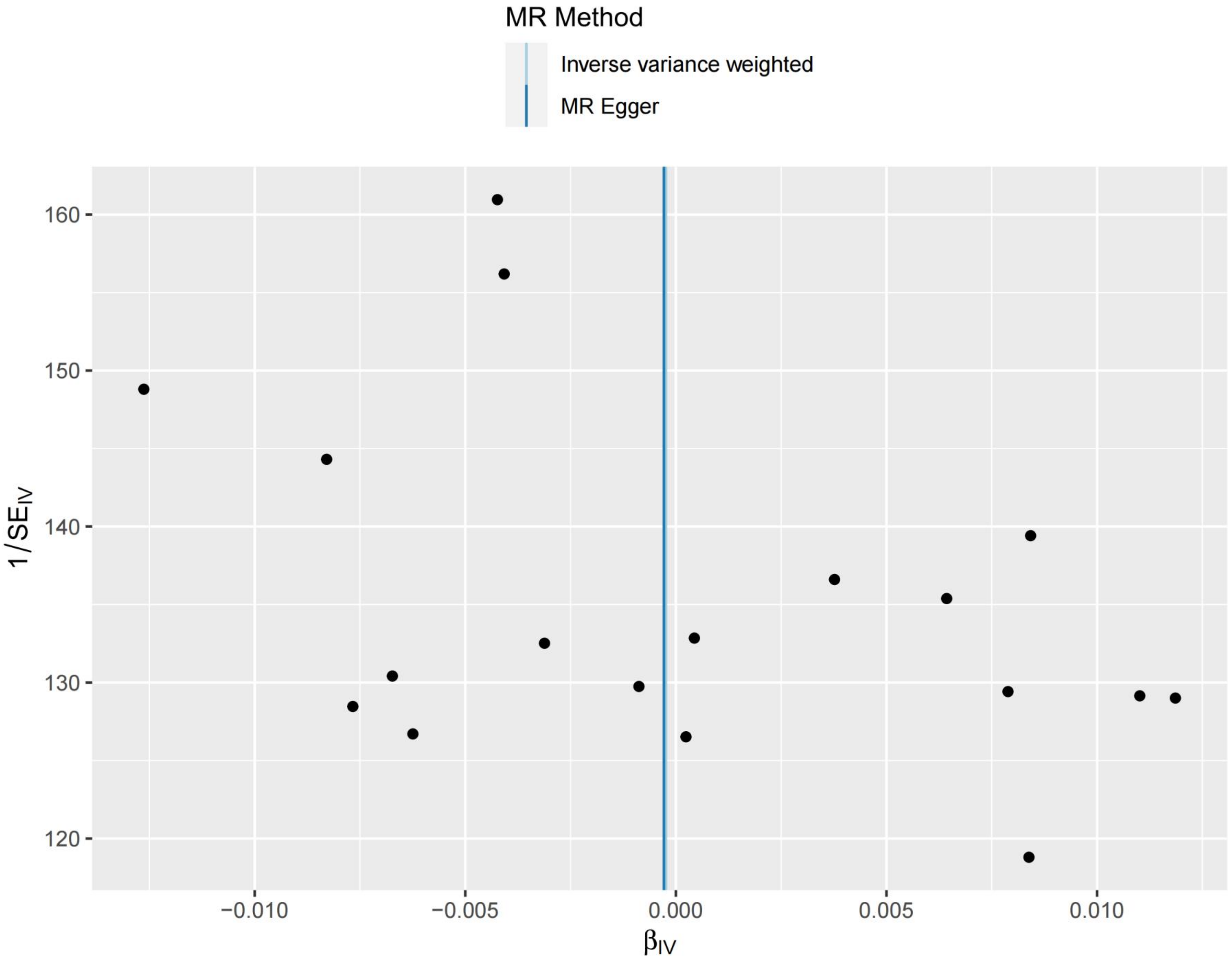

Supplementary Figure-65 Leave-one-out Analysis, Scatter Plot, Forest Plot, and Funnel Plot of Vestibular Schwannoma on Frailty Index

Supplementary Figure-65A Leave-one-out Analysis

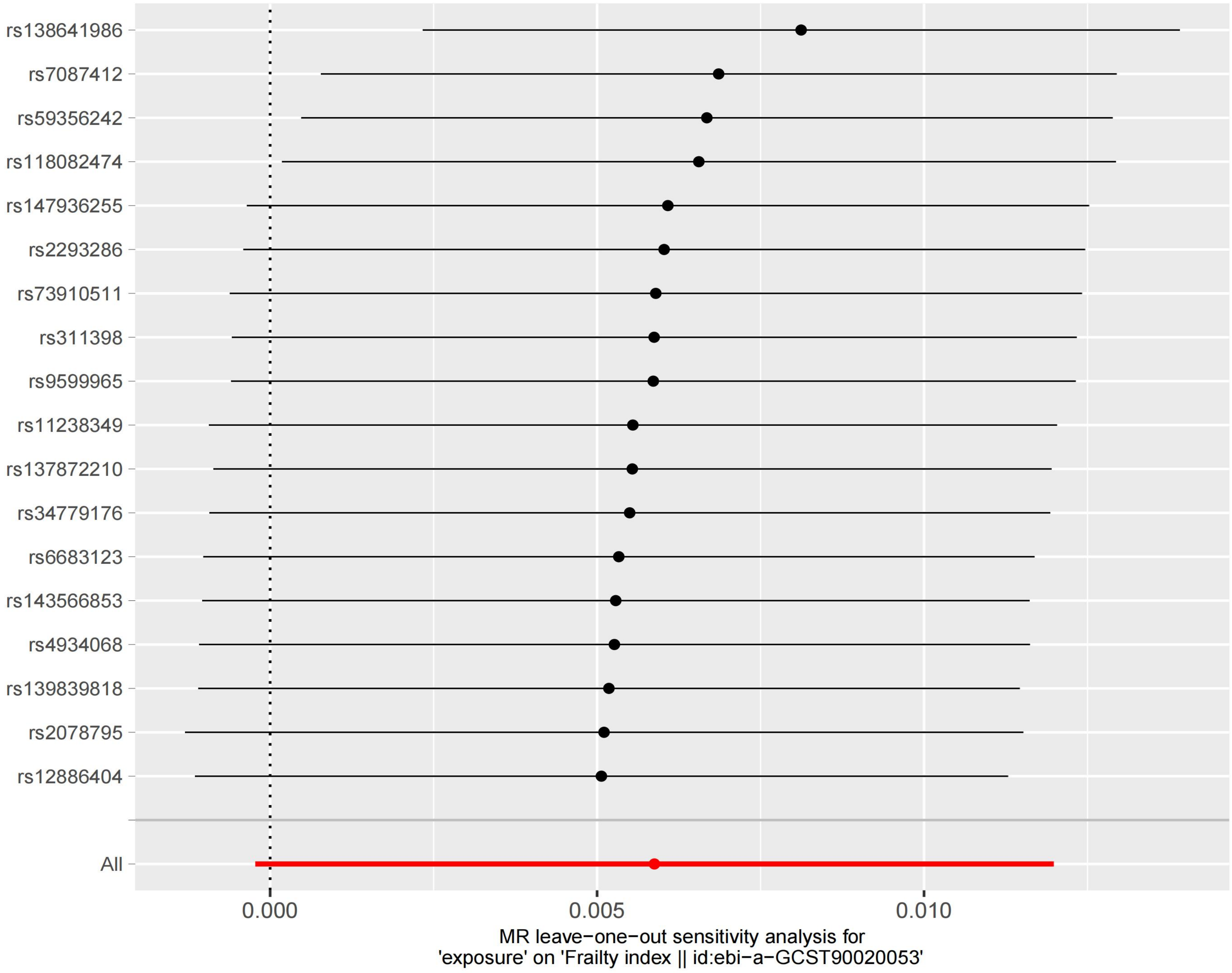

Supplementary Figure-65B Scatter

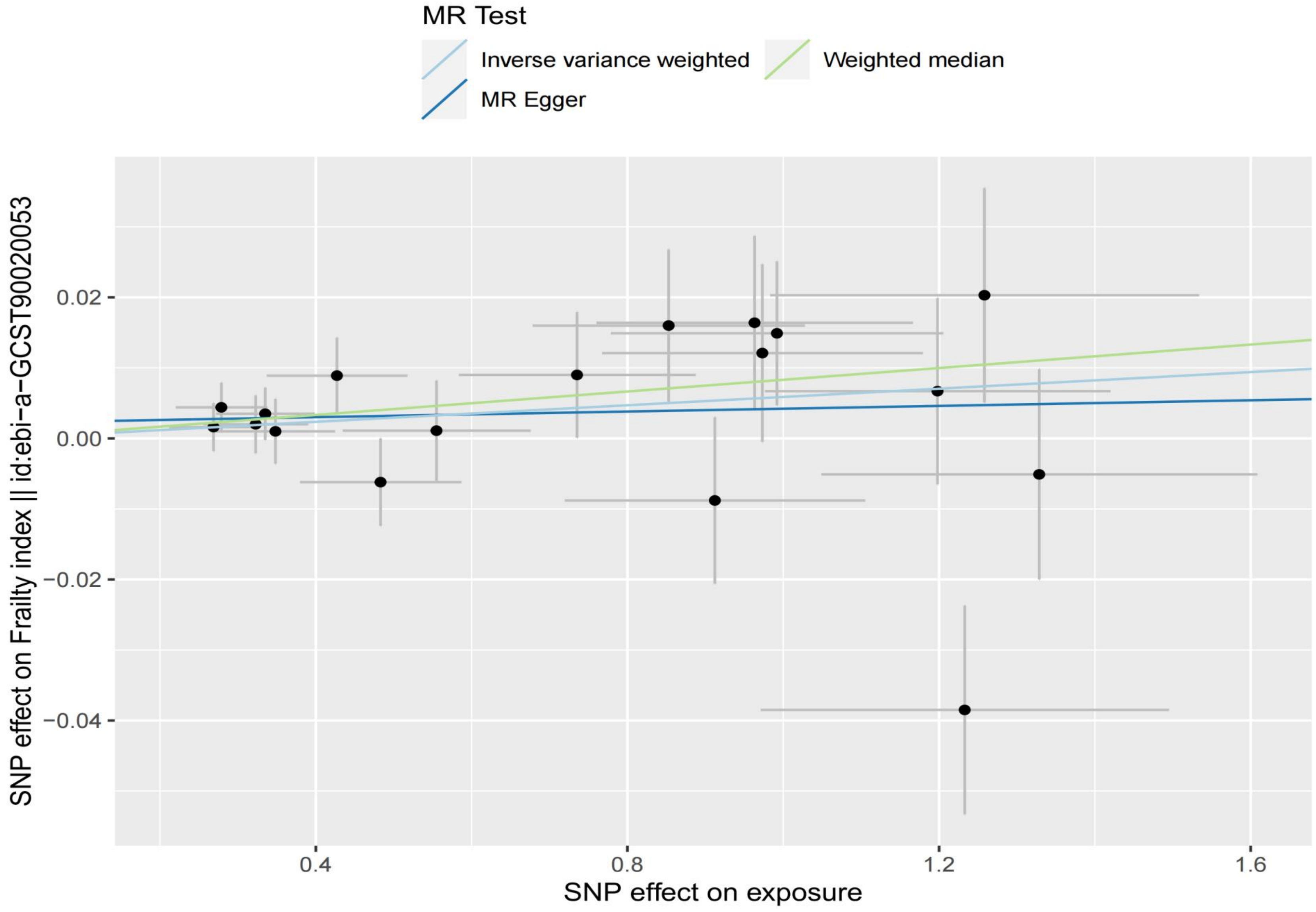

Supplementary Figure-65C Forest Plot

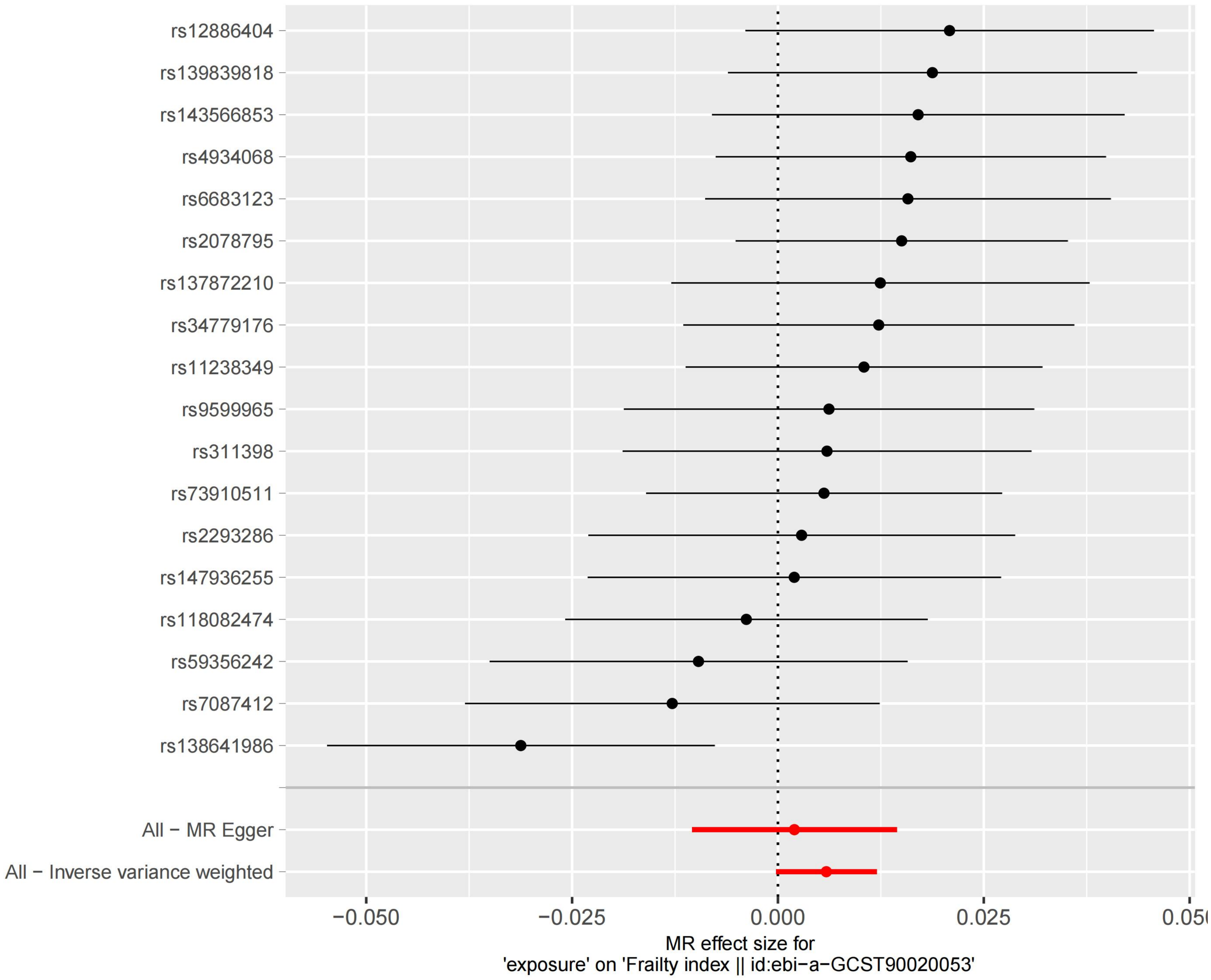

Supplementary Figure-65D Funnel Plot

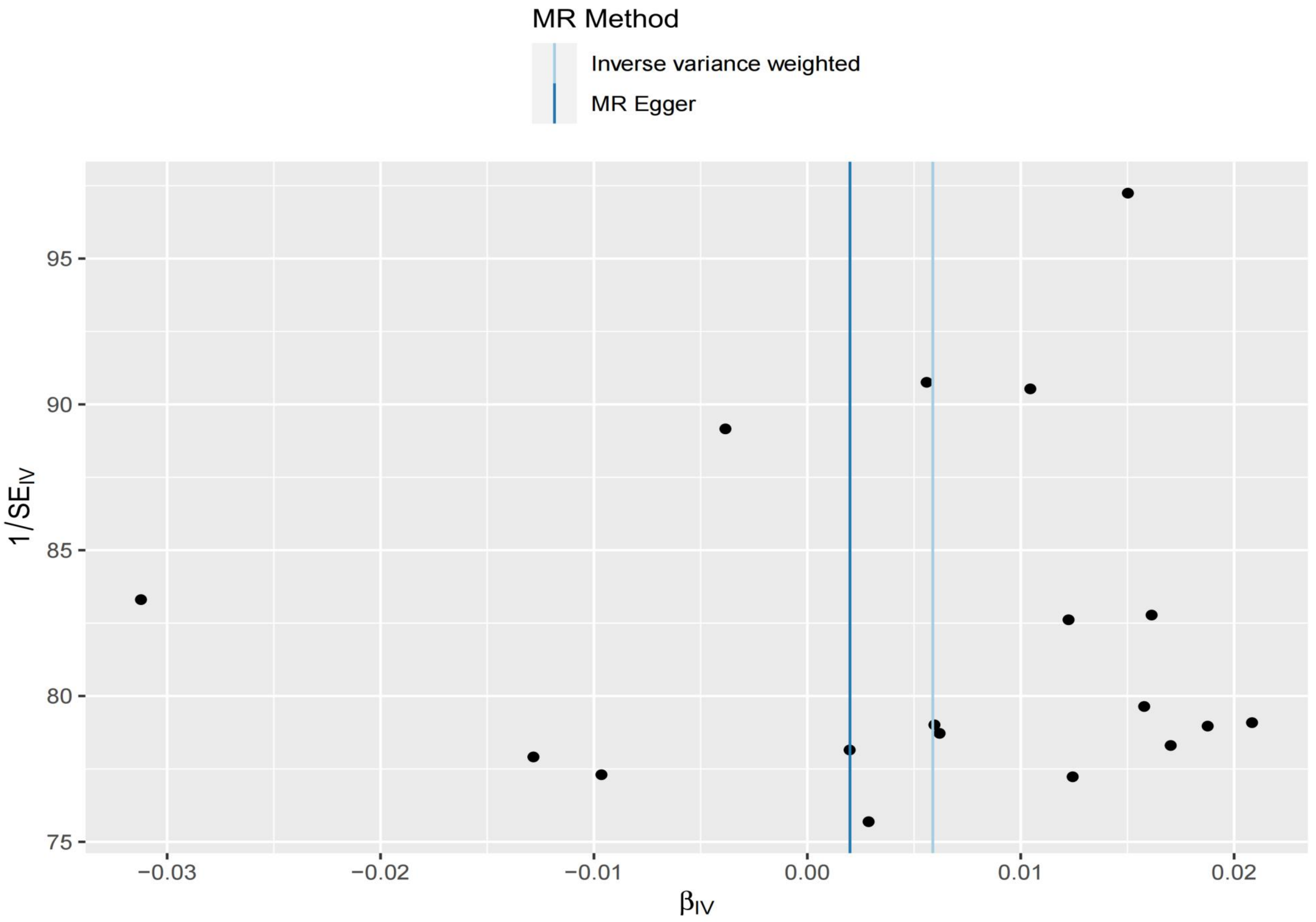

Supplement: Supplementary file 1 [file Data_Sheet_1.PDF]
